# Based on the Network Pharmacology to Investigate the Mechanism of Qingjie Fuzheng Granules against Colorectal Cancer

**DOI:** 10.1155/2022/7242640

**Published:** 2022-03-04

**Authors:** Yi Fang, Chi Yang, Yao Lu, Lihui Wei, Jinyan Zhao, Lisha Lu, Jiumao Lin

**Affiliations:** ^1^Academy of Integrative Medicine, Fujian University of Traditional Chinese Medicine, Fuzhou, Fujian 350122, China; ^2^Fujian Key Laboratory of Integrative Medicine on Geriatrics, Fujian University of Traditional Chinese Medicine, Fuzhou, Fujian 350122, China; ^3^Key Laboratory of Integrative Medicine of Fujian Province University, Fujian University of Traditional Chinese Medicine, Fuzhou, Fujian 350122, China; ^4^Institute of Edible Fungi, Fujian Academy of Agricultural Sciences, Fuzhou 350003, China; ^5^Oncology Department, Affiliated People's Hospital of Fujian University of Traditional Chinese Medicine, Fuzhou, Fujian 350004, China

## Abstract

Qingjie Fuzheng granules (QFG) exert an anticancer effect against colorectal cancers (CRC). However, the pharmacological molecular mechanisms are still unclear. This study was aimed to establish a simple method to predict targets of QFG against CRC by the network pharmacology strategy. 461 compounds and 1559 targets in QFG were enriched by BATMAN-TCM. 21 of the common targets were obtained by the groups of “Jun,” “Chen,” “Zuo,” and “Shi” medicine in QFG. The enrichment analyses of GO functional terms, KEGG pathway, and OMIM/TTD diseases displayed the targets in the different and complementary effects of four functional medicines in QFG. Then, 613 differential targets for QFG in CRC were identified. GO functional terms and KEGG pathway analyses showed that QFG regulated the inflammatory function and lipid metabolic process. There were also targets that played a role in the binding to the receptors in membranes, in the activation of the transportation signal, and provided pain relief by regulation of the neural related pathways. Next, the protein-protein interaction network was analyzed, and the levels of the predicted targets in CRC primary tumor were explored, and 7 candidate targets of QFG against CRC were obtained. Furthermore, with real-time PCR and enzyme-linked immunosorbent assay (ELISA) analysis, downregulation of dopamine D2 receptor (DRD2) and interleukin-6 (IL-6), and upregulation of interleukin-10 (IL-10) were identified following the treatment of QFG. At last, the survival and prognosis of the potential targets of QFG in CRC patients were analyzed by GenomicScape, and IL-6 was suggested to be an index for the regulation of QFG in CRC. These results might elucidate the possible antitumor mechanism of QFG and highlight the candidate therapeutic targets and the application direction in clinical treatment for QFG.

## 1. Introduction

Colorectal cancer (CRC) is the third most commonly diagnosed cancer. It is expected to increase the global burden of cancer by 60% to more than 2.2 million new cases and 1.1 million deaths in 2030 [1]. Due to the poor clinical outcomes, it is indispensable to investigate novel biomarkers for the diagnosis and prognosis of CRC. The conventional treatment of CRC is the surgical resection combined with radiation therapy and chemotherapy [2, 3]. Nevertheless, new chemotherapeutic resistance or unacceptable side effects can still occur. Therefore, searching for a new safe and acceptable resistance reversal medication would be of tremendous value.

Traditional Chinese medicine (TCM) had been widely used in China and proved to be effective in the prevention and treatment of various diseases. TCM also has a great advantage because of its relatively fewer adverse effects. Qingjie Fuzheng granule (QFG) is a Chinese medicinal formula comprising of *Hedyotis diffusa* Willd, Malt, Astragalus, *Scutellaria barbata* D. Don, *Codonopsis pilosula,* and *Glycyrrhiza uralensis* Fisch, which is widely used as an alternative medicine for the clinical treatment of various cancers with few side effects, including colon cancer [4]. In this formula, *Hedyotis diffusa* Willd, *Scutellaria barbata*, Astragalus, *Codonopsis pilosula,* and *Glycyrrhiza uralensis* Fisch were also wildly known with antioxidative, anti-inflammation, and antitumor activity [5–10]. Our previous studies found that QFG combined with mFOLFOX4 in the treatment of advanced colorectal cancer could improve the levels of tumor remission and the immune function, depress the expression levels of the tumor markers of CEA and CA199, and improve the life quality of patients [11]. QFG can reduce the intestinal mucositis and diarrhea induced by 5-FU, inhibit cell growth, induce apoptosis, and regulate cellular immune function in hepatoma cells [12–14]. QFG also depressed CRC cell proliferation by the induction of apoptosis through suppressing the PI3K/AKT and ERK signaling pathways, inhibition of the migration of CRC cells by lncRNA ANRIL/let-7a/TGF-*β*1/Smad Axis, induction of autophagy via mTOR signaling pathway, regulation of tumor angiogenesis of CRC xenograft mice by Sonic Hedgehog pathway, and suppression of lymphangiogenesis via VEGF-C/VEGFR-3-dependent PI3K/AKT pathway [15–19]. However, the underlying mechanism and molecular signaling pathways involved in QFG activity remain to be further explored.

In recent years, more and more pieces of evidences indicated that combining target prediction of herbal ingredients and subsequent network pharmacology analyses was a feasible and powerful way to analyze the molecular mechanism of TCM. In the present study, BATMAN-TCM online database was applied to predict the potential targets in QFG. BATMAN-TCM (http://bionet.ncpsb.org/batman-tcm/) is an online bioinformatics analysis tool, and the core idea of this method is ranking potential drug-target interactions based on their similarity to the known drug-target interactions [20]. Then, integrating with differentially expressed genes (DEGs) of CRC in the Cancer Genome Atlas (TCGA), disease and pathway enrichment analysis were identified via the WebGestalt [21]. Next, 9 targets were obtained by the protein-protein interaction (PPI) network enriched via the STRING database and Cytoscape [22, 23]. By analyzing the levels of 9 potential genes in CRC, 7 targets of AGT, DRD2, IL-1B, IL-6, ALB, IL-10, and IGF1 were obtained. Finally, real-time PCR, ELISA, and the survival and prognosis of the potential targets were applied to validate preliminarily to elucidate the potential targets in the mechanism of action of QFG.

## 2. Materials and Methods

### 2.1. Target Prediction

The network pharmacology of QFG was analyzed based on BATMAN-TCM [24]. 6 herbs of QFG were submitted, and the predicted candidate targets with scores not smaller than cutoff score = 20 and *P* value <0.05 for each ingredient were presented.

### 2.2. DEGs Screening of COAD

The RNA sequencing profile data of COAD were downloaded from the Cancer Genome Atlas (TCGA) database (https://portal.gdc.cancer.gov) and analyzed using SangerBox (http://ask.sangerbox.com/). Genes with an adjusted *P* < 0.05 and |log_2_ fold change (FC)| > 1 were considered DEGs. These DEGs were integrated with the targets using an online Venn webtool (http://bioinformatics.psb.ugent.be/webtools/Venn/).

### 2.3. Functional Enrichment Analysis

An overrepresentation enrichment analysis (ORA) in Gene Ontology (GO) terms and Kyoto Encyclopedia of Genes and Genomes (KEGG) pathway of the coexpressed genes were performed by WebGestalt 2019 (WEB-based GEne SeT AnaLysis Toolkit) [21]. *P* < 0.05 was considered as a significant difference.

### 2.4. Expression Levels in CRC Analysis

The expression levels of the targets in primary tumor with the normal samples in CRC were analyzed to use a database of UALCAN (http://ualcan.path.uab.edu/). *P* < 0.05 was considered as a significant difference.

### 2.5. Protein-Protein Interaction Network

Search Tool for the Retrieval of Interacting Genes (STRING; version 11.0; https://string-db.org/) [22] is an online tool to explore potential protein-protein interactions (PPIs), which consists of known and predicted PPIs. The interactions among DEGs were screened from the STRING database, and a combined score >0.4 was set as the threshold to select the significant interactions to visualize the PPI network using Cytoscape software V3.7.2. CytoNCA V2.1.6 was utilized to identify crucial genes in the network [25]. The crucial genes in this study were identified based on eigenvector centrality (EGC), degree centrality (DC), betweenness centrality (BC), and closeness centrality (CC). Common genes in the four different centrality measures were integrated with the targets using an online webtool, Venn diagrams (http://bioinformatics.psb.ugent.be/webtools/Venn/).

### 2.6. Treatment of QFG in Cells

Qingjie Fuzheng granule was provided by the Academy of Pharmacy of Fujian University of Traditional Chinese Medicine (Fuzhou, Fujian, China) and dissolved at a concentration of 10 mg/mL in PBS. Colorectal cancer cells (HCT-116 and HCT-8) were cultured in 1640 medium with 10% fetal bovine serum, 100 *µ*g/mL streptomycin, and 100 U/mL of penicillin. All the reagents used in cell culture were purchased from Hyclone (Carlsbad, CA, USA). The cells were seeded into a 6-well culture plate at a density of 1 × 10^5^ cells/mL. After 12 h, the cells were treated by QFG (0.5, 1, and 2 mg/mL) for 24 h (2 mL/well), and the control group was treated with PBS. Then, cell morphology was observed and photographed at a magnification of ×100 by a phase-contrast microscope (Olympus, Tokyo, Japan).

### 2.7. Detection of Potential Targets by Real-Time PCR

After cell treatment with QFG, the RNA of cells was isolated with Trizol (Life Technologies Corporation, Shanghai, China). Reverse transcription reaction was carried out using PrimeScript™ RT reagent Kit with gDNA Eraser (Perfect Real Time) obtained from Takara (Dalian, China), and real-time PCR amplification was carried out with SYBR Select Master Mix (Life Technologies Corporation, Shanghai, China). Primers for genes were obtained for Primerbank (PrimerBank (harvard.edu).

### 2.8. Analysis of Potential Targets by Enzyme-Linked Immunosorbent Assay (ELISA)

The tumor xenograft model was established by subcutaneous inoculation of CT-26 cells, which were murine cells in BALB/c mice ((weight: ∼18–20 g)), and gavage with 1 g/kg of QFG for 3 weeks, and then, the serum was collected as previously described by Zhang et al. [26]. The levels of AGT, DRD2, IL-1B, IL-6, ALB, IL-10, and IGF1 were detected according to the manufacturers' instructions (Jiangsu Meimian Industrial Co., Ltd. and Nanjing Jianchen Bioengineering Institute Co., Ltd., China).

## 3. Results

### 3.1. Target Prediction of QFG by Bioinformatics

QFG was composed of 6 herbs, including *Scutellaria Barbata* [Syn. Scutellaria Rivularis] (Banzhilian), *Codonopsis Pilosula* (Dangshen), *Glycyrrhiza uralensis* (Gancao), *Astragalus Membranaceus* (Huangqi), *Oldenlandia Diffusa* [Syn. Hedyotis Diffusa] (Baihuasheshecao), and *Fructus hordei* Germinatus Preparata (Chaomaiya) (Table 1). By the analysis of these 6 herbs in BATMAN-TCM, 461 compounds and 1559 potential targets were predicted (S1 Table).

**Figure 1:**
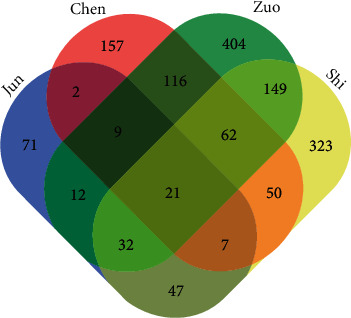
Venn diagrams of “Jun,” “Chen,” “Zuo,” and “Shi” medicines in QFG. The predicted candidate targets with scores not smaller than cutoff score = 20 and *P* value < 0.05 for each ingredient.

Next, the potential targets were split into groups according to the composition theory of a prescription in QFG. In the QFG, Scutellaria Barbata and Oldenlandia Diffusa were “Jun” (Monarch) medicine, Astragalus Membranaceus was the “Chen” (Minister) medicine, Codonopsis Pilosula and Fructus Hordei Germinatus Preparata were “zuo” (Assistant) medicine, and Glycyrrhiza Uralensis was “Shi” (Guide) medicine. The results showed that 21 of targets were common found in four functional herds, which were ANXA1, DRD3, ADRA2A, OPRK1, ATP1A1, NR3C1, DRD2, ESR1, GRIN2A, TRIM24, VDR, ADORA1, MED1, DRD4, PGR, NR1H4, RAPGEF2, IL-1B, WNT4, AR, and DRD1, respectively. These targets might have concentration superposition to enhance the effect of the component. In the same time, there were 71 targets in “Jun,” 157 targets in “Chen,” 404 targets in “Zuo,” and 323 targets in “Shi,” respectively, difference with other functions of herds. These targets regulated by the compounds might be complement with each other and play a synergistic role in QFG (Figure 1).

**Figure 2:**
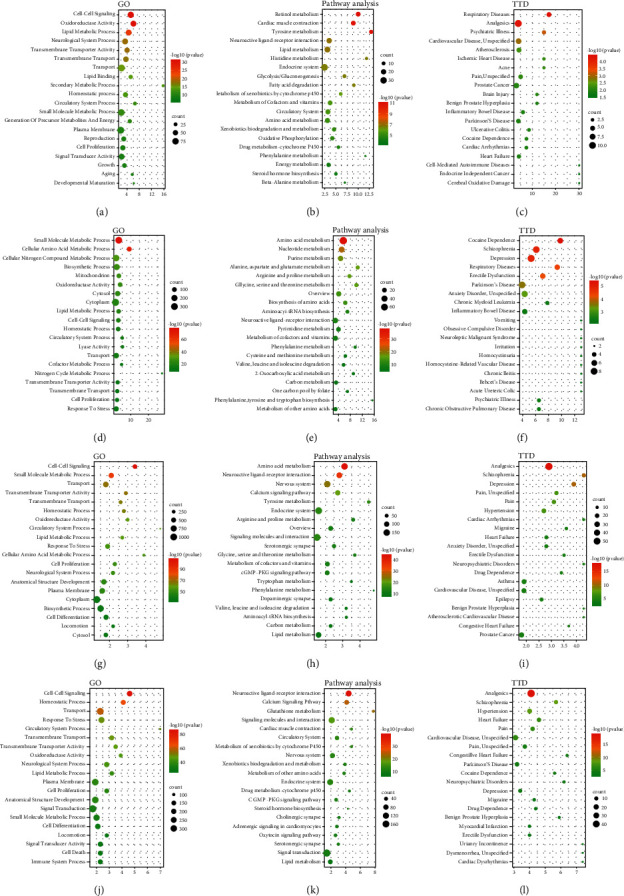
The term of GO analysis, KEGG pathway analysis, and OMIM/TTD disease enrichment (TTD) analysis of the “Jun,” “Chen,” “Zuo,” and “Shi” medicines of QFG in BATMAN-TCM. (a–c) represented the “Jun” medicine, (d–f) represented the “Chen” medicine, (g–i) represented the “Zuo” medicine, and (j–l) represented the “Shi” medicine. All analyses displayed for the top of 20 functions, pathways, or disease enrichments, respectively. The *x*-axis shows the enrichment of scores of the targets (*P* < 0.05). The compound-target work and candidate compounds.

Furthermore, we explored the term of GO analysis, KEGG pathway analysis, and OMIM/TTD disease enrichment (TTD) analysis of the “Jun,” “Chen,” “Zuo,” and “Shi” medicines of QFG in BATMAN-TCM. The results showed that both “Jun” and “Chen” medicines got involved in small molecule metabolic process, lipid metabolic process, cell proliferation, cell-cell signaling, transmembrane transporter activity, and transmembrane transport in GO-term analysis, which suggested these functions were the mainly function of QFG (Figures 2(a) and 2(d)). In the pathway analysis, steroid hormone biosynthesis was only found in “Jun” medicine, which suggested that the “Jun” medicine plays an important role in maintaining life function and immune regulation by the synthesis of steroid hormone (Figure 2(b)). Drug metabolism by cytochrome P450 was found in “Jun” and “Shi” medicines, which indicated that two kinds of these medicines in the prescription of QFG play the role in drug efficacy and toxicity change (Figures 2(b) and 2(k)). The “Jun” medicine was mainly involved in lipid or fatty acid metabolism pathway (Figure 2(b)). “Chen” and “Shi” medicines were mainly involved in different kinds of amino acid metabolism and carbon metabolism (Figures 2(e) and 2(k)). Moreover, both “Zuo” and “Shi” medicines were involved in nervous system relative pathways, such as the cGMP-PKG signaling pathway, which mediates the action of nitric oxide (NO) and natriuretic peptides, and the neuroactive ligand-receptor interaction (Figures 2(h) and 2(k)). In addition, they also regulated the nervous system by a complementary actin; for instance, “Zuo” medicine took part in the dopaminergic synapse, and “Shi” medicine mediated cholinergic synapse (Figures 2(h) and 2(k)). Additionally, the lipid metabolism and nervous ligand-receptor interaction were found in “Jun,” “Zuo,” and “Shi” medicines (Figures 2(b), 2(h) and 2(k)), while the TTD analysis showed that “Jun,” “Zuo,” and “Shi” medicines played roles in “Pain” disease (Figures 2(c), 2(i), and 2(l)), suggesting that these three kinds of medicines in QFG exert the analgesic effect for cancer patients. Furthermore, the analgesic effect might be involved in the lipid metabolism and nervous ligand-receptor interaction.

**Figure 3:**
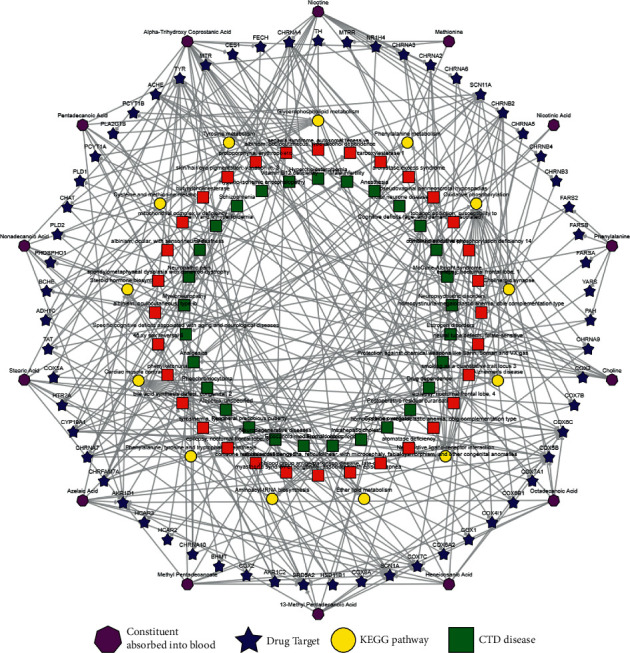
C-T network of QFG. C-T network of the predicted compounds and targets in QFG. The predicted candidate targets with scores not smaller than cutoff score = 500 and *P* value <0.05 for each ingredient.

The compound-target (C-T) work was obtained by the BATMAN-TCM (Figure 3). The C-T work contained four kinds of nodes distinguished with different shapes and colors including compound absorbed into blood, drug targets, biological pathways, and OMIM/TTD (Online Mendelian Inheritance in Man)/(Therapeutic Target Database) diseases. 14 compounds with cutoff score = 500 of targets in QFG were enriched. They were nicotine, nicotinic acid, methionine, alpha-trihydroxy coprostanic acid, pentadecanoic acid, nonadecanoic acid, stearic acid, azelaic acid, methyl pentadecanoate, 13-methyl pentadecanoic acid, heneicosanoic acid, octadecanoic acid, choline, and phenylalanine. Among these compounds, the precursor of acetylcholine, choline, possessed the antitumor effect [27]. Nicotine and nicotinic acid played a role in the central nervous system through its interactions with nicotinic acetylcholine receptors and various neurotransmitter releases. Alpha-trihydroxy coprostanic acid was one of the fecal short-chain fatty acids, which were known to exert the anti-inflammatory effect [28]. Azelaic acid plays the antioxidant and antitumor effect on various tumor cells [29]. Stearic acid was a benefit to anti-inflammatory, antioxidant, and antifibrotic effects [30–32].

### 3.2. Integrating Analysis with DEGs of COAD

**Figure 4:**
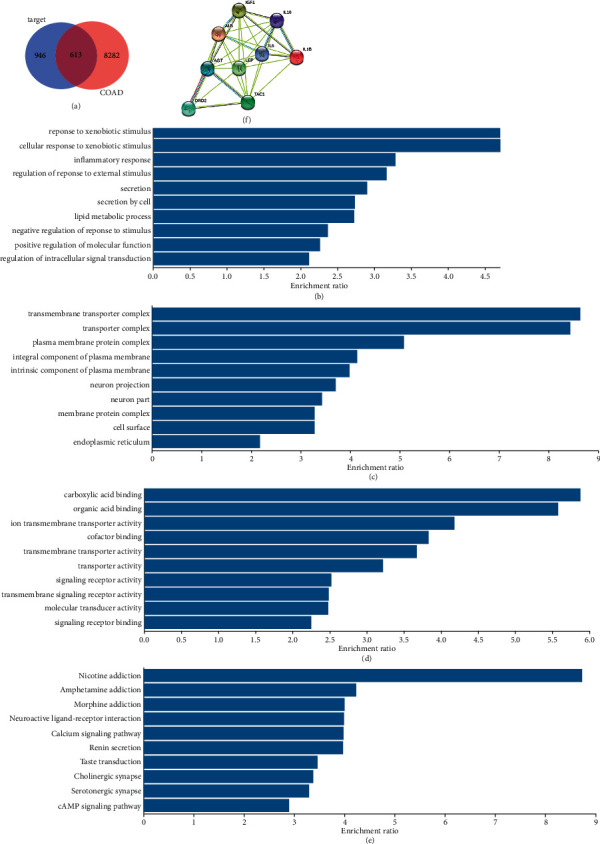
Analysis of the potential targets of QFG in COAD. (a) 613 of common DEGs were gotten among the targets of QFG in COAD. (b–d) GO analysis of the targets. The *y*-axis shows significantly enriched in “biological process” (b), “cellular component” (c), and “molecular function” (d) of the targets, and the *x*-axis shows the enrichment of scores of the targets (*P*  < 0.05). (e) KEGG analysis of the targets. The *x*-axis shows the enrichment of scores of the targets (*P*  < 0.05). (f) The PPI network of nine candidate targets of QFG.

Since we focused on the function of QFG in CRC, we searched the common targets of QFG in CRC. There were 480 cancer samples and 41 normal samples of COAD (colon adenocarcinoma) in the TCGA. A total of 8895 DEGs were identified, including 5192 upregulated genes and 3703 downregulated genes in COAD. Subsequently, we carried out Venn analysis to get the predicted targets differentially expressed in COAD. In total, 613 common DEGs were found (Figure 4(a)).

### 3.3. Functional Annotation of Common DEGs

The biological process in GO enrichment analysis was enriched in “response to xenobiotic stimulus,” “cellular response to xenobiotic stimulus,” “regulation of response to external stimulus,” and “positive regulation of molecular function” (Figure 4(b)), indicating that molecules in the QFG belonged to the xenobiotic stimulus. In addition, when the cells suffered from external stimulus, the processes of secretion might be activated, so the processes of “secretion” and “secretion by cell” were also significantly enriched. Recently, more and more evidence found that tumor microenvironment was crucial for the development of tumors. “Inflammatory response” and “lipid metabolic process,” which were two important processes in the tumor microenvironment [33, 34], were also found in the top of ten processes in the biological process. These results suggested that QFG might exert its effect on CRC from anti-inflammation and regulation of lipid metabolic.

In “cellular component” term of GO analysis (Figure 4(c)), in addition to the “neuron projection” and “neuron part,” the transportation process in membrane or endoplasmic was significantly enriched. When the transportation processes were active, the reaction between ligand and receptor must be active too. In the term of “molecular function,” three of transporter activity, receptor activity, molecular transducer activity, organic acid binding, and cofactor binding were enriched, indicating that the compounds in QFG might play the part as ligands and activate the signal transportation by binding to the receptor in membranes (Figure 4(d)).

KEGG pathway analysis found that the top 3 pathways enriched were nicotine addiction, amphetamine addiction, and morphine addiction (Figure 4(e)). Moreover, there were also neuroactive ligand-receptor interaction, cholinergic synapse, and serotonergic synapse pathways enriched in KEGG pathway. As we know, nicotine, amphetamine, and morphine can stimulate the nerve center, suggesting that using QFG for CRC patients might provide some pain relief somehow.

### 3.4. PPI Network of Common DEGs

To identify potential interactions between common DEGs, a PPI network was constructed by STRING tools, and then, the centrality values of genes in the PPI network were evaluated by CytoNCA V2.1.6. The top 20 ranked genes for each measure were identified as the crucial genes (Table 2). Nine common genes in these four measures were selected for further validation, including AGT, ALB, IL-1B, IL-10, DRD2, IGF1, LEP, IL-6, and TAC1 (Figure 4(f)).

### 3.5. Effect of QFG on the Expression Level of the Crucial Genes in CRC

**Figure 5:**
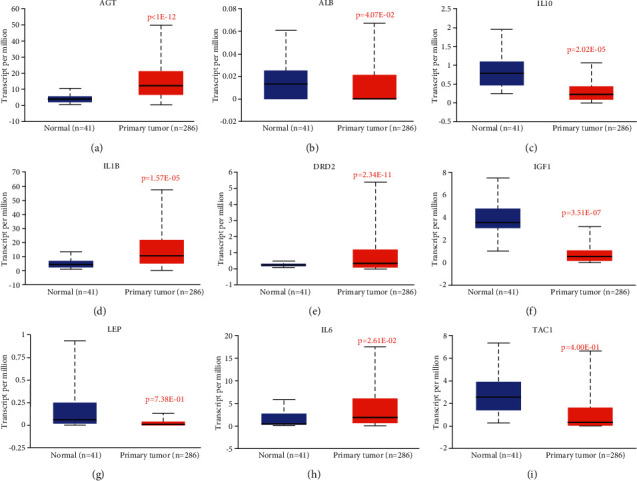
The expression levels of 9 potential targets in COAD (*P*  < 0.05).

Next, we compared the expression levels of the targets in primary tumor with the normal samples in CRC (UALCAN, http://ualcan.path.uab.edu/). Among these 9 candidate targets, AGT, DRD2, IL-1B, and IL-6 were significantly increased in primary tumor compared with the normal tissues, while ALB, IL-10, and IGF1 were significantly decreased in COAD tissues. However, LEP and TAC1 showed no significant difference compared with normal groups (Figure 5). So, we narrowed the candidate targets in the AGT, DRD2, IL-1B, IL-6, ALB, IL-10, and IGF1.

### 3.6. Identified the Candidate Targets by QFG

**Figure 6:**
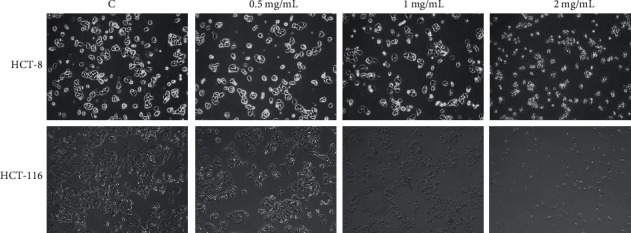
Cell morphology following the treatment with the indicated concentration of QFG for 24 h.

**Figure 7:**
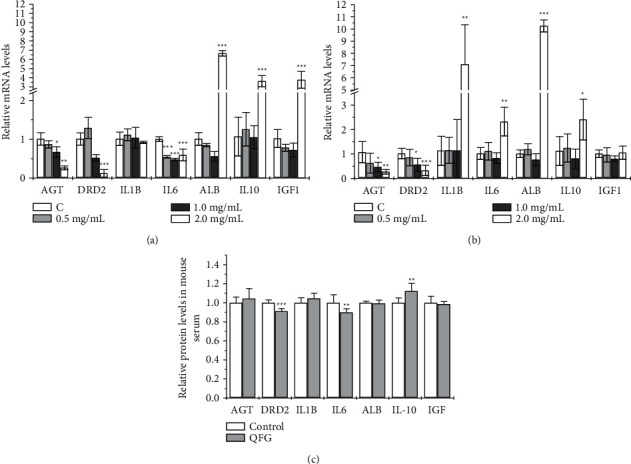
Detection of four potential targets in the treatment of QFG. The expression levels of AGT, DRD2, IL-1B, IL-6, ALB, IL-10, and IGF1 were detected by real-time PCR in HCT-8 (a) and HCT-116 (b) cells treated with different concentrations of QFG for 24 h (c). Protein levels of 7 targets were detected by ELISA in serum of mice with subcutaneous transplanted tumor treatment with QFG. ^*∗*^*P*  < 0.05, ^*∗∗*^*P* < 0.01, and ^*∗∗∗*^*P*  < 0.001, compared with the control group. The survival and prognosis of the potential targets of QFG in CRC patients were analyzed.

Our previous study found that the treatment of QFG could significantly suppress the cell viability of HCT-8 and HCT-116 cells [16], to further detect whether QFG would regulate these seven candidate targets, we analyzed the mRNA levels of AGT, DRD2, IL-1B, IL-6, ALB, IL-10, and IGF1 in HCT-8 and HCT-116 cells treated with QFG. Two cells were treated with different concentrations of QFG, light microscopy showed that the cell density was reduced, and cells were smaller and rounder with the concentration increase of QFG (Figure 6). As shown in Figure 7, AGT, DRD2, and IL-6 were downregulated in both two cells in the low concentrations of QFG (0.5 mg/mL and 1.0 mg/mL). Although IL-1B was not changed in HCT-8 cells, it was decreased in HCT-116 cells in the low concentrations of QFG (0.5 mg/mL and 1.0 mg/mL) too. In the treatment of 2.0 mg/mL of QFG in HCT-116 cells, both IL-1B and IL-6 were upregulated. Since both two genes were coding the proinflammatory cytokines, and our previous results showed that 2 mg/mL of QFG depressed the growth of HCT-8 and HCT-116 cells extremely significantly compared with cells in 0.5 mg/mL and 1.0 mg/mL of QFG [15], the results suggested that low concentrations of QFG would inhibit the growth of the cells by decreasing the expression of the inflammatory factors, while 2.0 mg/mL of QFG played its depression effect by inducing inflammatory reaction. ALB and IL-10 were upregulated in 2.0 mg/mL of QFG in both two cells, while IGF1 was only increased by 2.0 mg/mL of QFG in HCT-8 cells (Figures 7(a) and 7(b)).

Moreover, our previous study found that QFG could suppress the increase of tumor weight in the tumor xenograft models of CRC cells [17, 26], so we also analyzed the protein levels of these 7 targets in the serum of the tumor xenograft model treatment with QFG. The results showed that DRD2 and IL-6 were significantly decreased, while the IL-10 was significantly increased (Figure 7(c)). These results suggested that DRD2, IL-6, and IL-10 were regulated by QFG.

HCT-8 and HCT-116 cells were treated following with QFG (0.5, 1, and 2 mg/mL) for 24 h, and the control group was treated with PBS. Cell morphology was observed and photographed at a magnification of ×100.

**Figure 8:**
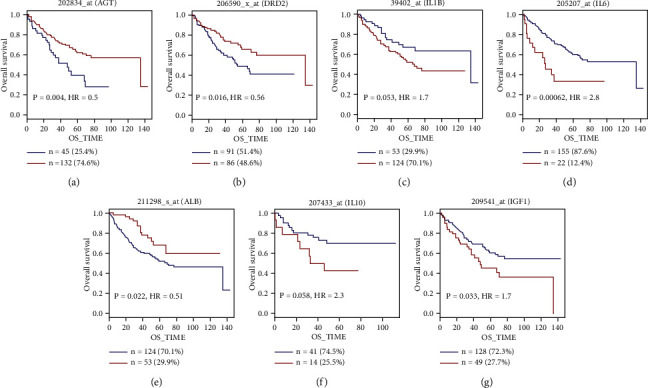
Overall survival curves for the 7 targets of QFG in CRC patients. Data were obtained from GenomicScape. The groups were spited based on the target expression levels (low vs. high), the blue line indicated the lower expression group, and the red line indicated the higher expression group. The cutoff point for groups was calculated by the maximally selected rank statistics methods.

In order to further determine the clinical significance of the targets of QFG in CRC, we analyzed the overall survival curves for the 7 potential targets of QFG in CRC patients in GenomicScape (http://www.genomicscape.com/). Results revealed that patients with high IL-6 and IGF1 expression had a lower overall survival percentage than those with low IL-6 and IGF1 expression, and low AGT, DRD2, and ALB expression had a lower overall survival percentage than those with high AGT, DRD2, and ALB expression (*P*  < 0.05, Figure 8). The data uncovered that in QFG, potential targets of IL-6 and IGF1, and ALB might be more valuable for its clinical application. To sum up, IL-6 could be regulated by QFG in both mRNA levels and protein levels, suggesting that IL-6 could be an index to evaluate the regulation of QFG in CRC.

## 4. Discussion

Traditional Chinese medicine (TCM), especially complex herbal formulations, illustrates the enormous potential and opportunities for new drug innovation. Network pharmacology provides a new chance to obtain systematic insights of TCM and to understand the effects of TCM on disease. Using network pharmacology strategy to predict therapeutic targets of QFG may provide the possibility for the further investigation of the pharmacological molecular mechanisms.

Some genes of mRNA levels of seven genes in two cell lines showed a different reaction in our detection. This may be accused of the different characteristics of the two cells. For example, IL-1*β* (interleukin-1 beta) and IL-6 (interleukin-6) in HCT-116 cells were downregulated in low concentration of QFG and even upregulated in 2 mg/mL of QFG, but IL-1*β* shows a significant difference in HCT-8 cells, or in the treatment of 2 mg/mL of QFG in HCT-116 cells, while the levels of IL-6 were not upregulated. As the proliferation rate for HCT-8 cells was slower than HCT-116, we suggested that the reaction ability for this cell line to the environment change was slower too based on our results. At the same time, although IL-1*β* and IL-6 were both proinflammatory cytokines, we could not exclude the possibility of two cytokines with a subtle activity difference in the anti-inflammatory response. In the GO analysis, the inflammation process was significantly enriched, and three inflammation genes of IL-6, IL-1*β,* and IL-10 were enriched in the seven candidate targets. On the wholesome, our results showed that QFG could downregulate the mRNA levels of the proinflammatory factors of IL6 and IL-1*β*, and upregulate the anti-inflammatory factor of IL-10 in two CRC cells.

Moreover, according to preliminary study, we found there was a dose-dependent effect in cell viability of the treatment of HCT-8 and HCT-116 following with QFG treatment (0, 0.5, 1, and 2 mg/mL) [16], so we designed the experiments to observe whether there is a concentration-dependent effect of the mRNA levels of genes, but the results did not show a dose-dependent effect. Considering for the complexity of regulation of TCM, it might need to be prove by more experiments in the future.

It was found that the upregulation of IL-6 was the reaction for the defence of the host from foreign pathogens. IL-6 would block apoptosis in cells during the inflammatory process to keep cells alive in the toxic environments. However, these processes would also maintain the cells progressing to neoplastic growth by the prevention of cell apoptotic. Our results showed that anti-inflammation effect might be a main way for the QFG. This might provide a basis for QFG in the cancer therapy. Meanwhile, there were pieces of evidences that the inflammation induced by IL-6 and IL-1*β* was through the MAPKs, NF-ΚB, and STAT3 pathways in many cancers [35–39]. In this view, QFG might be playing its inflammation effect on a multipathway on its function of cancer therapy.

Dopamine D2 receptor (D2R2) was involved in the inflammatory process too. The studies were found that DRD2 was upregulated in many cancers and could increase IL-6 production. It was also found to regulate the lipid metabolism. Studies found that D2R antagonists could affect lipid metabolism in cell culture and animal models and play its anti-cancer efficacy [40, 41]. In CRC patients, DRD2 was higher expressed in the CRC tissue patients (Figure 5); however, high expression of DRD2 was associated with the higher overall survival percentage than those with low DRD2 expression (Figure 8), and it is interesting and worthy of the further study.

The albumin (ALB) was also a protein in the lipid metabolism, which carries a wide range of endogenous molecules, including fatty acids to maintain cell osmotic pressure. In CRC patients, ALB was lower expressed in the CRC tissue compared with the normal tissue in patients (Figure 5), and high expression of ALB was associated with the higher overall survival percentage than those with low ALB expression (Figure 8). Several studies showed that the albumin was an independent factor for predicting postoperative survival outcomes since it could be a serum biomarker for the assessment of nutritional status due to shorter half-life (about 1.9 days) than albumin [42, 43]. Moreover, preoperative nutritional status is one of the critical factors for patient outcomes in a variety of surgeries. Therefore, increase of ALB in 2.0 mg/mL of QFG would be benefit to the cancer patients with surgery. In our results, the ALB was decreased in both cells in 1.0 mg/mL of QFG but increased by 2.0 mg/mL of QFG. Compared with other two genes of IGF1 and IL-10, ALB seemed more sensitive in the reaction of QFG. 1.0 mg/mL of QFG could decrease its levels by destroying cell osmotic pressure, but the increase of ALB in 2.0 mg/mL of QFG seems as its detoxification role automatically combined with heavy metal ions [44]. Moreover, ALB was related to the prognosis of different tumors [43, 45]. However, the single level of ALB is often of little significance in clinical application, and the ratio of CRP to ALB was always be used to evaluate the disease status of tumor patients. So, to better study the role of ALB in QFG, the analysis of CRP/ALB might be more valuable in our future study. At the same time, malnutrition is often seen in cancer patients. In clinical application, albumin is often used as a kind of nutrient supplement for cancer patients since it can increase blood volume and maintain plasma osmotic pressure to reduce the weak state of patients. Increase of ALB by QFG might provide a possibility in the improvement of malnutrition and cancer-related fatigue for patients.

Angiotensinogen (AGT) was characterized as a risk factor in many cancers [46–48]. As a main factor to maintained blood pressure and electrolyte homeostasis in body fluid, AGT was predicted to be a potential target for compounds for Stigmasta-5,22-Dien-3-One, Taraxerone, Alpha-Curcumene, 3-Methyl-6,7,8-Trihydropyrrolo[1,2-A] Pyrimidin-2-One, and Tetrahydropalmatine in QFG. In CRC patients, AGT was higher expressed in the CRC tissue in patients (Figure 5), and QFG could be downregulated the levels of AGT in both cell lines (Figures 7(a) and 7(b)). However, in serum of mice treatment following with the QFG treatment, AGT was not changed (Figure 7(c)); moreover, high expression of AGT was associated with the higher overall survival percentage than those with low AGT expression (Figure 8), and it is worthy of the further study.

Insulin-like growth factor (IGF1) was well known in cancer growth, and it was even suggested as a modified gene to cure cancers [49, 50]. There is an evidence that IGFs play strong mitogenic and antiapoptotic actions on various cancer cells [51]. In our study, the levels of IGF1 were upregulated in HCT-8 cells in the 2 mg/mL of QFG treatment, while this concentration of QFG would lead the cells extremely apoptosis. Considering the activation of IGF1 needs to bind to its receptor of IGF1-R, we may need to study the function of IGF1-R in the future study of QFG-induced cell apoptosis. In addition, IGF1/IGF1-R was found to a target associated with the PI3K/AKT pathway [52] in the depression of cancer proliferation, subcellular distribution, invasion, and chemoresistance [53–55]. So, it is believed that the application of QFG in the therapy of cancers would provide benefits in these aspects by the regulation of IGF1.

Pain caused by tumor and treatment is an unavoidable problem, which affects the quality of life for cancer patients for a long time. In our study, we found that compounds of nicotine and nicotinic acid and the neural related pathways were enriched in QFG (Figure 3). In addition, DRD2 was the common target among “Jun,” “Chen,” “Zuo” and “Shi” medicines (Figure 1), and the activation of DRD2 was found to potentially limit, such as anxiety, dizziness, serious digestive problems, agitation, and so on [56]. These clues suggested that QFG would give the benefits for the pain relief and quality of life for cancer patients. Since the inflammatory factors are also related to pain and cachexia, QFG may improve malnutrition, cancer fatigue, and analgesia by regulating multipathways in the treatment of CRC.

In our study, we aimed to analyze the compatibility law of TCM prescriptions for QFG by the network pharmacology method. Targets in QFG were split into groups according to the composition theory of a prescription based on the application of this Chinese medicine prescription. Then, we analyzed the distribution and the term of GO analysis, KEGG pathway analysis, and OMIM/TTD disease enrichment (TTD) of targets in four functions of medicines for the better understanding of the scientific principle of a prescription. According to the composition theory of a prescription in Traditional Chinese medicine, the “Jun” medicine plays a major role in the treatment of the main disease of the prescription, and “Chen” medicine strengthens the treatment of the main disease, while it always has the same effect of the “Jun” medicine. “Zuo” medicine eliminates or slows down the toxicity of “Jun” and “Chen” medicines. “Shi” medicine is always a concordant medicine for the prescription. We assumed that the pathway of “Jun” and “Chen” would be similar, but the results did not exactly consistent with our assumption. In a Traditional Chinese medicine prescription, a medicine played a role as “Jun” or “Chen” not only depends on its function, but also depends on the dose of each medicine in the prescription. The results showed that each function of medicine regulated the multiple targets and pathways, and these targets and pathways were both repetitive and complementary, which were consistent with the law of mutual coordination in the compatibility law of TCM prescriptions. Moreover, it was interesting that “Zuo” medicine (404 of targets) and “Shi” medicine (323 of targets) contained more targets that were not predicted by “Jun” and “Chen” medicines, and in TTD analysis, two of medicines could regulate “Pain,” which were not the main disease regulated by “Jun” and “Chen” medicines. These results might provide pieces of evidences for auxiliary effect of “Zuo” and “Shi” medicines in the compatibility law of TCM prescriptions. In addition, there is a mention that in a prescription, the dose of “Jun” medicine is the most, “Chen” medicine is less than “Jun” medicine, and “Zuo” and “Shi” medicines are less than “Chen” in the book of *medicine origin* [57], which suggested that the dose of a medicine in a prescription was as important as the role of a medicine. Therefore, the methods of network pharmacology analysis might be useful for searching the targets or pathways corresponding to its compounds, but could not include the differences bring by the dose. Since the effects of a target or a pathway activation by a different dose of a medicine cannot be simply superimposed based on its dose, in further study, it needs to be verified through more experiments in *vivo* and in *vitro*.

## 5. Conclusions

In our study, we found that there are 21 molecules enriched in QFG regulation, 613 targets of QFG were significantly different in the expression of CRC. QFG might have anti-inflammatory effect and the regulation of lipid metabolic process. 7 candidate targets were selected. At last, we found that QFG would downregulate the expression of AGT, DRD2, and IL-6 and upregulate the expression of ALB and IL-10 in both HCT-116 and HCT-8 cells. However, our validation of candidate targets was limited in cells. From a critical point of view, further experiments are needed to explore in *vivo* and clinically. Meanwhile, since there is a possibility of drug addiction in the analysis of KEGG pathway, the pharmacy of QFG in the clinical application should be strict and cautious in future study.

## Figures and Tables

**Table 1:** Herbs in the Qingjie Fuzheng granules.

Chinese name	English name	Latin name
Ban Zhi Lian	Barbed skullcap	*Scutellaria Barbata* [syn. Scutellaria rivularis]
Dang Shen	Codonopsis pilosula var modesta, codonopsis tangshen, codonopsis tubulosa, codonopsis subglobosa, codonopsis canescens, codonopsis clematidea	*Codonopsis Pilosula*
Gan Cao	Glycyrrhiza inflata, glycyrrhiza glabra, glycyrrhiza kansuensis, glycyrrhiza aspera, glycyrrhiza yunnanensis, glycyrrhiza squamulosa	*Glycyrrhiza Uralensis*
Huang Qi	*Astragalus mongholicus*, astragalus chrysopterus, astragalus ernestii	*Astragalus Membranaceus*
Bai hua She She Cao	Hedyotis corymbosa	*Oldenlandia Diffusa* [Syn. Hedyotis diffusa]
Chao Mai Ya	Prepared germinated barley	*Fructus Hordei* germinatus preparata

**Table 2:** The potential targets ranked by CytoNCA in the PPI network.

Rank	Betweenness		Closeness		Degree		Eigenvector		Common genes
	Genes	Score	Genes	Score	Genes	Score	Genes	Score	
**1**	ALB	40887.94	ALB	0.546119	ALB	160	ALB	0.197964	AGT
**2**	IL-6	17095.32	IL-6	0.513305	IL-6	136	IL-6	0.189082	ALB
**3**	ESR1	10869.45	IL-1B	0.486179	AGT	89	AGT	0.162717	IL-10
**4**	IL-1B	9260.864	IGF1	0.483037	IGF1	88	TAC1	0.141351	IL-1B
**5**	DRD2	7359.653	ESR1	0.479936	IL-1B	86	SST	0.140133	DRD2
**6**	COL1A1	7237.071	NGF	0.473851	DRD2	83	CCL5	0.136616	IGF1
**7**	NGF	7171.707	AGT	0.470866	TAC1	83	DRD2	0.133437	LEP
**8**	IGF1	7001.154	TAC1	0.468285	ESR1	78	IL-1B	0.133364	IL-6
**9**	AGT	6562.12	LEP	0.467919	IL-10	78	IGF1	0.129535	TAC1
**10**	AR	6017.094	DRD2	0.466823	MMP9	72	CNR1	0.127909	
**11**	TAC1	5999.071	TH	0.466095	SST	71	IL-10	0.124008	
**12**	CALB1	5883.848	REN	0.459293	NGF	71	CCR7	0.123485	
**13**	SHH	5830.178	CAV1	0.458589	LEP	67	BDKRB2	0.121594	
**14**	CAV1	5809.558	AR	0.458589	CCL5	66	NMUR2	0.120083	
**15**	LEP	5511.375	SST	0.458238	TH	65	MMP9	0.118587	
**16**	IL10	5465.809	IL10	0.458238	F2	63	CX3CR1	0.118028	
**17**	GPT	5407.28	CNR1	0.456837	CNR1	63	C3	0.113235	
**18**	REN	5348.518	ACHE	0.455446	BDKRB2	60	AGTR1	0.11234	
**19**	NOS1	5196.147	GRIN2B	0.454753	CCR7	59	PF4	0.112172	
**20**	CYP3A4	4893.269	MMP9	0.454062	AGTR1	59	LEP	0.112127	

## Data Availability

All data can be found in the manuscript.

## Supplementary Materials

Supplementary Materials S1 Table: predicted targets of QFG.
