# Supplementary material for: Based on the Network Pharmacology to Investigate the Mechanism of Qingjie Fuzheng Granules against Colorectal Cancer
Source: Evid Based Complement Alternat Med. 2022 Mar 4;2022:7242640. doi: 10.1155/2022/7242640 (PMC8916896; doi:10.1155/2022/7242640)
Supplement: Supplementary Materials — S1 Table: predicted targets of QFG. [file 7242640.f1.docx]

| **Compound** | | **Predicted targets [Gene Symbol] ranked according to the decreasing (**[**score**](http://bionet.ncpsb.org/batman-tcm/index.php/Home/document/index#Algorithm)**)** | |
| --- | --- | --- | --- |
| [18beta-Glycyrrhetinic Acid](http://www.megabionet.org/tcmid/ingredient/23092/) | [HSD11B1](http://www.ncbi.nlm.nih.gov/gene/?term=3290)(known target in KEGG)[AR](http://www.ncbi.nlm.nih.gov/gene/?term=367)(122.778)[NR3C1](http://www.ncbi.nlm.nih.gov/gene/?term=2908)(122.778)[ANXA1](http://www.ncbi.nlm.nih.gov/gene/?term=301)(122.778)[PTGER1](http://www.ncbi.nlm.nih.gov/gene/?term=5731)(80.882) [PTGER4](http://www.ncbi.nlm.nih.gov/gene/?term=5734)(80.882)[PTGER2](http://www.ncbi.nlm.nih.gov/gene/?term=5732)(80.882)[PTGER3](http://www.ncbi.nlm.nih.gov/gene/?term=5733)(80.882)[CD300A](http://www.ncbi.nlm.nih.gov/gene/?term=11314)(55.444)[KIF14](http://www.ncbi.nlm.nih.gov/gene/?term=9928)(55.444) [SRD5A1](http://www.ncbi.nlm.nih.gov/gene/?term=6715)(48.000)[CYP17A1](http://www.ncbi.nlm.nih.gov/gene/?term=1586)(48.000)[SLC8A1](http://www.ncbi.nlm.nih.gov/gene/?term=6546)(48.000)[F12](http://www.ncbi.nlm.nih.gov/gene/?term=2161)(48.000)[PRLR](http://www.ncbi.nlm.nih.gov/gene/?term=5618)(48.000) [ESR1](http://www.ncbi.nlm.nih.gov/gene/?term=2099)(48.000)[TRPV1](http://www.ncbi.nlm.nih.gov/gene/?term=7442)(48.000)[PPARG](http://www.ncbi.nlm.nih.gov/gene/?term=5468)(48.000)[FFAR1](http://www.ncbi.nlm.nih.gov/gene/?term=2864)(48.000)[PGR](http://www.ncbi.nlm.nih.gov/gene/?term=5241)(48.000) [OPRK1](http://www.ncbi.nlm.nih.gov/gene/?term=4986)(48.000)[FADS2](http://www.ncbi.nlm.nih.gov/gene/?term=9415)(48.000)[FADS1](http://www.ncbi.nlm.nih.gov/gene/?term=3992)(48.000)[PPARD](http://www.ncbi.nlm.nih.gov/gene/?term=5467)(48.000)[PTGS1](http://www.ncbi.nlm.nih.gov/gene/?term=5742)(48.000) [ACSL4](http://www.ncbi.nlm.nih.gov/gene/?term=2182)(48.000)[PTGS2](http://www.ncbi.nlm.nih.gov/gene/?term=5743)(48.000)[ELOVL4](http://www.ncbi.nlm.nih.gov/gene/?term=6785)(48.000)[ACSL3](http://www.ncbi.nlm.nih.gov/gene/?term=2181)(48.000)[NR3C2](http://www.ncbi.nlm.nih.gov/gene/?term=4306)(48.000) [CACNB1](http://www.ncbi.nlm.nih.gov/gene/?term=782)(25.857)[CACNA2D1](http://www.ncbi.nlm.nih.gov/gene/?term=781)(25.857)[CACNA1H](http://www.ncbi.nlm.nih.gov/gene/?term=8912)(25.857)[CACNA1F](http://www.ncbi.nlm.nih.gov/gene/?term=778)(25.857)[CACNA1A](http://www.ncbi.nlm.nih.gov/gene/?term=773)(25.857) [CACNA1I](http://www.ncbi.nlm.nih.gov/gene/?term=8911)(25.857)[CACNA2D2](http://www.ncbi.nlm.nih.gov/gene/?term=9254)(25.857)[SHBG](http://www.ncbi.nlm.nih.gov/gene/?term=6462)(25.857)[CACNA1S](http://www.ncbi.nlm.nih.gov/gene/?term=779)(25.857)[CACNA2D3](http://www.ncbi.nlm.nih.gov/gene/?term=55799)(25.857) [CACNB4](http://www.ncbi.nlm.nih.gov/gene/?term=785)(25.857)[CACNA1B](http://www.ncbi.nlm.nih.gov/gene/?term=774)(25.857)[CACNA1C](http://www.ncbi.nlm.nih.gov/gene/?term=775)(25.857)[CYP11B1](http://www.ncbi.nlm.nih.gov/gene/?term=1584)(25.857)[CACNA1D](http://www.ncbi.nlm.nih.gov/gene/?term=776)(25.857) [CACNB3](http://www.ncbi.nlm.nih.gov/gene/?term=784)(25.857)[CYP11B2](http://www.ncbi.nlm.nih.gov/gene/?term=1585)(25.857)[CACNA1G](http://www.ncbi.nlm.nih.gov/gene/?term=8913)(25.857)[CACNB2](http://www.ncbi.nlm.nih.gov/gene/?term=783)(25.857)[CACNG1](http://www.ncbi.nlm.nih.gov/gene/?term=786)(25.857) [DAPK2](http://www.ncbi.nlm.nih.gov/gene/?term=23604)(22.373)[HPGD](http://www.ncbi.nlm.nih.gov/gene/?term=3248)(22.373)[ADAM8](http://www.ncbi.nlm.nih.gov/gene/?term=101)(22.373)[CCL24](http://www.ncbi.nlm.nih.gov/gene/?term=6369)(22.373)[IL4](http://www.ncbi.nlm.nih.gov/gene/?term=3565)(22.373) | |
| [Ruvoside](http://www.megabionet.org/tcmid/ingredient/19075/) | [ATP1A1](http://www.ncbi.nlm.nih.gov/gene/?term=476)(122.778)[GLRA3](http://www.ncbi.nlm.nih.gov/gene/?term=8001)(80.882)[GABRB3](http://www.ncbi.nlm.nih.gov/gene/?term=2562)(80.882)[NRXN1](http://www.ncbi.nlm.nih.gov/gene/?term=9378)(22.373)[GLRB](http://www.ncbi.nlm.nih.gov/gene/?term=2743)(22.373) | |
| [2-Hydroxy-3-Methoxystrychnine](http://www.megabionet.org/tcmid/ingredient/10456/) | This compound doesn't have any potential target with score larger than 20. | |
| [Nicotiflorin](http://www.megabionet.org/tcmid/ingredient/15516/) | This compound doesn't have any potential target with score larger than 20. | |
| [Uridine](http://www.megabionet.org/tcmid/ingredient/22236/) | [TYMS](http://www.ncbi.nlm.nih.gov/gene/?term=7298)(122.778)[NT5C2](http://www.ncbi.nlm.nih.gov/gene/?term=22978)(80.882)[ADK](http://www.ncbi.nlm.nih.gov/gene/?term=132)(80.882)[IMPDH1](http://www.ncbi.nlm.nih.gov/gene/?term=3614)(80.882)[ENPP1](http://www.ncbi.nlm.nih.gov/gene/?term=5167)(80.882) [POLA1](http://www.ncbi.nlm.nih.gov/gene/?term=5422)(48.000)[PNP](http://www.ncbi.nlm.nih.gov/gene/?term=4860)(48.000)[POLB](http://www.ncbi.nlm.nih.gov/gene/?term=5423)(48.000)[TERT](http://www.ncbi.nlm.nih.gov/gene/?term=7015)(48.000)[GSR](http://www.ncbi.nlm.nih.gov/gene/?term=2936)(23.000) [GCDH](http://www.ncbi.nlm.nih.gov/gene/?term=2639)(23.000)[ERO1B](http://www.ncbi.nlm.nih.gov/gene/?term=56605)(23.000)[IVD](http://www.ncbi.nlm.nih.gov/gene/?term=3712)(23.000)[NQO2](http://www.ncbi.nlm.nih.gov/gene/?term=4835)(23.000)[DPYD](http://www.ncbi.nlm.nih.gov/gene/?term=1806)(23.000) [POR](http://www.ncbi.nlm.nih.gov/gene/?term=5447)(23.000)[CYB5R1](http://www.ncbi.nlm.nih.gov/gene/?term=51706)(23.000)[MAOB](http://www.ncbi.nlm.nih.gov/gene/?term=4129)(23.000)[FDXR](http://www.ncbi.nlm.nih.gov/gene/?term=2232)(23.000)[DAO](http://www.ncbi.nlm.nih.gov/gene/?term=1610)(23.000) [TXNRD1](http://www.ncbi.nlm.nih.gov/gene/?term=7296)(23.000)[ACADS](http://www.ncbi.nlm.nih.gov/gene/?term=35)(23.000)[CYB5R3](http://www.ncbi.nlm.nih.gov/gene/?term=1727)(23.000)[MAOA](http://www.ncbi.nlm.nih.gov/gene/?term=4128)(23.000)[IL4I1](http://www.ncbi.nlm.nih.gov/gene/?term=259307)(23.000) [AIFM1](http://www.ncbi.nlm.nih.gov/gene/?term=9131)(23.000)[ACOX1](http://www.ncbi.nlm.nih.gov/gene/?term=51)(23.000)[ACADM](http://www.ncbi.nlm.nih.gov/gene/?term=34)(23.000)[NQO1](http://www.ncbi.nlm.nih.gov/gene/?term=1728)(23.000)[XDH](http://www.ncbi.nlm.nih.gov/gene/?term=7498)(23.000) [DNMT1](http://www.ncbi.nlm.nih.gov/gene/?term=1786)(23.000)[ACAD8](http://www.ncbi.nlm.nih.gov/gene/?term=27034)(23.000)[NOS1](http://www.ncbi.nlm.nih.gov/gene/?term=4842)(23.000)[DLD](http://www.ncbi.nlm.nih.gov/gene/?term=1738)(23.000)[GFER](http://www.ncbi.nlm.nih.gov/gene/?term=2671)(23.000) [AK5](http://www.ncbi.nlm.nih.gov/gene/?term=26289)(22.373)[GUK1](http://www.ncbi.nlm.nih.gov/gene/?term=2987)(22.373)[ANKH](http://www.ncbi.nlm.nih.gov/gene/?term=56172)(22.373)[ENPP3](http://www.ncbi.nlm.nih.gov/gene/?term=5169)(22.373)[APRT](http://www.ncbi.nlm.nih.gov/gene/?term=353)(22.373) [AK9](http://www.ncbi.nlm.nih.gov/gene/?term=221264)(22.373)[NUDT12](http://www.ncbi.nlm.nih.gov/gene/?term=83594)(22.373)[IMPDH2](http://www.ncbi.nlm.nih.gov/gene/?term=3615)(22.373) | |
| [1-Peroxyferolide](http://www.megabionet.org/tcmid/ingredient/16960/) | This compound doesn't have any potential target with score larger than 20. | |
| [1,22-Docosanediol](http://www.megabionet.org/tcmid/ingredient/6535/) | [TRPV3](http://www.ncbi.nlm.nih.gov/gene/?term=162514)(122.778)[OPRK1](http://www.ncbi.nlm.nih.gov/gene/?term=4986)(122.778)[TRPM8](http://www.ncbi.nlm.nih.gov/gene/?term=79054)(122.778)[TRPA1](http://www.ncbi.nlm.nih.gov/gene/?term=8989)(122.778)[CACNB1](http://www.ncbi.nlm.nih.gov/gene/?term=782)(80.882) [GRIA4](http://www.ncbi.nlm.nih.gov/gene/?term=2893)(80.882)[GABRA2](http://www.ncbi.nlm.nih.gov/gene/?term=2555)(80.882)[CACNG2](http://www.ncbi.nlm.nih.gov/gene/?term=10369)(80.882)[GABRD](http://www.ncbi.nlm.nih.gov/gene/?term=2563)(80.882)[CHRNA4](http://www.ncbi.nlm.nih.gov/gene/?term=1137)(80.882) [CHRNB3](http://www.ncbi.nlm.nih.gov/gene/?term=1142)(80.882)[GABRB1](http://www.ncbi.nlm.nih.gov/gene/?term=2560)(80.882)[GRIA1](http://www.ncbi.nlm.nih.gov/gene/?term=2890)(80.882)[GLRA2](http://www.ncbi.nlm.nih.gov/gene/?term=2742)(80.882)[KCNJ3](http://www.ncbi.nlm.nih.gov/gene/?term=3760)(80.882) [GABRG3](http://www.ncbi.nlm.nih.gov/gene/?term=2567)(80.882)[VCAM1](http://www.ncbi.nlm.nih.gov/gene/?term=7412)(80.882)[CHRNA7](http://www.ncbi.nlm.nih.gov/gene/?term=1139)(80.882)[KCNJ6](http://www.ncbi.nlm.nih.gov/gene/?term=3763)(80.882)[GABRE](http://www.ncbi.nlm.nih.gov/gene/?term=2564)(80.882) [GABRA3](http://www.ncbi.nlm.nih.gov/gene/?term=2556)(80.882)[SLC29A1](http://www.ncbi.nlm.nih.gov/gene/?term=2030)(80.882)[CACNA1S](http://www.ncbi.nlm.nih.gov/gene/?term=779)(80.882)[GABRG1](http://www.ncbi.nlm.nih.gov/gene/?term=2565)(80.882)[GABRP](http://www.ncbi.nlm.nih.gov/gene/?term=2568)(80.882) [CHRNA10](http://www.ncbi.nlm.nih.gov/gene/?term=57053)(80.882)[GRIA2](http://www.ncbi.nlm.nih.gov/gene/?term=2891)(80.882)[GABRA4](http://www.ncbi.nlm.nih.gov/gene/?term=2557)(80.882)[CHRNA3](http://www.ncbi.nlm.nih.gov/gene/?term=1136)(80.882)[GABRB2](http://www.ncbi.nlm.nih.gov/gene/?term=2561)(80.882) [CACNA1C](http://www.ncbi.nlm.nih.gov/gene/?term=775)(80.882)[CHRNB2](http://www.ncbi.nlm.nih.gov/gene/?term=1141)(80.882)[CACNA1D](http://www.ncbi.nlm.nih.gov/gene/?term=776)(80.882)[HTR3D](http://www.ncbi.nlm.nih.gov/gene/?term=200909)(80.882)[GABRQ](http://www.ncbi.nlm.nih.gov/gene/?term=55879)(80.882) [CHRNA2](http://www.ncbi.nlm.nih.gov/gene/?term=1135)(80.882)[CHRNA6](http://www.ncbi.nlm.nih.gov/gene/?term=8973)(80.882)[GABRB3](http://www.ncbi.nlm.nih.gov/gene/?term=2562)(80.882)[KCNJ5](http://www.ncbi.nlm.nih.gov/gene/?term=3762)(80.882)[GRIN3A](http://www.ncbi.nlm.nih.gov/gene/?term=116443)(80.882) [HTR3B](http://www.ncbi.nlm.nih.gov/gene/?term=9177)(80.882)[GRIA3](http://www.ncbi.nlm.nih.gov/gene/?term=2892)(80.882)[GABRA5](http://www.ncbi.nlm.nih.gov/gene/?term=2558)(80.882)[SLC29A2](http://www.ncbi.nlm.nih.gov/gene/?term=3177)(80.882)[HTR3E](http://www.ncbi.nlm.nih.gov/gene/?term=285242)(80.882) [GABRA6](http://www.ncbi.nlm.nih.gov/gene/?term=2559)(80.882)[HTR3A](http://www.ncbi.nlm.nih.gov/gene/?term=3359)(80.882)[GABRA1](http://www.ncbi.nlm.nih.gov/gene/?term=2554)(80.882)[CHRNA9](http://www.ncbi.nlm.nih.gov/gene/?term=55584)(80.882)[CHRFAM7A](http://www.ncbi.nlm.nih.gov/gene/?term=89832)(80.882) [CHRNA5](http://www.ncbi.nlm.nih.gov/gene/?term=1138)(80.882)[CHRNB4](http://www.ncbi.nlm.nih.gov/gene/?term=1143)(80.882)[KCNJ9](http://www.ncbi.nlm.nih.gov/gene/?term=3765)(80.882)[HTR3C](http://www.ncbi.nlm.nih.gov/gene/?term=170572)(80.882)[GLRA1](http://www.ncbi.nlm.nih.gov/gene/?term=2741)(80.882) [CACNG1](http://www.ncbi.nlm.nih.gov/gene/?term=786)(80.882)[ADAM8](http://www.ncbi.nlm.nih.gov/gene/?term=101)(55.444)[SLC28A3](http://www.ncbi.nlm.nih.gov/gene/?term=64078)(55.444)[ADRA1B](http://www.ncbi.nlm.nih.gov/gene/?term=147)(55.444)[FOXL2](http://www.ncbi.nlm.nih.gov/gene/?term=668)(55.444) [DMTN](http://www.ncbi.nlm.nih.gov/gene/?term=2039)(55.444)[KCNMA1](http://www.ncbi.nlm.nih.gov/gene/?term=3778)(55.444)[SCN11A](http://www.ncbi.nlm.nih.gov/gene/?term=11280)(48.000)[SCN2B](http://www.ncbi.nlm.nih.gov/gene/?term=6327)(48.000)[ABAT](http://www.ncbi.nlm.nih.gov/gene/?term=18)(48.000) [SCN1A](http://www.ncbi.nlm.nih.gov/gene/?term=6323)(48.000)[SCN3B](http://www.ncbi.nlm.nih.gov/gene/?term=55800)(48.000)[SCN3A](http://www.ncbi.nlm.nih.gov/gene/?term=6328)(48.000)[SCN7A](http://www.ncbi.nlm.nih.gov/gene/?term=6332)(48.000)[SCN5A](http://www.ncbi.nlm.nih.gov/gene/?term=6331)(48.000) [SCN10A](http://www.ncbi.nlm.nih.gov/gene/?term=6336)(48.000)[AKR1D1](http://www.ncbi.nlm.nih.gov/gene/?term=6718)(48.000)[ALDH5A1](http://www.ncbi.nlm.nih.gov/gene/?term=7915)(48.000)[SCN2A](http://www.ncbi.nlm.nih.gov/gene/?term=6326)(48.000)[HDAC9](http://www.ncbi.nlm.nih.gov/gene/?term=9734)(48.000) [TYR](http://www.ncbi.nlm.nih.gov/gene/?term=7299)(48.000)[SCN9A](http://www.ncbi.nlm.nih.gov/gene/?term=6335)(48.000)[SCN4A](http://www.ncbi.nlm.nih.gov/gene/?term=6329)(48.000)[SCN4B](http://www.ncbi.nlm.nih.gov/gene/?term=6330)(48.000)[SRD5A2](http://www.ncbi.nlm.nih.gov/gene/?term=6716)(48.000) [ACADSB](http://www.ncbi.nlm.nih.gov/gene/?term=36)(48.000)[SCN8A](http://www.ncbi.nlm.nih.gov/gene/?term=6334)(48.000)[SCN1B](http://www.ncbi.nlm.nih.gov/gene/?term=6324)(48.000)[OGDH](http://www.ncbi.nlm.nih.gov/gene/?term=4967)(48.000)[HDAC2](http://www.ncbi.nlm.nih.gov/gene/?term=3066)(48.000) [GLRA3](http://www.ncbi.nlm.nih.gov/gene/?term=8001)(23.000)[HMGA2](http://www.ncbi.nlm.nih.gov/gene/?term=8091)(22.373)[CACNA1H](http://www.ncbi.nlm.nih.gov/gene/?term=8912)(22.373)[SLITRK6](http://www.ncbi.nlm.nih.gov/gene/?term=84189)(22.373)[UTS2](http://www.ncbi.nlm.nih.gov/gene/?term=10911)(22.373) [NAV2](http://www.ncbi.nlm.nih.gov/gene/?term=89797)(22.373)[P2RX2](http://www.ncbi.nlm.nih.gov/gene/?term=22953)(22.373)[HTR2A](http://www.ncbi.nlm.nih.gov/gene/?term=3356)(22.373)[AVPR1A](http://www.ncbi.nlm.nih.gov/gene/?term=552)(22.373)[ADORA2A](http://www.ncbi.nlm.nih.gov/gene/?term=135)(22.373) [CACNA1A](http://www.ncbi.nlm.nih.gov/gene/?term=773)(22.373)[ANK2](http://www.ncbi.nlm.nih.gov/gene/?term=287)(22.373)[ZP4](http://www.ncbi.nlm.nih.gov/gene/?term=57829)(22.373)[SUMO1](http://www.ncbi.nlm.nih.gov/gene/?term=7341)(22.373)[CNR2](http://www.ncbi.nlm.nih.gov/gene/?term=1269)(22.373) [MTNR1B](http://www.ncbi.nlm.nih.gov/gene/?term=4544)(22.373)[MAOB](http://www.ncbi.nlm.nih.gov/gene/?term=4129)(22.373)[GLRB](http://www.ncbi.nlm.nih.gov/gene/?term=2743)(22.373)[P2RX3](http://www.ncbi.nlm.nih.gov/gene/?term=5024)(22.373)[SLC44A4](http://www.ncbi.nlm.nih.gov/gene/?term=80736)(22.373) [FLOT1](http://www.ncbi.nlm.nih.gov/gene/?term=10211)(22.373)[NPY5R](http://www.ncbi.nlm.nih.gov/gene/?term=4889)(22.373)[MSN](http://www.ncbi.nlm.nih.gov/gene/?term=4478)(22.373)[GHRL](http://www.ncbi.nlm.nih.gov/gene/?term=51738)(22.373)[TBR1](http://www.ncbi.nlm.nih.gov/gene/?term=10716)(22.373) [ADRA1D](http://www.ncbi.nlm.nih.gov/gene/?term=146)(22.373)[DRD2](http://www.ncbi.nlm.nih.gov/gene/?term=1813)(22.373)[AVP](http://www.ncbi.nlm.nih.gov/gene/?term=551)(22.373)[PAX2](http://www.ncbi.nlm.nih.gov/gene/?term=5076)(22.373)[AOC1](http://www.ncbi.nlm.nih.gov/gene/?term=26)(22.373) [MAOA](http://www.ncbi.nlm.nih.gov/gene/?term=4128)(22.373)[UTS2R](http://www.ncbi.nlm.nih.gov/gene/?term=2837)(22.373)[ADIRF](http://www.ncbi.nlm.nih.gov/gene/?term=10974)(22.373)[GHRH](http://www.ncbi.nlm.nih.gov/gene/?term=2691)(22.373)[HCRT](http://www.ncbi.nlm.nih.gov/gene/?term=3060)(22.373) [RAB3B](http://www.ncbi.nlm.nih.gov/gene/?term=5865)(22.373)[KCNE5](http://www.ncbi.nlm.nih.gov/gene/?term=23630)(22.373)[CNR1](http://www.ncbi.nlm.nih.gov/gene/?term=1268)(22.373)[SLC18A3](http://www.ncbi.nlm.nih.gov/gene/?term=6572)(22.373)[EZR](http://www.ncbi.nlm.nih.gov/gene/?term=7430)(22.373) [DRD4](http://www.ncbi.nlm.nih.gov/gene/?term=1815)(22.373)[CRH](http://www.ncbi.nlm.nih.gov/gene/?term=1392)(22.373)[GLI3](http://www.ncbi.nlm.nih.gov/gene/?term=2737)(22.373)[CAV3](http://www.ncbi.nlm.nih.gov/gene/?term=859)(22.373)[SLC9A6](http://www.ncbi.nlm.nih.gov/gene/?term=10479)(22.373) [AOC3](http://www.ncbi.nlm.nih.gov/gene/?term=8639)(22.373)[NCOA1](http://www.ncbi.nlm.nih.gov/gene/?term=8648)(22.373)[AOC2](http://www.ncbi.nlm.nih.gov/gene/?term=314)(22.373)[IL6](http://www.ncbi.nlm.nih.gov/gene/?term=3569)(22.373)[GLRA4](http://www.ncbi.nlm.nih.gov/gene/?term=441509)(22.373) [RNF207](http://www.ncbi.nlm.nih.gov/gene/?term=388591)(22.373)[ROCK1](http://www.ncbi.nlm.nih.gov/gene/?term=6093)(22.373)[KCNA5](http://www.ncbi.nlm.nih.gov/gene/?term=3741)(22.373)[TACR2](http://www.ncbi.nlm.nih.gov/gene/?term=6865)(22.373)[NRP1](http://www.ncbi.nlm.nih.gov/gene/?term=8829)(22.373) [NPPA](http://www.ncbi.nlm.nih.gov/gene/?term=4878)(22.373)[KMT2A](http://www.ncbi.nlm.nih.gov/gene/?term=4297)(22.373)[TNR](http://www.ncbi.nlm.nih.gov/gene/?term=7143)(22.373)[ZP3](http://www.ncbi.nlm.nih.gov/gene/?term=7784)(22.373)[PLCB1](http://www.ncbi.nlm.nih.gov/gene/?term=23236)(22.373) [EPHB1](http://www.ncbi.nlm.nih.gov/gene/?term=2047)(22.373)[DRD1](http://www.ncbi.nlm.nih.gov/gene/?term=1812)(22.373)[EEA1](http://www.ncbi.nlm.nih.gov/gene/?term=8411)(22.373)[POU4F3](http://www.ncbi.nlm.nih.gov/gene/?term=5459)(22.373)[CALCA](http://www.ncbi.nlm.nih.gov/gene/?term=796)(22.373) [PINK1](http://www.ncbi.nlm.nih.gov/gene/?term=65018)(22.373)[KCNQ1](http://www.ncbi.nlm.nih.gov/gene/?term=3784)(22.373)[ICAM1](http://www.ncbi.nlm.nih.gov/gene/?term=3383)(22.373)[CARTPT](http://www.ncbi.nlm.nih.gov/gene/?term=9607)(22.373)[ATP8B1](http://www.ncbi.nlm.nih.gov/gene/?term=5205)(22.373) [KISS1](http://www.ncbi.nlm.nih.gov/gene/?term=3814)(22.373)[GRM7](http://www.ncbi.nlm.nih.gov/gene/?term=2917)(22.373)[FOS](http://www.ncbi.nlm.nih.gov/gene/?term=2353)(22.373)[GRIN1](http://www.ncbi.nlm.nih.gov/gene/?term=2902)(22.373) | |
| [Palmitic Acid](http://www.megabionet.org/tcmid/ingredient/23175/) | This compound doesn't have any potential target with score larger than 20. | |
| [Azelaic Acid](http://www.megabionet.org/tcmid/ingredient/24407/) | [AKR1D1](http://www.ncbi.nlm.nih.gov/gene/?term=6718)(686.000)[TYR](http://www.ncbi.nlm.nih.gov/gene/?term=7299)(686.000)[SRD5A2](http://www.ncbi.nlm.nih.gov/gene/?term=6716)(686.000)[SCN11A](http://www.ncbi.nlm.nih.gov/gene/?term=11280)(122.778)[SCN2B](http://www.ncbi.nlm.nih.gov/gene/?term=6327)(122.778) [ABAT](http://www.ncbi.nlm.nih.gov/gene/?term=18)(122.778)[SCN1A](http://www.ncbi.nlm.nih.gov/gene/?term=6323)(122.778)[ESRRG](http://www.ncbi.nlm.nih.gov/gene/?term=2104)(122.778)[SCN3B](http://www.ncbi.nlm.nih.gov/gene/?term=55800)(122.778)[COX6C](http://www.ncbi.nlm.nih.gov/gene/?term=1345)(122.778) [COX5B](http://www.ncbi.nlm.nih.gov/gene/?term=1329)(122.778)[COX7C](http://www.ncbi.nlm.nih.gov/gene/?term=1350)(122.778)[COX1](http://www.ncbi.nlm.nih.gov/gene/?term=4512)(122.778)[SCN3A](http://www.ncbi.nlm.nih.gov/gene/?term=6328)(122.778)[AKR1C2](http://www.ncbi.nlm.nih.gov/gene/?term=1646)(122.778) [SCN7A](http://www.ncbi.nlm.nih.gov/gene/?term=6332)(122.778)[SCN5A](http://www.ncbi.nlm.nih.gov/gene/?term=6331)(122.778)[SCN10A](http://www.ncbi.nlm.nih.gov/gene/?term=6336)(122.778)[ALDH5A1](http://www.ncbi.nlm.nih.gov/gene/?term=7915)(122.778)[SCN2A](http://www.ncbi.nlm.nih.gov/gene/?term=6326)(122.778) [COX5A](http://www.ncbi.nlm.nih.gov/gene/?term=9377)(122.778)[COX3](http://www.ncbi.nlm.nih.gov/gene/?term=4514)(122.778)[AR](http://www.ncbi.nlm.nih.gov/gene/?term=367)(122.778)[COX7A1](http://www.ncbi.nlm.nih.gov/gene/?term=1346)(122.778)[FECH](http://www.ncbi.nlm.nih.gov/gene/?term=2235)(122.778) [COX4I1](http://www.ncbi.nlm.nih.gov/gene/?term=1327)(122.778)[HDAC9](http://www.ncbi.nlm.nih.gov/gene/?term=9734)(122.778)[PLA2G1B](http://www.ncbi.nlm.nih.gov/gene/?term=5319)(122.778)[COX6A2](http://www.ncbi.nlm.nih.gov/gene/?term=1339)(122.778)[SCN9A](http://www.ncbi.nlm.nih.gov/gene/?term=6335)(122.778) [SCN4A](http://www.ncbi.nlm.nih.gov/gene/?term=6329)(122.778)[SCN4B](http://www.ncbi.nlm.nih.gov/gene/?term=6330)(122.778)[ADH1C](http://www.ncbi.nlm.nih.gov/gene/?term=126)(122.778)[COX6B1](http://www.ncbi.nlm.nih.gov/gene/?term=1340)(122.778)[FABP6](http://www.ncbi.nlm.nih.gov/gene/?term=2172)(122.778) [CES1](http://www.ncbi.nlm.nih.gov/gene/?term=1066)(122.778)[ACADSB](http://www.ncbi.nlm.nih.gov/gene/?term=36)(122.778)[SCN8A](http://www.ncbi.nlm.nih.gov/gene/?term=6334)(122.778)[COX7B](http://www.ncbi.nlm.nih.gov/gene/?term=1349)(122.778)[SCN1B](http://www.ncbi.nlm.nih.gov/gene/?term=6324)(122.778) [COX2](http://www.ncbi.nlm.nih.gov/gene/?term=4513)(122.778)[OGDH](http://www.ncbi.nlm.nih.gov/gene/?term=4967)(122.778)[HDAC2](http://www.ncbi.nlm.nih.gov/gene/?term=3066)(122.778)[COX8A](http://www.ncbi.nlm.nih.gov/gene/?term=1351)(122.778)[NR1H4](http://www.ncbi.nlm.nih.gov/gene/?term=9971)(122.778) [SUCLG2](http://www.ncbi.nlm.nih.gov/gene/?term=8801)(80.882)[SLC13A2](http://www.ncbi.nlm.nih.gov/gene/?term=9058)(80.882)[SLC13A1](http://www.ncbi.nlm.nih.gov/gene/?term=6561)(80.882)[SUCLG1](http://www.ncbi.nlm.nih.gov/gene/?term=8802)(80.882)[HSD17B6](http://www.ncbi.nlm.nih.gov/gene/?term=8630)(80.882) [SLC25A10](http://www.ncbi.nlm.nih.gov/gene/?term=1468)(80.882)[P3H3](http://www.ncbi.nlm.nih.gov/gene/?term=10536)(80.882)[OXCT2](http://www.ncbi.nlm.nih.gov/gene/?term=64064)(80.882)[TMLHE](http://www.ncbi.nlm.nih.gov/gene/?term=55217)(80.882)[PLOD1](http://www.ncbi.nlm.nih.gov/gene/?term=5351)(80.882) [SUCNR1](http://www.ncbi.nlm.nih.gov/gene/?term=56670)(80.882)[SLC13A3](http://www.ncbi.nlm.nih.gov/gene/?term=64849)(80.882)[ASPH](http://www.ncbi.nlm.nih.gov/gene/?term=444)(80.882)[BBOX1](http://www.ncbi.nlm.nih.gov/gene/?term=8424)(80.882)[SDHB](http://www.ncbi.nlm.nih.gov/gene/?term=6390)(80.882) [OXCT1](http://www.ncbi.nlm.nih.gov/gene/?term=5019)(80.882)[PLOD3](http://www.ncbi.nlm.nih.gov/gene/?term=8985)(80.882)[P4HA1](http://www.ncbi.nlm.nih.gov/gene/?term=5033)(80.882)[SDHC](http://www.ncbi.nlm.nih.gov/gene/?term=6391)(80.882)[P3H2](http://www.ncbi.nlm.nih.gov/gene/?term=55214)(80.882) [SDHA](http://www.ncbi.nlm.nih.gov/gene/?term=6389)(80.882)[P4HA2](http://www.ncbi.nlm.nih.gov/gene/?term=8974)(80.882)[SUCLA2](http://www.ncbi.nlm.nih.gov/gene/?term=8803)(80.882)[SDHD](http://www.ncbi.nlm.nih.gov/gene/?term=6392)(80.882)[P3H1](http://www.ncbi.nlm.nih.gov/gene/?term=64175)(80.882) [DCT](http://www.ncbi.nlm.nih.gov/gene/?term=1638)(55.444)[AKR1C3](http://www.ncbi.nlm.nih.gov/gene/?term=8644)(55.444)[CRTAP](http://www.ncbi.nlm.nih.gov/gene/?term=10491)(55.444)[SLIT2](http://www.ncbi.nlm.nih.gov/gene/?term=9353)(55.444)[ACO2](http://www.ncbi.nlm.nih.gov/gene/?term=50)(55.444) [JMJD6](http://www.ncbi.nlm.nih.gov/gene/?term=23210)(55.444)[SLC13A4](http://www.ncbi.nlm.nih.gov/gene/?term=26266)(55.444)[SDHAF2](http://www.ncbi.nlm.nih.gov/gene/?term=54949)(55.444)[SLC1A3](http://www.ncbi.nlm.nih.gov/gene/?term=6507)(55.444)[SALL1](http://www.ncbi.nlm.nih.gov/gene/?term=6299)(55.444) [HSD17B11](http://www.ncbi.nlm.nih.gov/gene/?term=51170)(55.444)[TYRP1](http://www.ncbi.nlm.nih.gov/gene/?term=7306)(55.444)[HIF1AN](http://www.ncbi.nlm.nih.gov/gene/?term=55662)(55.444)[UROS](http://www.ncbi.nlm.nih.gov/gene/?term=7390)(55.444)[SLC13A5](http://www.ncbi.nlm.nih.gov/gene/?term=284111)(55.444) [P4HB](http://www.ncbi.nlm.nih.gov/gene/?term=5034)(55.444)[CACNA2D1](http://www.ncbi.nlm.nih.gov/gene/?term=781)(48.000)[PLAT](http://www.ncbi.nlm.nih.gov/gene/?term=5327)(48.000)[SLC7A2](http://www.ncbi.nlm.nih.gov/gene/?term=6542)(48.000)[CACNA1A](http://www.ncbi.nlm.nih.gov/gene/?term=773)(48.000) [GRIN3B](http://www.ncbi.nlm.nih.gov/gene/?term=116444)(48.000)[CACNA2D2](http://www.ncbi.nlm.nih.gov/gene/?term=9254)(48.000)[GRIN2A](http://www.ncbi.nlm.nih.gov/gene/?term=2903)(48.000)[TOP1](http://www.ncbi.nlm.nih.gov/gene/?term=7150)(48.000)[PLG](http://www.ncbi.nlm.nih.gov/gene/?term=5340)(48.000) [CACNA1B](http://www.ncbi.nlm.nih.gov/gene/?term=774)(48.000)[GRIN2C](http://www.ncbi.nlm.nih.gov/gene/?term=2905)(48.000)[GRIN2B](http://www.ncbi.nlm.nih.gov/gene/?term=2904)(48.000)[SLC7A3](http://www.ncbi.nlm.nih.gov/gene/?term=84889)(48.000)[KARS](http://www.ncbi.nlm.nih.gov/gene/?term=3735)(48.000) [GRIN3A](http://www.ncbi.nlm.nih.gov/gene/?term=116443)(48.000)[SLC7A1](http://www.ncbi.nlm.nih.gov/gene/?term=6541)(48.000)[SLC7A4](http://www.ncbi.nlm.nih.gov/gene/?term=6545)(48.000)[GRIN2D](http://www.ncbi.nlm.nih.gov/gene/?term=2906)(48.000)[ADORA1](http://www.ncbi.nlm.nih.gov/gene/?term=134)(48.000) [GRIN1](http://www.ncbi.nlm.nih.gov/gene/?term=2902)(48.000)[HDAC1](http://www.ncbi.nlm.nih.gov/gene/?term=3065)(23.000)[GPD1L](http://www.ncbi.nlm.nih.gov/gene/?term=23171)(22.373)[SRD5A1](http://www.ncbi.nlm.nih.gov/gene/?term=6715)(22.373)[FBXO45](http://www.ncbi.nlm.nih.gov/gene/?term=200933)(22.373) [SIRT1](http://www.ncbi.nlm.nih.gov/gene/?term=23411)(22.373)[SHMT1](http://www.ncbi.nlm.nih.gov/gene/?term=6470)(22.373)[SUGCT](http://www.ncbi.nlm.nih.gov/gene/?term=79783)(22.373)[ACSS2](http://www.ncbi.nlm.nih.gov/gene/?term=55902)(22.373)[ARV1](http://www.ncbi.nlm.nih.gov/gene/?term=64801)(22.373) [SIX1](http://www.ncbi.nlm.nih.gov/gene/?term=6495)(22.373)[BDH1](http://www.ncbi.nlm.nih.gov/gene/?term=622)(22.373)[COLGALT2](http://www.ncbi.nlm.nih.gov/gene/?term=23127)(22.373)[SIX4](http://www.ncbi.nlm.nih.gov/gene/?term=51804)(22.373)[MRPS36](http://www.ncbi.nlm.nih.gov/gene/?term=92259)(22.373) [OSBPL8](http://www.ncbi.nlm.nih.gov/gene/?term=114882)(22.373)[SPI1](http://www.ncbi.nlm.nih.gov/gene/?term=6688)(22.373)[SUOX](http://www.ncbi.nlm.nih.gov/gene/?term=6821)(22.373)[CCL2](http://www.ncbi.nlm.nih.gov/gene/?term=6347)(22.373)[BCKDK](http://www.ncbi.nlm.nih.gov/gene/?term=10295)(22.373) [UBE2B](http://www.ncbi.nlm.nih.gov/gene/?term=7320)(22.373)[CYP1A2](http://www.ncbi.nlm.nih.gov/gene/?term=1544)(22.373)[CCL5](http://www.ncbi.nlm.nih.gov/gene/?term=6352)(22.373)[ANK2](http://www.ncbi.nlm.nih.gov/gene/?term=287)(22.373)[HSD17B2](http://www.ncbi.nlm.nih.gov/gene/?term=3294)(22.373) [STIM2](http://www.ncbi.nlm.nih.gov/gene/?term=57620)(22.373)[PKD2](http://www.ncbi.nlm.nih.gov/gene/?term=5311)(22.373)[ALDH9A1](http://www.ncbi.nlm.nih.gov/gene/?term=223)(22.373)[TH](http://www.ncbi.nlm.nih.gov/gene/?term=7054)(22.373)[EPO](http://www.ncbi.nlm.nih.gov/gene/?term=2056)(22.373) [COLGALT1](http://www.ncbi.nlm.nih.gov/gene/?term=79709)(22.373)[NEDD4](http://www.ncbi.nlm.nih.gov/gene/?term=4734)(22.373)[CYP11A1](http://www.ncbi.nlm.nih.gov/gene/?term=1583)(22.373)[DHODH](http://www.ncbi.nlm.nih.gov/gene/?term=1723)(22.373)[CLN3](http://www.ncbi.nlm.nih.gov/gene/?term=1201)(22.373) [ARID1A](http://www.ncbi.nlm.nih.gov/gene/?term=8289)(22.373)[EGLN2](http://www.ncbi.nlm.nih.gov/gene/?term=112398)(22.373)[ACAT1](http://www.ncbi.nlm.nih.gov/gene/?term=38)(22.373)[GALC](http://www.ncbi.nlm.nih.gov/gene/?term=2581)(22.373)[PLCG2](http://www.ncbi.nlm.nih.gov/gene/?term=5336)(22.373) [ACOT4](http://www.ncbi.nlm.nih.gov/gene/?term=122970)(22.373)[PHGDH](http://www.ncbi.nlm.nih.gov/gene/?term=26227)(22.373)[PNLIPRP2](http://www.ncbi.nlm.nih.gov/gene/?term=5408)(22.373)[CYP39A1](http://www.ncbi.nlm.nih.gov/gene/?term=51302)(22.373)[BCKDHA](http://www.ncbi.nlm.nih.gov/gene/?term=593)(22.373) [SLC25A12](http://www.ncbi.nlm.nih.gov/gene/?term=8604)(22.373)[YBX3](http://www.ncbi.nlm.nih.gov/gene/?term=8531)(22.373)[8-Mar](http://www.ncbi.nlm.nih.gov/gene/?term=220972)(22.373)[STIM1](http://www.ncbi.nlm.nih.gov/gene/?term=6786)(22.373)[GAL3ST1](http://www.ncbi.nlm.nih.gov/gene/?term=9514)(22.373) [CDH11](http://www.ncbi.nlm.nih.gov/gene/?term=1009)(22.373)[CACNA1D](http://www.ncbi.nlm.nih.gov/gene/?term=776)(22.373)[DDC](http://www.ncbi.nlm.nih.gov/gene/?term=1644)(22.373)[DHTKD1](http://www.ncbi.nlm.nih.gov/gene/?term=55526)(22.373)[NEDD4L](http://www.ncbi.nlm.nih.gov/gene/?term=23327)(22.373) [BDKRB2](http://www.ncbi.nlm.nih.gov/gene/?term=624)(22.373)[PRPH](http://www.ncbi.nlm.nih.gov/gene/?term=5630)(22.373)[BCKDHB](http://www.ncbi.nlm.nih.gov/gene/?term=594)(22.373)[KCNE5](http://www.ncbi.nlm.nih.gov/gene/?term=23630)(22.373)[SRD5A3](http://www.ncbi.nlm.nih.gov/gene/?term=79644)(22.373) [SMAD7](http://www.ncbi.nlm.nih.gov/gene/?term=4092)(22.373)[PRDM8](http://www.ncbi.nlm.nih.gov/gene/?term=56978)(22.373)[UROD](http://www.ncbi.nlm.nih.gov/gene/?term=7389)(22.373)[UGT8](http://www.ncbi.nlm.nih.gov/gene/?term=7368)(22.373)[ETHE1](http://www.ncbi.nlm.nih.gov/gene/?term=23474)(22.373) [WNT10B](http://www.ncbi.nlm.nih.gov/gene/?term=7480)(22.373)[OGFOD1](http://www.ncbi.nlm.nih.gov/gene/?term=55239)(22.373)[SLC38A7](http://www.ncbi.nlm.nih.gov/gene/?term=55238)(22.373)[ACSS1](http://www.ncbi.nlm.nih.gov/gene/?term=84532)(22.373)[CYP4B1](http://www.ncbi.nlm.nih.gov/gene/?term=1580)(22.373) [STAR](http://www.ncbi.nlm.nih.gov/gene/?term=6770)(22.373)[DLST](http://www.ncbi.nlm.nih.gov/gene/?term=1743)(22.373)[PF4](http://www.ncbi.nlm.nih.gov/gene/?term=5196)(22.373)[CRACR2A](http://www.ncbi.nlm.nih.gov/gene/?term=84766)(22.373)[HSD17B1](http://www.ncbi.nlm.nih.gov/gene/?term=3292)(22.373) [CAMK2D](http://www.ncbi.nlm.nih.gov/gene/?term=817)(22.373)[AP3D1](http://www.ncbi.nlm.nih.gov/gene/?term=8943)(22.373)[MYO5A](http://www.ncbi.nlm.nih.gov/gene/?term=4644)(22.373)[ILVBL](http://www.ncbi.nlm.nih.gov/gene/?term=10994)(22.373)[CAV3](http://www.ncbi.nlm.nih.gov/gene/?term=859)(22.373) [HTT](http://www.ncbi.nlm.nih.gov/gene/?term=3064)(22.373)[IL10](http://www.ncbi.nlm.nih.gov/gene/?term=3586)(22.373)[EGLN3](http://www.ncbi.nlm.nih.gov/gene/?term=112399)(22.373)[OGDHL](http://www.ncbi.nlm.nih.gov/gene/?term=55753)(22.373)[KCNA5](http://www.ncbi.nlm.nih.gov/gene/?term=3741)(22.373) [TST](http://www.ncbi.nlm.nih.gov/gene/?term=7263)(22.373)[SLC1A6](http://www.ncbi.nlm.nih.gov/gene/?term=6511)(22.373)[NPPA](http://www.ncbi.nlm.nih.gov/gene/?term=4878)(22.373)[ACADM](http://www.ncbi.nlm.nih.gov/gene/?term=34)(22.373)[SQRDL](http://www.ncbi.nlm.nih.gov/gene/?term=58472)(22.373) [TMEM110](http://www.ncbi.nlm.nih.gov/gene/?term=375346)(22.373)[CDC42](http://www.ncbi.nlm.nih.gov/gene/?term=998)(22.373)[ARHGEF2](http://www.ncbi.nlm.nih.gov/gene/?term=9181)(22.373)[EGLN1](http://www.ncbi.nlm.nih.gov/gene/?term=54583)(22.373)[ETFDH](http://www.ncbi.nlm.nih.gov/gene/?term=2110)(22.373) [EPHA4](http://www.ncbi.nlm.nih.gov/gene/?term=2043)(22.373)[OCA2](http://www.ncbi.nlm.nih.gov/gene/?term=4948)(22.373)[HSD17B8](http://www.ncbi.nlm.nih.gov/gene/?term=7923)(22.373)[CTNNB1](http://www.ncbi.nlm.nih.gov/gene/?term=1499)(22.373)[P4HA3](http://www.ncbi.nlm.nih.gov/gene/?term=283208)(22.373) [EDA](http://www.ncbi.nlm.nih.gov/gene/?term=1896)(22.373)[HACL1](http://www.ncbi.nlm.nih.gov/gene/?term=26061)(22.373)[RYR2](http://www.ncbi.nlm.nih.gov/gene/?term=6262)(22.373)[HAP1](http://www.ncbi.nlm.nih.gov/gene/?term=9001)(22.373)[PCSK9](http://www.ncbi.nlm.nih.gov/gene/?term=255738)(22.373) [KCNQ1](http://www.ncbi.nlm.nih.gov/gene/?term=3784)(22.373)[CASQ2](http://www.ncbi.nlm.nih.gov/gene/?term=845)(22.373)[GJA5](http://www.ncbi.nlm.nih.gov/gene/?term=2702)(22.373)[DHRS9](http://www.ncbi.nlm.nih.gov/gene/?term=10170)(22.373)[GPR143](http://www.ncbi.nlm.nih.gov/gene/?term=4935)(22.373) [UGT1A1](http://www.ncbi.nlm.nih.gov/gene/?term=54658)(22.373)[DLD](http://www.ncbi.nlm.nih.gov/gene/?term=1738)(22.373)[GNAS](http://www.ncbi.nlm.nih.gov/gene/?term=2778)(22.373)[SLC25A13](http://www.ncbi.nlm.nih.gov/gene/?term=10165)(22.373)[SFXN5](http://www.ncbi.nlm.nih.gov/gene/?term=94097)(22.373) [PLOD2](http://www.ncbi.nlm.nih.gov/gene/?term=5352)(22.373)[CYP1A1](http://www.ncbi.nlm.nih.gov/gene/?term=1543)(22.373)[SULT2A1](http://www.ncbi.nlm.nih.gov/gene/?term=6822)(22.373)[NFX1](http://www.ncbi.nlm.nih.gov/gene/?term=4799)(22.373)[DBT](http://www.ncbi.nlm.nih.gov/gene/?term=1629)(22.373) [SLC25A1](http://www.ncbi.nlm.nih.gov/gene/?term=6576)(22.373) | |
| [Chrysanthemaxanthin](http://www.megabionet.org/tcmid/ingredient/3592/) | [ADA](http://www.ncbi.nlm.nih.gov/gene/?term=100)(23.000)[ITFG2](http://www.ncbi.nlm.nih.gov/gene/?term=55846)(22.373)[DGUOK](http://www.ncbi.nlm.nih.gov/gene/?term=1716)(22.373)[XDH](http://www.ncbi.nlm.nih.gov/gene/?term=7498)(22.373)[ADORA1](http://www.ncbi.nlm.nih.gov/gene/?term=134)(22.373) | |
| [Lupeol](http://www.megabionet.org/tcmid/ingredient/13091/) | [VDR](http://www.ncbi.nlm.nih.gov/gene/?term=7421)(80.882)[CYP27B1](http://www.ncbi.nlm.nih.gov/gene/?term=1594)(80.882)[GC](http://www.ncbi.nlm.nih.gov/gene/?term=2638)(55.444)[SNW1](http://www.ncbi.nlm.nih.gov/gene/?term=22938)(55.444)[TRPV3](http://www.ncbi.nlm.nih.gov/gene/?term=162514)(48.000) [ESR1](http://www.ncbi.nlm.nih.gov/gene/?term=2099)(48.000)[PGR](http://www.ncbi.nlm.nih.gov/gene/?term=5241)(48.000)[OPRK1](http://www.ncbi.nlm.nih.gov/gene/?term=4986)(48.000)[TRPM8](http://www.ncbi.nlm.nih.gov/gene/?term=79054)(48.000)[TRPA1](http://www.ncbi.nlm.nih.gov/gene/?term=8989)(48.000) [NFKB1](http://www.ncbi.nlm.nih.gov/gene/?term=4790)(22.373)[AKR1C3](http://www.ncbi.nlm.nih.gov/gene/?term=8644)(22.373)[CYP24A1](http://www.ncbi.nlm.nih.gov/gene/?term=1591)(22.373)[GPBAR1](http://www.ncbi.nlm.nih.gov/gene/?term=151306)(22.373)[SNAI2](http://www.ncbi.nlm.nih.gov/gene/?term=6591)(22.373) [MED1](http://www.ncbi.nlm.nih.gov/gene/?term=5469)(22.373)[SNAI1](http://www.ncbi.nlm.nih.gov/gene/?term=6615)(22.373)[CYP3A4](http://www.ncbi.nlm.nih.gov/gene/?term=1576)(22.373)[CALB1](http://www.ncbi.nlm.nih.gov/gene/?term=793)(22.373)[FGF23](http://www.ncbi.nlm.nih.gov/gene/?term=8074)(22.373) [GFI1](http://www.ncbi.nlm.nih.gov/gene/?term=2672)(22.373)[LANCL2](http://www.ncbi.nlm.nih.gov/gene/?term=55915)(22.373)[WNT4](http://www.ncbi.nlm.nih.gov/gene/?term=54361)(22.373)[TCF3](http://www.ncbi.nlm.nih.gov/gene/?term=6929)(22.373)[BAX](http://www.ncbi.nlm.nih.gov/gene/?term=581)(22.373) [KL](http://www.ncbi.nlm.nih.gov/gene/?term=9365)(22.373)[CYP2R1](http://www.ncbi.nlm.nih.gov/gene/?term=120227)(22.373)[PML](http://www.ncbi.nlm.nih.gov/gene/?term=5371)(22.373)[B4GALT1](http://www.ncbi.nlm.nih.gov/gene/?term=2683)(22.373)[S100G](http://www.ncbi.nlm.nih.gov/gene/?term=795)(22.373) [CYP27A1](http://www.ncbi.nlm.nih.gov/gene/?term=1593)(22.373)[KANK2](http://www.ncbi.nlm.nih.gov/gene/?term=25959)(22.373)[IRX5](http://www.ncbi.nlm.nih.gov/gene/?term=10265)(22.373)[RXRA](http://www.ncbi.nlm.nih.gov/gene/?term=6256)(22.373)[NR1H4](http://www.ncbi.nlm.nih.gov/gene/?term=9971)(22.373) [TRIM24](http://www.ncbi.nlm.nih.gov/gene/?term=8805)(22.373) | |
| [Methyl Linoleate](http://www.megabionet.org/tcmid/ingredient/14546/) | [SLC8A1](http://www.ncbi.nlm.nih.gov/gene/?term=6546)(48.000)[F12](http://www.ncbi.nlm.nih.gov/gene/?term=2161)(48.000)[ESR1](http://www.ncbi.nlm.nih.gov/gene/?term=2099)(48.000)[TRPV1](http://www.ncbi.nlm.nih.gov/gene/?term=7442)(48.000)[PTGER4](http://www.ncbi.nlm.nih.gov/gene/?term=5734)(48.000) [PGR](http://www.ncbi.nlm.nih.gov/gene/?term=5241)(48.000)[AR](http://www.ncbi.nlm.nih.gov/gene/?term=367)(48.000)[FADS2](http://www.ncbi.nlm.nih.gov/gene/?term=9415)(48.000)[FADS1](http://www.ncbi.nlm.nih.gov/gene/?term=3992)(48.000)[PTGER2](http://www.ncbi.nlm.nih.gov/gene/?term=5732)(48.000) [PTGS1](http://www.ncbi.nlm.nih.gov/gene/?term=5742)(48.000)[PTGER3](http://www.ncbi.nlm.nih.gov/gene/?term=5733)(48.000)[PTGS2](http://www.ncbi.nlm.nih.gov/gene/?term=5743)(48.000)[ELOVL4](http://www.ncbi.nlm.nih.gov/gene/?term=6785)(48.000)[NR3C2](http://www.ncbi.nlm.nih.gov/gene/?term=4306)(48.000) | |
| [Sinapic Acid](http://www.megabionet.org/tcmid/ingredient/23139/) | This compound doesn't have any potential target with score larger than 20. | |
| [Licoricesaponine C2](http://www.megabionet.org/tcmid/ingredient/31433/) | [GLRA3](http://www.ncbi.nlm.nih.gov/gene/?term=8001)(48.000)[NR3C1](http://www.ncbi.nlm.nih.gov/gene/?term=2908)(48.000)[GABRB3](http://www.ncbi.nlm.nih.gov/gene/?term=2562)(48.000) | |
| [Nona-2,4-Dienal](http://www.megabionet.org/tcmid/ingredient/23945/) | [ACHE](http://www.ncbi.nlm.nih.gov/gene/?term=43)(80.882)[BCHE](http://www.ncbi.nlm.nih.gov/gene/?term=590)(80.882)[COLQ](http://www.ncbi.nlm.nih.gov/gene/?term=8292)(55.444)[SLC5A7](http://www.ncbi.nlm.nih.gov/gene/?term=60482)(22.373)[NRG1](http://www.ncbi.nlm.nih.gov/gene/?term=3084)(22.373) [DMGDH](http://www.ncbi.nlm.nih.gov/gene/?term=29958)(22.373)[SIX3](http://www.ncbi.nlm.nih.gov/gene/?term=6496)(22.373)[CRP](http://www.ncbi.nlm.nih.gov/gene/?term=1401)(22.373)[PRSS12](http://www.ncbi.nlm.nih.gov/gene/?term=8492)(22.373)[SLC44A4](http://www.ncbi.nlm.nih.gov/gene/?term=80736)(22.373) [ALDH7A1](http://www.ncbi.nlm.nih.gov/gene/?term=501)(22.373)[DNM3](http://www.ncbi.nlm.nih.gov/gene/?term=26052)(22.373)[ASCL1](http://www.ncbi.nlm.nih.gov/gene/?term=429)(22.373)[ENPP6](http://www.ncbi.nlm.nih.gov/gene/?term=133121)(22.373)[CDH8](http://www.ncbi.nlm.nih.gov/gene/?term=1006)(22.373) [FNTA](http://www.ncbi.nlm.nih.gov/gene/?term=2339)(22.373)[AGRN](http://www.ncbi.nlm.nih.gov/gene/?term=375790)(22.373)[CHKA](http://www.ncbi.nlm.nih.gov/gene/?term=1119)(22.373)[CHDH](http://www.ncbi.nlm.nih.gov/gene/?term=55349)(22.373)[GRIN1](http://www.ncbi.nlm.nih.gov/gene/?term=2902)(22.373) | |
| [Licorisoflavan A](http://www.megabionet.org/tcmid/ingredient/12789/) | [SOAT1](http://www.ncbi.nlm.nih.gov/gene/?term=6646)(23.000)[MTTP](http://www.ncbi.nlm.nih.gov/gene/?term=4547)(23.000)[CNR2](http://www.ncbi.nlm.nih.gov/gene/?term=1269)(23.000)[CNR1](http://www.ncbi.nlm.nih.gov/gene/?term=1268)(23.000)[SOAT2](http://www.ncbi.nlm.nih.gov/gene/?term=8435)(23.000) [DRD2](http://www.ncbi.nlm.nih.gov/gene/?term=1813)(22.373)[PAWR](http://www.ncbi.nlm.nih.gov/gene/?term=5074)(22.373) | |
| [Isoramanone](http://www.megabionet.org/tcmid/ingredient/11640/) | [SRD5A1](http://www.ncbi.nlm.nih.gov/gene/?term=6715)(48.000)[ESR1](http://www.ncbi.nlm.nih.gov/gene/?term=2099)(48.000)[PTGER1](http://www.ncbi.nlm.nih.gov/gene/?term=5731)(48.000)[PTGER4](http://www.ncbi.nlm.nih.gov/gene/?term=5734)(48.000)[PGR](http://www.ncbi.nlm.nih.gov/gene/?term=5241)(48.000) [AR](http://www.ncbi.nlm.nih.gov/gene/?term=367)(48.000)[NR3C1](http://www.ncbi.nlm.nih.gov/gene/?term=2908)(48.000)[PTGIR](http://www.ncbi.nlm.nih.gov/gene/?term=5739)(48.000)[PTGER2](http://www.ncbi.nlm.nih.gov/gene/?term=5732)(48.000)[PTGER3](http://www.ncbi.nlm.nih.gov/gene/?term=5733)(48.000) [PTGFR](http://www.ncbi.nlm.nih.gov/gene/?term=5737)(48.000)[ANXA1](http://www.ncbi.nlm.nih.gov/gene/?term=301)(48.000)[VDR](http://www.ncbi.nlm.nih.gov/gene/?term=7421)(48.000)[NR3C2](http://www.ncbi.nlm.nih.gov/gene/?term=4306)(48.000)[ATP1A1](http://www.ncbi.nlm.nih.gov/gene/?term=476)(23.000) [HCAR2](http://www.ncbi.nlm.nih.gov/gene/?term=338442)(22.373)[NOTCH2](http://www.ncbi.nlm.nih.gov/gene/?term=4853)(22.373)[BIN3](http://www.ncbi.nlm.nih.gov/gene/?term=55909)(22.373)[ZRANB3](http://www.ncbi.nlm.nih.gov/gene/?term=84083)(22.373) | |
| [N-Octadecane](http://www.megabionet.org/tcmid/ingredient/15938/) | This compound doesn't have any potential target with score larger than 20. | |
| [Gancaonin F](http://www.megabionet.org/tcmid/ingredient/8138/) | [CNR2](http://www.ncbi.nlm.nih.gov/gene/?term=1269)(23.000)[CNR1](http://www.ncbi.nlm.nih.gov/gene/?term=1268)(23.000)[DRD2](http://www.ncbi.nlm.nih.gov/gene/?term=1813)(22.373) | |
| [Tangshenoside I](http://www.megabionet.org/tcmid/ingredient/20656/) | This compound doesn't have any potential target with score larger than 20. | |
| [N-Pentadecane](http://www.megabionet.org/tcmid/ingredient/16817/) | This compound doesn't have any potential target with score larger than 20. | |
| [Glycyrol](http://www.megabionet.org/tcmid/ingredient/8838/) | [CNR2](http://www.ncbi.nlm.nih.gov/gene/?term=1269)(23.000)[CNR1](http://www.ncbi.nlm.nih.gov/gene/?term=1268)(23.000)[DRD2](http://www.ncbi.nlm.nih.gov/gene/?term=1813)(22.373) | |
| [Betanidin](http://www.megabionet.org/tcmid/ingredient/23207/) | This compound doesn't have any potential target with score larger than 20. | |
| [N-Butyl Allophanate](http://www.megabionet.org/tcmid/ingredient/2788/) | This compound doesn't have any potential target with score larger than 20. | |
| [Dimethyl Sebacate](http://www.megabionet.org/tcmid/ingredient/6405/) | [NPR1](http://www.ncbi.nlm.nih.gov/gene/?term=4881)(80.882)[SCN11A](http://www.ncbi.nlm.nih.gov/gene/?term=11280)(48.000)[SCN2B](http://www.ncbi.nlm.nih.gov/gene/?term=6327)(48.000)[ABAT](http://www.ncbi.nlm.nih.gov/gene/?term=18)(48.000)[SCN1A](http://www.ncbi.nlm.nih.gov/gene/?term=6323)(48.000) [ESRRG](http://www.ncbi.nlm.nih.gov/gene/?term=2104)(48.000)[SCN3B](http://www.ncbi.nlm.nih.gov/gene/?term=55800)(48.000)[COX6C](http://www.ncbi.nlm.nih.gov/gene/?term=1345)(48.000)[COX5B](http://www.ncbi.nlm.nih.gov/gene/?term=1329)(48.000)[COX7C](http://www.ncbi.nlm.nih.gov/gene/?term=1350)(48.000) [COX1](http://www.ncbi.nlm.nih.gov/gene/?term=4512)(48.000)[SCN3A](http://www.ncbi.nlm.nih.gov/gene/?term=6328)(48.000)[AKR1C2](http://www.ncbi.nlm.nih.gov/gene/?term=1646)(48.000)[SCN7A](http://www.ncbi.nlm.nih.gov/gene/?term=6332)(48.000)[SCN5A](http://www.ncbi.nlm.nih.gov/gene/?term=6331)(48.000) [SCN10A](http://www.ncbi.nlm.nih.gov/gene/?term=6336)(48.000)[AKR1D1](http://www.ncbi.nlm.nih.gov/gene/?term=6718)(48.000)[ALDH5A1](http://www.ncbi.nlm.nih.gov/gene/?term=7915)(48.000)[SCN2A](http://www.ncbi.nlm.nih.gov/gene/?term=6326)(48.000)[COX5A](http://www.ncbi.nlm.nih.gov/gene/?term=9377)(48.000) [COX3](http://www.ncbi.nlm.nih.gov/gene/?term=4514)(48.000)[AR](http://www.ncbi.nlm.nih.gov/gene/?term=367)(48.000)[COX7A1](http://www.ncbi.nlm.nih.gov/gene/?term=1346)(48.000)[FECH](http://www.ncbi.nlm.nih.gov/gene/?term=2235)(48.000)[COX4I1](http://www.ncbi.nlm.nih.gov/gene/?term=1327)(48.000) [HDAC9](http://www.ncbi.nlm.nih.gov/gene/?term=9734)(48.000)[PLA2G1B](http://www.ncbi.nlm.nih.gov/gene/?term=5319)(48.000)[COX6A2](http://www.ncbi.nlm.nih.gov/gene/?term=1339)(48.000)[TYR](http://www.ncbi.nlm.nih.gov/gene/?term=7299)(48.000)[SCN9A](http://www.ncbi.nlm.nih.gov/gene/?term=6335)(48.000) [SCN4A](http://www.ncbi.nlm.nih.gov/gene/?term=6329)(48.000)[SCN4B](http://www.ncbi.nlm.nih.gov/gene/?term=6330)(48.000)[ADH1C](http://www.ncbi.nlm.nih.gov/gene/?term=126)(48.000)[COX6B1](http://www.ncbi.nlm.nih.gov/gene/?term=1340)(48.000)[FABP6](http://www.ncbi.nlm.nih.gov/gene/?term=2172)(48.000) [CES1](http://www.ncbi.nlm.nih.gov/gene/?term=1066)(48.000)[SRD5A2](http://www.ncbi.nlm.nih.gov/gene/?term=6716)(48.000)[ACADSB](http://www.ncbi.nlm.nih.gov/gene/?term=36)(48.000)[SCN8A](http://www.ncbi.nlm.nih.gov/gene/?term=6334)(48.000)[COX7B](http://www.ncbi.nlm.nih.gov/gene/?term=1349)(48.000) [SCN1B](http://www.ncbi.nlm.nih.gov/gene/?term=6324)(48.000)[COX2](http://www.ncbi.nlm.nih.gov/gene/?term=4513)(48.000)[OGDH](http://www.ncbi.nlm.nih.gov/gene/?term=4967)(48.000)[HDAC2](http://www.ncbi.nlm.nih.gov/gene/?term=3066)(48.000)[COX8A](http://www.ncbi.nlm.nih.gov/gene/?term=1351)(48.000) [NR1H4](http://www.ncbi.nlm.nih.gov/gene/?term=9971)(48.000)[NPR3](http://www.ncbi.nlm.nih.gov/gene/?term=4883)(22.373)[NPR2](http://www.ncbi.nlm.nih.gov/gene/?term=4882)(22.373) | |
| [Schaftoside](http://www.megabionet.org/tcmid/ingredient/19448/) | This compound doesn't have any potential target with score larger than 20. | |
| [Vicianin](http://www.megabionet.org/tcmid/ingredient/22449/) | This compound doesn't have any potential target with score larger than 20. | |
| [Butyl-Cyclohexane](http://www.megabionet.org/tcmid/ingredient/2790/) | This compound doesn't have any potential target with score larger than 20. | |
| [Astragaloside Iv](http://www.megabionet.org/tcmid/ingredient/1939/) | This compound doesn't have any potential target with score larger than 20. | |
| [Heptadecane](http://www.megabionet.org/tcmid/ingredient/9377/) | This compound doesn't have any potential target with score larger than 20. | |
| [Syringaldehyde](http://www.megabionet.org/tcmid/ingredient/20538/) | This compound doesn't have any potential target with score larger than 20. | |
| [2,4-Nonadienal](http://www.megabionet.org/tcmid/ingredient/15666/) | [ACHE](http://www.ncbi.nlm.nih.gov/gene/?term=43)(80.882)[BCHE](http://www.ncbi.nlm.nih.gov/gene/?term=590)(80.882)[COLQ](http://www.ncbi.nlm.nih.gov/gene/?term=8292)(55.444)[SLC5A7](http://www.ncbi.nlm.nih.gov/gene/?term=60482)(22.373)[NRG1](http://www.ncbi.nlm.nih.gov/gene/?term=3084)(22.373) [DMGDH](http://www.ncbi.nlm.nih.gov/gene/?term=29958)(22.373)[SIX3](http://www.ncbi.nlm.nih.gov/gene/?term=6496)(22.373)[CRP](http://www.ncbi.nlm.nih.gov/gene/?term=1401)(22.373)[PRSS12](http://www.ncbi.nlm.nih.gov/gene/?term=8492)(22.373)[SLC44A4](http://www.ncbi.nlm.nih.gov/gene/?term=80736)(22.373) [ALDH7A1](http://www.ncbi.nlm.nih.gov/gene/?term=501)(22.373)[DNM3](http://www.ncbi.nlm.nih.gov/gene/?term=26052)(22.373)[ASCL1](http://www.ncbi.nlm.nih.gov/gene/?term=429)(22.373)[ENPP6](http://www.ncbi.nlm.nih.gov/gene/?term=133121)(22.373)[CDH8](http://www.ncbi.nlm.nih.gov/gene/?term=1006)(22.373) [FNTA](http://www.ncbi.nlm.nih.gov/gene/?term=2339)(22.373)[AGRN](http://www.ncbi.nlm.nih.gov/gene/?term=375790)(22.373)[CHKA](http://www.ncbi.nlm.nih.gov/gene/?term=1119)(22.373)[CHDH](http://www.ncbi.nlm.nih.gov/gene/?term=55349)(22.373)[GRIN1](http://www.ncbi.nlm.nih.gov/gene/?term=2902)(22.373) | |
| [Rhamnocitrin](http://www.megabionet.org/tcmid/ingredient/18667/) | This compound doesn't have any potential target with score larger than 20. | |
| [Stigmasta-5,22-Dien-3-One](http://www.megabionet.org/tcmid/ingredient/20335/) | [CYP17A1](http://www.ncbi.nlm.nih.gov/gene/?term=1586)(122.778)[ESR1](http://www.ncbi.nlm.nih.gov/gene/?term=2099)(122.778)[PGR](http://www.ncbi.nlm.nih.gov/gene/?term=5241)(122.778)[OPRK1](http://www.ncbi.nlm.nih.gov/gene/?term=4986)(122.778)[NR3C2](http://www.ncbi.nlm.nih.gov/gene/?term=4306)(122.778) [PDE7B](http://www.ncbi.nlm.nih.gov/gene/?term=27115)(80.882)[PDE5A](http://www.ncbi.nlm.nih.gov/gene/?term=8654)(80.882)[PDE9A](http://www.ncbi.nlm.nih.gov/gene/?term=5152)(80.882)[PDE4A](http://www.ncbi.nlm.nih.gov/gene/?term=5141)(80.882)[RYR1](http://www.ncbi.nlm.nih.gov/gene/?term=6261)(80.882) [PDE3A](http://www.ncbi.nlm.nih.gov/gene/?term=5139)(80.882)[ADORA2A](http://www.ncbi.nlm.nih.gov/gene/?term=135)(80.882)[ITPR1](http://www.ncbi.nlm.nih.gov/gene/?term=3708)(80.882)[PDE6A](http://www.ncbi.nlm.nih.gov/gene/?term=5145)(80.882)[PRKDC](http://www.ncbi.nlm.nih.gov/gene/?term=5591)(80.882) [PIK3CD](http://www.ncbi.nlm.nih.gov/gene/?term=5293)(80.882)[PDE3B](http://www.ncbi.nlm.nih.gov/gene/?term=5140)(80.882)[PGD](http://www.ncbi.nlm.nih.gov/gene/?term=5226)(80.882)[PIK3CA](http://www.ncbi.nlm.nih.gov/gene/?term=5290)(80.882)[ADORA2B](http://www.ncbi.nlm.nih.gov/gene/?term=136)(80.882) [PDE1A](http://www.ncbi.nlm.nih.gov/gene/?term=5136)(80.882)[PDE4D](http://www.ncbi.nlm.nih.gov/gene/?term=5144)(80.882)[PIK3CB](http://www.ncbi.nlm.nih.gov/gene/?term=5291)(80.882)[ITPR2](http://www.ncbi.nlm.nih.gov/gene/?term=3709)(80.882)[PDE1B](http://www.ncbi.nlm.nih.gov/gene/?term=5153)(80.882) [PDE7A](http://www.ncbi.nlm.nih.gov/gene/?term=5150)(80.882)[ITPR3](http://www.ncbi.nlm.nih.gov/gene/?term=3710)(80.882)[POLA2](http://www.ncbi.nlm.nih.gov/gene/?term=23649)(80.882)[PDE4C](http://www.ncbi.nlm.nih.gov/gene/?term=5143)(80.882)[PDE10A](http://www.ncbi.nlm.nih.gov/gene/?term=10846)(80.882) [PDE1C](http://www.ncbi.nlm.nih.gov/gene/?term=5137)(80.882)[ATM](http://www.ncbi.nlm.nih.gov/gene/?term=472)(80.882)[PDE6B](http://www.ncbi.nlm.nih.gov/gene/?term=5158)(80.882)[PDE4B](http://www.ncbi.nlm.nih.gov/gene/?term=5142)(80.882)[PDE2A](http://www.ncbi.nlm.nih.gov/gene/?term=5138)(80.882) [ADORA1](http://www.ncbi.nlm.nih.gov/gene/?term=134)(80.882)[CYP19A1](http://www.ncbi.nlm.nih.gov/gene/?term=1588)(80.882)[PDE8B](http://www.ncbi.nlm.nih.gov/gene/?term=8622)(80.882)[NT5E](http://www.ncbi.nlm.nih.gov/gene/?term=4907)(80.882)[HDAC2](http://www.ncbi.nlm.nih.gov/gene/?term=3066)(80.882) [PDE6C](http://www.ncbi.nlm.nih.gov/gene/?term=5146)(80.882)[PDE11A](http://www.ncbi.nlm.nih.gov/gene/?term=50940)(80.882)[PDE8A](http://www.ncbi.nlm.nih.gov/gene/?term=5151)(80.882)[RINT1](http://www.ncbi.nlm.nih.gov/gene/?term=60561)(55.444)[RIPK1](http://www.ncbi.nlm.nih.gov/gene/?term=8737)(55.444) [PIK3R1](http://www.ncbi.nlm.nih.gov/gene/?term=5295)(55.444)[WNT4](http://www.ncbi.nlm.nih.gov/gene/?term=54361)(55.444)[TACR2](http://www.ncbi.nlm.nih.gov/gene/?term=6865)(55.444)[AMPD3](http://www.ncbi.nlm.nih.gov/gene/?term=272)(55.444)[CX3CR1](http://www.ncbi.nlm.nih.gov/gene/?term=1524)(55.444) [ADA](http://www.ncbi.nlm.nih.gov/gene/?term=100)(55.444)[HAP1](http://www.ncbi.nlm.nih.gov/gene/?term=9001)(55.444)[IDNK](http://www.ncbi.nlm.nih.gov/gene/?term=414328)(55.444)[SRD5A1](http://www.ncbi.nlm.nih.gov/gene/?term=6715)(48.000)[AR](http://www.ncbi.nlm.nih.gov/gene/?term=367)(48.000) [NR3C1](http://www.ncbi.nlm.nih.gov/gene/?term=2908)(48.000)[ANXA1](http://www.ncbi.nlm.nih.gov/gene/?term=301)(48.000)[HDAC1](http://www.ncbi.nlm.nih.gov/gene/?term=3065)(23.000)[TFAP2C](http://www.ncbi.nlm.nih.gov/gene/?term=7022)(22.373)[HMGA2](http://www.ncbi.nlm.nih.gov/gene/?term=8091)(22.373) [TNFSF11](http://www.ncbi.nlm.nih.gov/gene/?term=8600)(22.373)[NT5C1A](http://www.ncbi.nlm.nih.gov/gene/?term=84618)(22.373)[ACTN3](http://www.ncbi.nlm.nih.gov/gene/?term=89)(22.373)[NOS1AP](http://www.ncbi.nlm.nih.gov/gene/?term=9722)(22.373)[TAC1](http://www.ncbi.nlm.nih.gov/gene/?term=6863)(22.373) [PHB](http://www.ncbi.nlm.nih.gov/gene/?term=5245)(22.373)[SIRT1](http://www.ncbi.nlm.nih.gov/gene/?term=23411)(22.373)[DGKI](http://www.ncbi.nlm.nih.gov/gene/?term=9162)(22.373)[FKBP1A](http://www.ncbi.nlm.nih.gov/gene/?term=2280)(22.373)[FURIN](http://www.ncbi.nlm.nih.gov/gene/?term=5045)(22.373) [TOX3](http://www.ncbi.nlm.nih.gov/gene/?term=27324)(22.373)[UTS2](http://www.ncbi.nlm.nih.gov/gene/?term=10911)(22.373)[STUB1](http://www.ncbi.nlm.nih.gov/gene/?term=10273)(22.373)[CTR9](http://www.ncbi.nlm.nih.gov/gene/?term=9646)(22.373)[G6PD](http://www.ncbi.nlm.nih.gov/gene/?term=2539)(22.373) [GNAT1](http://www.ncbi.nlm.nih.gov/gene/?term=2779)(22.373)[SIX1](http://www.ncbi.nlm.nih.gov/gene/?term=6495)(22.373)[QDPR](http://www.ncbi.nlm.nih.gov/gene/?term=5860)(22.373)[TGFB1](http://www.ncbi.nlm.nih.gov/gene/?term=7040)(22.373)[ATP11C](http://www.ncbi.nlm.nih.gov/gene/?term=286410)(22.373) [KHDRBS1](http://www.ncbi.nlm.nih.gov/gene/?term=10657)(22.373)[PPP3CB](http://www.ncbi.nlm.nih.gov/gene/?term=5532)(22.373)[SYT2](http://www.ncbi.nlm.nih.gov/gene/?term=127833)(22.373)[SIX4](http://www.ncbi.nlm.nih.gov/gene/?term=51804)(22.373)[SIRT2](http://www.ncbi.nlm.nih.gov/gene/?term=22933)(22.373) [SPI1](http://www.ncbi.nlm.nih.gov/gene/?term=6688)(22.373)[HOMER1](http://www.ncbi.nlm.nih.gov/gene/?term=9456)(22.373)[SHANK3](http://www.ncbi.nlm.nih.gov/gene/?term=85358)(22.373)[TAS1R1](http://www.ncbi.nlm.nih.gov/gene/?term=80835)(22.373)[TRIM28](http://www.ncbi.nlm.nih.gov/gene/?term=10155)(22.373) [XRCC4](http://www.ncbi.nlm.nih.gov/gene/?term=7518)(22.373)[MED1](http://www.ncbi.nlm.nih.gov/gene/?term=5469)(22.373)[NKX3-1](http://www.ncbi.nlm.nih.gov/gene/?term=4824)(22.373)[UBE2B](http://www.ncbi.nlm.nih.gov/gene/?term=7320)(22.373)[ESR2](http://www.ncbi.nlm.nih.gov/gene/?term=2100)(22.373) [HCN4](http://www.ncbi.nlm.nih.gov/gene/?term=10021)(22.373)[EDN1](http://www.ncbi.nlm.nih.gov/gene/?term=1906)(22.373)[CACNA1A](http://www.ncbi.nlm.nih.gov/gene/?term=773)(22.373)[STAT5A](http://www.ncbi.nlm.nih.gov/gene/?term=6776)(22.373)[TP53](http://www.ncbi.nlm.nih.gov/gene/?term=7157)(22.373) [GNAT3](http://www.ncbi.nlm.nih.gov/gene/?term=346562)(22.373)[HELB](http://www.ncbi.nlm.nih.gov/gene/?term=92797)(22.373)[COMT](http://www.ncbi.nlm.nih.gov/gene/?term=1312)(22.373)[SLC26A6](http://www.ncbi.nlm.nih.gov/gene/?term=65010)(22.373)[ADRA2A](http://www.ncbi.nlm.nih.gov/gene/?term=150)(22.373) [YWHAE](http://www.ncbi.nlm.nih.gov/gene/?term=7531)(22.373)[CETN2](http://www.ncbi.nlm.nih.gov/gene/?term=1069)(22.373)[POLA1](http://www.ncbi.nlm.nih.gov/gene/?term=5422)(22.373)[CRP](http://www.ncbi.nlm.nih.gov/gene/?term=1401)(22.373)[CETN1](http://www.ncbi.nlm.nih.gov/gene/?term=1068)(22.373) [NUDT9](http://www.ncbi.nlm.nih.gov/gene/?term=53343)(22.373)[PIK3CG](http://www.ncbi.nlm.nih.gov/gene/?term=5294)(22.373)[BMP2](http://www.ncbi.nlm.nih.gov/gene/?term=650)(22.373)[TNF](http://www.ncbi.nlm.nih.gov/gene/?term=7124)(22.373)[NEDD4](http://www.ncbi.nlm.nih.gov/gene/?term=4734)(22.373) [AGT](http://www.ncbi.nlm.nih.gov/gene/?term=183)(22.373)[FER](http://www.ncbi.nlm.nih.gov/gene/?term=2241)(22.373)[SHH](http://www.ncbi.nlm.nih.gov/gene/?term=6469)(22.373)[SERPINB3](http://www.ncbi.nlm.nih.gov/gene/?term=6317)(22.373)[FOXP3](http://www.ncbi.nlm.nih.gov/gene/?term=50943)(22.373) [CXCL13](http://www.ncbi.nlm.nih.gov/gene/?term=10563)(22.373)[ARID1A](http://www.ncbi.nlm.nih.gov/gene/?term=8289)(22.373)[FGF10](http://www.ncbi.nlm.nih.gov/gene/?term=2255)(22.373)[KCNB1](http://www.ncbi.nlm.nih.gov/gene/?term=3745)(22.373)[LEF1](http://www.ncbi.nlm.nih.gov/gene/?term=51176)(22.373) [PTK2B](http://www.ncbi.nlm.nih.gov/gene/?term=2185)(22.373)[LIG4](http://www.ncbi.nlm.nih.gov/gene/?term=3981)(22.373)[ADRBK1](http://www.ncbi.nlm.nih.gov/gene/?term=156)(22.373)[MAOB](http://www.ncbi.nlm.nih.gov/gene/?term=4129)(22.373)[SORCS3](http://www.ncbi.nlm.nih.gov/gene/?term=22986)(22.373) [ADRB1](http://www.ncbi.nlm.nih.gov/gene/?term=153)(22.373)[ADAM8](http://www.ncbi.nlm.nih.gov/gene/?term=101)(22.373)[VTI1A](http://www.ncbi.nlm.nih.gov/gene/?term=143187)(22.373)[SLC44A4](http://www.ncbi.nlm.nih.gov/gene/?term=80736)(22.373)[ADAP2](http://www.ncbi.nlm.nih.gov/gene/?term=55803)(22.373) [LRRC8A](http://www.ncbi.nlm.nih.gov/gene/?term=56262)(22.373)[FGFR2](http://www.ncbi.nlm.nih.gov/gene/?term=2263)(22.373)[AURKA](http://www.ncbi.nlm.nih.gov/gene/?term=6790)(22.373)[MCM3](http://www.ncbi.nlm.nih.gov/gene/?term=4172)(22.373)[VCP](http://www.ncbi.nlm.nih.gov/gene/?term=7415)(22.373) [GHRL](http://www.ncbi.nlm.nih.gov/gene/?term=51738)(22.373)[BCL11B](http://www.ncbi.nlm.nih.gov/gene/?term=64919)(22.373)[BCL2](http://www.ncbi.nlm.nih.gov/gene/?term=596)(22.373)[8-Mar](http://www.ncbi.nlm.nih.gov/gene/?term=220972)(22.373)[GAS6](http://www.ncbi.nlm.nih.gov/gene/?term=2621)(22.373) [C5](http://www.ncbi.nlm.nih.gov/gene/?term=727)(22.373)[SREBF1](http://www.ncbi.nlm.nih.gov/gene/?term=6720)(22.373)[DRD2](http://www.ncbi.nlm.nih.gov/gene/?term=1813)(22.373)[PLN](http://www.ncbi.nlm.nih.gov/gene/?term=5350)(22.373)[DNM3](http://www.ncbi.nlm.nih.gov/gene/?term=26052)(22.373) [CHRNA3](http://www.ncbi.nlm.nih.gov/gene/?term=1136)(22.373)[TCF3](http://www.ncbi.nlm.nih.gov/gene/?term=6929)(22.373)[SRC](http://www.ncbi.nlm.nih.gov/gene/?term=6714)(22.373)[LONP1](http://www.ncbi.nlm.nih.gov/gene/?term=9361)(22.373)[XCL1](http://www.ncbi.nlm.nih.gov/gene/?term=6375)(22.373) [HSP90AB1](http://www.ncbi.nlm.nih.gov/gene/?term=3326)(22.373)[HIBADH](http://www.ncbi.nlm.nih.gov/gene/?term=11112)(22.373)[STAP1](http://www.ncbi.nlm.nih.gov/gene/?term=26228)(22.373)[HRC](http://www.ncbi.nlm.nih.gov/gene/?term=3270)(22.373)[UTS2R](http://www.ncbi.nlm.nih.gov/gene/?term=2837)(22.373) [SLC6A4](http://www.ncbi.nlm.nih.gov/gene/?term=6532)(22.373)[HMGCR](http://www.ncbi.nlm.nih.gov/gene/?term=3156)(22.373)[HIPK2](http://www.ncbi.nlm.nih.gov/gene/?term=28996)(22.373)[PPP3CA](http://www.ncbi.nlm.nih.gov/gene/?term=5530)(22.373)[RAPGEF2](http://www.ncbi.nlm.nih.gov/gene/?term=9693)(22.373) [TET1](http://www.ncbi.nlm.nih.gov/gene/?term=80312)(22.373)[C2CD5](http://www.ncbi.nlm.nih.gov/gene/?term=9847)(22.373)[CSF2](http://www.ncbi.nlm.nih.gov/gene/?term=1437)(22.373)[TALDO1](http://www.ncbi.nlm.nih.gov/gene/?term=6888)(22.373)[BDKRB2](http://www.ncbi.nlm.nih.gov/gene/?term=624)(22.373) [KCNH2](http://www.ncbi.nlm.nih.gov/gene/?term=3757)(22.373)[MMP28](http://www.ncbi.nlm.nih.gov/gene/?term=79148)(22.373)[HIF1A](http://www.ncbi.nlm.nih.gov/gene/?term=3091)(22.373)[KIT](http://www.ncbi.nlm.nih.gov/gene/?term=3815)(22.373)[INS](http://www.ncbi.nlm.nih.gov/gene/?term=3630)(22.373) [MTOR](http://www.ncbi.nlm.nih.gov/gene/?term=2475)(22.373)[WNT10B](http://www.ncbi.nlm.nih.gov/gene/?term=7480)(22.373)[SLC18A3](http://www.ncbi.nlm.nih.gov/gene/?term=6572)(22.373)[ABCC4](http://www.ncbi.nlm.nih.gov/gene/?term=10257)(22.373)[BNIP3](http://www.ncbi.nlm.nih.gov/gene/?term=664)(22.373) [ZFPM1](http://www.ncbi.nlm.nih.gov/gene/?term=161882)(22.373)[GATA3](http://www.ncbi.nlm.nih.gov/gene/?term=2625)(22.373)[UBR5](http://www.ncbi.nlm.nih.gov/gene/?term=51366)(22.373)[AREG](http://www.ncbi.nlm.nih.gov/gene/?term=374)(22.373)[CRH](http://www.ncbi.nlm.nih.gov/gene/?term=1392)(22.373) [ZFP42](http://www.ncbi.nlm.nih.gov/gene/?term=132625)(22.373)[PIK3R6](http://www.ncbi.nlm.nih.gov/gene/?term=146850)(22.373)[PF4](http://www.ncbi.nlm.nih.gov/gene/?term=5196)(22.373)[ATP1A2](http://www.ncbi.nlm.nih.gov/gene/?term=477)(22.373)[AVPR2](http://www.ncbi.nlm.nih.gov/gene/?term=554)(22.373) [CAMK2D](http://www.ncbi.nlm.nih.gov/gene/?term=817)(22.373)[ADRA1B](http://www.ncbi.nlm.nih.gov/gene/?term=147)(22.373)[IL10](http://www.ncbi.nlm.nih.gov/gene/?term=3586)(22.373)[NEFL](http://www.ncbi.nlm.nih.gov/gene/?term=4747)(22.373)[UCN2](http://www.ncbi.nlm.nih.gov/gene/?term=90226)(22.373) [ATP1A1](http://www.ncbi.nlm.nih.gov/gene/?term=476)(22.373)[RYR3](http://www.ncbi.nlm.nih.gov/gene/?term=6263)(22.373)[PPP3R1](http://www.ncbi.nlm.nih.gov/gene/?term=5534)(22.373)[SPX](http://www.ncbi.nlm.nih.gov/gene/?term=80763)(22.373)[ATP2A1](http://www.ncbi.nlm.nih.gov/gene/?term=487)(22.373) [SMO](http://www.ncbi.nlm.nih.gov/gene/?term=6608)(22.373)[RAG1](http://www.ncbi.nlm.nih.gov/gene/?term=5896)(22.373)[RAB8B](http://www.ncbi.nlm.nih.gov/gene/?term=51762)(22.373)[DKK3](http://www.ncbi.nlm.nih.gov/gene/?term=27122)(22.373)[AHCYL1](http://www.ncbi.nlm.nih.gov/gene/?term=10768)(22.373) [AQP1](http://www.ncbi.nlm.nih.gov/gene/?term=358)(22.373)[TNFAIP3](http://www.ncbi.nlm.nih.gov/gene/?term=7128)(22.373)[TAC4](http://www.ncbi.nlm.nih.gov/gene/?term=255061)(22.373)[DPPA3](http://www.ncbi.nlm.nih.gov/gene/?term=359787)(22.373)[CASQ1](http://www.ncbi.nlm.nih.gov/gene/?term=844)(22.373) [FKBP1B](http://www.ncbi.nlm.nih.gov/gene/?term=2281)(22.373)[CDC42](http://www.ncbi.nlm.nih.gov/gene/?term=998)(22.373)[IL4](http://www.ncbi.nlm.nih.gov/gene/?term=3565)(22.373)[POLB](http://www.ncbi.nlm.nih.gov/gene/?term=5423)(22.373)[IDO1](http://www.ncbi.nlm.nih.gov/gene/?term=3620)(22.373) [ATP2B4](http://www.ncbi.nlm.nih.gov/gene/?term=493)(22.373)[CTNNB1](http://www.ncbi.nlm.nih.gov/gene/?term=1499)(22.373)[PGLS](http://www.ncbi.nlm.nih.gov/gene/?term=25796)(22.373)[SLC9A3R1](http://www.ncbi.nlm.nih.gov/gene/?term=9368)(22.373)[IGF2](http://www.ncbi.nlm.nih.gov/gene/?term=3481)(22.373) [HPS4](http://www.ncbi.nlm.nih.gov/gene/?term=89781)(22.373)[TKT](http://www.ncbi.nlm.nih.gov/gene/?term=7086)(22.373)[DRD1](http://www.ncbi.nlm.nih.gov/gene/?term=1812)(22.373)[ADAP1](http://www.ncbi.nlm.nih.gov/gene/?term=11033)(22.373)[EDA](http://www.ncbi.nlm.nih.gov/gene/?term=1896)(22.373) [RYR2](http://www.ncbi.nlm.nih.gov/gene/?term=6262)(22.373)[CD34](http://www.ncbi.nlm.nih.gov/gene/?term=947)(22.373)[HSP90AA1](http://www.ncbi.nlm.nih.gov/gene/?term=3320)(22.373)[LTA](http://www.ncbi.nlm.nih.gov/gene/?term=4049)(22.373)[SPR](http://www.ncbi.nlm.nih.gov/gene/?term=6697)(22.373) [GPER1](http://www.ncbi.nlm.nih.gov/gene/?term=2852)(22.373)[CBFA2T3](http://www.ncbi.nlm.nih.gov/gene/?term=863)(22.373)[CALHM1](http://www.ncbi.nlm.nih.gov/gene/?term=255022)(22.373)[WNT5A](http://www.ncbi.nlm.nih.gov/gene/?term=7474)(22.373)[SELP](http://www.ncbi.nlm.nih.gov/gene/?term=6403)(22.373) [XRCC6BP1](http://www.ncbi.nlm.nih.gov/gene/?term=91419)(22.373)[PARP10](http://www.ncbi.nlm.nih.gov/gene/?term=84875)(22.373)[HPRT1](http://www.ncbi.nlm.nih.gov/gene/?term=3251)(22.373)[AMICA1](http://www.ncbi.nlm.nih.gov/gene/?term=120425)(22.373)[TAS1R3](http://www.ncbi.nlm.nih.gov/gene/?term=83756)(22.373) [NOS1](http://www.ncbi.nlm.nih.gov/gene/?term=4842)(22.373)[TREM1](http://www.ncbi.nlm.nih.gov/gene/?term=54210)(22.373)[TAL1](http://www.ncbi.nlm.nih.gov/gene/?term=6886)(22.373)[ALAD](http://www.ncbi.nlm.nih.gov/gene/?term=210)(22.373)[PAWR](http://www.ncbi.nlm.nih.gov/gene/?term=5074)(22.373) [SOX9](http://www.ncbi.nlm.nih.gov/gene/?term=6662)(22.373)[BMP5](http://www.ncbi.nlm.nih.gov/gene/?term=653)(22.373)[PDGFB](http://www.ncbi.nlm.nih.gov/gene/?term=5155)(22.373)[RTN2](http://www.ncbi.nlm.nih.gov/gene/?term=6253)(22.373)[CD63](http://www.ncbi.nlm.nih.gov/gene/?term=967)(22.373) [CAMK2G](http://www.ncbi.nlm.nih.gov/gene/?term=818)(22.373)[LAMP2](http://www.ncbi.nlm.nih.gov/gene/?term=3920)(22.373)[NEFH](http://www.ncbi.nlm.nih.gov/gene/?term=4744)(22.373)[GLYR1](http://www.ncbi.nlm.nih.gov/gene/?term=84656)(22.373)[VDR](http://www.ncbi.nlm.nih.gov/gene/?term=7421)(22.373) [GNAS](http://www.ncbi.nlm.nih.gov/gene/?term=2778)(22.373)[NCBP2](http://www.ncbi.nlm.nih.gov/gene/?term=22916)(22.373)[TPM1](http://www.ncbi.nlm.nih.gov/gene/?term=7168)(22.373)[PIK3R5](http://www.ncbi.nlm.nih.gov/gene/?term=23533)(22.373)[PCSK6](http://www.ncbi.nlm.nih.gov/gene/?term=5046)(22.373) [TRIM24](http://www.ncbi.nlm.nih.gov/gene/?term=8805)(22.373)[REST](http://www.ncbi.nlm.nih.gov/gene/?term=5978)(22.373)[CRHR2](http://www.ncbi.nlm.nih.gov/gene/?term=1395)(22.373)[HCN2](http://www.ncbi.nlm.nih.gov/gene/?term=610)(22.373)[RAG2](http://www.ncbi.nlm.nih.gov/gene/?term=5897)(22.373) [NFX1](http://www.ncbi.nlm.nih.gov/gene/?term=4799)(22.373)[TAS1R2](http://www.ncbi.nlm.nih.gov/gene/?term=80834)(22.373) | |
| [Ribose](http://www.megabionet.org/tcmid/ingredient/18822/) | This compound doesn't have any potential target with score larger than 20. | |
| [Suffruticoside A](http://www.megabionet.org/tcmid/ingredient/20435/) | This compound doesn't have any potential target with score larger than 20. | |
| [Betaine](http://www.megabionet.org/tcmid/ingredient/23051/) | [PPAT](http://www.ncbi.nlm.nih.gov/gene/?term=5471)(183.000)[NPY2R](http://www.ncbi.nlm.nih.gov/gene/?term=4887)(183.000)[CTPS1](http://www.ncbi.nlm.nih.gov/gene/?term=1503)(183.000)[CPS1](http://www.ncbi.nlm.nih.gov/gene/?term=1373)(183.000)[SST](http://www.ncbi.nlm.nih.gov/gene/?term=6750)(183.000) [GLUL](http://www.ncbi.nlm.nih.gov/gene/?term=2752)(183.000)[GOT2](http://www.ncbi.nlm.nih.gov/gene/?term=2806)(122.778)[SLC1A1](http://www.ncbi.nlm.nih.gov/gene/?term=6505)(122.778)[ACY3](http://www.ncbi.nlm.nih.gov/gene/?term=91703)(122.778)[ASPA](http://www.ncbi.nlm.nih.gov/gene/?term=443)(122.778) [ADSSL1](http://www.ncbi.nlm.nih.gov/gene/?term=122622)(122.778)[ASNS](http://www.ncbi.nlm.nih.gov/gene/?term=440)(122.778)[DARS2](http://www.ncbi.nlm.nih.gov/gene/?term=55157)(122.778)[GOT1](http://www.ncbi.nlm.nih.gov/gene/?term=2805)(122.778)[ASPH](http://www.ncbi.nlm.nih.gov/gene/?term=444)(122.778) [SLC25A12](http://www.ncbi.nlm.nih.gov/gene/?term=8604)(122.778)[DARS](http://www.ncbi.nlm.nih.gov/gene/?term=1615)(122.778)[ASRGL1](http://www.ncbi.nlm.nih.gov/gene/?term=80150)(122.778)[RNASE1](http://www.ncbi.nlm.nih.gov/gene/?term=6035)(122.778)[ASS1](http://www.ncbi.nlm.nih.gov/gene/?term=445)(122.778) [ADSS](http://www.ncbi.nlm.nih.gov/gene/?term=159)(122.778)[CAD](http://www.ncbi.nlm.nih.gov/gene/?term=790)(122.778)[ACY1](http://www.ncbi.nlm.nih.gov/gene/?term=95)(122.778)[SLC25A13](http://www.ncbi.nlm.nih.gov/gene/?term=10165)(122.778)[LYZ](http://www.ncbi.nlm.nih.gov/gene/?term=4069)(122.778) [PAICS](http://www.ncbi.nlm.nih.gov/gene/?term=10606)(122.778)[BCAT1](http://www.ncbi.nlm.nih.gov/gene/?term=586)(48.000)[VARS](http://www.ncbi.nlm.nih.gov/gene/?term=7407)(48.000)[TARS](http://www.ncbi.nlm.nih.gov/gene/?term=6897)(48.000)[THNSL1](http://www.ncbi.nlm.nih.gov/gene/?term=79896)(48.000) [TARS2](http://www.ncbi.nlm.nih.gov/gene/?term=80222)(48.000)[PCCB](http://www.ncbi.nlm.nih.gov/gene/?term=5096)(48.000)[SLC5A6](http://www.ncbi.nlm.nih.gov/gene/?term=8884)(36.688)[UGCG](http://www.ncbi.nlm.nih.gov/gene/?term=7357)(36.688)[LIAS](http://www.ncbi.nlm.nih.gov/gene/?term=11019)(36.688) [TH](http://www.ncbi.nlm.nih.gov/gene/?term=7054)(36.688)[LIPT1](http://www.ncbi.nlm.nih.gov/gene/?term=51601)(36.688)[HPD](http://www.ncbi.nlm.nih.gov/gene/?term=3242)(36.688)[TPH1](http://www.ncbi.nlm.nih.gov/gene/?term=7166)(36.688)[PAH](http://www.ncbi.nlm.nih.gov/gene/?term=5053)(36.688) [NOS3](http://www.ncbi.nlm.nih.gov/gene/?term=4846)(36.688) | |
| [Gancaonin A](http://www.megabionet.org/tcmid/ingredient/8133/) | This compound doesn't have any potential target with score larger than 20. | |
| [Maltose](http://www.megabionet.org/tcmid/ingredient/13447/) | [CXCR4](http://www.ncbi.nlm.nih.gov/gene/?term=7852)(48.000) | |
| [Borneol](http://www.megabionet.org/tcmid/ingredient/2550/) | [TRPV3](http://www.ncbi.nlm.nih.gov/gene/?term=162514)(122.778)[OPRK1](http://www.ncbi.nlm.nih.gov/gene/?term=4986)(122.778)[TRPM8](http://www.ncbi.nlm.nih.gov/gene/?term=79054)(122.778)[TRPA1](http://www.ncbi.nlm.nih.gov/gene/?term=8989)(122.778)[KCNK4](http://www.ncbi.nlm.nih.gov/gene/?term=50801)(55.444) [ESRRG](http://www.ncbi.nlm.nih.gov/gene/?term=2104)(48.000)[COX6C](http://www.ncbi.nlm.nih.gov/gene/?term=1345)(48.000)[COX5B](http://www.ncbi.nlm.nih.gov/gene/?term=1329)(48.000)[COX7C](http://www.ncbi.nlm.nih.gov/gene/?term=1350)(48.000)[COX1](http://www.ncbi.nlm.nih.gov/gene/?term=4512)(48.000) [AKR1C2](http://www.ncbi.nlm.nih.gov/gene/?term=1646)(48.000)[COX5A](http://www.ncbi.nlm.nih.gov/gene/?term=9377)(48.000)[COX3](http://www.ncbi.nlm.nih.gov/gene/?term=4514)(48.000)[AR](http://www.ncbi.nlm.nih.gov/gene/?term=367)(48.000)[COX7A1](http://www.ncbi.nlm.nih.gov/gene/?term=1346)(48.000) [FECH](http://www.ncbi.nlm.nih.gov/gene/?term=2235)(48.000)[COX4I1](http://www.ncbi.nlm.nih.gov/gene/?term=1327)(48.000)[PLA2G1B](http://www.ncbi.nlm.nih.gov/gene/?term=5319)(48.000)[COX6A2](http://www.ncbi.nlm.nih.gov/gene/?term=1339)(48.000)[ADH1C](http://www.ncbi.nlm.nih.gov/gene/?term=126)(48.000) [COX6B1](http://www.ncbi.nlm.nih.gov/gene/?term=1340)(48.000)[FABP6](http://www.ncbi.nlm.nih.gov/gene/?term=2172)(48.000)[CES1](http://www.ncbi.nlm.nih.gov/gene/?term=1066)(48.000)[COX7B](http://www.ncbi.nlm.nih.gov/gene/?term=1349)(48.000)[COX2](http://www.ncbi.nlm.nih.gov/gene/?term=4513)(48.000) [COX8A](http://www.ncbi.nlm.nih.gov/gene/?term=1351)(48.000)[NR1H4](http://www.ncbi.nlm.nih.gov/gene/?term=9971)(48.000)[ACTN3](http://www.ncbi.nlm.nih.gov/gene/?term=89)(22.373)[TAC1](http://www.ncbi.nlm.nih.gov/gene/?term=6863)(22.373)[NGFR](http://www.ncbi.nlm.nih.gov/gene/?term=4804)(22.373) [ADRA2A](http://www.ncbi.nlm.nih.gov/gene/?term=150)(22.373)[TRPV1](http://www.ncbi.nlm.nih.gov/gene/?term=7442)(22.373)[FGF10](http://www.ncbi.nlm.nih.gov/gene/?term=2255)(22.373)[VCP](http://www.ncbi.nlm.nih.gov/gene/?term=7415)(22.373)[HIF1A](http://www.ncbi.nlm.nih.gov/gene/?term=3091)(22.373) [BNIP3](http://www.ncbi.nlm.nih.gov/gene/?term=664)(22.373)[CRH](http://www.ncbi.nlm.nih.gov/gene/?term=1392)(22.373)[NEFL](http://www.ncbi.nlm.nih.gov/gene/?term=4747)(22.373)[UCN2](http://www.ncbi.nlm.nih.gov/gene/?term=90226)(22.373)[SMO](http://www.ncbi.nlm.nih.gov/gene/?term=6608)(22.373) [TACR2](http://www.ncbi.nlm.nih.gov/gene/?term=6865)(22.373)[AQP1](http://www.ncbi.nlm.nih.gov/gene/?term=358)(22.373)[TAC4](http://www.ncbi.nlm.nih.gov/gene/?term=255061)(22.373)[CDH3](http://www.ncbi.nlm.nih.gov/gene/?term=1001)(22.373)[CBFA2T3](http://www.ncbi.nlm.nih.gov/gene/?term=863)(22.373) [NEFH](http://www.ncbi.nlm.nih.gov/gene/?term=4744)(22.373)[CRHR2](http://www.ncbi.nlm.nih.gov/gene/?term=1395)(22.373) | |
| [Kaempferol](http://www.megabionet.org/tcmid/ingredient/12017/) | [HSD17B1](http://www.ncbi.nlm.nih.gov/gene/?term=3292)(26.373)[CRYZ](http://www.ncbi.nlm.nih.gov/gene/?term=1429)(23.000)[VKORC1](http://www.ncbi.nlm.nih.gov/gene/?term=79001)(23.000)[NQO1](http://www.ncbi.nlm.nih.gov/gene/?term=1728)(23.000) | |
| [Eicosane](http://www.megabionet.org/tcmid/ingredient/6719/) | This compound doesn't have any potential target with score larger than 20. | |
| [4-Methylsulfinyl Butyl Isothiocyanate](http://www.megabionet.org/tcmid/ingredient/14720/) | This compound doesn't have any potential target with score larger than 20. | |
| [Carthamidin](http://www.megabionet.org/tcmid/ingredient/3223/) | [SOAT1](http://www.ncbi.nlm.nih.gov/gene/?term=6646)(122.778)[MTTP](http://www.ncbi.nlm.nih.gov/gene/?term=4547)(122.778)[SOAT2](http://www.ncbi.nlm.nih.gov/gene/?term=8435)(122.778)[CNR2](http://www.ncbi.nlm.nih.gov/gene/?term=1269)(48.000)[CNR1](http://www.ncbi.nlm.nih.gov/gene/?term=1268)(48.000) [DRD2](http://www.ncbi.nlm.nih.gov/gene/?term=1813)(22.373)[PAWR](http://www.ncbi.nlm.nih.gov/gene/?term=5074)(22.373) | |
| [Coelogin](http://www.megabionet.org/tcmid/ingredient/3889/) | [SEC14L3](http://www.ncbi.nlm.nih.gov/gene/?term=266629)(80.882)[PPP2CA](http://www.ncbi.nlm.nih.gov/gene/?term=5515)(80.882)[PRKCA](http://www.ncbi.nlm.nih.gov/gene/?term=5578)(80.882)[NR1I2](http://www.ncbi.nlm.nih.gov/gene/?term=8856)(80.882)[ALOX5](http://www.ncbi.nlm.nih.gov/gene/?term=240)(80.882) [PPP2CB](http://www.ncbi.nlm.nih.gov/gene/?term=5516)(80.882)[SEC14L2](http://www.ncbi.nlm.nih.gov/gene/?term=23541)(80.882)[DGKA](http://www.ncbi.nlm.nih.gov/gene/?term=1606)(80.882)[PRKCB](http://www.ncbi.nlm.nih.gov/gene/?term=5579)(80.882)[SEC14L4](http://www.ncbi.nlm.nih.gov/gene/?term=284904)(80.882) [SERPINE1](http://www.ncbi.nlm.nih.gov/gene/?term=5054)(55.444)[CHGA](http://www.ncbi.nlm.nih.gov/gene/?term=1113)(55.444)[SEC14L6](http://www.ncbi.nlm.nih.gov/gene/?term=730005)(23.000)[CNR2](http://www.ncbi.nlm.nih.gov/gene/?term=1269)(23.000)[TYR](http://www.ncbi.nlm.nih.gov/gene/?term=7299)(23.000) [CNR1](http://www.ncbi.nlm.nih.gov/gene/?term=1268)(23.000)[SLC2A1](http://www.ncbi.nlm.nih.gov/gene/?term=6513)(22.373)[JUP](http://www.ncbi.nlm.nih.gov/gene/?term=3728)(22.373)[FGF1](http://www.ncbi.nlm.nih.gov/gene/?term=2246)(22.373)[PKP2](http://www.ncbi.nlm.nih.gov/gene/?term=5318)(22.373) [SERPINE2](http://www.ncbi.nlm.nih.gov/gene/?term=5270)(22.373)[POR](http://www.ncbi.nlm.nih.gov/gene/?term=5447)(22.373)[CCL3](http://www.ncbi.nlm.nih.gov/gene/?term=6348)(22.373)[ABCG2](http://www.ncbi.nlm.nih.gov/gene/?term=9429)(22.373)[SOCS1](http://www.ncbi.nlm.nih.gov/gene/?term=8651)(22.373) [SREBF1](http://www.ncbi.nlm.nih.gov/gene/?term=6720)(22.373)[PTPN2](http://www.ncbi.nlm.nih.gov/gene/?term=5771)(22.373)[TTPA](http://www.ncbi.nlm.nih.gov/gene/?term=7274)(22.373)[PRKAA1](http://www.ncbi.nlm.nih.gov/gene/?term=5562)(22.373)[PPP2R1A](http://www.ncbi.nlm.nih.gov/gene/?term=5518)(22.373) [ABCB4](http://www.ncbi.nlm.nih.gov/gene/?term=5244)(22.373)[PKP3](http://www.ncbi.nlm.nih.gov/gene/?term=11187)(22.373)[PRKCG](http://www.ncbi.nlm.nih.gov/gene/?term=5582)(22.373)[NF2](http://www.ncbi.nlm.nih.gov/gene/?term=4771)(22.373)[ABCB1](http://www.ncbi.nlm.nih.gov/gene/?term=5243)(22.373) [AFM](http://www.ncbi.nlm.nih.gov/gene/?term=173)(22.373)[ABCG1](http://www.ncbi.nlm.nih.gov/gene/?term=9619)(22.373)[REST](http://www.ncbi.nlm.nih.gov/gene/?term=5978)(22.373) | |
| [3-Methyl-3-Hexanol](http://www.megabionet.org/tcmid/ingredient/23521/) | [TRPV3](http://www.ncbi.nlm.nih.gov/gene/?term=162514)(48.000)[OPRK1](http://www.ncbi.nlm.nih.gov/gene/?term=4986)(48.000)[TRPM8](http://www.ncbi.nlm.nih.gov/gene/?term=79054)(48.000)[TRPA1](http://www.ncbi.nlm.nih.gov/gene/?term=8989)(48.000) | |
| [Glycyrin](http://www.megabionet.org/tcmid/ingredient/8837/) | [CNR2](http://www.ncbi.nlm.nih.gov/gene/?term=1269)(23.000)[CNR1](http://www.ncbi.nlm.nih.gov/gene/?term=1268)(23.000)[DRD2](http://www.ncbi.nlm.nih.gov/gene/?term=1813)(22.373) | |
| [Phenylic Acid](http://www.megabionet.org/tcmid/ingredient/24994/) | [PROS1](http://www.ncbi.nlm.nih.gov/gene/?term=5627)(183.000)[PROC](http://www.ncbi.nlm.nih.gov/gene/?term=5624)(183.000)[GLUD2](http://www.ncbi.nlm.nih.gov/gene/?term=2747)(183.000)[ESR1](http://www.ncbi.nlm.nih.gov/gene/?term=2099)(114.143)[F12](http://www.ncbi.nlm.nih.gov/gene/?term=2161)(112.667) [GLUD1](http://www.ncbi.nlm.nih.gov/gene/?term=2746)(112.667)[SCN10A](http://www.ncbi.nlm.nih.gov/gene/?term=6336)(112.667)[SDHD](http://www.ncbi.nlm.nih.gov/gene/?term=6392)(112.667)[GABRA2](http://www.ncbi.nlm.nih.gov/gene/?term=2555)(80.882)[GABRD](http://www.ncbi.nlm.nih.gov/gene/?term=2563)(80.882) [PDE3A](http://www.ncbi.nlm.nih.gov/gene/?term=5139)(80.882)[GABRB1](http://www.ncbi.nlm.nih.gov/gene/?term=2560)(80.882)[GABRG3](http://www.ncbi.nlm.nih.gov/gene/?term=2567)(80.882)[TNF](http://www.ncbi.nlm.nih.gov/gene/?term=7124)(80.882)[GABRE](http://www.ncbi.nlm.nih.gov/gene/?term=2564)(80.882) [SCN2A](http://www.ncbi.nlm.nih.gov/gene/?term=6326)(80.882)[GABRA3](http://www.ncbi.nlm.nih.gov/gene/?term=2556)(80.882)[GABRG1](http://www.ncbi.nlm.nih.gov/gene/?term=2565)(80.882)[GABRP](http://www.ncbi.nlm.nih.gov/gene/?term=2568)(80.882)[GABRA4](http://www.ncbi.nlm.nih.gov/gene/?term=2557)(80.882) [GABRB2](http://www.ncbi.nlm.nih.gov/gene/?term=2561)(80.882)[ACHE](http://www.ncbi.nlm.nih.gov/gene/?term=43)(80.882)[SCN4A](http://www.ncbi.nlm.nih.gov/gene/?term=6329)(80.882)[GABRQ](http://www.ncbi.nlm.nih.gov/gene/?term=55879)(80.882)[GABRB3](http://www.ncbi.nlm.nih.gov/gene/?term=2562)(80.882) [GABRA5](http://www.ncbi.nlm.nih.gov/gene/?term=2558)(80.882)[GABRA6](http://www.ncbi.nlm.nih.gov/gene/?term=2559)(80.882)[GABRA1](http://www.ncbi.nlm.nih.gov/gene/?term=2554)(80.882)[PDE4B](http://www.ncbi.nlm.nih.gov/gene/?term=5142)(80.882)[BCHE](http://www.ncbi.nlm.nih.gov/gene/?term=590)(80.882) [GABRG2](http://www.ncbi.nlm.nih.gov/gene/?term=2566)(80.882)[PDE3B](http://www.ncbi.nlm.nih.gov/gene/?term=5140)(55.444)[PDE4D](http://www.ncbi.nlm.nih.gov/gene/?term=5144)(55.444)[COLQ](http://www.ncbi.nlm.nih.gov/gene/?term=8292)(55.444)[LTA](http://www.ncbi.nlm.nih.gov/gene/?term=4049)(55.444) [RXRA](http://www.ncbi.nlm.nih.gov/gene/?term=6256)(55.444)[SLC6A3](http://www.ncbi.nlm.nih.gov/gene/?term=6531)(36.688)[SCN11A](http://www.ncbi.nlm.nih.gov/gene/?term=11280)(36.688)[KCNMB1](http://www.ncbi.nlm.nih.gov/gene/?term=3779)(36.688)[KCNN1](http://www.ncbi.nlm.nih.gov/gene/?term=3780)(36.688) [CXCR4](http://www.ncbi.nlm.nih.gov/gene/?term=7852)(36.688)[CHRM1](http://www.ncbi.nlm.nih.gov/gene/?term=1128)(36.688)[KCNN2](http://www.ncbi.nlm.nih.gov/gene/?term=3781)(36.688)[SCN5A](http://www.ncbi.nlm.nih.gov/gene/?term=6331)(36.688)[PTGER1](http://www.ncbi.nlm.nih.gov/gene/?term=5731)(36.688) [MAOB](http://www.ncbi.nlm.nih.gov/gene/?term=4129)(36.688)[NR3C1](http://www.ncbi.nlm.nih.gov/gene/?term=2908)(36.688)[CHRNA10](http://www.ncbi.nlm.nih.gov/gene/?term=57053)(36.688)[MAOA](http://www.ncbi.nlm.nih.gov/gene/?term=4128)(36.688)[SCN9A](http://www.ncbi.nlm.nih.gov/gene/?term=6335)(36.688) [SLC6A4](http://www.ncbi.nlm.nih.gov/gene/?term=6532)(36.688)[CHRNA2](http://www.ncbi.nlm.nih.gov/gene/?term=1135)(36.688)[KCNN4](http://www.ncbi.nlm.nih.gov/gene/?term=3783)(36.688)[GRIN3A](http://www.ncbi.nlm.nih.gov/gene/?term=116443)(36.688)[KCNMB2](http://www.ncbi.nlm.nih.gov/gene/?term=10242)(36.688) [KCNMB4](http://www.ncbi.nlm.nih.gov/gene/?term=27345)(36.688)[HTR3A](http://www.ncbi.nlm.nih.gov/gene/?term=3359)(36.688)[CALM2](http://www.ncbi.nlm.nih.gov/gene/?term=805)(36.688)[KCNMA1](http://www.ncbi.nlm.nih.gov/gene/?term=3778)(36.688)[ANXA1](http://www.ncbi.nlm.nih.gov/gene/?term=301)(36.688) [CALM3](http://www.ncbi.nlm.nih.gov/gene/?term=808)(36.688)[CHRM2](http://www.ncbi.nlm.nih.gov/gene/?term=1129)(36.688)[EGFR](http://www.ncbi.nlm.nih.gov/gene/?term=1956)(36.688)[KCNN3](http://www.ncbi.nlm.nih.gov/gene/?term=3782)(36.688)[KCNMB3](http://www.ncbi.nlm.nih.gov/gene/?term=27094)(36.688) [CALM1](http://www.ncbi.nlm.nih.gov/gene/?term=801)(36.688)[SLC6A2](http://www.ncbi.nlm.nih.gov/gene/?term=6530)(36.688)[CACNB1](http://www.ncbi.nlm.nih.gov/gene/?term=782)(25.857)[GRIA4](http://www.ncbi.nlm.nih.gov/gene/?term=2893)(25.857)[CACNG2](http://www.ncbi.nlm.nih.gov/gene/?term=10369)(25.857) [CHRNA4](http://www.ncbi.nlm.nih.gov/gene/?term=1137)(25.857)[CHRNB3](http://www.ncbi.nlm.nih.gov/gene/?term=1142)(25.857)[GRIA1](http://www.ncbi.nlm.nih.gov/gene/?term=2890)(25.857)[GLRA2](http://www.ncbi.nlm.nih.gov/gene/?term=2742)(25.857)[KCNJ3](http://www.ncbi.nlm.nih.gov/gene/?term=3760)(25.857) [VCAM1](http://www.ncbi.nlm.nih.gov/gene/?term=7412)(25.857)[CHRNA7](http://www.ncbi.nlm.nih.gov/gene/?term=1139)(25.857)[KCNJ6](http://www.ncbi.nlm.nih.gov/gene/?term=3763)(25.857)[SLC29A1](http://www.ncbi.nlm.nih.gov/gene/?term=2030)(25.857)[CACNA1S](http://www.ncbi.nlm.nih.gov/gene/?term=779)(25.857) [GRIA2](http://www.ncbi.nlm.nih.gov/gene/?term=2891)(25.857)[AKR1C1](http://www.ncbi.nlm.nih.gov/gene/?term=1645)(25.857)[CHRNA3](http://www.ncbi.nlm.nih.gov/gene/?term=1136)(25.857)[CACNA1C](http://www.ncbi.nlm.nih.gov/gene/?term=775)(25.857)[CHRNB2](http://www.ncbi.nlm.nih.gov/gene/?term=1141)(25.857) [CACNA1D](http://www.ncbi.nlm.nih.gov/gene/?term=776)(25.857)[HTR3D](http://www.ncbi.nlm.nih.gov/gene/?term=200909)(25.857)[PTGS1](http://www.ncbi.nlm.nih.gov/gene/?term=5742)(25.857)[CHRNA6](http://www.ncbi.nlm.nih.gov/gene/?term=8973)(25.857)[PTGS2](http://www.ncbi.nlm.nih.gov/gene/?term=5743)(25.857) [KCNJ5](http://www.ncbi.nlm.nih.gov/gene/?term=3762)(25.857)[HTR3B](http://www.ncbi.nlm.nih.gov/gene/?term=9177)(25.857)[GRIA3](http://www.ncbi.nlm.nih.gov/gene/?term=2892)(25.857)[SLC29A2](http://www.ncbi.nlm.nih.gov/gene/?term=3177)(25.857)[HTR3E](http://www.ncbi.nlm.nih.gov/gene/?term=285242)(25.857) [CHRNA9](http://www.ncbi.nlm.nih.gov/gene/?term=55584)(25.857)[CHRFAM7A](http://www.ncbi.nlm.nih.gov/gene/?term=89832)(25.857)[CHRNA5](http://www.ncbi.nlm.nih.gov/gene/?term=1138)(25.857)[CHRNB4](http://www.ncbi.nlm.nih.gov/gene/?term=1143)(25.857)[KCNJ9](http://www.ncbi.nlm.nih.gov/gene/?term=3765)(25.857) [HTR3C](http://www.ncbi.nlm.nih.gov/gene/?term=170572)(25.857)[GLRA1](http://www.ncbi.nlm.nih.gov/gene/?term=2741)(25.857)[CACNG1](http://www.ncbi.nlm.nih.gov/gene/?term=786)(25.857)[SCN1A](http://www.ncbi.nlm.nih.gov/gene/?term=6323)(23.000)[SCN3A](http://www.ncbi.nlm.nih.gov/gene/?term=6328)(23.000) [HMGA2](http://www.ncbi.nlm.nih.gov/gene/?term=8091)(22.373)[PDE5A](http://www.ncbi.nlm.nih.gov/gene/?term=8654)(22.373)[APOA2](http://www.ncbi.nlm.nih.gov/gene/?term=336)(22.373)[SLC5A7](http://www.ncbi.nlm.nih.gov/gene/?term=60482)(22.373)[SEMA4D](http://www.ncbi.nlm.nih.gov/gene/?term=10507)(22.373) [NRG1](http://www.ncbi.nlm.nih.gov/gene/?term=3084)(22.373)[CD74](http://www.ncbi.nlm.nih.gov/gene/?term=972)(22.373)[HPX](http://www.ncbi.nlm.nih.gov/gene/?term=3263)(22.373)[SIRT2](http://www.ncbi.nlm.nih.gov/gene/?term=22933)(22.373)[MIF](http://www.ncbi.nlm.nih.gov/gene/?term=4282)(22.373) [DMGDH](http://www.ncbi.nlm.nih.gov/gene/?term=29958)(22.373)[NKX3-1](http://www.ncbi.nlm.nih.gov/gene/?term=4824)(22.373)[SIX3](http://www.ncbi.nlm.nih.gov/gene/?term=6496)(22.373)[HCN4](http://www.ncbi.nlm.nih.gov/gene/?term=10021)(22.373)[FASLG](http://www.ncbi.nlm.nih.gov/gene/?term=356)(22.373) [LGALS3](http://www.ncbi.nlm.nih.gov/gene/?term=3958)(22.373)[KLF4](http://www.ncbi.nlm.nih.gov/gene/?term=9314)(22.373)[LILRB1](http://www.ncbi.nlm.nih.gov/gene/?term=10859)(22.373)[NCKAP1L](http://www.ncbi.nlm.nih.gov/gene/?term=3071)(22.373)[CRP](http://www.ncbi.nlm.nih.gov/gene/?term=1401)(22.373) [TLR3](http://www.ncbi.nlm.nih.gov/gene/?term=7098)(22.373)[EPO](http://www.ncbi.nlm.nih.gov/gene/?term=2056)(22.373)[MAP2K5](http://www.ncbi.nlm.nih.gov/gene/?term=5607)(22.373)[EIF2AK1](http://www.ncbi.nlm.nih.gov/gene/?term=27102)(22.373)[IL1B](http://www.ncbi.nlm.nih.gov/gene/?term=3553)(22.373) [SHH](http://www.ncbi.nlm.nih.gov/gene/?term=6469)(22.373)[RIPK1](http://www.ncbi.nlm.nih.gov/gene/?term=8737)(22.373)[FGF10](http://www.ncbi.nlm.nih.gov/gene/?term=2255)(22.373)[PRSS12](http://www.ncbi.nlm.nih.gov/gene/?term=8492)(22.373)[SLC44A4](http://www.ncbi.nlm.nih.gov/gene/?term=80736)(22.373) [ADORA2B](http://www.ncbi.nlm.nih.gov/gene/?term=136)(22.373)[ALDH7A1](http://www.ncbi.nlm.nih.gov/gene/?term=501)(22.373)[FADD](http://www.ncbi.nlm.nih.gov/gene/?term=8772)(22.373)[FGFR2](http://www.ncbi.nlm.nih.gov/gene/?term=2263)(22.373)[ANGPT1](http://www.ncbi.nlm.nih.gov/gene/?term=284)(22.373) [AURKA](http://www.ncbi.nlm.nih.gov/gene/?term=6790)(22.373)[WNT2](http://www.ncbi.nlm.nih.gov/gene/?term=7472)(22.373)[YBX3](http://www.ncbi.nlm.nih.gov/gene/?term=8531)(22.373)[IL1RN](http://www.ncbi.nlm.nih.gov/gene/?term=3557)(22.373)[PLN](http://www.ncbi.nlm.nih.gov/gene/?term=5350)(22.373) [DNM3](http://www.ncbi.nlm.nih.gov/gene/?term=26052)(22.373)[FCER2](http://www.ncbi.nlm.nih.gov/gene/?term=2208)(22.373)[WNT2B](http://www.ncbi.nlm.nih.gov/gene/?term=7482)(22.373)[IFNG](http://www.ncbi.nlm.nih.gov/gene/?term=3458)(22.373)[HRC](http://www.ncbi.nlm.nih.gov/gene/?term=3270)(22.373) [TGFB2](http://www.ncbi.nlm.nih.gov/gene/?term=7042)(22.373)[RAPGEF2](http://www.ncbi.nlm.nih.gov/gene/?term=9693)(22.373)[TIRAP](http://www.ncbi.nlm.nih.gov/gene/?term=114609)(22.373)[ROCK2](http://www.ncbi.nlm.nih.gov/gene/?term=9475)(22.373)[ADIRF](http://www.ncbi.nlm.nih.gov/gene/?term=10974)(22.373) [BDKRB2](http://www.ncbi.nlm.nih.gov/gene/?term=624)(22.373)[ASCL1](http://www.ncbi.nlm.nih.gov/gene/?term=429)(22.373)[ENPP6](http://www.ncbi.nlm.nih.gov/gene/?term=133121)(22.373)[CAMK2D](http://www.ncbi.nlm.nih.gov/gene/?term=817)(22.373)[IL10](http://www.ncbi.nlm.nih.gov/gene/?term=3586)(22.373) [CDH8](http://www.ncbi.nlm.nih.gov/gene/?term=1006)(22.373)[NCOA1](http://www.ncbi.nlm.nih.gov/gene/?term=8648)(22.373)[IL6](http://www.ncbi.nlm.nih.gov/gene/?term=3569)(22.373)[ROCK1](http://www.ncbi.nlm.nih.gov/gene/?term=6093)(22.373)[FNTA](http://www.ncbi.nlm.nih.gov/gene/?term=2339)(22.373) [HPN](http://www.ncbi.nlm.nih.gov/gene/?term=3249)(22.373)[KMT2A](http://www.ncbi.nlm.nih.gov/gene/?term=4297)(22.373)[NOD2](http://www.ncbi.nlm.nih.gov/gene/?term=64127)(22.373)[BMP4](http://www.ncbi.nlm.nih.gov/gene/?term=652)(22.373)[AHCY](http://www.ncbi.nlm.nih.gov/gene/?term=191)(22.373) [APOA1](http://www.ncbi.nlm.nih.gov/gene/?term=335)(22.373)[ARHGEF2](http://www.ncbi.nlm.nih.gov/gene/?term=9181)(22.373)[IL4](http://www.ncbi.nlm.nih.gov/gene/?term=3565)(22.373)[AGRN](http://www.ncbi.nlm.nih.gov/gene/?term=375790)(22.373)[IKBKB](http://www.ncbi.nlm.nih.gov/gene/?term=3551)(22.373) [IDO1](http://www.ncbi.nlm.nih.gov/gene/?term=3620)(22.373)[CTNNB1](http://www.ncbi.nlm.nih.gov/gene/?term=1499)(22.373)[CHKA](http://www.ncbi.nlm.nih.gov/gene/?term=1119)(22.373)[PDE2A](http://www.ncbi.nlm.nih.gov/gene/?term=5138)(22.373)[CHDH](http://www.ncbi.nlm.nih.gov/gene/?term=55349)(22.373) [SOX9](http://www.ncbi.nlm.nih.gov/gene/?term=6662)(22.373)[CAMK2G](http://www.ncbi.nlm.nih.gov/gene/?term=818)(22.373)[FAS](http://www.ncbi.nlm.nih.gov/gene/?term=355)(22.373)[GRIN1](http://www.ncbi.nlm.nih.gov/gene/?term=2902)(22.373)[HCN2](http://www.ncbi.nlm.nih.gov/gene/?term=610)(22.373) | |
| [Alpha-Pinene](http://www.megabionet.org/tcmid/ingredient/23880/) | [KCND1](http://www.ncbi.nlm.nih.gov/gene/?term=3750)(80.882)[KCNA3](http://www.ncbi.nlm.nih.gov/gene/?term=3738)(80.882)[PRKAB1](http://www.ncbi.nlm.nih.gov/gene/?term=5564)(80.882)[ADH1A](http://www.ncbi.nlm.nih.gov/gene/?term=124)(80.882)[KCNA10](http://www.ncbi.nlm.nih.gov/gene/?term=3744)(80.882) [GAMT](http://www.ncbi.nlm.nih.gov/gene/?term=2593)(80.882)[KCNC3](http://www.ncbi.nlm.nih.gov/gene/?term=3748)(80.882)[KCNA1](http://www.ncbi.nlm.nih.gov/gene/?term=3736)(80.882)[KCNA2](http://www.ncbi.nlm.nih.gov/gene/?term=3737)(80.882)[TPO](http://www.ncbi.nlm.nih.gov/gene/?term=7173)(80.882) [CAT](http://www.ncbi.nlm.nih.gov/gene/?term=847)(80.882)[KCNB1](http://www.ncbi.nlm.nih.gov/gene/?term=3745)(80.882)[ADH1B](http://www.ncbi.nlm.nih.gov/gene/?term=125)(80.882)[DLG4](http://www.ncbi.nlm.nih.gov/gene/?term=1742)(80.882)[KCNC2](http://www.ncbi.nlm.nih.gov/gene/?term=3747)(80.882) [KCNC1](http://www.ncbi.nlm.nih.gov/gene/?term=3746)(80.882)[ADH1C](http://www.ncbi.nlm.nih.gov/gene/?term=126)(80.882)[KCNB2](http://www.ncbi.nlm.nih.gov/gene/?term=9312)(80.882)[KCNA5](http://www.ncbi.nlm.nih.gov/gene/?term=3741)(80.882)[RNASE1](http://www.ncbi.nlm.nih.gov/gene/?term=6035)(80.882) [KCND2](http://www.ncbi.nlm.nih.gov/gene/?term=3751)(80.882)[ALDH2](http://www.ncbi.nlm.nih.gov/gene/?term=217)(80.882)[KCNA4](http://www.ncbi.nlm.nih.gov/gene/?term=3739)(80.882)[KCNA7](http://www.ncbi.nlm.nih.gov/gene/?term=3743)(80.882)[KCNA6](http://www.ncbi.nlm.nih.gov/gene/?term=3742)(80.882) [KCND3](http://www.ncbi.nlm.nih.gov/gene/?term=3752)(80.882)[GUCY1B3](http://www.ncbi.nlm.nih.gov/gene/?term=2983)(55.444)[GATM](http://www.ncbi.nlm.nih.gov/gene/?term=2628)(55.444)[KCNK4](http://www.ncbi.nlm.nih.gov/gene/?term=50801)(55.444)[IYD](http://www.ncbi.nlm.nih.gov/gene/?term=389434)(55.444) [KCNQ1](http://www.ncbi.nlm.nih.gov/gene/?term=3784)(55.444)[RDH11](http://www.ncbi.nlm.nih.gov/gene/?term=51109)(48.000)[RBP3](http://www.ncbi.nlm.nih.gov/gene/?term=5949)(48.000)[RETSAT](http://www.ncbi.nlm.nih.gov/gene/?term=54884)(48.000)[CYP17A1](http://www.ncbi.nlm.nih.gov/gene/?term=1586)(48.000) [RDH13](http://www.ncbi.nlm.nih.gov/gene/?term=112724)(48.000)[DHRS3](http://www.ncbi.nlm.nih.gov/gene/?term=9249)(48.000)[RDH5](http://www.ncbi.nlm.nih.gov/gene/?term=5959)(48.000)[ESR1](http://www.ncbi.nlm.nih.gov/gene/?term=2099)(48.000)[PGR](http://www.ncbi.nlm.nih.gov/gene/?term=5241)(48.000) [ALDH1A3](http://www.ncbi.nlm.nih.gov/gene/?term=220)(48.000)[RDH12](http://www.ncbi.nlm.nih.gov/gene/?term=145226)(48.000)[OPRK1](http://www.ncbi.nlm.nih.gov/gene/?term=4986)(48.000)[DHRS4](http://www.ncbi.nlm.nih.gov/gene/?term=10901)(48.000)[RBP1](http://www.ncbi.nlm.nih.gov/gene/?term=5947)(48.000) [ALDH1A1](http://www.ncbi.nlm.nih.gov/gene/?term=216)(48.000)[RLBP1](http://www.ncbi.nlm.nih.gov/gene/?term=6017)(48.000)[RDH14](http://www.ncbi.nlm.nih.gov/gene/?term=57665)(48.000)[RDH8](http://www.ncbi.nlm.nih.gov/gene/?term=50700)(48.000)[LRAT](http://www.ncbi.nlm.nih.gov/gene/?term=9227)(48.000) [ALDH1A2](http://www.ncbi.nlm.nih.gov/gene/?term=8854)(48.000)[NR3C2](http://www.ncbi.nlm.nih.gov/gene/?term=4306)(48.000)[FBP1](http://www.ncbi.nlm.nih.gov/gene/?term=2203)(22.373)[DGKI](http://www.ncbi.nlm.nih.gov/gene/?term=9162)(22.373)[ADH7](http://www.ncbi.nlm.nih.gov/gene/?term=131)(22.373) [NFIB](http://www.ncbi.nlm.nih.gov/gene/?term=4781)(22.373)[CYGB](http://www.ncbi.nlm.nih.gov/gene/?term=114757)(22.373)[NRXN3](http://www.ncbi.nlm.nih.gov/gene/?term=9369)(22.373)[LRRC4B](http://www.ncbi.nlm.nih.gov/gene/?term=94030)(22.373)[AQP8](http://www.ncbi.nlm.nih.gov/gene/?term=343)(22.373) [ARX](http://www.ncbi.nlm.nih.gov/gene/?term=170302)(22.373)[IGF1](http://www.ncbi.nlm.nih.gov/gene/?term=3479)(22.373)[SHANK3](http://www.ncbi.nlm.nih.gov/gene/?term=85358)(22.373)[ACY3](http://www.ncbi.nlm.nih.gov/gene/?term=91703)(22.373)[ASPA](http://www.ncbi.nlm.nih.gov/gene/?term=443)(22.373) [NRXN1](http://www.ncbi.nlm.nih.gov/gene/?term=9378)(22.373)[EDN1](http://www.ncbi.nlm.nih.gov/gene/?term=1906)(22.373)[OXT](http://www.ncbi.nlm.nih.gov/gene/?term=5020)(22.373)[MAGI2](http://www.ncbi.nlm.nih.gov/gene/?term=9863)(22.373)[APOE](http://www.ncbi.nlm.nih.gov/gene/?term=348)(22.373) [SOX15](http://www.ncbi.nlm.nih.gov/gene/?term=6665)(22.373)[CNTNAP4](http://www.ncbi.nlm.nih.gov/gene/?term=85445)(22.373)[RNASE4](http://www.ncbi.nlm.nih.gov/gene/?term=6038)(22.373)[RAB3A](http://www.ncbi.nlm.nih.gov/gene/?term=5864)(22.373)[MC4R](http://www.ncbi.nlm.nih.gov/gene/?term=4160)(22.373) [IL1B](http://www.ncbi.nlm.nih.gov/gene/?term=3553)(22.373)[GRIN2A](http://www.ncbi.nlm.nih.gov/gene/?term=2903)(22.373)[SLC17A7](http://www.ncbi.nlm.nih.gov/gene/?term=57030)(22.373)[CYP11A1](http://www.ncbi.nlm.nih.gov/gene/?term=1583)(22.373)[SCN5A](http://www.ncbi.nlm.nih.gov/gene/?term=6331)(22.373) [SCN10A](http://www.ncbi.nlm.nih.gov/gene/?term=6336)(22.373)[SPARC](http://www.ncbi.nlm.nih.gov/gene/?term=6678)(22.373)[SORCS3](http://www.ncbi.nlm.nih.gov/gene/?term=22986)(22.373)[RNASE8](http://www.ncbi.nlm.nih.gov/gene/?term=122665)(22.373)[KCNIP2](http://www.ncbi.nlm.nih.gov/gene/?term=30819)(22.373) [GPX7](http://www.ncbi.nlm.nih.gov/gene/?term=2882)(22.373)[CRLF1](http://www.ncbi.nlm.nih.gov/gene/?term=9244)(22.373)[FADD](http://www.ncbi.nlm.nih.gov/gene/?term=8772)(22.373)[SNTG2](http://www.ncbi.nlm.nih.gov/gene/?term=54221)(22.373)[SCGB1A1](http://www.ncbi.nlm.nih.gov/gene/?term=7356)(22.373) [OXTR](http://www.ncbi.nlm.nih.gov/gene/?term=5021)(22.373)[ALDH1B1](http://www.ncbi.nlm.nih.gov/gene/?term=219)(22.373)[LEP](http://www.ncbi.nlm.nih.gov/gene/?term=3952)(22.373)[NLGN1](http://www.ncbi.nlm.nih.gov/gene/?term=22871)(22.373)[CACNA1D](http://www.ncbi.nlm.nih.gov/gene/?term=776)(22.373) [RAPGEF2](http://www.ncbi.nlm.nih.gov/gene/?term=9693)(22.373)[DARS](http://www.ncbi.nlm.nih.gov/gene/?term=1615)(22.373)[ANK3](http://www.ncbi.nlm.nih.gov/gene/?term=288)(22.373)[KCNE5](http://www.ncbi.nlm.nih.gov/gene/?term=23630)(22.373)[PPARD](http://www.ncbi.nlm.nih.gov/gene/?term=5467)(22.373) [NKX2-1](http://www.ncbi.nlm.nih.gov/gene/?term=7080)(22.373)[MIP](http://www.ncbi.nlm.nih.gov/gene/?term=4284)(22.373)[RYR3](http://www.ncbi.nlm.nih.gov/gene/?term=6263)(22.373)[ZPR1](http://www.ncbi.nlm.nih.gov/gene/?term=8882)(22.373)[ALDH3B1](http://www.ncbi.nlm.nih.gov/gene/?term=221)(22.373) [NPPA](http://www.ncbi.nlm.nih.gov/gene/?term=4878)(22.373)[STX1A](http://www.ncbi.nlm.nih.gov/gene/?term=6804)(22.373)[PAXBP1](http://www.ncbi.nlm.nih.gov/gene/?term=94104)(22.373)[PAX7](http://www.ncbi.nlm.nih.gov/gene/?term=5081)(22.373)[ALDH3B2](http://www.ncbi.nlm.nih.gov/gene/?term=222)(22.373) [RNASE2](http://www.ncbi.nlm.nih.gov/gene/?term=6036)(22.373)[NRXN2](http://www.ncbi.nlm.nih.gov/gene/?term=9379)(22.373)[ADORA1](http://www.ncbi.nlm.nih.gov/gene/?term=134)(22.373)[ADH4](http://www.ncbi.nlm.nih.gov/gene/?term=127)(22.373)[GJA5](http://www.ncbi.nlm.nih.gov/gene/?term=2702)(22.373) [FAS](http://www.ncbi.nlm.nih.gov/gene/?term=355)(22.373)[DAB2IP](http://www.ncbi.nlm.nih.gov/gene/?term=153090)(22.373)[CYP2E1](http://www.ncbi.nlm.nih.gov/gene/?term=1571)(22.373)[ACY1](http://www.ncbi.nlm.nih.gov/gene/?term=95)(22.373) | |
| [Liquiritigenin-7,4'-Diglucoside](http://www.megabionet.org/tcmid/ingredient/12906/) | [SOAT1](http://www.ncbi.nlm.nih.gov/gene/?term=6646)(48.000)[MTTP](http://www.ncbi.nlm.nih.gov/gene/?term=4547)(48.000)[SOAT2](http://www.ncbi.nlm.nih.gov/gene/?term=8435)(48.000) | |
| [8alpha-Methoxyfuranodiene](http://www.megabionet.org/tcmid/ingredient/31541/) | This compound doesn't have any potential target with score larger than 20. | |
| [Beta-Pinene](http://www.megabionet.org/tcmid/ingredient/23545/) | [KCND1](http://www.ncbi.nlm.nih.gov/gene/?term=3750)(80.882)[KCNA3](http://www.ncbi.nlm.nih.gov/gene/?term=3738)(80.882)[PRKAB1](http://www.ncbi.nlm.nih.gov/gene/?term=5564)(80.882)[ADH1A](http://www.ncbi.nlm.nih.gov/gene/?term=124)(80.882)[KCNA10](http://www.ncbi.nlm.nih.gov/gene/?term=3744)(80.882) [GAMT](http://www.ncbi.nlm.nih.gov/gene/?term=2593)(80.882)[KCNC3](http://www.ncbi.nlm.nih.gov/gene/?term=3748)(80.882)[KCNA1](http://www.ncbi.nlm.nih.gov/gene/?term=3736)(80.882)[KCNA2](http://www.ncbi.nlm.nih.gov/gene/?term=3737)(80.882)[TPO](http://www.ncbi.nlm.nih.gov/gene/?term=7173)(80.882) [CAT](http://www.ncbi.nlm.nih.gov/gene/?term=847)(80.882)[KCNB1](http://www.ncbi.nlm.nih.gov/gene/?term=3745)(80.882)[ADH1B](http://www.ncbi.nlm.nih.gov/gene/?term=125)(80.882)[DLG4](http://www.ncbi.nlm.nih.gov/gene/?term=1742)(80.882)[KCNC2](http://www.ncbi.nlm.nih.gov/gene/?term=3747)(80.882) [KCNC1](http://www.ncbi.nlm.nih.gov/gene/?term=3746)(80.882)[ADH1C](http://www.ncbi.nlm.nih.gov/gene/?term=126)(80.882)[KCNB2](http://www.ncbi.nlm.nih.gov/gene/?term=9312)(80.882)[KCNA5](http://www.ncbi.nlm.nih.gov/gene/?term=3741)(80.882)[RNASE1](http://www.ncbi.nlm.nih.gov/gene/?term=6035)(80.882) [KCND2](http://www.ncbi.nlm.nih.gov/gene/?term=3751)(80.882)[ALDH2](http://www.ncbi.nlm.nih.gov/gene/?term=217)(80.882)[KCNA4](http://www.ncbi.nlm.nih.gov/gene/?term=3739)(80.882)[KCNA7](http://www.ncbi.nlm.nih.gov/gene/?term=3743)(80.882)[KCNA6](http://www.ncbi.nlm.nih.gov/gene/?term=3742)(80.882) [KCND3](http://www.ncbi.nlm.nih.gov/gene/?term=3752)(80.882)[GUCY1B3](http://www.ncbi.nlm.nih.gov/gene/?term=2983)(55.444)[GATM](http://www.ncbi.nlm.nih.gov/gene/?term=2628)(55.444)[KCNK4](http://www.ncbi.nlm.nih.gov/gene/?term=50801)(55.444)[IYD](http://www.ncbi.nlm.nih.gov/gene/?term=389434)(55.444) [KCNQ1](http://www.ncbi.nlm.nih.gov/gene/?term=3784)(55.444)[FBP1](http://www.ncbi.nlm.nih.gov/gene/?term=2203)(22.373)[DGKI](http://www.ncbi.nlm.nih.gov/gene/?term=9162)(22.373)[ADH7](http://www.ncbi.nlm.nih.gov/gene/?term=131)(22.373)[NFIB](http://www.ncbi.nlm.nih.gov/gene/?term=4781)(22.373) [CYGB](http://www.ncbi.nlm.nih.gov/gene/?term=114757)(22.373)[NRXN3](http://www.ncbi.nlm.nih.gov/gene/?term=9369)(22.373)[LRRC4B](http://www.ncbi.nlm.nih.gov/gene/?term=94030)(22.373)[AQP8](http://www.ncbi.nlm.nih.gov/gene/?term=343)(22.373)[ARX](http://www.ncbi.nlm.nih.gov/gene/?term=170302)(22.373) [IGF1](http://www.ncbi.nlm.nih.gov/gene/?term=3479)(22.373)[SHANK3](http://www.ncbi.nlm.nih.gov/gene/?term=85358)(22.373)[ACY3](http://www.ncbi.nlm.nih.gov/gene/?term=91703)(22.373)[ASPA](http://www.ncbi.nlm.nih.gov/gene/?term=443)(22.373)[NRXN1](http://www.ncbi.nlm.nih.gov/gene/?term=9378)(22.373) [EDN1](http://www.ncbi.nlm.nih.gov/gene/?term=1906)(22.373)[OXT](http://www.ncbi.nlm.nih.gov/gene/?term=5020)(22.373)[MAGI2](http://www.ncbi.nlm.nih.gov/gene/?term=9863)(22.373)[APOE](http://www.ncbi.nlm.nih.gov/gene/?term=348)(22.373)[SOX15](http://www.ncbi.nlm.nih.gov/gene/?term=6665)(22.373) [CNTNAP4](http://www.ncbi.nlm.nih.gov/gene/?term=85445)(22.373)[RNASE4](http://www.ncbi.nlm.nih.gov/gene/?term=6038)(22.373)[RAB3A](http://www.ncbi.nlm.nih.gov/gene/?term=5864)(22.373)[MC4R](http://www.ncbi.nlm.nih.gov/gene/?term=4160)(22.373)[IL1B](http://www.ncbi.nlm.nih.gov/gene/?term=3553)(22.373) [GRIN2A](http://www.ncbi.nlm.nih.gov/gene/?term=2903)(22.373)[SLC17A7](http://www.ncbi.nlm.nih.gov/gene/?term=57030)(22.373)[CYP11A1](http://www.ncbi.nlm.nih.gov/gene/?term=1583)(22.373)[SCN5A](http://www.ncbi.nlm.nih.gov/gene/?term=6331)(22.373)[SCN10A](http://www.ncbi.nlm.nih.gov/gene/?term=6336)(22.373) [SPARC](http://www.ncbi.nlm.nih.gov/gene/?term=6678)(22.373)[SORCS3](http://www.ncbi.nlm.nih.gov/gene/?term=22986)(22.373)[RNASE8](http://www.ncbi.nlm.nih.gov/gene/?term=122665)(22.373)[KCNIP2](http://www.ncbi.nlm.nih.gov/gene/?term=30819)(22.373)[GPX7](http://www.ncbi.nlm.nih.gov/gene/?term=2882)(22.373) [CRLF1](http://www.ncbi.nlm.nih.gov/gene/?term=9244)(22.373)[FADD](http://www.ncbi.nlm.nih.gov/gene/?term=8772)(22.373)[SNTG2](http://www.ncbi.nlm.nih.gov/gene/?term=54221)(22.373)[SCGB1A1](http://www.ncbi.nlm.nih.gov/gene/?term=7356)(22.373)[OXTR](http://www.ncbi.nlm.nih.gov/gene/?term=5021)(22.373) [ALDH1B1](http://www.ncbi.nlm.nih.gov/gene/?term=219)(22.373)[LEP](http://www.ncbi.nlm.nih.gov/gene/?term=3952)(22.373)[NLGN1](http://www.ncbi.nlm.nih.gov/gene/?term=22871)(22.373)[CACNA1D](http://www.ncbi.nlm.nih.gov/gene/?term=776)(22.373)[RAPGEF2](http://www.ncbi.nlm.nih.gov/gene/?term=9693)(22.373) [DARS](http://www.ncbi.nlm.nih.gov/gene/?term=1615)(22.373)[ANK3](http://www.ncbi.nlm.nih.gov/gene/?term=288)(22.373)[KCNE5](http://www.ncbi.nlm.nih.gov/gene/?term=23630)(22.373)[PPARD](http://www.ncbi.nlm.nih.gov/gene/?term=5467)(22.373)[NKX2-1](http://www.ncbi.nlm.nih.gov/gene/?term=7080)(22.373) [MIP](http://www.ncbi.nlm.nih.gov/gene/?term=4284)(22.373)[RYR3](http://www.ncbi.nlm.nih.gov/gene/?term=6263)(22.373)[ZPR1](http://www.ncbi.nlm.nih.gov/gene/?term=8882)(22.373)[ALDH3B1](http://www.ncbi.nlm.nih.gov/gene/?term=221)(22.373)[NPPA](http://www.ncbi.nlm.nih.gov/gene/?term=4878)(22.373) [STX1A](http://www.ncbi.nlm.nih.gov/gene/?term=6804)(22.373)[PAXBP1](http://www.ncbi.nlm.nih.gov/gene/?term=94104)(22.373)[PAX7](http://www.ncbi.nlm.nih.gov/gene/?term=5081)(22.373)[ALDH3B2](http://www.ncbi.nlm.nih.gov/gene/?term=222)(22.373)[RNASE2](http://www.ncbi.nlm.nih.gov/gene/?term=6036)(22.373) [NRXN2](http://www.ncbi.nlm.nih.gov/gene/?term=9379)(22.373)[ADORA1](http://www.ncbi.nlm.nih.gov/gene/?term=134)(22.373)[ADH4](http://www.ncbi.nlm.nih.gov/gene/?term=127)(22.373)[GJA5](http://www.ncbi.nlm.nih.gov/gene/?term=2702)(22.373)[FAS](http://www.ncbi.nlm.nih.gov/gene/?term=355)(22.373) [DAB2IP](http://www.ncbi.nlm.nih.gov/gene/?term=153090)(22.373)[CYP2E1](http://www.ncbi.nlm.nih.gov/gene/?term=1571)(22.373)[ACY1](http://www.ncbi.nlm.nih.gov/gene/?term=95)(22.373) | |
| [Rutin](http://www.megabionet.org/tcmid/ingredient/19072/) | This compound doesn't have any potential target with score larger than 20. | |
| [Methylglyoxal](http://www.megabionet.org/tcmid/ingredient/14467/) | [PLA2G1B](http://www.ncbi.nlm.nih.gov/gene/?term=5319)(known target in DrugBank)[GSR](http://www.ncbi.nlm.nih.gov/gene/?term=2936)(140.500)[MGST3](http://www.ncbi.nlm.nih.gov/gene/?term=4259)(140.500)[GPX4](http://www.ncbi.nlm.nih.gov/gene/?term=2879)(140.500)[GSTM2](http://www.ncbi.nlm.nih.gov/gene/?term=2946)(140.500) [GGT1](http://www.ncbi.nlm.nih.gov/gene/?term=2678)(140.500)[MGST2](http://www.ncbi.nlm.nih.gov/gene/?term=4258)(140.500)[GSS](http://www.ncbi.nlm.nih.gov/gene/?term=2937)(140.500)[GSTA5](http://www.ncbi.nlm.nih.gov/gene/?term=221357)(140.500)[GSTM5](http://www.ncbi.nlm.nih.gov/gene/?term=2949)(140.500) [GLO1](http://www.ncbi.nlm.nih.gov/gene/?term=2739)(140.500)[GSTZ1](http://www.ncbi.nlm.nih.gov/gene/?term=2954)(140.500)[GSTO2](http://www.ncbi.nlm.nih.gov/gene/?term=119391)(140.500)[GPX3](http://www.ncbi.nlm.nih.gov/gene/?term=2878)(140.500)[GSTA2](http://www.ncbi.nlm.nih.gov/gene/?term=2939)(140.500) [GPX7](http://www.ncbi.nlm.nih.gov/gene/?term=2882)(140.500)[GSTA4](http://www.ncbi.nlm.nih.gov/gene/?term=2941)(140.500)[GPX8](http://www.ncbi.nlm.nih.gov/gene/?term=493869)(140.500)[GLRX](http://www.ncbi.nlm.nih.gov/gene/?term=2745)(140.500)[GSTM4](http://www.ncbi.nlm.nih.gov/gene/?term=2948)(140.500) [GPX6](http://www.ncbi.nlm.nih.gov/gene/?term=257202)(140.500)[MGST1](http://www.ncbi.nlm.nih.gov/gene/?term=4257)(140.500)[GPX5](http://www.ncbi.nlm.nih.gov/gene/?term=2880)(140.500)[GSTM1](http://www.ncbi.nlm.nih.gov/gene/?term=2944)(140.500)[GSTO1](http://www.ncbi.nlm.nih.gov/gene/?term=9446)(140.500) [GSTT1](http://www.ncbi.nlm.nih.gov/gene/?term=2952)(140.500)[LTC4S](http://www.ncbi.nlm.nih.gov/gene/?term=4056)(140.500)[GSTK1](http://www.ncbi.nlm.nih.gov/gene/?term=373156)(140.500)[GPX1](http://www.ncbi.nlm.nih.gov/gene/?term=2876)(140.500)[GLRX2](http://www.ncbi.nlm.nih.gov/gene/?term=51022)(140.500) [GSTM3](http://www.ncbi.nlm.nih.gov/gene/?term=2947)(140.500)[GSTA3](http://www.ncbi.nlm.nih.gov/gene/?term=2940)(140.500)[HPGDS](http://www.ncbi.nlm.nih.gov/gene/?term=27306)(140.500)[GSTP1](http://www.ncbi.nlm.nih.gov/gene/?term=2950)(140.500)[TXNDC12](http://www.ncbi.nlm.nih.gov/gene/?term=51060)(140.500) [GPX2](http://www.ncbi.nlm.nih.gov/gene/?term=2877)(140.500)[HAGH](http://www.ncbi.nlm.nih.gov/gene/?term=3029)(140.500)[GSTA1](http://www.ncbi.nlm.nih.gov/gene/?term=2938)(140.500)[ESD](http://www.ncbi.nlm.nih.gov/gene/?term=2098)(140.500) | |
| [Hispaglabridin A](http://www.megabionet.org/tcmid/ingredient/9556/) | [CNR2](http://www.ncbi.nlm.nih.gov/gene/?term=1269)(80.882)[CNR1](http://www.ncbi.nlm.nih.gov/gene/?term=1268)(80.882)[DRD2](http://www.ncbi.nlm.nih.gov/gene/?term=1813)(55.444)[SEC14L3](http://www.ncbi.nlm.nih.gov/gene/?term=266629)(23.000)[PPP2CA](http://www.ncbi.nlm.nih.gov/gene/?term=5515)(23.000) [PRKCA](http://www.ncbi.nlm.nih.gov/gene/?term=5578)(23.000)[NR1I2](http://www.ncbi.nlm.nih.gov/gene/?term=8856)(23.000)[ALOX5](http://www.ncbi.nlm.nih.gov/gene/?term=240)(23.000)[PPP2CB](http://www.ncbi.nlm.nih.gov/gene/?term=5516)(23.000)[SEC14L2](http://www.ncbi.nlm.nih.gov/gene/?term=23541)(23.000) [DGKA](http://www.ncbi.nlm.nih.gov/gene/?term=1606)(23.000)[PRKCB](http://www.ncbi.nlm.nih.gov/gene/?term=5579)(23.000)[SEC14L4](http://www.ncbi.nlm.nih.gov/gene/?term=284904)(23.000)[AKT1](http://www.ncbi.nlm.nih.gov/gene/?term=207)(22.373)[CCR7](http://www.ncbi.nlm.nih.gov/gene/?term=1236)(22.373) [ABHD6](http://www.ncbi.nlm.nih.gov/gene/?term=57406)(22.373)[MGLL](http://www.ncbi.nlm.nih.gov/gene/?term=11343)(22.373)[FCER1G](http://www.ncbi.nlm.nih.gov/gene/?term=2207)(22.373)[SUMO1](http://www.ncbi.nlm.nih.gov/gene/?term=7341)(22.373)[GPR55](http://www.ncbi.nlm.nih.gov/gene/?term=9290)(22.373) [CHRNB2](http://www.ncbi.nlm.nih.gov/gene/?term=1141)(22.373)[FCER1A](http://www.ncbi.nlm.nih.gov/gene/?term=2205)(22.373)[CAV3](http://www.ncbi.nlm.nih.gov/gene/?term=859)(22.373)[RNF207](http://www.ncbi.nlm.nih.gov/gene/?term=388591)(22.373)[C3](http://www.ncbi.nlm.nih.gov/gene/?term=718)(22.373) [DAGLA](http://www.ncbi.nlm.nih.gov/gene/?term=747)(22.373)[PLIN5](http://www.ncbi.nlm.nih.gov/gene/?term=440503)(22.373)[ZP3](http://www.ncbi.nlm.nih.gov/gene/?term=7784)(22.373) | |
| [Astragaloside I](http://www.megabionet.org/tcmid/ingredient/1936/) | [SLC47A1](http://www.ncbi.nlm.nih.gov/gene/?term=55244)(48.000)[CYP3A4](http://www.ncbi.nlm.nih.gov/gene/?term=1576)(48.000)[CYP51A1](http://www.ncbi.nlm.nih.gov/gene/?term=1595)(48.000)[KCNH2](http://www.ncbi.nlm.nih.gov/gene/?term=3757)(48.000)[ATP1A1](http://www.ncbi.nlm.nih.gov/gene/?term=476)(48.000) [MLNR](http://www.ncbi.nlm.nih.gov/gene/?term=2862)(48.000)[SLCO1B3](http://www.ncbi.nlm.nih.gov/gene/?term=28234)(48.000)[ALB](http://www.ncbi.nlm.nih.gov/gene/?term=213)(48.000)[ABCB1](http://www.ncbi.nlm.nih.gov/gene/?term=5243)(48.000)[SLCO1B1](http://www.ncbi.nlm.nih.gov/gene/?term=10599)(48.000) | |
| [Licopyranocoumarin](http://www.megabionet.org/tcmid/ingredient/12776/) | This compound doesn't have any potential target with score larger than 20. | |
| [3,5-Dimethoxystilbene](http://www.megabionet.org/tcmid/ingredient/6292/) | [PDE4A](http://www.ncbi.nlm.nih.gov/gene/?term=5141)(80.882)[PDE3A](http://www.ncbi.nlm.nih.gov/gene/?term=5139)(80.882)[CACNA1C](http://www.ncbi.nlm.nih.gov/gene/?term=775)(80.882)[TRDMT1](http://www.ncbi.nlm.nih.gov/gene/?term=1787)(80.882)[DHFRL1](http://www.ncbi.nlm.nih.gov/gene/?term=200895)(80.882) [DHFR](http://www.ncbi.nlm.nih.gov/gene/?term=1719)(80.882)[PDE10A](http://www.ncbi.nlm.nih.gov/gene/?term=10846)(80.882)[PDE2A](http://www.ncbi.nlm.nih.gov/gene/?term=5138)(80.882)[TYMS](http://www.ncbi.nlm.nih.gov/gene/?term=7298)(80.882)[SHMT1](http://www.ncbi.nlm.nih.gov/gene/?term=6470)(55.444) [PDE3B](http://www.ncbi.nlm.nih.gov/gene/?term=5140)(55.444)[DMTN](http://www.ncbi.nlm.nih.gov/gene/?term=2039)(55.444)[KCNMA1](http://www.ncbi.nlm.nih.gov/gene/?term=3778)(55.444)[ABCC2](http://www.ncbi.nlm.nih.gov/gene/?term=1244)(22.373)[FOLR2](http://www.ncbi.nlm.nih.gov/gene/?term=2350)(22.373) [PDE5A](http://www.ncbi.nlm.nih.gov/gene/?term=8654)(22.373)[PDE9A](http://www.ncbi.nlm.nih.gov/gene/?term=5152)(22.373)[TBPL1](http://www.ncbi.nlm.nih.gov/gene/?term=9519)(22.373)[DNMT3A](http://www.ncbi.nlm.nih.gov/gene/?term=1788)(22.373)[STUB1](http://www.ncbi.nlm.nih.gov/gene/?term=10273)(22.373) [INSR](http://www.ncbi.nlm.nih.gov/gene/?term=3643)(22.373)[P2RX2](http://www.ncbi.nlm.nih.gov/gene/?term=22953)(22.373)[HTR2A](http://www.ncbi.nlm.nih.gov/gene/?term=3356)(22.373)[SIRT2](http://www.ncbi.nlm.nih.gov/gene/?term=22933)(22.373)[HCN4](http://www.ncbi.nlm.nih.gov/gene/?term=10021)(22.373) [DPYD](http://www.ncbi.nlm.nih.gov/gene/?term=1806)(22.373)[HOXA5](http://www.ncbi.nlm.nih.gov/gene/?term=3202)(22.373)[FBLN1](http://www.ncbi.nlm.nih.gov/gene/?term=2192)(22.373)[SLC22A6](http://www.ncbi.nlm.nih.gov/gene/?term=9356)(22.373)[ASNS](http://www.ncbi.nlm.nih.gov/gene/?term=440)(22.373) [ADK](http://www.ncbi.nlm.nih.gov/gene/?term=132)(22.373)[IL1B](http://www.ncbi.nlm.nih.gov/gene/?term=3553)(22.373)[NT5M](http://www.ncbi.nlm.nih.gov/gene/?term=56953)(22.373)[P2RX3](http://www.ncbi.nlm.nih.gov/gene/?term=5024)(22.373)[AURKA](http://www.ncbi.nlm.nih.gov/gene/?term=6790)(22.373) [GAS6](http://www.ncbi.nlm.nih.gov/gene/?term=2621)(22.373)[CMPK2](http://www.ncbi.nlm.nih.gov/gene/?term=129607)(22.373)[PDE1B](http://www.ncbi.nlm.nih.gov/gene/?term=5153)(22.373)[DPYS](http://www.ncbi.nlm.nih.gov/gene/?term=1807)(22.373)[CHRNA3](http://www.ncbi.nlm.nih.gov/gene/?term=1136)(22.373) [HSP90AB1](http://www.ncbi.nlm.nih.gov/gene/?term=3326)(22.373)[CHRNB2](http://www.ncbi.nlm.nih.gov/gene/?term=1141)(22.373)[SLC6A4](http://www.ncbi.nlm.nih.gov/gene/?term=6532)(22.373)[DUT](http://www.ncbi.nlm.nih.gov/gene/?term=1854)(22.373)[RAPGEF2](http://www.ncbi.nlm.nih.gov/gene/?term=9693)(22.373) [ATIC](http://www.ncbi.nlm.nih.gov/gene/?term=471)(22.373)[AK9](http://www.ncbi.nlm.nih.gov/gene/?term=221264)(22.373)[CDKN1A](http://www.ncbi.nlm.nih.gov/gene/?term=1026)(22.373)[HIF1A](http://www.ncbi.nlm.nih.gov/gene/?term=3091)(22.373)[FOLR1](http://www.ncbi.nlm.nih.gov/gene/?term=2348)(22.373) [RAB8B](http://www.ncbi.nlm.nih.gov/gene/?term=51762)(22.373)[HYAL2](http://www.ncbi.nlm.nih.gov/gene/?term=8692)(22.373)[DNMT3B](http://www.ncbi.nlm.nih.gov/gene/?term=1789)(22.373)[HSP90AA1](http://www.ncbi.nlm.nih.gov/gene/?term=3320)(22.373)[DNMT1](http://www.ncbi.nlm.nih.gov/gene/?term=1786)(22.373) [PARP10](http://www.ncbi.nlm.nih.gov/gene/?term=84875)(22.373)[CHRNB4](http://www.ncbi.nlm.nih.gov/gene/?term=1143)(22.373)[MGMT](http://www.ncbi.nlm.nih.gov/gene/?term=4255)(22.373)[DTYMK](http://www.ncbi.nlm.nih.gov/gene/?term=1841)(22.373)[PDE11A](http://www.ncbi.nlm.nih.gov/gene/?term=50940)(22.373) [HCN2](http://www.ncbi.nlm.nih.gov/gene/?term=610)(22.373) | |
| [Uralenol](http://www.megabionet.org/tcmid/ingredient/22225/) | This compound doesn't have any potential target with score larger than 20. | |
| [Lupiwighteone](http://www.megabionet.org/tcmid/ingredient/13100/) | This compound doesn't have any potential target with score larger than 20. | |
| [Glycyrrhizic Acid](http://www.megabionet.org/tcmid/ingredient/23251/) | [NR3C1](http://www.ncbi.nlm.nih.gov/gene/?term=2908)(48.000)[ATP1A1](http://www.ncbi.nlm.nih.gov/gene/?term=476)(48.000) | |
| [3-Methylcarbazole](http://www.megabionet.org/tcmid/ingredient/14209/) | [GABRB2](http://www.ncbi.nlm.nih.gov/gene/?term=2561)(80.882)[GABRB3](http://www.ncbi.nlm.nih.gov/gene/?term=2562)(80.882)[F2](http://www.ncbi.nlm.nih.gov/gene/?term=2147)(80.882)[GPR27](http://www.ncbi.nlm.nih.gov/gene/?term=2850)(55.444)[CD36](http://www.ncbi.nlm.nih.gov/gene/?term=948)(22.373) [SDC4](http://www.ncbi.nlm.nih.gov/gene/?term=6385)(22.373)[CD47](http://www.ncbi.nlm.nih.gov/gene/?term=961)(22.373)[BICD1](http://www.ncbi.nlm.nih.gov/gene/?term=636)(22.373) | |
| [2,4,4'-Trihydroxychalcone](http://www.megabionet.org/tcmid/ingredient/21678/) | [HSD17B1](http://www.ncbi.nlm.nih.gov/gene/?term=3292)(80.882)[ESR1](http://www.ncbi.nlm.nih.gov/gene/?term=2099)(23.000)[CFTR](http://www.ncbi.nlm.nih.gov/gene/?term=1080)(23.000)[AKR1C3](http://www.ncbi.nlm.nih.gov/gene/?term=8644)(22.373)[HSD17B6](http://www.ncbi.nlm.nih.gov/gene/?term=8630)(22.373) [HSD17B2](http://www.ncbi.nlm.nih.gov/gene/?term=3294)(22.373)[CYP11A1](http://www.ncbi.nlm.nih.gov/gene/?term=1583)(22.373)[WNT4](http://www.ncbi.nlm.nih.gov/gene/?term=54361)(22.373)[STAR](http://www.ncbi.nlm.nih.gov/gene/?term=6770)(22.373)[HSD17B8](http://www.ncbi.nlm.nih.gov/gene/?term=7923)(22.373) [DHRS9](http://www.ncbi.nlm.nih.gov/gene/?term=10170)(22.373)[HSD17B3](http://www.ncbi.nlm.nih.gov/gene/?term=3293)(22.373) | |
| [Ononitol](http://www.megabionet.org/tcmid/ingredient/16108/) | This compound doesn't have any potential target with score larger than 20. | |
| [Ethyl-P-Digallate](http://www.megabionet.org/tcmid/ingredient/7469/) | This compound doesn't have any potential target with score larger than 20. | |
| [Methyl Caprylate](http://www.megabionet.org/tcmid/ingredient/14208/) | [NPR1](http://www.ncbi.nlm.nih.gov/gene/?term=4881)(80.882)[SCN11A](http://www.ncbi.nlm.nih.gov/gene/?term=11280)(48.000)[SCN2B](http://www.ncbi.nlm.nih.gov/gene/?term=6327)(48.000)[ABAT](http://www.ncbi.nlm.nih.gov/gene/?term=18)(48.000)[SCN1A](http://www.ncbi.nlm.nih.gov/gene/?term=6323)(48.000) [ESRRG](http://www.ncbi.nlm.nih.gov/gene/?term=2104)(48.000)[SCN3B](http://www.ncbi.nlm.nih.gov/gene/?term=55800)(48.000)[COX6C](http://www.ncbi.nlm.nih.gov/gene/?term=1345)(48.000)[COX5B](http://www.ncbi.nlm.nih.gov/gene/?term=1329)(48.000)[COX7C](http://www.ncbi.nlm.nih.gov/gene/?term=1350)(48.000) [COX1](http://www.ncbi.nlm.nih.gov/gene/?term=4512)(48.000)[SCN3A](http://www.ncbi.nlm.nih.gov/gene/?term=6328)(48.000)[AKR1C2](http://www.ncbi.nlm.nih.gov/gene/?term=1646)(48.000)[SCN7A](http://www.ncbi.nlm.nih.gov/gene/?term=6332)(48.000)[SCN5A](http://www.ncbi.nlm.nih.gov/gene/?term=6331)(48.000) [SCN10A](http://www.ncbi.nlm.nih.gov/gene/?term=6336)(48.000)[AKR1D1](http://www.ncbi.nlm.nih.gov/gene/?term=6718)(48.000)[ALDH5A1](http://www.ncbi.nlm.nih.gov/gene/?term=7915)(48.000)[SCN2A](http://www.ncbi.nlm.nih.gov/gene/?term=6326)(48.000)[COX5A](http://www.ncbi.nlm.nih.gov/gene/?term=9377)(48.000) [COX3](http://www.ncbi.nlm.nih.gov/gene/?term=4514)(48.000)[AR](http://www.ncbi.nlm.nih.gov/gene/?term=367)(48.000)[COX7A1](http://www.ncbi.nlm.nih.gov/gene/?term=1346)(48.000)[FECH](http://www.ncbi.nlm.nih.gov/gene/?term=2235)(48.000)[COX4I1](http://www.ncbi.nlm.nih.gov/gene/?term=1327)(48.000) [HDAC9](http://www.ncbi.nlm.nih.gov/gene/?term=9734)(48.000)[PLA2G1B](http://www.ncbi.nlm.nih.gov/gene/?term=5319)(48.000)[COX6A2](http://www.ncbi.nlm.nih.gov/gene/?term=1339)(48.000)[TYR](http://www.ncbi.nlm.nih.gov/gene/?term=7299)(48.000)[SCN9A](http://www.ncbi.nlm.nih.gov/gene/?term=6335)(48.000) [SCN4A](http://www.ncbi.nlm.nih.gov/gene/?term=6329)(48.000)[SCN4B](http://www.ncbi.nlm.nih.gov/gene/?term=6330)(48.000)[ADH1C](http://www.ncbi.nlm.nih.gov/gene/?term=126)(48.000)[COX6B1](http://www.ncbi.nlm.nih.gov/gene/?term=1340)(48.000)[FABP6](http://www.ncbi.nlm.nih.gov/gene/?term=2172)(48.000) [CES1](http://www.ncbi.nlm.nih.gov/gene/?term=1066)(48.000)[SRD5A2](http://www.ncbi.nlm.nih.gov/gene/?term=6716)(48.000)[ACADSB](http://www.ncbi.nlm.nih.gov/gene/?term=36)(48.000)[SCN8A](http://www.ncbi.nlm.nih.gov/gene/?term=6334)(48.000)[COX7B](http://www.ncbi.nlm.nih.gov/gene/?term=1349)(48.000) [SCN1B](http://www.ncbi.nlm.nih.gov/gene/?term=6324)(48.000)[COX2](http://www.ncbi.nlm.nih.gov/gene/?term=4513)(48.000)[OGDH](http://www.ncbi.nlm.nih.gov/gene/?term=4967)(48.000)[HDAC2](http://www.ncbi.nlm.nih.gov/gene/?term=3066)(48.000)[COX8A](http://www.ncbi.nlm.nih.gov/gene/?term=1351)(48.000) [NR1H4](http://www.ncbi.nlm.nih.gov/gene/?term=9971)(48.000)[NPR3](http://www.ncbi.nlm.nih.gov/gene/?term=4883)(22.373)[NPR2](http://www.ncbi.nlm.nih.gov/gene/?term=4882)(22.373) | |
| [Xylose](http://www.megabionet.org/tcmid/ingredient/22827/) | [NFKB2](http://www.ncbi.nlm.nih.gov/gene/?term=4791)(48.000)[TNF](http://www.ncbi.nlm.nih.gov/gene/?term=7124)(48.000)[MMP9](http://www.ncbi.nlm.nih.gov/gene/?term=4318)(48.000)[IFNG](http://www.ncbi.nlm.nih.gov/gene/?term=3458)(48.000) | |
| [Stigmasteryl Ferulate](http://www.megabionet.org/tcmid/ingredient/20360/) | This compound doesn't have any potential target with score larger than 20. | |
| [Licofuranocoumarin](http://www.megabionet.org/tcmid/ingredient/12771/) | This compound doesn't have any potential target with score larger than 20. | |
| [3'-Methoxyglabridin](http://www.megabionet.org/tcmid/ingredient/13934/) | [CNR2](http://www.ncbi.nlm.nih.gov/gene/?term=1269)(80.882)[CNR1](http://www.ncbi.nlm.nih.gov/gene/?term=1268)(80.882)[DRD2](http://www.ncbi.nlm.nih.gov/gene/?term=1813)(55.444)[SEC14L3](http://www.ncbi.nlm.nih.gov/gene/?term=266629)(23.000)[PPP2CA](http://www.ncbi.nlm.nih.gov/gene/?term=5515)(23.000) [PRKCA](http://www.ncbi.nlm.nih.gov/gene/?term=5578)(23.000)[NR1I2](http://www.ncbi.nlm.nih.gov/gene/?term=8856)(23.000)[ALOX5](http://www.ncbi.nlm.nih.gov/gene/?term=240)(23.000)[PPP2CB](http://www.ncbi.nlm.nih.gov/gene/?term=5516)(23.000)[SEC14L2](http://www.ncbi.nlm.nih.gov/gene/?term=23541)(23.000) [DGKA](http://www.ncbi.nlm.nih.gov/gene/?term=1606)(23.000)[PRKCB](http://www.ncbi.nlm.nih.gov/gene/?term=5579)(23.000)[SEC14L4](http://www.ncbi.nlm.nih.gov/gene/?term=284904)(23.000)[AKT1](http://www.ncbi.nlm.nih.gov/gene/?term=207)(22.373)[CCR7](http://www.ncbi.nlm.nih.gov/gene/?term=1236)(22.373) [ABHD6](http://www.ncbi.nlm.nih.gov/gene/?term=57406)(22.373)[MGLL](http://www.ncbi.nlm.nih.gov/gene/?term=11343)(22.373)[FCER1G](http://www.ncbi.nlm.nih.gov/gene/?term=2207)(22.373)[SUMO1](http://www.ncbi.nlm.nih.gov/gene/?term=7341)(22.373)[GPR55](http://www.ncbi.nlm.nih.gov/gene/?term=9290)(22.373) [CHRNB2](http://www.ncbi.nlm.nih.gov/gene/?term=1141)(22.373)[FCER1A](http://www.ncbi.nlm.nih.gov/gene/?term=2205)(22.373)[CAV3](http://www.ncbi.nlm.nih.gov/gene/?term=859)(22.373)[RNF207](http://www.ncbi.nlm.nih.gov/gene/?term=388591)(22.373)[C3](http://www.ncbi.nlm.nih.gov/gene/?term=718)(22.373) [DAGLA](http://www.ncbi.nlm.nih.gov/gene/?term=747)(22.373)[PLIN5](http://www.ncbi.nlm.nih.gov/gene/?term=440503)(22.373)[ZP3](http://www.ncbi.nlm.nih.gov/gene/?term=7784)(22.373) | |
| [Gancaonin P-3'-Methylether](http://www.megabionet.org/tcmid/ingredient/8141/) | This compound doesn't have any potential target with score larger than 20. | |
| [Lauric Aldehyde](http://www.megabionet.org/tcmid/ingredient/31420/) | [SCN11A](http://www.ncbi.nlm.nih.gov/gene/?term=11280)(48.000)[SCN2B](http://www.ncbi.nlm.nih.gov/gene/?term=6327)(48.000)[ABAT](http://www.ncbi.nlm.nih.gov/gene/?term=18)(48.000)[SCN1A](http://www.ncbi.nlm.nih.gov/gene/?term=6323)(48.000)[SCN3B](http://www.ncbi.nlm.nih.gov/gene/?term=55800)(48.000) [SCN3A](http://www.ncbi.nlm.nih.gov/gene/?term=6328)(48.000)[SCN7A](http://www.ncbi.nlm.nih.gov/gene/?term=6332)(48.000)[SCN5A](http://www.ncbi.nlm.nih.gov/gene/?term=6331)(48.000)[SCN10A](http://www.ncbi.nlm.nih.gov/gene/?term=6336)(48.000)[AKR1D1](http://www.ncbi.nlm.nih.gov/gene/?term=6718)(48.000) [ALDH5A1](http://www.ncbi.nlm.nih.gov/gene/?term=7915)(48.000)[SCN2A](http://www.ncbi.nlm.nih.gov/gene/?term=6326)(48.000)[HDAC9](http://www.ncbi.nlm.nih.gov/gene/?term=9734)(48.000)[TYR](http://www.ncbi.nlm.nih.gov/gene/?term=7299)(48.000)[SCN9A](http://www.ncbi.nlm.nih.gov/gene/?term=6335)(48.000) [SCN4A](http://www.ncbi.nlm.nih.gov/gene/?term=6329)(48.000)[SCN4B](http://www.ncbi.nlm.nih.gov/gene/?term=6330)(48.000)[SRD5A2](http://www.ncbi.nlm.nih.gov/gene/?term=6716)(48.000)[ACADSB](http://www.ncbi.nlm.nih.gov/gene/?term=36)(48.000)[SCN8A](http://www.ncbi.nlm.nih.gov/gene/?term=6334)(48.000) [SCN1B](http://www.ncbi.nlm.nih.gov/gene/?term=6324)(48.000)[OGDH](http://www.ncbi.nlm.nih.gov/gene/?term=4967)(48.000)[HDAC2](http://www.ncbi.nlm.nih.gov/gene/?term=3066)(48.000) | |
| [Astragaloside Vii](http://www.megabionet.org/tcmid/ingredient/1942/) | [CXCR4](http://www.ncbi.nlm.nih.gov/gene/?term=7852)(48.000) | |
| [Licoricidin](http://www.megabionet.org/tcmid/ingredient/12787/) | [CNR2](http://www.ncbi.nlm.nih.gov/gene/?term=1269)(80.882)[CNR1](http://www.ncbi.nlm.nih.gov/gene/?term=1268)(80.882)[DRD2](http://www.ncbi.nlm.nih.gov/gene/?term=1813)(55.444)[SEC14L3](http://www.ncbi.nlm.nih.gov/gene/?term=266629)(23.000)[PPP2CA](http://www.ncbi.nlm.nih.gov/gene/?term=5515)(23.000) [PRKCA](http://www.ncbi.nlm.nih.gov/gene/?term=5578)(23.000)[NR1I2](http://www.ncbi.nlm.nih.gov/gene/?term=8856)(23.000)[ALOX5](http://www.ncbi.nlm.nih.gov/gene/?term=240)(23.000)[PPP2CB](http://www.ncbi.nlm.nih.gov/gene/?term=5516)(23.000)[SEC14L2](http://www.ncbi.nlm.nih.gov/gene/?term=23541)(23.000) [DGKA](http://www.ncbi.nlm.nih.gov/gene/?term=1606)(23.000)[PRKCB](http://www.ncbi.nlm.nih.gov/gene/?term=5579)(23.000)[SEC14L4](http://www.ncbi.nlm.nih.gov/gene/?term=284904)(23.000)[AKT1](http://www.ncbi.nlm.nih.gov/gene/?term=207)(22.373)[CCR7](http://www.ncbi.nlm.nih.gov/gene/?term=1236)(22.373) [ABHD6](http://www.ncbi.nlm.nih.gov/gene/?term=57406)(22.373)[MGLL](http://www.ncbi.nlm.nih.gov/gene/?term=11343)(22.373)[FCER1G](http://www.ncbi.nlm.nih.gov/gene/?term=2207)(22.373)[SUMO1](http://www.ncbi.nlm.nih.gov/gene/?term=7341)(22.373)[GPR55](http://www.ncbi.nlm.nih.gov/gene/?term=9290)(22.373) [CHRNB2](http://www.ncbi.nlm.nih.gov/gene/?term=1141)(22.373)[FCER1A](http://www.ncbi.nlm.nih.gov/gene/?term=2205)(22.373)[CAV3](http://www.ncbi.nlm.nih.gov/gene/?term=859)(22.373)[RNF207](http://www.ncbi.nlm.nih.gov/gene/?term=388591)(22.373)[C3](http://www.ncbi.nlm.nih.gov/gene/?term=718)(22.373) [DAGLA](http://www.ncbi.nlm.nih.gov/gene/?term=747)(22.373)[PLIN5](http://www.ncbi.nlm.nih.gov/gene/?term=440503)(22.373)[ZP3](http://www.ncbi.nlm.nih.gov/gene/?term=7784)(22.373) | |
| [Tert-Butylbenzene](http://www.megabionet.org/tcmid/ingredient/24593/) | [GABRB2](http://www.ncbi.nlm.nih.gov/gene/?term=2561)(80.882)[GABRB3](http://www.ncbi.nlm.nih.gov/gene/?term=2562)(80.882)[F2](http://www.ncbi.nlm.nih.gov/gene/?term=2147)(80.882)[GPR27](http://www.ncbi.nlm.nih.gov/gene/?term=2850)(55.444)[SLC6A3](http://www.ncbi.nlm.nih.gov/gene/?term=6531)(48.000) [ADRA1A](http://www.ncbi.nlm.nih.gov/gene/?term=148)(48.000)[GABRA2](http://www.ncbi.nlm.nih.gov/gene/?term=2555)(48.000)[GABRD](http://www.ncbi.nlm.nih.gov/gene/?term=2563)(48.000)[ADRB2](http://www.ncbi.nlm.nih.gov/gene/?term=154)(48.000)[GABRB1](http://www.ncbi.nlm.nih.gov/gene/?term=2560)(48.000) [GABRG3](http://www.ncbi.nlm.nih.gov/gene/?term=2567)(48.000)[ADRA2A](http://www.ncbi.nlm.nih.gov/gene/?term=150)(48.000)[GABRE](http://www.ncbi.nlm.nih.gov/gene/?term=2564)(48.000)[MAOB](http://www.ncbi.nlm.nih.gov/gene/?term=4129)(48.000)[SCN2A](http://www.ncbi.nlm.nih.gov/gene/?term=6326)(48.000) [GABRA3](http://www.ncbi.nlm.nih.gov/gene/?term=2556)(48.000)[ADRB1](http://www.ncbi.nlm.nih.gov/gene/?term=153)(48.000)[GABRG1](http://www.ncbi.nlm.nih.gov/gene/?term=2565)(48.000)[GABRP](http://www.ncbi.nlm.nih.gov/gene/?term=2568)(48.000)[ADRA1D](http://www.ncbi.nlm.nih.gov/gene/?term=146)(48.000) [GABRA4](http://www.ncbi.nlm.nih.gov/gene/?term=2557)(48.000)[DRD2](http://www.ncbi.nlm.nih.gov/gene/?term=1813)(48.000)[MAOA](http://www.ncbi.nlm.nih.gov/gene/?term=4128)(48.000)[SLC6A4](http://www.ncbi.nlm.nih.gov/gene/?term=6532)(48.000)[SLC18A2](http://www.ncbi.nlm.nih.gov/gene/?term=6571)(48.000) [SCN4A](http://www.ncbi.nlm.nih.gov/gene/?term=6329)(48.000)[GABRQ](http://www.ncbi.nlm.nih.gov/gene/?term=55879)(48.000)[ADRA1B](http://www.ncbi.nlm.nih.gov/gene/?term=147)(48.000)[ADRA2C](http://www.ncbi.nlm.nih.gov/gene/?term=152)(48.000)[GABRA5](http://www.ncbi.nlm.nih.gov/gene/?term=2558)(48.000) [GABRA6](http://www.ncbi.nlm.nih.gov/gene/?term=2559)(48.000)[GABRA1](http://www.ncbi.nlm.nih.gov/gene/?term=2554)(48.000)[GABRG2](http://www.ncbi.nlm.nih.gov/gene/?term=2566)(48.000)[ADRB3](http://www.ncbi.nlm.nih.gov/gene/?term=155)(48.000)[TAAR1](http://www.ncbi.nlm.nih.gov/gene/?term=134864)(48.000) [CARTPT](http://www.ncbi.nlm.nih.gov/gene/?term=9607)(48.000)[ADRA2B](http://www.ncbi.nlm.nih.gov/gene/?term=151)(48.000)[SLC6A2](http://www.ncbi.nlm.nih.gov/gene/?term=6530)(48.000)[CD36](http://www.ncbi.nlm.nih.gov/gene/?term=948)(22.373)[SDC4](http://www.ncbi.nlm.nih.gov/gene/?term=6385)(22.373) [CD47](http://www.ncbi.nlm.nih.gov/gene/?term=961)(22.373)[BICD1](http://www.ncbi.nlm.nih.gov/gene/?term=636)(22.373) | |
| [Licochalcone A](http://www.megabionet.org/tcmid/ingredient/12760/) | This compound doesn't have any potential target with score larger than 20. | |
| [Choline](http://www.megabionet.org/tcmid/ingredient/3589/) | [PLD1](http://www.ncbi.nlm.nih.gov/gene/?term=5337)(686.000)[PCYT1A](http://www.ncbi.nlm.nih.gov/gene/?term=5130)(686.000)[PCYT1B](http://www.ncbi.nlm.nih.gov/gene/?term=9468)(686.000)[PHOSPHO1](http://www.ncbi.nlm.nih.gov/gene/?term=162466)(686.000)[ACHE](http://www.ncbi.nlm.nih.gov/gene/?term=43)(686.000) [PLD2](http://www.ncbi.nlm.nih.gov/gene/?term=5338)(686.000)[BCHE](http://www.ncbi.nlm.nih.gov/gene/?term=590)(686.000)[COLQ](http://www.ncbi.nlm.nih.gov/gene/?term=8292)(55.444)[SLC5A7](http://www.ncbi.nlm.nih.gov/gene/?term=60482)(22.373)[HMOX1](http://www.ncbi.nlm.nih.gov/gene/?term=3162)(22.373) [NRG1](http://www.ncbi.nlm.nih.gov/gene/?term=3084)(22.373)[DMGDH](http://www.ncbi.nlm.nih.gov/gene/?term=29958)(22.373)[SIX3](http://www.ncbi.nlm.nih.gov/gene/?term=6496)(22.373)[RAP1GAP](http://www.ncbi.nlm.nih.gov/gene/?term=5909)(22.373)[CRP](http://www.ncbi.nlm.nih.gov/gene/?term=1401)(22.373) [NAPEPLD](http://www.ncbi.nlm.nih.gov/gene/?term=222236)(22.373)[FSCN1](http://www.ncbi.nlm.nih.gov/gene/?term=6624)(22.373)[PRSS12](http://www.ncbi.nlm.nih.gov/gene/?term=8492)(22.373)[KLF5](http://www.ncbi.nlm.nih.gov/gene/?term=688)(22.373)[SLC44A4](http://www.ncbi.nlm.nih.gov/gene/?term=80736)(22.373) [ALDH7A1](http://www.ncbi.nlm.nih.gov/gene/?term=501)(22.373)[DNM3](http://www.ncbi.nlm.nih.gov/gene/?term=26052)(22.373)[PLD4](http://www.ncbi.nlm.nih.gov/gene/?term=122618)(22.373)[ASCL1](http://www.ncbi.nlm.nih.gov/gene/?term=429)(22.373)[GPLD1](http://www.ncbi.nlm.nih.gov/gene/?term=2822)(22.373) [ENPP6](http://www.ncbi.nlm.nih.gov/gene/?term=133121)(22.373)[CDH8](http://www.ncbi.nlm.nih.gov/gene/?term=1006)(22.373)[PLD3](http://www.ncbi.nlm.nih.gov/gene/?term=23646)(22.373)[PODXL](http://www.ncbi.nlm.nih.gov/gene/?term=5420)(22.373)[FNTA](http://www.ncbi.nlm.nih.gov/gene/?term=2339)(22.373) [AGRN](http://www.ncbi.nlm.nih.gov/gene/?term=375790)(22.373)[CHKA](http://www.ncbi.nlm.nih.gov/gene/?term=1119)(22.373)[CHDH](http://www.ncbi.nlm.nih.gov/gene/?term=55349)(22.373)[ATP8B1](http://www.ncbi.nlm.nih.gov/gene/?term=5205)(22.373)[GRIN1](http://www.ncbi.nlm.nih.gov/gene/?term=2902)(22.373) | |
| [Hispaglabridin B](http://www.megabionet.org/tcmid/ingredient/9557/) | [CNR2](http://www.ncbi.nlm.nih.gov/gene/?term=1269)(80.882)[CNR1](http://www.ncbi.nlm.nih.gov/gene/?term=1268)(80.882)[DRD2](http://www.ncbi.nlm.nih.gov/gene/?term=1813)(55.444)[SEC14L3](http://www.ncbi.nlm.nih.gov/gene/?term=266629)(23.000)[PPP2CA](http://www.ncbi.nlm.nih.gov/gene/?term=5515)(23.000) [PRKCA](http://www.ncbi.nlm.nih.gov/gene/?term=5578)(23.000)[NR1I2](http://www.ncbi.nlm.nih.gov/gene/?term=8856)(23.000)[ALOX5](http://www.ncbi.nlm.nih.gov/gene/?term=240)(23.000)[PPP2CB](http://www.ncbi.nlm.nih.gov/gene/?term=5516)(23.000)[SEC14L2](http://www.ncbi.nlm.nih.gov/gene/?term=23541)(23.000) [DGKA](http://www.ncbi.nlm.nih.gov/gene/?term=1606)(23.000)[PRKCB](http://www.ncbi.nlm.nih.gov/gene/?term=5579)(23.000)[SEC14L4](http://www.ncbi.nlm.nih.gov/gene/?term=284904)(23.000)[AKT1](http://www.ncbi.nlm.nih.gov/gene/?term=207)(22.373)[CCR7](http://www.ncbi.nlm.nih.gov/gene/?term=1236)(22.373) [ABHD6](http://www.ncbi.nlm.nih.gov/gene/?term=57406)(22.373)[MGLL](http://www.ncbi.nlm.nih.gov/gene/?term=11343)(22.373)[FCER1G](http://www.ncbi.nlm.nih.gov/gene/?term=2207)(22.373)[SUMO1](http://www.ncbi.nlm.nih.gov/gene/?term=7341)(22.373)[GPR55](http://www.ncbi.nlm.nih.gov/gene/?term=9290)(22.373) [CHRNB2](http://www.ncbi.nlm.nih.gov/gene/?term=1141)(22.373)[FCER1A](http://www.ncbi.nlm.nih.gov/gene/?term=2205)(22.373)[CAV3](http://www.ncbi.nlm.nih.gov/gene/?term=859)(22.373)[RNF207](http://www.ncbi.nlm.nih.gov/gene/?term=388591)(22.373)[C3](http://www.ncbi.nlm.nih.gov/gene/?term=718)(22.373) [DAGLA](http://www.ncbi.nlm.nih.gov/gene/?term=747)(22.373)[PLIN5](http://www.ncbi.nlm.nih.gov/gene/?term=440503)(22.373)[ZP3](http://www.ncbi.nlm.nih.gov/gene/?term=7784)(22.373) | |
| [Methyl Palmitate](http://www.megabionet.org/tcmid/ingredient/23038/) | [NPR1](http://www.ncbi.nlm.nih.gov/gene/?term=4881)(80.882)[SCN11A](http://www.ncbi.nlm.nih.gov/gene/?term=11280)(48.000)[SCN2B](http://www.ncbi.nlm.nih.gov/gene/?term=6327)(48.000)[ABAT](http://www.ncbi.nlm.nih.gov/gene/?term=18)(48.000)[SCN1A](http://www.ncbi.nlm.nih.gov/gene/?term=6323)(48.000) [ESRRG](http://www.ncbi.nlm.nih.gov/gene/?term=2104)(48.000)[SCN3B](http://www.ncbi.nlm.nih.gov/gene/?term=55800)(48.000)[COX6C](http://www.ncbi.nlm.nih.gov/gene/?term=1345)(48.000)[COX5B](http://www.ncbi.nlm.nih.gov/gene/?term=1329)(48.000)[COX7C](http://www.ncbi.nlm.nih.gov/gene/?term=1350)(48.000) [COX1](http://www.ncbi.nlm.nih.gov/gene/?term=4512)(48.000)[SCN3A](http://www.ncbi.nlm.nih.gov/gene/?term=6328)(48.000)[AKR1C2](http://www.ncbi.nlm.nih.gov/gene/?term=1646)(48.000)[SCN7A](http://www.ncbi.nlm.nih.gov/gene/?term=6332)(48.000)[SCN5A](http://www.ncbi.nlm.nih.gov/gene/?term=6331)(48.000) [SCN10A](http://www.ncbi.nlm.nih.gov/gene/?term=6336)(48.000)[AKR1D1](http://www.ncbi.nlm.nih.gov/gene/?term=6718)(48.000)[ALDH5A1](http://www.ncbi.nlm.nih.gov/gene/?term=7915)(48.000)[SCN2A](http://www.ncbi.nlm.nih.gov/gene/?term=6326)(48.000)[COX5A](http://www.ncbi.nlm.nih.gov/gene/?term=9377)(48.000) [COX3](http://www.ncbi.nlm.nih.gov/gene/?term=4514)(48.000)[AR](http://www.ncbi.nlm.nih.gov/gene/?term=367)(48.000)[COX7A1](http://www.ncbi.nlm.nih.gov/gene/?term=1346)(48.000)[FECH](http://www.ncbi.nlm.nih.gov/gene/?term=2235)(48.000)[COX4I1](http://www.ncbi.nlm.nih.gov/gene/?term=1327)(48.000) [HDAC9](http://www.ncbi.nlm.nih.gov/gene/?term=9734)(48.000)[PLA2G1B](http://www.ncbi.nlm.nih.gov/gene/?term=5319)(48.000)[COX6A2](http://www.ncbi.nlm.nih.gov/gene/?term=1339)(48.000)[TYR](http://www.ncbi.nlm.nih.gov/gene/?term=7299)(48.000)[SCN9A](http://www.ncbi.nlm.nih.gov/gene/?term=6335)(48.000) [SCN4A](http://www.ncbi.nlm.nih.gov/gene/?term=6329)(48.000)[SCN4B](http://www.ncbi.nlm.nih.gov/gene/?term=6330)(48.000)[ADH1C](http://www.ncbi.nlm.nih.gov/gene/?term=126)(48.000)[COX6B1](http://www.ncbi.nlm.nih.gov/gene/?term=1340)(48.000)[FABP6](http://www.ncbi.nlm.nih.gov/gene/?term=2172)(48.000) [CES1](http://www.ncbi.nlm.nih.gov/gene/?term=1066)(48.000)[SRD5A2](http://www.ncbi.nlm.nih.gov/gene/?term=6716)(48.000)[ACADSB](http://www.ncbi.nlm.nih.gov/gene/?term=36)(48.000)[SCN8A](http://www.ncbi.nlm.nih.gov/gene/?term=6334)(48.000)[COX7B](http://www.ncbi.nlm.nih.gov/gene/?term=1349)(48.000) [SCN1B](http://www.ncbi.nlm.nih.gov/gene/?term=6324)(48.000)[COX2](http://www.ncbi.nlm.nih.gov/gene/?term=4513)(48.000)[OGDH](http://www.ncbi.nlm.nih.gov/gene/?term=4967)(48.000)[HDAC2](http://www.ncbi.nlm.nih.gov/gene/?term=3066)(48.000)[COX8A](http://www.ncbi.nlm.nih.gov/gene/?term=1351)(48.000) [NR1H4](http://www.ncbi.nlm.nih.gov/gene/?term=9971)(48.000)[NPR3](http://www.ncbi.nlm.nih.gov/gene/?term=4883)(22.373)[NPR2](http://www.ncbi.nlm.nih.gov/gene/?term=4882)(22.373) | |
| [8-Methoxy-5-O-Glucoside Flavone](http://www.megabionet.org/tcmid/ingredient/13935/) | This compound doesn't have any potential target with score larger than 20. | |
| [Hentriacontane](http://www.megabionet.org/tcmid/ingredient/9362/) | This compound doesn't have any potential target with score larger than 20. | |
| [Myristicin](http://www.megabionet.org/tcmid/ingredient/15196/) | This compound doesn't have any potential target with score larger than 20. | |
| [1-Heptadecanol](http://www.megabionet.org/tcmid/ingredient/9378/) | [TRPV3](http://www.ncbi.nlm.nih.gov/gene/?term=162514)(122.778)[OPRK1](http://www.ncbi.nlm.nih.gov/gene/?term=4986)(122.778)[TRPM8](http://www.ncbi.nlm.nih.gov/gene/?term=79054)(122.778)[TRPA1](http://www.ncbi.nlm.nih.gov/gene/?term=8989)(122.778)[CACNB1](http://www.ncbi.nlm.nih.gov/gene/?term=782)(80.882) [GRIA4](http://www.ncbi.nlm.nih.gov/gene/?term=2893)(80.882)[GABRA2](http://www.ncbi.nlm.nih.gov/gene/?term=2555)(80.882)[CACNG2](http://www.ncbi.nlm.nih.gov/gene/?term=10369)(80.882)[GABRD](http://www.ncbi.nlm.nih.gov/gene/?term=2563)(80.882)[CHRNA4](http://www.ncbi.nlm.nih.gov/gene/?term=1137)(80.882) [CHRNB3](http://www.ncbi.nlm.nih.gov/gene/?term=1142)(80.882)[GABRB1](http://www.ncbi.nlm.nih.gov/gene/?term=2560)(80.882)[GRIA1](http://www.ncbi.nlm.nih.gov/gene/?term=2890)(80.882)[GLRA2](http://www.ncbi.nlm.nih.gov/gene/?term=2742)(80.882)[KCNJ3](http://www.ncbi.nlm.nih.gov/gene/?term=3760)(80.882) [GABRG3](http://www.ncbi.nlm.nih.gov/gene/?term=2567)(80.882)[VCAM1](http://www.ncbi.nlm.nih.gov/gene/?term=7412)(80.882)[CHRNA7](http://www.ncbi.nlm.nih.gov/gene/?term=1139)(80.882)[KCNJ6](http://www.ncbi.nlm.nih.gov/gene/?term=3763)(80.882)[GABRE](http://www.ncbi.nlm.nih.gov/gene/?term=2564)(80.882) [GABRA3](http://www.ncbi.nlm.nih.gov/gene/?term=2556)(80.882)[SLC29A1](http://www.ncbi.nlm.nih.gov/gene/?term=2030)(80.882)[CACNA1S](http://www.ncbi.nlm.nih.gov/gene/?term=779)(80.882)[GABRG1](http://www.ncbi.nlm.nih.gov/gene/?term=2565)(80.882)[GABRP](http://www.ncbi.nlm.nih.gov/gene/?term=2568)(80.882) [CHRNA10](http://www.ncbi.nlm.nih.gov/gene/?term=57053)(80.882)[GRIA2](http://www.ncbi.nlm.nih.gov/gene/?term=2891)(80.882)[GABRA4](http://www.ncbi.nlm.nih.gov/gene/?term=2557)(80.882)[CHRNA3](http://www.ncbi.nlm.nih.gov/gene/?term=1136)(80.882)[GABRB2](http://www.ncbi.nlm.nih.gov/gene/?term=2561)(80.882) [CACNA1C](http://www.ncbi.nlm.nih.gov/gene/?term=775)(80.882)[CHRNB2](http://www.ncbi.nlm.nih.gov/gene/?term=1141)(80.882)[CACNA1D](http://www.ncbi.nlm.nih.gov/gene/?term=776)(80.882)[HTR3D](http://www.ncbi.nlm.nih.gov/gene/?term=200909)(80.882)[GABRQ](http://www.ncbi.nlm.nih.gov/gene/?term=55879)(80.882) [CHRNA2](http://www.ncbi.nlm.nih.gov/gene/?term=1135)(80.882)[CHRNA6](http://www.ncbi.nlm.nih.gov/gene/?term=8973)(80.882)[GABRB3](http://www.ncbi.nlm.nih.gov/gene/?term=2562)(80.882)[KCNJ5](http://www.ncbi.nlm.nih.gov/gene/?term=3762)(80.882)[GRIN3A](http://www.ncbi.nlm.nih.gov/gene/?term=116443)(80.882) [HTR3B](http://www.ncbi.nlm.nih.gov/gene/?term=9177)(80.882)[GRIA3](http://www.ncbi.nlm.nih.gov/gene/?term=2892)(80.882)[GABRA5](http://www.ncbi.nlm.nih.gov/gene/?term=2558)(80.882)[SLC29A2](http://www.ncbi.nlm.nih.gov/gene/?term=3177)(80.882)[HTR3E](http://www.ncbi.nlm.nih.gov/gene/?term=285242)(80.882) [GABRA6](http://www.ncbi.nlm.nih.gov/gene/?term=2559)(80.882)[HTR3A](http://www.ncbi.nlm.nih.gov/gene/?term=3359)(80.882)[GABRA1](http://www.ncbi.nlm.nih.gov/gene/?term=2554)(80.882)[CHRNA9](http://www.ncbi.nlm.nih.gov/gene/?term=55584)(80.882)[CHRFAM7A](http://www.ncbi.nlm.nih.gov/gene/?term=89832)(80.882) [CHRNA5](http://www.ncbi.nlm.nih.gov/gene/?term=1138)(80.882)[CHRNB4](http://www.ncbi.nlm.nih.gov/gene/?term=1143)(80.882)[KCNJ9](http://www.ncbi.nlm.nih.gov/gene/?term=3765)(80.882)[HTR3C](http://www.ncbi.nlm.nih.gov/gene/?term=170572)(80.882)[GLRA1](http://www.ncbi.nlm.nih.gov/gene/?term=2741)(80.882) [CACNG1](http://www.ncbi.nlm.nih.gov/gene/?term=786)(80.882)[ADAM8](http://www.ncbi.nlm.nih.gov/gene/?term=101)(55.444)[SLC28A3](http://www.ncbi.nlm.nih.gov/gene/?term=64078)(55.444)[ADRA1B](http://www.ncbi.nlm.nih.gov/gene/?term=147)(55.444)[FOXL2](http://www.ncbi.nlm.nih.gov/gene/?term=668)(55.444) [DMTN](http://www.ncbi.nlm.nih.gov/gene/?term=2039)(55.444)[KCNMA1](http://www.ncbi.nlm.nih.gov/gene/?term=3778)(55.444)[SCN11A](http://www.ncbi.nlm.nih.gov/gene/?term=11280)(48.000)[SCN2B](http://www.ncbi.nlm.nih.gov/gene/?term=6327)(48.000)[ABAT](http://www.ncbi.nlm.nih.gov/gene/?term=18)(48.000) [SCN1A](http://www.ncbi.nlm.nih.gov/gene/?term=6323)(48.000)[SCN3B](http://www.ncbi.nlm.nih.gov/gene/?term=55800)(48.000)[SCN3A](http://www.ncbi.nlm.nih.gov/gene/?term=6328)(48.000)[SCN7A](http://www.ncbi.nlm.nih.gov/gene/?term=6332)(48.000)[SCN5A](http://www.ncbi.nlm.nih.gov/gene/?term=6331)(48.000) [SCN10A](http://www.ncbi.nlm.nih.gov/gene/?term=6336)(48.000)[AKR1D1](http://www.ncbi.nlm.nih.gov/gene/?term=6718)(48.000)[ALDH5A1](http://www.ncbi.nlm.nih.gov/gene/?term=7915)(48.000)[SCN2A](http://www.ncbi.nlm.nih.gov/gene/?term=6326)(48.000)[HDAC9](http://www.ncbi.nlm.nih.gov/gene/?term=9734)(48.000) [TYR](http://www.ncbi.nlm.nih.gov/gene/?term=7299)(48.000)[SCN9A](http://www.ncbi.nlm.nih.gov/gene/?term=6335)(48.000)[SCN4A](http://www.ncbi.nlm.nih.gov/gene/?term=6329)(48.000)[SCN4B](http://www.ncbi.nlm.nih.gov/gene/?term=6330)(48.000)[SRD5A2](http://www.ncbi.nlm.nih.gov/gene/?term=6716)(48.000) [ACADSB](http://www.ncbi.nlm.nih.gov/gene/?term=36)(48.000)[SCN8A](http://www.ncbi.nlm.nih.gov/gene/?term=6334)(48.000)[SCN1B](http://www.ncbi.nlm.nih.gov/gene/?term=6324)(48.000)[OGDH](http://www.ncbi.nlm.nih.gov/gene/?term=4967)(48.000)[HDAC2](http://www.ncbi.nlm.nih.gov/gene/?term=3066)(48.000) [GLRA3](http://www.ncbi.nlm.nih.gov/gene/?term=8001)(23.000)[HMGA2](http://www.ncbi.nlm.nih.gov/gene/?term=8091)(22.373)[CACNA1H](http://www.ncbi.nlm.nih.gov/gene/?term=8912)(22.373)[SLITRK6](http://www.ncbi.nlm.nih.gov/gene/?term=84189)(22.373)[UTS2](http://www.ncbi.nlm.nih.gov/gene/?term=10911)(22.373) [NAV2](http://www.ncbi.nlm.nih.gov/gene/?term=89797)(22.373)[P2RX2](http://www.ncbi.nlm.nih.gov/gene/?term=22953)(22.373)[HTR2A](http://www.ncbi.nlm.nih.gov/gene/?term=3356)(22.373)[AVPR1A](http://www.ncbi.nlm.nih.gov/gene/?term=552)(22.373)[ADORA2A](http://www.ncbi.nlm.nih.gov/gene/?term=135)(22.373) [CACNA1A](http://www.ncbi.nlm.nih.gov/gene/?term=773)(22.373)[ANK2](http://www.ncbi.nlm.nih.gov/gene/?term=287)(22.373)[ZP4](http://www.ncbi.nlm.nih.gov/gene/?term=57829)(22.373)[SUMO1](http://www.ncbi.nlm.nih.gov/gene/?term=7341)(22.373)[CNR2](http://www.ncbi.nlm.nih.gov/gene/?term=1269)(22.373) [MTNR1B](http://www.ncbi.nlm.nih.gov/gene/?term=4544)(22.373)[MAOB](http://www.ncbi.nlm.nih.gov/gene/?term=4129)(22.373)[GLRB](http://www.ncbi.nlm.nih.gov/gene/?term=2743)(22.373)[P2RX3](http://www.ncbi.nlm.nih.gov/gene/?term=5024)(22.373)[SLC44A4](http://www.ncbi.nlm.nih.gov/gene/?term=80736)(22.373) [FLOT1](http://www.ncbi.nlm.nih.gov/gene/?term=10211)(22.373)[NPY5R](http://www.ncbi.nlm.nih.gov/gene/?term=4889)(22.373)[MSN](http://www.ncbi.nlm.nih.gov/gene/?term=4478)(22.373)[GHRL](http://www.ncbi.nlm.nih.gov/gene/?term=51738)(22.373)[TBR1](http://www.ncbi.nlm.nih.gov/gene/?term=10716)(22.373) [ADRA1D](http://www.ncbi.nlm.nih.gov/gene/?term=146)(22.373)[DRD2](http://www.ncbi.nlm.nih.gov/gene/?term=1813)(22.373)[AVP](http://www.ncbi.nlm.nih.gov/gene/?term=551)(22.373)[PAX2](http://www.ncbi.nlm.nih.gov/gene/?term=5076)(22.373)[AOC1](http://www.ncbi.nlm.nih.gov/gene/?term=26)(22.373) [MAOA](http://www.ncbi.nlm.nih.gov/gene/?term=4128)(22.373)[UTS2R](http://www.ncbi.nlm.nih.gov/gene/?term=2837)(22.373)[ADIRF](http://www.ncbi.nlm.nih.gov/gene/?term=10974)(22.373)[GHRH](http://www.ncbi.nlm.nih.gov/gene/?term=2691)(22.373)[HCRT](http://www.ncbi.nlm.nih.gov/gene/?term=3060)(22.373) [RAB3B](http://www.ncbi.nlm.nih.gov/gene/?term=5865)(22.373)[KCNE5](http://www.ncbi.nlm.nih.gov/gene/?term=23630)(22.373)[CNR1](http://www.ncbi.nlm.nih.gov/gene/?term=1268)(22.373)[SLC18A3](http://www.ncbi.nlm.nih.gov/gene/?term=6572)(22.373)[EZR](http://www.ncbi.nlm.nih.gov/gene/?term=7430)(22.373) [DRD4](http://www.ncbi.nlm.nih.gov/gene/?term=1815)(22.373)[CRH](http://www.ncbi.nlm.nih.gov/gene/?term=1392)(22.373)[GLI3](http://www.ncbi.nlm.nih.gov/gene/?term=2737)(22.373)[CAV3](http://www.ncbi.nlm.nih.gov/gene/?term=859)(22.373)[SLC9A6](http://www.ncbi.nlm.nih.gov/gene/?term=10479)(22.373) [AOC3](http://www.ncbi.nlm.nih.gov/gene/?term=8639)(22.373)[NCOA1](http://www.ncbi.nlm.nih.gov/gene/?term=8648)(22.373)[AOC2](http://www.ncbi.nlm.nih.gov/gene/?term=314)(22.373)[IL6](http://www.ncbi.nlm.nih.gov/gene/?term=3569)(22.373)[GLRA4](http://www.ncbi.nlm.nih.gov/gene/?term=441509)(22.373) [RNF207](http://www.ncbi.nlm.nih.gov/gene/?term=388591)(22.373)[ROCK1](http://www.ncbi.nlm.nih.gov/gene/?term=6093)(22.373)[KCNA5](http://www.ncbi.nlm.nih.gov/gene/?term=3741)(22.373)[TACR2](http://www.ncbi.nlm.nih.gov/gene/?term=6865)(22.373)[NRP1](http://www.ncbi.nlm.nih.gov/gene/?term=8829)(22.373) [NPPA](http://www.ncbi.nlm.nih.gov/gene/?term=4878)(22.373)[KMT2A](http://www.ncbi.nlm.nih.gov/gene/?term=4297)(22.373)[TNR](http://www.ncbi.nlm.nih.gov/gene/?term=7143)(22.373)[ZP3](http://www.ncbi.nlm.nih.gov/gene/?term=7784)(22.373)[PLCB1](http://www.ncbi.nlm.nih.gov/gene/?term=23236)(22.373) [EPHB1](http://www.ncbi.nlm.nih.gov/gene/?term=2047)(22.373)[DRD1](http://www.ncbi.nlm.nih.gov/gene/?term=1812)(22.373)[EEA1](http://www.ncbi.nlm.nih.gov/gene/?term=8411)(22.373)[POU4F3](http://www.ncbi.nlm.nih.gov/gene/?term=5459)(22.373)[CALCA](http://www.ncbi.nlm.nih.gov/gene/?term=796)(22.373) [PINK1](http://www.ncbi.nlm.nih.gov/gene/?term=65018)(22.373)[KCNQ1](http://www.ncbi.nlm.nih.gov/gene/?term=3784)(22.373)[ICAM1](http://www.ncbi.nlm.nih.gov/gene/?term=3383)(22.373)[CARTPT](http://www.ncbi.nlm.nih.gov/gene/?term=9607)(22.373)[ATP8B1](http://www.ncbi.nlm.nih.gov/gene/?term=5205)(22.373) [KISS1](http://www.ncbi.nlm.nih.gov/gene/?term=3814)(22.373)[GRM7](http://www.ncbi.nlm.nih.gov/gene/?term=2917)(22.373)[FOS](http://www.ncbi.nlm.nih.gov/gene/?term=2353)(22.373)[GRIN1](http://www.ncbi.nlm.nih.gov/gene/?term=2902)(22.373) | |
| [Phebalosin](http://www.megabionet.org/tcmid/ingredient/17037/) | This compound doesn't have any potential target with score larger than 20. | |
| [Licoricone](http://www.megabionet.org/tcmid/ingredient/12788/) | This compound doesn't have any potential target with score larger than 20. | |
| [N-Nonanol](http://www.megabionet.org/tcmid/ingredient/15677/) | [TRPV3](http://www.ncbi.nlm.nih.gov/gene/?term=162514)(122.778)[OPRK1](http://www.ncbi.nlm.nih.gov/gene/?term=4986)(122.778)[TRPM8](http://www.ncbi.nlm.nih.gov/gene/?term=79054)(122.778)[TRPA1](http://www.ncbi.nlm.nih.gov/gene/?term=8989)(122.778)[CACNB1](http://www.ncbi.nlm.nih.gov/gene/?term=782)(80.882) [GRIA4](http://www.ncbi.nlm.nih.gov/gene/?term=2893)(80.882)[GABRA2](http://www.ncbi.nlm.nih.gov/gene/?term=2555)(80.882)[CACNG2](http://www.ncbi.nlm.nih.gov/gene/?term=10369)(80.882)[GABRD](http://www.ncbi.nlm.nih.gov/gene/?term=2563)(80.882)[CHRNA4](http://www.ncbi.nlm.nih.gov/gene/?term=1137)(80.882) [CHRNB3](http://www.ncbi.nlm.nih.gov/gene/?term=1142)(80.882)[GABRB1](http://www.ncbi.nlm.nih.gov/gene/?term=2560)(80.882)[GRIA1](http://www.ncbi.nlm.nih.gov/gene/?term=2890)(80.882)[GLRA2](http://www.ncbi.nlm.nih.gov/gene/?term=2742)(80.882)[KCNJ3](http://www.ncbi.nlm.nih.gov/gene/?term=3760)(80.882) [GABRG3](http://www.ncbi.nlm.nih.gov/gene/?term=2567)(80.882)[VCAM1](http://www.ncbi.nlm.nih.gov/gene/?term=7412)(80.882)[CHRNA7](http://www.ncbi.nlm.nih.gov/gene/?term=1139)(80.882)[KCNJ6](http://www.ncbi.nlm.nih.gov/gene/?term=3763)(80.882)[GABRE](http://www.ncbi.nlm.nih.gov/gene/?term=2564)(80.882) [GABRA3](http://www.ncbi.nlm.nih.gov/gene/?term=2556)(80.882)[SLC29A1](http://www.ncbi.nlm.nih.gov/gene/?term=2030)(80.882)[CACNA1S](http://www.ncbi.nlm.nih.gov/gene/?term=779)(80.882)[GABRG1](http://www.ncbi.nlm.nih.gov/gene/?term=2565)(80.882)[GABRP](http://www.ncbi.nlm.nih.gov/gene/?term=2568)(80.882) [CHRNA10](http://www.ncbi.nlm.nih.gov/gene/?term=57053)(80.882)[GRIA2](http://www.ncbi.nlm.nih.gov/gene/?term=2891)(80.882)[GABRA4](http://www.ncbi.nlm.nih.gov/gene/?term=2557)(80.882)[CHRNA3](http://www.ncbi.nlm.nih.gov/gene/?term=1136)(80.882)[GABRB2](http://www.ncbi.nlm.nih.gov/gene/?term=2561)(80.882) [CACNA1C](http://www.ncbi.nlm.nih.gov/gene/?term=775)(80.882)[CHRNB2](http://www.ncbi.nlm.nih.gov/gene/?term=1141)(80.882)[CACNA1D](http://www.ncbi.nlm.nih.gov/gene/?term=776)(80.882)[HTR3D](http://www.ncbi.nlm.nih.gov/gene/?term=200909)(80.882)[GABRQ](http://www.ncbi.nlm.nih.gov/gene/?term=55879)(80.882) [CHRNA2](http://www.ncbi.nlm.nih.gov/gene/?term=1135)(80.882)[CHRNA6](http://www.ncbi.nlm.nih.gov/gene/?term=8973)(80.882)[GABRB3](http://www.ncbi.nlm.nih.gov/gene/?term=2562)(80.882)[KCNJ5](http://www.ncbi.nlm.nih.gov/gene/?term=3762)(80.882)[GRIN3A](http://www.ncbi.nlm.nih.gov/gene/?term=116443)(80.882) [HTR3B](http://www.ncbi.nlm.nih.gov/gene/?term=9177)(80.882)[GRIA3](http://www.ncbi.nlm.nih.gov/gene/?term=2892)(80.882)[GABRA5](http://www.ncbi.nlm.nih.gov/gene/?term=2558)(80.882)[SLC29A2](http://www.ncbi.nlm.nih.gov/gene/?term=3177)(80.882)[HTR3E](http://www.ncbi.nlm.nih.gov/gene/?term=285242)(80.882) [GABRA6](http://www.ncbi.nlm.nih.gov/gene/?term=2559)(80.882)[HTR3A](http://www.ncbi.nlm.nih.gov/gene/?term=3359)(80.882)[GABRA1](http://www.ncbi.nlm.nih.gov/gene/?term=2554)(80.882)[CHRNA9](http://www.ncbi.nlm.nih.gov/gene/?term=55584)(80.882)[CHRFAM7A](http://www.ncbi.nlm.nih.gov/gene/?term=89832)(80.882) [CHRNA5](http://www.ncbi.nlm.nih.gov/gene/?term=1138)(80.882)[CHRNB4](http://www.ncbi.nlm.nih.gov/gene/?term=1143)(80.882)[KCNJ9](http://www.ncbi.nlm.nih.gov/gene/?term=3765)(80.882)[HTR3C](http://www.ncbi.nlm.nih.gov/gene/?term=170572)(80.882)[GLRA1](http://www.ncbi.nlm.nih.gov/gene/?term=2741)(80.882) [CACNG1](http://www.ncbi.nlm.nih.gov/gene/?term=786)(80.882)[ADAM8](http://www.ncbi.nlm.nih.gov/gene/?term=101)(55.444)[SLC28A3](http://www.ncbi.nlm.nih.gov/gene/?term=64078)(55.444)[ADRA1B](http://www.ncbi.nlm.nih.gov/gene/?term=147)(55.444)[FOXL2](http://www.ncbi.nlm.nih.gov/gene/?term=668)(55.444) [DMTN](http://www.ncbi.nlm.nih.gov/gene/?term=2039)(55.444)[KCNMA1](http://www.ncbi.nlm.nih.gov/gene/?term=3778)(55.444)[SCN11A](http://www.ncbi.nlm.nih.gov/gene/?term=11280)(48.000)[SCN2B](http://www.ncbi.nlm.nih.gov/gene/?term=6327)(48.000)[ABAT](http://www.ncbi.nlm.nih.gov/gene/?term=18)(48.000) [SCN1A](http://www.ncbi.nlm.nih.gov/gene/?term=6323)(48.000)[SCN3B](http://www.ncbi.nlm.nih.gov/gene/?term=55800)(48.000)[SCN3A](http://www.ncbi.nlm.nih.gov/gene/?term=6328)(48.000)[SCN7A](http://www.ncbi.nlm.nih.gov/gene/?term=6332)(48.000)[SCN5A](http://www.ncbi.nlm.nih.gov/gene/?term=6331)(48.000) [SCN10A](http://www.ncbi.nlm.nih.gov/gene/?term=6336)(48.000)[AKR1D1](http://www.ncbi.nlm.nih.gov/gene/?term=6718)(48.000)[ALDH5A1](http://www.ncbi.nlm.nih.gov/gene/?term=7915)(48.000)[SCN2A](http://www.ncbi.nlm.nih.gov/gene/?term=6326)(48.000)[HDAC9](http://www.ncbi.nlm.nih.gov/gene/?term=9734)(48.000) [TYR](http://www.ncbi.nlm.nih.gov/gene/?term=7299)(48.000)[SCN9A](http://www.ncbi.nlm.nih.gov/gene/?term=6335)(48.000)[SCN4A](http://www.ncbi.nlm.nih.gov/gene/?term=6329)(48.000)[SCN4B](http://www.ncbi.nlm.nih.gov/gene/?term=6330)(48.000)[SRD5A2](http://www.ncbi.nlm.nih.gov/gene/?term=6716)(48.000) [ACADSB](http://www.ncbi.nlm.nih.gov/gene/?term=36)(48.000)[SCN8A](http://www.ncbi.nlm.nih.gov/gene/?term=6334)(48.000)[SCN1B](http://www.ncbi.nlm.nih.gov/gene/?term=6324)(48.000)[OGDH](http://www.ncbi.nlm.nih.gov/gene/?term=4967)(48.000)[HDAC2](http://www.ncbi.nlm.nih.gov/gene/?term=3066)(48.000) [GLRA3](http://www.ncbi.nlm.nih.gov/gene/?term=8001)(23.000)[HMGA2](http://www.ncbi.nlm.nih.gov/gene/?term=8091)(22.373)[CACNA1H](http://www.ncbi.nlm.nih.gov/gene/?term=8912)(22.373)[SLITRK6](http://www.ncbi.nlm.nih.gov/gene/?term=84189)(22.373)[UTS2](http://www.ncbi.nlm.nih.gov/gene/?term=10911)(22.373) [NAV2](http://www.ncbi.nlm.nih.gov/gene/?term=89797)(22.373)[P2RX2](http://www.ncbi.nlm.nih.gov/gene/?term=22953)(22.373)[HTR2A](http://www.ncbi.nlm.nih.gov/gene/?term=3356)(22.373)[AVPR1A](http://www.ncbi.nlm.nih.gov/gene/?term=552)(22.373)[ADORA2A](http://www.ncbi.nlm.nih.gov/gene/?term=135)(22.373) [CACNA1A](http://www.ncbi.nlm.nih.gov/gene/?term=773)(22.373)[ANK2](http://www.ncbi.nlm.nih.gov/gene/?term=287)(22.373)[ZP4](http://www.ncbi.nlm.nih.gov/gene/?term=57829)(22.373)[SUMO1](http://www.ncbi.nlm.nih.gov/gene/?term=7341)(22.373)[CNR2](http://www.ncbi.nlm.nih.gov/gene/?term=1269)(22.373) [MTNR1B](http://www.ncbi.nlm.nih.gov/gene/?term=4544)(22.373)[MAOB](http://www.ncbi.nlm.nih.gov/gene/?term=4129)(22.373)[GLRB](http://www.ncbi.nlm.nih.gov/gene/?term=2743)(22.373)[P2RX3](http://www.ncbi.nlm.nih.gov/gene/?term=5024)(22.373)[SLC44A4](http://www.ncbi.nlm.nih.gov/gene/?term=80736)(22.373) [FLOT1](http://www.ncbi.nlm.nih.gov/gene/?term=10211)(22.373)[NPY5R](http://www.ncbi.nlm.nih.gov/gene/?term=4889)(22.373)[MSN](http://www.ncbi.nlm.nih.gov/gene/?term=4478)(22.373)[GHRL](http://www.ncbi.nlm.nih.gov/gene/?term=51738)(22.373)[TBR1](http://www.ncbi.nlm.nih.gov/gene/?term=10716)(22.373) [ADRA1D](http://www.ncbi.nlm.nih.gov/gene/?term=146)(22.373)[DRD2](http://www.ncbi.nlm.nih.gov/gene/?term=1813)(22.373)[AVP](http://www.ncbi.nlm.nih.gov/gene/?term=551)(22.373)[PAX2](http://www.ncbi.nlm.nih.gov/gene/?term=5076)(22.373)[AOC1](http://www.ncbi.nlm.nih.gov/gene/?term=26)(22.373) [MAOA](http://www.ncbi.nlm.nih.gov/gene/?term=4128)(22.373)[UTS2R](http://www.ncbi.nlm.nih.gov/gene/?term=2837)(22.373)[ADIRF](http://www.ncbi.nlm.nih.gov/gene/?term=10974)(22.373)[GHRH](http://www.ncbi.nlm.nih.gov/gene/?term=2691)(22.373)[HCRT](http://www.ncbi.nlm.nih.gov/gene/?term=3060)(22.373) [RAB3B](http://www.ncbi.nlm.nih.gov/gene/?term=5865)(22.373)[KCNE5](http://www.ncbi.nlm.nih.gov/gene/?term=23630)(22.373)[CNR1](http://www.ncbi.nlm.nih.gov/gene/?term=1268)(22.373)[SLC18A3](http://www.ncbi.nlm.nih.gov/gene/?term=6572)(22.373)[EZR](http://www.ncbi.nlm.nih.gov/gene/?term=7430)(22.373) [DRD4](http://www.ncbi.nlm.nih.gov/gene/?term=1815)(22.373)[CRH](http://www.ncbi.nlm.nih.gov/gene/?term=1392)(22.373)[GLI3](http://www.ncbi.nlm.nih.gov/gene/?term=2737)(22.373)[CAV3](http://www.ncbi.nlm.nih.gov/gene/?term=859)(22.373)[SLC9A6](http://www.ncbi.nlm.nih.gov/gene/?term=10479)(22.373) [AOC3](http://www.ncbi.nlm.nih.gov/gene/?term=8639)(22.373)[NCOA1](http://www.ncbi.nlm.nih.gov/gene/?term=8648)(22.373)[AOC2](http://www.ncbi.nlm.nih.gov/gene/?term=314)(22.373)[IL6](http://www.ncbi.nlm.nih.gov/gene/?term=3569)(22.373)[GLRA4](http://www.ncbi.nlm.nih.gov/gene/?term=441509)(22.373) [RNF207](http://www.ncbi.nlm.nih.gov/gene/?term=388591)(22.373)[ROCK1](http://www.ncbi.nlm.nih.gov/gene/?term=6093)(22.373)[KCNA5](http://www.ncbi.nlm.nih.gov/gene/?term=3741)(22.373)[TACR2](http://www.ncbi.nlm.nih.gov/gene/?term=6865)(22.373)[NRP1](http://www.ncbi.nlm.nih.gov/gene/?term=8829)(22.373) [NPPA](http://www.ncbi.nlm.nih.gov/gene/?term=4878)(22.373)[KMT2A](http://www.ncbi.nlm.nih.gov/gene/?term=4297)(22.373)[TNR](http://www.ncbi.nlm.nih.gov/gene/?term=7143)(22.373)[ZP3](http://www.ncbi.nlm.nih.gov/gene/?term=7784)(22.373)[PLCB1](http://www.ncbi.nlm.nih.gov/gene/?term=23236)(22.373) [EPHB1](http://www.ncbi.nlm.nih.gov/gene/?term=2047)(22.373)[DRD1](http://www.ncbi.nlm.nih.gov/gene/?term=1812)(22.373)[EEA1](http://www.ncbi.nlm.nih.gov/gene/?term=8411)(22.373)[POU4F3](http://www.ncbi.nlm.nih.gov/gene/?term=5459)(22.373)[CALCA](http://www.ncbi.nlm.nih.gov/gene/?term=796)(22.373) [PINK1](http://www.ncbi.nlm.nih.gov/gene/?term=65018)(22.373)[KCNQ1](http://www.ncbi.nlm.nih.gov/gene/?term=3784)(22.373)[ICAM1](http://www.ncbi.nlm.nih.gov/gene/?term=3383)(22.373)[CARTPT](http://www.ncbi.nlm.nih.gov/gene/?term=9607)(22.373)[ATP8B1](http://www.ncbi.nlm.nih.gov/gene/?term=5205)(22.373) [KISS1](http://www.ncbi.nlm.nih.gov/gene/?term=3814)(22.373)[GRM7](http://www.ncbi.nlm.nih.gov/gene/?term=2917)(22.373)[FOS](http://www.ncbi.nlm.nih.gov/gene/?term=2353)(22.373)[GRIN1](http://www.ncbi.nlm.nih.gov/gene/?term=2902)(22.373) | |
| [Ethyl-Alpha-D-Fructofuranoside](http://www.megabionet.org/tcmid/ingredient/23457/) | [NFKB2](http://www.ncbi.nlm.nih.gov/gene/?term=4791)(48.000)[TNF](http://www.ncbi.nlm.nih.gov/gene/?term=7124)(48.000)[MMP9](http://www.ncbi.nlm.nih.gov/gene/?term=4318)(48.000)[IFNG](http://www.ncbi.nlm.nih.gov/gene/?term=3458)(48.000) | |
| [Fritillaziebinol](http://www.megabionet.org/tcmid/ingredient/7957/) | This compound doesn't have any potential target with score larger than 20. | |
| [Neouralenol](http://www.megabionet.org/tcmid/ingredient/15462/) | This compound doesn't have any potential target with score larger than 20. | |
| [Neowilforine](http://www.megabionet.org/tcmid/ingredient/15467/) | This compound doesn't have any potential target with score larger than 20. | |
| [Glyyunnanprosapogenin D](http://www.megabionet.org/tcmid/ingredient/8855/) | [GLRA3](http://www.ncbi.nlm.nih.gov/gene/?term=8001)(48.000)[NR3C1](http://www.ncbi.nlm.nih.gov/gene/?term=2908)(48.000)[GABRB3](http://www.ncbi.nlm.nih.gov/gene/?term=2562)(48.000) | |
| [Licoflavone](http://www.megabionet.org/tcmid/ingredient/31429/) | This compound doesn't have any potential target with score larger than 20. | |
| [Soyasaponin 1](http://www.megabionet.org/tcmid/ingredient/24621/) | [NR3C1](http://www.ncbi.nlm.nih.gov/gene/?term=2908)(48.000)[ATP1A1](http://www.ncbi.nlm.nih.gov/gene/?term=476)(48.000) | |
| [Uralenneoside](http://www.megabionet.org/tcmid/ingredient/22224/) | This compound doesn't have any potential target with score larger than 20. | |
| [Cycloastragenol](http://www.megabionet.org/tcmid/ingredient/4475/) | This compound doesn't have any potential target with score larger than 20. | |
| [Licobenzofuran](http://www.megabionet.org/tcmid/ingredient/12758/) | [CNR2](http://www.ncbi.nlm.nih.gov/gene/?term=1269)(80.882)[CNR1](http://www.ncbi.nlm.nih.gov/gene/?term=1268)(80.882)[DRD2](http://www.ncbi.nlm.nih.gov/gene/?term=1813)(55.444)[SEC14L3](http://www.ncbi.nlm.nih.gov/gene/?term=266629)(23.000)[PPP2CA](http://www.ncbi.nlm.nih.gov/gene/?term=5515)(23.000) [PRKCA](http://www.ncbi.nlm.nih.gov/gene/?term=5578)(23.000)[NR1I2](http://www.ncbi.nlm.nih.gov/gene/?term=8856)(23.000)[ALOX5](http://www.ncbi.nlm.nih.gov/gene/?term=240)(23.000)[PPP2CB](http://www.ncbi.nlm.nih.gov/gene/?term=5516)(23.000)[SEC14L2](http://www.ncbi.nlm.nih.gov/gene/?term=23541)(23.000) [DGKA](http://www.ncbi.nlm.nih.gov/gene/?term=1606)(23.000)[PRKCB](http://www.ncbi.nlm.nih.gov/gene/?term=5579)(23.000)[SEC14L4](http://www.ncbi.nlm.nih.gov/gene/?term=284904)(23.000)[AKT1](http://www.ncbi.nlm.nih.gov/gene/?term=207)(22.373)[CCR7](http://www.ncbi.nlm.nih.gov/gene/?term=1236)(22.373) [ABHD6](http://www.ncbi.nlm.nih.gov/gene/?term=57406)(22.373)[MGLL](http://www.ncbi.nlm.nih.gov/gene/?term=11343)(22.373)[FCER1G](http://www.ncbi.nlm.nih.gov/gene/?term=2207)(22.373)[SUMO1](http://www.ncbi.nlm.nih.gov/gene/?term=7341)(22.373)[GPR55](http://www.ncbi.nlm.nih.gov/gene/?term=9290)(22.373) [CHRNB2](http://www.ncbi.nlm.nih.gov/gene/?term=1141)(22.373)[FCER1A](http://www.ncbi.nlm.nih.gov/gene/?term=2205)(22.373)[CAV3](http://www.ncbi.nlm.nih.gov/gene/?term=859)(22.373)[RNF207](http://www.ncbi.nlm.nih.gov/gene/?term=388591)(22.373)[C3](http://www.ncbi.nlm.nih.gov/gene/?term=718)(22.373) [DAGLA](http://www.ncbi.nlm.nih.gov/gene/?term=747)(22.373)[PLIN5](http://www.ncbi.nlm.nih.gov/gene/?term=440503)(22.373)[ZP3](http://www.ncbi.nlm.nih.gov/gene/?term=7784)(22.373) | |
| [Isoorientin](http://www.megabionet.org/tcmid/ingredient/23134/) | This compound doesn't have any potential target with score larger than 20. | |
| [3,4-Dicaffeoyl-5-(3-Hydroxy-3-Methyl) Glutaroyl Quinic Acid](http://www.megabionet.org/tcmid/ingredient/30901/) | This compound doesn't have any potential target with score larger than 20. | |
| [20-Hexadecanoylingenol](http://www.megabionet.org/tcmid/ingredient/9488/) | [PRKCA](http://www.ncbi.nlm.nih.gov/gene/?term=5578)(122.778)[PRKCD](http://www.ncbi.nlm.nih.gov/gene/?term=5580)(122.778)[PTGER4](http://www.ncbi.nlm.nih.gov/gene/?term=5734)(23.000)[PTGER2](http://www.ncbi.nlm.nih.gov/gene/?term=5732)(23.000)[PTGER3](http://www.ncbi.nlm.nih.gov/gene/?term=5733)(23.000) [CD300A](http://www.ncbi.nlm.nih.gov/gene/?term=11314)(22.373)[CHGA](http://www.ncbi.nlm.nih.gov/gene/?term=1113)(22.373)[APOC2](http://www.ncbi.nlm.nih.gov/gene/?term=344)(22.373)[KIF14](http://www.ncbi.nlm.nih.gov/gene/?term=9928)(22.373)[PRKCB](http://www.ncbi.nlm.nih.gov/gene/?term=5579)(22.373) | |
| [Stearic Acid](http://www.megabionet.org/tcmid/ingredient/23171/) | [AKR1D1](http://www.ncbi.nlm.nih.gov/gene/?term=6718)(686.000)[TYR](http://www.ncbi.nlm.nih.gov/gene/?term=7299)(686.000)[SRD5A2](http://www.ncbi.nlm.nih.gov/gene/?term=6716)(686.000)[SCN11A](http://www.ncbi.nlm.nih.gov/gene/?term=11280)(122.778)[SCN2B](http://www.ncbi.nlm.nih.gov/gene/?term=6327)(122.778) [ABAT](http://www.ncbi.nlm.nih.gov/gene/?term=18)(122.778)[SCN1A](http://www.ncbi.nlm.nih.gov/gene/?term=6323)(122.778)[ESRRG](http://www.ncbi.nlm.nih.gov/gene/?term=2104)(122.778)[SCN3B](http://www.ncbi.nlm.nih.gov/gene/?term=55800)(122.778)[COX6C](http://www.ncbi.nlm.nih.gov/gene/?term=1345)(122.778) [COX5B](http://www.ncbi.nlm.nih.gov/gene/?term=1329)(122.778)[COX7C](http://www.ncbi.nlm.nih.gov/gene/?term=1350)(122.778)[COX1](http://www.ncbi.nlm.nih.gov/gene/?term=4512)(122.778)[SCN3A](http://www.ncbi.nlm.nih.gov/gene/?term=6328)(122.778)[AKR1C2](http://www.ncbi.nlm.nih.gov/gene/?term=1646)(122.778) [SCN7A](http://www.ncbi.nlm.nih.gov/gene/?term=6332)(122.778)[SCN5A](http://www.ncbi.nlm.nih.gov/gene/?term=6331)(122.778)[SCN10A](http://www.ncbi.nlm.nih.gov/gene/?term=6336)(122.778)[ALDH5A1](http://www.ncbi.nlm.nih.gov/gene/?term=7915)(122.778)[SCN2A](http://www.ncbi.nlm.nih.gov/gene/?term=6326)(122.778) [COX5A](http://www.ncbi.nlm.nih.gov/gene/?term=9377)(122.778)[COX3](http://www.ncbi.nlm.nih.gov/gene/?term=4514)(122.778)[AR](http://www.ncbi.nlm.nih.gov/gene/?term=367)(122.778)[COX7A1](http://www.ncbi.nlm.nih.gov/gene/?term=1346)(122.778)[FECH](http://www.ncbi.nlm.nih.gov/gene/?term=2235)(122.778) [COX4I1](http://www.ncbi.nlm.nih.gov/gene/?term=1327)(122.778)[HDAC9](http://www.ncbi.nlm.nih.gov/gene/?term=9734)(122.778)[PLA2G1B](http://www.ncbi.nlm.nih.gov/gene/?term=5319)(122.778)[COX6A2](http://www.ncbi.nlm.nih.gov/gene/?term=1339)(122.778)[SCN9A](http://www.ncbi.nlm.nih.gov/gene/?term=6335)(122.778) [SCN4A](http://www.ncbi.nlm.nih.gov/gene/?term=6329)(122.778)[SCN4B](http://www.ncbi.nlm.nih.gov/gene/?term=6330)(122.778)[ADH1C](http://www.ncbi.nlm.nih.gov/gene/?term=126)(122.778)[COX6B1](http://www.ncbi.nlm.nih.gov/gene/?term=1340)(122.778)[FABP6](http://www.ncbi.nlm.nih.gov/gene/?term=2172)(122.778) [CES1](http://www.ncbi.nlm.nih.gov/gene/?term=1066)(122.778)[ACADSB](http://www.ncbi.nlm.nih.gov/gene/?term=36)(122.778)[SCN8A](http://www.ncbi.nlm.nih.gov/gene/?term=6334)(122.778)[COX7B](http://www.ncbi.nlm.nih.gov/gene/?term=1349)(122.778)[SCN1B](http://www.ncbi.nlm.nih.gov/gene/?term=6324)(122.778) [COX2](http://www.ncbi.nlm.nih.gov/gene/?term=4513)(122.778)[OGDH](http://www.ncbi.nlm.nih.gov/gene/?term=4967)(122.778)[HDAC2](http://www.ncbi.nlm.nih.gov/gene/?term=3066)(122.778)[COX8A](http://www.ncbi.nlm.nih.gov/gene/?term=1351)(122.778)[NR1H4](http://www.ncbi.nlm.nih.gov/gene/?term=9971)(122.778) [SUCLG2](http://www.ncbi.nlm.nih.gov/gene/?term=8801)(80.882)[SLC13A2](http://www.ncbi.nlm.nih.gov/gene/?term=9058)(80.882)[SLC13A1](http://www.ncbi.nlm.nih.gov/gene/?term=6561)(80.882)[SUCLG1](http://www.ncbi.nlm.nih.gov/gene/?term=8802)(80.882)[HSD17B6](http://www.ncbi.nlm.nih.gov/gene/?term=8630)(80.882) [SLC25A10](http://www.ncbi.nlm.nih.gov/gene/?term=1468)(80.882)[P3H3](http://www.ncbi.nlm.nih.gov/gene/?term=10536)(80.882)[OXCT2](http://www.ncbi.nlm.nih.gov/gene/?term=64064)(80.882)[TMLHE](http://www.ncbi.nlm.nih.gov/gene/?term=55217)(80.882)[PLOD1](http://www.ncbi.nlm.nih.gov/gene/?term=5351)(80.882) [SUCNR1](http://www.ncbi.nlm.nih.gov/gene/?term=56670)(80.882)[SLC13A3](http://www.ncbi.nlm.nih.gov/gene/?term=64849)(80.882)[ASPH](http://www.ncbi.nlm.nih.gov/gene/?term=444)(80.882)[BBOX1](http://www.ncbi.nlm.nih.gov/gene/?term=8424)(80.882)[SDHB](http://www.ncbi.nlm.nih.gov/gene/?term=6390)(80.882) [OXCT1](http://www.ncbi.nlm.nih.gov/gene/?term=5019)(80.882)[PLOD3](http://www.ncbi.nlm.nih.gov/gene/?term=8985)(80.882)[P4HA1](http://www.ncbi.nlm.nih.gov/gene/?term=5033)(80.882)[SDHC](http://www.ncbi.nlm.nih.gov/gene/?term=6391)(80.882)[P3H2](http://www.ncbi.nlm.nih.gov/gene/?term=55214)(80.882) [SDHA](http://www.ncbi.nlm.nih.gov/gene/?term=6389)(80.882)[P4HA2](http://www.ncbi.nlm.nih.gov/gene/?term=8974)(80.882)[SUCLA2](http://www.ncbi.nlm.nih.gov/gene/?term=8803)(80.882)[SDHD](http://www.ncbi.nlm.nih.gov/gene/?term=6392)(80.882)[P3H1](http://www.ncbi.nlm.nih.gov/gene/?term=64175)(80.882) [DCT](http://www.ncbi.nlm.nih.gov/gene/?term=1638)(55.444)[AKR1C3](http://www.ncbi.nlm.nih.gov/gene/?term=8644)(55.444)[CRTAP](http://www.ncbi.nlm.nih.gov/gene/?term=10491)(55.444)[SLIT2](http://www.ncbi.nlm.nih.gov/gene/?term=9353)(55.444)[ACO2](http://www.ncbi.nlm.nih.gov/gene/?term=50)(55.444) [JMJD6](http://www.ncbi.nlm.nih.gov/gene/?term=23210)(55.444)[SLC13A4](http://www.ncbi.nlm.nih.gov/gene/?term=26266)(55.444)[SDHAF2](http://www.ncbi.nlm.nih.gov/gene/?term=54949)(55.444)[SLC1A3](http://www.ncbi.nlm.nih.gov/gene/?term=6507)(55.444)[SALL1](http://www.ncbi.nlm.nih.gov/gene/?term=6299)(55.444) [HSD17B11](http://www.ncbi.nlm.nih.gov/gene/?term=51170)(55.444)[TYRP1](http://www.ncbi.nlm.nih.gov/gene/?term=7306)(55.444)[HIF1AN](http://www.ncbi.nlm.nih.gov/gene/?term=55662)(55.444)[UROS](http://www.ncbi.nlm.nih.gov/gene/?term=7390)(55.444)[SLC13A5](http://www.ncbi.nlm.nih.gov/gene/?term=284111)(55.444) [P4HB](http://www.ncbi.nlm.nih.gov/gene/?term=5034)(55.444)[CACNA2D1](http://www.ncbi.nlm.nih.gov/gene/?term=781)(48.000)[PLAT](http://www.ncbi.nlm.nih.gov/gene/?term=5327)(48.000)[SLC7A2](http://www.ncbi.nlm.nih.gov/gene/?term=6542)(48.000)[CACNA1A](http://www.ncbi.nlm.nih.gov/gene/?term=773)(48.000) [GRIN3B](http://www.ncbi.nlm.nih.gov/gene/?term=116444)(48.000)[CACNA2D2](http://www.ncbi.nlm.nih.gov/gene/?term=9254)(48.000)[GRIN2A](http://www.ncbi.nlm.nih.gov/gene/?term=2903)(48.000)[TOP1](http://www.ncbi.nlm.nih.gov/gene/?term=7150)(48.000)[PLG](http://www.ncbi.nlm.nih.gov/gene/?term=5340)(48.000) [CACNA1B](http://www.ncbi.nlm.nih.gov/gene/?term=774)(48.000)[GRIN2C](http://www.ncbi.nlm.nih.gov/gene/?term=2905)(48.000)[GRIN2B](http://www.ncbi.nlm.nih.gov/gene/?term=2904)(48.000)[SLC7A3](http://www.ncbi.nlm.nih.gov/gene/?term=84889)(48.000)[KARS](http://www.ncbi.nlm.nih.gov/gene/?term=3735)(48.000) [GRIN3A](http://www.ncbi.nlm.nih.gov/gene/?term=116443)(48.000)[SLC7A1](http://www.ncbi.nlm.nih.gov/gene/?term=6541)(48.000)[SLC7A4](http://www.ncbi.nlm.nih.gov/gene/?term=6545)(48.000)[GRIN2D](http://www.ncbi.nlm.nih.gov/gene/?term=2906)(48.000)[ADORA1](http://www.ncbi.nlm.nih.gov/gene/?term=134)(48.000) [GRIN1](http://www.ncbi.nlm.nih.gov/gene/?term=2902)(48.000)[HDAC1](http://www.ncbi.nlm.nih.gov/gene/?term=3065)(23.000)[GPD1L](http://www.ncbi.nlm.nih.gov/gene/?term=23171)(22.373)[SRD5A1](http://www.ncbi.nlm.nih.gov/gene/?term=6715)(22.373)[FBXO45](http://www.ncbi.nlm.nih.gov/gene/?term=200933)(22.373) [SIRT1](http://www.ncbi.nlm.nih.gov/gene/?term=23411)(22.373)[SHMT1](http://www.ncbi.nlm.nih.gov/gene/?term=6470)(22.373)[SUGCT](http://www.ncbi.nlm.nih.gov/gene/?term=79783)(22.373)[ACSS2](http://www.ncbi.nlm.nih.gov/gene/?term=55902)(22.373)[ARV1](http://www.ncbi.nlm.nih.gov/gene/?term=64801)(22.373) [SIX1](http://www.ncbi.nlm.nih.gov/gene/?term=6495)(22.373)[BDH1](http://www.ncbi.nlm.nih.gov/gene/?term=622)(22.373)[COLGALT2](http://www.ncbi.nlm.nih.gov/gene/?term=23127)(22.373)[SIX4](http://www.ncbi.nlm.nih.gov/gene/?term=51804)(22.373)[MRPS36](http://www.ncbi.nlm.nih.gov/gene/?term=92259)(22.373) [OSBPL8](http://www.ncbi.nlm.nih.gov/gene/?term=114882)(22.373)[SPI1](http://www.ncbi.nlm.nih.gov/gene/?term=6688)(22.373)[SUOX](http://www.ncbi.nlm.nih.gov/gene/?term=6821)(22.373)[CCL2](http://www.ncbi.nlm.nih.gov/gene/?term=6347)(22.373)[BCKDK](http://www.ncbi.nlm.nih.gov/gene/?term=10295)(22.373) [UBE2B](http://www.ncbi.nlm.nih.gov/gene/?term=7320)(22.373)[CYP1A2](http://www.ncbi.nlm.nih.gov/gene/?term=1544)(22.373)[CCL5](http://www.ncbi.nlm.nih.gov/gene/?term=6352)(22.373)[ANK2](http://www.ncbi.nlm.nih.gov/gene/?term=287)(22.373)[HSD17B2](http://www.ncbi.nlm.nih.gov/gene/?term=3294)(22.373) [STIM2](http://www.ncbi.nlm.nih.gov/gene/?term=57620)(22.373)[PKD2](http://www.ncbi.nlm.nih.gov/gene/?term=5311)(22.373)[ALDH9A1](http://www.ncbi.nlm.nih.gov/gene/?term=223)(22.373)[TH](http://www.ncbi.nlm.nih.gov/gene/?term=7054)(22.373)[EPO](http://www.ncbi.nlm.nih.gov/gene/?term=2056)(22.373) [COLGALT1](http://www.ncbi.nlm.nih.gov/gene/?term=79709)(22.373)[NEDD4](http://www.ncbi.nlm.nih.gov/gene/?term=4734)(22.373)[CYP11A1](http://www.ncbi.nlm.nih.gov/gene/?term=1583)(22.373)[DHODH](http://www.ncbi.nlm.nih.gov/gene/?term=1723)(22.373)[CLN3](http://www.ncbi.nlm.nih.gov/gene/?term=1201)(22.373) [ARID1A](http://www.ncbi.nlm.nih.gov/gene/?term=8289)(22.373)[EGLN2](http://www.ncbi.nlm.nih.gov/gene/?term=112398)(22.373)[ACAT1](http://www.ncbi.nlm.nih.gov/gene/?term=38)(22.373)[GALC](http://www.ncbi.nlm.nih.gov/gene/?term=2581)(22.373)[PLCG2](http://www.ncbi.nlm.nih.gov/gene/?term=5336)(22.373) [ACOT4](http://www.ncbi.nlm.nih.gov/gene/?term=122970)(22.373)[PHGDH](http://www.ncbi.nlm.nih.gov/gene/?term=26227)(22.373)[PNLIPRP2](http://www.ncbi.nlm.nih.gov/gene/?term=5408)(22.373)[CYP39A1](http://www.ncbi.nlm.nih.gov/gene/?term=51302)(22.373)[BCKDHA](http://www.ncbi.nlm.nih.gov/gene/?term=593)(22.373) [SLC25A12](http://www.ncbi.nlm.nih.gov/gene/?term=8604)(22.373)[YBX3](http://www.ncbi.nlm.nih.gov/gene/?term=8531)(22.373)[8-Mar](http://www.ncbi.nlm.nih.gov/gene/?term=220972)(22.373)[STIM1](http://www.ncbi.nlm.nih.gov/gene/?term=6786)(22.373)[GAL3ST1](http://www.ncbi.nlm.nih.gov/gene/?term=9514)(22.373) [CDH11](http://www.ncbi.nlm.nih.gov/gene/?term=1009)(22.373)[CACNA1D](http://www.ncbi.nlm.nih.gov/gene/?term=776)(22.373)[DDC](http://www.ncbi.nlm.nih.gov/gene/?term=1644)(22.373)[DHTKD1](http://www.ncbi.nlm.nih.gov/gene/?term=55526)(22.373)[NEDD4L](http://www.ncbi.nlm.nih.gov/gene/?term=23327)(22.373) [BDKRB2](http://www.ncbi.nlm.nih.gov/gene/?term=624)(22.373)[PRPH](http://www.ncbi.nlm.nih.gov/gene/?term=5630)(22.373)[BCKDHB](http://www.ncbi.nlm.nih.gov/gene/?term=594)(22.373)[KCNE5](http://www.ncbi.nlm.nih.gov/gene/?term=23630)(22.373)[SRD5A3](http://www.ncbi.nlm.nih.gov/gene/?term=79644)(22.373) [SMAD7](http://www.ncbi.nlm.nih.gov/gene/?term=4092)(22.373)[PRDM8](http://www.ncbi.nlm.nih.gov/gene/?term=56978)(22.373)[UROD](http://www.ncbi.nlm.nih.gov/gene/?term=7389)(22.373)[UGT8](http://www.ncbi.nlm.nih.gov/gene/?term=7368)(22.373)[ETHE1](http://www.ncbi.nlm.nih.gov/gene/?term=23474)(22.373) [WNT10B](http://www.ncbi.nlm.nih.gov/gene/?term=7480)(22.373)[OGFOD1](http://www.ncbi.nlm.nih.gov/gene/?term=55239)(22.373)[SLC38A7](http://www.ncbi.nlm.nih.gov/gene/?term=55238)(22.373)[ACSS1](http://www.ncbi.nlm.nih.gov/gene/?term=84532)(22.373)[CYP4B1](http://www.ncbi.nlm.nih.gov/gene/?term=1580)(22.373) [STAR](http://www.ncbi.nlm.nih.gov/gene/?term=6770)(22.373)[DLST](http://www.ncbi.nlm.nih.gov/gene/?term=1743)(22.373)[PF4](http://www.ncbi.nlm.nih.gov/gene/?term=5196)(22.373)[CRACR2A](http://www.ncbi.nlm.nih.gov/gene/?term=84766)(22.373)[HSD17B1](http://www.ncbi.nlm.nih.gov/gene/?term=3292)(22.373) [CAMK2D](http://www.ncbi.nlm.nih.gov/gene/?term=817)(22.373)[AP3D1](http://www.ncbi.nlm.nih.gov/gene/?term=8943)(22.373)[MYO5A](http://www.ncbi.nlm.nih.gov/gene/?term=4644)(22.373)[ILVBL](http://www.ncbi.nlm.nih.gov/gene/?term=10994)(22.373)[CAV3](http://www.ncbi.nlm.nih.gov/gene/?term=859)(22.373) [HTT](http://www.ncbi.nlm.nih.gov/gene/?term=3064)(22.373)[IL10](http://www.ncbi.nlm.nih.gov/gene/?term=3586)(22.373)[EGLN3](http://www.ncbi.nlm.nih.gov/gene/?term=112399)(22.373)[OGDHL](http://www.ncbi.nlm.nih.gov/gene/?term=55753)(22.373)[KCNA5](http://www.ncbi.nlm.nih.gov/gene/?term=3741)(22.373) [TST](http://www.ncbi.nlm.nih.gov/gene/?term=7263)(22.373)[SLC1A6](http://www.ncbi.nlm.nih.gov/gene/?term=6511)(22.373)[NPPA](http://www.ncbi.nlm.nih.gov/gene/?term=4878)(22.373)[ACADM](http://www.ncbi.nlm.nih.gov/gene/?term=34)(22.373)[SQRDL](http://www.ncbi.nlm.nih.gov/gene/?term=58472)(22.373) [TMEM110](http://www.ncbi.nlm.nih.gov/gene/?term=375346)(22.373)[CDC42](http://www.ncbi.nlm.nih.gov/gene/?term=998)(22.373)[ARHGEF2](http://www.ncbi.nlm.nih.gov/gene/?term=9181)(22.373)[EGLN1](http://www.ncbi.nlm.nih.gov/gene/?term=54583)(22.373)[ETFDH](http://www.ncbi.nlm.nih.gov/gene/?term=2110)(22.373) [EPHA4](http://www.ncbi.nlm.nih.gov/gene/?term=2043)(22.373)[OCA2](http://www.ncbi.nlm.nih.gov/gene/?term=4948)(22.373)[HSD17B8](http://www.ncbi.nlm.nih.gov/gene/?term=7923)(22.373)[CTNNB1](http://www.ncbi.nlm.nih.gov/gene/?term=1499)(22.373)[P4HA3](http://www.ncbi.nlm.nih.gov/gene/?term=283208)(22.373) [EDA](http://www.ncbi.nlm.nih.gov/gene/?term=1896)(22.373)[HACL1](http://www.ncbi.nlm.nih.gov/gene/?term=26061)(22.373)[RYR2](http://www.ncbi.nlm.nih.gov/gene/?term=6262)(22.373)[HAP1](http://www.ncbi.nlm.nih.gov/gene/?term=9001)(22.373)[PCSK9](http://www.ncbi.nlm.nih.gov/gene/?term=255738)(22.373) [KCNQ1](http://www.ncbi.nlm.nih.gov/gene/?term=3784)(22.373)[CASQ2](http://www.ncbi.nlm.nih.gov/gene/?term=845)(22.373)[GJA5](http://www.ncbi.nlm.nih.gov/gene/?term=2702)(22.373)[DHRS9](http://www.ncbi.nlm.nih.gov/gene/?term=10170)(22.373)[GPR143](http://www.ncbi.nlm.nih.gov/gene/?term=4935)(22.373) [UGT1A1](http://www.ncbi.nlm.nih.gov/gene/?term=54658)(22.373)[DLD](http://www.ncbi.nlm.nih.gov/gene/?term=1738)(22.373)[GNAS](http://www.ncbi.nlm.nih.gov/gene/?term=2778)(22.373)[SLC25A13](http://www.ncbi.nlm.nih.gov/gene/?term=10165)(22.373)[SFXN5](http://www.ncbi.nlm.nih.gov/gene/?term=94097)(22.373) [PLOD2](http://www.ncbi.nlm.nih.gov/gene/?term=5352)(22.373)[CYP1A1](http://www.ncbi.nlm.nih.gov/gene/?term=1543)(22.373)[SULT2A1](http://www.ncbi.nlm.nih.gov/gene/?term=6822)(22.373)[NFX1](http://www.ncbi.nlm.nih.gov/gene/?term=4799)(22.373)[DBT](http://www.ncbi.nlm.nih.gov/gene/?term=1629)(22.373) [SLC25A1](http://www.ncbi.nlm.nih.gov/gene/?term=6576)(22.373) | |
| [Methyl Octadecanoate](http://www.megabionet.org/tcmid/ingredient/14621/) | [NPR1](http://www.ncbi.nlm.nih.gov/gene/?term=4881)(80.882)[SCN11A](http://www.ncbi.nlm.nih.gov/gene/?term=11280)(48.000)[SCN2B](http://www.ncbi.nlm.nih.gov/gene/?term=6327)(48.000)[ABAT](http://www.ncbi.nlm.nih.gov/gene/?term=18)(48.000)[SCN1A](http://www.ncbi.nlm.nih.gov/gene/?term=6323)(48.000) [ESRRG](http://www.ncbi.nlm.nih.gov/gene/?term=2104)(48.000)[SCN3B](http://www.ncbi.nlm.nih.gov/gene/?term=55800)(48.000)[COX6C](http://www.ncbi.nlm.nih.gov/gene/?term=1345)(48.000)[COX5B](http://www.ncbi.nlm.nih.gov/gene/?term=1329)(48.000)[COX7C](http://www.ncbi.nlm.nih.gov/gene/?term=1350)(48.000) [COX1](http://www.ncbi.nlm.nih.gov/gene/?term=4512)(48.000)[SCN3A](http://www.ncbi.nlm.nih.gov/gene/?term=6328)(48.000)[AKR1C2](http://www.ncbi.nlm.nih.gov/gene/?term=1646)(48.000)[SCN7A](http://www.ncbi.nlm.nih.gov/gene/?term=6332)(48.000)[SCN5A](http://www.ncbi.nlm.nih.gov/gene/?term=6331)(48.000) [SCN10A](http://www.ncbi.nlm.nih.gov/gene/?term=6336)(48.000)[AKR1D1](http://www.ncbi.nlm.nih.gov/gene/?term=6718)(48.000)[ALDH5A1](http://www.ncbi.nlm.nih.gov/gene/?term=7915)(48.000)[SCN2A](http://www.ncbi.nlm.nih.gov/gene/?term=6326)(48.000)[COX5A](http://www.ncbi.nlm.nih.gov/gene/?term=9377)(48.000) [COX3](http://www.ncbi.nlm.nih.gov/gene/?term=4514)(48.000)[AR](http://www.ncbi.nlm.nih.gov/gene/?term=367)(48.000)[COX7A1](http://www.ncbi.nlm.nih.gov/gene/?term=1346)(48.000)[FECH](http://www.ncbi.nlm.nih.gov/gene/?term=2235)(48.000)[COX4I1](http://www.ncbi.nlm.nih.gov/gene/?term=1327)(48.000) [HDAC9](http://www.ncbi.nlm.nih.gov/gene/?term=9734)(48.000)[PLA2G1B](http://www.ncbi.nlm.nih.gov/gene/?term=5319)(48.000)[COX6A2](http://www.ncbi.nlm.nih.gov/gene/?term=1339)(48.000)[TYR](http://www.ncbi.nlm.nih.gov/gene/?term=7299)(48.000)[SCN9A](http://www.ncbi.nlm.nih.gov/gene/?term=6335)(48.000) [SCN4A](http://www.ncbi.nlm.nih.gov/gene/?term=6329)(48.000)[SCN4B](http://www.ncbi.nlm.nih.gov/gene/?term=6330)(48.000)[ADH1C](http://www.ncbi.nlm.nih.gov/gene/?term=126)(48.000)[COX6B1](http://www.ncbi.nlm.nih.gov/gene/?term=1340)(48.000)[FABP6](http://www.ncbi.nlm.nih.gov/gene/?term=2172)(48.000) [CES1](http://www.ncbi.nlm.nih.gov/gene/?term=1066)(48.000)[SRD5A2](http://www.ncbi.nlm.nih.gov/gene/?term=6716)(48.000)[ACADSB](http://www.ncbi.nlm.nih.gov/gene/?term=36)(48.000)[SCN8A](http://www.ncbi.nlm.nih.gov/gene/?term=6334)(48.000)[COX7B](http://www.ncbi.nlm.nih.gov/gene/?term=1349)(48.000) [SCN1B](http://www.ncbi.nlm.nih.gov/gene/?term=6324)(48.000)[COX2](http://www.ncbi.nlm.nih.gov/gene/?term=4513)(48.000)[OGDH](http://www.ncbi.nlm.nih.gov/gene/?term=4967)(48.000)[HDAC2](http://www.ncbi.nlm.nih.gov/gene/?term=3066)(48.000)[COX8A](http://www.ncbi.nlm.nih.gov/gene/?term=1351)(48.000) [NR1H4](http://www.ncbi.nlm.nih.gov/gene/?term=9971)(48.000)[NPR3](http://www.ncbi.nlm.nih.gov/gene/?term=4883)(22.373)[NPR2](http://www.ncbi.nlm.nih.gov/gene/?term=4882)(22.373) | |
| [Isorhamnetin](http://www.megabionet.org/tcmid/ingredient/11645/) | This compound doesn't have any potential target with score larger than 20. | |
| [Phaseollinisoflavan](http://www.megabionet.org/tcmid/ingredient/17035/) | [CNR2](http://www.ncbi.nlm.nih.gov/gene/?term=1269)(80.882)[CNR1](http://www.ncbi.nlm.nih.gov/gene/?term=1268)(80.882)[DRD2](http://www.ncbi.nlm.nih.gov/gene/?term=1813)(55.444)[SEC14L3](http://www.ncbi.nlm.nih.gov/gene/?term=266629)(23.000)[PPP2CA](http://www.ncbi.nlm.nih.gov/gene/?term=5515)(23.000) [PRKCA](http://www.ncbi.nlm.nih.gov/gene/?term=5578)(23.000)[NR1I2](http://www.ncbi.nlm.nih.gov/gene/?term=8856)(23.000)[ALOX5](http://www.ncbi.nlm.nih.gov/gene/?term=240)(23.000)[PPP2CB](http://www.ncbi.nlm.nih.gov/gene/?term=5516)(23.000)[SEC14L2](http://www.ncbi.nlm.nih.gov/gene/?term=23541)(23.000) [DGKA](http://www.ncbi.nlm.nih.gov/gene/?term=1606)(23.000)[PRKCB](http://www.ncbi.nlm.nih.gov/gene/?term=5579)(23.000)[SEC14L4](http://www.ncbi.nlm.nih.gov/gene/?term=284904)(23.000)[AKT1](http://www.ncbi.nlm.nih.gov/gene/?term=207)(22.373)[CCR7](http://www.ncbi.nlm.nih.gov/gene/?term=1236)(22.373) [ABHD6](http://www.ncbi.nlm.nih.gov/gene/?term=57406)(22.373)[MGLL](http://www.ncbi.nlm.nih.gov/gene/?term=11343)(22.373)[FCER1G](http://www.ncbi.nlm.nih.gov/gene/?term=2207)(22.373)[SUMO1](http://www.ncbi.nlm.nih.gov/gene/?term=7341)(22.373)[GPR55](http://www.ncbi.nlm.nih.gov/gene/?term=9290)(22.373) [CHRNB2](http://www.ncbi.nlm.nih.gov/gene/?term=1141)(22.373)[FCER1A](http://www.ncbi.nlm.nih.gov/gene/?term=2205)(22.373)[CAV3](http://www.ncbi.nlm.nih.gov/gene/?term=859)(22.373)[RNF207](http://www.ncbi.nlm.nih.gov/gene/?term=388591)(22.373)[C3](http://www.ncbi.nlm.nih.gov/gene/?term=718)(22.373) [DAGLA](http://www.ncbi.nlm.nih.gov/gene/?term=747)(22.373)[PLIN5](http://www.ncbi.nlm.nih.gov/gene/?term=440503)(22.373)[ZP3](http://www.ncbi.nlm.nih.gov/gene/?term=7784)(22.373) | |
| [Licoricesaponine D3](http://www.megabionet.org/tcmid/ingredient/31434/) | [GLRA3](http://www.ncbi.nlm.nih.gov/gene/?term=8001)(48.000)[NR3C1](http://www.ncbi.nlm.nih.gov/gene/?term=2908)(48.000)[ATP1A1](http://www.ncbi.nlm.nih.gov/gene/?term=476)(48.000)[GABRB3](http://www.ncbi.nlm.nih.gov/gene/?term=2562)(48.000) | |
| [Spinoside A](http://www.megabionet.org/tcmid/ingredient/20155/) | [GLRA3](http://www.ncbi.nlm.nih.gov/gene/?term=8001)(48.000)[NR3C1](http://www.ncbi.nlm.nih.gov/gene/?term=2908)(48.000)[ATP1A1](http://www.ncbi.nlm.nih.gov/gene/?term=476)(48.000)[GABRB3](http://www.ncbi.nlm.nih.gov/gene/?term=2562)(48.000) | |
| [Ferulic Acid](http://www.megabionet.org/tcmid/ingredient/23696/) | This compound doesn't have any potential target with score larger than 20. | |
| [Perlolyrine](http://www.megabionet.org/tcmid/ingredient/16955/) | [SLC19A2](http://www.ncbi.nlm.nih.gov/gene/?term=10560)(23.000)[TPK1](http://www.ncbi.nlm.nih.gov/gene/?term=27010)(23.000) | |
| [Tartaric Acid](http://www.megabionet.org/tcmid/ingredient/23784/) | [SUCLG2](http://www.ncbi.nlm.nih.gov/gene/?term=8801)(48.000)[SLC13A2](http://www.ncbi.nlm.nih.gov/gene/?term=9058)(48.000)[SLC13A1](http://www.ncbi.nlm.nih.gov/gene/?term=6561)(48.000)[SUCLG1](http://www.ncbi.nlm.nih.gov/gene/?term=8802)(48.000)[HSD17B6](http://www.ncbi.nlm.nih.gov/gene/?term=8630)(48.000) [GABRB1](http://www.ncbi.nlm.nih.gov/gene/?term=2560)(48.000)[SLC25A10](http://www.ncbi.nlm.nih.gov/gene/?term=1468)(48.000)[P3H3](http://www.ncbi.nlm.nih.gov/gene/?term=10536)(48.000)[OXCT2](http://www.ncbi.nlm.nih.gov/gene/?term=64064)(48.000)[TMLHE](http://www.ncbi.nlm.nih.gov/gene/?term=55217)(48.000) [PLOD1](http://www.ncbi.nlm.nih.gov/gene/?term=5351)(48.000)[SLC52A2](http://www.ncbi.nlm.nih.gov/gene/?term=79581)(48.000)[SUCNR1](http://www.ncbi.nlm.nih.gov/gene/?term=56670)(48.000)[SLC13A3](http://www.ncbi.nlm.nih.gov/gene/?term=64849)(48.000)[ALDH5A1](http://www.ncbi.nlm.nih.gov/gene/?term=7915)(48.000) [TOP1](http://www.ncbi.nlm.nih.gov/gene/?term=7150)(48.000)[ASPH](http://www.ncbi.nlm.nih.gov/gene/?term=444)(48.000)[BBOX1](http://www.ncbi.nlm.nih.gov/gene/?term=8424)(48.000)[SDHB](http://www.ncbi.nlm.nih.gov/gene/?term=6390)(48.000)[OXCT1](http://www.ncbi.nlm.nih.gov/gene/?term=5019)(48.000) [PLOD3](http://www.ncbi.nlm.nih.gov/gene/?term=8985)(48.000)[P4HA1](http://www.ncbi.nlm.nih.gov/gene/?term=5033)(48.000)[SDHC](http://www.ncbi.nlm.nih.gov/gene/?term=6391)(48.000)[P3H2](http://www.ncbi.nlm.nih.gov/gene/?term=55214)(48.000)[SDHA](http://www.ncbi.nlm.nih.gov/gene/?term=6389)(48.000) [P4HA2](http://www.ncbi.nlm.nih.gov/gene/?term=8974)(48.000)[SUCLA2](http://www.ncbi.nlm.nih.gov/gene/?term=8803)(48.000)[SDHD](http://www.ncbi.nlm.nih.gov/gene/?term=6392)(48.000)[P3H1](http://www.ncbi.nlm.nih.gov/gene/?term=64175)(48.000) | |
| [Licoricesaponin C2](http://www.megabionet.org/tcmid/ingredient/12779/) | [GLRA3](http://www.ncbi.nlm.nih.gov/gene/?term=8001)(48.000)[NR3C1](http://www.ncbi.nlm.nih.gov/gene/?term=2908)(48.000)[GABRB3](http://www.ncbi.nlm.nih.gov/gene/?term=2562)(48.000) | |
| [Ethyl-N-Buthy-Uralsaponin A Esters](http://www.megabionet.org/tcmid/ingredient/7421/) | [ESR1](http://www.ncbi.nlm.nih.gov/gene/?term=2099)(48.000)[PGR](http://www.ncbi.nlm.nih.gov/gene/?term=5241)(48.000)[NR3C1](http://www.ncbi.nlm.nih.gov/gene/?term=2908)(48.000)[ATP1A1](http://www.ncbi.nlm.nih.gov/gene/?term=476)(48.000)[ANXA1](http://www.ncbi.nlm.nih.gov/gene/?term=301)(48.000) | |
| [Methyl Pentadecanoate](http://www.megabionet.org/tcmid/ingredient/14647/) | [AKR1D1](http://www.ncbi.nlm.nih.gov/gene/?term=6718)(686.000)[TYR](http://www.ncbi.nlm.nih.gov/gene/?term=7299)(686.000)[SRD5A2](http://www.ncbi.nlm.nih.gov/gene/?term=6716)(686.000)[SCN11A](http://www.ncbi.nlm.nih.gov/gene/?term=11280)(122.778)[SCN2B](http://www.ncbi.nlm.nih.gov/gene/?term=6327)(122.778) [ABAT](http://www.ncbi.nlm.nih.gov/gene/?term=18)(122.778)[SCN1A](http://www.ncbi.nlm.nih.gov/gene/?term=6323)(122.778)[ESRRG](http://www.ncbi.nlm.nih.gov/gene/?term=2104)(122.778)[SCN3B](http://www.ncbi.nlm.nih.gov/gene/?term=55800)(122.778)[COX6C](http://www.ncbi.nlm.nih.gov/gene/?term=1345)(122.778) [COX5B](http://www.ncbi.nlm.nih.gov/gene/?term=1329)(122.778)[COX7C](http://www.ncbi.nlm.nih.gov/gene/?term=1350)(122.778)[COX1](http://www.ncbi.nlm.nih.gov/gene/?term=4512)(122.778)[SCN3A](http://www.ncbi.nlm.nih.gov/gene/?term=6328)(122.778)[AKR1C2](http://www.ncbi.nlm.nih.gov/gene/?term=1646)(122.778) [SCN7A](http://www.ncbi.nlm.nih.gov/gene/?term=6332)(122.778)[SCN5A](http://www.ncbi.nlm.nih.gov/gene/?term=6331)(122.778)[SCN10A](http://www.ncbi.nlm.nih.gov/gene/?term=6336)(122.778)[ALDH5A1](http://www.ncbi.nlm.nih.gov/gene/?term=7915)(122.778)[SCN2A](http://www.ncbi.nlm.nih.gov/gene/?term=6326)(122.778) [COX5A](http://www.ncbi.nlm.nih.gov/gene/?term=9377)(122.778)[COX3](http://www.ncbi.nlm.nih.gov/gene/?term=4514)(122.778)[AR](http://www.ncbi.nlm.nih.gov/gene/?term=367)(122.778)[COX7A1](http://www.ncbi.nlm.nih.gov/gene/?term=1346)(122.778)[FECH](http://www.ncbi.nlm.nih.gov/gene/?term=2235)(122.778) [COX4I1](http://www.ncbi.nlm.nih.gov/gene/?term=1327)(122.778)[HDAC9](http://www.ncbi.nlm.nih.gov/gene/?term=9734)(122.778)[PLA2G1B](http://www.ncbi.nlm.nih.gov/gene/?term=5319)(122.778)[COX6A2](http://www.ncbi.nlm.nih.gov/gene/?term=1339)(122.778)[SCN9A](http://www.ncbi.nlm.nih.gov/gene/?term=6335)(122.778) [SCN4A](http://www.ncbi.nlm.nih.gov/gene/?term=6329)(122.778)[SCN4B](http://www.ncbi.nlm.nih.gov/gene/?term=6330)(122.778)[ADH1C](http://www.ncbi.nlm.nih.gov/gene/?term=126)(122.778)[COX6B1](http://www.ncbi.nlm.nih.gov/gene/?term=1340)(122.778)[FABP6](http://www.ncbi.nlm.nih.gov/gene/?term=2172)(122.778) [CES1](http://www.ncbi.nlm.nih.gov/gene/?term=1066)(122.778)[ACADSB](http://www.ncbi.nlm.nih.gov/gene/?term=36)(122.778)[SCN8A](http://www.ncbi.nlm.nih.gov/gene/?term=6334)(122.778)[COX7B](http://www.ncbi.nlm.nih.gov/gene/?term=1349)(122.778)[SCN1B](http://www.ncbi.nlm.nih.gov/gene/?term=6324)(122.778) [COX2](http://www.ncbi.nlm.nih.gov/gene/?term=4513)(122.778)[OGDH](http://www.ncbi.nlm.nih.gov/gene/?term=4967)(122.778)[HDAC2](http://www.ncbi.nlm.nih.gov/gene/?term=3066)(122.778)[COX8A](http://www.ncbi.nlm.nih.gov/gene/?term=1351)(122.778)[NR1H4](http://www.ncbi.nlm.nih.gov/gene/?term=9971)(122.778) [SUCLG2](http://www.ncbi.nlm.nih.gov/gene/?term=8801)(80.882)[SLC13A2](http://www.ncbi.nlm.nih.gov/gene/?term=9058)(80.882)[SLC13A1](http://www.ncbi.nlm.nih.gov/gene/?term=6561)(80.882)[SUCLG1](http://www.ncbi.nlm.nih.gov/gene/?term=8802)(80.882)[HSD17B6](http://www.ncbi.nlm.nih.gov/gene/?term=8630)(80.882) [SLC25A10](http://www.ncbi.nlm.nih.gov/gene/?term=1468)(80.882)[P3H3](http://www.ncbi.nlm.nih.gov/gene/?term=10536)(80.882)[OXCT2](http://www.ncbi.nlm.nih.gov/gene/?term=64064)(80.882)[TMLHE](http://www.ncbi.nlm.nih.gov/gene/?term=55217)(80.882)[PLOD1](http://www.ncbi.nlm.nih.gov/gene/?term=5351)(80.882) [SUCNR1](http://www.ncbi.nlm.nih.gov/gene/?term=56670)(80.882)[SLC13A3](http://www.ncbi.nlm.nih.gov/gene/?term=64849)(80.882)[ASPH](http://www.ncbi.nlm.nih.gov/gene/?term=444)(80.882)[BBOX1](http://www.ncbi.nlm.nih.gov/gene/?term=8424)(80.882)[SDHB](http://www.ncbi.nlm.nih.gov/gene/?term=6390)(80.882) [OXCT1](http://www.ncbi.nlm.nih.gov/gene/?term=5019)(80.882)[PLOD3](http://www.ncbi.nlm.nih.gov/gene/?term=8985)(80.882)[P4HA1](http://www.ncbi.nlm.nih.gov/gene/?term=5033)(80.882)[SDHC](http://www.ncbi.nlm.nih.gov/gene/?term=6391)(80.882)[P3H2](http://www.ncbi.nlm.nih.gov/gene/?term=55214)(80.882) [SDHA](http://www.ncbi.nlm.nih.gov/gene/?term=6389)(80.882)[P4HA2](http://www.ncbi.nlm.nih.gov/gene/?term=8974)(80.882)[SUCLA2](http://www.ncbi.nlm.nih.gov/gene/?term=8803)(80.882)[SDHD](http://www.ncbi.nlm.nih.gov/gene/?term=6392)(80.882)[P3H1](http://www.ncbi.nlm.nih.gov/gene/?term=64175)(80.882) [DCT](http://www.ncbi.nlm.nih.gov/gene/?term=1638)(55.444)[AKR1C3](http://www.ncbi.nlm.nih.gov/gene/?term=8644)(55.444)[CRTAP](http://www.ncbi.nlm.nih.gov/gene/?term=10491)(55.444)[SLIT2](http://www.ncbi.nlm.nih.gov/gene/?term=9353)(55.444)[ACO2](http://www.ncbi.nlm.nih.gov/gene/?term=50)(55.444) [JMJD6](http://www.ncbi.nlm.nih.gov/gene/?term=23210)(55.444)[SLC13A4](http://www.ncbi.nlm.nih.gov/gene/?term=26266)(55.444)[SDHAF2](http://www.ncbi.nlm.nih.gov/gene/?term=54949)(55.444)[SLC1A3](http://www.ncbi.nlm.nih.gov/gene/?term=6507)(55.444)[SALL1](http://www.ncbi.nlm.nih.gov/gene/?term=6299)(55.444) [HSD17B11](http://www.ncbi.nlm.nih.gov/gene/?term=51170)(55.444)[TYRP1](http://www.ncbi.nlm.nih.gov/gene/?term=7306)(55.444)[HIF1AN](http://www.ncbi.nlm.nih.gov/gene/?term=55662)(55.444)[UROS](http://www.ncbi.nlm.nih.gov/gene/?term=7390)(55.444)[SLC13A5](http://www.ncbi.nlm.nih.gov/gene/?term=284111)(55.444) [P4HB](http://www.ncbi.nlm.nih.gov/gene/?term=5034)(55.444)[CACNA2D1](http://www.ncbi.nlm.nih.gov/gene/?term=781)(48.000)[PLAT](http://www.ncbi.nlm.nih.gov/gene/?term=5327)(48.000)[SLC7A2](http://www.ncbi.nlm.nih.gov/gene/?term=6542)(48.000)[CACNA1A](http://www.ncbi.nlm.nih.gov/gene/?term=773)(48.000) [GRIN3B](http://www.ncbi.nlm.nih.gov/gene/?term=116444)(48.000)[CACNA2D2](http://www.ncbi.nlm.nih.gov/gene/?term=9254)(48.000)[GRIN2A](http://www.ncbi.nlm.nih.gov/gene/?term=2903)(48.000)[TOP1](http://www.ncbi.nlm.nih.gov/gene/?term=7150)(48.000)[PLG](http://www.ncbi.nlm.nih.gov/gene/?term=5340)(48.000) [CACNA1B](http://www.ncbi.nlm.nih.gov/gene/?term=774)(48.000)[GRIN2C](http://www.ncbi.nlm.nih.gov/gene/?term=2905)(48.000)[GRIN2B](http://www.ncbi.nlm.nih.gov/gene/?term=2904)(48.000)[SLC7A3](http://www.ncbi.nlm.nih.gov/gene/?term=84889)(48.000)[KARS](http://www.ncbi.nlm.nih.gov/gene/?term=3735)(48.000) [GRIN3A](http://www.ncbi.nlm.nih.gov/gene/?term=116443)(48.000)[SLC7A1](http://www.ncbi.nlm.nih.gov/gene/?term=6541)(48.000)[SLC7A4](http://www.ncbi.nlm.nih.gov/gene/?term=6545)(48.000)[GRIN2D](http://www.ncbi.nlm.nih.gov/gene/?term=2906)(48.000)[ADORA1](http://www.ncbi.nlm.nih.gov/gene/?term=134)(48.000) [GRIN1](http://www.ncbi.nlm.nih.gov/gene/?term=2902)(48.000)[HDAC1](http://www.ncbi.nlm.nih.gov/gene/?term=3065)(23.000)[GPD1L](http://www.ncbi.nlm.nih.gov/gene/?term=23171)(22.373)[SRD5A1](http://www.ncbi.nlm.nih.gov/gene/?term=6715)(22.373)[FBXO45](http://www.ncbi.nlm.nih.gov/gene/?term=200933)(22.373) [SIRT1](http://www.ncbi.nlm.nih.gov/gene/?term=23411)(22.373)[SHMT1](http://www.ncbi.nlm.nih.gov/gene/?term=6470)(22.373)[SUGCT](http://www.ncbi.nlm.nih.gov/gene/?term=79783)(22.373)[ACSS2](http://www.ncbi.nlm.nih.gov/gene/?term=55902)(22.373)[ARV1](http://www.ncbi.nlm.nih.gov/gene/?term=64801)(22.373) [SIX1](http://www.ncbi.nlm.nih.gov/gene/?term=6495)(22.373)[BDH1](http://www.ncbi.nlm.nih.gov/gene/?term=622)(22.373)[COLGALT2](http://www.ncbi.nlm.nih.gov/gene/?term=23127)(22.373)[SIX4](http://www.ncbi.nlm.nih.gov/gene/?term=51804)(22.373)[MRPS36](http://www.ncbi.nlm.nih.gov/gene/?term=92259)(22.373) [OSBPL8](http://www.ncbi.nlm.nih.gov/gene/?term=114882)(22.373)[SPI1](http://www.ncbi.nlm.nih.gov/gene/?term=6688)(22.373)[SUOX](http://www.ncbi.nlm.nih.gov/gene/?term=6821)(22.373)[CCL2](http://www.ncbi.nlm.nih.gov/gene/?term=6347)(22.373)[BCKDK](http://www.ncbi.nlm.nih.gov/gene/?term=10295)(22.373) [UBE2B](http://www.ncbi.nlm.nih.gov/gene/?term=7320)(22.373)[CYP1A2](http://www.ncbi.nlm.nih.gov/gene/?term=1544)(22.373)[CCL5](http://www.ncbi.nlm.nih.gov/gene/?term=6352)(22.373)[ANK2](http://www.ncbi.nlm.nih.gov/gene/?term=287)(22.373)[HSD17B2](http://www.ncbi.nlm.nih.gov/gene/?term=3294)(22.373) [STIM2](http://www.ncbi.nlm.nih.gov/gene/?term=57620)(22.373)[PKD2](http://www.ncbi.nlm.nih.gov/gene/?term=5311)(22.373)[ALDH9A1](http://www.ncbi.nlm.nih.gov/gene/?term=223)(22.373)[TH](http://www.ncbi.nlm.nih.gov/gene/?term=7054)(22.373)[EPO](http://www.ncbi.nlm.nih.gov/gene/?term=2056)(22.373) [COLGALT1](http://www.ncbi.nlm.nih.gov/gene/?term=79709)(22.373)[NEDD4](http://www.ncbi.nlm.nih.gov/gene/?term=4734)(22.373)[CYP11A1](http://www.ncbi.nlm.nih.gov/gene/?term=1583)(22.373)[DHODH](http://www.ncbi.nlm.nih.gov/gene/?term=1723)(22.373)[CLN3](http://www.ncbi.nlm.nih.gov/gene/?term=1201)(22.373) [ARID1A](http://www.ncbi.nlm.nih.gov/gene/?term=8289)(22.373)[EGLN2](http://www.ncbi.nlm.nih.gov/gene/?term=112398)(22.373)[ACAT1](http://www.ncbi.nlm.nih.gov/gene/?term=38)(22.373)[GALC](http://www.ncbi.nlm.nih.gov/gene/?term=2581)(22.373)[PLCG2](http://www.ncbi.nlm.nih.gov/gene/?term=5336)(22.373) [ACOT4](http://www.ncbi.nlm.nih.gov/gene/?term=122970)(22.373)[PHGDH](http://www.ncbi.nlm.nih.gov/gene/?term=26227)(22.373)[PNLIPRP2](http://www.ncbi.nlm.nih.gov/gene/?term=5408)(22.373)[CYP39A1](http://www.ncbi.nlm.nih.gov/gene/?term=51302)(22.373)[BCKDHA](http://www.ncbi.nlm.nih.gov/gene/?term=593)(22.373) [SLC25A12](http://www.ncbi.nlm.nih.gov/gene/?term=8604)(22.373)[YBX3](http://www.ncbi.nlm.nih.gov/gene/?term=8531)(22.373)[8-Mar](http://www.ncbi.nlm.nih.gov/gene/?term=220972)(22.373)[STIM1](http://www.ncbi.nlm.nih.gov/gene/?term=6786)(22.373)[GAL3ST1](http://www.ncbi.nlm.nih.gov/gene/?term=9514)(22.373) [CDH11](http://www.ncbi.nlm.nih.gov/gene/?term=1009)(22.373)[CACNA1D](http://www.ncbi.nlm.nih.gov/gene/?term=776)(22.373)[DDC](http://www.ncbi.nlm.nih.gov/gene/?term=1644)(22.373)[DHTKD1](http://www.ncbi.nlm.nih.gov/gene/?term=55526)(22.373)[NEDD4L](http://www.ncbi.nlm.nih.gov/gene/?term=23327)(22.373) [BDKRB2](http://www.ncbi.nlm.nih.gov/gene/?term=624)(22.373)[PRPH](http://www.ncbi.nlm.nih.gov/gene/?term=5630)(22.373)[BCKDHB](http://www.ncbi.nlm.nih.gov/gene/?term=594)(22.373)[KCNE5](http://www.ncbi.nlm.nih.gov/gene/?term=23630)(22.373)[SRD5A3](http://www.ncbi.nlm.nih.gov/gene/?term=79644)(22.373) [SMAD7](http://www.ncbi.nlm.nih.gov/gene/?term=4092)(22.373)[PRDM8](http://www.ncbi.nlm.nih.gov/gene/?term=56978)(22.373)[UROD](http://www.ncbi.nlm.nih.gov/gene/?term=7389)(22.373)[UGT8](http://www.ncbi.nlm.nih.gov/gene/?term=7368)(22.373)[ETHE1](http://www.ncbi.nlm.nih.gov/gene/?term=23474)(22.373) [WNT10B](http://www.ncbi.nlm.nih.gov/gene/?term=7480)(22.373)[OGFOD1](http://www.ncbi.nlm.nih.gov/gene/?term=55239)(22.373)[SLC38A7](http://www.ncbi.nlm.nih.gov/gene/?term=55238)(22.373)[ACSS1](http://www.ncbi.nlm.nih.gov/gene/?term=84532)(22.373)[CYP4B1](http://www.ncbi.nlm.nih.gov/gene/?term=1580)(22.373) [STAR](http://www.ncbi.nlm.nih.gov/gene/?term=6770)(22.373)[DLST](http://www.ncbi.nlm.nih.gov/gene/?term=1743)(22.373)[PF4](http://www.ncbi.nlm.nih.gov/gene/?term=5196)(22.373)[CRACR2A](http://www.ncbi.nlm.nih.gov/gene/?term=84766)(22.373)[HSD17B1](http://www.ncbi.nlm.nih.gov/gene/?term=3292)(22.373) [CAMK2D](http://www.ncbi.nlm.nih.gov/gene/?term=817)(22.373)[AP3D1](http://www.ncbi.nlm.nih.gov/gene/?term=8943)(22.373)[MYO5A](http://www.ncbi.nlm.nih.gov/gene/?term=4644)(22.373)[ILVBL](http://www.ncbi.nlm.nih.gov/gene/?term=10994)(22.373)[CAV3](http://www.ncbi.nlm.nih.gov/gene/?term=859)(22.373) [HTT](http://www.ncbi.nlm.nih.gov/gene/?term=3064)(22.373)[IL10](http://www.ncbi.nlm.nih.gov/gene/?term=3586)(22.373)[EGLN3](http://www.ncbi.nlm.nih.gov/gene/?term=112399)(22.373)[OGDHL](http://www.ncbi.nlm.nih.gov/gene/?term=55753)(22.373)[KCNA5](http://www.ncbi.nlm.nih.gov/gene/?term=3741)(22.373) [TST](http://www.ncbi.nlm.nih.gov/gene/?term=7263)(22.373)[SLC1A6](http://www.ncbi.nlm.nih.gov/gene/?term=6511)(22.373)[NPPA](http://www.ncbi.nlm.nih.gov/gene/?term=4878)(22.373)[ACADM](http://www.ncbi.nlm.nih.gov/gene/?term=34)(22.373)[SQRDL](http://www.ncbi.nlm.nih.gov/gene/?term=58472)(22.373) [TMEM110](http://www.ncbi.nlm.nih.gov/gene/?term=375346)(22.373)[CDC42](http://www.ncbi.nlm.nih.gov/gene/?term=998)(22.373)[ARHGEF2](http://www.ncbi.nlm.nih.gov/gene/?term=9181)(22.373)[EGLN1](http://www.ncbi.nlm.nih.gov/gene/?term=54583)(22.373)[ETFDH](http://www.ncbi.nlm.nih.gov/gene/?term=2110)(22.373) [EPHA4](http://www.ncbi.nlm.nih.gov/gene/?term=2043)(22.373)[OCA2](http://www.ncbi.nlm.nih.gov/gene/?term=4948)(22.373)[HSD17B8](http://www.ncbi.nlm.nih.gov/gene/?term=7923)(22.373)[CTNNB1](http://www.ncbi.nlm.nih.gov/gene/?term=1499)(22.373)[P4HA3](http://www.ncbi.nlm.nih.gov/gene/?term=283208)(22.373) [EDA](http://www.ncbi.nlm.nih.gov/gene/?term=1896)(22.373)[HACL1](http://www.ncbi.nlm.nih.gov/gene/?term=26061)(22.373)[RYR2](http://www.ncbi.nlm.nih.gov/gene/?term=6262)(22.373)[HAP1](http://www.ncbi.nlm.nih.gov/gene/?term=9001)(22.373)[PCSK9](http://www.ncbi.nlm.nih.gov/gene/?term=255738)(22.373) [KCNQ1](http://www.ncbi.nlm.nih.gov/gene/?term=3784)(22.373)[CASQ2](http://www.ncbi.nlm.nih.gov/gene/?term=845)(22.373)[GJA5](http://www.ncbi.nlm.nih.gov/gene/?term=2702)(22.373)[DHRS9](http://www.ncbi.nlm.nih.gov/gene/?term=10170)(22.373)[GPR143](http://www.ncbi.nlm.nih.gov/gene/?term=4935)(22.373) [UGT1A1](http://www.ncbi.nlm.nih.gov/gene/?term=54658)(22.373)[DLD](http://www.ncbi.nlm.nih.gov/gene/?term=1738)(22.373)[GNAS](http://www.ncbi.nlm.nih.gov/gene/?term=2778)(22.373)[SLC25A13](http://www.ncbi.nlm.nih.gov/gene/?term=10165)(22.373)[SFXN5](http://www.ncbi.nlm.nih.gov/gene/?term=94097)(22.373) [PLOD2](http://www.ncbi.nlm.nih.gov/gene/?term=5352)(22.373)[CYP1A1](http://www.ncbi.nlm.nih.gov/gene/?term=1543)(22.373)[SULT2A1](http://www.ncbi.nlm.nih.gov/gene/?term=6822)(22.373)[NFX1](http://www.ncbi.nlm.nih.gov/gene/?term=4799)(22.373)[DBT](http://www.ncbi.nlm.nih.gov/gene/?term=1629)(22.373) [SLC25A1](http://www.ncbi.nlm.nih.gov/gene/?term=6576)(22.373) | |
| [Gancaonin B](http://www.megabionet.org/tcmid/ingredient/8134/) | This compound doesn't have any potential target with score larger than 20. | |
| [Codonopsine](http://www.megabionet.org/tcmid/ingredient/3886/) | [CHRNG](http://www.ncbi.nlm.nih.gov/gene/?term=1146)(23.000)[KCNK1](http://www.ncbi.nlm.nih.gov/gene/?term=3775)(23.000)[CHRNA4](http://www.ncbi.nlm.nih.gov/gene/?term=1137)(23.000)[CHRNB3](http://www.ncbi.nlm.nih.gov/gene/?term=1142)(23.000)[OPRD1](http://www.ncbi.nlm.nih.gov/gene/?term=4985)(23.000) [CHRNA7](http://www.ncbi.nlm.nih.gov/gene/?term=1139)(23.000)[SCN5A](http://www.ncbi.nlm.nih.gov/gene/?term=6331)(23.000)[CHRNA1](http://www.ncbi.nlm.nih.gov/gene/?term=1134)(23.000)[CHRNA10](http://www.ncbi.nlm.nih.gov/gene/?term=57053)(23.000)[OPRK1](http://www.ncbi.nlm.nih.gov/gene/?term=4986)(23.000) [CHRNA3](http://www.ncbi.nlm.nih.gov/gene/?term=1136)(23.000)[CHRNB2](http://www.ncbi.nlm.nih.gov/gene/?term=1141)(23.000)[SCN9A](http://www.ncbi.nlm.nih.gov/gene/?term=6335)(23.000)[OPRM1](http://www.ncbi.nlm.nih.gov/gene/?term=4988)(23.000)[ACHE](http://www.ncbi.nlm.nih.gov/gene/?term=43)(23.000) [KCNH2](http://www.ncbi.nlm.nih.gov/gene/?term=3757)(23.000)[CHRNE](http://www.ncbi.nlm.nih.gov/gene/?term=1145)(23.000)[CHRNA2](http://www.ncbi.nlm.nih.gov/gene/?term=1135)(23.000)[CHRNA6](http://www.ncbi.nlm.nih.gov/gene/?term=8973)(23.000)[GP9](http://www.ncbi.nlm.nih.gov/gene/?term=2815)(23.000) [KCNN4](http://www.ncbi.nlm.nih.gov/gene/?term=3783)(23.000)[KCNK6](http://www.ncbi.nlm.nih.gov/gene/?term=9424)(23.000)[CHRND](http://www.ncbi.nlm.nih.gov/gene/?term=1144)(23.000)[BCHE](http://www.ncbi.nlm.nih.gov/gene/?term=590)(23.000)[CHRNA9](http://www.ncbi.nlm.nih.gov/gene/?term=55584)(23.000) [CHRFAM7A](http://www.ncbi.nlm.nih.gov/gene/?term=89832)(23.000)[CHRNA5](http://www.ncbi.nlm.nih.gov/gene/?term=1138)(23.000)[CHRNB4](http://www.ncbi.nlm.nih.gov/gene/?term=1143)(23.000)[CHRNB1](http://www.ncbi.nlm.nih.gov/gene/?term=1140)(23.000) | |
| [Beta-Sitosterol](http://www.megabionet.org/tcmid/ingredient/23097/) | [ESR1](http://www.ncbi.nlm.nih.gov/gene/?term=2099)(122.778)[PGR](http://www.ncbi.nlm.nih.gov/gene/?term=5241)(122.778)[VDR](http://www.ncbi.nlm.nih.gov/gene/?term=7421)(80.882)[CYP27B1](http://www.ncbi.nlm.nih.gov/gene/?term=1594)(80.882)[GC](http://www.ncbi.nlm.nih.gov/gene/?term=2638)(55.444) [SNW1](http://www.ncbi.nlm.nih.gov/gene/?term=22938)(55.444)[AR](http://www.ncbi.nlm.nih.gov/gene/?term=367)(48.000)[NR3C1](http://www.ncbi.nlm.nih.gov/gene/?term=2908)(48.000)[NFKB1](http://www.ncbi.nlm.nih.gov/gene/?term=4790)(22.373)[AKR1C3](http://www.ncbi.nlm.nih.gov/gene/?term=8644)(22.373) [CYP24A1](http://www.ncbi.nlm.nih.gov/gene/?term=1591)(22.373)[GPBAR1](http://www.ncbi.nlm.nih.gov/gene/?term=151306)(22.373)[SNAI2](http://www.ncbi.nlm.nih.gov/gene/?term=6591)(22.373)[MED1](http://www.ncbi.nlm.nih.gov/gene/?term=5469)(22.373)[SNAI1](http://www.ncbi.nlm.nih.gov/gene/?term=6615)(22.373) [CYP3A4](http://www.ncbi.nlm.nih.gov/gene/?term=1576)(22.373)[CALB1](http://www.ncbi.nlm.nih.gov/gene/?term=793)(22.373)[FGF23](http://www.ncbi.nlm.nih.gov/gene/?term=8074)(22.373)[GFI1](http://www.ncbi.nlm.nih.gov/gene/?term=2672)(22.373)[LANCL2](http://www.ncbi.nlm.nih.gov/gene/?term=55915)(22.373) [WNT4](http://www.ncbi.nlm.nih.gov/gene/?term=54361)(22.373)[TCF3](http://www.ncbi.nlm.nih.gov/gene/?term=6929)(22.373)[BAX](http://www.ncbi.nlm.nih.gov/gene/?term=581)(22.373)[KL](http://www.ncbi.nlm.nih.gov/gene/?term=9365)(22.373)[CYP2R1](http://www.ncbi.nlm.nih.gov/gene/?term=120227)(22.373) [PML](http://www.ncbi.nlm.nih.gov/gene/?term=5371)(22.373)[B4GALT1](http://www.ncbi.nlm.nih.gov/gene/?term=2683)(22.373)[S100G](http://www.ncbi.nlm.nih.gov/gene/?term=795)(22.373)[CYP27A1](http://www.ncbi.nlm.nih.gov/gene/?term=1593)(22.373)[KANK2](http://www.ncbi.nlm.nih.gov/gene/?term=25959)(22.373) [IRX5](http://www.ncbi.nlm.nih.gov/gene/?term=10265)(22.373)[RXRA](http://www.ncbi.nlm.nih.gov/gene/?term=6256)(22.373)[NR1H4](http://www.ncbi.nlm.nih.gov/gene/?term=9971)(22.373)[TRIM24](http://www.ncbi.nlm.nih.gov/gene/?term=8805)(22.373) | |
| [Stigmasterol](http://www.megabionet.org/tcmid/ingredient/20353/) | [KCND1](http://www.ncbi.nlm.nih.gov/gene/?term=3750)(80.882)[KCNA3](http://www.ncbi.nlm.nih.gov/gene/?term=3738)(80.882)[PRKAB1](http://www.ncbi.nlm.nih.gov/gene/?term=5564)(80.882)[ADH1A](http://www.ncbi.nlm.nih.gov/gene/?term=124)(80.882)[KCNA10](http://www.ncbi.nlm.nih.gov/gene/?term=3744)(80.882) [GAMT](http://www.ncbi.nlm.nih.gov/gene/?term=2593)(80.882)[KCNC3](http://www.ncbi.nlm.nih.gov/gene/?term=3748)(80.882)[KCNA1](http://www.ncbi.nlm.nih.gov/gene/?term=3736)(80.882)[KCNA2](http://www.ncbi.nlm.nih.gov/gene/?term=3737)(80.882)[TPO](http://www.ncbi.nlm.nih.gov/gene/?term=7173)(80.882) [CAT](http://www.ncbi.nlm.nih.gov/gene/?term=847)(80.882)[KCNB1](http://www.ncbi.nlm.nih.gov/gene/?term=3745)(80.882)[ADH1B](http://www.ncbi.nlm.nih.gov/gene/?term=125)(80.882)[DLG4](http://www.ncbi.nlm.nih.gov/gene/?term=1742)(80.882)[KCNC2](http://www.ncbi.nlm.nih.gov/gene/?term=3747)(80.882) [KCNC1](http://www.ncbi.nlm.nih.gov/gene/?term=3746)(80.882)[ADH1C](http://www.ncbi.nlm.nih.gov/gene/?term=126)(80.882)[KCNB2](http://www.ncbi.nlm.nih.gov/gene/?term=9312)(80.882)[KCNA5](http://www.ncbi.nlm.nih.gov/gene/?term=3741)(80.882)[RNASE1](http://www.ncbi.nlm.nih.gov/gene/?term=6035)(80.882) [KCND2](http://www.ncbi.nlm.nih.gov/gene/?term=3751)(80.882)[ALDH2](http://www.ncbi.nlm.nih.gov/gene/?term=217)(80.882)[KCNA4](http://www.ncbi.nlm.nih.gov/gene/?term=3739)(80.882)[KCNA7](http://www.ncbi.nlm.nih.gov/gene/?term=3743)(80.882)[KCNA6](http://www.ncbi.nlm.nih.gov/gene/?term=3742)(80.882) [KCND3](http://www.ncbi.nlm.nih.gov/gene/?term=3752)(80.882)[GUCY1B3](http://www.ncbi.nlm.nih.gov/gene/?term=2983)(55.444)[GATM](http://www.ncbi.nlm.nih.gov/gene/?term=2628)(55.444)[KCNK4](http://www.ncbi.nlm.nih.gov/gene/?term=50801)(55.444)[IYD](http://www.ncbi.nlm.nih.gov/gene/?term=389434)(55.444) [KCNQ1](http://www.ncbi.nlm.nih.gov/gene/?term=3784)(55.444)[RDH11](http://www.ncbi.nlm.nih.gov/gene/?term=51109)(48.000)[RBP3](http://www.ncbi.nlm.nih.gov/gene/?term=5949)(48.000)[RETSAT](http://www.ncbi.nlm.nih.gov/gene/?term=54884)(48.000)[CYP17A1](http://www.ncbi.nlm.nih.gov/gene/?term=1586)(48.000) [RDH13](http://www.ncbi.nlm.nih.gov/gene/?term=112724)(48.000)[DHRS3](http://www.ncbi.nlm.nih.gov/gene/?term=9249)(48.000)[RDH5](http://www.ncbi.nlm.nih.gov/gene/?term=5959)(48.000)[ESR1](http://www.ncbi.nlm.nih.gov/gene/?term=2099)(48.000)[PGR](http://www.ncbi.nlm.nih.gov/gene/?term=5241)(48.000) [ALDH1A3](http://www.ncbi.nlm.nih.gov/gene/?term=220)(48.000)[RDH12](http://www.ncbi.nlm.nih.gov/gene/?term=145226)(48.000)[OPRK1](http://www.ncbi.nlm.nih.gov/gene/?term=4986)(48.000)[DHRS4](http://www.ncbi.nlm.nih.gov/gene/?term=10901)(48.000)[RBP1](http://www.ncbi.nlm.nih.gov/gene/?term=5947)(48.000) [ALDH1A1](http://www.ncbi.nlm.nih.gov/gene/?term=216)(48.000)[RLBP1](http://www.ncbi.nlm.nih.gov/gene/?term=6017)(48.000)[RDH14](http://www.ncbi.nlm.nih.gov/gene/?term=57665)(48.000)[RDH8](http://www.ncbi.nlm.nih.gov/gene/?term=50700)(48.000)[LRAT](http://www.ncbi.nlm.nih.gov/gene/?term=9227)(48.000) [ALDH1A2](http://www.ncbi.nlm.nih.gov/gene/?term=8854)(48.000)[NR3C2](http://www.ncbi.nlm.nih.gov/gene/?term=4306)(48.000)[FBP1](http://www.ncbi.nlm.nih.gov/gene/?term=2203)(22.373)[DGKI](http://www.ncbi.nlm.nih.gov/gene/?term=9162)(22.373)[ADH7](http://www.ncbi.nlm.nih.gov/gene/?term=131)(22.373) [NFIB](http://www.ncbi.nlm.nih.gov/gene/?term=4781)(22.373)[CYGB](http://www.ncbi.nlm.nih.gov/gene/?term=114757)(22.373)[NRXN3](http://www.ncbi.nlm.nih.gov/gene/?term=9369)(22.373)[LRRC4B](http://www.ncbi.nlm.nih.gov/gene/?term=94030)(22.373)[AQP8](http://www.ncbi.nlm.nih.gov/gene/?term=343)(22.373) [ARX](http://www.ncbi.nlm.nih.gov/gene/?term=170302)(22.373)[IGF1](http://www.ncbi.nlm.nih.gov/gene/?term=3479)(22.373)[SHANK3](http://www.ncbi.nlm.nih.gov/gene/?term=85358)(22.373)[ACY3](http://www.ncbi.nlm.nih.gov/gene/?term=91703)(22.373)[ASPA](http://www.ncbi.nlm.nih.gov/gene/?term=443)(22.373) [NRXN1](http://www.ncbi.nlm.nih.gov/gene/?term=9378)(22.373)[EDN1](http://www.ncbi.nlm.nih.gov/gene/?term=1906)(22.373)[OXT](http://www.ncbi.nlm.nih.gov/gene/?term=5020)(22.373)[MAGI2](http://www.ncbi.nlm.nih.gov/gene/?term=9863)(22.373)[APOE](http://www.ncbi.nlm.nih.gov/gene/?term=348)(22.373) [SOX15](http://www.ncbi.nlm.nih.gov/gene/?term=6665)(22.373)[CNTNAP4](http://www.ncbi.nlm.nih.gov/gene/?term=85445)(22.373)[RNASE4](http://www.ncbi.nlm.nih.gov/gene/?term=6038)(22.373)[RAB3A](http://www.ncbi.nlm.nih.gov/gene/?term=5864)(22.373)[MC4R](http://www.ncbi.nlm.nih.gov/gene/?term=4160)(22.373) [IL1B](http://www.ncbi.nlm.nih.gov/gene/?term=3553)(22.373)[GRIN2A](http://www.ncbi.nlm.nih.gov/gene/?term=2903)(22.373)[SLC17A7](http://www.ncbi.nlm.nih.gov/gene/?term=57030)(22.373)[CYP11A1](http://www.ncbi.nlm.nih.gov/gene/?term=1583)(22.373)[SCN5A](http://www.ncbi.nlm.nih.gov/gene/?term=6331)(22.373) [SCN10A](http://www.ncbi.nlm.nih.gov/gene/?term=6336)(22.373)[SPARC](http://www.ncbi.nlm.nih.gov/gene/?term=6678)(22.373)[SORCS3](http://www.ncbi.nlm.nih.gov/gene/?term=22986)(22.373)[RNASE8](http://www.ncbi.nlm.nih.gov/gene/?term=122665)(22.373)[KCNIP2](http://www.ncbi.nlm.nih.gov/gene/?term=30819)(22.373) [GPX7](http://www.ncbi.nlm.nih.gov/gene/?term=2882)(22.373)[CRLF1](http://www.ncbi.nlm.nih.gov/gene/?term=9244)(22.373)[FADD](http://www.ncbi.nlm.nih.gov/gene/?term=8772)(22.373)[SNTG2](http://www.ncbi.nlm.nih.gov/gene/?term=54221)(22.373)[SCGB1A1](http://www.ncbi.nlm.nih.gov/gene/?term=7356)(22.373) [OXTR](http://www.ncbi.nlm.nih.gov/gene/?term=5021)(22.373)[ALDH1B1](http://www.ncbi.nlm.nih.gov/gene/?term=219)(22.373)[LEP](http://www.ncbi.nlm.nih.gov/gene/?term=3952)(22.373)[NLGN1](http://www.ncbi.nlm.nih.gov/gene/?term=22871)(22.373)[CACNA1D](http://www.ncbi.nlm.nih.gov/gene/?term=776)(22.373) [RAPGEF2](http://www.ncbi.nlm.nih.gov/gene/?term=9693)(22.373)[DARS](http://www.ncbi.nlm.nih.gov/gene/?term=1615)(22.373)[ANK3](http://www.ncbi.nlm.nih.gov/gene/?term=288)(22.373)[KCNE5](http://www.ncbi.nlm.nih.gov/gene/?term=23630)(22.373)[PPARD](http://www.ncbi.nlm.nih.gov/gene/?term=5467)(22.373) [NKX2-1](http://www.ncbi.nlm.nih.gov/gene/?term=7080)(22.373)[MIP](http://www.ncbi.nlm.nih.gov/gene/?term=4284)(22.373)[RYR3](http://www.ncbi.nlm.nih.gov/gene/?term=6263)(22.373)[ZPR1](http://www.ncbi.nlm.nih.gov/gene/?term=8882)(22.373)[ALDH3B1](http://www.ncbi.nlm.nih.gov/gene/?term=221)(22.373) [NPPA](http://www.ncbi.nlm.nih.gov/gene/?term=4878)(22.373)[STX1A](http://www.ncbi.nlm.nih.gov/gene/?term=6804)(22.373)[PAXBP1](http://www.ncbi.nlm.nih.gov/gene/?term=94104)(22.373)[PAX7](http://www.ncbi.nlm.nih.gov/gene/?term=5081)(22.373)[ALDH3B2](http://www.ncbi.nlm.nih.gov/gene/?term=222)(22.373) [RNASE2](http://www.ncbi.nlm.nih.gov/gene/?term=6036)(22.373)[NRXN2](http://www.ncbi.nlm.nih.gov/gene/?term=9379)(22.373)[ADORA1](http://www.ncbi.nlm.nih.gov/gene/?term=134)(22.373)[ADH4](http://www.ncbi.nlm.nih.gov/gene/?term=127)(22.373)[GJA5](http://www.ncbi.nlm.nih.gov/gene/?term=2702)(22.373) [FAS](http://www.ncbi.nlm.nih.gov/gene/?term=355)(22.373)[DAB2IP](http://www.ncbi.nlm.nih.gov/gene/?term=153090)(22.373)[CYP2E1](http://www.ncbi.nlm.nih.gov/gene/?term=1571)(22.373)[ACY1](http://www.ncbi.nlm.nih.gov/gene/?term=95)(22.373) | |
| [Atractylenolide Iii](http://www.megabionet.org/tcmid/ingredient/1967/) | [CHRM3](http://www.ncbi.nlm.nih.gov/gene/?term=1131)(80.882)[CHRM1](http://www.ncbi.nlm.nih.gov/gene/?term=1128)(80.882)[CHRM2](http://www.ncbi.nlm.nih.gov/gene/?term=1129)(80.882)[ATP1A1](http://www.ncbi.nlm.nih.gov/gene/?term=476)(48.000)[ARFGEF2](http://www.ncbi.nlm.nih.gov/gene/?term=10564)(22.373) [DOCK5](http://www.ncbi.nlm.nih.gov/gene/?term=80005)(22.373)[NTSR1](http://www.ncbi.nlm.nih.gov/gene/?term=4923)(22.373)[CHRM5](http://www.ncbi.nlm.nih.gov/gene/?term=1133)(22.373)[GNA15](http://www.ncbi.nlm.nih.gov/gene/?term=2769)(22.373)[P2RX1](http://www.ncbi.nlm.nih.gov/gene/?term=5023)(22.373) [DOCK4](http://www.ncbi.nlm.nih.gov/gene/?term=9732)(22.373)[MAP2K1](http://www.ncbi.nlm.nih.gov/gene/?term=5604)(22.373)[CHRM4](http://www.ncbi.nlm.nih.gov/gene/?term=1132)(22.373) | |
| [Glyeurysaponin](http://www.megabionet.org/tcmid/ingredient/8846/) | This compound doesn't have any potential target with score larger than 20. | |
| [Taraxerol](http://www.megabionet.org/tcmid/ingredient/20695/) | [ESR1](http://www.ncbi.nlm.nih.gov/gene/?term=2099)(122.778)[PGR](http://www.ncbi.nlm.nih.gov/gene/?term=5241)(122.778)[VDR](http://www.ncbi.nlm.nih.gov/gene/?term=7421)(80.882)[CYP27B1](http://www.ncbi.nlm.nih.gov/gene/?term=1594)(80.882)[GC](http://www.ncbi.nlm.nih.gov/gene/?term=2638)(55.444) [SNW1](http://www.ncbi.nlm.nih.gov/gene/?term=22938)(55.444)[RDH11](http://www.ncbi.nlm.nih.gov/gene/?term=51109)(48.000)[SRD5A1](http://www.ncbi.nlm.nih.gov/gene/?term=6715)(48.000)[RBP3](http://www.ncbi.nlm.nih.gov/gene/?term=5949)(48.000)[RETSAT](http://www.ncbi.nlm.nih.gov/gene/?term=54884)(48.000) [RDH13](http://www.ncbi.nlm.nih.gov/gene/?term=112724)(48.000)[DHRS3](http://www.ncbi.nlm.nih.gov/gene/?term=9249)(48.000)[RDH5](http://www.ncbi.nlm.nih.gov/gene/?term=5959)(48.000)[F12](http://www.ncbi.nlm.nih.gov/gene/?term=2161)(48.000)[AR](http://www.ncbi.nlm.nih.gov/gene/?term=367)(48.000) [NR3C1](http://www.ncbi.nlm.nih.gov/gene/?term=2908)(48.000)[ALDH1A3](http://www.ncbi.nlm.nih.gov/gene/?term=220)(48.000)[RDH12](http://www.ncbi.nlm.nih.gov/gene/?term=145226)(48.000)[DHRS4](http://www.ncbi.nlm.nih.gov/gene/?term=10901)(48.000)[RBP1](http://www.ncbi.nlm.nih.gov/gene/?term=5947)(48.000) [ALDH1A1](http://www.ncbi.nlm.nih.gov/gene/?term=216)(48.000)[RLBP1](http://www.ncbi.nlm.nih.gov/gene/?term=6017)(48.000)[RDH14](http://www.ncbi.nlm.nih.gov/gene/?term=57665)(48.000)[RDH8](http://www.ncbi.nlm.nih.gov/gene/?term=50700)(48.000)[ANXA1](http://www.ncbi.nlm.nih.gov/gene/?term=301)(48.000) [LRAT](http://www.ncbi.nlm.nih.gov/gene/?term=9227)(48.000)[ALDH1A2](http://www.ncbi.nlm.nih.gov/gene/?term=8854)(48.000)[NFKB1](http://www.ncbi.nlm.nih.gov/gene/?term=4790)(22.373)[AKR1C3](http://www.ncbi.nlm.nih.gov/gene/?term=8644)(22.373)[CYP24A1](http://www.ncbi.nlm.nih.gov/gene/?term=1591)(22.373) [GPBAR1](http://www.ncbi.nlm.nih.gov/gene/?term=151306)(22.373)[SNAI2](http://www.ncbi.nlm.nih.gov/gene/?term=6591)(22.373)[MED1](http://www.ncbi.nlm.nih.gov/gene/?term=5469)(22.373)[SNAI1](http://www.ncbi.nlm.nih.gov/gene/?term=6615)(22.373)[CYP3A4](http://www.ncbi.nlm.nih.gov/gene/?term=1576)(22.373) [CALB1](http://www.ncbi.nlm.nih.gov/gene/?term=793)(22.373)[FGF23](http://www.ncbi.nlm.nih.gov/gene/?term=8074)(22.373)[GFI1](http://www.ncbi.nlm.nih.gov/gene/?term=2672)(22.373)[LANCL2](http://www.ncbi.nlm.nih.gov/gene/?term=55915)(22.373)[WNT4](http://www.ncbi.nlm.nih.gov/gene/?term=54361)(22.373) [TCF3](http://www.ncbi.nlm.nih.gov/gene/?term=6929)(22.373)[BAX](http://www.ncbi.nlm.nih.gov/gene/?term=581)(22.373)[KL](http://www.ncbi.nlm.nih.gov/gene/?term=9365)(22.373)[CYP2R1](http://www.ncbi.nlm.nih.gov/gene/?term=120227)(22.373)[PML](http://www.ncbi.nlm.nih.gov/gene/?term=5371)(22.373) [B4GALT1](http://www.ncbi.nlm.nih.gov/gene/?term=2683)(22.373)[S100G](http://www.ncbi.nlm.nih.gov/gene/?term=795)(22.373)[CYP27A1](http://www.ncbi.nlm.nih.gov/gene/?term=1593)(22.373)[KANK2](http://www.ncbi.nlm.nih.gov/gene/?term=25959)(22.373)[IRX5](http://www.ncbi.nlm.nih.gov/gene/?term=10265)(22.373) [RXRA](http://www.ncbi.nlm.nih.gov/gene/?term=6256)(22.373)[NR1H4](http://www.ncbi.nlm.nih.gov/gene/?term=9971)(22.373)[TRIM24](http://www.ncbi.nlm.nih.gov/gene/?term=8805)(22.373) | |
| [Tangshenoside Ii](http://www.megabionet.org/tcmid/ingredient/20657/) | This compound doesn't have any potential target with score larger than 20. | |
| [Panasenoside](http://www.megabionet.org/tcmid/ingredient/16576/) | This compound doesn't have any potential target with score larger than 20. | |
| [Isoliquiritigenin](http://www.megabionet.org/tcmid/ingredient/11501/) | [HSD17B1](http://www.ncbi.nlm.nih.gov/gene/?term=3292)(80.882)[ESR1](http://www.ncbi.nlm.nih.gov/gene/?term=2099)(23.000)[CFTR](http://www.ncbi.nlm.nih.gov/gene/?term=1080)(23.000)[AKR1C3](http://www.ncbi.nlm.nih.gov/gene/?term=8644)(22.373)[HSD17B6](http://www.ncbi.nlm.nih.gov/gene/?term=8630)(22.373) [HSD17B2](http://www.ncbi.nlm.nih.gov/gene/?term=3294)(22.373)[CYP11A1](http://www.ncbi.nlm.nih.gov/gene/?term=1583)(22.373)[WNT4](http://www.ncbi.nlm.nih.gov/gene/?term=54361)(22.373)[STAR](http://www.ncbi.nlm.nih.gov/gene/?term=6770)(22.373)[HSD17B8](http://www.ncbi.nlm.nih.gov/gene/?term=7923)(22.373) [DHRS9](http://www.ncbi.nlm.nih.gov/gene/?term=10170)(22.373)[HSD17B3](http://www.ncbi.nlm.nih.gov/gene/?term=3293)(22.373) | |
| [Methyl 2-Hydroxy-3,4-Dimethoxy Benzoate](http://www.megabionet.org/tcmid/ingredient/14494/) | This compound doesn't have any potential target with score larger than 20. | |
| [Rhamnose](http://www.megabionet.org/tcmid/ingredient/18724/) | [NFKB2](http://www.ncbi.nlm.nih.gov/gene/?term=4791)(48.000)[TNF](http://www.ncbi.nlm.nih.gov/gene/?term=7124)(48.000)[MMP9](http://www.ncbi.nlm.nih.gov/gene/?term=4318)(48.000)[IFNG](http://www.ncbi.nlm.nih.gov/gene/?term=3458)(48.000) | |
| [Soyasapogenol B](http://www.megabionet.org/tcmid/ingredient/20100/) | [ESR1](http://www.ncbi.nlm.nih.gov/gene/?term=2099)(122.778)[PGR](http://www.ncbi.nlm.nih.gov/gene/?term=5241)(122.778)[AR](http://www.ncbi.nlm.nih.gov/gene/?term=367)(48.000)[NR3C1](http://www.ncbi.nlm.nih.gov/gene/?term=2908)(48.000)[ANXA1](http://www.ncbi.nlm.nih.gov/gene/?term=301)(48.000) | |
| [Neoliquiritin](http://www.megabionet.org/tcmid/ingredient/15421/) | [SOAT1](http://www.ncbi.nlm.nih.gov/gene/?term=6646)(48.000)[MTTP](http://www.ncbi.nlm.nih.gov/gene/?term=4547)(48.000)[SOAT2](http://www.ncbi.nlm.nih.gov/gene/?term=8435)(48.000) | |
| [Ganoderic Acid A](http://www.megabionet.org/tcmid/ingredient/27722/) | [PTGER1](http://www.ncbi.nlm.nih.gov/gene/?term=5731)(80.882)[PTGER4](http://www.ncbi.nlm.nih.gov/gene/?term=5734)(80.882)[PTGER2](http://www.ncbi.nlm.nih.gov/gene/?term=5732)(80.882)[PTGER3](http://www.ncbi.nlm.nih.gov/gene/?term=5733)(80.882)[CD300A](http://www.ncbi.nlm.nih.gov/gene/?term=11314)(55.444) [KIF14](http://www.ncbi.nlm.nih.gov/gene/?term=9928)(55.444)[SRD5A1](http://www.ncbi.nlm.nih.gov/gene/?term=6715)(48.000)[CYP17A1](http://www.ncbi.nlm.nih.gov/gene/?term=1586)(48.000)[PRLR](http://www.ncbi.nlm.nih.gov/gene/?term=5618)(48.000)[ESR1](http://www.ncbi.nlm.nih.gov/gene/?term=2099)(48.000) [PGR](http://www.ncbi.nlm.nih.gov/gene/?term=5241)(48.000)[AR](http://www.ncbi.nlm.nih.gov/gene/?term=367)(48.000)[NR3C1](http://www.ncbi.nlm.nih.gov/gene/?term=2908)(48.000)[PTGIR](http://www.ncbi.nlm.nih.gov/gene/?term=5739)(48.000)[OPRK1](http://www.ncbi.nlm.nih.gov/gene/?term=4986)(48.000) [PTGFR](http://www.ncbi.nlm.nih.gov/gene/?term=5737)(48.000)[ANXA1](http://www.ncbi.nlm.nih.gov/gene/?term=301)(48.000)[NR3C2](http://www.ncbi.nlm.nih.gov/gene/?term=4306)(48.000)[DAPK2](http://www.ncbi.nlm.nih.gov/gene/?term=23604)(22.373)[HPGD](http://www.ncbi.nlm.nih.gov/gene/?term=3248)(22.373) [ADAM8](http://www.ncbi.nlm.nih.gov/gene/?term=101)(22.373)[CCL24](http://www.ncbi.nlm.nih.gov/gene/?term=6369)(22.373)[IL4](http://www.ncbi.nlm.nih.gov/gene/?term=3565)(22.373) | |
| [Astragaloside V](http://www.megabionet.org/tcmid/ingredient/1940/) | [CXCR4](http://www.ncbi.nlm.nih.gov/gene/?term=7852)(48.000) | |
| [Frutinone A](http://www.megabionet.org/tcmid/ingredient/7972/) | This compound doesn't have any potential target with score larger than 20. | |
| [5-Methoxymethyl Furfural](http://www.megabionet.org/tcmid/ingredient/14016/) | This compound doesn't have any potential target with score larger than 20. | |
| [Uralsaponin A](http://www.megabionet.org/tcmid/ingredient/22227/) | [AR](http://www.ncbi.nlm.nih.gov/gene/?term=367)(48.000)[NR3C1](http://www.ncbi.nlm.nih.gov/gene/?term=2908)(48.000)[ATP1A1](http://www.ncbi.nlm.nih.gov/gene/?term=476)(48.000) | |
| [Glycyroside](http://www.megabionet.org/tcmid/ingredient/8839/) | This compound doesn't have any potential target with score larger than 20. | |
| [Friedelan-3-One](http://www.megabionet.org/tcmid/ingredient/7950/) | [AR](http://www.ncbi.nlm.nih.gov/gene/?term=367)(48.000) | |
| [Heneicosanic Acid](http://www.megabionet.org/tcmid/ingredient/31073/) | [AKR1D1](http://www.ncbi.nlm.nih.gov/gene/?term=6718)(686.000)[TYR](http://www.ncbi.nlm.nih.gov/gene/?term=7299)(686.000)[SRD5A2](http://www.ncbi.nlm.nih.gov/gene/?term=6716)(686.000)[SCN11A](http://www.ncbi.nlm.nih.gov/gene/?term=11280)(122.778)[SCN2B](http://www.ncbi.nlm.nih.gov/gene/?term=6327)(122.778) [ABAT](http://www.ncbi.nlm.nih.gov/gene/?term=18)(122.778)[SCN1A](http://www.ncbi.nlm.nih.gov/gene/?term=6323)(122.778)[ESRRG](http://www.ncbi.nlm.nih.gov/gene/?term=2104)(122.778)[SCN3B](http://www.ncbi.nlm.nih.gov/gene/?term=55800)(122.778)[COX6C](http://www.ncbi.nlm.nih.gov/gene/?term=1345)(122.778) [COX5B](http://www.ncbi.nlm.nih.gov/gene/?term=1329)(122.778)[COX7C](http://www.ncbi.nlm.nih.gov/gene/?term=1350)(122.778)[COX1](http://www.ncbi.nlm.nih.gov/gene/?term=4512)(122.778)[SCN3A](http://www.ncbi.nlm.nih.gov/gene/?term=6328)(122.778)[AKR1C2](http://www.ncbi.nlm.nih.gov/gene/?term=1646)(122.778) [SCN7A](http://www.ncbi.nlm.nih.gov/gene/?term=6332)(122.778)[SCN5A](http://www.ncbi.nlm.nih.gov/gene/?term=6331)(122.778)[SCN10A](http://www.ncbi.nlm.nih.gov/gene/?term=6336)(122.778)[ALDH5A1](http://www.ncbi.nlm.nih.gov/gene/?term=7915)(122.778)[SCN2A](http://www.ncbi.nlm.nih.gov/gene/?term=6326)(122.778) [COX5A](http://www.ncbi.nlm.nih.gov/gene/?term=9377)(122.778)[COX3](http://www.ncbi.nlm.nih.gov/gene/?term=4514)(122.778)[AR](http://www.ncbi.nlm.nih.gov/gene/?term=367)(122.778)[COX7A1](http://www.ncbi.nlm.nih.gov/gene/?term=1346)(122.778)[FECH](http://www.ncbi.nlm.nih.gov/gene/?term=2235)(122.778) [COX4I1](http://www.ncbi.nlm.nih.gov/gene/?term=1327)(122.778)[HDAC9](http://www.ncbi.nlm.nih.gov/gene/?term=9734)(122.778)[PLA2G1B](http://www.ncbi.nlm.nih.gov/gene/?term=5319)(122.778)[COX6A2](http://www.ncbi.nlm.nih.gov/gene/?term=1339)(122.778)[SCN9A](http://www.ncbi.nlm.nih.gov/gene/?term=6335)(122.778) [SCN4A](http://www.ncbi.nlm.nih.gov/gene/?term=6329)(122.778)[SCN4B](http://www.ncbi.nlm.nih.gov/gene/?term=6330)(122.778)[ADH1C](http://www.ncbi.nlm.nih.gov/gene/?term=126)(122.778)[COX6B1](http://www.ncbi.nlm.nih.gov/gene/?term=1340)(122.778)[FABP6](http://www.ncbi.nlm.nih.gov/gene/?term=2172)(122.778) [CES1](http://www.ncbi.nlm.nih.gov/gene/?term=1066)(122.778)[ACADSB](http://www.ncbi.nlm.nih.gov/gene/?term=36)(122.778)[SCN8A](http://www.ncbi.nlm.nih.gov/gene/?term=6334)(122.778)[COX7B](http://www.ncbi.nlm.nih.gov/gene/?term=1349)(122.778)[SCN1B](http://www.ncbi.nlm.nih.gov/gene/?term=6324)(122.778) [COX2](http://www.ncbi.nlm.nih.gov/gene/?term=4513)(122.778)[OGDH](http://www.ncbi.nlm.nih.gov/gene/?term=4967)(122.778)[HDAC2](http://www.ncbi.nlm.nih.gov/gene/?term=3066)(122.778)[COX8A](http://www.ncbi.nlm.nih.gov/gene/?term=1351)(122.778)[NR1H4](http://www.ncbi.nlm.nih.gov/gene/?term=9971)(122.778) [SUCLG2](http://www.ncbi.nlm.nih.gov/gene/?term=8801)(80.882)[SLC13A2](http://www.ncbi.nlm.nih.gov/gene/?term=9058)(80.882)[SLC13A1](http://www.ncbi.nlm.nih.gov/gene/?term=6561)(80.882)[SUCLG1](http://www.ncbi.nlm.nih.gov/gene/?term=8802)(80.882)[HSD17B6](http://www.ncbi.nlm.nih.gov/gene/?term=8630)(80.882) [SLC25A10](http://www.ncbi.nlm.nih.gov/gene/?term=1468)(80.882)[P3H3](http://www.ncbi.nlm.nih.gov/gene/?term=10536)(80.882)[OXCT2](http://www.ncbi.nlm.nih.gov/gene/?term=64064)(80.882)[TMLHE](http://www.ncbi.nlm.nih.gov/gene/?term=55217)(80.882)[PLOD1](http://www.ncbi.nlm.nih.gov/gene/?term=5351)(80.882) [SUCNR1](http://www.ncbi.nlm.nih.gov/gene/?term=56670)(80.882)[SLC13A3](http://www.ncbi.nlm.nih.gov/gene/?term=64849)(80.882)[ASPH](http://www.ncbi.nlm.nih.gov/gene/?term=444)(80.882)[BBOX1](http://www.ncbi.nlm.nih.gov/gene/?term=8424)(80.882)[SDHB](http://www.ncbi.nlm.nih.gov/gene/?term=6390)(80.882) [OXCT1](http://www.ncbi.nlm.nih.gov/gene/?term=5019)(80.882)[PLOD3](http://www.ncbi.nlm.nih.gov/gene/?term=8985)(80.882)[P4HA1](http://www.ncbi.nlm.nih.gov/gene/?term=5033)(80.882)[SDHC](http://www.ncbi.nlm.nih.gov/gene/?term=6391)(80.882)[P3H2](http://www.ncbi.nlm.nih.gov/gene/?term=55214)(80.882) [SDHA](http://www.ncbi.nlm.nih.gov/gene/?term=6389)(80.882)[P4HA2](http://www.ncbi.nlm.nih.gov/gene/?term=8974)(80.882)[SUCLA2](http://www.ncbi.nlm.nih.gov/gene/?term=8803)(80.882)[SDHD](http://www.ncbi.nlm.nih.gov/gene/?term=6392)(80.882)[P3H1](http://www.ncbi.nlm.nih.gov/gene/?term=64175)(80.882) [DCT](http://www.ncbi.nlm.nih.gov/gene/?term=1638)(55.444)[AKR1C3](http://www.ncbi.nlm.nih.gov/gene/?term=8644)(55.444)[CRTAP](http://www.ncbi.nlm.nih.gov/gene/?term=10491)(55.444)[SLIT2](http://www.ncbi.nlm.nih.gov/gene/?term=9353)(55.444)[ACO2](http://www.ncbi.nlm.nih.gov/gene/?term=50)(55.444) [JMJD6](http://www.ncbi.nlm.nih.gov/gene/?term=23210)(55.444)[SLC13A4](http://www.ncbi.nlm.nih.gov/gene/?term=26266)(55.444)[SDHAF2](http://www.ncbi.nlm.nih.gov/gene/?term=54949)(55.444)[SLC1A3](http://www.ncbi.nlm.nih.gov/gene/?term=6507)(55.444)[SALL1](http://www.ncbi.nlm.nih.gov/gene/?term=6299)(55.444) [HSD17B11](http://www.ncbi.nlm.nih.gov/gene/?term=51170)(55.444)[TYRP1](http://www.ncbi.nlm.nih.gov/gene/?term=7306)(55.444)[HIF1AN](http://www.ncbi.nlm.nih.gov/gene/?term=55662)(55.444)[UROS](http://www.ncbi.nlm.nih.gov/gene/?term=7390)(55.444)[SLC13A5](http://www.ncbi.nlm.nih.gov/gene/?term=284111)(55.444) [P4HB](http://www.ncbi.nlm.nih.gov/gene/?term=5034)(55.444)[CACNA2D1](http://www.ncbi.nlm.nih.gov/gene/?term=781)(48.000)[PLAT](http://www.ncbi.nlm.nih.gov/gene/?term=5327)(48.000)[SLC7A2](http://www.ncbi.nlm.nih.gov/gene/?term=6542)(48.000)[CACNA1A](http://www.ncbi.nlm.nih.gov/gene/?term=773)(48.000) [GRIN3B](http://www.ncbi.nlm.nih.gov/gene/?term=116444)(48.000)[CACNA2D2](http://www.ncbi.nlm.nih.gov/gene/?term=9254)(48.000)[GRIN2A](http://www.ncbi.nlm.nih.gov/gene/?term=2903)(48.000)[TOP1](http://www.ncbi.nlm.nih.gov/gene/?term=7150)(48.000)[PLG](http://www.ncbi.nlm.nih.gov/gene/?term=5340)(48.000) [CACNA1B](http://www.ncbi.nlm.nih.gov/gene/?term=774)(48.000)[GRIN2C](http://www.ncbi.nlm.nih.gov/gene/?term=2905)(48.000)[GRIN2B](http://www.ncbi.nlm.nih.gov/gene/?term=2904)(48.000)[SLC7A3](http://www.ncbi.nlm.nih.gov/gene/?term=84889)(48.000)[KARS](http://www.ncbi.nlm.nih.gov/gene/?term=3735)(48.000) [GRIN3A](http://www.ncbi.nlm.nih.gov/gene/?term=116443)(48.000)[SLC7A1](http://www.ncbi.nlm.nih.gov/gene/?term=6541)(48.000)[SLC7A4](http://www.ncbi.nlm.nih.gov/gene/?term=6545)(48.000)[GRIN2D](http://www.ncbi.nlm.nih.gov/gene/?term=2906)(48.000)[ADORA1](http://www.ncbi.nlm.nih.gov/gene/?term=134)(48.000) [GRIN1](http://www.ncbi.nlm.nih.gov/gene/?term=2902)(48.000)[HDAC1](http://www.ncbi.nlm.nih.gov/gene/?term=3065)(23.000)[GPD1L](http://www.ncbi.nlm.nih.gov/gene/?term=23171)(22.373)[SRD5A1](http://www.ncbi.nlm.nih.gov/gene/?term=6715)(22.373)[FBXO45](http://www.ncbi.nlm.nih.gov/gene/?term=200933)(22.373) [SIRT1](http://www.ncbi.nlm.nih.gov/gene/?term=23411)(22.373)[SHMT1](http://www.ncbi.nlm.nih.gov/gene/?term=6470)(22.373)[SUGCT](http://www.ncbi.nlm.nih.gov/gene/?term=79783)(22.373)[ACSS2](http://www.ncbi.nlm.nih.gov/gene/?term=55902)(22.373)[ARV1](http://www.ncbi.nlm.nih.gov/gene/?term=64801)(22.373) [SIX1](http://www.ncbi.nlm.nih.gov/gene/?term=6495)(22.373)[BDH1](http://www.ncbi.nlm.nih.gov/gene/?term=622)(22.373)[COLGALT2](http://www.ncbi.nlm.nih.gov/gene/?term=23127)(22.373)[SIX4](http://www.ncbi.nlm.nih.gov/gene/?term=51804)(22.373)[MRPS36](http://www.ncbi.nlm.nih.gov/gene/?term=92259)(22.373) [OSBPL8](http://www.ncbi.nlm.nih.gov/gene/?term=114882)(22.373)[SPI1](http://www.ncbi.nlm.nih.gov/gene/?term=6688)(22.373)[SUOX](http://www.ncbi.nlm.nih.gov/gene/?term=6821)(22.373)[CCL2](http://www.ncbi.nlm.nih.gov/gene/?term=6347)(22.373)[BCKDK](http://www.ncbi.nlm.nih.gov/gene/?term=10295)(22.373) [UBE2B](http://www.ncbi.nlm.nih.gov/gene/?term=7320)(22.373)[CYP1A2](http://www.ncbi.nlm.nih.gov/gene/?term=1544)(22.373)[CCL5](http://www.ncbi.nlm.nih.gov/gene/?term=6352)(22.373)[ANK2](http://www.ncbi.nlm.nih.gov/gene/?term=287)(22.373)[HSD17B2](http://www.ncbi.nlm.nih.gov/gene/?term=3294)(22.373) [STIM2](http://www.ncbi.nlm.nih.gov/gene/?term=57620)(22.373)[PKD2](http://www.ncbi.nlm.nih.gov/gene/?term=5311)(22.373)[ALDH9A1](http://www.ncbi.nlm.nih.gov/gene/?term=223)(22.373)[TH](http://www.ncbi.nlm.nih.gov/gene/?term=7054)(22.373)[EPO](http://www.ncbi.nlm.nih.gov/gene/?term=2056)(22.373) [COLGALT1](http://www.ncbi.nlm.nih.gov/gene/?term=79709)(22.373)[NEDD4](http://www.ncbi.nlm.nih.gov/gene/?term=4734)(22.373)[CYP11A1](http://www.ncbi.nlm.nih.gov/gene/?term=1583)(22.373)[DHODH](http://www.ncbi.nlm.nih.gov/gene/?term=1723)(22.373)[CLN3](http://www.ncbi.nlm.nih.gov/gene/?term=1201)(22.373) [ARID1A](http://www.ncbi.nlm.nih.gov/gene/?term=8289)(22.373)[EGLN2](http://www.ncbi.nlm.nih.gov/gene/?term=112398)(22.373)[ACAT1](http://www.ncbi.nlm.nih.gov/gene/?term=38)(22.373)[GALC](http://www.ncbi.nlm.nih.gov/gene/?term=2581)(22.373)[PLCG2](http://www.ncbi.nlm.nih.gov/gene/?term=5336)(22.373) [ACOT4](http://www.ncbi.nlm.nih.gov/gene/?term=122970)(22.373)[PHGDH](http://www.ncbi.nlm.nih.gov/gene/?term=26227)(22.373)[PNLIPRP2](http://www.ncbi.nlm.nih.gov/gene/?term=5408)(22.373)[CYP39A1](http://www.ncbi.nlm.nih.gov/gene/?term=51302)(22.373)[BCKDHA](http://www.ncbi.nlm.nih.gov/gene/?term=593)(22.373) [SLC25A12](http://www.ncbi.nlm.nih.gov/gene/?term=8604)(22.373)[YBX3](http://www.ncbi.nlm.nih.gov/gene/?term=8531)(22.373)[8-Mar](http://www.ncbi.nlm.nih.gov/gene/?term=220972)(22.373)[STIM1](http://www.ncbi.nlm.nih.gov/gene/?term=6786)(22.373)[GAL3ST1](http://www.ncbi.nlm.nih.gov/gene/?term=9514)(22.373) [CDH11](http://www.ncbi.nlm.nih.gov/gene/?term=1009)(22.373)[CACNA1D](http://www.ncbi.nlm.nih.gov/gene/?term=776)(22.373)[DDC](http://www.ncbi.nlm.nih.gov/gene/?term=1644)(22.373)[DHTKD1](http://www.ncbi.nlm.nih.gov/gene/?term=55526)(22.373)[NEDD4L](http://www.ncbi.nlm.nih.gov/gene/?term=23327)(22.373) [BDKRB2](http://www.ncbi.nlm.nih.gov/gene/?term=624)(22.373)[PRPH](http://www.ncbi.nlm.nih.gov/gene/?term=5630)(22.373)[BCKDHB](http://www.ncbi.nlm.nih.gov/gene/?term=594)(22.373)[KCNE5](http://www.ncbi.nlm.nih.gov/gene/?term=23630)(22.373)[SRD5A3](http://www.ncbi.nlm.nih.gov/gene/?term=79644)(22.373) [SMAD7](http://www.ncbi.nlm.nih.gov/gene/?term=4092)(22.373)[PRDM8](http://www.ncbi.nlm.nih.gov/gene/?term=56978)(22.373)[UROD](http://www.ncbi.nlm.nih.gov/gene/?term=7389)(22.373)[UGT8](http://www.ncbi.nlm.nih.gov/gene/?term=7368)(22.373)[ETHE1](http://www.ncbi.nlm.nih.gov/gene/?term=23474)(22.373) [WNT10B](http://www.ncbi.nlm.nih.gov/gene/?term=7480)(22.373)[OGFOD1](http://www.ncbi.nlm.nih.gov/gene/?term=55239)(22.373)[SLC38A7](http://www.ncbi.nlm.nih.gov/gene/?term=55238)(22.373)[ACSS1](http://www.ncbi.nlm.nih.gov/gene/?term=84532)(22.373)[CYP4B1](http://www.ncbi.nlm.nih.gov/gene/?term=1580)(22.373) [STAR](http://www.ncbi.nlm.nih.gov/gene/?term=6770)(22.373)[DLST](http://www.ncbi.nlm.nih.gov/gene/?term=1743)(22.373)[PF4](http://www.ncbi.nlm.nih.gov/gene/?term=5196)(22.373)[CRACR2A](http://www.ncbi.nlm.nih.gov/gene/?term=84766)(22.373)[HSD17B1](http://www.ncbi.nlm.nih.gov/gene/?term=3292)(22.373) [CAMK2D](http://www.ncbi.nlm.nih.gov/gene/?term=817)(22.373)[AP3D1](http://www.ncbi.nlm.nih.gov/gene/?term=8943)(22.373)[MYO5A](http://www.ncbi.nlm.nih.gov/gene/?term=4644)(22.373)[ILVBL](http://www.ncbi.nlm.nih.gov/gene/?term=10994)(22.373)[CAV3](http://www.ncbi.nlm.nih.gov/gene/?term=859)(22.373) [HTT](http://www.ncbi.nlm.nih.gov/gene/?term=3064)(22.373)[IL10](http://www.ncbi.nlm.nih.gov/gene/?term=3586)(22.373)[EGLN3](http://www.ncbi.nlm.nih.gov/gene/?term=112399)(22.373)[OGDHL](http://www.ncbi.nlm.nih.gov/gene/?term=55753)(22.373)[KCNA5](http://www.ncbi.nlm.nih.gov/gene/?term=3741)(22.373) [TST](http://www.ncbi.nlm.nih.gov/gene/?term=7263)(22.373)[SLC1A6](http://www.ncbi.nlm.nih.gov/gene/?term=6511)(22.373)[NPPA](http://www.ncbi.nlm.nih.gov/gene/?term=4878)(22.373)[ACADM](http://www.ncbi.nlm.nih.gov/gene/?term=34)(22.373)[SQRDL](http://www.ncbi.nlm.nih.gov/gene/?term=58472)(22.373) [TMEM110](http://www.ncbi.nlm.nih.gov/gene/?term=375346)(22.373)[CDC42](http://www.ncbi.nlm.nih.gov/gene/?term=998)(22.373)[ARHGEF2](http://www.ncbi.nlm.nih.gov/gene/?term=9181)(22.373)[EGLN1](http://www.ncbi.nlm.nih.gov/gene/?term=54583)(22.373)[ETFDH](http://www.ncbi.nlm.nih.gov/gene/?term=2110)(22.373) [EPHA4](http://www.ncbi.nlm.nih.gov/gene/?term=2043)(22.373)[OCA2](http://www.ncbi.nlm.nih.gov/gene/?term=4948)(22.373)[HSD17B8](http://www.ncbi.nlm.nih.gov/gene/?term=7923)(22.373)[CTNNB1](http://www.ncbi.nlm.nih.gov/gene/?term=1499)(22.373)[P4HA3](http://www.ncbi.nlm.nih.gov/gene/?term=283208)(22.373) [EDA](http://www.ncbi.nlm.nih.gov/gene/?term=1896)(22.373)[HACL1](http://www.ncbi.nlm.nih.gov/gene/?term=26061)(22.373)[RYR2](http://www.ncbi.nlm.nih.gov/gene/?term=6262)(22.373)[HAP1](http://www.ncbi.nlm.nih.gov/gene/?term=9001)(22.373)[PCSK9](http://www.ncbi.nlm.nih.gov/gene/?term=255738)(22.373) [KCNQ1](http://www.ncbi.nlm.nih.gov/gene/?term=3784)(22.373)[CASQ2](http://www.ncbi.nlm.nih.gov/gene/?term=845)(22.373)[GJA5](http://www.ncbi.nlm.nih.gov/gene/?term=2702)(22.373)[DHRS9](http://www.ncbi.nlm.nih.gov/gene/?term=10170)(22.373)[GPR143](http://www.ncbi.nlm.nih.gov/gene/?term=4935)(22.373) [UGT1A1](http://www.ncbi.nlm.nih.gov/gene/?term=54658)(22.373)[DLD](http://www.ncbi.nlm.nih.gov/gene/?term=1738)(22.373)[GNAS](http://www.ncbi.nlm.nih.gov/gene/?term=2778)(22.373)[SLC25A13](http://www.ncbi.nlm.nih.gov/gene/?term=10165)(22.373)[SFXN5](http://www.ncbi.nlm.nih.gov/gene/?term=94097)(22.373) [PLOD2](http://www.ncbi.nlm.nih.gov/gene/?term=5352)(22.373)[CYP1A1](http://www.ncbi.nlm.nih.gov/gene/?term=1543)(22.373)[SULT2A1](http://www.ncbi.nlm.nih.gov/gene/?term=6822)(22.373)[NFX1](http://www.ncbi.nlm.nih.gov/gene/?term=4799)(22.373)[DBT](http://www.ncbi.nlm.nih.gov/gene/?term=1629)(22.373) [SLC25A1](http://www.ncbi.nlm.nih.gov/gene/?term=6576)(22.373) | |
| [Licoricesaponine K2](http://www.megabionet.org/tcmid/ingredient/31439/) | [GLRA3](http://www.ncbi.nlm.nih.gov/gene/?term=8001)(48.000)[NR3C1](http://www.ncbi.nlm.nih.gov/gene/?term=2908)(48.000)[GABRB3](http://www.ncbi.nlm.nih.gov/gene/?term=2562)(48.000) | |
| [Umbelliferone](http://www.megabionet.org/tcmid/ingredient/22179/) | [CRYZ](http://www.ncbi.nlm.nih.gov/gene/?term=1429)(80.882)[VKORC1](http://www.ncbi.nlm.nih.gov/gene/?term=79001)(80.882)[NQO1](http://www.ncbi.nlm.nih.gov/gene/?term=1728)(80.882)[CBR4](http://www.ncbi.nlm.nih.gov/gene/?term=84869)(55.444)[VKORC1L1](http://www.ncbi.nlm.nih.gov/gene/?term=154807)(55.444) [CYP4F2](http://www.ncbi.nlm.nih.gov/gene/?term=8529)(22.373)[CYP4F11](http://www.ncbi.nlm.nih.gov/gene/?term=57834)(22.373)[CRYZL1](http://www.ncbi.nlm.nih.gov/gene/?term=9946)(22.373)[SOD1](http://www.ncbi.nlm.nih.gov/gene/?term=6647)(22.373)[CCS](http://www.ncbi.nlm.nih.gov/gene/?term=9973)(22.373) [SOD3](http://www.ncbi.nlm.nih.gov/gene/?term=6649)(22.373)[SOD2](http://www.ncbi.nlm.nih.gov/gene/?term=6648)(22.373)[ADH4](http://www.ncbi.nlm.nih.gov/gene/?term=127)(22.373)[UBIAD1](http://www.ncbi.nlm.nih.gov/gene/?term=29914)(22.373)[CBR1](http://www.ncbi.nlm.nih.gov/gene/?term=873)(22.373) [TP53I3](http://www.ncbi.nlm.nih.gov/gene/?term=9540)(22.373) | |
| [Astragalin](http://www.megabionet.org/tcmid/ingredient/1935/) | This compound doesn't have any potential target with score larger than 20. | |
| [Octadecanoic Acid](http://www.megabionet.org/tcmid/ingredient/23678/) | [AKR1D1](http://www.ncbi.nlm.nih.gov/gene/?term=6718)(686.000)[TYR](http://www.ncbi.nlm.nih.gov/gene/?term=7299)(686.000)[SRD5A2](http://www.ncbi.nlm.nih.gov/gene/?term=6716)(686.000)[SCN11A](http://www.ncbi.nlm.nih.gov/gene/?term=11280)(122.778)[SCN2B](http://www.ncbi.nlm.nih.gov/gene/?term=6327)(122.778) [ABAT](http://www.ncbi.nlm.nih.gov/gene/?term=18)(122.778)[SCN1A](http://www.ncbi.nlm.nih.gov/gene/?term=6323)(122.778)[ESRRG](http://www.ncbi.nlm.nih.gov/gene/?term=2104)(122.778)[SCN3B](http://www.ncbi.nlm.nih.gov/gene/?term=55800)(122.778)[COX6C](http://www.ncbi.nlm.nih.gov/gene/?term=1345)(122.778) [COX5B](http://www.ncbi.nlm.nih.gov/gene/?term=1329)(122.778)[COX7C](http://www.ncbi.nlm.nih.gov/gene/?term=1350)(122.778)[COX1](http://www.ncbi.nlm.nih.gov/gene/?term=4512)(122.778)[SCN3A](http://www.ncbi.nlm.nih.gov/gene/?term=6328)(122.778)[AKR1C2](http://www.ncbi.nlm.nih.gov/gene/?term=1646)(122.778) [SCN7A](http://www.ncbi.nlm.nih.gov/gene/?term=6332)(122.778)[SCN5A](http://www.ncbi.nlm.nih.gov/gene/?term=6331)(122.778)[SCN10A](http://www.ncbi.nlm.nih.gov/gene/?term=6336)(122.778)[ALDH5A1](http://www.ncbi.nlm.nih.gov/gene/?term=7915)(122.778)[SCN2A](http://www.ncbi.nlm.nih.gov/gene/?term=6326)(122.778) [COX5A](http://www.ncbi.nlm.nih.gov/gene/?term=9377)(122.778)[COX3](http://www.ncbi.nlm.nih.gov/gene/?term=4514)(122.778)[AR](http://www.ncbi.nlm.nih.gov/gene/?term=367)(122.778)[COX7A1](http://www.ncbi.nlm.nih.gov/gene/?term=1346)(122.778)[FECH](http://www.ncbi.nlm.nih.gov/gene/?term=2235)(122.778) [COX4I1](http://www.ncbi.nlm.nih.gov/gene/?term=1327)(122.778)[HDAC9](http://www.ncbi.nlm.nih.gov/gene/?term=9734)(122.778)[PLA2G1B](http://www.ncbi.nlm.nih.gov/gene/?term=5319)(122.778)[COX6A2](http://www.ncbi.nlm.nih.gov/gene/?term=1339)(122.778)[SCN9A](http://www.ncbi.nlm.nih.gov/gene/?term=6335)(122.778) [SCN4A](http://www.ncbi.nlm.nih.gov/gene/?term=6329)(122.778)[SCN4B](http://www.ncbi.nlm.nih.gov/gene/?term=6330)(122.778)[ADH1C](http://www.ncbi.nlm.nih.gov/gene/?term=126)(122.778)[COX6B1](http://www.ncbi.nlm.nih.gov/gene/?term=1340)(122.778)[FABP6](http://www.ncbi.nlm.nih.gov/gene/?term=2172)(122.778) [CES1](http://www.ncbi.nlm.nih.gov/gene/?term=1066)(122.778)[ACADSB](http://www.ncbi.nlm.nih.gov/gene/?term=36)(122.778)[SCN8A](http://www.ncbi.nlm.nih.gov/gene/?term=6334)(122.778)[COX7B](http://www.ncbi.nlm.nih.gov/gene/?term=1349)(122.778)[SCN1B](http://www.ncbi.nlm.nih.gov/gene/?term=6324)(122.778) [COX2](http://www.ncbi.nlm.nih.gov/gene/?term=4513)(122.778)[OGDH](http://www.ncbi.nlm.nih.gov/gene/?term=4967)(122.778)[HDAC2](http://www.ncbi.nlm.nih.gov/gene/?term=3066)(122.778)[COX8A](http://www.ncbi.nlm.nih.gov/gene/?term=1351)(122.778)[NR1H4](http://www.ncbi.nlm.nih.gov/gene/?term=9971)(122.778) [SUCLG2](http://www.ncbi.nlm.nih.gov/gene/?term=8801)(80.882)[SLC13A2](http://www.ncbi.nlm.nih.gov/gene/?term=9058)(80.882)[SLC13A1](http://www.ncbi.nlm.nih.gov/gene/?term=6561)(80.882)[SUCLG1](http://www.ncbi.nlm.nih.gov/gene/?term=8802)(80.882)[HSD17B6](http://www.ncbi.nlm.nih.gov/gene/?term=8630)(80.882) [SLC25A10](http://www.ncbi.nlm.nih.gov/gene/?term=1468)(80.882)[P3H3](http://www.ncbi.nlm.nih.gov/gene/?term=10536)(80.882)[OXCT2](http://www.ncbi.nlm.nih.gov/gene/?term=64064)(80.882)[TMLHE](http://www.ncbi.nlm.nih.gov/gene/?term=55217)(80.882)[PLOD1](http://www.ncbi.nlm.nih.gov/gene/?term=5351)(80.882) [SUCNR1](http://www.ncbi.nlm.nih.gov/gene/?term=56670)(80.882)[SLC13A3](http://www.ncbi.nlm.nih.gov/gene/?term=64849)(80.882)[ASPH](http://www.ncbi.nlm.nih.gov/gene/?term=444)(80.882)[BBOX1](http://www.ncbi.nlm.nih.gov/gene/?term=8424)(80.882)[SDHB](http://www.ncbi.nlm.nih.gov/gene/?term=6390)(80.882) [OXCT1](http://www.ncbi.nlm.nih.gov/gene/?term=5019)(80.882)[PLOD3](http://www.ncbi.nlm.nih.gov/gene/?term=8985)(80.882)[P4HA1](http://www.ncbi.nlm.nih.gov/gene/?term=5033)(80.882)[SDHC](http://www.ncbi.nlm.nih.gov/gene/?term=6391)(80.882)[P3H2](http://www.ncbi.nlm.nih.gov/gene/?term=55214)(80.882) [SDHA](http://www.ncbi.nlm.nih.gov/gene/?term=6389)(80.882)[P4HA2](http://www.ncbi.nlm.nih.gov/gene/?term=8974)(80.882)[SUCLA2](http://www.ncbi.nlm.nih.gov/gene/?term=8803)(80.882)[SDHD](http://www.ncbi.nlm.nih.gov/gene/?term=6392)(80.882)[P3H1](http://www.ncbi.nlm.nih.gov/gene/?term=64175)(80.882) [DCT](http://www.ncbi.nlm.nih.gov/gene/?term=1638)(55.444)[AKR1C3](http://www.ncbi.nlm.nih.gov/gene/?term=8644)(55.444)[CRTAP](http://www.ncbi.nlm.nih.gov/gene/?term=10491)(55.444)[SLIT2](http://www.ncbi.nlm.nih.gov/gene/?term=9353)(55.444)[ACO2](http://www.ncbi.nlm.nih.gov/gene/?term=50)(55.444) [JMJD6](http://www.ncbi.nlm.nih.gov/gene/?term=23210)(55.444)[SLC13A4](http://www.ncbi.nlm.nih.gov/gene/?term=26266)(55.444)[SDHAF2](http://www.ncbi.nlm.nih.gov/gene/?term=54949)(55.444)[SLC1A3](http://www.ncbi.nlm.nih.gov/gene/?term=6507)(55.444)[SALL1](http://www.ncbi.nlm.nih.gov/gene/?term=6299)(55.444) [HSD17B11](http://www.ncbi.nlm.nih.gov/gene/?term=51170)(55.444)[TYRP1](http://www.ncbi.nlm.nih.gov/gene/?term=7306)(55.444)[HIF1AN](http://www.ncbi.nlm.nih.gov/gene/?term=55662)(55.444)[UROS](http://www.ncbi.nlm.nih.gov/gene/?term=7390)(55.444)[SLC13A5](http://www.ncbi.nlm.nih.gov/gene/?term=284111)(55.444) [P4HB](http://www.ncbi.nlm.nih.gov/gene/?term=5034)(55.444)[CACNA2D1](http://www.ncbi.nlm.nih.gov/gene/?term=781)(48.000)[PLAT](http://www.ncbi.nlm.nih.gov/gene/?term=5327)(48.000)[SLC7A2](http://www.ncbi.nlm.nih.gov/gene/?term=6542)(48.000)[CACNA1A](http://www.ncbi.nlm.nih.gov/gene/?term=773)(48.000) [GRIN3B](http://www.ncbi.nlm.nih.gov/gene/?term=116444)(48.000)[CACNA2D2](http://www.ncbi.nlm.nih.gov/gene/?term=9254)(48.000)[GRIN2A](http://www.ncbi.nlm.nih.gov/gene/?term=2903)(48.000)[TOP1](http://www.ncbi.nlm.nih.gov/gene/?term=7150)(48.000)[PLG](http://www.ncbi.nlm.nih.gov/gene/?term=5340)(48.000) [CACNA1B](http://www.ncbi.nlm.nih.gov/gene/?term=774)(48.000)[GRIN2C](http://www.ncbi.nlm.nih.gov/gene/?term=2905)(48.000)[GRIN2B](http://www.ncbi.nlm.nih.gov/gene/?term=2904)(48.000)[SLC7A3](http://www.ncbi.nlm.nih.gov/gene/?term=84889)(48.000)[KARS](http://www.ncbi.nlm.nih.gov/gene/?term=3735)(48.000) [GRIN3A](http://www.ncbi.nlm.nih.gov/gene/?term=116443)(48.000)[SLC7A1](http://www.ncbi.nlm.nih.gov/gene/?term=6541)(48.000)[SLC7A4](http://www.ncbi.nlm.nih.gov/gene/?term=6545)(48.000)[GRIN2D](http://www.ncbi.nlm.nih.gov/gene/?term=2906)(48.000)[ADORA1](http://www.ncbi.nlm.nih.gov/gene/?term=134)(48.000) [GRIN1](http://www.ncbi.nlm.nih.gov/gene/?term=2902)(48.000)[HDAC1](http://www.ncbi.nlm.nih.gov/gene/?term=3065)(23.000)[GPD1L](http://www.ncbi.nlm.nih.gov/gene/?term=23171)(22.373)[SRD5A1](http://www.ncbi.nlm.nih.gov/gene/?term=6715)(22.373)[FBXO45](http://www.ncbi.nlm.nih.gov/gene/?term=200933)(22.373) [SIRT1](http://www.ncbi.nlm.nih.gov/gene/?term=23411)(22.373)[SHMT1](http://www.ncbi.nlm.nih.gov/gene/?term=6470)(22.373)[SUGCT](http://www.ncbi.nlm.nih.gov/gene/?term=79783)(22.373)[ACSS2](http://www.ncbi.nlm.nih.gov/gene/?term=55902)(22.373)[ARV1](http://www.ncbi.nlm.nih.gov/gene/?term=64801)(22.373) [SIX1](http://www.ncbi.nlm.nih.gov/gene/?term=6495)(22.373)[BDH1](http://www.ncbi.nlm.nih.gov/gene/?term=622)(22.373)[COLGALT2](http://www.ncbi.nlm.nih.gov/gene/?term=23127)(22.373)[SIX4](http://www.ncbi.nlm.nih.gov/gene/?term=51804)(22.373)[MRPS36](http://www.ncbi.nlm.nih.gov/gene/?term=92259)(22.373) [OSBPL8](http://www.ncbi.nlm.nih.gov/gene/?term=114882)(22.373)[SPI1](http://www.ncbi.nlm.nih.gov/gene/?term=6688)(22.373)[SUOX](http://www.ncbi.nlm.nih.gov/gene/?term=6821)(22.373)[CCL2](http://www.ncbi.nlm.nih.gov/gene/?term=6347)(22.373)[BCKDK](http://www.ncbi.nlm.nih.gov/gene/?term=10295)(22.373) [UBE2B](http://www.ncbi.nlm.nih.gov/gene/?term=7320)(22.373)[CYP1A2](http://www.ncbi.nlm.nih.gov/gene/?term=1544)(22.373)[CCL5](http://www.ncbi.nlm.nih.gov/gene/?term=6352)(22.373)[ANK2](http://www.ncbi.nlm.nih.gov/gene/?term=287)(22.373)[HSD17B2](http://www.ncbi.nlm.nih.gov/gene/?term=3294)(22.373) [STIM2](http://www.ncbi.nlm.nih.gov/gene/?term=57620)(22.373)[PKD2](http://www.ncbi.nlm.nih.gov/gene/?term=5311)(22.373)[ALDH9A1](http://www.ncbi.nlm.nih.gov/gene/?term=223)(22.373)[TH](http://www.ncbi.nlm.nih.gov/gene/?term=7054)(22.373)[EPO](http://www.ncbi.nlm.nih.gov/gene/?term=2056)(22.373) [COLGALT1](http://www.ncbi.nlm.nih.gov/gene/?term=79709)(22.373)[NEDD4](http://www.ncbi.nlm.nih.gov/gene/?term=4734)(22.373)[CYP11A1](http://www.ncbi.nlm.nih.gov/gene/?term=1583)(22.373)[DHODH](http://www.ncbi.nlm.nih.gov/gene/?term=1723)(22.373)[CLN3](http://www.ncbi.nlm.nih.gov/gene/?term=1201)(22.373) [ARID1A](http://www.ncbi.nlm.nih.gov/gene/?term=8289)(22.373)[EGLN2](http://www.ncbi.nlm.nih.gov/gene/?term=112398)(22.373)[ACAT1](http://www.ncbi.nlm.nih.gov/gene/?term=38)(22.373)[GALC](http://www.ncbi.nlm.nih.gov/gene/?term=2581)(22.373)[PLCG2](http://www.ncbi.nlm.nih.gov/gene/?term=5336)(22.373) [ACOT4](http://www.ncbi.nlm.nih.gov/gene/?term=122970)(22.373)[PHGDH](http://www.ncbi.nlm.nih.gov/gene/?term=26227)(22.373)[PNLIPRP2](http://www.ncbi.nlm.nih.gov/gene/?term=5408)(22.373)[CYP39A1](http://www.ncbi.nlm.nih.gov/gene/?term=51302)(22.373)[BCKDHA](http://www.ncbi.nlm.nih.gov/gene/?term=593)(22.373) [SLC25A12](http://www.ncbi.nlm.nih.gov/gene/?term=8604)(22.373)[YBX3](http://www.ncbi.nlm.nih.gov/gene/?term=8531)(22.373)[8-Mar](http://www.ncbi.nlm.nih.gov/gene/?term=220972)(22.373)[STIM1](http://www.ncbi.nlm.nih.gov/gene/?term=6786)(22.373)[GAL3ST1](http://www.ncbi.nlm.nih.gov/gene/?term=9514)(22.373) [CDH11](http://www.ncbi.nlm.nih.gov/gene/?term=1009)(22.373)[CACNA1D](http://www.ncbi.nlm.nih.gov/gene/?term=776)(22.373)[DDC](http://www.ncbi.nlm.nih.gov/gene/?term=1644)(22.373)[DHTKD1](http://www.ncbi.nlm.nih.gov/gene/?term=55526)(22.373)[NEDD4L](http://www.ncbi.nlm.nih.gov/gene/?term=23327)(22.373) [BDKRB2](http://www.ncbi.nlm.nih.gov/gene/?term=624)(22.373)[PRPH](http://www.ncbi.nlm.nih.gov/gene/?term=5630)(22.373)[BCKDHB](http://www.ncbi.nlm.nih.gov/gene/?term=594)(22.373)[KCNE5](http://www.ncbi.nlm.nih.gov/gene/?term=23630)(22.373)[SRD5A3](http://www.ncbi.nlm.nih.gov/gene/?term=79644)(22.373) [SMAD7](http://www.ncbi.nlm.nih.gov/gene/?term=4092)(22.373)[PRDM8](http://www.ncbi.nlm.nih.gov/gene/?term=56978)(22.373)[UROD](http://www.ncbi.nlm.nih.gov/gene/?term=7389)(22.373)[UGT8](http://www.ncbi.nlm.nih.gov/gene/?term=7368)(22.373)[ETHE1](http://www.ncbi.nlm.nih.gov/gene/?term=23474)(22.373) [WNT10B](http://www.ncbi.nlm.nih.gov/gene/?term=7480)(22.373)[OGFOD1](http://www.ncbi.nlm.nih.gov/gene/?term=55239)(22.373)[SLC38A7](http://www.ncbi.nlm.nih.gov/gene/?term=55238)(22.373)[ACSS1](http://www.ncbi.nlm.nih.gov/gene/?term=84532)(22.373)[CYP4B1](http://www.ncbi.nlm.nih.gov/gene/?term=1580)(22.373) [STAR](http://www.ncbi.nlm.nih.gov/gene/?term=6770)(22.373)[DLST](http://www.ncbi.nlm.nih.gov/gene/?term=1743)(22.373)[PF4](http://www.ncbi.nlm.nih.gov/gene/?term=5196)(22.373)[CRACR2A](http://www.ncbi.nlm.nih.gov/gene/?term=84766)(22.373)[HSD17B1](http://www.ncbi.nlm.nih.gov/gene/?term=3292)(22.373) [CAMK2D](http://www.ncbi.nlm.nih.gov/gene/?term=817)(22.373)[AP3D1](http://www.ncbi.nlm.nih.gov/gene/?term=8943)(22.373)[MYO5A](http://www.ncbi.nlm.nih.gov/gene/?term=4644)(22.373)[ILVBL](http://www.ncbi.nlm.nih.gov/gene/?term=10994)(22.373)[CAV3](http://www.ncbi.nlm.nih.gov/gene/?term=859)(22.373) [HTT](http://www.ncbi.nlm.nih.gov/gene/?term=3064)(22.373)[IL10](http://www.ncbi.nlm.nih.gov/gene/?term=3586)(22.373)[EGLN3](http://www.ncbi.nlm.nih.gov/gene/?term=112399)(22.373)[OGDHL](http://www.ncbi.nlm.nih.gov/gene/?term=55753)(22.373)[KCNA5](http://www.ncbi.nlm.nih.gov/gene/?term=3741)(22.373) [TST](http://www.ncbi.nlm.nih.gov/gene/?term=7263)(22.373)[SLC1A6](http://www.ncbi.nlm.nih.gov/gene/?term=6511)(22.373)[NPPA](http://www.ncbi.nlm.nih.gov/gene/?term=4878)(22.373)[ACADM](http://www.ncbi.nlm.nih.gov/gene/?term=34)(22.373)[SQRDL](http://www.ncbi.nlm.nih.gov/gene/?term=58472)(22.373) [TMEM110](http://www.ncbi.nlm.nih.gov/gene/?term=375346)(22.373)[CDC42](http://www.ncbi.nlm.nih.gov/gene/?term=998)(22.373)[ARHGEF2](http://www.ncbi.nlm.nih.gov/gene/?term=9181)(22.373)[EGLN1](http://www.ncbi.nlm.nih.gov/gene/?term=54583)(22.373)[ETFDH](http://www.ncbi.nlm.nih.gov/gene/?term=2110)(22.373) [EPHA4](http://www.ncbi.nlm.nih.gov/gene/?term=2043)(22.373)[OCA2](http://www.ncbi.nlm.nih.gov/gene/?term=4948)(22.373)[HSD17B8](http://www.ncbi.nlm.nih.gov/gene/?term=7923)(22.373)[CTNNB1](http://www.ncbi.nlm.nih.gov/gene/?term=1499)(22.373)[P4HA3](http://www.ncbi.nlm.nih.gov/gene/?term=283208)(22.373) [EDA](http://www.ncbi.nlm.nih.gov/gene/?term=1896)(22.373)[HACL1](http://www.ncbi.nlm.nih.gov/gene/?term=26061)(22.373)[RYR2](http://www.ncbi.nlm.nih.gov/gene/?term=6262)(22.373)[HAP1](http://www.ncbi.nlm.nih.gov/gene/?term=9001)(22.373)[PCSK9](http://www.ncbi.nlm.nih.gov/gene/?term=255738)(22.373) [KCNQ1](http://www.ncbi.nlm.nih.gov/gene/?term=3784)(22.373)[CASQ2](http://www.ncbi.nlm.nih.gov/gene/?term=845)(22.373)[GJA5](http://www.ncbi.nlm.nih.gov/gene/?term=2702)(22.373)[DHRS9](http://www.ncbi.nlm.nih.gov/gene/?term=10170)(22.373)[GPR143](http://www.ncbi.nlm.nih.gov/gene/?term=4935)(22.373) [UGT1A1](http://www.ncbi.nlm.nih.gov/gene/?term=54658)(22.373)[DLD](http://www.ncbi.nlm.nih.gov/gene/?term=1738)(22.373)[GNAS](http://www.ncbi.nlm.nih.gov/gene/?term=2778)(22.373)[SLC25A13](http://www.ncbi.nlm.nih.gov/gene/?term=10165)(22.373)[SFXN5](http://www.ncbi.nlm.nih.gov/gene/?term=94097)(22.373) [PLOD2](http://www.ncbi.nlm.nih.gov/gene/?term=5352)(22.373)[CYP1A1](http://www.ncbi.nlm.nih.gov/gene/?term=1543)(22.373)[SULT2A1](http://www.ncbi.nlm.nih.gov/gene/?term=6822)(22.373)[NFX1](http://www.ncbi.nlm.nih.gov/gene/?term=4799)(22.373)[DBT](http://www.ncbi.nlm.nih.gov/gene/?term=1629)(22.373) [SLC25A1](http://www.ncbi.nlm.nih.gov/gene/?term=6576)(22.373) | |
| [Gamma-Sitosterol](http://www.megabionet.org/tcmid/ingredient/29509/) | [ESR1](http://www.ncbi.nlm.nih.gov/gene/?term=2099)(122.778)[PGR](http://www.ncbi.nlm.nih.gov/gene/?term=5241)(122.778)[VDR](http://www.ncbi.nlm.nih.gov/gene/?term=7421)(80.882)[CYP27B1](http://www.ncbi.nlm.nih.gov/gene/?term=1594)(80.882)[GC](http://www.ncbi.nlm.nih.gov/gene/?term=2638)(55.444) [SNW1](http://www.ncbi.nlm.nih.gov/gene/?term=22938)(55.444)[AR](http://www.ncbi.nlm.nih.gov/gene/?term=367)(48.000)[NR3C1](http://www.ncbi.nlm.nih.gov/gene/?term=2908)(48.000)[NFKB1](http://www.ncbi.nlm.nih.gov/gene/?term=4790)(22.373)[AKR1C3](http://www.ncbi.nlm.nih.gov/gene/?term=8644)(22.373) [CYP24A1](http://www.ncbi.nlm.nih.gov/gene/?term=1591)(22.373)[GPBAR1](http://www.ncbi.nlm.nih.gov/gene/?term=151306)(22.373)[SNAI2](http://www.ncbi.nlm.nih.gov/gene/?term=6591)(22.373)[MED1](http://www.ncbi.nlm.nih.gov/gene/?term=5469)(22.373)[SNAI1](http://www.ncbi.nlm.nih.gov/gene/?term=6615)(22.373) [CYP3A4](http://www.ncbi.nlm.nih.gov/gene/?term=1576)(22.373)[CALB1](http://www.ncbi.nlm.nih.gov/gene/?term=793)(22.373)[FGF23](http://www.ncbi.nlm.nih.gov/gene/?term=8074)(22.373)[GFI1](http://www.ncbi.nlm.nih.gov/gene/?term=2672)(22.373)[LANCL2](http://www.ncbi.nlm.nih.gov/gene/?term=55915)(22.373) [WNT4](http://www.ncbi.nlm.nih.gov/gene/?term=54361)(22.373)[TCF3](http://www.ncbi.nlm.nih.gov/gene/?term=6929)(22.373)[BAX](http://www.ncbi.nlm.nih.gov/gene/?term=581)(22.373)[KL](http://www.ncbi.nlm.nih.gov/gene/?term=9365)(22.373)[CYP2R1](http://www.ncbi.nlm.nih.gov/gene/?term=120227)(22.373) [PML](http://www.ncbi.nlm.nih.gov/gene/?term=5371)(22.373)[B4GALT1](http://www.ncbi.nlm.nih.gov/gene/?term=2683)(22.373)[S100G](http://www.ncbi.nlm.nih.gov/gene/?term=795)(22.373)[CYP27A1](http://www.ncbi.nlm.nih.gov/gene/?term=1593)(22.373)[KANK2](http://www.ncbi.nlm.nih.gov/gene/?term=25959)(22.373) [IRX5](http://www.ncbi.nlm.nih.gov/gene/?term=10265)(22.373)[RXRA](http://www.ncbi.nlm.nih.gov/gene/?term=6256)(22.373)[NR1H4](http://www.ncbi.nlm.nih.gov/gene/?term=9971)(22.373)[TRIM24](http://www.ncbi.nlm.nih.gov/gene/?term=8805)(22.373) | |
| [Pentadecanoic Acid](http://www.megabionet.org/tcmid/ingredient/23379/) | [AKR1D1](http://www.ncbi.nlm.nih.gov/gene/?term=6718)(686.000)[TYR](http://www.ncbi.nlm.nih.gov/gene/?term=7299)(686.000)[SRD5A2](http://www.ncbi.nlm.nih.gov/gene/?term=6716)(686.000)[SCN11A](http://www.ncbi.nlm.nih.gov/gene/?term=11280)(122.778)[SCN2B](http://www.ncbi.nlm.nih.gov/gene/?term=6327)(122.778) [ABAT](http://www.ncbi.nlm.nih.gov/gene/?term=18)(122.778)[SCN1A](http://www.ncbi.nlm.nih.gov/gene/?term=6323)(122.778)[ESRRG](http://www.ncbi.nlm.nih.gov/gene/?term=2104)(122.778)[SCN3B](http://www.ncbi.nlm.nih.gov/gene/?term=55800)(122.778)[COX6C](http://www.ncbi.nlm.nih.gov/gene/?term=1345)(122.778) [COX5B](http://www.ncbi.nlm.nih.gov/gene/?term=1329)(122.778)[COX7C](http://www.ncbi.nlm.nih.gov/gene/?term=1350)(122.778)[COX1](http://www.ncbi.nlm.nih.gov/gene/?term=4512)(122.778)[SCN3A](http://www.ncbi.nlm.nih.gov/gene/?term=6328)(122.778)[AKR1C2](http://www.ncbi.nlm.nih.gov/gene/?term=1646)(122.778) [SCN7A](http://www.ncbi.nlm.nih.gov/gene/?term=6332)(122.778)[SCN5A](http://www.ncbi.nlm.nih.gov/gene/?term=6331)(122.778)[SCN10A](http://www.ncbi.nlm.nih.gov/gene/?term=6336)(122.778)[ALDH5A1](http://www.ncbi.nlm.nih.gov/gene/?term=7915)(122.778)[SCN2A](http://www.ncbi.nlm.nih.gov/gene/?term=6326)(122.778) [COX5A](http://www.ncbi.nlm.nih.gov/gene/?term=9377)(122.778)[COX3](http://www.ncbi.nlm.nih.gov/gene/?term=4514)(122.778)[AR](http://www.ncbi.nlm.nih.gov/gene/?term=367)(122.778)[COX7A1](http://www.ncbi.nlm.nih.gov/gene/?term=1346)(122.778)[FECH](http://www.ncbi.nlm.nih.gov/gene/?term=2235)(122.778) [COX4I1](http://www.ncbi.nlm.nih.gov/gene/?term=1327)(122.778)[HDAC9](http://www.ncbi.nlm.nih.gov/gene/?term=9734)(122.778)[PLA2G1B](http://www.ncbi.nlm.nih.gov/gene/?term=5319)(122.778)[COX6A2](http://www.ncbi.nlm.nih.gov/gene/?term=1339)(122.778)[SCN9A](http://www.ncbi.nlm.nih.gov/gene/?term=6335)(122.778) [SCN4A](http://www.ncbi.nlm.nih.gov/gene/?term=6329)(122.778)[SCN4B](http://www.ncbi.nlm.nih.gov/gene/?term=6330)(122.778)[ADH1C](http://www.ncbi.nlm.nih.gov/gene/?term=126)(122.778)[COX6B1](http://www.ncbi.nlm.nih.gov/gene/?term=1340)(122.778)[FABP6](http://www.ncbi.nlm.nih.gov/gene/?term=2172)(122.778) [CES1](http://www.ncbi.nlm.nih.gov/gene/?term=1066)(122.778)[ACADSB](http://www.ncbi.nlm.nih.gov/gene/?term=36)(122.778)[SCN8A](http://www.ncbi.nlm.nih.gov/gene/?term=6334)(122.778)[COX7B](http://www.ncbi.nlm.nih.gov/gene/?term=1349)(122.778)[SCN1B](http://www.ncbi.nlm.nih.gov/gene/?term=6324)(122.778) [COX2](http://www.ncbi.nlm.nih.gov/gene/?term=4513)(122.778)[OGDH](http://www.ncbi.nlm.nih.gov/gene/?term=4967)(122.778)[HDAC2](http://www.ncbi.nlm.nih.gov/gene/?term=3066)(122.778)[COX8A](http://www.ncbi.nlm.nih.gov/gene/?term=1351)(122.778)[NR1H4](http://www.ncbi.nlm.nih.gov/gene/?term=9971)(122.778) [SUCLG2](http://www.ncbi.nlm.nih.gov/gene/?term=8801)(80.882)[SLC13A2](http://www.ncbi.nlm.nih.gov/gene/?term=9058)(80.882)[SLC13A1](http://www.ncbi.nlm.nih.gov/gene/?term=6561)(80.882)[SUCLG1](http://www.ncbi.nlm.nih.gov/gene/?term=8802)(80.882)[HSD17B6](http://www.ncbi.nlm.nih.gov/gene/?term=8630)(80.882) [SLC25A10](http://www.ncbi.nlm.nih.gov/gene/?term=1468)(80.882)[P3H3](http://www.ncbi.nlm.nih.gov/gene/?term=10536)(80.882)[OXCT2](http://www.ncbi.nlm.nih.gov/gene/?term=64064)(80.882)[TMLHE](http://www.ncbi.nlm.nih.gov/gene/?term=55217)(80.882)[PLOD1](http://www.ncbi.nlm.nih.gov/gene/?term=5351)(80.882) [SUCNR1](http://www.ncbi.nlm.nih.gov/gene/?term=56670)(80.882)[SLC13A3](http://www.ncbi.nlm.nih.gov/gene/?term=64849)(80.882)[ASPH](http://www.ncbi.nlm.nih.gov/gene/?term=444)(80.882)[BBOX1](http://www.ncbi.nlm.nih.gov/gene/?term=8424)(80.882)[SDHB](http://www.ncbi.nlm.nih.gov/gene/?term=6390)(80.882) [OXCT1](http://www.ncbi.nlm.nih.gov/gene/?term=5019)(80.882)[PLOD3](http://www.ncbi.nlm.nih.gov/gene/?term=8985)(80.882)[P4HA1](http://www.ncbi.nlm.nih.gov/gene/?term=5033)(80.882)[SDHC](http://www.ncbi.nlm.nih.gov/gene/?term=6391)(80.882)[P3H2](http://www.ncbi.nlm.nih.gov/gene/?term=55214)(80.882) [SDHA](http://www.ncbi.nlm.nih.gov/gene/?term=6389)(80.882)[P4HA2](http://www.ncbi.nlm.nih.gov/gene/?term=8974)(80.882)[SUCLA2](http://www.ncbi.nlm.nih.gov/gene/?term=8803)(80.882)[SDHD](http://www.ncbi.nlm.nih.gov/gene/?term=6392)(80.882)[P3H1](http://www.ncbi.nlm.nih.gov/gene/?term=64175)(80.882) [DCT](http://www.ncbi.nlm.nih.gov/gene/?term=1638)(55.444)[AKR1C3](http://www.ncbi.nlm.nih.gov/gene/?term=8644)(55.444)[CRTAP](http://www.ncbi.nlm.nih.gov/gene/?term=10491)(55.444)[SLIT2](http://www.ncbi.nlm.nih.gov/gene/?term=9353)(55.444)[ACO2](http://www.ncbi.nlm.nih.gov/gene/?term=50)(55.444) [JMJD6](http://www.ncbi.nlm.nih.gov/gene/?term=23210)(55.444)[SLC13A4](http://www.ncbi.nlm.nih.gov/gene/?term=26266)(55.444)[SDHAF2](http://www.ncbi.nlm.nih.gov/gene/?term=54949)(55.444)[SLC1A3](http://www.ncbi.nlm.nih.gov/gene/?term=6507)(55.444)[SALL1](http://www.ncbi.nlm.nih.gov/gene/?term=6299)(55.444) [HSD17B11](http://www.ncbi.nlm.nih.gov/gene/?term=51170)(55.444)[TYRP1](http://www.ncbi.nlm.nih.gov/gene/?term=7306)(55.444)[HIF1AN](http://www.ncbi.nlm.nih.gov/gene/?term=55662)(55.444)[UROS](http://www.ncbi.nlm.nih.gov/gene/?term=7390)(55.444)[SLC13A5](http://www.ncbi.nlm.nih.gov/gene/?term=284111)(55.444) [P4HB](http://www.ncbi.nlm.nih.gov/gene/?term=5034)(55.444)[CACNA2D1](http://www.ncbi.nlm.nih.gov/gene/?term=781)(48.000)[PLAT](http://www.ncbi.nlm.nih.gov/gene/?term=5327)(48.000)[SLC7A2](http://www.ncbi.nlm.nih.gov/gene/?term=6542)(48.000)[CACNA1A](http://www.ncbi.nlm.nih.gov/gene/?term=773)(48.000) [GRIN3B](http://www.ncbi.nlm.nih.gov/gene/?term=116444)(48.000)[CACNA2D2](http://www.ncbi.nlm.nih.gov/gene/?term=9254)(48.000)[GRIN2A](http://www.ncbi.nlm.nih.gov/gene/?term=2903)(48.000)[TOP1](http://www.ncbi.nlm.nih.gov/gene/?term=7150)(48.000)[PLG](http://www.ncbi.nlm.nih.gov/gene/?term=5340)(48.000) [CACNA1B](http://www.ncbi.nlm.nih.gov/gene/?term=774)(48.000)[GRIN2C](http://www.ncbi.nlm.nih.gov/gene/?term=2905)(48.000)[GRIN2B](http://www.ncbi.nlm.nih.gov/gene/?term=2904)(48.000)[SLC7A3](http://www.ncbi.nlm.nih.gov/gene/?term=84889)(48.000)[KARS](http://www.ncbi.nlm.nih.gov/gene/?term=3735)(48.000) [GRIN3A](http://www.ncbi.nlm.nih.gov/gene/?term=116443)(48.000)[SLC7A1](http://www.ncbi.nlm.nih.gov/gene/?term=6541)(48.000)[SLC7A4](http://www.ncbi.nlm.nih.gov/gene/?term=6545)(48.000)[GRIN2D](http://www.ncbi.nlm.nih.gov/gene/?term=2906)(48.000)[ADORA1](http://www.ncbi.nlm.nih.gov/gene/?term=134)(48.000) [GRIN1](http://www.ncbi.nlm.nih.gov/gene/?term=2902)(48.000)[HDAC1](http://www.ncbi.nlm.nih.gov/gene/?term=3065)(23.000)[GPD1L](http://www.ncbi.nlm.nih.gov/gene/?term=23171)(22.373)[SRD5A1](http://www.ncbi.nlm.nih.gov/gene/?term=6715)(22.373)[FBXO45](http://www.ncbi.nlm.nih.gov/gene/?term=200933)(22.373) [SIRT1](http://www.ncbi.nlm.nih.gov/gene/?term=23411)(22.373)[SHMT1](http://www.ncbi.nlm.nih.gov/gene/?term=6470)(22.373)[SUGCT](http://www.ncbi.nlm.nih.gov/gene/?term=79783)(22.373)[ACSS2](http://www.ncbi.nlm.nih.gov/gene/?term=55902)(22.373)[ARV1](http://www.ncbi.nlm.nih.gov/gene/?term=64801)(22.373) [SIX1](http://www.ncbi.nlm.nih.gov/gene/?term=6495)(22.373)[BDH1](http://www.ncbi.nlm.nih.gov/gene/?term=622)(22.373)[COLGALT2](http://www.ncbi.nlm.nih.gov/gene/?term=23127)(22.373)[SIX4](http://www.ncbi.nlm.nih.gov/gene/?term=51804)(22.373)[MRPS36](http://www.ncbi.nlm.nih.gov/gene/?term=92259)(22.373) [OSBPL8](http://www.ncbi.nlm.nih.gov/gene/?term=114882)(22.373)[SPI1](http://www.ncbi.nlm.nih.gov/gene/?term=6688)(22.373)[SUOX](http://www.ncbi.nlm.nih.gov/gene/?term=6821)(22.373)[CCL2](http://www.ncbi.nlm.nih.gov/gene/?term=6347)(22.373)[BCKDK](http://www.ncbi.nlm.nih.gov/gene/?term=10295)(22.373) [UBE2B](http://www.ncbi.nlm.nih.gov/gene/?term=7320)(22.373)[CYP1A2](http://www.ncbi.nlm.nih.gov/gene/?term=1544)(22.373)[CCL5](http://www.ncbi.nlm.nih.gov/gene/?term=6352)(22.373)[ANK2](http://www.ncbi.nlm.nih.gov/gene/?term=287)(22.373)[HSD17B2](http://www.ncbi.nlm.nih.gov/gene/?term=3294)(22.373) [STIM2](http://www.ncbi.nlm.nih.gov/gene/?term=57620)(22.373)[PKD2](http://www.ncbi.nlm.nih.gov/gene/?term=5311)(22.373)[ALDH9A1](http://www.ncbi.nlm.nih.gov/gene/?term=223)(22.373)[TH](http://www.ncbi.nlm.nih.gov/gene/?term=7054)(22.373)[EPO](http://www.ncbi.nlm.nih.gov/gene/?term=2056)(22.373) [COLGALT1](http://www.ncbi.nlm.nih.gov/gene/?term=79709)(22.373)[NEDD4](http://www.ncbi.nlm.nih.gov/gene/?term=4734)(22.373)[CYP11A1](http://www.ncbi.nlm.nih.gov/gene/?term=1583)(22.373)[DHODH](http://www.ncbi.nlm.nih.gov/gene/?term=1723)(22.373)[CLN3](http://www.ncbi.nlm.nih.gov/gene/?term=1201)(22.373) [ARID1A](http://www.ncbi.nlm.nih.gov/gene/?term=8289)(22.373)[EGLN2](http://www.ncbi.nlm.nih.gov/gene/?term=112398)(22.373)[ACAT1](http://www.ncbi.nlm.nih.gov/gene/?term=38)(22.373)[GALC](http://www.ncbi.nlm.nih.gov/gene/?term=2581)(22.373)[PLCG2](http://www.ncbi.nlm.nih.gov/gene/?term=5336)(22.373) [ACOT4](http://www.ncbi.nlm.nih.gov/gene/?term=122970)(22.373)[PHGDH](http://www.ncbi.nlm.nih.gov/gene/?term=26227)(22.373)[PNLIPRP2](http://www.ncbi.nlm.nih.gov/gene/?term=5408)(22.373)[CYP39A1](http://www.ncbi.nlm.nih.gov/gene/?term=51302)(22.373)[BCKDHA](http://www.ncbi.nlm.nih.gov/gene/?term=593)(22.373) [SLC25A12](http://www.ncbi.nlm.nih.gov/gene/?term=8604)(22.373)[YBX3](http://www.ncbi.nlm.nih.gov/gene/?term=8531)(22.373)[8-Mar](http://www.ncbi.nlm.nih.gov/gene/?term=220972)(22.373)[STIM1](http://www.ncbi.nlm.nih.gov/gene/?term=6786)(22.373)[GAL3ST1](http://www.ncbi.nlm.nih.gov/gene/?term=9514)(22.373) [CDH11](http://www.ncbi.nlm.nih.gov/gene/?term=1009)(22.373)[CACNA1D](http://www.ncbi.nlm.nih.gov/gene/?term=776)(22.373)[DDC](http://www.ncbi.nlm.nih.gov/gene/?term=1644)(22.373)[DHTKD1](http://www.ncbi.nlm.nih.gov/gene/?term=55526)(22.373)[NEDD4L](http://www.ncbi.nlm.nih.gov/gene/?term=23327)(22.373) [BDKRB2](http://www.ncbi.nlm.nih.gov/gene/?term=624)(22.373)[PRPH](http://www.ncbi.nlm.nih.gov/gene/?term=5630)(22.373)[BCKDHB](http://www.ncbi.nlm.nih.gov/gene/?term=594)(22.373)[KCNE5](http://www.ncbi.nlm.nih.gov/gene/?term=23630)(22.373)[SRD5A3](http://www.ncbi.nlm.nih.gov/gene/?term=79644)(22.373) [SMAD7](http://www.ncbi.nlm.nih.gov/gene/?term=4092)(22.373)[PRDM8](http://www.ncbi.nlm.nih.gov/gene/?term=56978)(22.373)[UROD](http://www.ncbi.nlm.nih.gov/gene/?term=7389)(22.373)[UGT8](http://www.ncbi.nlm.nih.gov/gene/?term=7368)(22.373)[ETHE1](http://www.ncbi.nlm.nih.gov/gene/?term=23474)(22.373) [WNT10B](http://www.ncbi.nlm.nih.gov/gene/?term=7480)(22.373)[OGFOD1](http://www.ncbi.nlm.nih.gov/gene/?term=55239)(22.373)[SLC38A7](http://www.ncbi.nlm.nih.gov/gene/?term=55238)(22.373)[ACSS1](http://www.ncbi.nlm.nih.gov/gene/?term=84532)(22.373)[CYP4B1](http://www.ncbi.nlm.nih.gov/gene/?term=1580)(22.373) [STAR](http://www.ncbi.nlm.nih.gov/gene/?term=6770)(22.373)[DLST](http://www.ncbi.nlm.nih.gov/gene/?term=1743)(22.373)[PF4](http://www.ncbi.nlm.nih.gov/gene/?term=5196)(22.373)[CRACR2A](http://www.ncbi.nlm.nih.gov/gene/?term=84766)(22.373)[HSD17B1](http://www.ncbi.nlm.nih.gov/gene/?term=3292)(22.373) [CAMK2D](http://www.ncbi.nlm.nih.gov/gene/?term=817)(22.373)[AP3D1](http://www.ncbi.nlm.nih.gov/gene/?term=8943)(22.373)[MYO5A](http://www.ncbi.nlm.nih.gov/gene/?term=4644)(22.373)[ILVBL](http://www.ncbi.nlm.nih.gov/gene/?term=10994)(22.373)[CAV3](http://www.ncbi.nlm.nih.gov/gene/?term=859)(22.373) [HTT](http://www.ncbi.nlm.nih.gov/gene/?term=3064)(22.373)[IL10](http://www.ncbi.nlm.nih.gov/gene/?term=3586)(22.373)[EGLN3](http://www.ncbi.nlm.nih.gov/gene/?term=112399)(22.373)[OGDHL](http://www.ncbi.nlm.nih.gov/gene/?term=55753)(22.373)[KCNA5](http://www.ncbi.nlm.nih.gov/gene/?term=3741)(22.373) [TST](http://www.ncbi.nlm.nih.gov/gene/?term=7263)(22.373)[SLC1A6](http://www.ncbi.nlm.nih.gov/gene/?term=6511)(22.373)[NPPA](http://www.ncbi.nlm.nih.gov/gene/?term=4878)(22.373)[ACADM](http://www.ncbi.nlm.nih.gov/gene/?term=34)(22.373)[SQRDL](http://www.ncbi.nlm.nih.gov/gene/?term=58472)(22.373) [TMEM110](http://www.ncbi.nlm.nih.gov/gene/?term=375346)(22.373)[CDC42](http://www.ncbi.nlm.nih.gov/gene/?term=998)(22.373)[ARHGEF2](http://www.ncbi.nlm.nih.gov/gene/?term=9181)(22.373)[EGLN1](http://www.ncbi.nlm.nih.gov/gene/?term=54583)(22.373)[ETFDH](http://www.ncbi.nlm.nih.gov/gene/?term=2110)(22.373) [EPHA4](http://www.ncbi.nlm.nih.gov/gene/?term=2043)(22.373)[OCA2](http://www.ncbi.nlm.nih.gov/gene/?term=4948)(22.373)[HSD17B8](http://www.ncbi.nlm.nih.gov/gene/?term=7923)(22.373)[CTNNB1](http://www.ncbi.nlm.nih.gov/gene/?term=1499)(22.373)[P4HA3](http://www.ncbi.nlm.nih.gov/gene/?term=283208)(22.373) [EDA](http://www.ncbi.nlm.nih.gov/gene/?term=1896)(22.373)[HACL1](http://www.ncbi.nlm.nih.gov/gene/?term=26061)(22.373)[RYR2](http://www.ncbi.nlm.nih.gov/gene/?term=6262)(22.373)[HAP1](http://www.ncbi.nlm.nih.gov/gene/?term=9001)(22.373)[PCSK9](http://www.ncbi.nlm.nih.gov/gene/?term=255738)(22.373) [KCNQ1](http://www.ncbi.nlm.nih.gov/gene/?term=3784)(22.373)[CASQ2](http://www.ncbi.nlm.nih.gov/gene/?term=845)(22.373)[GJA5](http://www.ncbi.nlm.nih.gov/gene/?term=2702)(22.373)[DHRS9](http://www.ncbi.nlm.nih.gov/gene/?term=10170)(22.373)[GPR143](http://www.ncbi.nlm.nih.gov/gene/?term=4935)(22.373) [UGT1A1](http://www.ncbi.nlm.nih.gov/gene/?term=54658)(22.373)[DLD](http://www.ncbi.nlm.nih.gov/gene/?term=1738)(22.373)[GNAS](http://www.ncbi.nlm.nih.gov/gene/?term=2778)(22.373)[SLC25A13](http://www.ncbi.nlm.nih.gov/gene/?term=10165)(22.373)[SFXN5](http://www.ncbi.nlm.nih.gov/gene/?term=94097)(22.373) [PLOD2](http://www.ncbi.nlm.nih.gov/gene/?term=5352)(22.373)[CYP1A1](http://www.ncbi.nlm.nih.gov/gene/?term=1543)(22.373)[SULT2A1](http://www.ncbi.nlm.nih.gov/gene/?term=6822)(22.373)[NFX1](http://www.ncbi.nlm.nih.gov/gene/?term=4799)(22.373)[DBT](http://www.ncbi.nlm.nih.gov/gene/?term=1629)(22.373) [SLC25A1](http://www.ncbi.nlm.nih.gov/gene/?term=6576)(22.373) | |
| [Glycycoumarin](http://www.megabionet.org/tcmid/ingredient/8832/) | [CNR2](http://www.ncbi.nlm.nih.gov/gene/?term=1269)(23.000)[CNR1](http://www.ncbi.nlm.nih.gov/gene/?term=1268)(23.000)[DRD2](http://www.ncbi.nlm.nih.gov/gene/?term=1813)(22.373) | |
| [Methyl Myristate](http://www.megabionet.org/tcmid/ingredient/14602/) | [NPR1](http://www.ncbi.nlm.nih.gov/gene/?term=4881)(80.882)[SCN11A](http://www.ncbi.nlm.nih.gov/gene/?term=11280)(48.000)[SCN2B](http://www.ncbi.nlm.nih.gov/gene/?term=6327)(48.000)[ABAT](http://www.ncbi.nlm.nih.gov/gene/?term=18)(48.000)[SCN1A](http://www.ncbi.nlm.nih.gov/gene/?term=6323)(48.000) [ESRRG](http://www.ncbi.nlm.nih.gov/gene/?term=2104)(48.000)[SCN3B](http://www.ncbi.nlm.nih.gov/gene/?term=55800)(48.000)[COX6C](http://www.ncbi.nlm.nih.gov/gene/?term=1345)(48.000)[COX5B](http://www.ncbi.nlm.nih.gov/gene/?term=1329)(48.000)[COX7C](http://www.ncbi.nlm.nih.gov/gene/?term=1350)(48.000) [COX1](http://www.ncbi.nlm.nih.gov/gene/?term=4512)(48.000)[SCN3A](http://www.ncbi.nlm.nih.gov/gene/?term=6328)(48.000)[AKR1C2](http://www.ncbi.nlm.nih.gov/gene/?term=1646)(48.000)[SCN7A](http://www.ncbi.nlm.nih.gov/gene/?term=6332)(48.000)[SCN5A](http://www.ncbi.nlm.nih.gov/gene/?term=6331)(48.000) [SCN10A](http://www.ncbi.nlm.nih.gov/gene/?term=6336)(48.000)[AKR1D1](http://www.ncbi.nlm.nih.gov/gene/?term=6718)(48.000)[ALDH5A1](http://www.ncbi.nlm.nih.gov/gene/?term=7915)(48.000)[SCN2A](http://www.ncbi.nlm.nih.gov/gene/?term=6326)(48.000)[COX5A](http://www.ncbi.nlm.nih.gov/gene/?term=9377)(48.000) [COX3](http://www.ncbi.nlm.nih.gov/gene/?term=4514)(48.000)[AR](http://www.ncbi.nlm.nih.gov/gene/?term=367)(48.000)[COX7A1](http://www.ncbi.nlm.nih.gov/gene/?term=1346)(48.000)[FECH](http://www.ncbi.nlm.nih.gov/gene/?term=2235)(48.000)[COX4I1](http://www.ncbi.nlm.nih.gov/gene/?term=1327)(48.000) [HDAC9](http://www.ncbi.nlm.nih.gov/gene/?term=9734)(48.000)[PLA2G1B](http://www.ncbi.nlm.nih.gov/gene/?term=5319)(48.000)[COX6A2](http://www.ncbi.nlm.nih.gov/gene/?term=1339)(48.000)[TYR](http://www.ncbi.nlm.nih.gov/gene/?term=7299)(48.000)[SCN9A](http://www.ncbi.nlm.nih.gov/gene/?term=6335)(48.000) [SCN4A](http://www.ncbi.nlm.nih.gov/gene/?term=6329)(48.000)[SCN4B](http://www.ncbi.nlm.nih.gov/gene/?term=6330)(48.000)[ADH1C](http://www.ncbi.nlm.nih.gov/gene/?term=126)(48.000)[COX6B1](http://www.ncbi.nlm.nih.gov/gene/?term=1340)(48.000)[FABP6](http://www.ncbi.nlm.nih.gov/gene/?term=2172)(48.000) [CES1](http://www.ncbi.nlm.nih.gov/gene/?term=1066)(48.000)[SRD5A2](http://www.ncbi.nlm.nih.gov/gene/?term=6716)(48.000)[ACADSB](http://www.ncbi.nlm.nih.gov/gene/?term=36)(48.000)[SCN8A](http://www.ncbi.nlm.nih.gov/gene/?term=6334)(48.000)[COX7B](http://www.ncbi.nlm.nih.gov/gene/?term=1349)(48.000) [SCN1B](http://www.ncbi.nlm.nih.gov/gene/?term=6324)(48.000)[COX2](http://www.ncbi.nlm.nih.gov/gene/?term=4513)(48.000)[OGDH](http://www.ncbi.nlm.nih.gov/gene/?term=4967)(48.000)[HDAC2](http://www.ncbi.nlm.nih.gov/gene/?term=3066)(48.000)[COX8A](http://www.ncbi.nlm.nih.gov/gene/?term=1351)(48.000) [NR1H4](http://www.ncbi.nlm.nih.gov/gene/?term=9971)(48.000)[NPR3](http://www.ncbi.nlm.nih.gov/gene/?term=4883)(22.373)[NPR2](http://www.ncbi.nlm.nih.gov/gene/?term=4882)(22.373) | |
| [Liquoric Acid](http://www.megabionet.org/tcmid/ingredient/31455/) | [ESR1](http://www.ncbi.nlm.nih.gov/gene/?term=2099)(48.000)[PGR](http://www.ncbi.nlm.nih.gov/gene/?term=5241)(48.000)[AR](http://www.ncbi.nlm.nih.gov/gene/?term=367)(48.000)[NR3C1](http://www.ncbi.nlm.nih.gov/gene/?term=2908)(48.000)[HMGCR](http://www.ncbi.nlm.nih.gov/gene/?term=3156)(48.000) [ITGB2](http://www.ncbi.nlm.nih.gov/gene/?term=3689)(48.000)[ANXA1](http://www.ncbi.nlm.nih.gov/gene/?term=301)(48.000)[ITGAL](http://www.ncbi.nlm.nih.gov/gene/?term=3683)(48.000)[HDAC2](http://www.ncbi.nlm.nih.gov/gene/?term=3066)(48.000)[NR3C2](http://www.ncbi.nlm.nih.gov/gene/?term=4306)(48.000) | |
| [Calycosin](http://www.megabionet.org/tcmid/ingredient/3004/) | This compound doesn't have any potential target with score larger than 20. | |
| [Gmelofuran](http://www.megabionet.org/tcmid/ingredient/8862/) | [CHRNA10](http://www.ncbi.nlm.nih.gov/gene/?term=57053)(80.882) | |
| [Oleanolic Acid](http://www.megabionet.org/tcmid/ingredient/23101/) | [AR](http://www.ncbi.nlm.nih.gov/gene/?term=367)(122.778)[SRD5A1](http://www.ncbi.nlm.nih.gov/gene/?term=6715)(48.000)[CYP17A1](http://www.ncbi.nlm.nih.gov/gene/?term=1586)(48.000)[SLC8A1](http://www.ncbi.nlm.nih.gov/gene/?term=6546)(48.000)[F12](http://www.ncbi.nlm.nih.gov/gene/?term=2161)(48.000) [PRLR](http://www.ncbi.nlm.nih.gov/gene/?term=5618)(48.000)[ESR1](http://www.ncbi.nlm.nih.gov/gene/?term=2099)(48.000)[TRPV1](http://www.ncbi.nlm.nih.gov/gene/?term=7442)(48.000)[PPARG](http://www.ncbi.nlm.nih.gov/gene/?term=5468)(48.000)[PTGER1](http://www.ncbi.nlm.nih.gov/gene/?term=5731)(48.000) [FFAR1](http://www.ncbi.nlm.nih.gov/gene/?term=2864)(48.000)[PTGER4](http://www.ncbi.nlm.nih.gov/gene/?term=5734)(48.000)[PGR](http://www.ncbi.nlm.nih.gov/gene/?term=5241)(48.000)[NR3C1](http://www.ncbi.nlm.nih.gov/gene/?term=2908)(48.000)[OPRK1](http://www.ncbi.nlm.nih.gov/gene/?term=4986)(48.000) [FADS2](http://www.ncbi.nlm.nih.gov/gene/?term=9415)(48.000)[FADS1](http://www.ncbi.nlm.nih.gov/gene/?term=3992)(48.000)[PPARD](http://www.ncbi.nlm.nih.gov/gene/?term=5467)(48.000)[PTGER2](http://www.ncbi.nlm.nih.gov/gene/?term=5732)(48.000)[PTGS1](http://www.ncbi.nlm.nih.gov/gene/?term=5742)(48.000) [ACSL4](http://www.ncbi.nlm.nih.gov/gene/?term=2182)(48.000)[PTGER3](http://www.ncbi.nlm.nih.gov/gene/?term=5733)(48.000)[PTGS2](http://www.ncbi.nlm.nih.gov/gene/?term=5743)(48.000)[ELOVL4](http://www.ncbi.nlm.nih.gov/gene/?term=6785)(48.000)[ANXA1](http://www.ncbi.nlm.nih.gov/gene/?term=301)(48.000) [ACSL3](http://www.ncbi.nlm.nih.gov/gene/?term=2181)(48.000)[NR3C2](http://www.ncbi.nlm.nih.gov/gene/?term=4306)(48.000)[ESRRG](http://www.ncbi.nlm.nih.gov/gene/?term=2104)(23.000)[COX6C](http://www.ncbi.nlm.nih.gov/gene/?term=1345)(23.000)[COX5B](http://www.ncbi.nlm.nih.gov/gene/?term=1329)(23.000) [COX7C](http://www.ncbi.nlm.nih.gov/gene/?term=1350)(23.000)[COX1](http://www.ncbi.nlm.nih.gov/gene/?term=4512)(23.000)[AKR1C2](http://www.ncbi.nlm.nih.gov/gene/?term=1646)(23.000)[ADH1B](http://www.ncbi.nlm.nih.gov/gene/?term=125)(23.000)[COX5A](http://www.ncbi.nlm.nih.gov/gene/?term=9377)(23.000) [COX3](http://www.ncbi.nlm.nih.gov/gene/?term=4514)(23.000)[COX7A1](http://www.ncbi.nlm.nih.gov/gene/?term=1346)(23.000)[FECH](http://www.ncbi.nlm.nih.gov/gene/?term=2235)(23.000)[COX4I1](http://www.ncbi.nlm.nih.gov/gene/?term=1327)(23.000)[PLA2G1B](http://www.ncbi.nlm.nih.gov/gene/?term=5319)(23.000) [AKR1C1](http://www.ncbi.nlm.nih.gov/gene/?term=1645)(23.000)[COX6A2](http://www.ncbi.nlm.nih.gov/gene/?term=1339)(23.000)[HMGCR](http://www.ncbi.nlm.nih.gov/gene/?term=3156)(23.000)[ADH1C](http://www.ncbi.nlm.nih.gov/gene/?term=126)(23.000)[COX6B1](http://www.ncbi.nlm.nih.gov/gene/?term=1340)(23.000) [FABP6](http://www.ncbi.nlm.nih.gov/gene/?term=2172)(23.000)[CES1](http://www.ncbi.nlm.nih.gov/gene/?term=1066)(23.000)[COX7B](http://www.ncbi.nlm.nih.gov/gene/?term=1349)(23.000)[COX2](http://www.ncbi.nlm.nih.gov/gene/?term=4513)(23.000)[COX8A](http://www.ncbi.nlm.nih.gov/gene/?term=1351)(23.000) [NR1H4](http://www.ncbi.nlm.nih.gov/gene/?term=9971)(23.000) | |
| [Methyl-24-Hydroxy-11-Deoxoglycyrrhetate](http://www.megabionet.org/tcmid/ingredient/14493/) | [SRD5A1](http://www.ncbi.nlm.nih.gov/gene/?term=6715)(48.000)[SLC8A1](http://www.ncbi.nlm.nih.gov/gene/?term=6546)(48.000)[F12](http://www.ncbi.nlm.nih.gov/gene/?term=2161)(48.000)[ESR1](http://www.ncbi.nlm.nih.gov/gene/?term=2099)(48.000)[TRPV1](http://www.ncbi.nlm.nih.gov/gene/?term=7442)(48.000) [PTGER4](http://www.ncbi.nlm.nih.gov/gene/?term=5734)(48.000)[PGR](http://www.ncbi.nlm.nih.gov/gene/?term=5241)(48.000)[AR](http://www.ncbi.nlm.nih.gov/gene/?term=367)(48.000)[NR3C1](http://www.ncbi.nlm.nih.gov/gene/?term=2908)(48.000)[FADS2](http://www.ncbi.nlm.nih.gov/gene/?term=9415)(48.000) [FADS1](http://www.ncbi.nlm.nih.gov/gene/?term=3992)(48.000)[PTGER2](http://www.ncbi.nlm.nih.gov/gene/?term=5732)(48.000)[PTGS1](http://www.ncbi.nlm.nih.gov/gene/?term=5742)(48.000)[PTGER3](http://www.ncbi.nlm.nih.gov/gene/?term=5733)(48.000)[PTGS2](http://www.ncbi.nlm.nih.gov/gene/?term=5743)(48.000) [ELOVL4](http://www.ncbi.nlm.nih.gov/gene/?term=6785)(48.000)[ANXA1](http://www.ncbi.nlm.nih.gov/gene/?term=301)(48.000)[NR3C2](http://www.ncbi.nlm.nih.gov/gene/?term=4306)(48.000) | |
| [Coumarin](http://www.megabionet.org/tcmid/ingredient/4140/) | This compound doesn't have any potential target with score larger than 20. | |
| [Hispidulin](http://www.megabionet.org/tcmid/ingredient/9563/) | This compound doesn't have any potential target with score larger than 20. | |
| [Methyl Stearate](http://www.megabionet.org/tcmid/ingredient/24923/) | [NPR1](http://www.ncbi.nlm.nih.gov/gene/?term=4881)(80.882)[SCN11A](http://www.ncbi.nlm.nih.gov/gene/?term=11280)(48.000)[SCN2B](http://www.ncbi.nlm.nih.gov/gene/?term=6327)(48.000)[ABAT](http://www.ncbi.nlm.nih.gov/gene/?term=18)(48.000)[SCN1A](http://www.ncbi.nlm.nih.gov/gene/?term=6323)(48.000) [ESRRG](http://www.ncbi.nlm.nih.gov/gene/?term=2104)(48.000)[SCN3B](http://www.ncbi.nlm.nih.gov/gene/?term=55800)(48.000)[COX6C](http://www.ncbi.nlm.nih.gov/gene/?term=1345)(48.000)[COX5B](http://www.ncbi.nlm.nih.gov/gene/?term=1329)(48.000)[COX7C](http://www.ncbi.nlm.nih.gov/gene/?term=1350)(48.000) [COX1](http://www.ncbi.nlm.nih.gov/gene/?term=4512)(48.000)[SCN3A](http://www.ncbi.nlm.nih.gov/gene/?term=6328)(48.000)[AKR1C2](http://www.ncbi.nlm.nih.gov/gene/?term=1646)(48.000)[SCN7A](http://www.ncbi.nlm.nih.gov/gene/?term=6332)(48.000)[SCN5A](http://www.ncbi.nlm.nih.gov/gene/?term=6331)(48.000) [SCN10A](http://www.ncbi.nlm.nih.gov/gene/?term=6336)(48.000)[AKR1D1](http://www.ncbi.nlm.nih.gov/gene/?term=6718)(48.000)[ALDH5A1](http://www.ncbi.nlm.nih.gov/gene/?term=7915)(48.000)[SCN2A](http://www.ncbi.nlm.nih.gov/gene/?term=6326)(48.000)[COX5A](http://www.ncbi.nlm.nih.gov/gene/?term=9377)(48.000) [COX3](http://www.ncbi.nlm.nih.gov/gene/?term=4514)(48.000)[AR](http://www.ncbi.nlm.nih.gov/gene/?term=367)(48.000)[COX7A1](http://www.ncbi.nlm.nih.gov/gene/?term=1346)(48.000)[FECH](http://www.ncbi.nlm.nih.gov/gene/?term=2235)(48.000)[COX4I1](http://www.ncbi.nlm.nih.gov/gene/?term=1327)(48.000) [HDAC9](http://www.ncbi.nlm.nih.gov/gene/?term=9734)(48.000)[PLA2G1B](http://www.ncbi.nlm.nih.gov/gene/?term=5319)(48.000)[COX6A2](http://www.ncbi.nlm.nih.gov/gene/?term=1339)(48.000)[TYR](http://www.ncbi.nlm.nih.gov/gene/?term=7299)(48.000)[SCN9A](http://www.ncbi.nlm.nih.gov/gene/?term=6335)(48.000) [SCN4A](http://www.ncbi.nlm.nih.gov/gene/?term=6329)(48.000)[SCN4B](http://www.ncbi.nlm.nih.gov/gene/?term=6330)(48.000)[ADH1C](http://www.ncbi.nlm.nih.gov/gene/?term=126)(48.000)[COX6B1](http://www.ncbi.nlm.nih.gov/gene/?term=1340)(48.000)[FABP6](http://www.ncbi.nlm.nih.gov/gene/?term=2172)(48.000) [CES1](http://www.ncbi.nlm.nih.gov/gene/?term=1066)(48.000)[SRD5A2](http://www.ncbi.nlm.nih.gov/gene/?term=6716)(48.000)[ACADSB](http://www.ncbi.nlm.nih.gov/gene/?term=36)(48.000)[SCN8A](http://www.ncbi.nlm.nih.gov/gene/?term=6334)(48.000)[COX7B](http://www.ncbi.nlm.nih.gov/gene/?term=1349)(48.000) [SCN1B](http://www.ncbi.nlm.nih.gov/gene/?term=6324)(48.000)[COX2](http://www.ncbi.nlm.nih.gov/gene/?term=4513)(48.000)[OGDH](http://www.ncbi.nlm.nih.gov/gene/?term=4967)(48.000)[HDAC2](http://www.ncbi.nlm.nih.gov/gene/?term=3066)(48.000)[COX8A](http://www.ncbi.nlm.nih.gov/gene/?term=1351)(48.000) [NR1H4](http://www.ncbi.nlm.nih.gov/gene/?term=9971)(48.000)[NPR3](http://www.ncbi.nlm.nih.gov/gene/?term=4883)(22.373)[NPR2](http://www.ncbi.nlm.nih.gov/gene/?term=4882)(22.373) | |
| [Ethyl Caprylate](http://www.megabionet.org/tcmid/ingredient/24306/) | [AR](http://www.ncbi.nlm.nih.gov/gene/?term=367)(122.778)[NPR1](http://www.ncbi.nlm.nih.gov/gene/?term=4881)(80.882)[CHRM1](http://www.ncbi.nlm.nih.gov/gene/?term=1128)(48.000)[AKR1D1](http://www.ncbi.nlm.nih.gov/gene/?term=6718)(48.000)[TYR](http://www.ncbi.nlm.nih.gov/gene/?term=7299)(48.000) [SRD5A2](http://www.ncbi.nlm.nih.gov/gene/?term=6716)(48.000)[CHRM2](http://www.ncbi.nlm.nih.gov/gene/?term=1129)(48.000)[NPR3](http://www.ncbi.nlm.nih.gov/gene/?term=4883)(22.373)[NPR2](http://www.ncbi.nlm.nih.gov/gene/?term=4882)(22.373) | |
| [Isotrifoliol](http://www.megabionet.org/tcmid/ingredient/11743/) | [SEC14L3](http://www.ncbi.nlm.nih.gov/gene/?term=266629)(23.000)[PPP2CA](http://www.ncbi.nlm.nih.gov/gene/?term=5515)(23.000)[PRKCA](http://www.ncbi.nlm.nih.gov/gene/?term=5578)(23.000)[NR1I2](http://www.ncbi.nlm.nih.gov/gene/?term=8856)(23.000)[ALOX5](http://www.ncbi.nlm.nih.gov/gene/?term=240)(23.000) [PPP2CB](http://www.ncbi.nlm.nih.gov/gene/?term=5516)(23.000)[SEC14L2](http://www.ncbi.nlm.nih.gov/gene/?term=23541)(23.000)[DGKA](http://www.ncbi.nlm.nih.gov/gene/?term=1606)(23.000)[PRKCB](http://www.ncbi.nlm.nih.gov/gene/?term=5579)(23.000)[SEC14L4](http://www.ncbi.nlm.nih.gov/gene/?term=284904)(23.000) | |
| [Glycyrrhisoflavanone](http://www.megabionet.org/tcmid/ingredient/8842/) | [SOAT1](http://www.ncbi.nlm.nih.gov/gene/?term=6646)(23.000)[MTTP](http://www.ncbi.nlm.nih.gov/gene/?term=4547)(23.000)[CNR2](http://www.ncbi.nlm.nih.gov/gene/?term=1269)(23.000)[CNR1](http://www.ncbi.nlm.nih.gov/gene/?term=1268)(23.000)[SOAT2](http://www.ncbi.nlm.nih.gov/gene/?term=8435)(23.000) [DRD2](http://www.ncbi.nlm.nih.gov/gene/?term=1813)(22.373)[PAWR](http://www.ncbi.nlm.nih.gov/gene/?term=5074)(22.373) | |
| [Nicotine](http://www.megabionet.org/tcmid/ingredient/23307/) | [CHRNA4](http://www.ncbi.nlm.nih.gov/gene/?term=1137)(686.000)[CHRNB3](http://www.ncbi.nlm.nih.gov/gene/?term=1142)(686.000)[CHRNA7](http://www.ncbi.nlm.nih.gov/gene/?term=1139)(686.000)[CHRNA10](http://www.ncbi.nlm.nih.gov/gene/?term=57053)(686.000)[CHRNA3](http://www.ncbi.nlm.nih.gov/gene/?term=1136)(686.000) [CHRNB2](http://www.ncbi.nlm.nih.gov/gene/?term=1141)(686.000)[CHRNA2](http://www.ncbi.nlm.nih.gov/gene/?term=1135)(686.000)[CHRNA6](http://www.ncbi.nlm.nih.gov/gene/?term=8973)(686.000)[CHAT](http://www.ncbi.nlm.nih.gov/gene/?term=1103)(686.000)[CHRNA9](http://www.ncbi.nlm.nih.gov/gene/?term=55584)(686.000) [CHRFAM7A](http://www.ncbi.nlm.nih.gov/gene/?term=89832)(686.000)[CYP19A1](http://www.ncbi.nlm.nih.gov/gene/?term=1588)(686.000)[CHRNA5](http://www.ncbi.nlm.nih.gov/gene/?term=1138)(686.000)[CHRNB4](http://www.ncbi.nlm.nih.gov/gene/?term=1143)(686.000)[HTR2A](http://www.ncbi.nlm.nih.gov/gene/?term=3356)(80.882) [CHRM3](http://www.ncbi.nlm.nih.gov/gene/?term=1131)(80.882)[SULT1E1](http://www.ncbi.nlm.nih.gov/gene/?term=6783)(80.882)[CHRM1](http://www.ncbi.nlm.nih.gov/gene/?term=1128)(80.882)[HTR2C](http://www.ncbi.nlm.nih.gov/gene/?term=3358)(80.882)[HRH1](http://www.ncbi.nlm.nih.gov/gene/?term=3269)(80.882) [CHRM2](http://www.ncbi.nlm.nih.gov/gene/?term=1129)(80.882)[ADRA1B](http://www.ncbi.nlm.nih.gov/gene/?term=147)(55.444)[SLC6A3](http://www.ncbi.nlm.nih.gov/gene/?term=6531)(23.000)[CACNA1H](http://www.ncbi.nlm.nih.gov/gene/?term=8912)(23.000)[HTR5A](http://www.ncbi.nlm.nih.gov/gene/?term=3361)(23.000) [ADRA1A](http://www.ncbi.nlm.nih.gov/gene/?term=148)(23.000)[GABRA2](http://www.ncbi.nlm.nih.gov/gene/?term=2555)(23.000)[CACNA1F](http://www.ncbi.nlm.nih.gov/gene/?term=778)(23.000)[GABRD](http://www.ncbi.nlm.nih.gov/gene/?term=2563)(23.000)[ADRB2](http://www.ncbi.nlm.nih.gov/gene/?term=154)(23.000) [GABRB1](http://www.ncbi.nlm.nih.gov/gene/?term=2560)(23.000)[GABRG3](http://www.ncbi.nlm.nih.gov/gene/?term=2567)(23.000)[HTR2B](http://www.ncbi.nlm.nih.gov/gene/?term=3357)(23.000)[ADRA2A](http://www.ncbi.nlm.nih.gov/gene/?term=150)(23.000)[CACNA1I](http://www.ncbi.nlm.nih.gov/gene/?term=8911)(23.000) [HTR1B](http://www.ncbi.nlm.nih.gov/gene/?term=3351)(23.000)[HTR6](http://www.ncbi.nlm.nih.gov/gene/?term=3362)(23.000)[SCN10A](http://www.ncbi.nlm.nih.gov/gene/?term=6336)(23.000)[GABRE](http://www.ncbi.nlm.nih.gov/gene/?term=2564)(23.000)[PTGER1](http://www.ncbi.nlm.nih.gov/gene/?term=5731)(23.000) [GABRA3](http://www.ncbi.nlm.nih.gov/gene/?term=2556)(23.000)[CACNA1S](http://www.ncbi.nlm.nih.gov/gene/?term=779)(23.000)[ADRB1](http://www.ncbi.nlm.nih.gov/gene/?term=153)(23.000)[GABRG1](http://www.ncbi.nlm.nih.gov/gene/?term=2565)(23.000)[HRH3](http://www.ncbi.nlm.nih.gov/gene/?term=11255)(23.000) [GABRP](http://www.ncbi.nlm.nih.gov/gene/?term=2568)(23.000)[ADRA1D](http://www.ncbi.nlm.nih.gov/gene/?term=146)(23.000)[GABRA4](http://www.ncbi.nlm.nih.gov/gene/?term=2557)(23.000)[HTR1D](http://www.ncbi.nlm.nih.gov/gene/?term=3352)(23.000)[DRD2](http://www.ncbi.nlm.nih.gov/gene/?term=1813)(23.000) [OPRK1](http://www.ncbi.nlm.nih.gov/gene/?term=4986)(23.000)[GABRB2](http://www.ncbi.nlm.nih.gov/gene/?term=2561)(23.000)[CACNA1C](http://www.ncbi.nlm.nih.gov/gene/?term=775)(23.000)[CACNA1D](http://www.ncbi.nlm.nih.gov/gene/?term=776)(23.000)[CHRM5](http://www.ncbi.nlm.nih.gov/gene/?term=1133)(23.000) [SLC6A4](http://www.ncbi.nlm.nih.gov/gene/?term=6532)(23.000)[HRH4](http://www.ncbi.nlm.nih.gov/gene/?term=59340)(23.000)[ACHE](http://www.ncbi.nlm.nih.gov/gene/?term=43)(23.000)[KCNH2](http://www.ncbi.nlm.nih.gov/gene/?term=3757)(23.000)[GABRQ](http://www.ncbi.nlm.nih.gov/gene/?term=55879)(23.000) [DRD4](http://www.ncbi.nlm.nih.gov/gene/?term=1815)(23.000)[HTR7](http://www.ncbi.nlm.nih.gov/gene/?term=3363)(23.000)[HRH2](http://www.ncbi.nlm.nih.gov/gene/?term=3274)(23.000)[HTR1F](http://www.ncbi.nlm.nih.gov/gene/?term=3355)(23.000)[HTR1A](http://www.ncbi.nlm.nih.gov/gene/?term=3350)(23.000) [ADRA2C](http://www.ncbi.nlm.nih.gov/gene/?term=152)(23.000)[DRD3](http://www.ncbi.nlm.nih.gov/gene/?term=1814)(23.000)[GABRB3](http://www.ncbi.nlm.nih.gov/gene/?term=2562)(23.000)[KCND2](http://www.ncbi.nlm.nih.gov/gene/?term=3751)(23.000)[GABRA5](http://www.ncbi.nlm.nih.gov/gene/?term=2558)(23.000) [DRD5](http://www.ncbi.nlm.nih.gov/gene/?term=1816)(23.000)[CACNA1G](http://www.ncbi.nlm.nih.gov/gene/?term=8913)(23.000)[HTR1E](http://www.ncbi.nlm.nih.gov/gene/?term=3354)(23.000)[GABRA6](http://www.ncbi.nlm.nih.gov/gene/?term=2559)(23.000)[HTR3A](http://www.ncbi.nlm.nih.gov/gene/?term=3359)(23.000) [GABRA1](http://www.ncbi.nlm.nih.gov/gene/?term=2554)(23.000)[DRD1](http://www.ncbi.nlm.nih.gov/gene/?term=1812)(23.000)[GABRG2](http://www.ncbi.nlm.nih.gov/gene/?term=2566)(23.000)[ADRB3](http://www.ncbi.nlm.nih.gov/gene/?term=155)(23.000)[CHRM4](http://www.ncbi.nlm.nih.gov/gene/?term=1132)(23.000) [KCND3](http://www.ncbi.nlm.nih.gov/gene/?term=3752)(23.000)[ADRA2B](http://www.ncbi.nlm.nih.gov/gene/?term=151)(23.000)[SLC6A2](http://www.ncbi.nlm.nih.gov/gene/?term=6530)(23.000)[TNFSF11](http://www.ncbi.nlm.nih.gov/gene/?term=8600)(22.373)[SLITRK6](http://www.ncbi.nlm.nih.gov/gene/?term=84189)(22.373) [UTS2](http://www.ncbi.nlm.nih.gov/gene/?term=10911)(22.373)[NAV2](http://www.ncbi.nlm.nih.gov/gene/?term=89797)(22.373)[P2RX2](http://www.ncbi.nlm.nih.gov/gene/?term=22953)(22.373)[LPCAT4](http://www.ncbi.nlm.nih.gov/gene/?term=254531)(22.373)[ARFGEF2](http://www.ncbi.nlm.nih.gov/gene/?term=10564)(22.373) [ADORA2A](http://www.ncbi.nlm.nih.gov/gene/?term=135)(22.373)[CACNA1A](http://www.ncbi.nlm.nih.gov/gene/?term=773)(22.373)[LHCGR](http://www.ncbi.nlm.nih.gov/gene/?term=3973)(22.373)[SUMO1](http://www.ncbi.nlm.nih.gov/gene/?term=7341)(22.373)[DOCK5](http://www.ncbi.nlm.nih.gov/gene/?term=80005)(22.373) [NTSR1](http://www.ncbi.nlm.nih.gov/gene/?term=4923)(22.373)[CNR2](http://www.ncbi.nlm.nih.gov/gene/?term=1269)(22.373)[SULT1A1](http://www.ncbi.nlm.nih.gov/gene/?term=6817)(22.373)[MTNR1B](http://www.ncbi.nlm.nih.gov/gene/?term=4544)(22.373)[FOXP3](http://www.ncbi.nlm.nih.gov/gene/?term=50943)(22.373) [PTK2B](http://www.ncbi.nlm.nih.gov/gene/?term=2185)(22.373)[P2RX3](http://www.ncbi.nlm.nih.gov/gene/?term=5024)(22.373)[SLC44A4](http://www.ncbi.nlm.nih.gov/gene/?term=80736)(22.373)[FLOT1](http://www.ncbi.nlm.nih.gov/gene/?term=10211)(22.373)[NPY5R](http://www.ncbi.nlm.nih.gov/gene/?term=4889)(22.373) [SULT2B1](http://www.ncbi.nlm.nih.gov/gene/?term=6820)(22.373)[P2RY1](http://www.ncbi.nlm.nih.gov/gene/?term=5028)(22.373)[APLN](http://www.ncbi.nlm.nih.gov/gene/?term=8862)(22.373)[GHRL](http://www.ncbi.nlm.nih.gov/gene/?term=51738)(22.373)[GNPAT](http://www.ncbi.nlm.nih.gov/gene/?term=8443)(22.373) [TBR1](http://www.ncbi.nlm.nih.gov/gene/?term=10716)(22.373)[C5](http://www.ncbi.nlm.nih.gov/gene/?term=727)(22.373)[SULT1A2](http://www.ncbi.nlm.nih.gov/gene/?term=6799)(22.373)[LPCAT2](http://www.ncbi.nlm.nih.gov/gene/?term=54947)(22.373)[PAX2](http://www.ncbi.nlm.nih.gov/gene/?term=5076)(22.373) [STAP1](http://www.ncbi.nlm.nih.gov/gene/?term=26228)(22.373)[UTS2R](http://www.ncbi.nlm.nih.gov/gene/?term=2837)(22.373)[GHRH](http://www.ncbi.nlm.nih.gov/gene/?term=2691)(22.373)[HCRT](http://www.ncbi.nlm.nih.gov/gene/?term=3060)(22.373)[RAB3B](http://www.ncbi.nlm.nih.gov/gene/?term=5865)(22.373) [CNR1](http://www.ncbi.nlm.nih.gov/gene/?term=1268)(22.373)[MMP28](http://www.ncbi.nlm.nih.gov/gene/?term=79148)(22.373)[SLC18A3](http://www.ncbi.nlm.nih.gov/gene/?term=6572)(22.373)[CRH](http://www.ncbi.nlm.nih.gov/gene/?term=1392)(22.373)[GLI3](http://www.ncbi.nlm.nih.gov/gene/?term=2737)(22.373) [CAV3](http://www.ncbi.nlm.nih.gov/gene/?term=859)(22.373)[IL10](http://www.ncbi.nlm.nih.gov/gene/?term=3586)(22.373)[IL6](http://www.ncbi.nlm.nih.gov/gene/?term=3569)(22.373)[RNF207](http://www.ncbi.nlm.nih.gov/gene/?term=388591)(22.373)[TACR2](http://www.ncbi.nlm.nih.gov/gene/?term=6865)(22.373) [PDGFA](http://www.ncbi.nlm.nih.gov/gene/?term=5154)(22.373)[NRP1](http://www.ncbi.nlm.nih.gov/gene/?term=8829)(22.373)[TNFAIP3](http://www.ncbi.nlm.nih.gov/gene/?term=7128)(22.373)[ZNF219](http://www.ncbi.nlm.nih.gov/gene/?term=51222)(22.373)[TNR](http://www.ncbi.nlm.nih.gov/gene/?term=7143)(22.373) [GNA15](http://www.ncbi.nlm.nih.gov/gene/?term=2769)(22.373)[IL4](http://www.ncbi.nlm.nih.gov/gene/?term=3565)(22.373)[ZP3](http://www.ncbi.nlm.nih.gov/gene/?term=7784)(22.373)[LPCAT1](http://www.ncbi.nlm.nih.gov/gene/?term=79888)(22.373)[EPHB1](http://www.ncbi.nlm.nih.gov/gene/?term=2047)(22.373) [CX3CR1](http://www.ncbi.nlm.nih.gov/gene/?term=1524)(22.373)[POU1F1](http://www.ncbi.nlm.nih.gov/gene/?term=5449)(22.373)[KCNMA1](http://www.ncbi.nlm.nih.gov/gene/?term=3778)(22.373)[GPER1](http://www.ncbi.nlm.nih.gov/gene/?term=2852)(22.373)[POU4F3](http://www.ncbi.nlm.nih.gov/gene/?term=5459)(22.373) [CALCA](http://www.ncbi.nlm.nih.gov/gene/?term=796)(22.373)[PINK1](http://www.ncbi.nlm.nih.gov/gene/?term=65018)(22.373)[P2RX1](http://www.ncbi.nlm.nih.gov/gene/?term=5023)(22.373)[DOCK4](http://www.ncbi.nlm.nih.gov/gene/?term=9732)(22.373)[PDGFB](http://www.ncbi.nlm.nih.gov/gene/?term=5155)(22.373) [MAP2K1](http://www.ncbi.nlm.nih.gov/gene/?term=5604)(22.373)[CARTPT](http://www.ncbi.nlm.nih.gov/gene/?term=9607)(22.373)[CRAT](http://www.ncbi.nlm.nih.gov/gene/?term=1384)(22.373)[ATP8B1](http://www.ncbi.nlm.nih.gov/gene/?term=5205)(22.373)[KISS1](http://www.ncbi.nlm.nih.gov/gene/?term=3814)(22.373) [GRM7](http://www.ncbi.nlm.nih.gov/gene/?term=2917)(22.373)[FOS](http://www.ncbi.nlm.nih.gov/gene/?term=2353)(22.373)[GRIN1](http://www.ncbi.nlm.nih.gov/gene/?term=2902)(22.373) | |
| [Methionine Sulfoxide](http://www.megabionet.org/tcmid/ingredient/13817/) | This compound doesn't have any potential target with score larger than 20. | |
| [Ursolicacid](http://www.megabionet.org/tcmid/ingredient/22254/) | [SRD5A1](http://www.ncbi.nlm.nih.gov/gene/?term=6715)(48.000)[CYP17A1](http://www.ncbi.nlm.nih.gov/gene/?term=1586)(48.000)[SLC8A1](http://www.ncbi.nlm.nih.gov/gene/?term=6546)(48.000)[F12](http://www.ncbi.nlm.nih.gov/gene/?term=2161)(48.000)[PRLR](http://www.ncbi.nlm.nih.gov/gene/?term=5618)(48.000) [ESR1](http://www.ncbi.nlm.nih.gov/gene/?term=2099)(48.000)[TRPV1](http://www.ncbi.nlm.nih.gov/gene/?term=7442)(48.000)[PTGER1](http://www.ncbi.nlm.nih.gov/gene/?term=5731)(48.000)[PTGER4](http://www.ncbi.nlm.nih.gov/gene/?term=5734)(48.000)[PGR](http://www.ncbi.nlm.nih.gov/gene/?term=5241)(48.000) [AR](http://www.ncbi.nlm.nih.gov/gene/?term=367)(48.000)[NR3C1](http://www.ncbi.nlm.nih.gov/gene/?term=2908)(48.000)[OPRK1](http://www.ncbi.nlm.nih.gov/gene/?term=4986)(48.000)[FADS2](http://www.ncbi.nlm.nih.gov/gene/?term=9415)(48.000)[FADS1](http://www.ncbi.nlm.nih.gov/gene/?term=3992)(48.000) [PTGER2](http://www.ncbi.nlm.nih.gov/gene/?term=5732)(48.000)[PTGS1](http://www.ncbi.nlm.nih.gov/gene/?term=5742)(48.000)[PTGER3](http://www.ncbi.nlm.nih.gov/gene/?term=5733)(48.000)[PTGS2](http://www.ncbi.nlm.nih.gov/gene/?term=5743)(48.000)[ELOVL4](http://www.ncbi.nlm.nih.gov/gene/?term=6785)(48.000) [ANXA1](http://www.ncbi.nlm.nih.gov/gene/?term=301)(48.000)[NR3C2](http://www.ncbi.nlm.nih.gov/gene/?term=4306)(48.000)[ESRRG](http://www.ncbi.nlm.nih.gov/gene/?term=2104)(23.000)[COX6C](http://www.ncbi.nlm.nih.gov/gene/?term=1345)(23.000)[COX5B](http://www.ncbi.nlm.nih.gov/gene/?term=1329)(23.000) [COX7C](http://www.ncbi.nlm.nih.gov/gene/?term=1350)(23.000)[COX1](http://www.ncbi.nlm.nih.gov/gene/?term=4512)(23.000)[AKR1C2](http://www.ncbi.nlm.nih.gov/gene/?term=1646)(23.000)[ADH1B](http://www.ncbi.nlm.nih.gov/gene/?term=125)(23.000)[COX5A](http://www.ncbi.nlm.nih.gov/gene/?term=9377)(23.000) [COX3](http://www.ncbi.nlm.nih.gov/gene/?term=4514)(23.000)[COX7A1](http://www.ncbi.nlm.nih.gov/gene/?term=1346)(23.000)[FECH](http://www.ncbi.nlm.nih.gov/gene/?term=2235)(23.000)[COX4I1](http://www.ncbi.nlm.nih.gov/gene/?term=1327)(23.000)[PLA2G1B](http://www.ncbi.nlm.nih.gov/gene/?term=5319)(23.000) [AKR1C1](http://www.ncbi.nlm.nih.gov/gene/?term=1645)(23.000)[COX6A2](http://www.ncbi.nlm.nih.gov/gene/?term=1339)(23.000)[HMGCR](http://www.ncbi.nlm.nih.gov/gene/?term=3156)(23.000)[ADH1C](http://www.ncbi.nlm.nih.gov/gene/?term=126)(23.000)[COX6B1](http://www.ncbi.nlm.nih.gov/gene/?term=1340)(23.000) [FABP6](http://www.ncbi.nlm.nih.gov/gene/?term=2172)(23.000)[CES1](http://www.ncbi.nlm.nih.gov/gene/?term=1066)(23.000)[COX7B](http://www.ncbi.nlm.nih.gov/gene/?term=1349)(23.000)[COX2](http://www.ncbi.nlm.nih.gov/gene/?term=4513)(23.000)[COX8A](http://www.ncbi.nlm.nih.gov/gene/?term=1351)(23.000) [NR1H4](http://www.ncbi.nlm.nih.gov/gene/?term=9971)(23.000) | |
| [Furanodiene](http://www.megabionet.org/tcmid/ingredient/8013/) | This compound doesn't have any potential target with score larger than 20. | |
| [Canavanine](http://www.megabionet.org/tcmid/ingredient/3063/) | [AZIN2](http://www.ncbi.nlm.nih.gov/gene/?term=113451)(80.882)[ASL](http://www.ncbi.nlm.nih.gov/gene/?term=435)(80.882)[NOS2](http://www.ncbi.nlm.nih.gov/gene/?term=4843)(80.882)[SLC7A3](http://www.ncbi.nlm.nih.gov/gene/?term=84889)(80.882)[ASS1](http://www.ncbi.nlm.nih.gov/gene/?term=445)(80.882) [SLC7A1](http://www.ncbi.nlm.nih.gov/gene/?term=6541)(80.882)[SLC7A4](http://www.ncbi.nlm.nih.gov/gene/?term=6545)(80.882)[NOS3](http://www.ncbi.nlm.nih.gov/gene/?term=4846)(80.882)[ARG2](http://www.ncbi.nlm.nih.gov/gene/?term=384)(80.882)[HMOX1](http://www.ncbi.nlm.nih.gov/gene/?term=3162)(55.444) [OAZ3](http://www.ncbi.nlm.nih.gov/gene/?term=51686)(55.444)[AGMAT](http://www.ncbi.nlm.nih.gov/gene/?term=79814)(55.444)[KLF4](http://www.ncbi.nlm.nih.gov/gene/?term=9314)(55.444)[GNRH1](http://www.ncbi.nlm.nih.gov/gene/?term=2796)(55.444)[ARG1](http://www.ncbi.nlm.nih.gov/gene/?term=383)(55.444) [AZIN1](http://www.ncbi.nlm.nih.gov/gene/?term=51582)(55.444)[OTC](http://www.ncbi.nlm.nih.gov/gene/?term=5009)(55.444)[SLC1A5](http://www.ncbi.nlm.nih.gov/gene/?term=6510)(23.000)[GOT2](http://www.ncbi.nlm.nih.gov/gene/?term=2806)(23.000)[GPT2](http://www.ncbi.nlm.nih.gov/gene/?term=84706)(23.000) [BAAT](http://www.ncbi.nlm.nih.gov/gene/?term=570)(23.000)[SHMT1](http://www.ncbi.nlm.nih.gov/gene/?term=6470)(23.000)[ABAT](http://www.ncbi.nlm.nih.gov/gene/?term=18)(23.000)[SLC1A1](http://www.ncbi.nlm.nih.gov/gene/?term=6505)(23.000)[SLC7A2](http://www.ncbi.nlm.nih.gov/gene/?term=6542)(23.000) [GCSH](http://www.ncbi.nlm.nih.gov/gene/?term=2653)(23.000)[SLC7A8](http://www.ncbi.nlm.nih.gov/gene/?term=23428)(23.000)[PPAT](http://www.ncbi.nlm.nih.gov/gene/?term=5471)(23.000)[ACY3](http://www.ncbi.nlm.nih.gov/gene/?term=91703)(23.000)[ASPA](http://www.ncbi.nlm.nih.gov/gene/?term=443)(23.000) [IARS2](http://www.ncbi.nlm.nih.gov/gene/?term=55699)(23.000)[GLRA2](http://www.ncbi.nlm.nih.gov/gene/?term=2742)(23.000)[ALAS1](http://www.ncbi.nlm.nih.gov/gene/?term=211)(23.000)[BCAT1](http://www.ncbi.nlm.nih.gov/gene/?term=586)(23.000)[GCAT](http://www.ncbi.nlm.nih.gov/gene/?term=23464)(23.000) [SLC38A3](http://www.ncbi.nlm.nih.gov/gene/?term=10991)(23.000)[VARS](http://www.ncbi.nlm.nih.gov/gene/?term=7407)(23.000)[NFS1](http://www.ncbi.nlm.nih.gov/gene/?term=9054)(23.000)[SLC25A15](http://www.ncbi.nlm.nih.gov/gene/?term=10166)(23.000)[SLC1A4](http://www.ncbi.nlm.nih.gov/gene/?term=6509)(23.000) [ADSSL1](http://www.ncbi.nlm.nih.gov/gene/?term=122622)(23.000)[VDAC3](http://www.ncbi.nlm.nih.gov/gene/?term=7419)(23.000)[GSS](http://www.ncbi.nlm.nih.gov/gene/?term=2937)(23.000)[ASNS](http://www.ncbi.nlm.nih.gov/gene/?term=440)(23.000)[GLRA3](http://www.ncbi.nlm.nih.gov/gene/?term=8001)(23.000) [DARS2](http://www.ncbi.nlm.nih.gov/gene/?term=55157)(23.000)[GRIN3B](http://www.ncbi.nlm.nih.gov/gene/?term=116444)(23.000)[LCMT1](http://www.ncbi.nlm.nih.gov/gene/?term=51451)(23.000)[GRIN2A](http://www.ncbi.nlm.nih.gov/gene/?term=2903)(23.000)[GOT1](http://www.ncbi.nlm.nih.gov/gene/?term=2805)(23.000) [GNMT](http://www.ncbi.nlm.nih.gov/gene/?term=27232)(23.000)[GLRB](http://www.ncbi.nlm.nih.gov/gene/?term=2743)(23.000)[GLYAT](http://www.ncbi.nlm.nih.gov/gene/?term=10249)(23.000)[AARS2](http://www.ncbi.nlm.nih.gov/gene/?term=57505)(23.000)[ASPH](http://www.ncbi.nlm.nih.gov/gene/?term=444)(23.000) [GPR18](http://www.ncbi.nlm.nih.gov/gene/?term=2841)(23.000)[CTPS1](http://www.ncbi.nlm.nih.gov/gene/?term=1503)(23.000)[SLC25A12](http://www.ncbi.nlm.nih.gov/gene/?term=8604)(23.000)[PHYKPL](http://www.ncbi.nlm.nih.gov/gene/?term=85007)(23.000)[GLYATL1](http://www.ncbi.nlm.nih.gov/gene/?term=92292)(23.000) [GATM](http://www.ncbi.nlm.nih.gov/gene/?term=2628)(23.000)[LARS](http://www.ncbi.nlm.nih.gov/gene/?term=51520)(23.000)[NARS2](http://www.ncbi.nlm.nih.gov/gene/?term=79731)(23.000)[TNNC1](http://www.ncbi.nlm.nih.gov/gene/?term=7134)(23.000)[DARS](http://www.ncbi.nlm.nih.gov/gene/?term=1615)(23.000) [GRIN2C](http://www.ncbi.nlm.nih.gov/gene/?term=2905)(23.000)[OAT](http://www.ncbi.nlm.nih.gov/gene/?term=4942)(23.000)[GLDC](http://www.ncbi.nlm.nih.gov/gene/?term=2731)(23.000)[VDAC2](http://www.ncbi.nlm.nih.gov/gene/?term=7417)(23.000)[OAZ2](http://www.ncbi.nlm.nih.gov/gene/?term=4947)(23.000) [PIPOX](http://www.ncbi.nlm.nih.gov/gene/?term=51268)(23.000)[SLC25A2](http://www.ncbi.nlm.nih.gov/gene/?term=83884)(23.000)[GARS](http://www.ncbi.nlm.nih.gov/gene/?term=2617)(23.000)[GPT](http://www.ncbi.nlm.nih.gov/gene/?term=2875)(23.000)[AARS](http://www.ncbi.nlm.nih.gov/gene/?term=16)(23.000) [SHMT2](http://www.ncbi.nlm.nih.gov/gene/?term=6472)(23.000)[ALAS2](http://www.ncbi.nlm.nih.gov/gene/?term=212)(23.000)[VDAC1](http://www.ncbi.nlm.nih.gov/gene/?term=7416)(23.000)[SLC6A9](http://www.ncbi.nlm.nih.gov/gene/?term=6536)(23.000)[ASRGL1](http://www.ncbi.nlm.nih.gov/gene/?term=80150)(23.000) [GLYATL2](http://www.ncbi.nlm.nih.gov/gene/?term=219970)(23.000)[SLC32A1](http://www.ncbi.nlm.nih.gov/gene/?term=140679)(23.000)[OAZ1](http://www.ncbi.nlm.nih.gov/gene/?term=4946)(23.000)[KARS](http://www.ncbi.nlm.nih.gov/gene/?term=3735)(23.000)[AGXT](http://www.ncbi.nlm.nih.gov/gene/?term=189)(23.000) [LARS2](http://www.ncbi.nlm.nih.gov/gene/?term=23395)(23.000)[RNASE1](http://www.ncbi.nlm.nih.gov/gene/?term=6035)(23.000)[ADSS](http://www.ncbi.nlm.nih.gov/gene/?term=159)(23.000)[SLC36A1](http://www.ncbi.nlm.nih.gov/gene/?term=206358)(23.000)[ACADSB](http://www.ncbi.nlm.nih.gov/gene/?term=36)(23.000) [SLC6A5](http://www.ncbi.nlm.nih.gov/gene/?term=9152)(23.000)[CAD](http://www.ncbi.nlm.nih.gov/gene/?term=790)(23.000)[PCCB](http://www.ncbi.nlm.nih.gov/gene/?term=5096)(23.000)[GLUL](http://www.ncbi.nlm.nih.gov/gene/?term=2752)(23.000)[AGXT2](http://www.ncbi.nlm.nih.gov/gene/?term=64902)(23.000) [IARS](http://www.ncbi.nlm.nih.gov/gene/?term=3376)(23.000)[BCAT2](http://www.ncbi.nlm.nih.gov/gene/?term=587)(23.000)[ACY1](http://www.ncbi.nlm.nih.gov/gene/?term=95)(23.000)[KYNU](http://www.ncbi.nlm.nih.gov/gene/?term=8942)(23.000)[SLC25A13](http://www.ncbi.nlm.nih.gov/gene/?term=10165)(23.000) [GLRA1](http://www.ncbi.nlm.nih.gov/gene/?term=2741)(23.000)[LYZ](http://www.ncbi.nlm.nih.gov/gene/?term=4069)(23.000)[LCMT2](http://www.ncbi.nlm.nih.gov/gene/?term=9836)(23.000)[PAICS](http://www.ncbi.nlm.nih.gov/gene/?term=10606)(23.000)[NARS](http://www.ncbi.nlm.nih.gov/gene/?term=4677)(23.000) [DGAT2](http://www.ncbi.nlm.nih.gov/gene/?term=84649)(22.373)[CYBA](http://www.ncbi.nlm.nih.gov/gene/?term=1535)(22.373)[RARS](http://www.ncbi.nlm.nih.gov/gene/?term=5917)(22.373)[P2RX2](http://www.ncbi.nlm.nih.gov/gene/?term=22953)(22.373)[TPH2](http://www.ncbi.nlm.nih.gov/gene/?term=121278)(22.373) [EDN1](http://www.ncbi.nlm.nih.gov/gene/?term=1906)(22.373)[SLC24A2](http://www.ncbi.nlm.nih.gov/gene/?term=25769)(22.373)[PRLR](http://www.ncbi.nlm.nih.gov/gene/?term=5618)(22.373)[HTR2B](http://www.ncbi.nlm.nih.gov/gene/?term=3357)(22.373)[PAOX](http://www.ncbi.nlm.nih.gov/gene/?term=196743)(22.373) [MTRR](http://www.ncbi.nlm.nih.gov/gene/?term=4552)(22.373)[TH](http://www.ncbi.nlm.nih.gov/gene/?term=7054)(22.373)[EDN3](http://www.ncbi.nlm.nih.gov/gene/?term=1908)(22.373)[IL1B](http://www.ncbi.nlm.nih.gov/gene/?term=3553)(22.373)[BAK1](http://www.ncbi.nlm.nih.gov/gene/?term=578)(22.373) [POR](http://www.ncbi.nlm.nih.gov/gene/?term=5447)(22.373)[PQLC2](http://www.ncbi.nlm.nih.gov/gene/?term=54896)(22.373)[HMBS](http://www.ncbi.nlm.nih.gov/gene/?term=3145)(22.373)[TPH1](http://www.ncbi.nlm.nih.gov/gene/?term=7166)(22.373)[TAF7](http://www.ncbi.nlm.nih.gov/gene/?term=6879)(22.373) [BCL2L11](http://www.ncbi.nlm.nih.gov/gene/?term=10018)(22.373)[ECE1](http://www.ncbi.nlm.nih.gov/gene/?term=1889)(22.373)[FCER2](http://www.ncbi.nlm.nih.gov/gene/?term=2208)(22.373)[NDOR1](http://www.ncbi.nlm.nih.gov/gene/?term=27158)(22.373)[IFNG](http://www.ncbi.nlm.nih.gov/gene/?term=3458)(22.373) [F2RL1](http://www.ncbi.nlm.nih.gov/gene/?term=2150)(22.373)[DDC](http://www.ncbi.nlm.nih.gov/gene/?term=1644)(22.373)[PAM](http://www.ncbi.nlm.nih.gov/gene/?term=5066)(22.373)[SLC11A1](http://www.ncbi.nlm.nih.gov/gene/?term=6556)(22.373)[AIF1](http://www.ncbi.nlm.nih.gov/gene/?term=199)(22.373) [SLC3A1](http://www.ncbi.nlm.nih.gov/gene/?term=6519)(22.373)[SLC11A2](http://www.ncbi.nlm.nih.gov/gene/?term=4891)(22.373)[MT3](http://www.ncbi.nlm.nih.gov/gene/?term=4504)(22.373)[SLC7A7](http://www.ncbi.nlm.nih.gov/gene/?term=9056)(22.373)[UROS](http://www.ncbi.nlm.nih.gov/gene/?term=7390)(22.373) [CPS1](http://www.ncbi.nlm.nih.gov/gene/?term=1373)(22.373)[BAD](http://www.ncbi.nlm.nih.gov/gene/?term=572)(22.373)[LCN2](http://www.ncbi.nlm.nih.gov/gene/?term=3934)(22.373)[ODC1](http://www.ncbi.nlm.nih.gov/gene/?term=4953)(22.373)[ADSL](http://www.ncbi.nlm.nih.gov/gene/?term=158)(22.373) [NAGS](http://www.ncbi.nlm.nih.gov/gene/?term=162417)(22.373)[CERS1](http://www.ncbi.nlm.nih.gov/gene/?term=10715)(22.373)[NOS1](http://www.ncbi.nlm.nih.gov/gene/?term=4842)(22.373)[EDN2](http://www.ncbi.nlm.nih.gov/gene/?term=1907)(22.373) | |
| [5-Methoxyfuraldehyde](http://www.megabionet.org/tcmid/ingredient/13924/) | This compound doesn't have any potential target with score larger than 20. | |
| [Pyridine](http://www.megabionet.org/tcmid/ingredient/23886/) | This compound doesn't have any potential target with score larger than 20. | |
| [Glycyrrhetinic Acid](http://www.megabionet.org/tcmid/ingredient/23291/) | [AR](http://www.ncbi.nlm.nih.gov/gene/?term=367)(122.778)[NR3C1](http://www.ncbi.nlm.nih.gov/gene/?term=2908)(122.778)[ANXA1](http://www.ncbi.nlm.nih.gov/gene/?term=301)(122.778)[PTGER1](http://www.ncbi.nlm.nih.gov/gene/?term=5731)(80.882)[PTGER4](http://www.ncbi.nlm.nih.gov/gene/?term=5734)(80.882) [PTGER2](http://www.ncbi.nlm.nih.gov/gene/?term=5732)(80.882)[PTGER3](http://www.ncbi.nlm.nih.gov/gene/?term=5733)(80.882)[CD300A](http://www.ncbi.nlm.nih.gov/gene/?term=11314)(55.444)[KIF14](http://www.ncbi.nlm.nih.gov/gene/?term=9928)(55.444)[SRD5A1](http://www.ncbi.nlm.nih.gov/gene/?term=6715)(48.000) [CYP17A1](http://www.ncbi.nlm.nih.gov/gene/?term=1586)(48.000)[SLC8A1](http://www.ncbi.nlm.nih.gov/gene/?term=6546)(48.000)[F12](http://www.ncbi.nlm.nih.gov/gene/?term=2161)(48.000)[PRLR](http://www.ncbi.nlm.nih.gov/gene/?term=5618)(48.000)[ESR1](http://www.ncbi.nlm.nih.gov/gene/?term=2099)(48.000) [TRPV1](http://www.ncbi.nlm.nih.gov/gene/?term=7442)(48.000)[PPARG](http://www.ncbi.nlm.nih.gov/gene/?term=5468)(48.000)[FFAR1](http://www.ncbi.nlm.nih.gov/gene/?term=2864)(48.000)[PGR](http://www.ncbi.nlm.nih.gov/gene/?term=5241)(48.000)[OPRK1](http://www.ncbi.nlm.nih.gov/gene/?term=4986)(48.000) [FADS2](http://www.ncbi.nlm.nih.gov/gene/?term=9415)(48.000)[FADS1](http://www.ncbi.nlm.nih.gov/gene/?term=3992)(48.000)[PPARD](http://www.ncbi.nlm.nih.gov/gene/?term=5467)(48.000)[PTGS1](http://www.ncbi.nlm.nih.gov/gene/?term=5742)(48.000)[ACSL4](http://www.ncbi.nlm.nih.gov/gene/?term=2182)(48.000) [PTGS2](http://www.ncbi.nlm.nih.gov/gene/?term=5743)(48.000)[ELOVL4](http://www.ncbi.nlm.nih.gov/gene/?term=6785)(48.000)[HSD11B1](http://www.ncbi.nlm.nih.gov/gene/?term=3290)(48.000)[ACSL3](http://www.ncbi.nlm.nih.gov/gene/?term=2181)(48.000)[NR3C2](http://www.ncbi.nlm.nih.gov/gene/?term=4306)(48.000) [DAPK2](http://www.ncbi.nlm.nih.gov/gene/?term=23604)(22.373)[HPGD](http://www.ncbi.nlm.nih.gov/gene/?term=3248)(22.373)[ADAM8](http://www.ncbi.nlm.nih.gov/gene/?term=101)(22.373)[CCL24](http://www.ncbi.nlm.nih.gov/gene/?term=6369)(22.373)[IL4](http://www.ncbi.nlm.nih.gov/gene/?term=3565)(22.373) | |
| [Licoricesaponine F3](http://www.megabionet.org/tcmid/ingredient/31435/) | [GLRA3](http://www.ncbi.nlm.nih.gov/gene/?term=8001)(48.000)[NR3C1](http://www.ncbi.nlm.nih.gov/gene/?term=2908)(48.000)[ATP1A1](http://www.ncbi.nlm.nih.gov/gene/?term=476)(48.000)[GABRB3](http://www.ncbi.nlm.nih.gov/gene/?term=2562)(48.000) | |
| [Corylifolinin](http://www.megabionet.org/tcmid/ingredient/4108/) | [HSD17B1](http://www.ncbi.nlm.nih.gov/gene/?term=3292)(80.882)[ESR1](http://www.ncbi.nlm.nih.gov/gene/?term=2099)(23.000)[CFTR](http://www.ncbi.nlm.nih.gov/gene/?term=1080)(23.000)[AKR1C3](http://www.ncbi.nlm.nih.gov/gene/?term=8644)(22.373)[HSD17B6](http://www.ncbi.nlm.nih.gov/gene/?term=8630)(22.373) [HSD17B2](http://www.ncbi.nlm.nih.gov/gene/?term=3294)(22.373)[CYP11A1](http://www.ncbi.nlm.nih.gov/gene/?term=1583)(22.373)[WNT4](http://www.ncbi.nlm.nih.gov/gene/?term=54361)(22.373)[STAR](http://www.ncbi.nlm.nih.gov/gene/?term=6770)(22.373)[HSD17B8](http://www.ncbi.nlm.nih.gov/gene/?term=7923)(22.373) [DHRS9](http://www.ncbi.nlm.nih.gov/gene/?term=10170)(22.373)[HSD17B3](http://www.ncbi.nlm.nih.gov/gene/?term=3293)(22.373) | |
| [18alpha-Glycyrrhetinic Acid](http://www.megabionet.org/tcmid/ingredient/23166/) | [AR](http://www.ncbi.nlm.nih.gov/gene/?term=367)(122.778)[NR3C1](http://www.ncbi.nlm.nih.gov/gene/?term=2908)(122.778)[ANXA1](http://www.ncbi.nlm.nih.gov/gene/?term=301)(122.778)[PTGER1](http://www.ncbi.nlm.nih.gov/gene/?term=5731)(80.882)[PTGER4](http://www.ncbi.nlm.nih.gov/gene/?term=5734)(80.882) [PTGER2](http://www.ncbi.nlm.nih.gov/gene/?term=5732)(80.882)[PTGER3](http://www.ncbi.nlm.nih.gov/gene/?term=5733)(80.882)[CD300A](http://www.ncbi.nlm.nih.gov/gene/?term=11314)(55.444)[KIF14](http://www.ncbi.nlm.nih.gov/gene/?term=9928)(55.444)[SRD5A1](http://www.ncbi.nlm.nih.gov/gene/?term=6715)(48.000) [CYP17A1](http://www.ncbi.nlm.nih.gov/gene/?term=1586)(48.000)[SLC8A1](http://www.ncbi.nlm.nih.gov/gene/?term=6546)(48.000)[F12](http://www.ncbi.nlm.nih.gov/gene/?term=2161)(48.000)[PRLR](http://www.ncbi.nlm.nih.gov/gene/?term=5618)(48.000)[ESR1](http://www.ncbi.nlm.nih.gov/gene/?term=2099)(48.000) [TRPV1](http://www.ncbi.nlm.nih.gov/gene/?term=7442)(48.000)[PPARG](http://www.ncbi.nlm.nih.gov/gene/?term=5468)(48.000)[FFAR1](http://www.ncbi.nlm.nih.gov/gene/?term=2864)(48.000)[PGR](http://www.ncbi.nlm.nih.gov/gene/?term=5241)(48.000)[OPRK1](http://www.ncbi.nlm.nih.gov/gene/?term=4986)(48.000) [FADS2](http://www.ncbi.nlm.nih.gov/gene/?term=9415)(48.000)[FADS1](http://www.ncbi.nlm.nih.gov/gene/?term=3992)(48.000)[PPARD](http://www.ncbi.nlm.nih.gov/gene/?term=5467)(48.000)[PTGS1](http://www.ncbi.nlm.nih.gov/gene/?term=5742)(48.000)[ACSL4](http://www.ncbi.nlm.nih.gov/gene/?term=2182)(48.000) [PTGS2](http://www.ncbi.nlm.nih.gov/gene/?term=5743)(48.000)[ELOVL4](http://www.ncbi.nlm.nih.gov/gene/?term=6785)(48.000)[HSD11B1](http://www.ncbi.nlm.nih.gov/gene/?term=3290)(48.000)[ACSL3](http://www.ncbi.nlm.nih.gov/gene/?term=2181)(48.000)[NR3C2](http://www.ncbi.nlm.nih.gov/gene/?term=4306)(48.000) [DAPK2](http://www.ncbi.nlm.nih.gov/gene/?term=23604)(22.373)[HPGD](http://www.ncbi.nlm.nih.gov/gene/?term=3248)(22.373)[ADAM8](http://www.ncbi.nlm.nih.gov/gene/?term=101)(22.373)[CCL24](http://www.ncbi.nlm.nih.gov/gene/?term=6369)(22.373)[IL4](http://www.ncbi.nlm.nih.gov/gene/?term=3565)(22.373) | |
| [Scutevulin](http://www.megabionet.org/tcmid/ingredient/19573/) | This compound doesn't have any potential target with score larger than 20. | |
| [Octanol](http://www.megabionet.org/tcmid/ingredient/15967/) | [TRPV3](http://www.ncbi.nlm.nih.gov/gene/?term=162514)(122.778)[OPRK1](http://www.ncbi.nlm.nih.gov/gene/?term=4986)(122.778)[TRPM8](http://www.ncbi.nlm.nih.gov/gene/?term=79054)(122.778)[TRPA1](http://www.ncbi.nlm.nih.gov/gene/?term=8989)(122.778)[KCNK4](http://www.ncbi.nlm.nih.gov/gene/?term=50801)(55.444) [SCN11A](http://www.ncbi.nlm.nih.gov/gene/?term=11280)(48.000)[SCN2B](http://www.ncbi.nlm.nih.gov/gene/?term=6327)(48.000)[ABAT](http://www.ncbi.nlm.nih.gov/gene/?term=18)(48.000)[SCN1A](http://www.ncbi.nlm.nih.gov/gene/?term=6323)(48.000)[SCN3B](http://www.ncbi.nlm.nih.gov/gene/?term=55800)(48.000) [SCN3A](http://www.ncbi.nlm.nih.gov/gene/?term=6328)(48.000)[SCN7A](http://www.ncbi.nlm.nih.gov/gene/?term=6332)(48.000)[SCN5A](http://www.ncbi.nlm.nih.gov/gene/?term=6331)(48.000)[SCN10A](http://www.ncbi.nlm.nih.gov/gene/?term=6336)(48.000)[AKR1D1](http://www.ncbi.nlm.nih.gov/gene/?term=6718)(48.000) [ALDH5A1](http://www.ncbi.nlm.nih.gov/gene/?term=7915)(48.000)[SCN2A](http://www.ncbi.nlm.nih.gov/gene/?term=6326)(48.000)[HDAC9](http://www.ncbi.nlm.nih.gov/gene/?term=9734)(48.000)[TYR](http://www.ncbi.nlm.nih.gov/gene/?term=7299)(48.000)[SCN9A](http://www.ncbi.nlm.nih.gov/gene/?term=6335)(48.000) [SCN4A](http://www.ncbi.nlm.nih.gov/gene/?term=6329)(48.000)[SCN4B](http://www.ncbi.nlm.nih.gov/gene/?term=6330)(48.000)[SRD5A2](http://www.ncbi.nlm.nih.gov/gene/?term=6716)(48.000)[ACADSB](http://www.ncbi.nlm.nih.gov/gene/?term=36)(48.000)[SCN8A](http://www.ncbi.nlm.nih.gov/gene/?term=6334)(48.000) [SCN1B](http://www.ncbi.nlm.nih.gov/gene/?term=6324)(48.000)[OGDH](http://www.ncbi.nlm.nih.gov/gene/?term=4967)(48.000)[HDAC2](http://www.ncbi.nlm.nih.gov/gene/?term=3066)(48.000)[ACTN3](http://www.ncbi.nlm.nih.gov/gene/?term=89)(22.373)[TAC1](http://www.ncbi.nlm.nih.gov/gene/?term=6863)(22.373) [NGFR](http://www.ncbi.nlm.nih.gov/gene/?term=4804)(22.373)[ADRA2A](http://www.ncbi.nlm.nih.gov/gene/?term=150)(22.373)[TRPV1](http://www.ncbi.nlm.nih.gov/gene/?term=7442)(22.373)[FGF10](http://www.ncbi.nlm.nih.gov/gene/?term=2255)(22.373)[VCP](http://www.ncbi.nlm.nih.gov/gene/?term=7415)(22.373) [HIF1A](http://www.ncbi.nlm.nih.gov/gene/?term=3091)(22.373)[BNIP3](http://www.ncbi.nlm.nih.gov/gene/?term=664)(22.373)[CRH](http://www.ncbi.nlm.nih.gov/gene/?term=1392)(22.373)[NEFL](http://www.ncbi.nlm.nih.gov/gene/?term=4747)(22.373)[UCN2](http://www.ncbi.nlm.nih.gov/gene/?term=90226)(22.373) [SMO](http://www.ncbi.nlm.nih.gov/gene/?term=6608)(22.373)[TACR2](http://www.ncbi.nlm.nih.gov/gene/?term=6865)(22.373)[AQP1](http://www.ncbi.nlm.nih.gov/gene/?term=358)(22.373)[TAC4](http://www.ncbi.nlm.nih.gov/gene/?term=255061)(22.373)[CDH3](http://www.ncbi.nlm.nih.gov/gene/?term=1001)(22.373) [CBFA2T3](http://www.ncbi.nlm.nih.gov/gene/?term=863)(22.373)[NEFH](http://www.ncbi.nlm.nih.gov/gene/?term=4744)(22.373)[CRHR2](http://www.ncbi.nlm.nih.gov/gene/?term=1395)(22.373) | |
| [Gancaonin C](http://www.megabionet.org/tcmid/ingredient/8135/) | This compound doesn't have any potential target with score larger than 20. | |
| [Methyl Octadecadienoate](http://www.megabionet.org/tcmid/ingredient/23336/) | [SLC8A1](http://www.ncbi.nlm.nih.gov/gene/?term=6546)(122.778)[TRPV1](http://www.ncbi.nlm.nih.gov/gene/?term=7442)(122.778)[FADS2](http://www.ncbi.nlm.nih.gov/gene/?term=9415)(122.778)[FADS1](http://www.ncbi.nlm.nih.gov/gene/?term=3992)(122.778)[PTGS1](http://www.ncbi.nlm.nih.gov/gene/?term=5742)(122.778) [PTGS2](http://www.ncbi.nlm.nih.gov/gene/?term=5743)(122.778)[ELOVL4](http://www.ncbi.nlm.nih.gov/gene/?term=6785)(122.778)[F12](http://www.ncbi.nlm.nih.gov/gene/?term=2161)(48.000)[PPARG](http://www.ncbi.nlm.nih.gov/gene/?term=5468)(48.000)[FFAR1](http://www.ncbi.nlm.nih.gov/gene/?term=2864)(48.000) [PTGER4](http://www.ncbi.nlm.nih.gov/gene/?term=5734)(48.000)[AR](http://www.ncbi.nlm.nih.gov/gene/?term=367)(48.000)[PPARD](http://www.ncbi.nlm.nih.gov/gene/?term=5467)(48.000)[PTGER2](http://www.ncbi.nlm.nih.gov/gene/?term=5732)(48.000)[ACSL4](http://www.ncbi.nlm.nih.gov/gene/?term=2182)(48.000) [PTGER3](http://www.ncbi.nlm.nih.gov/gene/?term=5733)(48.000)[CYP19A1](http://www.ncbi.nlm.nih.gov/gene/?term=1588)(48.000)[ACSL3](http://www.ncbi.nlm.nih.gov/gene/?term=2181)(48.000) | |
| [4'-O-Methylglabridin](http://www.megabionet.org/tcmid/ingredient/14458/) | [CNR2](http://www.ncbi.nlm.nih.gov/gene/?term=1269)(80.882)[CNR1](http://www.ncbi.nlm.nih.gov/gene/?term=1268)(80.882)[DRD2](http://www.ncbi.nlm.nih.gov/gene/?term=1813)(55.444)[SEC14L3](http://www.ncbi.nlm.nih.gov/gene/?term=266629)(23.000)[PPP2CA](http://www.ncbi.nlm.nih.gov/gene/?term=5515)(23.000) [PRKCA](http://www.ncbi.nlm.nih.gov/gene/?term=5578)(23.000)[NR1I2](http://www.ncbi.nlm.nih.gov/gene/?term=8856)(23.000)[ALOX5](http://www.ncbi.nlm.nih.gov/gene/?term=240)(23.000)[PPP2CB](http://www.ncbi.nlm.nih.gov/gene/?term=5516)(23.000)[SEC14L2](http://www.ncbi.nlm.nih.gov/gene/?term=23541)(23.000) [DGKA](http://www.ncbi.nlm.nih.gov/gene/?term=1606)(23.000)[PRKCB](http://www.ncbi.nlm.nih.gov/gene/?term=5579)(23.000)[SEC14L4](http://www.ncbi.nlm.nih.gov/gene/?term=284904)(23.000)[AKT1](http://www.ncbi.nlm.nih.gov/gene/?term=207)(22.373)[CCR7](http://www.ncbi.nlm.nih.gov/gene/?term=1236)(22.373) [ABHD6](http://www.ncbi.nlm.nih.gov/gene/?term=57406)(22.373)[MGLL](http://www.ncbi.nlm.nih.gov/gene/?term=11343)(22.373)[FCER1G](http://www.ncbi.nlm.nih.gov/gene/?term=2207)(22.373)[SUMO1](http://www.ncbi.nlm.nih.gov/gene/?term=7341)(22.373)[GPR55](http://www.ncbi.nlm.nih.gov/gene/?term=9290)(22.373) [CHRNB2](http://www.ncbi.nlm.nih.gov/gene/?term=1141)(22.373)[FCER1A](http://www.ncbi.nlm.nih.gov/gene/?term=2205)(22.373)[CAV3](http://www.ncbi.nlm.nih.gov/gene/?term=859)(22.373)[RNF207](http://www.ncbi.nlm.nih.gov/gene/?term=388591)(22.373)[C3](http://www.ncbi.nlm.nih.gov/gene/?term=718)(22.373) [DAGLA](http://www.ncbi.nlm.nih.gov/gene/?term=747)(22.373)[PLIN5](http://www.ncbi.nlm.nih.gov/gene/?term=440503)(22.373)[ZP3](http://www.ncbi.nlm.nih.gov/gene/?term=7784)(22.373) | |
| [Isoschaftoside](http://www.megabionet.org/tcmid/ingredient/11694/) | This compound doesn't have any potential target with score larger than 20. | |
| [Monoammonium Glycyrrhizinate](http://www.megabionet.org/tcmid/ingredient/23259/) | [NR3C1](http://www.ncbi.nlm.nih.gov/gene/?term=2908)(48.000)[ATP1A1](http://www.ncbi.nlm.nih.gov/gene/?term=476)(48.000) | |
| [Isoliensinine](http://www.megabionet.org/tcmid/ingredient/11488/) | [ACHE](http://www.ncbi.nlm.nih.gov/gene/?term=43)(122.778)[CHRNA2](http://www.ncbi.nlm.nih.gov/gene/?term=1135)(122.778)[HTR3A](http://www.ncbi.nlm.nih.gov/gene/?term=3359)(122.778)[CHRM2](http://www.ncbi.nlm.nih.gov/gene/?term=1129)(122.778)[CHRM3](http://www.ncbi.nlm.nih.gov/gene/?term=1131)(48.000) [DRD2](http://www.ncbi.nlm.nih.gov/gene/?term=1813)(48.000)[SLC18A2](http://www.ncbi.nlm.nih.gov/gene/?term=6571)(48.000)[BCHE](http://www.ncbi.nlm.nih.gov/gene/?term=590)(48.000) | |
| [Guanosine](http://www.megabionet.org/tcmid/ingredient/9070/) | [POLA1](http://www.ncbi.nlm.nih.gov/gene/?term=5422)(122.778)[ADORA2A](http://www.ncbi.nlm.nih.gov/gene/?term=135)(80.882)[POLB](http://www.ncbi.nlm.nih.gov/gene/?term=5423)(80.882)[DNMT1](http://www.ncbi.nlm.nih.gov/gene/?term=1786)(80.882)[PNP](http://www.ncbi.nlm.nih.gov/gene/?term=4860)(48.000) [ADORA2B](http://www.ncbi.nlm.nih.gov/gene/?term=136)(48.000)[ADORA3](http://www.ncbi.nlm.nih.gov/gene/?term=140)(48.000)[ADORA1](http://www.ncbi.nlm.nih.gov/gene/?term=134)(48.000)[TCN1](http://www.ncbi.nlm.nih.gov/gene/?term=6947)(23.000)[MUT](http://www.ncbi.nlm.nih.gov/gene/?term=4594)(23.000) [MMAB](http://www.ncbi.nlm.nih.gov/gene/?term=326625)(23.000)[MTRR](http://www.ncbi.nlm.nih.gov/gene/?term=4552)(23.000)[MTR](http://www.ncbi.nlm.nih.gov/gene/?term=4548)(23.000)[CUBN](http://www.ncbi.nlm.nih.gov/gene/?term=8029)(23.000)[AMN](http://www.ncbi.nlm.nih.gov/gene/?term=81693)(23.000) [MMACHC](http://www.ncbi.nlm.nih.gov/gene/?term=25974)(23.000)[MMAA](http://www.ncbi.nlm.nih.gov/gene/?term=166785)(23.000)[UHRF1](http://www.ncbi.nlm.nih.gov/gene/?term=29128)(22.373)[DNMT3A](http://www.ncbi.nlm.nih.gov/gene/?term=1788)(22.373)[POLE](http://www.ncbi.nlm.nih.gov/gene/?term=5426)(22.373) [BRCA1](http://www.ncbi.nlm.nih.gov/gene/?term=672)(22.373)[PRKDC](http://www.ncbi.nlm.nih.gov/gene/?term=5591)(22.373)[KDM1A](http://www.ncbi.nlm.nih.gov/gene/?term=23028)(22.373)[POLG](http://www.ncbi.nlm.nih.gov/gene/?term=5428)(22.373)[LIG4](http://www.ncbi.nlm.nih.gov/gene/?term=3981)(22.373) [USP7](http://www.ncbi.nlm.nih.gov/gene/?term=7874)(22.373)[TCF3](http://www.ncbi.nlm.nih.gov/gene/?term=6929)(22.373)[POLD1](http://www.ncbi.nlm.nih.gov/gene/?term=5424)(22.373)[PAX5](http://www.ncbi.nlm.nih.gov/gene/?term=5079)(22.373)[TRDMT1](http://www.ncbi.nlm.nih.gov/gene/?term=1787)(22.373) [TACR2](http://www.ncbi.nlm.nih.gov/gene/?term=6865)(22.373)[UHRF2](http://www.ncbi.nlm.nih.gov/gene/?term=115426)(22.373)[DNMT3B](http://www.ncbi.nlm.nih.gov/gene/?term=1789)(22.373)[CTCF](http://www.ncbi.nlm.nih.gov/gene/?term=10664)(22.373)[KDM3A](http://www.ncbi.nlm.nih.gov/gene/?term=55818)(22.373) [MGMT](http://www.ncbi.nlm.nih.gov/gene/?term=4255)(22.373)[HELLS](http://www.ncbi.nlm.nih.gov/gene/?term=3070)(22.373) | |
| [P-Hydroxybenzyl Acetone](http://www.megabionet.org/tcmid/ingredient/9830/) | [PTGS1](http://www.ncbi.nlm.nih.gov/gene/?term=5742)(122.778)[PTGS2](http://www.ncbi.nlm.nih.gov/gene/?term=5743)(122.778)[ESR1](http://www.ncbi.nlm.nih.gov/gene/?term=2099)(80.882)[ADRA2A](http://www.ncbi.nlm.nih.gov/gene/?term=150)(48.000)[TH](http://www.ncbi.nlm.nih.gov/gene/?term=7054)(48.000) [TAT](http://www.ncbi.nlm.nih.gov/gene/?term=6898)(48.000)[DRD2](http://www.ncbi.nlm.nih.gov/gene/?term=1813)(48.000)[DDC](http://www.ncbi.nlm.nih.gov/gene/?term=1644)(48.000)[DRD4](http://www.ncbi.nlm.nih.gov/gene/?term=1815)(48.000)[DRD3](http://www.ncbi.nlm.nih.gov/gene/?term=1814)(48.000) [ALOX5](http://www.ncbi.nlm.nih.gov/gene/?term=240)(48.000)[YARS](http://www.ncbi.nlm.nih.gov/gene/?term=8565)(48.000)[DRD5](http://www.ncbi.nlm.nih.gov/gene/?term=1816)(48.000)[DRD1](http://www.ncbi.nlm.nih.gov/gene/?term=1812)(48.000)[YARS2](http://www.ncbi.nlm.nih.gov/gene/?term=51067)(48.000) [HSD17B1](http://www.ncbi.nlm.nih.gov/gene/?term=3292)(23.000)[TFAP2C](http://www.ncbi.nlm.nih.gov/gene/?term=7022)(22.373)[TOX3](http://www.ncbi.nlm.nih.gov/gene/?term=27324)(22.373)[TGFB1](http://www.ncbi.nlm.nih.gov/gene/?term=7040)(22.373)[MED1](http://www.ncbi.nlm.nih.gov/gene/?term=5469)(22.373) [NKX3-1](http://www.ncbi.nlm.nih.gov/gene/?term=4824)(22.373)[ESR2](http://www.ncbi.nlm.nih.gov/gene/?term=2100)(22.373)[SHH](http://www.ncbi.nlm.nih.gov/gene/?term=6469)(22.373)[LEF1](http://www.ncbi.nlm.nih.gov/gene/?term=51176)(22.373)[FGFR2](http://www.ncbi.nlm.nih.gov/gene/?term=2263)(22.373) [PGR](http://www.ncbi.nlm.nih.gov/gene/?term=5241)(22.373)[WNT4](http://www.ncbi.nlm.nih.gov/gene/?term=54361)(22.373)[AREG](http://www.ncbi.nlm.nih.gov/gene/?term=374)(22.373)[GPER1](http://www.ncbi.nlm.nih.gov/gene/?term=2852)(22.373)[WNT5A](http://www.ncbi.nlm.nih.gov/gene/?term=7474)(22.373) [SOX9](http://www.ncbi.nlm.nih.gov/gene/?term=6662)(22.373)[VDR](http://www.ncbi.nlm.nih.gov/gene/?term=7421)(22.373)[TRIM24](http://www.ncbi.nlm.nih.gov/gene/?term=8805)(22.373) | |
| [Scutellarein](http://www.megabionet.org/tcmid/ingredient/19567/) | This compound doesn't have any potential target with score larger than 20. | |
| [Licoricesaponine A3](http://www.megabionet.org/tcmid/ingredient/31431/) | [NR3C1](http://www.ncbi.nlm.nih.gov/gene/?term=2908)(48.000)[ATP1A1](http://www.ncbi.nlm.nih.gov/gene/?term=476)(48.000)[ANXA1](http://www.ncbi.nlm.nih.gov/gene/?term=301)(48.000) | |
| [3,3'-Dimethylquercetin](http://www.megabionet.org/tcmid/ingredient/6401/) | This compound doesn't have any potential target with score larger than 20. | |
| [1-Pentadecene](http://www.megabionet.org/tcmid/ingredient/16820/) | [KCND1](http://www.ncbi.nlm.nih.gov/gene/?term=3750)(80.882)[KCNA3](http://www.ncbi.nlm.nih.gov/gene/?term=3738)(80.882)[PRKAB1](http://www.ncbi.nlm.nih.gov/gene/?term=5564)(80.882)[ADH1A](http://www.ncbi.nlm.nih.gov/gene/?term=124)(80.882)[KCNA10](http://www.ncbi.nlm.nih.gov/gene/?term=3744)(80.882) [GAMT](http://www.ncbi.nlm.nih.gov/gene/?term=2593)(80.882)[KCNC3](http://www.ncbi.nlm.nih.gov/gene/?term=3748)(80.882)[KCNA1](http://www.ncbi.nlm.nih.gov/gene/?term=3736)(80.882)[KCNA2](http://www.ncbi.nlm.nih.gov/gene/?term=3737)(80.882)[TPO](http://www.ncbi.nlm.nih.gov/gene/?term=7173)(80.882) [CAT](http://www.ncbi.nlm.nih.gov/gene/?term=847)(80.882)[KCNB1](http://www.ncbi.nlm.nih.gov/gene/?term=3745)(80.882)[ADH1B](http://www.ncbi.nlm.nih.gov/gene/?term=125)(80.882)[DLG4](http://www.ncbi.nlm.nih.gov/gene/?term=1742)(80.882)[KCNC2](http://www.ncbi.nlm.nih.gov/gene/?term=3747)(80.882) [KCNC1](http://www.ncbi.nlm.nih.gov/gene/?term=3746)(80.882)[ADH1C](http://www.ncbi.nlm.nih.gov/gene/?term=126)(80.882)[KCNB2](http://www.ncbi.nlm.nih.gov/gene/?term=9312)(80.882)[KCNA5](http://www.ncbi.nlm.nih.gov/gene/?term=3741)(80.882)[RNASE1](http://www.ncbi.nlm.nih.gov/gene/?term=6035)(80.882) [KCND2](http://www.ncbi.nlm.nih.gov/gene/?term=3751)(80.882)[ALDH2](http://www.ncbi.nlm.nih.gov/gene/?term=217)(80.882)[KCNA4](http://www.ncbi.nlm.nih.gov/gene/?term=3739)(80.882)[KCNA7](http://www.ncbi.nlm.nih.gov/gene/?term=3743)(80.882)[KCNA6](http://www.ncbi.nlm.nih.gov/gene/?term=3742)(80.882) [KCND3](http://www.ncbi.nlm.nih.gov/gene/?term=3752)(80.882)[GUCY1B3](http://www.ncbi.nlm.nih.gov/gene/?term=2983)(55.444)[GATM](http://www.ncbi.nlm.nih.gov/gene/?term=2628)(55.444)[KCNK4](http://www.ncbi.nlm.nih.gov/gene/?term=50801)(55.444)[IYD](http://www.ncbi.nlm.nih.gov/gene/?term=389434)(55.444) [KCNQ1](http://www.ncbi.nlm.nih.gov/gene/?term=3784)(55.444)[FBP1](http://www.ncbi.nlm.nih.gov/gene/?term=2203)(22.373)[DGKI](http://www.ncbi.nlm.nih.gov/gene/?term=9162)(22.373)[ADH7](http://www.ncbi.nlm.nih.gov/gene/?term=131)(22.373)[NFIB](http://www.ncbi.nlm.nih.gov/gene/?term=4781)(22.373) [CYGB](http://www.ncbi.nlm.nih.gov/gene/?term=114757)(22.373)[NRXN3](http://www.ncbi.nlm.nih.gov/gene/?term=9369)(22.373)[LRRC4B](http://www.ncbi.nlm.nih.gov/gene/?term=94030)(22.373)[AQP8](http://www.ncbi.nlm.nih.gov/gene/?term=343)(22.373)[ARX](http://www.ncbi.nlm.nih.gov/gene/?term=170302)(22.373) [IGF1](http://www.ncbi.nlm.nih.gov/gene/?term=3479)(22.373)[SHANK3](http://www.ncbi.nlm.nih.gov/gene/?term=85358)(22.373)[ACY3](http://www.ncbi.nlm.nih.gov/gene/?term=91703)(22.373)[ASPA](http://www.ncbi.nlm.nih.gov/gene/?term=443)(22.373)[NRXN1](http://www.ncbi.nlm.nih.gov/gene/?term=9378)(22.373) [EDN1](http://www.ncbi.nlm.nih.gov/gene/?term=1906)(22.373)[OXT](http://www.ncbi.nlm.nih.gov/gene/?term=5020)(22.373)[MAGI2](http://www.ncbi.nlm.nih.gov/gene/?term=9863)(22.373)[APOE](http://www.ncbi.nlm.nih.gov/gene/?term=348)(22.373)[SOX15](http://www.ncbi.nlm.nih.gov/gene/?term=6665)(22.373) [CNTNAP4](http://www.ncbi.nlm.nih.gov/gene/?term=85445)(22.373)[RNASE4](http://www.ncbi.nlm.nih.gov/gene/?term=6038)(22.373)[RAB3A](http://www.ncbi.nlm.nih.gov/gene/?term=5864)(22.373)[MC4R](http://www.ncbi.nlm.nih.gov/gene/?term=4160)(22.373)[IL1B](http://www.ncbi.nlm.nih.gov/gene/?term=3553)(22.373) [GRIN2A](http://www.ncbi.nlm.nih.gov/gene/?term=2903)(22.373)[SLC17A7](http://www.ncbi.nlm.nih.gov/gene/?term=57030)(22.373)[CYP11A1](http://www.ncbi.nlm.nih.gov/gene/?term=1583)(22.373)[SCN5A](http://www.ncbi.nlm.nih.gov/gene/?term=6331)(22.373)[SCN10A](http://www.ncbi.nlm.nih.gov/gene/?term=6336)(22.373) [SPARC](http://www.ncbi.nlm.nih.gov/gene/?term=6678)(22.373)[SORCS3](http://www.ncbi.nlm.nih.gov/gene/?term=22986)(22.373)[RNASE8](http://www.ncbi.nlm.nih.gov/gene/?term=122665)(22.373)[KCNIP2](http://www.ncbi.nlm.nih.gov/gene/?term=30819)(22.373)[GPX7](http://www.ncbi.nlm.nih.gov/gene/?term=2882)(22.373) [CRLF1](http://www.ncbi.nlm.nih.gov/gene/?term=9244)(22.373)[FADD](http://www.ncbi.nlm.nih.gov/gene/?term=8772)(22.373)[SNTG2](http://www.ncbi.nlm.nih.gov/gene/?term=54221)(22.373)[SCGB1A1](http://www.ncbi.nlm.nih.gov/gene/?term=7356)(22.373)[OXTR](http://www.ncbi.nlm.nih.gov/gene/?term=5021)(22.373) [ALDH1B1](http://www.ncbi.nlm.nih.gov/gene/?term=219)(22.373)[LEP](http://www.ncbi.nlm.nih.gov/gene/?term=3952)(22.373)[NLGN1](http://www.ncbi.nlm.nih.gov/gene/?term=22871)(22.373)[CACNA1D](http://www.ncbi.nlm.nih.gov/gene/?term=776)(22.373)[RAPGEF2](http://www.ncbi.nlm.nih.gov/gene/?term=9693)(22.373) [DARS](http://www.ncbi.nlm.nih.gov/gene/?term=1615)(22.373)[ANK3](http://www.ncbi.nlm.nih.gov/gene/?term=288)(22.373)[KCNE5](http://www.ncbi.nlm.nih.gov/gene/?term=23630)(22.373)[PPARD](http://www.ncbi.nlm.nih.gov/gene/?term=5467)(22.373)[NKX2-1](http://www.ncbi.nlm.nih.gov/gene/?term=7080)(22.373) [MIP](http://www.ncbi.nlm.nih.gov/gene/?term=4284)(22.373)[RYR3](http://www.ncbi.nlm.nih.gov/gene/?term=6263)(22.373)[ZPR1](http://www.ncbi.nlm.nih.gov/gene/?term=8882)(22.373)[ALDH3B1](http://www.ncbi.nlm.nih.gov/gene/?term=221)(22.373)[NPPA](http://www.ncbi.nlm.nih.gov/gene/?term=4878)(22.373) [STX1A](http://www.ncbi.nlm.nih.gov/gene/?term=6804)(22.373)[PAXBP1](http://www.ncbi.nlm.nih.gov/gene/?term=94104)(22.373)[PAX7](http://www.ncbi.nlm.nih.gov/gene/?term=5081)(22.373)[ALDH3B2](http://www.ncbi.nlm.nih.gov/gene/?term=222)(22.373)[RNASE2](http://www.ncbi.nlm.nih.gov/gene/?term=6036)(22.373) [NRXN2](http://www.ncbi.nlm.nih.gov/gene/?term=9379)(22.373)[ADORA1](http://www.ncbi.nlm.nih.gov/gene/?term=134)(22.373)[ADH4](http://www.ncbi.nlm.nih.gov/gene/?term=127)(22.373)[GJA5](http://www.ncbi.nlm.nih.gov/gene/?term=2702)(22.373)[FAS](http://www.ncbi.nlm.nih.gov/gene/?term=355)(22.373) [DAB2IP](http://www.ncbi.nlm.nih.gov/gene/?term=153090)(22.373)[CYP2E1](http://www.ncbi.nlm.nih.gov/gene/?term=1571)(22.373)[ACY1](http://www.ncbi.nlm.nih.gov/gene/?term=95)(22.373) | |
| [Neoisoliquiritin](http://www.megabionet.org/tcmid/ingredient/15406/) | This compound doesn't have any potential target with score larger than 20. | |
| [Ononin](http://www.megabionet.org/tcmid/ingredient/24040/) | This compound doesn't have any potential target with score larger than 20. | |
| [Atractylenolide Ii](http://www.megabionet.org/tcmid/ingredient/1966/) | [CHRM3](http://www.ncbi.nlm.nih.gov/gene/?term=1131)(80.882)[CHRM1](http://www.ncbi.nlm.nih.gov/gene/?term=1128)(80.882)[CHRM2](http://www.ncbi.nlm.nih.gov/gene/?term=1129)(80.882)[ESR1](http://www.ncbi.nlm.nih.gov/gene/?term=2099)(48.000)[PGR](http://www.ncbi.nlm.nih.gov/gene/?term=5241)(48.000) [ATP1A1](http://www.ncbi.nlm.nih.gov/gene/?term=476)(48.000)[ARFGEF2](http://www.ncbi.nlm.nih.gov/gene/?term=10564)(22.373)[DOCK5](http://www.ncbi.nlm.nih.gov/gene/?term=80005)(22.373)[NTSR1](http://www.ncbi.nlm.nih.gov/gene/?term=4923)(22.373)[CHRM5](http://www.ncbi.nlm.nih.gov/gene/?term=1133)(22.373) [GNA15](http://www.ncbi.nlm.nih.gov/gene/?term=2769)(22.373)[P2RX1](http://www.ncbi.nlm.nih.gov/gene/?term=5023)(22.373)[DOCK4](http://www.ncbi.nlm.nih.gov/gene/?term=9732)(22.373)[MAP2K1](http://www.ncbi.nlm.nih.gov/gene/?term=5604)(22.373)[CHRM4](http://www.ncbi.nlm.nih.gov/gene/?term=1132)(22.373) | |
| [Isotrilobine](http://www.megabionet.org/tcmid/ingredient/11744/) | [ACHE](http://www.ncbi.nlm.nih.gov/gene/?term=43)(122.778)[CHRNA2](http://www.ncbi.nlm.nih.gov/gene/?term=1135)(122.778)[HTR3A](http://www.ncbi.nlm.nih.gov/gene/?term=3359)(122.778)[CHRM2](http://www.ncbi.nlm.nih.gov/gene/?term=1129)(122.778)[CHRM3](http://www.ncbi.nlm.nih.gov/gene/?term=1131)(48.000) [DRD2](http://www.ncbi.nlm.nih.gov/gene/?term=1813)(48.000)[SLC18A2](http://www.ncbi.nlm.nih.gov/gene/?term=6571)(48.000)[BCHE](http://www.ncbi.nlm.nih.gov/gene/?term=590)(48.000)[CYBA](http://www.ncbi.nlm.nih.gov/gene/?term=1535)(23.000)[CHRNA4](http://www.ncbi.nlm.nih.gov/gene/?term=1137)(23.000) [RAC2](http://www.ncbi.nlm.nih.gov/gene/?term=5880)(23.000)[RAC1](http://www.ncbi.nlm.nih.gov/gene/?term=5879)(23.000)[OPRD1](http://www.ncbi.nlm.nih.gov/gene/?term=4985)(23.000)[NCF4](http://www.ncbi.nlm.nih.gov/gene/?term=4689)(23.000)[CHRNA7](http://www.ncbi.nlm.nih.gov/gene/?term=1139)(23.000) [CYBB](http://www.ncbi.nlm.nih.gov/gene/?term=1536)(23.000)[NCF1](http://www.ncbi.nlm.nih.gov/gene/?term=653361)(23.000)[SIGMAR1](http://www.ncbi.nlm.nih.gov/gene/?term=10280)(23.000)[NCF2](http://www.ncbi.nlm.nih.gov/gene/?term=4688)(23.000)[OPRK1](http://www.ncbi.nlm.nih.gov/gene/?term=4986)(23.000) [CHRNA3](http://www.ncbi.nlm.nih.gov/gene/?term=1136)(23.000)[RAC3](http://www.ncbi.nlm.nih.gov/gene/?term=5881)(23.000)[CHRNB2](http://www.ncbi.nlm.nih.gov/gene/?term=1141)(23.000)[SLC6A4](http://www.ncbi.nlm.nih.gov/gene/?term=6532)(23.000)[OPRM1](http://www.ncbi.nlm.nih.gov/gene/?term=4988)(23.000) [PGRMC1](http://www.ncbi.nlm.nih.gov/gene/?term=10857)(23.000)[GRIN3A](http://www.ncbi.nlm.nih.gov/gene/?term=116443)(23.000)[CHRFAM7A](http://www.ncbi.nlm.nih.gov/gene/?term=89832)(23.000)[CHRNB4](http://www.ncbi.nlm.nih.gov/gene/?term=1143)(23.000)[SLC6A2](http://www.ncbi.nlm.nih.gov/gene/?term=6530)(23.000) [TLR4](http://www.ncbi.nlm.nih.gov/gene/?term=7099)(22.373)[ELANE](http://www.ncbi.nlm.nih.gov/gene/?term=1991)(22.373)[CTSG](http://www.ncbi.nlm.nih.gov/gene/?term=1511)(22.373)[SLC18A1](http://www.ncbi.nlm.nih.gov/gene/?term=6570)(22.373)[CEND1](http://www.ncbi.nlm.nih.gov/gene/?term=51286)(22.373) [SLC22A1](http://www.ncbi.nlm.nih.gov/gene/?term=6580)(22.373)[ADRA1B](http://www.ncbi.nlm.nih.gov/gene/?term=147)(22.373)[WLS](http://www.ncbi.nlm.nih.gov/gene/?term=79971)(22.373)[CHRNA9](http://www.ncbi.nlm.nih.gov/gene/?term=55584)(22.373) | |
| [2,5-Dihydroxymethyl-3,4-Dihydroxypyrrolidine](http://www.megabionet.org/tcmid/ingredient/6027/) | [GAA](http://www.ncbi.nlm.nih.gov/gene/?term=2548)(48.000)[MGAM](http://www.ncbi.nlm.nih.gov/gene/?term=8972)(48.000)[UGCG](http://www.ncbi.nlm.nih.gov/gene/?term=7357)(48.000)[GANAB](http://www.ncbi.nlm.nih.gov/gene/?term=23193)(48.000)[GANC](http://www.ncbi.nlm.nih.gov/gene/?term=2595)(48.000) | |
| [Licoricesaponine J2](http://www.megabionet.org/tcmid/ingredient/31438/) | [NR3C1](http://www.ncbi.nlm.nih.gov/gene/?term=2908)(48.000) | |
| [Heneicosane](http://www.megabionet.org/tcmid/ingredient/9358/) | This compound doesn't have any potential target with score larger than 20. | |
| [2,4,4-Trimethyl-1-Pentene](http://www.megabionet.org/tcmid/ingredient/25077/) | [KCND1](http://www.ncbi.nlm.nih.gov/gene/?term=3750)(80.882)[KCNA3](http://www.ncbi.nlm.nih.gov/gene/?term=3738)(80.882)[PRKAB1](http://www.ncbi.nlm.nih.gov/gene/?term=5564)(80.882)[ADH1A](http://www.ncbi.nlm.nih.gov/gene/?term=124)(80.882)[KCNA10](http://www.ncbi.nlm.nih.gov/gene/?term=3744)(80.882) [GAMT](http://www.ncbi.nlm.nih.gov/gene/?term=2593)(80.882)[KCNC3](http://www.ncbi.nlm.nih.gov/gene/?term=3748)(80.882)[KCNA1](http://www.ncbi.nlm.nih.gov/gene/?term=3736)(80.882)[KCNA2](http://www.ncbi.nlm.nih.gov/gene/?term=3737)(80.882)[TPO](http://www.ncbi.nlm.nih.gov/gene/?term=7173)(80.882) [CAT](http://www.ncbi.nlm.nih.gov/gene/?term=847)(80.882)[KCNB1](http://www.ncbi.nlm.nih.gov/gene/?term=3745)(80.882)[ADH1B](http://www.ncbi.nlm.nih.gov/gene/?term=125)(80.882)[DLG4](http://www.ncbi.nlm.nih.gov/gene/?term=1742)(80.882)[KCNC2](http://www.ncbi.nlm.nih.gov/gene/?term=3747)(80.882) [KCNC1](http://www.ncbi.nlm.nih.gov/gene/?term=3746)(80.882)[ADH1C](http://www.ncbi.nlm.nih.gov/gene/?term=126)(80.882)[KCNB2](http://www.ncbi.nlm.nih.gov/gene/?term=9312)(80.882)[KCNA5](http://www.ncbi.nlm.nih.gov/gene/?term=3741)(80.882)[RNASE1](http://www.ncbi.nlm.nih.gov/gene/?term=6035)(80.882) [KCND2](http://www.ncbi.nlm.nih.gov/gene/?term=3751)(80.882)[ALDH2](http://www.ncbi.nlm.nih.gov/gene/?term=217)(80.882)[KCNA4](http://www.ncbi.nlm.nih.gov/gene/?term=3739)(80.882)[KCNA7](http://www.ncbi.nlm.nih.gov/gene/?term=3743)(80.882)[KCNA6](http://www.ncbi.nlm.nih.gov/gene/?term=3742)(80.882) [KCND3](http://www.ncbi.nlm.nih.gov/gene/?term=3752)(80.882)[GUCY1B3](http://www.ncbi.nlm.nih.gov/gene/?term=2983)(55.444)[GATM](http://www.ncbi.nlm.nih.gov/gene/?term=2628)(55.444)[KCNK4](http://www.ncbi.nlm.nih.gov/gene/?term=50801)(55.444)[IYD](http://www.ncbi.nlm.nih.gov/gene/?term=389434)(55.444) [KCNQ1](http://www.ncbi.nlm.nih.gov/gene/?term=3784)(55.444)[FBP1](http://www.ncbi.nlm.nih.gov/gene/?term=2203)(22.373)[DGKI](http://www.ncbi.nlm.nih.gov/gene/?term=9162)(22.373)[ADH7](http://www.ncbi.nlm.nih.gov/gene/?term=131)(22.373)[NFIB](http://www.ncbi.nlm.nih.gov/gene/?term=4781)(22.373) [CYGB](http://www.ncbi.nlm.nih.gov/gene/?term=114757)(22.373)[NRXN3](http://www.ncbi.nlm.nih.gov/gene/?term=9369)(22.373)[LRRC4B](http://www.ncbi.nlm.nih.gov/gene/?term=94030)(22.373)[AQP8](http://www.ncbi.nlm.nih.gov/gene/?term=343)(22.373)[ARX](http://www.ncbi.nlm.nih.gov/gene/?term=170302)(22.373) [IGF1](http://www.ncbi.nlm.nih.gov/gene/?term=3479)(22.373)[SHANK3](http://www.ncbi.nlm.nih.gov/gene/?term=85358)(22.373)[ACY3](http://www.ncbi.nlm.nih.gov/gene/?term=91703)(22.373)[ASPA](http://www.ncbi.nlm.nih.gov/gene/?term=443)(22.373)[NRXN1](http://www.ncbi.nlm.nih.gov/gene/?term=9378)(22.373) [EDN1](http://www.ncbi.nlm.nih.gov/gene/?term=1906)(22.373)[OXT](http://www.ncbi.nlm.nih.gov/gene/?term=5020)(22.373)[MAGI2](http://www.ncbi.nlm.nih.gov/gene/?term=9863)(22.373)[APOE](http://www.ncbi.nlm.nih.gov/gene/?term=348)(22.373)[SOX15](http://www.ncbi.nlm.nih.gov/gene/?term=6665)(22.373) [CNTNAP4](http://www.ncbi.nlm.nih.gov/gene/?term=85445)(22.373)[RNASE4](http://www.ncbi.nlm.nih.gov/gene/?term=6038)(22.373)[RAB3A](http://www.ncbi.nlm.nih.gov/gene/?term=5864)(22.373)[MC4R](http://www.ncbi.nlm.nih.gov/gene/?term=4160)(22.373)[IL1B](http://www.ncbi.nlm.nih.gov/gene/?term=3553)(22.373) [GRIN2A](http://www.ncbi.nlm.nih.gov/gene/?term=2903)(22.373)[SLC17A7](http://www.ncbi.nlm.nih.gov/gene/?term=57030)(22.373)[CYP11A1](http://www.ncbi.nlm.nih.gov/gene/?term=1583)(22.373)[SCN5A](http://www.ncbi.nlm.nih.gov/gene/?term=6331)(22.373)[SCN10A](http://www.ncbi.nlm.nih.gov/gene/?term=6336)(22.373) [SPARC](http://www.ncbi.nlm.nih.gov/gene/?term=6678)(22.373)[SORCS3](http://www.ncbi.nlm.nih.gov/gene/?term=22986)(22.373)[RNASE8](http://www.ncbi.nlm.nih.gov/gene/?term=122665)(22.373)[KCNIP2](http://www.ncbi.nlm.nih.gov/gene/?term=30819)(22.373)[GPX7](http://www.ncbi.nlm.nih.gov/gene/?term=2882)(22.373) [CRLF1](http://www.ncbi.nlm.nih.gov/gene/?term=9244)(22.373)[FADD](http://www.ncbi.nlm.nih.gov/gene/?term=8772)(22.373)[SNTG2](http://www.ncbi.nlm.nih.gov/gene/?term=54221)(22.373)[SCGB1A1](http://www.ncbi.nlm.nih.gov/gene/?term=7356)(22.373)[OXTR](http://www.ncbi.nlm.nih.gov/gene/?term=5021)(22.373) [ALDH1B1](http://www.ncbi.nlm.nih.gov/gene/?term=219)(22.373)[LEP](http://www.ncbi.nlm.nih.gov/gene/?term=3952)(22.373)[NLGN1](http://www.ncbi.nlm.nih.gov/gene/?term=22871)(22.373)[CACNA1D](http://www.ncbi.nlm.nih.gov/gene/?term=776)(22.373)[RAPGEF2](http://www.ncbi.nlm.nih.gov/gene/?term=9693)(22.373) [DARS](http://www.ncbi.nlm.nih.gov/gene/?term=1615)(22.373)[ANK3](http://www.ncbi.nlm.nih.gov/gene/?term=288)(22.373)[KCNE5](http://www.ncbi.nlm.nih.gov/gene/?term=23630)(22.373)[PPARD](http://www.ncbi.nlm.nih.gov/gene/?term=5467)(22.373)[NKX2-1](http://www.ncbi.nlm.nih.gov/gene/?term=7080)(22.373) [MIP](http://www.ncbi.nlm.nih.gov/gene/?term=4284)(22.373)[RYR3](http://www.ncbi.nlm.nih.gov/gene/?term=6263)(22.373)[ZPR1](http://www.ncbi.nlm.nih.gov/gene/?term=8882)(22.373)[ALDH3B1](http://www.ncbi.nlm.nih.gov/gene/?term=221)(22.373)[NPPA](http://www.ncbi.nlm.nih.gov/gene/?term=4878)(22.373) [STX1A](http://www.ncbi.nlm.nih.gov/gene/?term=6804)(22.373)[PAXBP1](http://www.ncbi.nlm.nih.gov/gene/?term=94104)(22.373)[PAX7](http://www.ncbi.nlm.nih.gov/gene/?term=5081)(22.373)[ALDH3B2](http://www.ncbi.nlm.nih.gov/gene/?term=222)(22.373)[RNASE2](http://www.ncbi.nlm.nih.gov/gene/?term=6036)(22.373) [NRXN2](http://www.ncbi.nlm.nih.gov/gene/?term=9379)(22.373)[ADORA1](http://www.ncbi.nlm.nih.gov/gene/?term=134)(22.373)[ADH4](http://www.ncbi.nlm.nih.gov/gene/?term=127)(22.373)[GJA5](http://www.ncbi.nlm.nih.gov/gene/?term=2702)(22.373)[FAS](http://www.ncbi.nlm.nih.gov/gene/?term=355)(22.373) [DAB2IP](http://www.ncbi.nlm.nih.gov/gene/?term=153090)(22.373)[CYP2E1](http://www.ncbi.nlm.nih.gov/gene/?term=1571)(22.373)[ACY1](http://www.ncbi.nlm.nih.gov/gene/?term=95)(22.373) | |
| [Alpha-Spinasterol-Beta-D-Glucoside](http://www.megabionet.org/tcmid/ingredient/23720/) | [ATP1A1](http://www.ncbi.nlm.nih.gov/gene/?term=476)(48.000) | |
| [Gloeosteretriol](http://www.megabionet.org/tcmid/ingredient/8580/) | [TRPV3](http://www.ncbi.nlm.nih.gov/gene/?term=162514)(48.000)[OPRK1](http://www.ncbi.nlm.nih.gov/gene/?term=4986)(48.000)[TRPM8](http://www.ncbi.nlm.nih.gov/gene/?term=79054)(48.000)[TRPA1](http://www.ncbi.nlm.nih.gov/gene/?term=8989)(48.000) | |
| [Narwedine](http://www.megabionet.org/tcmid/ingredient/15280/) | [CHRNG](http://www.ncbi.nlm.nih.gov/gene/?term=1146)(122.778)[CHRNA4](http://www.ncbi.nlm.nih.gov/gene/?term=1137)(122.778)[CHRNB3](http://www.ncbi.nlm.nih.gov/gene/?term=1142)(122.778)[CHRNA7](http://www.ncbi.nlm.nih.gov/gene/?term=1139)(122.778)[CHRNA1](http://www.ncbi.nlm.nih.gov/gene/?term=1134)(122.778) [CHRNA10](http://www.ncbi.nlm.nih.gov/gene/?term=57053)(122.778)[CHRNA3](http://www.ncbi.nlm.nih.gov/gene/?term=1136)(122.778)[CHRNB2](http://www.ncbi.nlm.nih.gov/gene/?term=1141)(122.778)[ACHE](http://www.ncbi.nlm.nih.gov/gene/?term=43)(122.778)[CHRNE](http://www.ncbi.nlm.nih.gov/gene/?term=1145)(122.778) [CHRNA2](http://www.ncbi.nlm.nih.gov/gene/?term=1135)(122.778)[CHRNA6](http://www.ncbi.nlm.nih.gov/gene/?term=8973)(122.778)[CHRND](http://www.ncbi.nlm.nih.gov/gene/?term=1144)(122.778)[BCHE](http://www.ncbi.nlm.nih.gov/gene/?term=590)(122.778)[CHRNA9](http://www.ncbi.nlm.nih.gov/gene/?term=55584)(122.778) [CHRFAM7A](http://www.ncbi.nlm.nih.gov/gene/?term=89832)(122.778)[CHRNA5](http://www.ncbi.nlm.nih.gov/gene/?term=1138)(122.778)[CHRNB4](http://www.ncbi.nlm.nih.gov/gene/?term=1143)(122.778)[CHRNB1](http://www.ncbi.nlm.nih.gov/gene/?term=1140)(122.778)[OPRD1](http://www.ncbi.nlm.nih.gov/gene/?term=4985)(48.000) [OPRK1](http://www.ncbi.nlm.nih.gov/gene/?term=4986)(48.000)[OPRM1](http://www.ncbi.nlm.nih.gov/gene/?term=4988)(48.000)[CACNB1](http://www.ncbi.nlm.nih.gov/gene/?term=782)(23.000)[PDE5A](http://www.ncbi.nlm.nih.gov/gene/?term=8654)(23.000)[ADRA1A](http://www.ncbi.nlm.nih.gov/gene/?term=148)(23.000) [SCN1A](http://www.ncbi.nlm.nih.gov/gene/?term=6323)(23.000)[PDE6G](http://www.ncbi.nlm.nih.gov/gene/?term=5148)(23.000)[CACNA1F](http://www.ncbi.nlm.nih.gov/gene/?term=778)(23.000)[HTR2A](http://www.ncbi.nlm.nih.gov/gene/?term=3356)(23.000)[ADRA2A](http://www.ncbi.nlm.nih.gov/gene/?term=150)(23.000) [KCNK2](http://www.ncbi.nlm.nih.gov/gene/?term=3776)(23.000)[SCN5A](http://www.ncbi.nlm.nih.gov/gene/?term=6331)(23.000)[SCN10A](http://www.ncbi.nlm.nih.gov/gene/?term=6336)(23.000)[CACNA1S](http://www.ncbi.nlm.nih.gov/gene/?term=779)(23.000)[ADRB1](http://www.ncbi.nlm.nih.gov/gene/?term=153)(23.000) [CACNB4](http://www.ncbi.nlm.nih.gov/gene/?term=785)(23.000)[ADRA1D](http://www.ncbi.nlm.nih.gov/gene/?term=146)(23.000)[DRD2](http://www.ncbi.nlm.nih.gov/gene/?term=1813)(23.000)[CACNA1C](http://www.ncbi.nlm.nih.gov/gene/?term=775)(23.000)[CACNA1D](http://www.ncbi.nlm.nih.gov/gene/?term=776)(23.000) [CACNB3](http://www.ncbi.nlm.nih.gov/gene/?term=784)(23.000)[KCNH2](http://www.ncbi.nlm.nih.gov/gene/?term=3757)(23.000)[SLC18A2](http://www.ncbi.nlm.nih.gov/gene/?term=6571)(23.000)[ADRA1B](http://www.ncbi.nlm.nih.gov/gene/?term=147)(23.000)[ADRA2C](http://www.ncbi.nlm.nih.gov/gene/?term=152)(23.000) [CACNB2](http://www.ncbi.nlm.nih.gov/gene/?term=783)(23.000)[PDE6H](http://www.ncbi.nlm.nih.gov/gene/?term=5149)(23.000)[ADRA2B](http://www.ncbi.nlm.nih.gov/gene/?term=151)(23.000)[SLC17A7](http://www.ncbi.nlm.nih.gov/gene/?term=57030)(22.373)[HTR2C](http://www.ncbi.nlm.nih.gov/gene/?term=3358)(22.373) [COLQ](http://www.ncbi.nlm.nih.gov/gene/?term=8292)(22.373)[CNR1](http://www.ncbi.nlm.nih.gov/gene/?term=1268)(22.373)[SCN1B](http://www.ncbi.nlm.nih.gov/gene/?term=6324)(22.373)[WLS](http://www.ncbi.nlm.nih.gov/gene/?term=79971)(22.373)[CD34](http://www.ncbi.nlm.nih.gov/gene/?term=947)(22.373) | |
| [Glycine](http://www.megabionet.org/tcmid/ingredient/8816/) | This compound doesn't have any potential target with score larger than 20. | |
| [6,6'-Dimethoxygossypol](http://www.megabionet.org/tcmid/ingredient/6231/) | [TYR](http://www.ncbi.nlm.nih.gov/gene/?term=7299)(80.882)[DCT](http://www.ncbi.nlm.nih.gov/gene/?term=1638)(55.444)[TYRP1](http://www.ncbi.nlm.nih.gov/gene/?term=7306)(55.444)[SEC14L3](http://www.ncbi.nlm.nih.gov/gene/?term=266629)(23.000)[PPP2CA](http://www.ncbi.nlm.nih.gov/gene/?term=5515)(23.000) [PRKCA](http://www.ncbi.nlm.nih.gov/gene/?term=5578)(23.000)[NR1I2](http://www.ncbi.nlm.nih.gov/gene/?term=8856)(23.000)[ALOX5](http://www.ncbi.nlm.nih.gov/gene/?term=240)(23.000)[PPP2CB](http://www.ncbi.nlm.nih.gov/gene/?term=5516)(23.000)[SEC14L2](http://www.ncbi.nlm.nih.gov/gene/?term=23541)(23.000) [DGKA](http://www.ncbi.nlm.nih.gov/gene/?term=1606)(23.000)[PRKCB](http://www.ncbi.nlm.nih.gov/gene/?term=5579)(23.000)[SEC14L4](http://www.ncbi.nlm.nih.gov/gene/?term=284904)(23.000)[AP3D1](http://www.ncbi.nlm.nih.gov/gene/?term=8943)(22.373)[OCA2](http://www.ncbi.nlm.nih.gov/gene/?term=4948)(22.373) [GPR143](http://www.ncbi.nlm.nih.gov/gene/?term=4935)(22.373) | |
| [12-O-Nicotinoylisolineolone](http://www.megabionet.org/tcmid/ingredient/15520/) | [PTGER4](http://www.ncbi.nlm.nih.gov/gene/?term=5734)(23.000)[PRKCA](http://www.ncbi.nlm.nih.gov/gene/?term=5578)(23.000)[PTGER2](http://www.ncbi.nlm.nih.gov/gene/?term=5732)(23.000)[PTGER3](http://www.ncbi.nlm.nih.gov/gene/?term=5733)(23.000)[PRKCD](http://www.ncbi.nlm.nih.gov/gene/?term=5580)(23.000) | |
| [Asperulosidic Acid](http://www.megabionet.org/tcmid/ingredient/30635/) | This compound doesn't have any potential target with score larger than 20. | |
| [Tetrahydroharmine](http://www.megabionet.org/tcmid/ingredient/21054/) | [SLC6A3](http://www.ncbi.nlm.nih.gov/gene/?term=6531)(80.882)[SLC6A4](http://www.ncbi.nlm.nih.gov/gene/?term=6532)(80.882)[SLC6A2](http://www.ncbi.nlm.nih.gov/gene/?term=6530)(80.882)[SLC18A1](http://www.ncbi.nlm.nih.gov/gene/?term=6570)(55.444)[CEND1](http://www.ncbi.nlm.nih.gov/gene/?term=51286)(55.444) [SLC22A1](http://www.ncbi.nlm.nih.gov/gene/?term=6580)(55.444)[KCNJ8](http://www.ncbi.nlm.nih.gov/gene/?term=3764)(48.000)[KCNJ12](http://www.ncbi.nlm.nih.gov/gene/?term=3768)(48.000)[HTR2A](http://www.ncbi.nlm.nih.gov/gene/?term=3356)(48.000)[HTR2B](http://www.ncbi.nlm.nih.gov/gene/?term=3357)(48.000) [ADRA2A](http://www.ncbi.nlm.nih.gov/gene/?term=150)(48.000)[HTR1B](http://www.ncbi.nlm.nih.gov/gene/?term=3351)(48.000)[KCNJ11](http://www.ncbi.nlm.nih.gov/gene/?term=3767)(48.000)[KCNJ15](http://www.ncbi.nlm.nih.gov/gene/?term=3772)(48.000)[HTR2C](http://www.ncbi.nlm.nih.gov/gene/?term=3358)(48.000) [HTR1D](http://www.ncbi.nlm.nih.gov/gene/?term=3352)(48.000)[DRD2](http://www.ncbi.nlm.nih.gov/gene/?term=1813)(48.000)[KCNJ10](http://www.ncbi.nlm.nih.gov/gene/?term=3766)(48.000)[HTR1A](http://www.ncbi.nlm.nih.gov/gene/?term=3350)(48.000)[ADRA2C](http://www.ncbi.nlm.nih.gov/gene/?term=152)(48.000) [DRD3](http://www.ncbi.nlm.nih.gov/gene/?term=1814)(48.000)[KCNJ1](http://www.ncbi.nlm.nih.gov/gene/?term=3758)(48.000)[ADRA2B](http://www.ncbi.nlm.nih.gov/gene/?term=151)(48.000)[KCNJ14](http://www.ncbi.nlm.nih.gov/gene/?term=3770)(48.000)[GRIN3B](http://www.ncbi.nlm.nih.gov/gene/?term=116444)(23.000) [GRIN2A](http://www.ncbi.nlm.nih.gov/gene/?term=2903)(23.000)[GRIN2C](http://www.ncbi.nlm.nih.gov/gene/?term=2905)(23.000)[GRIN2B](http://www.ncbi.nlm.nih.gov/gene/?term=2904)(23.000)[GRIN3A](http://www.ncbi.nlm.nih.gov/gene/?term=116443)(23.000)[GRIN2D](http://www.ncbi.nlm.nih.gov/gene/?term=2906)(23.000) [GRIN1](http://www.ncbi.nlm.nih.gov/gene/?term=2902)(23.000)[GPM6B](http://www.ncbi.nlm.nih.gov/gene/?term=2824)(22.373)[PDE3A](http://www.ncbi.nlm.nih.gov/gene/?term=5139)(22.373)[AVPR1A](http://www.ncbi.nlm.nih.gov/gene/?term=552)(22.373)[HCN4](http://www.ncbi.nlm.nih.gov/gene/?term=10021)(22.373) [OXT](http://www.ncbi.nlm.nih.gov/gene/?term=5020)(22.373)[SLC22A2](http://www.ncbi.nlm.nih.gov/gene/?term=6582)(22.373)[COMT](http://www.ncbi.nlm.nih.gov/gene/?term=1312)(22.373)[MAOB](http://www.ncbi.nlm.nih.gov/gene/?term=4129)(22.373)[OXTR](http://www.ncbi.nlm.nih.gov/gene/?term=5021)(22.373) [MAOA](http://www.ncbi.nlm.nih.gov/gene/?term=4128)(22.373)[ACVR2A](http://www.ncbi.nlm.nih.gov/gene/?term=92)(22.373)[RAPGEF2](http://www.ncbi.nlm.nih.gov/gene/?term=9693)(22.373)[LRTOMT](http://www.ncbi.nlm.nih.gov/gene/?term=220074)(22.373)[DBH](http://www.ncbi.nlm.nih.gov/gene/?term=1621)(22.373) [PTGS2](http://www.ncbi.nlm.nih.gov/gene/?term=5743)(22.373)[SNCA](http://www.ncbi.nlm.nih.gov/gene/?term=6622)(22.373)[PDE2A](http://www.ncbi.nlm.nih.gov/gene/?term=5138)(22.373)[NOS1](http://www.ncbi.nlm.nih.gov/gene/?term=4842)(22.373)[SLC22A3](http://www.ncbi.nlm.nih.gov/gene/?term=6581)(22.373) [HCN2](http://www.ncbi.nlm.nih.gov/gene/?term=610)(22.373) | |
| [Glycyrrhiza-Flavonol A](http://www.megabionet.org/tcmid/ingredient/8844/) | This compound doesn't have any potential target with score larger than 20. | |
| [Nonadecanoic Acid](http://www.megabionet.org/tcmid/ingredient/24770/) | [AKR1D1](http://www.ncbi.nlm.nih.gov/gene/?term=6718)(686.000)[TYR](http://www.ncbi.nlm.nih.gov/gene/?term=7299)(686.000)[SRD5A2](http://www.ncbi.nlm.nih.gov/gene/?term=6716)(686.000)[SCN11A](http://www.ncbi.nlm.nih.gov/gene/?term=11280)(122.778)[SCN2B](http://www.ncbi.nlm.nih.gov/gene/?term=6327)(122.778) [ABAT](http://www.ncbi.nlm.nih.gov/gene/?term=18)(122.778)[SCN1A](http://www.ncbi.nlm.nih.gov/gene/?term=6323)(122.778)[ESRRG](http://www.ncbi.nlm.nih.gov/gene/?term=2104)(122.778)[SCN3B](http://www.ncbi.nlm.nih.gov/gene/?term=55800)(122.778)[COX6C](http://www.ncbi.nlm.nih.gov/gene/?term=1345)(122.778) [COX5B](http://www.ncbi.nlm.nih.gov/gene/?term=1329)(122.778)[COX7C](http://www.ncbi.nlm.nih.gov/gene/?term=1350)(122.778)[COX1](http://www.ncbi.nlm.nih.gov/gene/?term=4512)(122.778)[SCN3A](http://www.ncbi.nlm.nih.gov/gene/?term=6328)(122.778)[AKR1C2](http://www.ncbi.nlm.nih.gov/gene/?term=1646)(122.778) [SCN7A](http://www.ncbi.nlm.nih.gov/gene/?term=6332)(122.778)[SCN5A](http://www.ncbi.nlm.nih.gov/gene/?term=6331)(122.778)[SCN10A](http://www.ncbi.nlm.nih.gov/gene/?term=6336)(122.778)[ALDH5A1](http://www.ncbi.nlm.nih.gov/gene/?term=7915)(122.778)[SCN2A](http://www.ncbi.nlm.nih.gov/gene/?term=6326)(122.778) [COX5A](http://www.ncbi.nlm.nih.gov/gene/?term=9377)(122.778)[COX3](http://www.ncbi.nlm.nih.gov/gene/?term=4514)(122.778)[AR](http://www.ncbi.nlm.nih.gov/gene/?term=367)(122.778)[COX7A1](http://www.ncbi.nlm.nih.gov/gene/?term=1346)(122.778)[FECH](http://www.ncbi.nlm.nih.gov/gene/?term=2235)(122.778) [COX4I1](http://www.ncbi.nlm.nih.gov/gene/?term=1327)(122.778)[HDAC9](http://www.ncbi.nlm.nih.gov/gene/?term=9734)(122.778)[PLA2G1B](http://www.ncbi.nlm.nih.gov/gene/?term=5319)(122.778)[COX6A2](http://www.ncbi.nlm.nih.gov/gene/?term=1339)(122.778)[SCN9A](http://www.ncbi.nlm.nih.gov/gene/?term=6335)(122.778) [SCN4A](http://www.ncbi.nlm.nih.gov/gene/?term=6329)(122.778)[SCN4B](http://www.ncbi.nlm.nih.gov/gene/?term=6330)(122.778)[ADH1C](http://www.ncbi.nlm.nih.gov/gene/?term=126)(122.778)[COX6B1](http://www.ncbi.nlm.nih.gov/gene/?term=1340)(122.778)[FABP6](http://www.ncbi.nlm.nih.gov/gene/?term=2172)(122.778) [CES1](http://www.ncbi.nlm.nih.gov/gene/?term=1066)(122.778)[ACADSB](http://www.ncbi.nlm.nih.gov/gene/?term=36)(122.778)[SCN8A](http://www.ncbi.nlm.nih.gov/gene/?term=6334)(122.778)[COX7B](http://www.ncbi.nlm.nih.gov/gene/?term=1349)(122.778)[SCN1B](http://www.ncbi.nlm.nih.gov/gene/?term=6324)(122.778) [COX2](http://www.ncbi.nlm.nih.gov/gene/?term=4513)(122.778)[OGDH](http://www.ncbi.nlm.nih.gov/gene/?term=4967)(122.778)[HDAC2](http://www.ncbi.nlm.nih.gov/gene/?term=3066)(122.778)[COX8A](http://www.ncbi.nlm.nih.gov/gene/?term=1351)(122.778)[NR1H4](http://www.ncbi.nlm.nih.gov/gene/?term=9971)(122.778) [SUCLG2](http://www.ncbi.nlm.nih.gov/gene/?term=8801)(80.882)[SLC13A2](http://www.ncbi.nlm.nih.gov/gene/?term=9058)(80.882)[SLC13A1](http://www.ncbi.nlm.nih.gov/gene/?term=6561)(80.882)[SUCLG1](http://www.ncbi.nlm.nih.gov/gene/?term=8802)(80.882)[HSD17B6](http://www.ncbi.nlm.nih.gov/gene/?term=8630)(80.882) [SLC25A10](http://www.ncbi.nlm.nih.gov/gene/?term=1468)(80.882)[P3H3](http://www.ncbi.nlm.nih.gov/gene/?term=10536)(80.882)[OXCT2](http://www.ncbi.nlm.nih.gov/gene/?term=64064)(80.882)[TMLHE](http://www.ncbi.nlm.nih.gov/gene/?term=55217)(80.882)[PLOD1](http://www.ncbi.nlm.nih.gov/gene/?term=5351)(80.882) [SUCNR1](http://www.ncbi.nlm.nih.gov/gene/?term=56670)(80.882)[SLC13A3](http://www.ncbi.nlm.nih.gov/gene/?term=64849)(80.882)[ASPH](http://www.ncbi.nlm.nih.gov/gene/?term=444)(80.882)[BBOX1](http://www.ncbi.nlm.nih.gov/gene/?term=8424)(80.882)[SDHB](http://www.ncbi.nlm.nih.gov/gene/?term=6390)(80.882) [OXCT1](http://www.ncbi.nlm.nih.gov/gene/?term=5019)(80.882)[PLOD3](http://www.ncbi.nlm.nih.gov/gene/?term=8985)(80.882)[P4HA1](http://www.ncbi.nlm.nih.gov/gene/?term=5033)(80.882)[SDHC](http://www.ncbi.nlm.nih.gov/gene/?term=6391)(80.882)[P3H2](http://www.ncbi.nlm.nih.gov/gene/?term=55214)(80.882) [SDHA](http://www.ncbi.nlm.nih.gov/gene/?term=6389)(80.882)[P4HA2](http://www.ncbi.nlm.nih.gov/gene/?term=8974)(80.882)[SUCLA2](http://www.ncbi.nlm.nih.gov/gene/?term=8803)(80.882)[SDHD](http://www.ncbi.nlm.nih.gov/gene/?term=6392)(80.882)[P3H1](http://www.ncbi.nlm.nih.gov/gene/?term=64175)(80.882) [DCT](http://www.ncbi.nlm.nih.gov/gene/?term=1638)(55.444)[AKR1C3](http://www.ncbi.nlm.nih.gov/gene/?term=8644)(55.444)[CRTAP](http://www.ncbi.nlm.nih.gov/gene/?term=10491)(55.444)[SLIT2](http://www.ncbi.nlm.nih.gov/gene/?term=9353)(55.444)[ACO2](http://www.ncbi.nlm.nih.gov/gene/?term=50)(55.444) [JMJD6](http://www.ncbi.nlm.nih.gov/gene/?term=23210)(55.444)[SLC13A4](http://www.ncbi.nlm.nih.gov/gene/?term=26266)(55.444)[SDHAF2](http://www.ncbi.nlm.nih.gov/gene/?term=54949)(55.444)[SLC1A3](http://www.ncbi.nlm.nih.gov/gene/?term=6507)(55.444)[SALL1](http://www.ncbi.nlm.nih.gov/gene/?term=6299)(55.444) [HSD17B11](http://www.ncbi.nlm.nih.gov/gene/?term=51170)(55.444)[TYRP1](http://www.ncbi.nlm.nih.gov/gene/?term=7306)(55.444)[HIF1AN](http://www.ncbi.nlm.nih.gov/gene/?term=55662)(55.444)[UROS](http://www.ncbi.nlm.nih.gov/gene/?term=7390)(55.444)[SLC13A5](http://www.ncbi.nlm.nih.gov/gene/?term=284111)(55.444) [P4HB](http://www.ncbi.nlm.nih.gov/gene/?term=5034)(55.444)[CACNA2D1](http://www.ncbi.nlm.nih.gov/gene/?term=781)(48.000)[PLAT](http://www.ncbi.nlm.nih.gov/gene/?term=5327)(48.000)[SLC7A2](http://www.ncbi.nlm.nih.gov/gene/?term=6542)(48.000)[CACNA1A](http://www.ncbi.nlm.nih.gov/gene/?term=773)(48.000) [GRIN3B](http://www.ncbi.nlm.nih.gov/gene/?term=116444)(48.000)[CACNA2D2](http://www.ncbi.nlm.nih.gov/gene/?term=9254)(48.000)[GRIN2A](http://www.ncbi.nlm.nih.gov/gene/?term=2903)(48.000)[TOP1](http://www.ncbi.nlm.nih.gov/gene/?term=7150)(48.000)[PLG](http://www.ncbi.nlm.nih.gov/gene/?term=5340)(48.000) [CACNA1B](http://www.ncbi.nlm.nih.gov/gene/?term=774)(48.000)[GRIN2C](http://www.ncbi.nlm.nih.gov/gene/?term=2905)(48.000)[GRIN2B](http://www.ncbi.nlm.nih.gov/gene/?term=2904)(48.000)[SLC7A3](http://www.ncbi.nlm.nih.gov/gene/?term=84889)(48.000)[KARS](http://www.ncbi.nlm.nih.gov/gene/?term=3735)(48.000) [GRIN3A](http://www.ncbi.nlm.nih.gov/gene/?term=116443)(48.000)[SLC7A1](http://www.ncbi.nlm.nih.gov/gene/?term=6541)(48.000)[SLC7A4](http://www.ncbi.nlm.nih.gov/gene/?term=6545)(48.000)[GRIN2D](http://www.ncbi.nlm.nih.gov/gene/?term=2906)(48.000)[ADORA1](http://www.ncbi.nlm.nih.gov/gene/?term=134)(48.000) [GRIN1](http://www.ncbi.nlm.nih.gov/gene/?term=2902)(48.000)[HDAC1](http://www.ncbi.nlm.nih.gov/gene/?term=3065)(23.000)[GPD1L](http://www.ncbi.nlm.nih.gov/gene/?term=23171)(22.373)[SRD5A1](http://www.ncbi.nlm.nih.gov/gene/?term=6715)(22.373)[FBXO45](http://www.ncbi.nlm.nih.gov/gene/?term=200933)(22.373) [SIRT1](http://www.ncbi.nlm.nih.gov/gene/?term=23411)(22.373)[SHMT1](http://www.ncbi.nlm.nih.gov/gene/?term=6470)(22.373)[SUGCT](http://www.ncbi.nlm.nih.gov/gene/?term=79783)(22.373)[ACSS2](http://www.ncbi.nlm.nih.gov/gene/?term=55902)(22.373)[ARV1](http://www.ncbi.nlm.nih.gov/gene/?term=64801)(22.373) [SIX1](http://www.ncbi.nlm.nih.gov/gene/?term=6495)(22.373)[BDH1](http://www.ncbi.nlm.nih.gov/gene/?term=622)(22.373)[COLGALT2](http://www.ncbi.nlm.nih.gov/gene/?term=23127)(22.373)[SIX4](http://www.ncbi.nlm.nih.gov/gene/?term=51804)(22.373)[MRPS36](http://www.ncbi.nlm.nih.gov/gene/?term=92259)(22.373) [OSBPL8](http://www.ncbi.nlm.nih.gov/gene/?term=114882)(22.373)[SPI1](http://www.ncbi.nlm.nih.gov/gene/?term=6688)(22.373)[SUOX](http://www.ncbi.nlm.nih.gov/gene/?term=6821)(22.373)[CCL2](http://www.ncbi.nlm.nih.gov/gene/?term=6347)(22.373)[BCKDK](http://www.ncbi.nlm.nih.gov/gene/?term=10295)(22.373) [UBE2B](http://www.ncbi.nlm.nih.gov/gene/?term=7320)(22.373)[CYP1A2](http://www.ncbi.nlm.nih.gov/gene/?term=1544)(22.373)[CCL5](http://www.ncbi.nlm.nih.gov/gene/?term=6352)(22.373)[ANK2](http://www.ncbi.nlm.nih.gov/gene/?term=287)(22.373)[HSD17B2](http://www.ncbi.nlm.nih.gov/gene/?term=3294)(22.373) [STIM2](http://www.ncbi.nlm.nih.gov/gene/?term=57620)(22.373)[PKD2](http://www.ncbi.nlm.nih.gov/gene/?term=5311)(22.373)[ALDH9A1](http://www.ncbi.nlm.nih.gov/gene/?term=223)(22.373)[TH](http://www.ncbi.nlm.nih.gov/gene/?term=7054)(22.373)[EPO](http://www.ncbi.nlm.nih.gov/gene/?term=2056)(22.373) [COLGALT1](http://www.ncbi.nlm.nih.gov/gene/?term=79709)(22.373)[NEDD4](http://www.ncbi.nlm.nih.gov/gene/?term=4734)(22.373)[CYP11A1](http://www.ncbi.nlm.nih.gov/gene/?term=1583)(22.373)[DHODH](http://www.ncbi.nlm.nih.gov/gene/?term=1723)(22.373)[CLN3](http://www.ncbi.nlm.nih.gov/gene/?term=1201)(22.373) [ARID1A](http://www.ncbi.nlm.nih.gov/gene/?term=8289)(22.373)[EGLN2](http://www.ncbi.nlm.nih.gov/gene/?term=112398)(22.373)[ACAT1](http://www.ncbi.nlm.nih.gov/gene/?term=38)(22.373)[GALC](http://www.ncbi.nlm.nih.gov/gene/?term=2581)(22.373)[PLCG2](http://www.ncbi.nlm.nih.gov/gene/?term=5336)(22.373) [ACOT4](http://www.ncbi.nlm.nih.gov/gene/?term=122970)(22.373)[PHGDH](http://www.ncbi.nlm.nih.gov/gene/?term=26227)(22.373)[PNLIPRP2](http://www.ncbi.nlm.nih.gov/gene/?term=5408)(22.373)[CYP39A1](http://www.ncbi.nlm.nih.gov/gene/?term=51302)(22.373)[BCKDHA](http://www.ncbi.nlm.nih.gov/gene/?term=593)(22.373) [SLC25A12](http://www.ncbi.nlm.nih.gov/gene/?term=8604)(22.373)[YBX3](http://www.ncbi.nlm.nih.gov/gene/?term=8531)(22.373)[8-Mar](http://www.ncbi.nlm.nih.gov/gene/?term=220972)(22.373)[STIM1](http://www.ncbi.nlm.nih.gov/gene/?term=6786)(22.373)[GAL3ST1](http://www.ncbi.nlm.nih.gov/gene/?term=9514)(22.373) [CDH11](http://www.ncbi.nlm.nih.gov/gene/?term=1009)(22.373)[CACNA1D](http://www.ncbi.nlm.nih.gov/gene/?term=776)(22.373)[DDC](http://www.ncbi.nlm.nih.gov/gene/?term=1644)(22.373)[DHTKD1](http://www.ncbi.nlm.nih.gov/gene/?term=55526)(22.373)[NEDD4L](http://www.ncbi.nlm.nih.gov/gene/?term=23327)(22.373) [BDKRB2](http://www.ncbi.nlm.nih.gov/gene/?term=624)(22.373)[PRPH](http://www.ncbi.nlm.nih.gov/gene/?term=5630)(22.373)[BCKDHB](http://www.ncbi.nlm.nih.gov/gene/?term=594)(22.373)[KCNE5](http://www.ncbi.nlm.nih.gov/gene/?term=23630)(22.373)[SRD5A3](http://www.ncbi.nlm.nih.gov/gene/?term=79644)(22.373) [SMAD7](http://www.ncbi.nlm.nih.gov/gene/?term=4092)(22.373)[PRDM8](http://www.ncbi.nlm.nih.gov/gene/?term=56978)(22.373)[UROD](http://www.ncbi.nlm.nih.gov/gene/?term=7389)(22.373)[UGT8](http://www.ncbi.nlm.nih.gov/gene/?term=7368)(22.373)[ETHE1](http://www.ncbi.nlm.nih.gov/gene/?term=23474)(22.373) [WNT10B](http://www.ncbi.nlm.nih.gov/gene/?term=7480)(22.373)[OGFOD1](http://www.ncbi.nlm.nih.gov/gene/?term=55239)(22.373)[SLC38A7](http://www.ncbi.nlm.nih.gov/gene/?term=55238)(22.373)[ACSS1](http://www.ncbi.nlm.nih.gov/gene/?term=84532)(22.373)[CYP4B1](http://www.ncbi.nlm.nih.gov/gene/?term=1580)(22.373) [STAR](http://www.ncbi.nlm.nih.gov/gene/?term=6770)(22.373)[DLST](http://www.ncbi.nlm.nih.gov/gene/?term=1743)(22.373)[PF4](http://www.ncbi.nlm.nih.gov/gene/?term=5196)(22.373)[CRACR2A](http://www.ncbi.nlm.nih.gov/gene/?term=84766)(22.373)[HSD17B1](http://www.ncbi.nlm.nih.gov/gene/?term=3292)(22.373) [CAMK2D](http://www.ncbi.nlm.nih.gov/gene/?term=817)(22.373)[AP3D1](http://www.ncbi.nlm.nih.gov/gene/?term=8943)(22.373)[MYO5A](http://www.ncbi.nlm.nih.gov/gene/?term=4644)(22.373)[ILVBL](http://www.ncbi.nlm.nih.gov/gene/?term=10994)(22.373)[CAV3](http://www.ncbi.nlm.nih.gov/gene/?term=859)(22.373) [HTT](http://www.ncbi.nlm.nih.gov/gene/?term=3064)(22.373)[IL10](http://www.ncbi.nlm.nih.gov/gene/?term=3586)(22.373)[EGLN3](http://www.ncbi.nlm.nih.gov/gene/?term=112399)(22.373)[OGDHL](http://www.ncbi.nlm.nih.gov/gene/?term=55753)(22.373)[KCNA5](http://www.ncbi.nlm.nih.gov/gene/?term=3741)(22.373) [TST](http://www.ncbi.nlm.nih.gov/gene/?term=7263)(22.373)[SLC1A6](http://www.ncbi.nlm.nih.gov/gene/?term=6511)(22.373)[NPPA](http://www.ncbi.nlm.nih.gov/gene/?term=4878)(22.373)[ACADM](http://www.ncbi.nlm.nih.gov/gene/?term=34)(22.373)[SQRDL](http://www.ncbi.nlm.nih.gov/gene/?term=58472)(22.373) [TMEM110](http://www.ncbi.nlm.nih.gov/gene/?term=375346)(22.373)[CDC42](http://www.ncbi.nlm.nih.gov/gene/?term=998)(22.373)[ARHGEF2](http://www.ncbi.nlm.nih.gov/gene/?term=9181)(22.373)[EGLN1](http://www.ncbi.nlm.nih.gov/gene/?term=54583)(22.373)[ETFDH](http://www.ncbi.nlm.nih.gov/gene/?term=2110)(22.373) [EPHA4](http://www.ncbi.nlm.nih.gov/gene/?term=2043)(22.373)[OCA2](http://www.ncbi.nlm.nih.gov/gene/?term=4948)(22.373)[HSD17B8](http://www.ncbi.nlm.nih.gov/gene/?term=7923)(22.373)[CTNNB1](http://www.ncbi.nlm.nih.gov/gene/?term=1499)(22.373)[P4HA3](http://www.ncbi.nlm.nih.gov/gene/?term=283208)(22.373) [EDA](http://www.ncbi.nlm.nih.gov/gene/?term=1896)(22.373)[HACL1](http://www.ncbi.nlm.nih.gov/gene/?term=26061)(22.373)[RYR2](http://www.ncbi.nlm.nih.gov/gene/?term=6262)(22.373)[HAP1](http://www.ncbi.nlm.nih.gov/gene/?term=9001)(22.373)[PCSK9](http://www.ncbi.nlm.nih.gov/gene/?term=255738)(22.373) [KCNQ1](http://www.ncbi.nlm.nih.gov/gene/?term=3784)(22.373)[CASQ2](http://www.ncbi.nlm.nih.gov/gene/?term=845)(22.373)[GJA5](http://www.ncbi.nlm.nih.gov/gene/?term=2702)(22.373)[DHRS9](http://www.ncbi.nlm.nih.gov/gene/?term=10170)(22.373)[GPR143](http://www.ncbi.nlm.nih.gov/gene/?term=4935)(22.373) [UGT1A1](http://www.ncbi.nlm.nih.gov/gene/?term=54658)(22.373)[DLD](http://www.ncbi.nlm.nih.gov/gene/?term=1738)(22.373)[GNAS](http://www.ncbi.nlm.nih.gov/gene/?term=2778)(22.373)[SLC25A13](http://www.ncbi.nlm.nih.gov/gene/?term=10165)(22.373)[SFXN5](http://www.ncbi.nlm.nih.gov/gene/?term=94097)(22.373) [PLOD2](http://www.ncbi.nlm.nih.gov/gene/?term=5352)(22.373)[CYP1A1](http://www.ncbi.nlm.nih.gov/gene/?term=1543)(22.373)[SULT2A1](http://www.ncbi.nlm.nih.gov/gene/?term=6822)(22.373)[NFX1](http://www.ncbi.nlm.nih.gov/gene/?term=4799)(22.373)[DBT](http://www.ncbi.nlm.nih.gov/gene/?term=1629)(22.373) [SLC25A1](http://www.ncbi.nlm.nih.gov/gene/?term=6576)(22.373) | |
| [Quercetin](http://www.megabionet.org/tcmid/ingredient/18302/) | [HTR2A](http://www.ncbi.nlm.nih.gov/gene/?term=3356)(26.373)[HTR2B](http://www.ncbi.nlm.nih.gov/gene/?term=3357)(26.373)[ADRA2A](http://www.ncbi.nlm.nih.gov/gene/?term=150)(26.373)[HTR1B](http://www.ncbi.nlm.nih.gov/gene/?term=3351)(26.373)[HTR2C](http://www.ncbi.nlm.nih.gov/gene/?term=3358)(26.373) [HTR1D](http://www.ncbi.nlm.nih.gov/gene/?term=3352)(26.373)[DRD2](http://www.ncbi.nlm.nih.gov/gene/?term=1813)(26.373)[DRD4](http://www.ncbi.nlm.nih.gov/gene/?term=1815)(26.373)[HTR1A](http://www.ncbi.nlm.nih.gov/gene/?term=3350)(26.373)[ADRA2C](http://www.ncbi.nlm.nih.gov/gene/?term=152)(26.373) [CALY](http://www.ncbi.nlm.nih.gov/gene/?term=50632)(26.373)[DRD3](http://www.ncbi.nlm.nih.gov/gene/?term=1814)(26.373)[DRD5](http://www.ncbi.nlm.nih.gov/gene/?term=1816)(26.373)[DRD1](http://www.ncbi.nlm.nih.gov/gene/?term=1812)(26.373)[ADRA2B](http://www.ncbi.nlm.nih.gov/gene/?term=151)(26.373) | |
| [Kumatakenin](http://www.megabionet.org/tcmid/ingredient/12327/) | This compound doesn't have any potential target with score larger than 20. | |
| [Friedelin](http://www.megabionet.org/tcmid/ingredient/24310/) | [AR](http://www.ncbi.nlm.nih.gov/gene/?term=367)(48.000) | |
| [2-Methyl-1,3,6-Trihydroxyanthraquinone](http://www.megabionet.org/tcmid/ingredient/14767/) | [ESR1](http://www.ncbi.nlm.nih.gov/gene/?term=2099)(80.882)[HSD17B1](http://www.ncbi.nlm.nih.gov/gene/?term=3292)(23.000)[TFAP2C](http://www.ncbi.nlm.nih.gov/gene/?term=7022)(22.373)[TOX3](http://www.ncbi.nlm.nih.gov/gene/?term=27324)(22.373)[TGFB1](http://www.ncbi.nlm.nih.gov/gene/?term=7040)(22.373) [MED1](http://www.ncbi.nlm.nih.gov/gene/?term=5469)(22.373)[NKX3-1](http://www.ncbi.nlm.nih.gov/gene/?term=4824)(22.373)[ESR2](http://www.ncbi.nlm.nih.gov/gene/?term=2100)(22.373)[SHH](http://www.ncbi.nlm.nih.gov/gene/?term=6469)(22.373)[LEF1](http://www.ncbi.nlm.nih.gov/gene/?term=51176)(22.373) [FGFR2](http://www.ncbi.nlm.nih.gov/gene/?term=2263)(22.373)[PGR](http://www.ncbi.nlm.nih.gov/gene/?term=5241)(22.373)[WNT4](http://www.ncbi.nlm.nih.gov/gene/?term=54361)(22.373)[AREG](http://www.ncbi.nlm.nih.gov/gene/?term=374)(22.373)[GPER1](http://www.ncbi.nlm.nih.gov/gene/?term=2852)(22.373) [WNT5A](http://www.ncbi.nlm.nih.gov/gene/?term=7474)(22.373)[SOX9](http://www.ncbi.nlm.nih.gov/gene/?term=6662)(22.373)[VDR](http://www.ncbi.nlm.nih.gov/gene/?term=7421)(22.373)[TRIM24](http://www.ncbi.nlm.nih.gov/gene/?term=8805)(22.373) | |
| [Nicotinic Acid](http://www.megabionet.org/tcmid/ingredient/23165/) | [HCAR2](http://www.ncbi.nlm.nih.gov/gene/?term=338442)(686.000)[QPRT](http://www.ncbi.nlm.nih.gov/gene/?term=23475)(686.000)[HCAR3](http://www.ncbi.nlm.nih.gov/gene/?term=8843)(686.000)[NNMT](http://www.ncbi.nlm.nih.gov/gene/?term=4837)(686.000)[PLAT](http://www.ncbi.nlm.nih.gov/gene/?term=5327)(80.882) [THBD](http://www.ncbi.nlm.nih.gov/gene/?term=7056)(80.882)[PPARG](http://www.ncbi.nlm.nih.gov/gene/?term=5468)(80.882)[BCL2](http://www.ncbi.nlm.nih.gov/gene/?term=596)(80.882)[FABP2](http://www.ncbi.nlm.nih.gov/gene/?term=2169)(80.882)[CFTR](http://www.ncbi.nlm.nih.gov/gene/?term=1080)(80.882) [PTGS1](http://www.ncbi.nlm.nih.gov/gene/?term=5742)(80.882)[PTGS2](http://www.ncbi.nlm.nih.gov/gene/?term=5743)(80.882)[APOA2](http://www.ncbi.nlm.nih.gov/gene/?term=336)(55.444)[TCAF1](http://www.ncbi.nlm.nih.gov/gene/?term=9747)(55.444)[IL1B](http://www.ncbi.nlm.nih.gov/gene/?term=3553)(55.444) [IL13](http://www.ncbi.nlm.nih.gov/gene/?term=3596)(55.444)[C1QTNF3](http://www.ncbi.nlm.nih.gov/gene/?term=114899)(55.444)[KCNE2](http://www.ncbi.nlm.nih.gov/gene/?term=9992)(55.444)[ANXA1](http://www.ncbi.nlm.nih.gov/gene/?term=301)(55.444)[SERPINB7](http://www.ncbi.nlm.nih.gov/gene/?term=8710)(55.444) [PDXP](http://www.ncbi.nlm.nih.gov/gene/?term=57026)(22.373)[NDRG2](http://www.ncbi.nlm.nih.gov/gene/?term=57447)(22.373)[ASPDH](http://www.ncbi.nlm.nih.gov/gene/?term=554235)(22.373)[ATOH1](http://www.ncbi.nlm.nih.gov/gene/?term=474)(22.373)[SLC4A7](http://www.ncbi.nlm.nih.gov/gene/?term=9497)(22.373) [NOX1](http://www.ncbi.nlm.nih.gov/gene/?term=27035)(22.373)[NAMPT](http://www.ncbi.nlm.nih.gov/gene/?term=10135)(22.373)[RGCC](http://www.ncbi.nlm.nih.gov/gene/?term=28984)(22.373)[SIRT1](http://www.ncbi.nlm.nih.gov/gene/?term=23411)(22.373)[NGFR](http://www.ncbi.nlm.nih.gov/gene/?term=4804)(22.373) [FNDC5](http://www.ncbi.nlm.nih.gov/gene/?term=252995)(22.373)[RAB11FIP5](http://www.ncbi.nlm.nih.gov/gene/?term=26056)(22.373)[NR1H3](http://www.ncbi.nlm.nih.gov/gene/?term=10062)(22.373)[ADAMTS20](http://www.ncbi.nlm.nih.gov/gene/?term=80070)(22.373)[SIRT2](http://www.ncbi.nlm.nih.gov/gene/?term=22933)(22.373) [OSBPL8](http://www.ncbi.nlm.nih.gov/gene/?term=114882)(22.373)[CA7](http://www.ncbi.nlm.nih.gov/gene/?term=766)(22.373)[MYF5](http://www.ncbi.nlm.nih.gov/gene/?term=4617)(22.373)[ACMSD](http://www.ncbi.nlm.nih.gov/gene/?term=130013)(22.373)[AVPR1A](http://www.ncbi.nlm.nih.gov/gene/?term=552)(22.373) [DNAJC3](http://www.ncbi.nlm.nih.gov/gene/?term=5611)(22.373)[ANO9](http://www.ncbi.nlm.nih.gov/gene/?term=338440)(22.373)[SHOX2](http://www.ncbi.nlm.nih.gov/gene/?term=6474)(22.373)[KITLG](http://www.ncbi.nlm.nih.gov/gene/?term=4254)(22.373)[FASLG](http://www.ncbi.nlm.nih.gov/gene/?term=356)(22.373) [ITGB3](http://www.ncbi.nlm.nih.gov/gene/?term=3690)(22.373)[TP53](http://www.ncbi.nlm.nih.gov/gene/?term=7157)(22.373)[PRKDC](http://www.ncbi.nlm.nih.gov/gene/?term=5591)(22.373)[METRNL](http://www.ncbi.nlm.nih.gov/gene/?term=284207)(22.373)[MAPK9](http://www.ncbi.nlm.nih.gov/gene/?term=5601)(22.373) [ADAMTS9](http://www.ncbi.nlm.nih.gov/gene/?term=56999)(22.373)[SLC26A6](http://www.ncbi.nlm.nih.gov/gene/?term=65010)(22.373)[FXYD1](http://www.ncbi.nlm.nih.gov/gene/?term=5348)(22.373)[STX3](http://www.ncbi.nlm.nih.gov/gene/?term=6809)(22.373)[OXER1](http://www.ncbi.nlm.nih.gov/gene/?term=165140)(22.373) [PNPLA2](http://www.ncbi.nlm.nih.gov/gene/?term=57104)(22.373)[MYF6](http://www.ncbi.nlm.nih.gov/gene/?term=4618)(22.373)[MEF2C](http://www.ncbi.nlm.nih.gov/gene/?term=4208)(22.373)[PTGIS](http://www.ncbi.nlm.nih.gov/gene/?term=5740)(22.373)[RUNX1](http://www.ncbi.nlm.nih.gov/gene/?term=861)(22.373) [CLN3](http://www.ncbi.nlm.nih.gov/gene/?term=1201)(22.373)[PNP](http://www.ncbi.nlm.nih.gov/gene/?term=4860)(22.373)[AURKA](http://www.ncbi.nlm.nih.gov/gene/?term=6790)(22.373)[HEG1](http://www.ncbi.nlm.nih.gov/gene/?term=57493)(22.373)[HSH2D](http://www.ncbi.nlm.nih.gov/gene/?term=84941)(22.373) [ITGAV](http://www.ncbi.nlm.nih.gov/gene/?term=3685)(22.373)[SCRIB](http://www.ncbi.nlm.nih.gov/gene/?term=23513)(22.373)[MYOD1](http://www.ncbi.nlm.nih.gov/gene/?term=4654)(22.373)[MCL1](http://www.ncbi.nlm.nih.gov/gene/?term=4170)(22.373)[CNTF](http://www.ncbi.nlm.nih.gov/gene/?term=1270)(22.373) [PTPN2](http://www.ncbi.nlm.nih.gov/gene/?term=5771)(22.373)[S100A8](http://www.ncbi.nlm.nih.gov/gene/?term=6279)(22.373)[ALOX5AP](http://www.ncbi.nlm.nih.gov/gene/?term=241)(22.373)[TCF3](http://www.ncbi.nlm.nih.gov/gene/?term=6929)(22.373)[SRC](http://www.ncbi.nlm.nih.gov/gene/?term=6714)(22.373) [AVP](http://www.ncbi.nlm.nih.gov/gene/?term=551)(22.373)[BAX](http://www.ncbi.nlm.nih.gov/gene/?term=581)(22.373)[IFNG](http://www.ncbi.nlm.nih.gov/gene/?term=3458)(22.373)[SEC24B](http://www.ncbi.nlm.nih.gov/gene/?term=10427)(22.373)[SLC6A4](http://www.ncbi.nlm.nih.gov/gene/?term=6532)(22.373) [TCAF2](http://www.ncbi.nlm.nih.gov/gene/?term=285966)(22.373)[BDKRB2](http://www.ncbi.nlm.nih.gov/gene/?term=624)(22.373)[NR1H2](http://www.ncbi.nlm.nih.gov/gene/?term=7376)(22.373)[NEUROD2](http://www.ncbi.nlm.nih.gov/gene/?term=4761)(22.373)[INS](http://www.ncbi.nlm.nih.gov/gene/?term=3630)(22.373) [NGF](http://www.ncbi.nlm.nih.gov/gene/?term=4803)(22.373)[RAB11FIP3](http://www.ncbi.nlm.nih.gov/gene/?term=9727)(22.373)[ANAPC2](http://www.ncbi.nlm.nih.gov/gene/?term=29882)(22.373)[HAAO](http://www.ncbi.nlm.nih.gov/gene/?term=23498)(22.373)[WNT11](http://www.ncbi.nlm.nih.gov/gene/?term=7481)(22.373) [CDKN2A](http://www.ncbi.nlm.nih.gov/gene/?term=1029)(22.373)[ALOX15B](http://www.ncbi.nlm.nih.gov/gene/?term=247)(22.373)[CAV3](http://www.ncbi.nlm.nih.gov/gene/?term=859)(22.373)[HTT](http://www.ncbi.nlm.nih.gov/gene/?term=3064)(22.373)[SLC26A3](http://www.ncbi.nlm.nih.gov/gene/?term=1811)(22.373) [CDC20](http://www.ncbi.nlm.nih.gov/gene/?term=991)(22.373)[NUDT12](http://www.ncbi.nlm.nih.gov/gene/?term=83594)(22.373)[DNAJA3](http://www.ncbi.nlm.nih.gov/gene/?term=9093)(22.373)[ZEB2](http://www.ncbi.nlm.nih.gov/gene/?term=9839)(22.373)[IL6](http://www.ncbi.nlm.nih.gov/gene/?term=3569)(22.373) [RET](http://www.ncbi.nlm.nih.gov/gene/?term=5979)(22.373)[BCL2L1](http://www.ncbi.nlm.nih.gov/gene/?term=598)(22.373)[COL1A1](http://www.ncbi.nlm.nih.gov/gene/?term=1277)(22.373)[BDNF](http://www.ncbi.nlm.nih.gov/gene/?term=627)(22.373)[NR1D1](http://www.ncbi.nlm.nih.gov/gene/?term=9572)(22.373) [IFI6](http://www.ncbi.nlm.nih.gov/gene/?term=2537)(22.373)[ABCA1](http://www.ncbi.nlm.nih.gov/gene/?term=19)(22.373)[KMO](http://www.ncbi.nlm.nih.gov/gene/?term=8564)(22.373)[NAPRT](http://www.ncbi.nlm.nih.gov/gene/?term=93100)(22.373)[SLC34A1](http://www.ncbi.nlm.nih.gov/gene/?term=6569)(22.373) [MECOM](http://www.ncbi.nlm.nih.gov/gene/?term=2122)(22.373)[ABHD5](http://www.ncbi.nlm.nih.gov/gene/?term=51099)(22.373)[SNCA](http://www.ncbi.nlm.nih.gov/gene/?term=6622)(22.373)[ADIPOQ](http://www.ncbi.nlm.nih.gov/gene/?term=9370)(22.373)[CPLX2](http://www.ncbi.nlm.nih.gov/gene/?term=10814)(22.373) [PPARA](http://www.ncbi.nlm.nih.gov/gene/?term=5465)(22.373)[CA2](http://www.ncbi.nlm.nih.gov/gene/?term=760)(22.373)[ALOX15](http://www.ncbi.nlm.nih.gov/gene/?term=246)(22.373)[PAWR](http://www.ncbi.nlm.nih.gov/gene/?term=5074)(22.373)[MYOG](http://www.ncbi.nlm.nih.gov/gene/?term=4656)(22.373) [S100A9](http://www.ncbi.nlm.nih.gov/gene/?term=6280)(22.373)[RAB11FIP1](http://www.ncbi.nlm.nih.gov/gene/?term=80223)(22.373)[KYNU](http://www.ncbi.nlm.nih.gov/gene/?term=8942)(22.373)[FABP3](http://www.ncbi.nlm.nih.gov/gene/?term=2170)(22.373)[SEC61B](http://www.ncbi.nlm.nih.gov/gene/?term=10952)(22.373) [NR1H4](http://www.ncbi.nlm.nih.gov/gene/?term=9971)(22.373)[ABCG1](http://www.ncbi.nlm.nih.gov/gene/?term=9619)(22.373)[PRDM16](http://www.ncbi.nlm.nih.gov/gene/?term=63976)(22.373)[RAG2](http://www.ncbi.nlm.nih.gov/gene/?term=5897)(22.373)[CCM2L](http://www.ncbi.nlm.nih.gov/gene/?term=140706)(22.373) | |
| [Uralenin](http://www.megabionet.org/tcmid/ingredient/22223/) | This compound doesn't have any potential target with score larger than 20. | |
| [N-Nonadecane](http://www.megabionet.org/tcmid/ingredient/15662/) | This compound doesn't have any potential target with score larger than 20. | |
| [Wogonin](http://www.megabionet.org/tcmid/ingredient/22702/) | This compound doesn't have any potential target with score larger than 20. | |
| [Neohancoside A](http://www.megabionet.org/tcmid/ingredient/15393/) | [ATP1A1](http://www.ncbi.nlm.nih.gov/gene/?term=476)(48.000) | |
| [Astragaloside Iii](http://www.megabionet.org/tcmid/ingredient/1938/) | [CXCR4](http://www.ncbi.nlm.nih.gov/gene/?term=7852)(48.000) | |
| [Phenylalanine](http://www.megabionet.org/tcmid/ingredient/17079/) | [SLC7A8](http://www.ncbi.nlm.nih.gov/gene/?term=23428)(686.000)[TH](http://www.ncbi.nlm.nih.gov/gene/?term=7054)(686.000)[TAT](http://www.ncbi.nlm.nih.gov/gene/?term=6898)(686.000)[FARS2](http://www.ncbi.nlm.nih.gov/gene/?term=10667)(686.000)[PAH](http://www.ncbi.nlm.nih.gov/gene/?term=5053)(686.000) [FARSA](http://www.ncbi.nlm.nih.gov/gene/?term=2193)(686.000)[FARSB](http://www.ncbi.nlm.nih.gov/gene/?term=10056)(686.000)[YARS](http://www.ncbi.nlm.nih.gov/gene/?term=8565)(122.778)[YARS2](http://www.ncbi.nlm.nih.gov/gene/?term=51067)(122.778)[BCO2](http://www.ncbi.nlm.nih.gov/gene/?term=83875)(55.444) [PNMT](http://www.ncbi.nlm.nih.gov/gene/?term=5409)(55.444)[DDC](http://www.ncbi.nlm.nih.gov/gene/?term=1644)(55.444)[SLC6A3](http://www.ncbi.nlm.nih.gov/gene/?term=6531)(48.000)[ATF4](http://www.ncbi.nlm.nih.gov/gene/?term=468)(48.000)[ADRA1A](http://www.ncbi.nlm.nih.gov/gene/?term=148)(48.000) [ADRB2](http://www.ncbi.nlm.nih.gov/gene/?term=154)(48.000)[JDP2](http://www.ncbi.nlm.nih.gov/gene/?term=122953)(48.000)[IL2](http://www.ncbi.nlm.nih.gov/gene/?term=3558)(48.000)[ADRA2A](http://www.ncbi.nlm.nih.gov/gene/?term=150)(48.000)[ATF3](http://www.ncbi.nlm.nih.gov/gene/?term=467)(48.000) [TNF](http://www.ncbi.nlm.nih.gov/gene/?term=7124)(48.000)[MAOB](http://www.ncbi.nlm.nih.gov/gene/?term=4129)(48.000)[ATF2](http://www.ncbi.nlm.nih.gov/gene/?term=1386)(48.000)[ATF1](http://www.ncbi.nlm.nih.gov/gene/?term=466)(48.000)[ADRB1](http://www.ncbi.nlm.nih.gov/gene/?term=153)(48.000) [NFATC1](http://www.ncbi.nlm.nih.gov/gene/?term=4772)(48.000)[ADRA1D](http://www.ncbi.nlm.nih.gov/gene/?term=146)(48.000)[DRD2](http://www.ncbi.nlm.nih.gov/gene/?term=1813)(48.000)[ATF5](http://www.ncbi.nlm.nih.gov/gene/?term=22809)(48.000)[JUN](http://www.ncbi.nlm.nih.gov/gene/?term=3725)(48.000) [MAOA](http://www.ncbi.nlm.nih.gov/gene/?term=4128)(48.000)[SLC6A4](http://www.ncbi.nlm.nih.gov/gene/?term=6532)(48.000)[ACHE](http://www.ncbi.nlm.nih.gov/gene/?term=43)(48.000)[SLC18A2](http://www.ncbi.nlm.nih.gov/gene/?term=6571)(48.000)[GABBR2](http://www.ncbi.nlm.nih.gov/gene/?term=9568)(48.000) [ATF7](http://www.ncbi.nlm.nih.gov/gene/?term=11016)(48.000)[DRD4](http://www.ncbi.nlm.nih.gov/gene/?term=1815)(48.000)[ADRA1B](http://www.ncbi.nlm.nih.gov/gene/?term=147)(48.000)[ADRA2C](http://www.ncbi.nlm.nih.gov/gene/?term=152)(48.000)[DRD3](http://www.ncbi.nlm.nih.gov/gene/?term=1814)(48.000) [DRD5](http://www.ncbi.nlm.nih.gov/gene/?term=1816)(48.000)[ATF6](http://www.ncbi.nlm.nih.gov/gene/?term=22926)(48.000)[DRD1](http://www.ncbi.nlm.nih.gov/gene/?term=1812)(48.000)[ADRB3](http://www.ncbi.nlm.nih.gov/gene/?term=155)(48.000)[GABBR1](http://www.ncbi.nlm.nih.gov/gene/?term=2550)(48.000) [TAAR1](http://www.ncbi.nlm.nih.gov/gene/?term=134864)(48.000)[CARTPT](http://www.ncbi.nlm.nih.gov/gene/?term=9607)(48.000)[ADRA2B](http://www.ncbi.nlm.nih.gov/gene/?term=151)(48.000)[FOS](http://www.ncbi.nlm.nih.gov/gene/?term=2353)(48.000)[SLC6A2](http://www.ncbi.nlm.nih.gov/gene/?term=6530)(48.000) [SLC1A5](http://www.ncbi.nlm.nih.gov/gene/?term=6510)(23.000)[GOT2](http://www.ncbi.nlm.nih.gov/gene/?term=2806)(23.000)[GPT2](http://www.ncbi.nlm.nih.gov/gene/?term=84706)(23.000)[BAAT](http://www.ncbi.nlm.nih.gov/gene/?term=570)(23.000)[OAZ3](http://www.ncbi.nlm.nih.gov/gene/?term=51686)(23.000) [SHMT1](http://www.ncbi.nlm.nih.gov/gene/?term=6470)(23.000)[ABAT](http://www.ncbi.nlm.nih.gov/gene/?term=18)(23.000)[SLC1A1](http://www.ncbi.nlm.nih.gov/gene/?term=6505)(23.000)[SLC7A2](http://www.ncbi.nlm.nih.gov/gene/?term=6542)(23.000)[GCSH](http://www.ncbi.nlm.nih.gov/gene/?term=2653)(23.000) [PPAT](http://www.ncbi.nlm.nih.gov/gene/?term=5471)(23.000)[ACY3](http://www.ncbi.nlm.nih.gov/gene/?term=91703)(23.000)[ASPA](http://www.ncbi.nlm.nih.gov/gene/?term=443)(23.000)[IARS2](http://www.ncbi.nlm.nih.gov/gene/?term=55699)(23.000)[GLRA2](http://www.ncbi.nlm.nih.gov/gene/?term=2742)(23.000) [ALAS1](http://www.ncbi.nlm.nih.gov/gene/?term=211)(23.000)[BCAT1](http://www.ncbi.nlm.nih.gov/gene/?term=586)(23.000)[GCAT](http://www.ncbi.nlm.nih.gov/gene/?term=23464)(23.000)[SLC38A3](http://www.ncbi.nlm.nih.gov/gene/?term=10991)(23.000)[VARS](http://www.ncbi.nlm.nih.gov/gene/?term=7407)(23.000) [NFS1](http://www.ncbi.nlm.nih.gov/gene/?term=9054)(23.000)[SLC25A15](http://www.ncbi.nlm.nih.gov/gene/?term=10166)(23.000)[SLC1A4](http://www.ncbi.nlm.nih.gov/gene/?term=6509)(23.000)[ADSSL1](http://www.ncbi.nlm.nih.gov/gene/?term=122622)(23.000)[VDAC3](http://www.ncbi.nlm.nih.gov/gene/?term=7419)(23.000) [GSS](http://www.ncbi.nlm.nih.gov/gene/?term=2937)(23.000)[ASNS](http://www.ncbi.nlm.nih.gov/gene/?term=440)(23.000)[GLRA3](http://www.ncbi.nlm.nih.gov/gene/?term=8001)(23.000)[DARS2](http://www.ncbi.nlm.nih.gov/gene/?term=55157)(23.000)[GRIN3B](http://www.ncbi.nlm.nih.gov/gene/?term=116444)(23.000) [LCMT1](http://www.ncbi.nlm.nih.gov/gene/?term=51451)(23.000)[GRIN2A](http://www.ncbi.nlm.nih.gov/gene/?term=2903)(23.000)[GOT1](http://www.ncbi.nlm.nih.gov/gene/?term=2805)(23.000)[GNMT](http://www.ncbi.nlm.nih.gov/gene/?term=27232)(23.000)[HDC](http://www.ncbi.nlm.nih.gov/gene/?term=3067)(23.000) [GLRB](http://www.ncbi.nlm.nih.gov/gene/?term=2743)(23.000)[GLYAT](http://www.ncbi.nlm.nih.gov/gene/?term=10249)(23.000)[AARS2](http://www.ncbi.nlm.nih.gov/gene/?term=57505)(23.000)[ASPH](http://www.ncbi.nlm.nih.gov/gene/?term=444)(23.000)[WARS2](http://www.ncbi.nlm.nih.gov/gene/?term=10352)(23.000) [GPR18](http://www.ncbi.nlm.nih.gov/gene/?term=2841)(23.000)[CTPS1](http://www.ncbi.nlm.nih.gov/gene/?term=1503)(23.000)[SLC25A12](http://www.ncbi.nlm.nih.gov/gene/?term=8604)(23.000)[PHYKPL](http://www.ncbi.nlm.nih.gov/gene/?term=85007)(23.000)[HAL](http://www.ncbi.nlm.nih.gov/gene/?term=3034)(23.000) [GLYATL1](http://www.ncbi.nlm.nih.gov/gene/?term=92292)(23.000)[GATM](http://www.ncbi.nlm.nih.gov/gene/?term=2628)(23.000)[LARS](http://www.ncbi.nlm.nih.gov/gene/?term=51520)(23.000)[ARG1](http://www.ncbi.nlm.nih.gov/gene/?term=383)(23.000)[NARS2](http://www.ncbi.nlm.nih.gov/gene/?term=79731)(23.000) [TNNC1](http://www.ncbi.nlm.nih.gov/gene/?term=7134)(23.000)[WARS](http://www.ncbi.nlm.nih.gov/gene/?term=7453)(23.000)[DARS](http://www.ncbi.nlm.nih.gov/gene/?term=1615)(23.000)[GRIN2C](http://www.ncbi.nlm.nih.gov/gene/?term=2905)(23.000)[OAT](http://www.ncbi.nlm.nih.gov/gene/?term=4942)(23.000) [GLDC](http://www.ncbi.nlm.nih.gov/gene/?term=2731)(23.000)[VDAC2](http://www.ncbi.nlm.nih.gov/gene/?term=7417)(23.000)[OAZ2](http://www.ncbi.nlm.nih.gov/gene/?term=4947)(23.000)[PIPOX](http://www.ncbi.nlm.nih.gov/gene/?term=51268)(23.000)[SLC25A2](http://www.ncbi.nlm.nih.gov/gene/?term=83884)(23.000) [GARS](http://www.ncbi.nlm.nih.gov/gene/?term=2617)(23.000)[GPT](http://www.ncbi.nlm.nih.gov/gene/?term=2875)(23.000)[AARS](http://www.ncbi.nlm.nih.gov/gene/?term=16)(23.000)[SHMT2](http://www.ncbi.nlm.nih.gov/gene/?term=6472)(23.000)[ALAS2](http://www.ncbi.nlm.nih.gov/gene/?term=212)(23.000) [VDAC1](http://www.ncbi.nlm.nih.gov/gene/?term=7416)(23.000)[SLC6A9](http://www.ncbi.nlm.nih.gov/gene/?term=6536)(23.000)[SLC7A3](http://www.ncbi.nlm.nih.gov/gene/?term=84889)(23.000)[ASRGL1](http://www.ncbi.nlm.nih.gov/gene/?term=80150)(23.000)[GLYATL2](http://www.ncbi.nlm.nih.gov/gene/?term=219970)(23.000) [SLC32A1](http://www.ncbi.nlm.nih.gov/gene/?term=140679)(23.000)[OAZ1](http://www.ncbi.nlm.nih.gov/gene/?term=4946)(23.000)[KARS](http://www.ncbi.nlm.nih.gov/gene/?term=3735)(23.000)[AGXT](http://www.ncbi.nlm.nih.gov/gene/?term=189)(23.000)[LARS2](http://www.ncbi.nlm.nih.gov/gene/?term=23395)(23.000) [RNASE1](http://www.ncbi.nlm.nih.gov/gene/?term=6035)(23.000)[ASS1](http://www.ncbi.nlm.nih.gov/gene/?term=445)(23.000)[ADSS](http://www.ncbi.nlm.nih.gov/gene/?term=159)(23.000)[SLC7A1](http://www.ncbi.nlm.nih.gov/gene/?term=6541)(23.000)[SLC36A1](http://www.ncbi.nlm.nih.gov/gene/?term=206358)(23.000) [ACADSB](http://www.ncbi.nlm.nih.gov/gene/?term=36)(23.000)[SLC6A5](http://www.ncbi.nlm.nih.gov/gene/?term=9152)(23.000)[SLC7A4](http://www.ncbi.nlm.nih.gov/gene/?term=6545)(23.000)[HARS](http://www.ncbi.nlm.nih.gov/gene/?term=3035)(23.000)[CAD](http://www.ncbi.nlm.nih.gov/gene/?term=790)(23.000) [PCCB](http://www.ncbi.nlm.nih.gov/gene/?term=5096)(23.000)[OTC](http://www.ncbi.nlm.nih.gov/gene/?term=5009)(23.000)[GLUL](http://www.ncbi.nlm.nih.gov/gene/?term=2752)(23.000)[AGXT2](http://www.ncbi.nlm.nih.gov/gene/?term=64902)(23.000)[ARG2](http://www.ncbi.nlm.nih.gov/gene/?term=384)(23.000) [IARS](http://www.ncbi.nlm.nih.gov/gene/?term=3376)(23.000)[BCAT2](http://www.ncbi.nlm.nih.gov/gene/?term=587)(23.000)[ACY1](http://www.ncbi.nlm.nih.gov/gene/?term=95)(23.000)[KYNU](http://www.ncbi.nlm.nih.gov/gene/?term=8942)(23.000)[SLC25A13](http://www.ncbi.nlm.nih.gov/gene/?term=10165)(23.000) [GLRA1](http://www.ncbi.nlm.nih.gov/gene/?term=2741)(23.000)[LYZ](http://www.ncbi.nlm.nih.gov/gene/?term=4069)(23.000)[LCMT2](http://www.ncbi.nlm.nih.gov/gene/?term=9836)(23.000)[PAICS](http://www.ncbi.nlm.nih.gov/gene/?term=10606)(23.000)[NARS](http://www.ncbi.nlm.nih.gov/gene/?term=4677)(23.000) [SCN2B](http://www.ncbi.nlm.nih.gov/gene/?term=6327)(22.373)[PCK1](http://www.ncbi.nlm.nih.gov/gene/?term=5105)(22.373)[CCBL1](http://www.ncbi.nlm.nih.gov/gene/?term=883)(22.373)[LRRC47](http://www.ncbi.nlm.nih.gov/gene/?term=57470)(22.373)[PRG3](http://www.ncbi.nlm.nih.gov/gene/?term=10394)(22.373) [PCBD2](http://www.ncbi.nlm.nih.gov/gene/?term=84105)(22.373)[HGD](http://www.ncbi.nlm.nih.gov/gene/?term=3081)(22.373)[MBD1](http://www.ncbi.nlm.nih.gov/gene/?term=4152)(22.373)[OXT](http://www.ncbi.nlm.nih.gov/gene/?term=5020)(22.373)[HAND2](http://www.ncbi.nlm.nih.gov/gene/?term=9464)(22.373) [GSTZ1](http://www.ncbi.nlm.nih.gov/gene/?term=2954)(22.373)[CYP11A1](http://www.ncbi.nlm.nih.gov/gene/?term=1583)(22.373)[INSM1](http://www.ncbi.nlm.nih.gov/gene/?term=3642)(22.373)[HPD](http://www.ncbi.nlm.nih.gov/gene/?term=3242)(22.373)[NOS2](http://www.ncbi.nlm.nih.gov/gene/?term=4843)(22.373) [ATP7A](http://www.ncbi.nlm.nih.gov/gene/?term=538)(22.373)[CYP2D6](http://www.ncbi.nlm.nih.gov/gene/?term=1565)(22.373)[GATA3](http://www.ncbi.nlm.nih.gov/gene/?term=2625)(22.373)[CRH](http://www.ncbi.nlm.nih.gov/gene/?term=1392)(22.373)[SLC38A7](http://www.ncbi.nlm.nih.gov/gene/?term=55238)(22.373) [STAR](http://www.ncbi.nlm.nih.gov/gene/?term=6770)(22.373)[ATP13A2](http://www.ncbi.nlm.nih.gov/gene/?term=23400)(22.373)[DBH](http://www.ncbi.nlm.nih.gov/gene/?term=1621)(22.373)[LRRK2](http://www.ncbi.nlm.nih.gov/gene/?term=120892)(22.373)[BDNF](http://www.ncbi.nlm.nih.gov/gene/?term=627)(22.373) [ABCB4](http://www.ncbi.nlm.nih.gov/gene/?term=5244)(22.373)[SRD5A2](http://www.ncbi.nlm.nih.gov/gene/?term=6716)(22.373)[NOS3](http://www.ncbi.nlm.nih.gov/gene/?term=4846)(22.373)[PARK2](http://www.ncbi.nlm.nih.gov/gene/?term=5071)(22.373)[FAH](http://www.ncbi.nlm.nih.gov/gene/?term=2184)(22.373) [HARS2](http://www.ncbi.nlm.nih.gov/gene/?term=23438)(22.373)[NOS1](http://www.ncbi.nlm.nih.gov/gene/?term=4842)(22.373)[PCBD1](http://www.ncbi.nlm.nih.gov/gene/?term=5092)(22.373)[GRIN1](http://www.ncbi.nlm.nih.gov/gene/?term=2902)(22.373) | |
| [Gancaonin I](http://www.megabionet.org/tcmid/ingredient/8139/) | [CNR2](http://www.ncbi.nlm.nih.gov/gene/?term=1269)(80.882)[CNR1](http://www.ncbi.nlm.nih.gov/gene/?term=1268)(80.882)[DRD2](http://www.ncbi.nlm.nih.gov/gene/?term=1813)(55.444)[SEC14L3](http://www.ncbi.nlm.nih.gov/gene/?term=266629)(23.000)[PPP2CA](http://www.ncbi.nlm.nih.gov/gene/?term=5515)(23.000) [PRKCA](http://www.ncbi.nlm.nih.gov/gene/?term=5578)(23.000)[NR1I2](http://www.ncbi.nlm.nih.gov/gene/?term=8856)(23.000)[ALOX5](http://www.ncbi.nlm.nih.gov/gene/?term=240)(23.000)[PPP2CB](http://www.ncbi.nlm.nih.gov/gene/?term=5516)(23.000)[SEC14L2](http://www.ncbi.nlm.nih.gov/gene/?term=23541)(23.000) [DGKA](http://www.ncbi.nlm.nih.gov/gene/?term=1606)(23.000)[PRKCB](http://www.ncbi.nlm.nih.gov/gene/?term=5579)(23.000)[SEC14L4](http://www.ncbi.nlm.nih.gov/gene/?term=284904)(23.000)[AKT1](http://www.ncbi.nlm.nih.gov/gene/?term=207)(22.373)[CCR7](http://www.ncbi.nlm.nih.gov/gene/?term=1236)(22.373) [ABHD6](http://www.ncbi.nlm.nih.gov/gene/?term=57406)(22.373)[MGLL](http://www.ncbi.nlm.nih.gov/gene/?term=11343)(22.373)[FCER1G](http://www.ncbi.nlm.nih.gov/gene/?term=2207)(22.373)[SUMO1](http://www.ncbi.nlm.nih.gov/gene/?term=7341)(22.373)[GPR55](http://www.ncbi.nlm.nih.gov/gene/?term=9290)(22.373) [CHRNB2](http://www.ncbi.nlm.nih.gov/gene/?term=1141)(22.373)[FCER1A](http://www.ncbi.nlm.nih.gov/gene/?term=2205)(22.373)[CAV3](http://www.ncbi.nlm.nih.gov/gene/?term=859)(22.373)[RNF207](http://www.ncbi.nlm.nih.gov/gene/?term=388591)(22.373)[C3](http://www.ncbi.nlm.nih.gov/gene/?term=718)(22.373) [DAGLA](http://www.ncbi.nlm.nih.gov/gene/?term=747)(22.373)[PLIN5](http://www.ncbi.nlm.nih.gov/gene/?term=440503)(22.373)[ZP3](http://www.ncbi.nlm.nih.gov/gene/?term=7784)(22.373) | |
| [Glycyphyllin](http://www.megabionet.org/tcmid/ingredient/8833/) | This compound doesn't have any potential target with score larger than 20. | |
| [8-Methyl-10-Hydroxylycoctonine](http://www.megabionet.org/tcmid/ingredient/14505/) | [GAA](http://www.ncbi.nlm.nih.gov/gene/?term=2548)(48.000)[MGAM](http://www.ncbi.nlm.nih.gov/gene/?term=8972)(48.000)[UGCG](http://www.ncbi.nlm.nih.gov/gene/?term=7357)(48.000)[GANAB](http://www.ncbi.nlm.nih.gov/gene/?term=23193)(48.000)[GANC](http://www.ncbi.nlm.nih.gov/gene/?term=2595)(48.000) | |
| [Syringin](http://www.megabionet.org/tcmid/ingredient/20553/) | This compound doesn't have any potential target with score larger than 20. | |
| [Isoglycyrol](http://www.megabionet.org/tcmid/ingredient/11441/) | [SEC14L3](http://www.ncbi.nlm.nih.gov/gene/?term=266629)(23.000)[PPP2CA](http://www.ncbi.nlm.nih.gov/gene/?term=5515)(23.000)[PRKCA](http://www.ncbi.nlm.nih.gov/gene/?term=5578)(23.000)[NR1I2](http://www.ncbi.nlm.nih.gov/gene/?term=8856)(23.000)[ALOX5](http://www.ncbi.nlm.nih.gov/gene/?term=240)(23.000) [PPP2CB](http://www.ncbi.nlm.nih.gov/gene/?term=5516)(23.000)[SEC14L2](http://www.ncbi.nlm.nih.gov/gene/?term=23541)(23.000)[DGKA](http://www.ncbi.nlm.nih.gov/gene/?term=1606)(23.000)[PRKCB](http://www.ncbi.nlm.nih.gov/gene/?term=5579)(23.000)[SEC14L4](http://www.ncbi.nlm.nih.gov/gene/?term=284904)(23.000) | |
| [Fructose](http://www.megabionet.org/tcmid/ingredient/7970/) | This compound doesn't have any potential target with score larger than 20. | |
| [Glycyrrhetol](http://www.megabionet.org/tcmid/ingredient/8841/) | [SRD5A1](http://www.ncbi.nlm.nih.gov/gene/?term=6715)(122.778)[CYP17A1](http://www.ncbi.nlm.nih.gov/gene/?term=1586)(122.778)[ESR1](http://www.ncbi.nlm.nih.gov/gene/?term=2099)(122.778)[PGR](http://www.ncbi.nlm.nih.gov/gene/?term=5241)(122.778)[AR](http://www.ncbi.nlm.nih.gov/gene/?term=367)(122.778) [NR3C1](http://www.ncbi.nlm.nih.gov/gene/?term=2908)(122.778)[OPRK1](http://www.ncbi.nlm.nih.gov/gene/?term=4986)(122.778)[ANXA1](http://www.ncbi.nlm.nih.gov/gene/?term=301)(122.778)[NR3C2](http://www.ncbi.nlm.nih.gov/gene/?term=4306)(122.778)[SLC8A1](http://www.ncbi.nlm.nih.gov/gene/?term=6546)(48.000) [F12](http://www.ncbi.nlm.nih.gov/gene/?term=2161)(48.000)[PRLR](http://www.ncbi.nlm.nih.gov/gene/?term=5618)(48.000)[TRPV1](http://www.ncbi.nlm.nih.gov/gene/?term=7442)(48.000)[FADS2](http://www.ncbi.nlm.nih.gov/gene/?term=9415)(48.000)[FADS1](http://www.ncbi.nlm.nih.gov/gene/?term=3992)(48.000) [PTGS1](http://www.ncbi.nlm.nih.gov/gene/?term=5742)(48.000)[PTGS2](http://www.ncbi.nlm.nih.gov/gene/?term=5743)(48.000)[ELOVL4](http://www.ncbi.nlm.nih.gov/gene/?term=6785)(48.000)[HSD11B1](http://www.ncbi.nlm.nih.gov/gene/?term=3290)(48.000)[PDE7B](http://www.ncbi.nlm.nih.gov/gene/?term=27115)(23.000) [PDE4A](http://www.ncbi.nlm.nih.gov/gene/?term=5141)(23.000)[ADORA2A](http://www.ncbi.nlm.nih.gov/gene/?term=135)(23.000)[PDE4D](http://www.ncbi.nlm.nih.gov/gene/?term=5144)(23.000)[PDE7A](http://www.ncbi.nlm.nih.gov/gene/?term=5150)(23.000)[PDE4C](http://www.ncbi.nlm.nih.gov/gene/?term=5143)(23.000) [ATP1A1](http://www.ncbi.nlm.nih.gov/gene/?term=476)(23.000)[PDE4B](http://www.ncbi.nlm.nih.gov/gene/?term=5142)(23.000)[ADORA1](http://www.ncbi.nlm.nih.gov/gene/?term=134)(23.000)[HCAR2](http://www.ncbi.nlm.nih.gov/gene/?term=338442)(22.373)[NOTCH2](http://www.ncbi.nlm.nih.gov/gene/?term=4853)(22.373) [BIN3](http://www.ncbi.nlm.nih.gov/gene/?term=55909)(22.373)[RIPK1](http://www.ncbi.nlm.nih.gov/gene/?term=8737)(22.373)[ZRANB3](http://www.ncbi.nlm.nih.gov/gene/?term=84083)(22.373)[TACR2](http://www.ncbi.nlm.nih.gov/gene/?term=6865)(22.373)[ADA](http://www.ncbi.nlm.nih.gov/gene/?term=100)(22.373) [HAP1](http://www.ncbi.nlm.nih.gov/gene/?term=9001)(22.373) | |
| [Glyzaglabrin](http://www.megabionet.org/tcmid/ingredient/8861/) | This compound doesn't have any potential target with score larger than 20. | |
| [Gancaonin D](http://www.megabionet.org/tcmid/ingredient/8136/) | This compound doesn't have any potential target with score larger than 20. | |
| [Adenine](http://www.megabionet.org/tcmid/ingredient/617/) | [ADORA2A](http://www.ncbi.nlm.nih.gov/gene/?term=135)(122.778)[ADORA2B](http://www.ncbi.nlm.nih.gov/gene/?term=136)(122.778)[ADORA3](http://www.ncbi.nlm.nih.gov/gene/?term=140)(122.778)[ADORA1](http://www.ncbi.nlm.nih.gov/gene/?term=134)(122.778)[FBP1](http://www.ncbi.nlm.nih.gov/gene/?term=2203)(48.000) [PRKAB1](http://www.ncbi.nlm.nih.gov/gene/?term=5564)(48.000)[ACSS2](http://www.ncbi.nlm.nih.gov/gene/?term=55902)(48.000)[POLE](http://www.ncbi.nlm.nih.gov/gene/?term=5426)(48.000)[CBS](http://www.ncbi.nlm.nih.gov/gene/?term=875)(48.000)[POLE3](http://www.ncbi.nlm.nih.gov/gene/?term=54107)(48.000) [ADCY1](http://www.ncbi.nlm.nih.gov/gene/?term=107)(48.000)[COMT](http://www.ncbi.nlm.nih.gov/gene/?term=1312)(48.000)[POLA1](http://www.ncbi.nlm.nih.gov/gene/?term=5422)(48.000)[DCK](http://www.ncbi.nlm.nih.gov/gene/?term=1633)(48.000)[RRM2B](http://www.ncbi.nlm.nih.gov/gene/?term=50484)(48.000) [ADK](http://www.ncbi.nlm.nih.gov/gene/?term=132)(48.000)[GNMT](http://www.ncbi.nlm.nih.gov/gene/?term=27232)(48.000)[PNP](http://www.ncbi.nlm.nih.gov/gene/?term=4860)(48.000)[PDE4D](http://www.ncbi.nlm.nih.gov/gene/?term=5144)(48.000)[CBSL](http://www.ncbi.nlm.nih.gov/gene/?term=102724560)(48.000) [RRM2](http://www.ncbi.nlm.nih.gov/gene/?term=6241)(48.000)[PRKAA1](http://www.ncbi.nlm.nih.gov/gene/?term=5562)(48.000)[MAT1A](http://www.ncbi.nlm.nih.gov/gene/?term=4143)(48.000)[CREB1](http://www.ncbi.nlm.nih.gov/gene/?term=1385)(48.000)[ACSS1](http://www.ncbi.nlm.nih.gov/gene/?term=84532)(48.000) [PIM1](http://www.ncbi.nlm.nih.gov/gene/?term=5292)(48.000)[PYGL](http://www.ncbi.nlm.nih.gov/gene/?term=5836)(48.000)[POLE2](http://www.ncbi.nlm.nih.gov/gene/?term=5427)(48.000)[HINT1](http://www.ncbi.nlm.nih.gov/gene/?term=3094)(48.000)[MAT2A](http://www.ncbi.nlm.nih.gov/gene/?term=4144)(48.000) [AMD1](http://www.ncbi.nlm.nih.gov/gene/?term=262)(48.000)[ACSL1](http://www.ncbi.nlm.nih.gov/gene/?term=2180)(48.000)[PDE4B](http://www.ncbi.nlm.nih.gov/gene/?term=5142)(48.000)[PRKAB2](http://www.ncbi.nlm.nih.gov/gene/?term=5565)(48.000)[POLE4](http://www.ncbi.nlm.nih.gov/gene/?term=56655)(48.000) [RRM1](http://www.ncbi.nlm.nih.gov/gene/?term=6240)(48.000)[MTAP](http://www.ncbi.nlm.nih.gov/gene/?term=4507)(22.373)[PRPS1](http://www.ncbi.nlm.nih.gov/gene/?term=5631)(22.373) | |
| [4'-Hydroxywogonin](http://www.megabionet.org/tcmid/ingredient/10828/) | This compound doesn't have any potential target with score larger than 20. | |
| [Octadecane](http://www.megabionet.org/tcmid/ingredient/23828/) | This compound doesn't have any potential target with score larger than 20. | |
| [Docosane](http://www.megabionet.org/tcmid/ingredient/6533/) | This compound doesn't have any potential target with score larger than 20. | |
| [Sucrose](http://www.megabionet.org/tcmid/ingredient/20430/) | [CXCR4](http://www.ncbi.nlm.nih.gov/gene/?term=7852)(48.000)[NFKB2](http://www.ncbi.nlm.nih.gov/gene/?term=4791)(48.000)[TNF](http://www.ncbi.nlm.nih.gov/gene/?term=7124)(48.000)[MMP9](http://www.ncbi.nlm.nih.gov/gene/?term=4318)(48.000)[IFNG](http://www.ncbi.nlm.nih.gov/gene/?term=3458)(48.000) | |
| [11-Hydroxyrankinidine](http://www.megabionet.org/tcmid/ingredient/10670/) | This compound doesn't have any potential target with score larger than 20. | |
| [Alpha-Trihydroxy Coprostanic Acid](http://www.megabionet.org/tcmid/ingredient/32116/) | [ESRRG](http://www.ncbi.nlm.nih.gov/gene/?term=2104)(686.000)[COX6C](http://www.ncbi.nlm.nih.gov/gene/?term=1345)(686.000)[COX5B](http://www.ncbi.nlm.nih.gov/gene/?term=1329)(686.000)[COX7C](http://www.ncbi.nlm.nih.gov/gene/?term=1350)(686.000)[COX1](http://www.ncbi.nlm.nih.gov/gene/?term=4512)(686.000) [AKR1C2](http://www.ncbi.nlm.nih.gov/gene/?term=1646)(686.000)[COX5A](http://www.ncbi.nlm.nih.gov/gene/?term=9377)(686.000)[COX3](http://www.ncbi.nlm.nih.gov/gene/?term=4514)(686.000)[COX7A1](http://www.ncbi.nlm.nih.gov/gene/?term=1346)(686.000)[FECH](http://www.ncbi.nlm.nih.gov/gene/?term=2235)(686.000) [COX4I1](http://www.ncbi.nlm.nih.gov/gene/?term=1327)(686.000)[PLA2G1B](http://www.ncbi.nlm.nih.gov/gene/?term=5319)(686.000)[COX6A2](http://www.ncbi.nlm.nih.gov/gene/?term=1339)(686.000)[ADH1C](http://www.ncbi.nlm.nih.gov/gene/?term=126)(686.000)[COX6B1](http://www.ncbi.nlm.nih.gov/gene/?term=1340)(686.000) [FABP6](http://www.ncbi.nlm.nih.gov/gene/?term=2172)(686.000)[CES1](http://www.ncbi.nlm.nih.gov/gene/?term=1066)(686.000)[COX7B](http://www.ncbi.nlm.nih.gov/gene/?term=1349)(686.000)[COX2](http://www.ncbi.nlm.nih.gov/gene/?term=4513)(686.000)[COX8A](http://www.ncbi.nlm.nih.gov/gene/?term=1351)(686.000) [NR1H4](http://www.ncbi.nlm.nih.gov/gene/?term=9971)(686.000)[AKR1D1](http://www.ncbi.nlm.nih.gov/gene/?term=6718)(122.778)[AR](http://www.ncbi.nlm.nih.gov/gene/?term=367)(122.778)[TYR](http://www.ncbi.nlm.nih.gov/gene/?term=7299)(122.778)[SRD5A2](http://www.ncbi.nlm.nih.gov/gene/?term=6716)(122.778) [ADH1A](http://www.ncbi.nlm.nih.gov/gene/?term=124)(80.882)[ADH1B](http://www.ncbi.nlm.nih.gov/gene/?term=125)(80.882)[AKR1C1](http://www.ncbi.nlm.nih.gov/gene/?term=1645)(80.882)[ATCAY](http://www.ncbi.nlm.nih.gov/gene/?term=85300)(55.444)[EIF2AK1](http://www.ncbi.nlm.nih.gov/gene/?term=27102)(55.444) [ABCB4](http://www.ncbi.nlm.nih.gov/gene/?term=5244)(55.444)[ALAD](http://www.ncbi.nlm.nih.gov/gene/?term=210)(55.444)[SCN11A](http://www.ncbi.nlm.nih.gov/gene/?term=11280)(48.000)[SCN2B](http://www.ncbi.nlm.nih.gov/gene/?term=6327)(48.000)[CACNA2D1](http://www.ncbi.nlm.nih.gov/gene/?term=781)(48.000) [PLAT](http://www.ncbi.nlm.nih.gov/gene/?term=5327)(48.000)[ABAT](http://www.ncbi.nlm.nih.gov/gene/?term=18)(48.000)[SCN1A](http://www.ncbi.nlm.nih.gov/gene/?term=6323)(48.000)[SCN3B](http://www.ncbi.nlm.nih.gov/gene/?term=55800)(48.000)[SCN3A](http://www.ncbi.nlm.nih.gov/gene/?term=6328)(48.000) [CACNA1A](http://www.ncbi.nlm.nih.gov/gene/?term=773)(48.000)[SCN7A](http://www.ncbi.nlm.nih.gov/gene/?term=6332)(48.000)[GRIN3B](http://www.ncbi.nlm.nih.gov/gene/?term=116444)(48.000)[CACNA2D2](http://www.ncbi.nlm.nih.gov/gene/?term=9254)(48.000)[GRIN2A](http://www.ncbi.nlm.nih.gov/gene/?term=2903)(48.000) [SCN5A](http://www.ncbi.nlm.nih.gov/gene/?term=6331)(48.000)[SCN10A](http://www.ncbi.nlm.nih.gov/gene/?term=6336)(48.000)[ALDH5A1](http://www.ncbi.nlm.nih.gov/gene/?term=7915)(48.000)[TOP1](http://www.ncbi.nlm.nih.gov/gene/?term=7150)(48.000)[SCN2A](http://www.ncbi.nlm.nih.gov/gene/?term=6326)(48.000) [PLG](http://www.ncbi.nlm.nih.gov/gene/?term=5340)(48.000)[HDAC9](http://www.ncbi.nlm.nih.gov/gene/?term=9734)(48.000)[CACNA1B](http://www.ncbi.nlm.nih.gov/gene/?term=774)(48.000)[SCN9A](http://www.ncbi.nlm.nih.gov/gene/?term=6335)(48.000)[GRIN2C](http://www.ncbi.nlm.nih.gov/gene/?term=2905)(48.000) [GRIN2B](http://www.ncbi.nlm.nih.gov/gene/?term=2904)(48.000)[SCN4A](http://www.ncbi.nlm.nih.gov/gene/?term=6329)(48.000)[SCN4B](http://www.ncbi.nlm.nih.gov/gene/?term=6330)(48.000)[GRIN3A](http://www.ncbi.nlm.nih.gov/gene/?term=116443)(48.000)[ACADSB](http://www.ncbi.nlm.nih.gov/gene/?term=36)(48.000) [SCN8A](http://www.ncbi.nlm.nih.gov/gene/?term=6334)(48.000)[SCN1B](http://www.ncbi.nlm.nih.gov/gene/?term=6324)(48.000)[GRIN2D](http://www.ncbi.nlm.nih.gov/gene/?term=2906)(48.000)[ADORA1](http://www.ncbi.nlm.nih.gov/gene/?term=134)(48.000)[OGDH](http://www.ncbi.nlm.nih.gov/gene/?term=4967)(48.000) [HDAC2](http://www.ncbi.nlm.nih.gov/gene/?term=3066)(48.000)[GRIN1](http://www.ncbi.nlm.nih.gov/gene/?term=2902)(48.000)[AKR1C3](http://www.ncbi.nlm.nih.gov/gene/?term=8644)(23.000)[AKR1C4](http://www.ncbi.nlm.nih.gov/gene/?term=1109)(23.000)[ACO1](http://www.ncbi.nlm.nih.gov/gene/?term=48)(22.373) [CES2](http://www.ncbi.nlm.nih.gov/gene/?term=8824)(22.373)[GPBAR1](http://www.ncbi.nlm.nih.gov/gene/?term=151306)(22.373)[ANG](http://www.ncbi.nlm.nih.gov/gene/?term=283)(22.373)[SIRT4](http://www.ncbi.nlm.nih.gov/gene/?term=23409)(22.373)[FGF19](http://www.ncbi.nlm.nih.gov/gene/?term=9965)(22.373) [KLF4](http://www.ncbi.nlm.nih.gov/gene/?term=9314)(22.373)[COMT](http://www.ncbi.nlm.nih.gov/gene/?term=1312)(22.373)[NR0B1](http://www.ncbi.nlm.nih.gov/gene/?term=190)(22.373)[IREB2](http://www.ncbi.nlm.nih.gov/gene/?term=3658)(22.373)[PROX1](http://www.ncbi.nlm.nih.gov/gene/?term=5629)(22.373) [PRMT1](http://www.ncbi.nlm.nih.gov/gene/?term=3276)(22.373)[PPARGC1B](http://www.ncbi.nlm.nih.gov/gene/?term=133522)(22.373)[BCL2](http://www.ncbi.nlm.nih.gov/gene/?term=596)(22.373)[BAX](http://www.ncbi.nlm.nih.gov/gene/?term=581)(22.373)[PARK7](http://www.ncbi.nlm.nih.gov/gene/?term=11315)(22.373) [NMUR2](http://www.ncbi.nlm.nih.gov/gene/?term=56923)(22.373)[GATA3](http://www.ncbi.nlm.nih.gov/gene/?term=2625)(22.373)[SLC7A7](http://www.ncbi.nlm.nih.gov/gene/?term=9056)(22.373)[DHDH](http://www.ncbi.nlm.nih.gov/gene/?term=27294)(22.373)[HPN](http://www.ncbi.nlm.nih.gov/gene/?term=3249)(22.373) [PTGFR](http://www.ncbi.nlm.nih.gov/gene/?term=5737)(22.373)[ATP2B4](http://www.ncbi.nlm.nih.gov/gene/?term=493)(22.373)[LDB1](http://www.ncbi.nlm.nih.gov/gene/?term=8861)(22.373)[PAX8](http://www.ncbi.nlm.nih.gov/gene/?term=7849)(22.373)[AGTR1](http://www.ncbi.nlm.nih.gov/gene/?term=185)(22.373) [CES5A](http://www.ncbi.nlm.nih.gov/gene/?term=221223)(22.373)[LIPA](http://www.ncbi.nlm.nih.gov/gene/?term=3988)(22.373)[VDR](http://www.ncbi.nlm.nih.gov/gene/?term=7421)(22.373)[EGFR](http://www.ncbi.nlm.nih.gov/gene/?term=1956)(22.373)[CES3](http://www.ncbi.nlm.nih.gov/gene/?term=23491)(22.373) [PTGDR](http://www.ncbi.nlm.nih.gov/gene/?term=5729)(22.373)[ESD](http://www.ncbi.nlm.nih.gov/gene/?term=2098)(22.373) | |
| [Mannose](http://www.megabionet.org/tcmid/ingredient/13500/) | [TOP1](http://www.ncbi.nlm.nih.gov/gene/?term=7150)(122.778)[ESRRG](http://www.ncbi.nlm.nih.gov/gene/?term=2104)(48.000)[COX6C](http://www.ncbi.nlm.nih.gov/gene/?term=1345)(48.000)[COX5B](http://www.ncbi.nlm.nih.gov/gene/?term=1329)(48.000)[COX7C](http://www.ncbi.nlm.nih.gov/gene/?term=1350)(48.000) [COX1](http://www.ncbi.nlm.nih.gov/gene/?term=4512)(48.000)[AKR1C2](http://www.ncbi.nlm.nih.gov/gene/?term=1646)(48.000)[AKR1D1](http://www.ncbi.nlm.nih.gov/gene/?term=6718)(48.000)[COX5A](http://www.ncbi.nlm.nih.gov/gene/?term=9377)(48.000)[COX3](http://www.ncbi.nlm.nih.gov/gene/?term=4514)(48.000) [AR](http://www.ncbi.nlm.nih.gov/gene/?term=367)(48.000)[COX7A1](http://www.ncbi.nlm.nih.gov/gene/?term=1346)(48.000)[FECH](http://www.ncbi.nlm.nih.gov/gene/?term=2235)(48.000)[COX4I1](http://www.ncbi.nlm.nih.gov/gene/?term=1327)(48.000)[PLA2G1B](http://www.ncbi.nlm.nih.gov/gene/?term=5319)(48.000) [COX6A2](http://www.ncbi.nlm.nih.gov/gene/?term=1339)(48.000)[TYR](http://www.ncbi.nlm.nih.gov/gene/?term=7299)(48.000)[ADH1C](http://www.ncbi.nlm.nih.gov/gene/?term=126)(48.000)[COX6B1](http://www.ncbi.nlm.nih.gov/gene/?term=1340)(48.000)[FABP6](http://www.ncbi.nlm.nih.gov/gene/?term=2172)(48.000) [CES1](http://www.ncbi.nlm.nih.gov/gene/?term=1066)(48.000)[SRD5A2](http://www.ncbi.nlm.nih.gov/gene/?term=6716)(48.000)[COX7B](http://www.ncbi.nlm.nih.gov/gene/?term=1349)(48.000)[COX2](http://www.ncbi.nlm.nih.gov/gene/?term=4513)(48.000)[COX8A](http://www.ncbi.nlm.nih.gov/gene/?term=1351)(48.000) [NR1H4](http://www.ncbi.nlm.nih.gov/gene/?term=9971)(48.000)[RENBP](http://www.ncbi.nlm.nih.gov/gene/?term=5973)(23.000)[NAGLU](http://www.ncbi.nlm.nih.gov/gene/?term=4669)(23.000)[NAGK](http://www.ncbi.nlm.nih.gov/gene/?term=55577)(23.000)[B4GALT3](http://www.ncbi.nlm.nih.gov/gene/?term=8703)(23.000) [B4GALT2](http://www.ncbi.nlm.nih.gov/gene/?term=8704)(23.000)[B4GALT1](http://www.ncbi.nlm.nih.gov/gene/?term=2683)(23.000)[NAGPA](http://www.ncbi.nlm.nih.gov/gene/?term=51172)(23.000)[B4GALT4](http://www.ncbi.nlm.nih.gov/gene/?term=8702)(23.000)[GNE](http://www.ncbi.nlm.nih.gov/gene/?term=10020)(22.373) [DEFB126](http://www.ncbi.nlm.nih.gov/gene/?term=81623)(22.373) | |
| [Methionine](http://www.megabionet.org/tcmid/ingredient/13816/) | [BHMT2](http://www.ncbi.nlm.nih.gov/gene/?term=23743)(686.000)[MTRR](http://www.ncbi.nlm.nih.gov/gene/?term=4552)(686.000)[METAP2](http://www.ncbi.nlm.nih.gov/gene/?term=10988)(686.000)[MTR](http://www.ncbi.nlm.nih.gov/gene/?term=4548)(686.000)[BHMT](http://www.ncbi.nlm.nih.gov/gene/?term=635)(686.000) [GSTP1](http://www.ncbi.nlm.nih.gov/gene/?term=2950)(80.882)[TRAF2](http://www.ncbi.nlm.nih.gov/gene/?term=7186)(55.444)[CBS](http://www.ncbi.nlm.nih.gov/gene/?term=875)(55.444)[COMT](http://www.ncbi.nlm.nih.gov/gene/?term=1312)(55.444)[SLC1A5](http://www.ncbi.nlm.nih.gov/gene/?term=6510)(23.000) [GOT2](http://www.ncbi.nlm.nih.gov/gene/?term=2806)(23.000)[GPT2](http://www.ncbi.nlm.nih.gov/gene/?term=84706)(23.000)[BAAT](http://www.ncbi.nlm.nih.gov/gene/?term=570)(23.000)[OAZ3](http://www.ncbi.nlm.nih.gov/gene/?term=51686)(23.000)[SHMT1](http://www.ncbi.nlm.nih.gov/gene/?term=6470)(23.000) [ABAT](http://www.ncbi.nlm.nih.gov/gene/?term=18)(23.000)[SLC1A1](http://www.ncbi.nlm.nih.gov/gene/?term=6505)(23.000)[SLC7A2](http://www.ncbi.nlm.nih.gov/gene/?term=6542)(23.000)[GCSH](http://www.ncbi.nlm.nih.gov/gene/?term=2653)(23.000)[SLC7A8](http://www.ncbi.nlm.nih.gov/gene/?term=23428)(23.000) [PPAT](http://www.ncbi.nlm.nih.gov/gene/?term=5471)(23.000)[ACY3](http://www.ncbi.nlm.nih.gov/gene/?term=91703)(23.000)[ASPA](http://www.ncbi.nlm.nih.gov/gene/?term=443)(23.000)[IARS2](http://www.ncbi.nlm.nih.gov/gene/?term=55699)(23.000)[GLRA2](http://www.ncbi.nlm.nih.gov/gene/?term=2742)(23.000) [ALAS1](http://www.ncbi.nlm.nih.gov/gene/?term=211)(23.000)[BCAT1](http://www.ncbi.nlm.nih.gov/gene/?term=586)(23.000)[GCAT](http://www.ncbi.nlm.nih.gov/gene/?term=23464)(23.000)[SLC38A3](http://www.ncbi.nlm.nih.gov/gene/?term=10991)(23.000)[VARS](http://www.ncbi.nlm.nih.gov/gene/?term=7407)(23.000) [NFS1](http://www.ncbi.nlm.nih.gov/gene/?term=9054)(23.000)[SLC25A15](http://www.ncbi.nlm.nih.gov/gene/?term=10166)(23.000)[SLC1A4](http://www.ncbi.nlm.nih.gov/gene/?term=6509)(23.000)[ADSSL1](http://www.ncbi.nlm.nih.gov/gene/?term=122622)(23.000)[VDAC3](http://www.ncbi.nlm.nih.gov/gene/?term=7419)(23.000) [GSS](http://www.ncbi.nlm.nih.gov/gene/?term=2937)(23.000)[ASNS](http://www.ncbi.nlm.nih.gov/gene/?term=440)(23.000)[GLRA3](http://www.ncbi.nlm.nih.gov/gene/?term=8001)(23.000)[DARS2](http://www.ncbi.nlm.nih.gov/gene/?term=55157)(23.000)[GRIN3B](http://www.ncbi.nlm.nih.gov/gene/?term=116444)(23.000) [LCMT1](http://www.ncbi.nlm.nih.gov/gene/?term=51451)(23.000)[GRIN2A](http://www.ncbi.nlm.nih.gov/gene/?term=2903)(23.000)[GOT1](http://www.ncbi.nlm.nih.gov/gene/?term=2805)(23.000)[GNMT](http://www.ncbi.nlm.nih.gov/gene/?term=27232)(23.000)[GLRB](http://www.ncbi.nlm.nih.gov/gene/?term=2743)(23.000) [GLYAT](http://www.ncbi.nlm.nih.gov/gene/?term=10249)(23.000)[AARS2](http://www.ncbi.nlm.nih.gov/gene/?term=57505)(23.000)[ASPH](http://www.ncbi.nlm.nih.gov/gene/?term=444)(23.000)[GPR18](http://www.ncbi.nlm.nih.gov/gene/?term=2841)(23.000)[CTPS1](http://www.ncbi.nlm.nih.gov/gene/?term=1503)(23.000) [SLC25A12](http://www.ncbi.nlm.nih.gov/gene/?term=8604)(23.000)[PHYKPL](http://www.ncbi.nlm.nih.gov/gene/?term=85007)(23.000)[GLYATL1](http://www.ncbi.nlm.nih.gov/gene/?term=92292)(23.000)[GATM](http://www.ncbi.nlm.nih.gov/gene/?term=2628)(23.000)[LARS](http://www.ncbi.nlm.nih.gov/gene/?term=51520)(23.000) [ARG1](http://www.ncbi.nlm.nih.gov/gene/?term=383)(23.000)[NARS2](http://www.ncbi.nlm.nih.gov/gene/?term=79731)(23.000)[TNNC1](http://www.ncbi.nlm.nih.gov/gene/?term=7134)(23.000)[DARS](http://www.ncbi.nlm.nih.gov/gene/?term=1615)(23.000)[GRIN2C](http://www.ncbi.nlm.nih.gov/gene/?term=2905)(23.000) [OAT](http://www.ncbi.nlm.nih.gov/gene/?term=4942)(23.000)[GLDC](http://www.ncbi.nlm.nih.gov/gene/?term=2731)(23.000)[VDAC2](http://www.ncbi.nlm.nih.gov/gene/?term=7417)(23.000)[OAZ2](http://www.ncbi.nlm.nih.gov/gene/?term=4947)(23.000)[PIPOX](http://www.ncbi.nlm.nih.gov/gene/?term=51268)(23.000) [SLC25A2](http://www.ncbi.nlm.nih.gov/gene/?term=83884)(23.000)[GARS](http://www.ncbi.nlm.nih.gov/gene/?term=2617)(23.000)[GPT](http://www.ncbi.nlm.nih.gov/gene/?term=2875)(23.000)[AARS](http://www.ncbi.nlm.nih.gov/gene/?term=16)(23.000)[SHMT2](http://www.ncbi.nlm.nih.gov/gene/?term=6472)(23.000) [ALAS2](http://www.ncbi.nlm.nih.gov/gene/?term=212)(23.000)[VDAC1](http://www.ncbi.nlm.nih.gov/gene/?term=7416)(23.000)[SLC6A9](http://www.ncbi.nlm.nih.gov/gene/?term=6536)(23.000)[SLC7A3](http://www.ncbi.nlm.nih.gov/gene/?term=84889)(23.000)[ASRGL1](http://www.ncbi.nlm.nih.gov/gene/?term=80150)(23.000) [GLYATL2](http://www.ncbi.nlm.nih.gov/gene/?term=219970)(23.000)[SLC32A1](http://www.ncbi.nlm.nih.gov/gene/?term=140679)(23.000)[OAZ1](http://www.ncbi.nlm.nih.gov/gene/?term=4946)(23.000)[KARS](http://www.ncbi.nlm.nih.gov/gene/?term=3735)(23.000)[AGXT](http://www.ncbi.nlm.nih.gov/gene/?term=189)(23.000) [LARS2](http://www.ncbi.nlm.nih.gov/gene/?term=23395)(23.000)[RNASE1](http://www.ncbi.nlm.nih.gov/gene/?term=6035)(23.000)[ASS1](http://www.ncbi.nlm.nih.gov/gene/?term=445)(23.000)[ADSS](http://www.ncbi.nlm.nih.gov/gene/?term=159)(23.000)[SLC7A1](http://www.ncbi.nlm.nih.gov/gene/?term=6541)(23.000) [SLC36A1](http://www.ncbi.nlm.nih.gov/gene/?term=206358)(23.000)[ACADSB](http://www.ncbi.nlm.nih.gov/gene/?term=36)(23.000)[SLC6A5](http://www.ncbi.nlm.nih.gov/gene/?term=9152)(23.000)[SLC7A4](http://www.ncbi.nlm.nih.gov/gene/?term=6545)(23.000)[CAD](http://www.ncbi.nlm.nih.gov/gene/?term=790)(23.000) [PCCB](http://www.ncbi.nlm.nih.gov/gene/?term=5096)(23.000)[OTC](http://www.ncbi.nlm.nih.gov/gene/?term=5009)(23.000)[GLUL](http://www.ncbi.nlm.nih.gov/gene/?term=2752)(23.000)[AGXT2](http://www.ncbi.nlm.nih.gov/gene/?term=64902)(23.000)[ARG2](http://www.ncbi.nlm.nih.gov/gene/?term=384)(23.000) [IARS](http://www.ncbi.nlm.nih.gov/gene/?term=3376)(23.000)[BCAT2](http://www.ncbi.nlm.nih.gov/gene/?term=587)(23.000)[ACY1](http://www.ncbi.nlm.nih.gov/gene/?term=95)(23.000)[KYNU](http://www.ncbi.nlm.nih.gov/gene/?term=8942)(23.000)[SLC25A13](http://www.ncbi.nlm.nih.gov/gene/?term=10165)(23.000) [DPEP1](http://www.ncbi.nlm.nih.gov/gene/?term=1800)(23.000)[GLRA1](http://www.ncbi.nlm.nih.gov/gene/?term=2741)(23.000)[LYZ](http://www.ncbi.nlm.nih.gov/gene/?term=4069)(23.000)[LCMT2](http://www.ncbi.nlm.nih.gov/gene/?term=9836)(23.000)[PAICS](http://www.ncbi.nlm.nih.gov/gene/?term=10606)(23.000) [NARS](http://www.ncbi.nlm.nih.gov/gene/?term=4677)(23.000)[SPAG9](http://www.ncbi.nlm.nih.gov/gene/?term=9043)(22.373)[APIP](http://www.ncbi.nlm.nih.gov/gene/?term=51074)(22.373)[SAMD9L](http://www.ncbi.nlm.nih.gov/gene/?term=219285)(22.373)[DMGDH](http://www.ncbi.nlm.nih.gov/gene/?term=29958)(22.373) [PGGT1B](http://www.ncbi.nlm.nih.gov/gene/?term=5229)(22.373)[EDN1](http://www.ncbi.nlm.nih.gov/gene/?term=1906)(22.373)[ADNP](http://www.ncbi.nlm.nih.gov/gene/?term=23394)(22.373)[UNCX](http://www.ncbi.nlm.nih.gov/gene/?term=340260)(22.373)[MAPK8IP3](http://www.ncbi.nlm.nih.gov/gene/?term=23162)(22.373) [POR](http://www.ncbi.nlm.nih.gov/gene/?term=5447)(22.373)[NOS2](http://www.ncbi.nlm.nih.gov/gene/?term=4843)(22.373)[AHCYL2](http://www.ncbi.nlm.nih.gov/gene/?term=23382)(22.373)[VIMP](http://www.ncbi.nlm.nih.gov/gene/?term=55829)(22.373)[APOD](http://www.ncbi.nlm.nih.gov/gene/?term=347)(22.373) [CPQ](http://www.ncbi.nlm.nih.gov/gene/?term=10404)(22.373)[MRI1](http://www.ncbi.nlm.nih.gov/gene/?term=84245)(22.373)[MAD2L2](http://www.ncbi.nlm.nih.gov/gene/?term=10459)(22.373)[NDOR1](http://www.ncbi.nlm.nih.gov/gene/?term=27158)(22.373)[NR4A3](http://www.ncbi.nlm.nih.gov/gene/?term=8013)(22.373) [ACADL](http://www.ncbi.nlm.nih.gov/gene/?term=33)(22.373)[CAV1](http://www.ncbi.nlm.nih.gov/gene/?term=857)(22.373)[ALLC](http://www.ncbi.nlm.nih.gov/gene/?term=55821)(22.373)[AHCYL1](http://www.ncbi.nlm.nih.gov/gene/?term=10768)(22.373)[C1QTNF3](http://www.ncbi.nlm.nih.gov/gene/?term=114899)(22.373) [AHCY](http://www.ncbi.nlm.nih.gov/gene/?term=191)(22.373)[PTK2](http://www.ncbi.nlm.nih.gov/gene/?term=5747)(22.373)[NOS3](http://www.ncbi.nlm.nih.gov/gene/?term=4846)(22.373)[FLT3](http://www.ncbi.nlm.nih.gov/gene/?term=2322)(22.373)[NOS1](http://www.ncbi.nlm.nih.gov/gene/?term=4842)(22.373) | |
| [Glisoflavanone](http://www.megabionet.org/tcmid/ingredient/8552/) | [SOAT1](http://www.ncbi.nlm.nih.gov/gene/?term=6646)(23.000)[MTTP](http://www.ncbi.nlm.nih.gov/gene/?term=4547)(23.000)[CNR2](http://www.ncbi.nlm.nih.gov/gene/?term=1269)(23.000)[CNR1](http://www.ncbi.nlm.nih.gov/gene/?term=1268)(23.000)[SOAT2](http://www.ncbi.nlm.nih.gov/gene/?term=8435)(23.000) [DRD2](http://www.ncbi.nlm.nih.gov/gene/?term=1813)(22.373)[PAWR](http://www.ncbi.nlm.nih.gov/gene/?term=5074)(22.373) | |
| [Menthyl Acetate](http://www.megabionet.org/tcmid/ingredient/13772/) | [NPR1](http://www.ncbi.nlm.nih.gov/gene/?term=4881)(80.882)[AR](http://www.ncbi.nlm.nih.gov/gene/?term=367)(48.000)[NPR3](http://www.ncbi.nlm.nih.gov/gene/?term=4883)(22.373)[NPR2](http://www.ncbi.nlm.nih.gov/gene/?term=4882)(22.373) | |
| [Astragaloside Vi](http://www.megabionet.org/tcmid/ingredient/1941/) | [CXCR4](http://www.ncbi.nlm.nih.gov/gene/?term=7852)(48.000) | |
| [Scutellarein-5-Galactoside](http://www.megabionet.org/tcmid/ingredient/19569/) | This compound doesn't have any potential target with score larger than 20. | |
| [Narcissin](http://www.megabionet.org/tcmid/ingredient/15257/) | This compound doesn't have any potential target with score larger than 20. | |
| [Isolicoflavonol](http://www.megabionet.org/tcmid/ingredient/11487/) | This compound doesn't have any potential target with score larger than 20. | |  |
| [13-Methyl Pentadecanoic Acid](http://www.megabionet.org/tcmid/ingredient/30339/) | [AKR1D1](http://www.ncbi.nlm.nih.gov/gene/?term=6718)(686.000)[TYR](http://www.ncbi.nlm.nih.gov/gene/?term=7299)(686.000)[SRD5A2](http://www.ncbi.nlm.nih.gov/gene/?term=6716)(686.000)[SCN11A](http://www.ncbi.nlm.nih.gov/gene/?term=11280)(122.778)[SCN2B](http://www.ncbi.nlm.nih.gov/gene/?term=6327)(122.778) [ABAT](http://www.ncbi.nlm.nih.gov/gene/?term=18)(122.778)[SCN1A](http://www.ncbi.nlm.nih.gov/gene/?term=6323)(122.778)[ESRRG](http://www.ncbi.nlm.nih.gov/gene/?term=2104)(122.778)[SCN3B](http://www.ncbi.nlm.nih.gov/gene/?term=55800)(122.778)[COX6C](http://www.ncbi.nlm.nih.gov/gene/?term=1345)(122.778) [COX5B](http://www.ncbi.nlm.nih.gov/gene/?term=1329)(122.778)[COX7C](http://www.ncbi.nlm.nih.gov/gene/?term=1350)(122.778)[COX1](http://www.ncbi.nlm.nih.gov/gene/?term=4512)(122.778)[SCN3A](http://www.ncbi.nlm.nih.gov/gene/?term=6328)(122.778)[AKR1C2](http://www.ncbi.nlm.nih.gov/gene/?term=1646)(122.778) [SCN7A](http://www.ncbi.nlm.nih.gov/gene/?term=6332)(122.778)[SCN5A](http://www.ncbi.nlm.nih.gov/gene/?term=6331)(122.778)[SCN10A](http://www.ncbi.nlm.nih.gov/gene/?term=6336)(122.778)[ALDH5A1](http://www.ncbi.nlm.nih.gov/gene/?term=7915)(122.778)[SCN2A](http://www.ncbi.nlm.nih.gov/gene/?term=6326)(122.778) [COX5A](http://www.ncbi.nlm.nih.gov/gene/?term=9377)(122.778)[COX3](http://www.ncbi.nlm.nih.gov/gene/?term=4514)(122.778)[AR](http://www.ncbi.nlm.nih.gov/gene/?term=367)(122.778)[COX7A1](http://www.ncbi.nlm.nih.gov/gene/?term=1346)(122.778)[FECH](http://www.ncbi.nlm.nih.gov/gene/?term=2235)(122.778) [COX4I1](http://www.ncbi.nlm.nih.gov/gene/?term=1327)(122.778)[HDAC9](http://www.ncbi.nlm.nih.gov/gene/?term=9734)(122.778)[PLA2G1B](http://www.ncbi.nlm.nih.gov/gene/?term=5319)(122.778)[COX6A2](http://www.ncbi.nlm.nih.gov/gene/?term=1339)(122.778)[SCN9A](http://www.ncbi.nlm.nih.gov/gene/?term=6335)(122.778) [SCN4A](http://www.ncbi.nlm.nih.gov/gene/?term=6329)(122.778)[SCN4B](http://www.ncbi.nlm.nih.gov/gene/?term=6330)(122.778)[ADH1C](http://www.ncbi.nlm.nih.gov/gene/?term=126)(122.778)[COX6B1](http://www.ncbi.nlm.nih.gov/gene/?term=1340)(122.778)[FABP6](http://www.ncbi.nlm.nih.gov/gene/?term=2172)(122.778) [CES1](http://www.ncbi.nlm.nih.gov/gene/?term=1066)(122.778)[ACADSB](http://www.ncbi.nlm.nih.gov/gene/?term=36)(122.778)[SCN8A](http://www.ncbi.nlm.nih.gov/gene/?term=6334)(122.778)[COX7B](http://www.ncbi.nlm.nih.gov/gene/?term=1349)(122.778)[SCN1B](http://www.ncbi.nlm.nih.gov/gene/?term=6324)(122.778) [COX2](http://www.ncbi.nlm.nih.gov/gene/?term=4513)(122.778)[OGDH](http://www.ncbi.nlm.nih.gov/gene/?term=4967)(122.778)[HDAC2](http://www.ncbi.nlm.nih.gov/gene/?term=3066)(122.778)[COX8A](http://www.ncbi.nlm.nih.gov/gene/?term=1351)(122.778)[NR1H4](http://www.ncbi.nlm.nih.gov/gene/?term=9971)(122.778) [SUCLG2](http://www.ncbi.nlm.nih.gov/gene/?term=8801)(80.882)[SLC13A2](http://www.ncbi.nlm.nih.gov/gene/?term=9058)(80.882)[SLC13A1](http://www.ncbi.nlm.nih.gov/gene/?term=6561)(80.882)[SUCLG1](http://www.ncbi.nlm.nih.gov/gene/?term=8802)(80.882)[HSD17B6](http://www.ncbi.nlm.nih.gov/gene/?term=8630)(80.882) [SLC25A10](http://www.ncbi.nlm.nih.gov/gene/?term=1468)(80.882)[P3H3](http://www.ncbi.nlm.nih.gov/gene/?term=10536)(80.882)[OXCT2](http://www.ncbi.nlm.nih.gov/gene/?term=64064)(80.882)[TMLHE](http://www.ncbi.nlm.nih.gov/gene/?term=55217)(80.882)[PLOD1](http://www.ncbi.nlm.nih.gov/gene/?term=5351)(80.882) [SUCNR1](http://www.ncbi.nlm.nih.gov/gene/?term=56670)(80.882)[SLC13A3](http://www.ncbi.nlm.nih.gov/gene/?term=64849)(80.882)[ASPH](http://www.ncbi.nlm.nih.gov/gene/?term=444)(80.882)[BBOX1](http://www.ncbi.nlm.nih.gov/gene/?term=8424)(80.882)[SDHB](http://www.ncbi.nlm.nih.gov/gene/?term=6390)(80.882) [OXCT1](http://www.ncbi.nlm.nih.gov/gene/?term=5019)(80.882)[PLOD3](http://www.ncbi.nlm.nih.gov/gene/?term=8985)(80.882)[P4HA1](http://www.ncbi.nlm.nih.gov/gene/?term=5033)(80.882)[SDHC](http://www.ncbi.nlm.nih.gov/gene/?term=6391)(80.882)[P3H2](http://www.ncbi.nlm.nih.gov/gene/?term=55214)(80.882) [SDHA](http://www.ncbi.nlm.nih.gov/gene/?term=6389)(80.882)[P4HA2](http://www.ncbi.nlm.nih.gov/gene/?term=8974)(80.882)[SUCLA2](http://www.ncbi.nlm.nih.gov/gene/?term=8803)(80.882)[SDHD](http://www.ncbi.nlm.nih.gov/gene/?term=6392)(80.882)[P3H1](http://www.ncbi.nlm.nih.gov/gene/?term=64175)(80.882) [DCT](http://www.ncbi.nlm.nih.gov/gene/?term=1638)(55.444)[AKR1C3](http://www.ncbi.nlm.nih.gov/gene/?term=8644)(55.444)[CRTAP](http://www.ncbi.nlm.nih.gov/gene/?term=10491)(55.444)[SLIT2](http://www.ncbi.nlm.nih.gov/gene/?term=9353)(55.444)[ACO2](http://www.ncbi.nlm.nih.gov/gene/?term=50)(55.444) [JMJD6](http://www.ncbi.nlm.nih.gov/gene/?term=23210)(55.444)[SLC13A4](http://www.ncbi.nlm.nih.gov/gene/?term=26266)(55.444)[SDHAF2](http://www.ncbi.nlm.nih.gov/gene/?term=54949)(55.444)[SLC1A3](http://www.ncbi.nlm.nih.gov/gene/?term=6507)(55.444)[SALL1](http://www.ncbi.nlm.nih.gov/gene/?term=6299)(55.444) [HSD17B11](http://www.ncbi.nlm.nih.gov/gene/?term=51170)(55.444)[TYRP1](http://www.ncbi.nlm.nih.gov/gene/?term=7306)(55.444)[HIF1AN](http://www.ncbi.nlm.nih.gov/gene/?term=55662)(55.444)[UROS](http://www.ncbi.nlm.nih.gov/gene/?term=7390)(55.444)[SLC13A5](http://www.ncbi.nlm.nih.gov/gene/?term=284111)(55.444) [P4HB](http://www.ncbi.nlm.nih.gov/gene/?term=5034)(55.444)[CACNA2D1](http://www.ncbi.nlm.nih.gov/gene/?term=781)(48.000)[PLAT](http://www.ncbi.nlm.nih.gov/gene/?term=5327)(48.000)[SLC7A2](http://www.ncbi.nlm.nih.gov/gene/?term=6542)(48.000)[CACNA1A](http://www.ncbi.nlm.nih.gov/gene/?term=773)(48.000) [GRIN3B](http://www.ncbi.nlm.nih.gov/gene/?term=116444)(48.000)[CACNA2D2](http://www.ncbi.nlm.nih.gov/gene/?term=9254)(48.000)[GRIN2A](http://www.ncbi.nlm.nih.gov/gene/?term=2903)(48.000)[TOP1](http://www.ncbi.nlm.nih.gov/gene/?term=7150)(48.000)[PLG](http://www.ncbi.nlm.nih.gov/gene/?term=5340)(48.000) [CACNA1B](http://www.ncbi.nlm.nih.gov/gene/?term=774)(48.000)[GRIN2C](http://www.ncbi.nlm.nih.gov/gene/?term=2905)(48.000)[GRIN2B](http://www.ncbi.nlm.nih.gov/gene/?term=2904)(48.000)[SLC7A3](http://www.ncbi.nlm.nih.gov/gene/?term=84889)(48.000)[KARS](http://www.ncbi.nlm.nih.gov/gene/?term=3735)(48.000) [GRIN3A](http://www.ncbi.nlm.nih.gov/gene/?term=116443)(48.000)[SLC7A1](http://www.ncbi.nlm.nih.gov/gene/?term=6541)(48.000)[SLC7A4](http://www.ncbi.nlm.nih.gov/gene/?term=6545)(48.000)[GRIN2D](http://www.ncbi.nlm.nih.gov/gene/?term=2906)(48.000)[ADORA1](http://www.ncbi.nlm.nih.gov/gene/?term=134)(48.000) [GRIN1](http://www.ncbi.nlm.nih.gov/gene/?term=2902)(48.000)[HDAC1](http://www.ncbi.nlm.nih.gov/gene/?term=3065)(23.000)[GPD1L](http://www.ncbi.nlm.nih.gov/gene/?term=23171)(22.373)[SRD5A1](http://www.ncbi.nlm.nih.gov/gene/?term=6715)(22.373)[FBXO45](http://www.ncbi.nlm.nih.gov/gene/?term=200933)(22.373) [SIRT1](http://www.ncbi.nlm.nih.gov/gene/?term=23411)(22.373)[SHMT1](http://www.ncbi.nlm.nih.gov/gene/?term=6470)(22.373)[SUGCT](http://www.ncbi.nlm.nih.gov/gene/?term=79783)(22.373)[ACSS2](http://www.ncbi.nlm.nih.gov/gene/?term=55902)(22.373)[ARV1](http://www.ncbi.nlm.nih.gov/gene/?term=64801)(22.373) [SIX1](http://www.ncbi.nlm.nih.gov/gene/?term=6495)(22.373)[BDH1](http://www.ncbi.nlm.nih.gov/gene/?term=622)(22.373)[COLGALT2](http://www.ncbi.nlm.nih.gov/gene/?term=23127)(22.373)[SIX4](http://www.ncbi.nlm.nih.gov/gene/?term=51804)(22.373)[MRPS36](http://www.ncbi.nlm.nih.gov/gene/?term=92259)(22.373) [OSBPL8](http://www.ncbi.nlm.nih.gov/gene/?term=114882)(22.373)[SPI1](http://www.ncbi.nlm.nih.gov/gene/?term=6688)(22.373)[SUOX](http://www.ncbi.nlm.nih.gov/gene/?term=6821)(22.373)[CCL2](http://www.ncbi.nlm.nih.gov/gene/?term=6347)(22.373)[BCKDK](http://www.ncbi.nlm.nih.gov/gene/?term=10295)(22.373) [UBE2B](http://www.ncbi.nlm.nih.gov/gene/?term=7320)(22.373)[CYP1A2](http://www.ncbi.nlm.nih.gov/gene/?term=1544)(22.373)[CCL5](http://www.ncbi.nlm.nih.gov/gene/?term=6352)(22.373)[ANK2](http://www.ncbi.nlm.nih.gov/gene/?term=287)(22.373)[HSD17B2](http://www.ncbi.nlm.nih.gov/gene/?term=3294)(22.373) [STIM2](http://www.ncbi.nlm.nih.gov/gene/?term=57620)(22.373)[PKD2](http://www.ncbi.nlm.nih.gov/gene/?term=5311)(22.373)[ALDH9A1](http://www.ncbi.nlm.nih.gov/gene/?term=223)(22.373)[TH](http://www.ncbi.nlm.nih.gov/gene/?term=7054)(22.373)[EPO](http://www.ncbi.nlm.nih.gov/gene/?term=2056)(22.373) [COLGALT1](http://www.ncbi.nlm.nih.gov/gene/?term=79709)(22.373)[NEDD4](http://www.ncbi.nlm.nih.gov/gene/?term=4734)(22.373)[CYP11A1](http://www.ncbi.nlm.nih.gov/gene/?term=1583)(22.373)[DHODH](http://www.ncbi.nlm.nih.gov/gene/?term=1723)(22.373)[CLN3](http://www.ncbi.nlm.nih.gov/gene/?term=1201)(22.373) [ARID1A](http://www.ncbi.nlm.nih.gov/gene/?term=8289)(22.373)[EGLN2](http://www.ncbi.nlm.nih.gov/gene/?term=112398)(22.373)[ACAT1](http://www.ncbi.nlm.nih.gov/gene/?term=38)(22.373)[GALC](http://www.ncbi.nlm.nih.gov/gene/?term=2581)(22.373)[PLCG2](http://www.ncbi.nlm.nih.gov/gene/?term=5336)(22.373) [ACOT4](http://www.ncbi.nlm.nih.gov/gene/?term=122970)(22.373)[PHGDH](http://www.ncbi.nlm.nih.gov/gene/?term=26227)(22.373)[PNLIPRP2](http://www.ncbi.nlm.nih.gov/gene/?term=5408)(22.373)[CYP39A1](http://www.ncbi.nlm.nih.gov/gene/?term=51302)(22.373)[BCKDHA](http://www.ncbi.nlm.nih.gov/gene/?term=593)(22.373) [SLC25A12](http://www.ncbi.nlm.nih.gov/gene/?term=8604)(22.373)[YBX3](http://www.ncbi.nlm.nih.gov/gene/?term=8531)(22.373)[8-Mar](http://www.ncbi.nlm.nih.gov/gene/?term=220972)(22.373)[STIM1](http://www.ncbi.nlm.nih.gov/gene/?term=6786)(22.373)[GAL3ST1](http://www.ncbi.nlm.nih.gov/gene/?term=9514)(22.373) [CDH11](http://www.ncbi.nlm.nih.gov/gene/?term=1009)(22.373)[CACNA1D](http://www.ncbi.nlm.nih.gov/gene/?term=776)(22.373)[DDC](http://www.ncbi.nlm.nih.gov/gene/?term=1644)(22.373)[DHTKD1](http://www.ncbi.nlm.nih.gov/gene/?term=55526)(22.373)[NEDD4L](http://www.ncbi.nlm.nih.gov/gene/?term=23327)(22.373) [BDKRB2](http://www.ncbi.nlm.nih.gov/gene/?term=624)(22.373)[PRPH](http://www.ncbi.nlm.nih.gov/gene/?term=5630)(22.373)[BCKDHB](http://www.ncbi.nlm.nih.gov/gene/?term=594)(22.373)[KCNE5](http://www.ncbi.nlm.nih.gov/gene/?term=23630)(22.373)[SRD5A3](http://www.ncbi.nlm.nih.gov/gene/?term=79644)(22.373) [SMAD7](http://www.ncbi.nlm.nih.gov/gene/?term=4092)(22.373)[PRDM8](http://www.ncbi.nlm.nih.gov/gene/?term=56978)(22.373)[UROD](http://www.ncbi.nlm.nih.gov/gene/?term=7389)(22.373)[UGT8](http://www.ncbi.nlm.nih.gov/gene/?term=7368)(22.373)[ETHE1](http://www.ncbi.nlm.nih.gov/gene/?term=23474)(22.373) [WNT10B](http://www.ncbi.nlm.nih.gov/gene/?term=7480)(22.373)[OGFOD1](http://www.ncbi.nlm.nih.gov/gene/?term=55239)(22.373)[SLC38A7](http://www.ncbi.nlm.nih.gov/gene/?term=55238)(22.373)[ACSS1](http://www.ncbi.nlm.nih.gov/gene/?term=84532)(22.373)[CYP4B1](http://www.ncbi.nlm.nih.gov/gene/?term=1580)(22.373) [STAR](http://www.ncbi.nlm.nih.gov/gene/?term=6770)(22.373)[DLST](http://www.ncbi.nlm.nih.gov/gene/?term=1743)(22.373)[PF4](http://www.ncbi.nlm.nih.gov/gene/?term=5196)(22.373)[CRACR2A](http://www.ncbi.nlm.nih.gov/gene/?term=84766)(22.373)[HSD17B1](http://www.ncbi.nlm.nih.gov/gene/?term=3292)(22.373) [CAMK2D](http://www.ncbi.nlm.nih.gov/gene/?term=817)(22.373)[AP3D1](http://www.ncbi.nlm.nih.gov/gene/?term=8943)(22.373)[MYO5A](http://www.ncbi.nlm.nih.gov/gene/?term=4644)(22.373)[ILVBL](http://www.ncbi.nlm.nih.gov/gene/?term=10994)(22.373)[CAV3](http://www.ncbi.nlm.nih.gov/gene/?term=859)(22.373) [HTT](http://www.ncbi.nlm.nih.gov/gene/?term=3064)(22.373)[IL10](http://www.ncbi.nlm.nih.gov/gene/?term=3586)(22.373)[EGLN3](http://www.ncbi.nlm.nih.gov/gene/?term=112399)(22.373)[OGDHL](http://www.ncbi.nlm.nih.gov/gene/?term=55753)(22.373)[KCNA5](http://www.ncbi.nlm.nih.gov/gene/?term=3741)(22.373) [TST](http://www.ncbi.nlm.nih.gov/gene/?term=7263)(22.373)[SLC1A6](http://www.ncbi.nlm.nih.gov/gene/?term=6511)(22.373)[NPPA](http://www.ncbi.nlm.nih.gov/gene/?term=4878)(22.373)[ACADM](http://www.ncbi.nlm.nih.gov/gene/?term=34)(22.373)[SQRDL](http://www.ncbi.nlm.nih.gov/gene/?term=58472)(22.373) [TMEM110](http://www.ncbi.nlm.nih.gov/gene/?term=375346)(22.373)[CDC42](http://www.ncbi.nlm.nih.gov/gene/?term=998)(22.373)[ARHGEF2](http://www.ncbi.nlm.nih.gov/gene/?term=9181)(22.373)[EGLN1](http://www.ncbi.nlm.nih.gov/gene/?term=54583)(22.373)[ETFDH](http://www.ncbi.nlm.nih.gov/gene/?term=2110)(22.373) [EPHA4](http://www.ncbi.nlm.nih.gov/gene/?term=2043)(22.373)[OCA2](http://www.ncbi.nlm.nih.gov/gene/?term=4948)(22.373)[HSD17B8](http://www.ncbi.nlm.nih.gov/gene/?term=7923)(22.373)[CTNNB1](http://www.ncbi.nlm.nih.gov/gene/?term=1499)(22.373)[P4HA3](http://www.ncbi.nlm.nih.gov/gene/?term=283208)(22.373) [EDA](http://www.ncbi.nlm.nih.gov/gene/?term=1896)(22.373)[HACL1](http://www.ncbi.nlm.nih.gov/gene/?term=26061)(22.373)[RYR2](http://www.ncbi.nlm.nih.gov/gene/?term=6262)(22.373)[HAP1](http://www.ncbi.nlm.nih.gov/gene/?term=9001)(22.373)[PCSK9](http://www.ncbi.nlm.nih.gov/gene/?term=255738)(22.373) [KCNQ1](http://www.ncbi.nlm.nih.gov/gene/?term=3784)(22.373)[CASQ2](http://www.ncbi.nlm.nih.gov/gene/?term=845)(22.373)[GJA5](http://www.ncbi.nlm.nih.gov/gene/?term=2702)(22.373)[DHRS9](http://www.ncbi.nlm.nih.gov/gene/?term=10170)(22.373)[GPR143](http://www.ncbi.nlm.nih.gov/gene/?term=4935)(22.373) [UGT1A1](http://www.ncbi.nlm.nih.gov/gene/?term=54658)(22.373)[DLD](http://www.ncbi.nlm.nih.gov/gene/?term=1738)(22.373)[GNAS](http://www.ncbi.nlm.nih.gov/gene/?term=2778)(22.373)[SLC25A13](http://www.ncbi.nlm.nih.gov/gene/?term=10165)(22.373)[SFXN5](http://www.ncbi.nlm.nih.gov/gene/?term=94097)(22.373) [PLOD2](http://www.ncbi.nlm.nih.gov/gene/?term=5352)(22.373)[CYP1A1](http://www.ncbi.nlm.nih.gov/gene/?term=1543)(22.373)[SULT2A1](http://www.ncbi.nlm.nih.gov/gene/?term=6822)(22.373)[NFX1](http://www.ncbi.nlm.nih.gov/gene/?term=4799)(22.373)[DBT](http://www.ncbi.nlm.nih.gov/gene/?term=1629)(22.373) [SLC25A1](http://www.ncbi.nlm.nih.gov/gene/?term=6576)(22.373) | |  |
| [Isoquercitrin](http://www.megabionet.org/tcmid/ingredient/11639/) | This compound doesn't have any potential target with score larger than 20. | |  |
| [Isoononin](http://www.megabionet.org/tcmid/ingredient/11573/) | This compound doesn't have any potential target with score larger than 20. | |  |
| [Urea](http://www.megabionet.org/tcmid/ingredient/22230/) | [MTNR1A](http://www.ncbi.nlm.nih.gov/gene/?term=4543)(80.882)[MTNR1B](http://www.ncbi.nlm.nih.gov/gene/?term=4544)(80.882)[HTR2C](http://www.ncbi.nlm.nih.gov/gene/?term=3358)(80.882)[HTR2A](http://www.ncbi.nlm.nih.gov/gene/?term=3356)(55.444)[TNFSF11](http://www.ncbi.nlm.nih.gov/gene/?term=8600)(22.373) [HPCA](http://www.ncbi.nlm.nih.gov/gene/?term=3208)(22.373)[AVPR1A](http://www.ncbi.nlm.nih.gov/gene/?term=552)(22.373)[HTR2B](http://www.ncbi.nlm.nih.gov/gene/?term=3357)(22.373)[NPY2R](http://www.ncbi.nlm.nih.gov/gene/?term=4887)(22.373)[APLN](http://www.ncbi.nlm.nih.gov/gene/?term=8862)(22.373) [GHRL](http://www.ncbi.nlm.nih.gov/gene/?term=51738)(22.373)[AVP](http://www.ncbi.nlm.nih.gov/gene/?term=551)(22.373)[IFNG](http://www.ncbi.nlm.nih.gov/gene/?term=3458)(22.373)[HCRT](http://www.ncbi.nlm.nih.gov/gene/?term=3060)(22.373)[IL6](http://www.ncbi.nlm.nih.gov/gene/?term=3569)(22.373) [PDGFA](http://www.ncbi.nlm.nih.gov/gene/?term=5154)(22.373)[PDZD3](http://www.ncbi.nlm.nih.gov/gene/?term=79849)(22.373)[TNR](http://www.ncbi.nlm.nih.gov/gene/?term=7143)(22.373)[ZP3](http://www.ncbi.nlm.nih.gov/gene/?term=7784)(22.373)[GPR50](http://www.ncbi.nlm.nih.gov/gene/?term=9248)(22.373) [PAWR](http://www.ncbi.nlm.nih.gov/gene/?term=5074)(22.373)[CHRNB4](http://www.ncbi.nlm.nih.gov/gene/?term=1143)(22.373)[PDGFB](http://www.ncbi.nlm.nih.gov/gene/?term=5155)(22.373)[CARTPT](http://www.ncbi.nlm.nih.gov/gene/?term=9607)(22.373)[GLRA1](http://www.ncbi.nlm.nih.gov/gene/?term=2741)(22.373) | |  |
| [Astragaloside Ii](http://www.megabionet.org/tcmid/ingredient/1937/) | [SLC47A1](http://www.ncbi.nlm.nih.gov/gene/?term=55244)(48.000)[CYP3A4](http://www.ncbi.nlm.nih.gov/gene/?term=1576)(48.000)[CYP51A1](http://www.ncbi.nlm.nih.gov/gene/?term=1595)(48.000)[KCNH2](http://www.ncbi.nlm.nih.gov/gene/?term=3757)(48.000)[ATP1A1](http://www.ncbi.nlm.nih.gov/gene/?term=476)(48.000) [MLNR](http://www.ncbi.nlm.nih.gov/gene/?term=2862)(48.000)[SLCO1B3](http://www.ncbi.nlm.nih.gov/gene/?term=28234)(48.000)[ALB](http://www.ncbi.nlm.nih.gov/gene/?term=213)(48.000)[ABCB1](http://www.ncbi.nlm.nih.gov/gene/?term=5243)(48.000)[SLCO1B1](http://www.ncbi.nlm.nih.gov/gene/?term=10599)(48.000) | |  |
| [Inulin](http://www.megabionet.org/tcmid/ingredient/23243/) | [CXCR4](http://www.ncbi.nlm.nih.gov/gene/?term=7852)(48.000) | |  |
| [3-Hydroxyglabrol](http://www.megabionet.org/tcmid/ingredient/10148/) | [SOAT1](http://www.ncbi.nlm.nih.gov/gene/?term=6646)(48.000)[MTTP](http://www.ncbi.nlm.nih.gov/gene/?term=4547)(48.000)[SOAT2](http://www.ncbi.nlm.nih.gov/gene/?term=8435)(48.000) | |  |
| [Glycyrrhizin](http://www.megabionet.org/tcmid/ingredient/23172/) | [NR3C1](http://www.ncbi.nlm.nih.gov/gene/?term=2908)(48.000)[ATP1A1](http://www.ncbi.nlm.nih.gov/gene/?term=476)(48.000) | |  |
| [Scutellarin](http://www.megabionet.org/tcmid/ingredient/19572/) | This compound doesn't have any potential target with score larger than 20. | |  |
| [Encecalin](http://www.megabionet.org/tcmid/ingredient/6795/) | [ADRA1A](http://www.ncbi.nlm.nih.gov/gene/?term=148)(80.882)[ADRA2A](http://www.ncbi.nlm.nih.gov/gene/?term=150)(80.882)[KCNH6](http://www.ncbi.nlm.nih.gov/gene/?term=81033)(80.882)[ADRA1D](http://www.ncbi.nlm.nih.gov/gene/?term=146)(80.882)[KCNH2](http://www.ncbi.nlm.nih.gov/gene/?term=3757)(80.882) [ADRA1B](http://www.ncbi.nlm.nih.gov/gene/?term=147)(80.882)[KCNH7](http://www.ncbi.nlm.nih.gov/gene/?term=90134)(80.882)[ADRA2B](http://www.ncbi.nlm.nih.gov/gene/?term=151)(80.882)[PHKG2](http://www.ncbi.nlm.nih.gov/gene/?term=5261)(55.444)[APLP1](http://www.ncbi.nlm.nih.gov/gene/?term=333)(55.444) [YWHAE](http://www.ncbi.nlm.nih.gov/gene/?term=7531)(55.444)[CHRNA7](http://www.ncbi.nlm.nih.gov/gene/?term=1139)(55.444)[INS](http://www.ncbi.nlm.nih.gov/gene/?term=3630)(55.444)[ADRA2C](http://www.ncbi.nlm.nih.gov/gene/?term=152)(55.444)[NPPA](http://www.ncbi.nlm.nih.gov/gene/?term=4878)(55.444) [CALCA](http://www.ncbi.nlm.nih.gov/gene/?term=796)(55.444)[NFKB1](http://www.ncbi.nlm.nih.gov/gene/?term=4790)(23.000)[PDE3A](http://www.ncbi.nlm.nih.gov/gene/?term=5139)(23.000)[CYSLTR1](http://www.ncbi.nlm.nih.gov/gene/?term=10800)(23.000)[TNF](http://www.ncbi.nlm.nih.gov/gene/?term=7124)(23.000) [RNASE3](http://www.ncbi.nlm.nih.gov/gene/?term=6037)(23.000)[MUC2](http://www.ncbi.nlm.nih.gov/gene/?term=4583)(23.000)[IL5](http://www.ncbi.nlm.nih.gov/gene/?term=3567)(23.000)[NOS1AP](http://www.ncbi.nlm.nih.gov/gene/?term=9722)(22.373)[NAV2](http://www.ncbi.nlm.nih.gov/gene/?term=89797)(22.373) [DNAJA1](http://www.ncbi.nlm.nih.gov/gene/?term=3301)(22.373)[ADRB2](http://www.ncbi.nlm.nih.gov/gene/?term=154)(22.373)[GSK3A](http://www.ncbi.nlm.nih.gov/gene/?term=2931)(22.373)[CETN2](http://www.ncbi.nlm.nih.gov/gene/?term=1069)(22.373)[HSPA1B](http://www.ncbi.nlm.nih.gov/gene/?term=3304)(22.373) [CETN1](http://www.ncbi.nlm.nih.gov/gene/?term=1068)(22.373)[TRPV1](http://www.ncbi.nlm.nih.gov/gene/?term=7442)(22.373)[TRIB1](http://www.ncbi.nlm.nih.gov/gene/?term=10221)(22.373)[ARRB2](http://www.ncbi.nlm.nih.gov/gene/?term=409)(22.373)[ADRB1](http://www.ncbi.nlm.nih.gov/gene/?term=153)(22.373) [PDE4D](http://www.ncbi.nlm.nih.gov/gene/?term=5144)(22.373)[SREBF1](http://www.ncbi.nlm.nih.gov/gene/?term=6720)(22.373)[SDCBP](http://www.ncbi.nlm.nih.gov/gene/?term=6386)(22.373)[ARRB1](http://www.ncbi.nlm.nih.gov/gene/?term=408)(22.373)[NCOA3](http://www.ncbi.nlm.nih.gov/gene/?term=8202)(22.373) [HSPA1A](http://www.ncbi.nlm.nih.gov/gene/?term=3303)(22.373)[DRD5](http://www.ncbi.nlm.nih.gov/gene/?term=1816)(22.373)[ATP2B4](http://www.ncbi.nlm.nih.gov/gene/?term=493)(22.373)[ARRDC3](http://www.ncbi.nlm.nih.gov/gene/?term=57561)(22.373)[LTA](http://www.ncbi.nlm.nih.gov/gene/?term=4049)(22.373) [KCNE2](http://www.ncbi.nlm.nih.gov/gene/?term=9992)(22.373)[ADORA1](http://www.ncbi.nlm.nih.gov/gene/?term=134)(22.373)[ADAM17](http://www.ncbi.nlm.nih.gov/gene/?term=6868)(22.373)[PINK1](http://www.ncbi.nlm.nih.gov/gene/?term=65018)(22.373)[ADRB3](http://www.ncbi.nlm.nih.gov/gene/?term=155)(22.373) [NOS1](http://www.ncbi.nlm.nih.gov/gene/?term=4842)(22.373)[KCNQ1](http://www.ncbi.nlm.nih.gov/gene/?term=3784)(22.373)[RGS2](http://www.ncbi.nlm.nih.gov/gene/?term=5997)(22.373)[TRPM8](http://www.ncbi.nlm.nih.gov/gene/?term=79054)(22.373)[HSPA8](http://www.ncbi.nlm.nih.gov/gene/?term=3312)(22.373) [RXRA](http://www.ncbi.nlm.nih.gov/gene/?term=6256)(22.373)[TPM1](http://www.ncbi.nlm.nih.gov/gene/?term=7168)(22.373)[EDN2](http://www.ncbi.nlm.nih.gov/gene/?term=1907)(22.373)[KCNE1](http://www.ncbi.nlm.nih.gov/gene/?term=3753)(22.373)[TRPA1](http://www.ncbi.nlm.nih.gov/gene/?term=8989)(22.373) | |  |
| [Formononetin](http://www.megabionet.org/tcmid/ingredient/7882/) | This compound doesn't have any potential target with score larger than 20. | |  |
| [Oleanolic Acid-28-O-Beta-D-Glucopyranoside](http://www.megabionet.org/tcmid/ingredient/31751/) | [GLRA3](http://www.ncbi.nlm.nih.gov/gene/?term=8001)(48.000)[NR3C1](http://www.ncbi.nlm.nih.gov/gene/?term=2908)(48.000)[ATP1A1](http://www.ncbi.nlm.nih.gov/gene/?term=476)(48.000)[GABRB3](http://www.ncbi.nlm.nih.gov/gene/?term=2562)(48.000) | |  |
| [Isogosferol](http://www.megabionet.org/tcmid/ingredient/11442/) | This compound doesn't have any potential target with score larger than 20. | |  |
| [Licoricesaponine G2](http://www.megabionet.org/tcmid/ingredient/31436/) | [NR3C1](http://www.ncbi.nlm.nih.gov/gene/?term=2908)(48.000)[ATP1A1](http://www.ncbi.nlm.nih.gov/gene/?term=476)(48.000) | |  |
| [Caprylic Acid](http://www.megabionet.org/tcmid/ingredient/23099/) | [SCN11A](http://www.ncbi.nlm.nih.gov/gene/?term=11280)(122.778)[SCN2B](http://www.ncbi.nlm.nih.gov/gene/?term=6327)(122.778)[ABAT](http://www.ncbi.nlm.nih.gov/gene/?term=18)(122.778)[SCN1A](http://www.ncbi.nlm.nih.gov/gene/?term=6323)(122.778)[ESRRG](http://www.ncbi.nlm.nih.gov/gene/?term=2104)(122.778) [SCN3B](http://www.ncbi.nlm.nih.gov/gene/?term=55800)(122.778)[COX6C](http://www.ncbi.nlm.nih.gov/gene/?term=1345)(122.778)[COX5B](http://www.ncbi.nlm.nih.gov/gene/?term=1329)(122.778)[COX7C](http://www.ncbi.nlm.nih.gov/gene/?term=1350)(122.778)[COX1](http://www.ncbi.nlm.nih.gov/gene/?term=4512)(122.778) [SCN3A](http://www.ncbi.nlm.nih.gov/gene/?term=6328)(122.778)[AKR1C2](http://www.ncbi.nlm.nih.gov/gene/?term=1646)(122.778)[SCN7A](http://www.ncbi.nlm.nih.gov/gene/?term=6332)(122.778)[SCN5A](http://www.ncbi.nlm.nih.gov/gene/?term=6331)(122.778)[SCN10A](http://www.ncbi.nlm.nih.gov/gene/?term=6336)(122.778) [AKR1D1](http://www.ncbi.nlm.nih.gov/gene/?term=6718)(122.778)[ALDH5A1](http://www.ncbi.nlm.nih.gov/gene/?term=7915)(122.778)[SCN2A](http://www.ncbi.nlm.nih.gov/gene/?term=6326)(122.778)[COX5A](http://www.ncbi.nlm.nih.gov/gene/?term=9377)(122.778)[COX3](http://www.ncbi.nlm.nih.gov/gene/?term=4514)(122.778) [AR](http://www.ncbi.nlm.nih.gov/gene/?term=367)(122.778)[COX7A1](http://www.ncbi.nlm.nih.gov/gene/?term=1346)(122.778)[FECH](http://www.ncbi.nlm.nih.gov/gene/?term=2235)(122.778)[COX4I1](http://www.ncbi.nlm.nih.gov/gene/?term=1327)(122.778)[HDAC9](http://www.ncbi.nlm.nih.gov/gene/?term=9734)(122.778) [PLA2G1B](http://www.ncbi.nlm.nih.gov/gene/?term=5319)(122.778)[COX6A2](http://www.ncbi.nlm.nih.gov/gene/?term=1339)(122.778)[TYR](http://www.ncbi.nlm.nih.gov/gene/?term=7299)(122.778)[SCN9A](http://www.ncbi.nlm.nih.gov/gene/?term=6335)(122.778)[SCN4A](http://www.ncbi.nlm.nih.gov/gene/?term=6329)(122.778) [SCN4B](http://www.ncbi.nlm.nih.gov/gene/?term=6330)(122.778)[ADH1C](http://www.ncbi.nlm.nih.gov/gene/?term=126)(122.778)[COX6B1](http://www.ncbi.nlm.nih.gov/gene/?term=1340)(122.778)[FABP6](http://www.ncbi.nlm.nih.gov/gene/?term=2172)(122.778)[CES1](http://www.ncbi.nlm.nih.gov/gene/?term=1066)(122.778) [SRD5A2](http://www.ncbi.nlm.nih.gov/gene/?term=6716)(122.778)[ACADSB](http://www.ncbi.nlm.nih.gov/gene/?term=36)(122.778)[SCN8A](http://www.ncbi.nlm.nih.gov/gene/?term=6334)(122.778)[COX7B](http://www.ncbi.nlm.nih.gov/gene/?term=1349)(122.778)[SCN1B](http://www.ncbi.nlm.nih.gov/gene/?term=6324)(122.778) [COX2](http://www.ncbi.nlm.nih.gov/gene/?term=4513)(122.778)[OGDH](http://www.ncbi.nlm.nih.gov/gene/?term=4967)(122.778)[HDAC2](http://www.ncbi.nlm.nih.gov/gene/?term=3066)(122.778)[COX8A](http://www.ncbi.nlm.nih.gov/gene/?term=1351)(122.778)[NR1H4](http://www.ncbi.nlm.nih.gov/gene/?term=9971)(122.778) [SUCLG2](http://www.ncbi.nlm.nih.gov/gene/?term=8801)(80.882)[SLC13A2](http://www.ncbi.nlm.nih.gov/gene/?term=9058)(80.882)[SLC13A1](http://www.ncbi.nlm.nih.gov/gene/?term=6561)(80.882)[SUCLG1](http://www.ncbi.nlm.nih.gov/gene/?term=8802)(80.882)[HSD17B6](http://www.ncbi.nlm.nih.gov/gene/?term=8630)(80.882) [SLC25A10](http://www.ncbi.nlm.nih.gov/gene/?term=1468)(80.882)[P3H3](http://www.ncbi.nlm.nih.gov/gene/?term=10536)(80.882)[OXCT2](http://www.ncbi.nlm.nih.gov/gene/?term=64064)(80.882)[TMLHE](http://www.ncbi.nlm.nih.gov/gene/?term=55217)(80.882)[PLOD1](http://www.ncbi.nlm.nih.gov/gene/?term=5351)(80.882) [SUCNR1](http://www.ncbi.nlm.nih.gov/gene/?term=56670)(80.882)[SLC13A3](http://www.ncbi.nlm.nih.gov/gene/?term=64849)(80.882)[ASPH](http://www.ncbi.nlm.nih.gov/gene/?term=444)(80.882)[BBOX1](http://www.ncbi.nlm.nih.gov/gene/?term=8424)(80.882)[SDHB](http://www.ncbi.nlm.nih.gov/gene/?term=6390)(80.882) [OXCT1](http://www.ncbi.nlm.nih.gov/gene/?term=5019)(80.882)[PLOD3](http://www.ncbi.nlm.nih.gov/gene/?term=8985)(80.882)[P4HA1](http://www.ncbi.nlm.nih.gov/gene/?term=5033)(80.882)[SDHC](http://www.ncbi.nlm.nih.gov/gene/?term=6391)(80.882)[P3H2](http://www.ncbi.nlm.nih.gov/gene/?term=55214)(80.882) [SDHA](http://www.ncbi.nlm.nih.gov/gene/?term=6389)(80.882)[P4HA2](http://www.ncbi.nlm.nih.gov/gene/?term=8974)(80.882)[SUCLA2](http://www.ncbi.nlm.nih.gov/gene/?term=8803)(80.882)[SDHD](http://www.ncbi.nlm.nih.gov/gene/?term=6392)(80.882)[P3H1](http://www.ncbi.nlm.nih.gov/gene/?term=64175)(80.882) [DCT](http://www.ncbi.nlm.nih.gov/gene/?term=1638)(55.444)[AKR1C3](http://www.ncbi.nlm.nih.gov/gene/?term=8644)(55.444)[CRTAP](http://www.ncbi.nlm.nih.gov/gene/?term=10491)(55.444)[SLIT2](http://www.ncbi.nlm.nih.gov/gene/?term=9353)(55.444)[ACO2](http://www.ncbi.nlm.nih.gov/gene/?term=50)(55.444) [JMJD6](http://www.ncbi.nlm.nih.gov/gene/?term=23210)(55.444)[SLC13A4](http://www.ncbi.nlm.nih.gov/gene/?term=26266)(55.444)[SDHAF2](http://www.ncbi.nlm.nih.gov/gene/?term=54949)(55.444)[SLC1A3](http://www.ncbi.nlm.nih.gov/gene/?term=6507)(55.444)[SALL1](http://www.ncbi.nlm.nih.gov/gene/?term=6299)(55.444) [HSD17B11](http://www.ncbi.nlm.nih.gov/gene/?term=51170)(55.444)[TYRP1](http://www.ncbi.nlm.nih.gov/gene/?term=7306)(55.444)[HIF1AN](http://www.ncbi.nlm.nih.gov/gene/?term=55662)(55.444)[UROS](http://www.ncbi.nlm.nih.gov/gene/?term=7390)(55.444)[SLC13A5](http://www.ncbi.nlm.nih.gov/gene/?term=284111)(55.444) [P4HB](http://www.ncbi.nlm.nih.gov/gene/?term=5034)(55.444)[CACNA2D1](http://www.ncbi.nlm.nih.gov/gene/?term=781)(48.000)[PLAT](http://www.ncbi.nlm.nih.gov/gene/?term=5327)(48.000)[SLC7A2](http://www.ncbi.nlm.nih.gov/gene/?term=6542)(48.000)[CACNA1A](http://www.ncbi.nlm.nih.gov/gene/?term=773)(48.000) [GRIN3B](http://www.ncbi.nlm.nih.gov/gene/?term=116444)(48.000)[CACNA2D2](http://www.ncbi.nlm.nih.gov/gene/?term=9254)(48.000)[GRIN2A](http://www.ncbi.nlm.nih.gov/gene/?term=2903)(48.000)[TOP1](http://www.ncbi.nlm.nih.gov/gene/?term=7150)(48.000)[PLG](http://www.ncbi.nlm.nih.gov/gene/?term=5340)(48.000) [CACNA1B](http://www.ncbi.nlm.nih.gov/gene/?term=774)(48.000)[GRIN2C](http://www.ncbi.nlm.nih.gov/gene/?term=2905)(48.000)[GRIN2B](http://www.ncbi.nlm.nih.gov/gene/?term=2904)(48.000)[SLC7A3](http://www.ncbi.nlm.nih.gov/gene/?term=84889)(48.000)[KARS](http://www.ncbi.nlm.nih.gov/gene/?term=3735)(48.000) [GRIN3A](http://www.ncbi.nlm.nih.gov/gene/?term=116443)(48.000)[SLC7A1](http://www.ncbi.nlm.nih.gov/gene/?term=6541)(48.000)[SLC7A4](http://www.ncbi.nlm.nih.gov/gene/?term=6545)(48.000)[GRIN2D](http://www.ncbi.nlm.nih.gov/gene/?term=2906)(48.000)[ADORA1](http://www.ncbi.nlm.nih.gov/gene/?term=134)(48.000) [GRIN1](http://www.ncbi.nlm.nih.gov/gene/?term=2902)(48.000)[HDAC1](http://www.ncbi.nlm.nih.gov/gene/?term=3065)(23.000)[GPD1L](http://www.ncbi.nlm.nih.gov/gene/?term=23171)(22.373)[SRD5A1](http://www.ncbi.nlm.nih.gov/gene/?term=6715)(22.373)[FBXO45](http://www.ncbi.nlm.nih.gov/gene/?term=200933)(22.373) [SIRT1](http://www.ncbi.nlm.nih.gov/gene/?term=23411)(22.373)[SHMT1](http://www.ncbi.nlm.nih.gov/gene/?term=6470)(22.373)[SUGCT](http://www.ncbi.nlm.nih.gov/gene/?term=79783)(22.373)[ACSS2](http://www.ncbi.nlm.nih.gov/gene/?term=55902)(22.373)[ARV1](http://www.ncbi.nlm.nih.gov/gene/?term=64801)(22.373) [SIX1](http://www.ncbi.nlm.nih.gov/gene/?term=6495)(22.373)[BDH1](http://www.ncbi.nlm.nih.gov/gene/?term=622)(22.373)[COLGALT2](http://www.ncbi.nlm.nih.gov/gene/?term=23127)(22.373)[SIX4](http://www.ncbi.nlm.nih.gov/gene/?term=51804)(22.373)[MRPS36](http://www.ncbi.nlm.nih.gov/gene/?term=92259)(22.373) [OSBPL8](http://www.ncbi.nlm.nih.gov/gene/?term=114882)(22.373)[SPI1](http://www.ncbi.nlm.nih.gov/gene/?term=6688)(22.373)[SUOX](http://www.ncbi.nlm.nih.gov/gene/?term=6821)(22.373)[CCL2](http://www.ncbi.nlm.nih.gov/gene/?term=6347)(22.373)[BCKDK](http://www.ncbi.nlm.nih.gov/gene/?term=10295)(22.373) [UBE2B](http://www.ncbi.nlm.nih.gov/gene/?term=7320)(22.373)[CYP1A2](http://www.ncbi.nlm.nih.gov/gene/?term=1544)(22.373)[CCL5](http://www.ncbi.nlm.nih.gov/gene/?term=6352)(22.373)[ANK2](http://www.ncbi.nlm.nih.gov/gene/?term=287)(22.373)[HSD17B2](http://www.ncbi.nlm.nih.gov/gene/?term=3294)(22.373) [STIM2](http://www.ncbi.nlm.nih.gov/gene/?term=57620)(22.373)[PKD2](http://www.ncbi.nlm.nih.gov/gene/?term=5311)(22.373)[ALDH9A1](http://www.ncbi.nlm.nih.gov/gene/?term=223)(22.373)[TH](http://www.ncbi.nlm.nih.gov/gene/?term=7054)(22.373)[EPO](http://www.ncbi.nlm.nih.gov/gene/?term=2056)(22.373) [COLGALT1](http://www.ncbi.nlm.nih.gov/gene/?term=79709)(22.373)[NEDD4](http://www.ncbi.nlm.nih.gov/gene/?term=4734)(22.373)[CYP11A1](http://www.ncbi.nlm.nih.gov/gene/?term=1583)(22.373)[DHODH](http://www.ncbi.nlm.nih.gov/gene/?term=1723)(22.373)[CLN3](http://www.ncbi.nlm.nih.gov/gene/?term=1201)(22.373) [ARID1A](http://www.ncbi.nlm.nih.gov/gene/?term=8289)(22.373)[EGLN2](http://www.ncbi.nlm.nih.gov/gene/?term=112398)(22.373)[ACAT1](http://www.ncbi.nlm.nih.gov/gene/?term=38)(22.373)[GALC](http://www.ncbi.nlm.nih.gov/gene/?term=2581)(22.373)[PLCG2](http://www.ncbi.nlm.nih.gov/gene/?term=5336)(22.373) [ACOT4](http://www.ncbi.nlm.nih.gov/gene/?term=122970)(22.373)[PHGDH](http://www.ncbi.nlm.nih.gov/gene/?term=26227)(22.373)[PNLIPRP2](http://www.ncbi.nlm.nih.gov/gene/?term=5408)(22.373)[CYP39A1](http://www.ncbi.nlm.nih.gov/gene/?term=51302)(22.373)[BCKDHA](http://www.ncbi.nlm.nih.gov/gene/?term=593)(22.373) [SLC25A12](http://www.ncbi.nlm.nih.gov/gene/?term=8604)(22.373)[YBX3](http://www.ncbi.nlm.nih.gov/gene/?term=8531)(22.373)[8-Mar](http://www.ncbi.nlm.nih.gov/gene/?term=220972)(22.373)[STIM1](http://www.ncbi.nlm.nih.gov/gene/?term=6786)(22.373)[GAL3ST1](http://www.ncbi.nlm.nih.gov/gene/?term=9514)(22.373) [CDH11](http://www.ncbi.nlm.nih.gov/gene/?term=1009)(22.373)[CACNA1D](http://www.ncbi.nlm.nih.gov/gene/?term=776)(22.373)[DDC](http://www.ncbi.nlm.nih.gov/gene/?term=1644)(22.373)[DHTKD1](http://www.ncbi.nlm.nih.gov/gene/?term=55526)(22.373)[NEDD4L](http://www.ncbi.nlm.nih.gov/gene/?term=23327)(22.373) [BDKRB2](http://www.ncbi.nlm.nih.gov/gene/?term=624)(22.373)[PRPH](http://www.ncbi.nlm.nih.gov/gene/?term=5630)(22.373)[BCKDHB](http://www.ncbi.nlm.nih.gov/gene/?term=594)(22.373)[KCNE5](http://www.ncbi.nlm.nih.gov/gene/?term=23630)(22.373)[SRD5A3](http://www.ncbi.nlm.nih.gov/gene/?term=79644)(22.373) [SMAD7](http://www.ncbi.nlm.nih.gov/gene/?term=4092)(22.373)[PRDM8](http://www.ncbi.nlm.nih.gov/gene/?term=56978)(22.373)[UROD](http://www.ncbi.nlm.nih.gov/gene/?term=7389)(22.373)[UGT8](http://www.ncbi.nlm.nih.gov/gene/?term=7368)(22.373)[ETHE1](http://www.ncbi.nlm.nih.gov/gene/?term=23474)(22.373) [WNT10B](http://www.ncbi.nlm.nih.gov/gene/?term=7480)(22.373)[OGFOD1](http://www.ncbi.nlm.nih.gov/gene/?term=55239)(22.373)[SLC38A7](http://www.ncbi.nlm.nih.gov/gene/?term=55238)(22.373)[ACSS1](http://www.ncbi.nlm.nih.gov/gene/?term=84532)(22.373)[CYP4B1](http://www.ncbi.nlm.nih.gov/gene/?term=1580)(22.373) [STAR](http://www.ncbi.nlm.nih.gov/gene/?term=6770)(22.373)[DLST](http://www.ncbi.nlm.nih.gov/gene/?term=1743)(22.373)[PF4](http://www.ncbi.nlm.nih.gov/gene/?term=5196)(22.373)[CRACR2A](http://www.ncbi.nlm.nih.gov/gene/?term=84766)(22.373)[HSD17B1](http://www.ncbi.nlm.nih.gov/gene/?term=3292)(22.373) [CAMK2D](http://www.ncbi.nlm.nih.gov/gene/?term=817)(22.373)[AP3D1](http://www.ncbi.nlm.nih.gov/gene/?term=8943)(22.373)[MYO5A](http://www.ncbi.nlm.nih.gov/gene/?term=4644)(22.373)[ILVBL](http://www.ncbi.nlm.nih.gov/gene/?term=10994)(22.373)[CAV3](http://www.ncbi.nlm.nih.gov/gene/?term=859)(22.373) [HTT](http://www.ncbi.nlm.nih.gov/gene/?term=3064)(22.373)[IL10](http://www.ncbi.nlm.nih.gov/gene/?term=3586)(22.373)[EGLN3](http://www.ncbi.nlm.nih.gov/gene/?term=112399)(22.373)[OGDHL](http://www.ncbi.nlm.nih.gov/gene/?term=55753)(22.373)[KCNA5](http://www.ncbi.nlm.nih.gov/gene/?term=3741)(22.373) [TST](http://www.ncbi.nlm.nih.gov/gene/?term=7263)(22.373)[SLC1A6](http://www.ncbi.nlm.nih.gov/gene/?term=6511)(22.373)[NPPA](http://www.ncbi.nlm.nih.gov/gene/?term=4878)(22.373)[ACADM](http://www.ncbi.nlm.nih.gov/gene/?term=34)(22.373)[SQRDL](http://www.ncbi.nlm.nih.gov/gene/?term=58472)(22.373) [TMEM110](http://www.ncbi.nlm.nih.gov/gene/?term=375346)(22.373)[CDC42](http://www.ncbi.nlm.nih.gov/gene/?term=998)(22.373)[ARHGEF2](http://www.ncbi.nlm.nih.gov/gene/?term=9181)(22.373)[EGLN1](http://www.ncbi.nlm.nih.gov/gene/?term=54583)(22.373)[ETFDH](http://www.ncbi.nlm.nih.gov/gene/?term=2110)(22.373) [EPHA4](http://www.ncbi.nlm.nih.gov/gene/?term=2043)(22.373)[OCA2](http://www.ncbi.nlm.nih.gov/gene/?term=4948)(22.373)[HSD17B8](http://www.ncbi.nlm.nih.gov/gene/?term=7923)(22.373)[CTNNB1](http://www.ncbi.nlm.nih.gov/gene/?term=1499)(22.373)[P4HA3](http://www.ncbi.nlm.nih.gov/gene/?term=283208)(22.373) [EDA](http://www.ncbi.nlm.nih.gov/gene/?term=1896)(22.373)[HACL1](http://www.ncbi.nlm.nih.gov/gene/?term=26061)(22.373)[RYR2](http://www.ncbi.nlm.nih.gov/gene/?term=6262)(22.373)[HAP1](http://www.ncbi.nlm.nih.gov/gene/?term=9001)(22.373)[PCSK9](http://www.ncbi.nlm.nih.gov/gene/?term=255738)(22.373) [KCNQ1](http://www.ncbi.nlm.nih.gov/gene/?term=3784)(22.373)[CASQ2](http://www.ncbi.nlm.nih.gov/gene/?term=845)(22.373)[GJA5](http://www.ncbi.nlm.nih.gov/gene/?term=2702)(22.373)[DHRS9](http://www.ncbi.nlm.nih.gov/gene/?term=10170)(22.373)[GPR143](http://www.ncbi.nlm.nih.gov/gene/?term=4935)(22.373) [UGT1A1](http://www.ncbi.nlm.nih.gov/gene/?term=54658)(22.373)[DLD](http://www.ncbi.nlm.nih.gov/gene/?term=1738)(22.373)[GNAS](http://www.ncbi.nlm.nih.gov/gene/?term=2778)(22.373)[SLC25A13](http://www.ncbi.nlm.nih.gov/gene/?term=10165)(22.373)[SFXN5](http://www.ncbi.nlm.nih.gov/gene/?term=94097)(22.373) [PLOD2](http://www.ncbi.nlm.nih.gov/gene/?term=5352)(22.373)[CYP1A1](http://www.ncbi.nlm.nih.gov/gene/?term=1543)(22.373)[SULT2A1](http://www.ncbi.nlm.nih.gov/gene/?term=6822)(22.373)[NFX1](http://www.ncbi.nlm.nih.gov/gene/?term=4799)(22.373)[DBT](http://www.ncbi.nlm.nih.gov/gene/?term=1629)(22.373) [SLC25A1](http://www.ncbi.nlm.nih.gov/gene/?term=6576)(22.373) | |  |
| [Isocarthamidin](http://www.megabionet.org/tcmid/ingredient/23706/) | [SOAT1](http://www.ncbi.nlm.nih.gov/gene/?term=6646)(122.778)[MTTP](http://www.ncbi.nlm.nih.gov/gene/?term=4547)(122.778)[SOAT2](http://www.ncbi.nlm.nih.gov/gene/?term=8435)(122.778)[CNR2](http://www.ncbi.nlm.nih.gov/gene/?term=1269)(48.000)[CNR1](http://www.ncbi.nlm.nih.gov/gene/?term=1268)(48.000) [DRD2](http://www.ncbi.nlm.nih.gov/gene/?term=1813)(22.373)[PAWR](http://www.ncbi.nlm.nih.gov/gene/?term=5074)(22.373) | |  |
| [Isoliquiritin](http://www.megabionet.org/tcmid/ingredient/11502/) | This compound doesn't have any potential target with score larger than 20. | |  |
| [Wogonoside](http://www.megabionet.org/tcmid/ingredient/22704/) | This compound doesn't have any potential target with score larger than 20. | |  |
| [Ricinine](http://www.megabionet.org/tcmid/ingredient/18825/) | [SEC14L3](http://www.ncbi.nlm.nih.gov/gene/?term=266629)(23.000)[PPP2CA](http://www.ncbi.nlm.nih.gov/gene/?term=5515)(23.000)[PRKCA](http://www.ncbi.nlm.nih.gov/gene/?term=5578)(23.000)[NR1I2](http://www.ncbi.nlm.nih.gov/gene/?term=8856)(23.000)[ALOX5](http://www.ncbi.nlm.nih.gov/gene/?term=240)(23.000) [PPP2CB](http://www.ncbi.nlm.nih.gov/gene/?term=5516)(23.000)[SEC14L2](http://www.ncbi.nlm.nih.gov/gene/?term=23541)(23.000)[DGKA](http://www.ncbi.nlm.nih.gov/gene/?term=1606)(23.000)[PRKCB](http://www.ncbi.nlm.nih.gov/gene/?term=5579)(23.000)[SEC14L4](http://www.ncbi.nlm.nih.gov/gene/?term=284904)(23.000) | |  |
| [Methyl-24-Hydroxyglycyrrhetate](http://www.megabionet.org/tcmid/ingredient/14500/) | [AR](http://www.ncbi.nlm.nih.gov/gene/?term=367)(122.778)[SRD5A1](http://www.ncbi.nlm.nih.gov/gene/?term=6715)(48.000)[CYP17A1](http://www.ncbi.nlm.nih.gov/gene/?term=1586)(48.000)[PRLR](http://www.ncbi.nlm.nih.gov/gene/?term=5618)(48.000)[ESR1](http://www.ncbi.nlm.nih.gov/gene/?term=2099)(48.000) [PTGER4](http://www.ncbi.nlm.nih.gov/gene/?term=5734)(48.000)[PGR](http://www.ncbi.nlm.nih.gov/gene/?term=5241)(48.000)[NR3C1](http://www.ncbi.nlm.nih.gov/gene/?term=2908)(48.000)[OPRK1](http://www.ncbi.nlm.nih.gov/gene/?term=4986)(48.000)[PTGER2](http://www.ncbi.nlm.nih.gov/gene/?term=5732)(48.000) [PTGER3](http://www.ncbi.nlm.nih.gov/gene/?term=5733)(48.000)[ANXA1](http://www.ncbi.nlm.nih.gov/gene/?term=301)(48.000)[CYP19A1](http://www.ncbi.nlm.nih.gov/gene/?term=1588)(48.000)[NR3C2](http://www.ncbi.nlm.nih.gov/gene/?term=4306)(48.000)[PRKCA](http://www.ncbi.nlm.nih.gov/gene/?term=5578)(23.000) [PRKCD](http://www.ncbi.nlm.nih.gov/gene/?term=5580)(23.000) | |  |
| [Astramembrannin I](http://www.megabionet.org/tcmid/ingredient/1945/) | [CXCR4](http://www.ncbi.nlm.nih.gov/gene/?term=7852)(48.000) | |  |
| [Glyuranolide](http://www.megabionet.org/tcmid/ingredient/8854/) | [NR3C2](http://www.ncbi.nlm.nih.gov/gene/?term=4306)(122.778)[ESR1](http://www.ncbi.nlm.nih.gov/gene/?term=2099)(48.000)[PGR](http://www.ncbi.nlm.nih.gov/gene/?term=5241)(48.000)[AR](http://www.ncbi.nlm.nih.gov/gene/?term=367)(48.000)[NR3C1](http://www.ncbi.nlm.nih.gov/gene/?term=2908)(48.000) [HMGCR](http://www.ncbi.nlm.nih.gov/gene/?term=3156)(48.000)[ITGB2](http://www.ncbi.nlm.nih.gov/gene/?term=3689)(48.000)[ITGAL](http://www.ncbi.nlm.nih.gov/gene/?term=3683)(48.000)[CYP19A1](http://www.ncbi.nlm.nih.gov/gene/?term=1588)(48.000)[HDAC2](http://www.ncbi.nlm.nih.gov/gene/?term=3066)(48.000) | |  |
| [Taraxerone](http://www.megabionet.org/tcmid/ingredient/20696/) | [CYP17A1](http://www.ncbi.nlm.nih.gov/gene/?term=1586)(122.778)[ESR1](http://www.ncbi.nlm.nih.gov/gene/?term=2099)(122.778)[PGR](http://www.ncbi.nlm.nih.gov/gene/?term=5241)(122.778)[OPRK1](http://www.ncbi.nlm.nih.gov/gene/?term=4986)(122.778)[NR3C2](http://www.ncbi.nlm.nih.gov/gene/?term=4306)(122.778) [PDE7B](http://www.ncbi.nlm.nih.gov/gene/?term=27115)(80.882)[PDE5A](http://www.ncbi.nlm.nih.gov/gene/?term=8654)(80.882)[PDE9A](http://www.ncbi.nlm.nih.gov/gene/?term=5152)(80.882)[PDE4A](http://www.ncbi.nlm.nih.gov/gene/?term=5141)(80.882)[RYR1](http://www.ncbi.nlm.nih.gov/gene/?term=6261)(80.882) [PDE3A](http://www.ncbi.nlm.nih.gov/gene/?term=5139)(80.882)[ADORA2A](http://www.ncbi.nlm.nih.gov/gene/?term=135)(80.882)[ITPR1](http://www.ncbi.nlm.nih.gov/gene/?term=3708)(80.882)[PDE6A](http://www.ncbi.nlm.nih.gov/gene/?term=5145)(80.882)[PRKDC](http://www.ncbi.nlm.nih.gov/gene/?term=5591)(80.882) [PIK3CD](http://www.ncbi.nlm.nih.gov/gene/?term=5293)(80.882)[PDE3B](http://www.ncbi.nlm.nih.gov/gene/?term=5140)(80.882)[PGD](http://www.ncbi.nlm.nih.gov/gene/?term=5226)(80.882)[PIK3CA](http://www.ncbi.nlm.nih.gov/gene/?term=5290)(80.882)[ADORA2B](http://www.ncbi.nlm.nih.gov/gene/?term=136)(80.882) [PDE1A](http://www.ncbi.nlm.nih.gov/gene/?term=5136)(80.882)[PDE4D](http://www.ncbi.nlm.nih.gov/gene/?term=5144)(80.882)[PIK3CB](http://www.ncbi.nlm.nih.gov/gene/?term=5291)(80.882)[ITPR2](http://www.ncbi.nlm.nih.gov/gene/?term=3709)(80.882)[PDE1B](http://www.ncbi.nlm.nih.gov/gene/?term=5153)(80.882) [PDE7A](http://www.ncbi.nlm.nih.gov/gene/?term=5150)(80.882)[ITPR3](http://www.ncbi.nlm.nih.gov/gene/?term=3710)(80.882)[POLA2](http://www.ncbi.nlm.nih.gov/gene/?term=23649)(80.882)[PDE4C](http://www.ncbi.nlm.nih.gov/gene/?term=5143)(80.882)[PDE10A](http://www.ncbi.nlm.nih.gov/gene/?term=10846)(80.882) [PDE1C](http://www.ncbi.nlm.nih.gov/gene/?term=5137)(80.882)[ATM](http://www.ncbi.nlm.nih.gov/gene/?term=472)(80.882)[PDE6B](http://www.ncbi.nlm.nih.gov/gene/?term=5158)(80.882)[PDE4B](http://www.ncbi.nlm.nih.gov/gene/?term=5142)(80.882)[PDE2A](http://www.ncbi.nlm.nih.gov/gene/?term=5138)(80.882) [ADORA1](http://www.ncbi.nlm.nih.gov/gene/?term=134)(80.882)[CYP19A1](http://www.ncbi.nlm.nih.gov/gene/?term=1588)(80.882)[PDE8B](http://www.ncbi.nlm.nih.gov/gene/?term=8622)(80.882)[NT5E](http://www.ncbi.nlm.nih.gov/gene/?term=4907)(80.882)[HDAC2](http://www.ncbi.nlm.nih.gov/gene/?term=3066)(80.882) [PDE6C](http://www.ncbi.nlm.nih.gov/gene/?term=5146)(80.882)[PDE11A](http://www.ncbi.nlm.nih.gov/gene/?term=50940)(80.882)[PDE8A](http://www.ncbi.nlm.nih.gov/gene/?term=5151)(80.882)[RINT1](http://www.ncbi.nlm.nih.gov/gene/?term=60561)(55.444)[RIPK1](http://www.ncbi.nlm.nih.gov/gene/?term=8737)(55.444) [PIK3R1](http://www.ncbi.nlm.nih.gov/gene/?term=5295)(55.444)[WNT4](http://www.ncbi.nlm.nih.gov/gene/?term=54361)(55.444)[TACR2](http://www.ncbi.nlm.nih.gov/gene/?term=6865)(55.444)[AMPD3](http://www.ncbi.nlm.nih.gov/gene/?term=272)(55.444)[CX3CR1](http://www.ncbi.nlm.nih.gov/gene/?term=1524)(55.444) [ADA](http://www.ncbi.nlm.nih.gov/gene/?term=100)(55.444)[HAP1](http://www.ncbi.nlm.nih.gov/gene/?term=9001)(55.444)[IDNK](http://www.ncbi.nlm.nih.gov/gene/?term=414328)(55.444)[SRD5A1](http://www.ncbi.nlm.nih.gov/gene/?term=6715)(48.000)[F12](http://www.ncbi.nlm.nih.gov/gene/?term=2161)(48.000) [AR](http://www.ncbi.nlm.nih.gov/gene/?term=367)(48.000)[NR3C1](http://www.ncbi.nlm.nih.gov/gene/?term=2908)(48.000)[ANXA1](http://www.ncbi.nlm.nih.gov/gene/?term=301)(48.000)[HDAC1](http://www.ncbi.nlm.nih.gov/gene/?term=3065)(23.000)[TFAP2C](http://www.ncbi.nlm.nih.gov/gene/?term=7022)(22.373) [HMGA2](http://www.ncbi.nlm.nih.gov/gene/?term=8091)(22.373)[TNFSF11](http://www.ncbi.nlm.nih.gov/gene/?term=8600)(22.373)[NT5C1A](http://www.ncbi.nlm.nih.gov/gene/?term=84618)(22.373)[ACTN3](http://www.ncbi.nlm.nih.gov/gene/?term=89)(22.373)[NOS1AP](http://www.ncbi.nlm.nih.gov/gene/?term=9722)(22.373) [TAC1](http://www.ncbi.nlm.nih.gov/gene/?term=6863)(22.373)[PHB](http://www.ncbi.nlm.nih.gov/gene/?term=5245)(22.373)[SIRT1](http://www.ncbi.nlm.nih.gov/gene/?term=23411)(22.373)[DGKI](http://www.ncbi.nlm.nih.gov/gene/?term=9162)(22.373)[FKBP1A](http://www.ncbi.nlm.nih.gov/gene/?term=2280)(22.373) [FURIN](http://www.ncbi.nlm.nih.gov/gene/?term=5045)(22.373)[TOX3](http://www.ncbi.nlm.nih.gov/gene/?term=27324)(22.373)[UTS2](http://www.ncbi.nlm.nih.gov/gene/?term=10911)(22.373)[STUB1](http://www.ncbi.nlm.nih.gov/gene/?term=10273)(22.373)[CTR9](http://www.ncbi.nlm.nih.gov/gene/?term=9646)(22.373) [G6PD](http://www.ncbi.nlm.nih.gov/gene/?term=2539)(22.373)[GNAT1](http://www.ncbi.nlm.nih.gov/gene/?term=2779)(22.373)[SIX1](http://www.ncbi.nlm.nih.gov/gene/?term=6495)(22.373)[QDPR](http://www.ncbi.nlm.nih.gov/gene/?term=5860)(22.373)[TGFB1](http://www.ncbi.nlm.nih.gov/gene/?term=7040)(22.373) [ATP11C](http://www.ncbi.nlm.nih.gov/gene/?term=286410)(22.373)[KHDRBS1](http://www.ncbi.nlm.nih.gov/gene/?term=10657)(22.373)[PPP3CB](http://www.ncbi.nlm.nih.gov/gene/?term=5532)(22.373)[SYT2](http://www.ncbi.nlm.nih.gov/gene/?term=127833)(22.373)[SIX4](http://www.ncbi.nlm.nih.gov/gene/?term=51804)(22.373) [SIRT2](http://www.ncbi.nlm.nih.gov/gene/?term=22933)(22.373)[SPI1](http://www.ncbi.nlm.nih.gov/gene/?term=6688)(22.373)[HOMER1](http://www.ncbi.nlm.nih.gov/gene/?term=9456)(22.373)[SHANK3](http://www.ncbi.nlm.nih.gov/gene/?term=85358)(22.373)[TAS1R1](http://www.ncbi.nlm.nih.gov/gene/?term=80835)(22.373) [TRIM28](http://www.ncbi.nlm.nih.gov/gene/?term=10155)(22.373)[XRCC4](http://www.ncbi.nlm.nih.gov/gene/?term=7518)(22.373)[MED1](http://www.ncbi.nlm.nih.gov/gene/?term=5469)(22.373)[NKX3-1](http://www.ncbi.nlm.nih.gov/gene/?term=4824)(22.373)[UBE2B](http://www.ncbi.nlm.nih.gov/gene/?term=7320)(22.373) [ESR2](http://www.ncbi.nlm.nih.gov/gene/?term=2100)(22.373)[HCN4](http://www.ncbi.nlm.nih.gov/gene/?term=10021)(22.373)[EDN1](http://www.ncbi.nlm.nih.gov/gene/?term=1906)(22.373)[CACNA1A](http://www.ncbi.nlm.nih.gov/gene/?term=773)(22.373)[STAT5A](http://www.ncbi.nlm.nih.gov/gene/?term=6776)(22.373) [TP53](http://www.ncbi.nlm.nih.gov/gene/?term=7157)(22.373)[GNAT3](http://www.ncbi.nlm.nih.gov/gene/?term=346562)(22.373)[HELB](http://www.ncbi.nlm.nih.gov/gene/?term=92797)(22.373)[COMT](http://www.ncbi.nlm.nih.gov/gene/?term=1312)(22.373)[SLC26A6](http://www.ncbi.nlm.nih.gov/gene/?term=65010)(22.373) [ADRA2A](http://www.ncbi.nlm.nih.gov/gene/?term=150)(22.373)[YWHAE](http://www.ncbi.nlm.nih.gov/gene/?term=7531)(22.373)[CETN2](http://www.ncbi.nlm.nih.gov/gene/?term=1069)(22.373)[POLA1](http://www.ncbi.nlm.nih.gov/gene/?term=5422)(22.373)[CRP](http://www.ncbi.nlm.nih.gov/gene/?term=1401)(22.373) [CETN1](http://www.ncbi.nlm.nih.gov/gene/?term=1068)(22.373)[NUDT9](http://www.ncbi.nlm.nih.gov/gene/?term=53343)(22.373)[PIK3CG](http://www.ncbi.nlm.nih.gov/gene/?term=5294)(22.373)[BMP2](http://www.ncbi.nlm.nih.gov/gene/?term=650)(22.373)[TNF](http://www.ncbi.nlm.nih.gov/gene/?term=7124)(22.373) [NEDD4](http://www.ncbi.nlm.nih.gov/gene/?term=4734)(22.373)[AGT](http://www.ncbi.nlm.nih.gov/gene/?term=183)(22.373)[FER](http://www.ncbi.nlm.nih.gov/gene/?term=2241)(22.373)[SHH](http://www.ncbi.nlm.nih.gov/gene/?term=6469)(22.373)[SERPINB3](http://www.ncbi.nlm.nih.gov/gene/?term=6317)(22.373) [FOXP3](http://www.ncbi.nlm.nih.gov/gene/?term=50943)(22.373)[CXCL13](http://www.ncbi.nlm.nih.gov/gene/?term=10563)(22.373)[ARID1A](http://www.ncbi.nlm.nih.gov/gene/?term=8289)(22.373)[FGF10](http://www.ncbi.nlm.nih.gov/gene/?term=2255)(22.373)[KCNB1](http://www.ncbi.nlm.nih.gov/gene/?term=3745)(22.373) [LEF1](http://www.ncbi.nlm.nih.gov/gene/?term=51176)(22.373)[PTK2B](http://www.ncbi.nlm.nih.gov/gene/?term=2185)(22.373)[LIG4](http://www.ncbi.nlm.nih.gov/gene/?term=3981)(22.373)[ADRBK1](http://www.ncbi.nlm.nih.gov/gene/?term=156)(22.373)[MAOB](http://www.ncbi.nlm.nih.gov/gene/?term=4129)(22.373) [SORCS3](http://www.ncbi.nlm.nih.gov/gene/?term=22986)(22.373)[ADRB1](http://www.ncbi.nlm.nih.gov/gene/?term=153)(22.373)[ADAM8](http://www.ncbi.nlm.nih.gov/gene/?term=101)(22.373)[VTI1A](http://www.ncbi.nlm.nih.gov/gene/?term=143187)(22.373)[SLC44A4](http://www.ncbi.nlm.nih.gov/gene/?term=80736)(22.373) [ADAP2](http://www.ncbi.nlm.nih.gov/gene/?term=55803)(22.373)[LRRC8A](http://www.ncbi.nlm.nih.gov/gene/?term=56262)(22.373)[FGFR2](http://www.ncbi.nlm.nih.gov/gene/?term=2263)(22.373)[AURKA](http://www.ncbi.nlm.nih.gov/gene/?term=6790)(22.373)[MCM3](http://www.ncbi.nlm.nih.gov/gene/?term=4172)(22.373) [VCP](http://www.ncbi.nlm.nih.gov/gene/?term=7415)(22.373)[GHRL](http://www.ncbi.nlm.nih.gov/gene/?term=51738)(22.373)[BCL11B](http://www.ncbi.nlm.nih.gov/gene/?term=64919)(22.373)[BCL2](http://www.ncbi.nlm.nih.gov/gene/?term=596)(22.373)[8-Mar](http://www.ncbi.nlm.nih.gov/gene/?term=220972)(22.373) [GAS6](http://www.ncbi.nlm.nih.gov/gene/?term=2621)(22.373)[C5](http://www.ncbi.nlm.nih.gov/gene/?term=727)(22.373)[SREBF1](http://www.ncbi.nlm.nih.gov/gene/?term=6720)(22.373)[DRD2](http://www.ncbi.nlm.nih.gov/gene/?term=1813)(22.373)[PLN](http://www.ncbi.nlm.nih.gov/gene/?term=5350)(22.373) [DNM3](http://www.ncbi.nlm.nih.gov/gene/?term=26052)(22.373)[CHRNA3](http://www.ncbi.nlm.nih.gov/gene/?term=1136)(22.373)[TCF3](http://www.ncbi.nlm.nih.gov/gene/?term=6929)(22.373)[SRC](http://www.ncbi.nlm.nih.gov/gene/?term=6714)(22.373)[LONP1](http://www.ncbi.nlm.nih.gov/gene/?term=9361)(22.373) [XCL1](http://www.ncbi.nlm.nih.gov/gene/?term=6375)(22.373)[HSP90AB1](http://www.ncbi.nlm.nih.gov/gene/?term=3326)(22.373)[HIBADH](http://www.ncbi.nlm.nih.gov/gene/?term=11112)(22.373)[STAP1](http://www.ncbi.nlm.nih.gov/gene/?term=26228)(22.373)[HRC](http://www.ncbi.nlm.nih.gov/gene/?term=3270)(22.373) [UTS2R](http://www.ncbi.nlm.nih.gov/gene/?term=2837)(22.373)[SLC6A4](http://www.ncbi.nlm.nih.gov/gene/?term=6532)(22.373)[HMGCR](http://www.ncbi.nlm.nih.gov/gene/?term=3156)(22.373)[HIPK2](http://www.ncbi.nlm.nih.gov/gene/?term=28996)(22.373)[PPP3CA](http://www.ncbi.nlm.nih.gov/gene/?term=5530)(22.373) [RAPGEF2](http://www.ncbi.nlm.nih.gov/gene/?term=9693)(22.373)[TET1](http://www.ncbi.nlm.nih.gov/gene/?term=80312)(22.373)[C2CD5](http://www.ncbi.nlm.nih.gov/gene/?term=9847)(22.373)[CSF2](http://www.ncbi.nlm.nih.gov/gene/?term=1437)(22.373)[TALDO1](http://www.ncbi.nlm.nih.gov/gene/?term=6888)(22.373) [BDKRB2](http://www.ncbi.nlm.nih.gov/gene/?term=624)(22.373)[KCNH2](http://www.ncbi.nlm.nih.gov/gene/?term=3757)(22.373)[MMP28](http://www.ncbi.nlm.nih.gov/gene/?term=79148)(22.373)[HIF1A](http://www.ncbi.nlm.nih.gov/gene/?term=3091)(22.373)[KIT](http://www.ncbi.nlm.nih.gov/gene/?term=3815)(22.373) [INS](http://www.ncbi.nlm.nih.gov/gene/?term=3630)(22.373)[MTOR](http://www.ncbi.nlm.nih.gov/gene/?term=2475)(22.373)[WNT10B](http://www.ncbi.nlm.nih.gov/gene/?term=7480)(22.373)[SLC18A3](http://www.ncbi.nlm.nih.gov/gene/?term=6572)(22.373)[ABCC4](http://www.ncbi.nlm.nih.gov/gene/?term=10257)(22.373) [BNIP3](http://www.ncbi.nlm.nih.gov/gene/?term=664)(22.373)[ZFPM1](http://www.ncbi.nlm.nih.gov/gene/?term=161882)(22.373)[GATA3](http://www.ncbi.nlm.nih.gov/gene/?term=2625)(22.373)[UBR5](http://www.ncbi.nlm.nih.gov/gene/?term=51366)(22.373)[AREG](http://www.ncbi.nlm.nih.gov/gene/?term=374)(22.373) [CRH](http://www.ncbi.nlm.nih.gov/gene/?term=1392)(22.373)[ZFP42](http://www.ncbi.nlm.nih.gov/gene/?term=132625)(22.373)[PIK3R6](http://www.ncbi.nlm.nih.gov/gene/?term=146850)(22.373)[PF4](http://www.ncbi.nlm.nih.gov/gene/?term=5196)(22.373)[ATP1A2](http://www.ncbi.nlm.nih.gov/gene/?term=477)(22.373) [AVPR2](http://www.ncbi.nlm.nih.gov/gene/?term=554)(22.373)[CAMK2D](http://www.ncbi.nlm.nih.gov/gene/?term=817)(22.373)[ADRA1B](http://www.ncbi.nlm.nih.gov/gene/?term=147)(22.373)[IL10](http://www.ncbi.nlm.nih.gov/gene/?term=3586)(22.373)[NEFL](http://www.ncbi.nlm.nih.gov/gene/?term=4747)(22.373) [UCN2](http://www.ncbi.nlm.nih.gov/gene/?term=90226)(22.373)[ATP1A1](http://www.ncbi.nlm.nih.gov/gene/?term=476)(22.373)[RYR3](http://www.ncbi.nlm.nih.gov/gene/?term=6263)(22.373)[PPP3R1](http://www.ncbi.nlm.nih.gov/gene/?term=5534)(22.373)[SPX](http://www.ncbi.nlm.nih.gov/gene/?term=80763)(22.373) [ATP2A1](http://www.ncbi.nlm.nih.gov/gene/?term=487)(22.373)[SMO](http://www.ncbi.nlm.nih.gov/gene/?term=6608)(22.373)[RAG1](http://www.ncbi.nlm.nih.gov/gene/?term=5896)(22.373)[RAB8B](http://www.ncbi.nlm.nih.gov/gene/?term=51762)(22.373)[DKK3](http://www.ncbi.nlm.nih.gov/gene/?term=27122)(22.373) [AHCYL1](http://www.ncbi.nlm.nih.gov/gene/?term=10768)(22.373)[AQP1](http://www.ncbi.nlm.nih.gov/gene/?term=358)(22.373)[TNFAIP3](http://www.ncbi.nlm.nih.gov/gene/?term=7128)(22.373)[TAC4](http://www.ncbi.nlm.nih.gov/gene/?term=255061)(22.373)[DPPA3](http://www.ncbi.nlm.nih.gov/gene/?term=359787)(22.373) [CASQ1](http://www.ncbi.nlm.nih.gov/gene/?term=844)(22.373)[FKBP1B](http://www.ncbi.nlm.nih.gov/gene/?term=2281)(22.373)[CDC42](http://www.ncbi.nlm.nih.gov/gene/?term=998)(22.373)[IL4](http://www.ncbi.nlm.nih.gov/gene/?term=3565)(22.373)[POLB](http://www.ncbi.nlm.nih.gov/gene/?term=5423)(22.373) [IDO1](http://www.ncbi.nlm.nih.gov/gene/?term=3620)(22.373)[ATP2B4](http://www.ncbi.nlm.nih.gov/gene/?term=493)(22.373)[CTNNB1](http://www.ncbi.nlm.nih.gov/gene/?term=1499)(22.373)[PGLS](http://www.ncbi.nlm.nih.gov/gene/?term=25796)(22.373)[SLC9A3R1](http://www.ncbi.nlm.nih.gov/gene/?term=9368)(22.373) [IGF2](http://www.ncbi.nlm.nih.gov/gene/?term=3481)(22.373)[HPS4](http://www.ncbi.nlm.nih.gov/gene/?term=89781)(22.373)[TKT](http://www.ncbi.nlm.nih.gov/gene/?term=7086)(22.373)[DRD1](http://www.ncbi.nlm.nih.gov/gene/?term=1812)(22.373)[ADAP1](http://www.ncbi.nlm.nih.gov/gene/?term=11033)(22.373) [EDA](http://www.ncbi.nlm.nih.gov/gene/?term=1896)(22.373)[RYR2](http://www.ncbi.nlm.nih.gov/gene/?term=6262)(22.373)[CD34](http://www.ncbi.nlm.nih.gov/gene/?term=947)(22.373)[HSP90AA1](http://www.ncbi.nlm.nih.gov/gene/?term=3320)(22.373)[LTA](http://www.ncbi.nlm.nih.gov/gene/?term=4049)(22.373) [SPR](http://www.ncbi.nlm.nih.gov/gene/?term=6697)(22.373)[GPER1](http://www.ncbi.nlm.nih.gov/gene/?term=2852)(22.373)[CBFA2T3](http://www.ncbi.nlm.nih.gov/gene/?term=863)(22.373)[CALHM1](http://www.ncbi.nlm.nih.gov/gene/?term=255022)(22.373)[WNT5A](http://www.ncbi.nlm.nih.gov/gene/?term=7474)(22.373) [SELP](http://www.ncbi.nlm.nih.gov/gene/?term=6403)(22.373)[XRCC6BP1](http://www.ncbi.nlm.nih.gov/gene/?term=91419)(22.373)[PARP10](http://www.ncbi.nlm.nih.gov/gene/?term=84875)(22.373)[HPRT1](http://www.ncbi.nlm.nih.gov/gene/?term=3251)(22.373)[AMICA1](http://www.ncbi.nlm.nih.gov/gene/?term=120425)(22.373) [TAS1R3](http://www.ncbi.nlm.nih.gov/gene/?term=83756)(22.373)[NOS1](http://www.ncbi.nlm.nih.gov/gene/?term=4842)(22.373)[TREM1](http://www.ncbi.nlm.nih.gov/gene/?term=54210)(22.373)[TAL1](http://www.ncbi.nlm.nih.gov/gene/?term=6886)(22.373)[ALAD](http://www.ncbi.nlm.nih.gov/gene/?term=210)(22.373) [PAWR](http://www.ncbi.nlm.nih.gov/gene/?term=5074)(22.373)[SOX9](http://www.ncbi.nlm.nih.gov/gene/?term=6662)(22.373)[BMP5](http://www.ncbi.nlm.nih.gov/gene/?term=653)(22.373)[PDGFB](http://www.ncbi.nlm.nih.gov/gene/?term=5155)(22.373)[RTN2](http://www.ncbi.nlm.nih.gov/gene/?term=6253)(22.373) [CD63](http://www.ncbi.nlm.nih.gov/gene/?term=967)(22.373)[CAMK2G](http://www.ncbi.nlm.nih.gov/gene/?term=818)(22.373)[LAMP2](http://www.ncbi.nlm.nih.gov/gene/?term=3920)(22.373)[NEFH](http://www.ncbi.nlm.nih.gov/gene/?term=4744)(22.373)[GLYR1](http://www.ncbi.nlm.nih.gov/gene/?term=84656)(22.373) [VDR](http://www.ncbi.nlm.nih.gov/gene/?term=7421)(22.373)[GNAS](http://www.ncbi.nlm.nih.gov/gene/?term=2778)(22.373)[NCBP2](http://www.ncbi.nlm.nih.gov/gene/?term=22916)(22.373)[TPM1](http://www.ncbi.nlm.nih.gov/gene/?term=7168)(22.373)[PIK3R5](http://www.ncbi.nlm.nih.gov/gene/?term=23533)(22.373) [PCSK6](http://www.ncbi.nlm.nih.gov/gene/?term=5046)(22.373)[TRIM24](http://www.ncbi.nlm.nih.gov/gene/?term=8805)(22.373)[REST](http://www.ncbi.nlm.nih.gov/gene/?term=5978)(22.373)[CRHR2](http://www.ncbi.nlm.nih.gov/gene/?term=1395)(22.373)[HCN2](http://www.ncbi.nlm.nih.gov/gene/?term=610)(22.373) [RAG2](http://www.ncbi.nlm.nih.gov/gene/?term=5897)(22.373)[NFX1](http://www.ncbi.nlm.nih.gov/gene/?term=4799)(22.373)[TAS1R2](http://www.ncbi.nlm.nih.gov/gene/?term=80834)(22.373) | |  |
| [5,6,7,8-Tetrahydro-4-Methylquinoline](http://www.megabionet.org/tcmid/ingredient/21059/) | [GABRB2](http://www.ncbi.nlm.nih.gov/gene/?term=2561)(80.882)[GABRB3](http://www.ncbi.nlm.nih.gov/gene/?term=2562)(80.882)[F2](http://www.ncbi.nlm.nih.gov/gene/?term=2147)(80.882)[GPR27](http://www.ncbi.nlm.nih.gov/gene/?term=2850)(55.444)[HTR1B](http://www.ncbi.nlm.nih.gov/gene/?term=3351)(48.000) [HTR1D](http://www.ncbi.nlm.nih.gov/gene/?term=3352)(48.000)[CD36](http://www.ncbi.nlm.nih.gov/gene/?term=948)(22.373)[SDC4](http://www.ncbi.nlm.nih.gov/gene/?term=6385)(22.373)[CD47](http://www.ncbi.nlm.nih.gov/gene/?term=961)(22.373)[BICD1](http://www.ncbi.nlm.nih.gov/gene/?term=636)(22.373) | |  |
| [Gancaonin E](http://www.megabionet.org/tcmid/ingredient/8137/) | [SOAT1](http://www.ncbi.nlm.nih.gov/gene/?term=6646)(122.778)[MTTP](http://www.ncbi.nlm.nih.gov/gene/?term=4547)(122.778)[SOAT2](http://www.ncbi.nlm.nih.gov/gene/?term=8435)(122.778) | |  |
| [Licocoumarone](http://www.megabionet.org/tcmid/ingredient/12765/) | [CNR2](http://www.ncbi.nlm.nih.gov/gene/?term=1269)(80.882)[CNR1](http://www.ncbi.nlm.nih.gov/gene/?term=1268)(80.882)[DRD2](http://www.ncbi.nlm.nih.gov/gene/?term=1813)(55.444)[SEC14L3](http://www.ncbi.nlm.nih.gov/gene/?term=266629)(23.000)[PPP2CA](http://www.ncbi.nlm.nih.gov/gene/?term=5515)(23.000) [PRKCA](http://www.ncbi.nlm.nih.gov/gene/?term=5578)(23.000)[NR1I2](http://www.ncbi.nlm.nih.gov/gene/?term=8856)(23.000)[ALOX5](http://www.ncbi.nlm.nih.gov/gene/?term=240)(23.000)[PPP2CB](http://www.ncbi.nlm.nih.gov/gene/?term=5516)(23.000)[SEC14L2](http://www.ncbi.nlm.nih.gov/gene/?term=23541)(23.000) [DGKA](http://www.ncbi.nlm.nih.gov/gene/?term=1606)(23.000)[PRKCB](http://www.ncbi.nlm.nih.gov/gene/?term=5579)(23.000)[SEC14L4](http://www.ncbi.nlm.nih.gov/gene/?term=284904)(23.000)[AKT1](http://www.ncbi.nlm.nih.gov/gene/?term=207)(22.373)[CCR7](http://www.ncbi.nlm.nih.gov/gene/?term=1236)(22.373) [ABHD6](http://www.ncbi.nlm.nih.gov/gene/?term=57406)(22.373)[MGLL](http://www.ncbi.nlm.nih.gov/gene/?term=11343)(22.373)[FCER1G](http://www.ncbi.nlm.nih.gov/gene/?term=2207)(22.373)[SUMO1](http://www.ncbi.nlm.nih.gov/gene/?term=7341)(22.373)[GPR55](http://www.ncbi.nlm.nih.gov/gene/?term=9290)(22.373) [CHRNB2](http://www.ncbi.nlm.nih.gov/gene/?term=1141)(22.373)[FCER1A](http://www.ncbi.nlm.nih.gov/gene/?term=2205)(22.373)[CAV3](http://www.ncbi.nlm.nih.gov/gene/?term=859)(22.373)[RNF207](http://www.ncbi.nlm.nih.gov/gene/?term=388591)(22.373)[C3](http://www.ncbi.nlm.nih.gov/gene/?term=718)(22.373) [DAGLA](http://www.ncbi.nlm.nih.gov/gene/?term=747)(22.373)[PLIN5](http://www.ncbi.nlm.nih.gov/gene/?term=440503)(22.373)[ZP3](http://www.ncbi.nlm.nih.gov/gene/?term=7784)(22.373) | |  |
| [7-Hydroxy-5,8-Dimethoxyflavone](http://www.megabionet.org/tcmid/ingredient/10027/) | This compound doesn't have any potential target with score larger than 20. | |  |
| [Foliosidine](http://www.megabionet.org/tcmid/ingredient/7853/) | This compound doesn't have any potential target with score larger than 20. | |  |
| [Ethyl Palmitate](http://www.megabionet.org/tcmid/ingredient/23863/) | [AR](http://www.ncbi.nlm.nih.gov/gene/?term=367)(122.778)[NPR1](http://www.ncbi.nlm.nih.gov/gene/?term=4881)(80.882)[CHRM1](http://www.ncbi.nlm.nih.gov/gene/?term=1128)(48.000)[AKR1D1](http://www.ncbi.nlm.nih.gov/gene/?term=6718)(48.000)[TYR](http://www.ncbi.nlm.nih.gov/gene/?term=7299)(48.000) [SRD5A2](http://www.ncbi.nlm.nih.gov/gene/?term=6716)(48.000)[CHRM2](http://www.ncbi.nlm.nih.gov/gene/?term=1129)(48.000)[NPR3](http://www.ncbi.nlm.nih.gov/gene/?term=4883)(22.373)[NPR2](http://www.ncbi.nlm.nih.gov/gene/?term=4882)(22.373) | |  |
| [Methyl Tetradecanoate](http://www.megabionet.org/tcmid/ingredient/23647/) | [NPR1](http://www.ncbi.nlm.nih.gov/gene/?term=4881)(80.882)[SCN11A](http://www.ncbi.nlm.nih.gov/gene/?term=11280)(48.000)[SCN2B](http://www.ncbi.nlm.nih.gov/gene/?term=6327)(48.000)[ABAT](http://www.ncbi.nlm.nih.gov/gene/?term=18)(48.000)[SCN1A](http://www.ncbi.nlm.nih.gov/gene/?term=6323)(48.000) [ESRRG](http://www.ncbi.nlm.nih.gov/gene/?term=2104)(48.000)[SCN3B](http://www.ncbi.nlm.nih.gov/gene/?term=55800)(48.000)[COX6C](http://www.ncbi.nlm.nih.gov/gene/?term=1345)(48.000)[COX5B](http://www.ncbi.nlm.nih.gov/gene/?term=1329)(48.000)[COX7C](http://www.ncbi.nlm.nih.gov/gene/?term=1350)(48.000) [COX1](http://www.ncbi.nlm.nih.gov/gene/?term=4512)(48.000)[SCN3A](http://www.ncbi.nlm.nih.gov/gene/?term=6328)(48.000)[AKR1C2](http://www.ncbi.nlm.nih.gov/gene/?term=1646)(48.000)[SCN7A](http://www.ncbi.nlm.nih.gov/gene/?term=6332)(48.000)[SCN5A](http://www.ncbi.nlm.nih.gov/gene/?term=6331)(48.000) [SCN10A](http://www.ncbi.nlm.nih.gov/gene/?term=6336)(48.000)[AKR1D1](http://www.ncbi.nlm.nih.gov/gene/?term=6718)(48.000)[ALDH5A1](http://www.ncbi.nlm.nih.gov/gene/?term=7915)(48.000)[SCN2A](http://www.ncbi.nlm.nih.gov/gene/?term=6326)(48.000)[COX5A](http://www.ncbi.nlm.nih.gov/gene/?term=9377)(48.000) [COX3](http://www.ncbi.nlm.nih.gov/gene/?term=4514)(48.000)[AR](http://www.ncbi.nlm.nih.gov/gene/?term=367)(48.000)[COX7A1](http://www.ncbi.nlm.nih.gov/gene/?term=1346)(48.000)[FECH](http://www.ncbi.nlm.nih.gov/gene/?term=2235)(48.000)[COX4I1](http://www.ncbi.nlm.nih.gov/gene/?term=1327)(48.000) [HDAC9](http://www.ncbi.nlm.nih.gov/gene/?term=9734)(48.000)[PLA2G1B](http://www.ncbi.nlm.nih.gov/gene/?term=5319)(48.000)[COX6A2](http://www.ncbi.nlm.nih.gov/gene/?term=1339)(48.000)[TYR](http://www.ncbi.nlm.nih.gov/gene/?term=7299)(48.000)[SCN9A](http://www.ncbi.nlm.nih.gov/gene/?term=6335)(48.000) [SCN4A](http://www.ncbi.nlm.nih.gov/gene/?term=6329)(48.000)[SCN4B](http://www.ncbi.nlm.nih.gov/gene/?term=6330)(48.000)[ADH1C](http://www.ncbi.nlm.nih.gov/gene/?term=126)(48.000)[COX6B1](http://www.ncbi.nlm.nih.gov/gene/?term=1340)(48.000)[FABP6](http://www.ncbi.nlm.nih.gov/gene/?term=2172)(48.000) [CES1](http://www.ncbi.nlm.nih.gov/gene/?term=1066)(48.000)[SRD5A2](http://www.ncbi.nlm.nih.gov/gene/?term=6716)(48.000)[ACADSB](http://www.ncbi.nlm.nih.gov/gene/?term=36)(48.000)[SCN8A](http://www.ncbi.nlm.nih.gov/gene/?term=6334)(48.000)[COX7B](http://www.ncbi.nlm.nih.gov/gene/?term=1349)(48.000) [SCN1B](http://www.ncbi.nlm.nih.gov/gene/?term=6324)(48.000)[COX2](http://www.ncbi.nlm.nih.gov/gene/?term=4513)(48.000)[OGDH](http://www.ncbi.nlm.nih.gov/gene/?term=4967)(48.000)[HDAC2](http://www.ncbi.nlm.nih.gov/gene/?term=3066)(48.000)[COX8A](http://www.ncbi.nlm.nih.gov/gene/?term=1351)(48.000) [NR1H4](http://www.ncbi.nlm.nih.gov/gene/?term=9971)(48.000)[NPR3](http://www.ncbi.nlm.nih.gov/gene/?term=4883)(22.373)[NPR2](http://www.ncbi.nlm.nih.gov/gene/?term=4882)(22.373) | |  |
| [Sigmoidin B](http://www.megabionet.org/tcmid/ingredient/19869/) | [SOAT1](http://www.ncbi.nlm.nih.gov/gene/?term=6646)(122.778)[MTTP](http://www.ncbi.nlm.nih.gov/gene/?term=4547)(122.778)[SOAT2](http://www.ncbi.nlm.nih.gov/gene/?term=8435)(122.778) | |  |
| [Uralene](http://www.megabionet.org/tcmid/ingredient/22222/) | This compound doesn't have any potential target with score larger than 20. | |  |
| [5alpha-Stigmastan-3,6-Dione](http://www.megabionet.org/tcmid/ingredient/25252/) | [ESRRG](http://www.ncbi.nlm.nih.gov/gene/?term=2104)(48.000)[COX6C](http://www.ncbi.nlm.nih.gov/gene/?term=1345)(48.000)[COX5B](http://www.ncbi.nlm.nih.gov/gene/?term=1329)(48.000)[COX7C](http://www.ncbi.nlm.nih.gov/gene/?term=1350)(48.000)[COX1](http://www.ncbi.nlm.nih.gov/gene/?term=4512)(48.000) [AKR1C2](http://www.ncbi.nlm.nih.gov/gene/?term=1646)(48.000)[COX5A](http://www.ncbi.nlm.nih.gov/gene/?term=9377)(48.000)[COX3](http://www.ncbi.nlm.nih.gov/gene/?term=4514)(48.000)[AR](http://www.ncbi.nlm.nih.gov/gene/?term=367)(48.000)[COX7A1](http://www.ncbi.nlm.nih.gov/gene/?term=1346)(48.000) [FECH](http://www.ncbi.nlm.nih.gov/gene/?term=2235)(48.000)[COX4I1](http://www.ncbi.nlm.nih.gov/gene/?term=1327)(48.000)[PLA2G1B](http://www.ncbi.nlm.nih.gov/gene/?term=5319)(48.000)[COX6A2](http://www.ncbi.nlm.nih.gov/gene/?term=1339)(48.000)[ADH1C](http://www.ncbi.nlm.nih.gov/gene/?term=126)(48.000) [COX6B1](http://www.ncbi.nlm.nih.gov/gene/?term=1340)(48.000)[FABP6](http://www.ncbi.nlm.nih.gov/gene/?term=2172)(48.000)[CES1](http://www.ncbi.nlm.nih.gov/gene/?term=1066)(48.000)[COX7B](http://www.ncbi.nlm.nih.gov/gene/?term=1349)(48.000)[COX2](http://www.ncbi.nlm.nih.gov/gene/?term=4513)(48.000) [COX8A](http://www.ncbi.nlm.nih.gov/gene/?term=1351)(48.000)[NR1H4](http://www.ncbi.nlm.nih.gov/gene/?term=9971)(48.000) | |  |
| [N-Tricosane](http://www.megabionet.org/tcmid/ingredient/21578/) | This compound doesn't have any potential target with score larger than 20. | |  |
| [Uralsaponin B](http://www.megabionet.org/tcmid/ingredient/22228/) | [AR](http://www.ncbi.nlm.nih.gov/gene/?term=367)(48.000)[NR3C1](http://www.ncbi.nlm.nih.gov/gene/?term=2908)(48.000)[ATP1A1](http://www.ncbi.nlm.nih.gov/gene/?term=476)(48.000) | |  |
| [Guaijaverin](http://www.megabionet.org/tcmid/ingredient/9042/) | This compound doesn't have any potential target with score larger than 20. | |  |
| [Alpha-Spinasterol](http://www.megabionet.org/tcmid/ingredient/24890/) | [ESR1](http://www.ncbi.nlm.nih.gov/gene/?term=2099)(122.778)[PGR](http://www.ncbi.nlm.nih.gov/gene/?term=5241)(122.778)[VDR](http://www.ncbi.nlm.nih.gov/gene/?term=7421)(80.882)[CYP27B1](http://www.ncbi.nlm.nih.gov/gene/?term=1594)(80.882)[GC](http://www.ncbi.nlm.nih.gov/gene/?term=2638)(55.444) [SNW1](http://www.ncbi.nlm.nih.gov/gene/?term=22938)(55.444)[SRD5A1](http://www.ncbi.nlm.nih.gov/gene/?term=6715)(48.000)[AR](http://www.ncbi.nlm.nih.gov/gene/?term=367)(48.000)[NR3C1](http://www.ncbi.nlm.nih.gov/gene/?term=2908)(48.000)[ANXA1](http://www.ncbi.nlm.nih.gov/gene/?term=301)(48.000) [NFKB1](http://www.ncbi.nlm.nih.gov/gene/?term=4790)(22.373)[AKR1C3](http://www.ncbi.nlm.nih.gov/gene/?term=8644)(22.373)[CYP24A1](http://www.ncbi.nlm.nih.gov/gene/?term=1591)(22.373)[GPBAR1](http://www.ncbi.nlm.nih.gov/gene/?term=151306)(22.373)[SNAI2](http://www.ncbi.nlm.nih.gov/gene/?term=6591)(22.373) [MED1](http://www.ncbi.nlm.nih.gov/gene/?term=5469)(22.373)[SNAI1](http://www.ncbi.nlm.nih.gov/gene/?term=6615)(22.373)[CYP3A4](http://www.ncbi.nlm.nih.gov/gene/?term=1576)(22.373)[CALB1](http://www.ncbi.nlm.nih.gov/gene/?term=793)(22.373)[FGF23](http://www.ncbi.nlm.nih.gov/gene/?term=8074)(22.373) [GFI1](http://www.ncbi.nlm.nih.gov/gene/?term=2672)(22.373)[LANCL2](http://www.ncbi.nlm.nih.gov/gene/?term=55915)(22.373)[WNT4](http://www.ncbi.nlm.nih.gov/gene/?term=54361)(22.373)[TCF3](http://www.ncbi.nlm.nih.gov/gene/?term=6929)(22.373)[BAX](http://www.ncbi.nlm.nih.gov/gene/?term=581)(22.373) [KL](http://www.ncbi.nlm.nih.gov/gene/?term=9365)(22.373)[CYP2R1](http://www.ncbi.nlm.nih.gov/gene/?term=120227)(22.373)[PML](http://www.ncbi.nlm.nih.gov/gene/?term=5371)(22.373)[B4GALT1](http://www.ncbi.nlm.nih.gov/gene/?term=2683)(22.373)[S100G](http://www.ncbi.nlm.nih.gov/gene/?term=795)(22.373) [CYP27A1](http://www.ncbi.nlm.nih.gov/gene/?term=1593)(22.373)[KANK2](http://www.ncbi.nlm.nih.gov/gene/?term=25959)(22.373)[IRX5](http://www.ncbi.nlm.nih.gov/gene/?term=10265)(22.373)[RXRA](http://www.ncbi.nlm.nih.gov/gene/?term=6256)(22.373)[NR1H4](http://www.ncbi.nlm.nih.gov/gene/?term=9971)(22.373) [TRIM24](http://www.ncbi.nlm.nih.gov/gene/?term=8805)(22.373) | |  |
| [Glycyrrhisoflavone](http://www.megabionet.org/tcmid/ingredient/8843/) | This compound doesn't have any potential target with score larger than 20. | |  |
| [Alpha-Curcumene](http://www.megabionet.org/tcmid/ingredient/24308/) | [SCNN1A](http://www.ncbi.nlm.nih.gov/gene/?term=6337)(80.882)[KCNJ8](http://www.ncbi.nlm.nih.gov/gene/?term=3764)(80.882)[ADRA1A](http://www.ncbi.nlm.nih.gov/gene/?term=148)(80.882)[SCN1A](http://www.ncbi.nlm.nih.gov/gene/?term=6323)(80.882)[SCNN1G](http://www.ncbi.nlm.nih.gov/gene/?term=6340)(80.882) [HTR2A](http://www.ncbi.nlm.nih.gov/gene/?term=3356)(80.882)[ADRB2](http://www.ncbi.nlm.nih.gov/gene/?term=154)(80.882)[ACACB](http://www.ncbi.nlm.nih.gov/gene/?term=32)(80.882)[MTAP](http://www.ncbi.nlm.nih.gov/gene/?term=4507)(80.882)[ADRA2A](http://www.ncbi.nlm.nih.gov/gene/?term=150)(80.882) [TLR8](http://www.ncbi.nlm.nih.gov/gene/?term=51311)(80.882)[ADRB1](http://www.ncbi.nlm.nih.gov/gene/?term=153)(80.882)[APRT](http://www.ncbi.nlm.nih.gov/gene/?term=353)(80.882)[ADRA1D](http://www.ncbi.nlm.nih.gov/gene/?term=146)(80.882)[PECR](http://www.ncbi.nlm.nih.gov/gene/?term=55825)(80.882) [HRH1](http://www.ncbi.nlm.nih.gov/gene/?term=3269)(80.882)[PRKAA1](http://www.ncbi.nlm.nih.gov/gene/?term=5562)(80.882)[HTR7](http://www.ncbi.nlm.nih.gov/gene/?term=3363)(80.882)[HRH2](http://www.ncbi.nlm.nih.gov/gene/?term=3274)(80.882)[ADRA1B](http://www.ncbi.nlm.nih.gov/gene/?term=147)(80.882) [SCNN1D](http://www.ncbi.nlm.nih.gov/gene/?term=6339)(80.882)[ADRA2C](http://www.ncbi.nlm.nih.gov/gene/?term=152)(80.882)[AOC3](http://www.ncbi.nlm.nih.gov/gene/?term=8639)(80.882)[P4HA1](http://www.ncbi.nlm.nih.gov/gene/?term=5033)(80.882)[SRPK2](http://www.ncbi.nlm.nih.gov/gene/?term=6733)(80.882) [TLR7](http://www.ncbi.nlm.nih.gov/gene/?term=51284)(80.882)[KCNJ1](http://www.ncbi.nlm.nih.gov/gene/?term=3758)(80.882)[AGTR1](http://www.ncbi.nlm.nih.gov/gene/?term=185)(80.882)[ADRB3](http://www.ncbi.nlm.nih.gov/gene/?term=155)(80.882)[ACP1](http://www.ncbi.nlm.nih.gov/gene/?term=52)(80.882) [SCNN1B](http://www.ncbi.nlm.nih.gov/gene/?term=6338)(80.882)[CYP2B6](http://www.ncbi.nlm.nih.gov/gene/?term=1555)(80.882)[ADRA2B](http://www.ncbi.nlm.nih.gov/gene/?term=151)(80.882)[SLC6A2](http://www.ncbi.nlm.nih.gov/gene/?term=6530)(80.882)[SAXO1](http://www.ncbi.nlm.nih.gov/gene/?term=158297)(55.444) [SCT](http://www.ncbi.nlm.nih.gov/gene/?term=6343)(55.444)[UGCG](http://www.ncbi.nlm.nih.gov/gene/?term=7357)(55.444)[PHKG2](http://www.ncbi.nlm.nih.gov/gene/?term=5261)(55.444)[APLP1](http://www.ncbi.nlm.nih.gov/gene/?term=333)(55.444)[ADK](http://www.ncbi.nlm.nih.gov/gene/?term=132)(55.444) [ACACA](http://www.ncbi.nlm.nih.gov/gene/?term=31)(55.444)[CHRNA7](http://www.ncbi.nlm.nih.gov/gene/?term=1139)(55.444)[PNP](http://www.ncbi.nlm.nih.gov/gene/?term=4860)(55.444)[ARRB2](http://www.ncbi.nlm.nih.gov/gene/?term=409)(55.444)[RAB7A](http://www.ncbi.nlm.nih.gov/gene/?term=7879)(55.444) [PRKAA2](http://www.ncbi.nlm.nih.gov/gene/?term=5563)(55.444)[HTR2C](http://www.ncbi.nlm.nih.gov/gene/?term=3358)(55.444)[HSP90AB1](http://www.ncbi.nlm.nih.gov/gene/?term=3326)(55.444)[ALDH3A2](http://www.ncbi.nlm.nih.gov/gene/?term=224)(55.444)[INS](http://www.ncbi.nlm.nih.gov/gene/?term=3630)(55.444) [CALCOCO2](http://www.ncbi.nlm.nih.gov/gene/?term=10241)(55.444)[SLC22A1](http://www.ncbi.nlm.nih.gov/gene/?term=6580)(55.444)[AOC2](http://www.ncbi.nlm.nih.gov/gene/?term=314)(55.444)[ARRDC3](http://www.ncbi.nlm.nih.gov/gene/?term=57561)(55.444)[CALCA](http://www.ncbi.nlm.nih.gov/gene/?term=796)(55.444) [HPRT1](http://www.ncbi.nlm.nih.gov/gene/?term=3251)(55.444)[ACTN2](http://www.ncbi.nlm.nih.gov/gene/?term=88)(55.444)[SCN3A](http://www.ncbi.nlm.nih.gov/gene/?term=6328)(23.000)[SCN2A](http://www.ncbi.nlm.nih.gov/gene/?term=6326)(23.000)[ADCY6](http://www.ncbi.nlm.nih.gov/gene/?term=112)(22.373) [ACTN3](http://www.ncbi.nlm.nih.gov/gene/?term=89)(22.373)[RSAD2](http://www.ncbi.nlm.nih.gov/gene/?term=91543)(22.373)[C1QTNF1](http://www.ncbi.nlm.nih.gov/gene/?term=114897)(22.373)[APIP](http://www.ncbi.nlm.nih.gov/gene/?term=51074)(22.373)[ORMDL1](http://www.ncbi.nlm.nih.gov/gene/?term=94101)(22.373) [UTS2](http://www.ncbi.nlm.nih.gov/gene/?term=10911)(22.373)[NAV2](http://www.ncbi.nlm.nih.gov/gene/?term=89797)(22.373)[GBA2](http://www.ncbi.nlm.nih.gov/gene/?term=57704)(22.373)[PCCA](http://www.ncbi.nlm.nih.gov/gene/?term=5095)(22.373)[ANG](http://www.ncbi.nlm.nih.gov/gene/?term=283)(22.373) [P2RX2](http://www.ncbi.nlm.nih.gov/gene/?term=22953)(22.373)[GAA](http://www.ncbi.nlm.nih.gov/gene/?term=2548)(22.373)[GBA](http://www.ncbi.nlm.nih.gov/gene/?term=2629)(22.373)[GSK3A](http://www.ncbi.nlm.nih.gov/gene/?term=2931)(22.373)[FGF1](http://www.ncbi.nlm.nih.gov/gene/?term=2246)(22.373) [OXT](http://www.ncbi.nlm.nih.gov/gene/?term=5020)(22.373)[PC](http://www.ncbi.nlm.nih.gov/gene/?term=5091)(22.373)[DLL1](http://www.ncbi.nlm.nih.gov/gene/?term=28514)(22.373)[KLHL3](http://www.ncbi.nlm.nih.gov/gene/?term=26249)(22.373)[RXFP4](http://www.ncbi.nlm.nih.gov/gene/?term=339403)(22.373) [HTR2B](http://www.ncbi.nlm.nih.gov/gene/?term=3357)(22.373)[WNK4](http://www.ncbi.nlm.nih.gov/gene/?term=65266)(22.373)[LHCGR](http://www.ncbi.nlm.nih.gov/gene/?term=3973)(22.373)[CETN2](http://www.ncbi.nlm.nih.gov/gene/?term=1069)(22.373)[CETN1](http://www.ncbi.nlm.nih.gov/gene/?term=1068)(22.373) [TLR3](http://www.ncbi.nlm.nih.gov/gene/?term=7098)(22.373)[SPIDR](http://www.ncbi.nlm.nih.gov/gene/?term=23514)(22.373)[SNX3](http://www.ncbi.nlm.nih.gov/gene/?term=8724)(22.373)[VCAM1](http://www.ncbi.nlm.nih.gov/gene/?term=7412)(22.373)[TRPV1](http://www.ncbi.nlm.nih.gov/gene/?term=7442)(22.373) [ADI1](http://www.ncbi.nlm.nih.gov/gene/?term=55256)(22.373)[BLM](http://www.ncbi.nlm.nih.gov/gene/?term=641)(22.373)[AGT](http://www.ncbi.nlm.nih.gov/gene/?term=183)(22.373)[CECR1](http://www.ncbi.nlm.nih.gov/gene/?term=51816)(22.373)[GOT1](http://www.ncbi.nlm.nih.gov/gene/?term=2805)(22.373) [HTR6](http://www.ncbi.nlm.nih.gov/gene/?term=3362)(22.373)[ENOPH1](http://www.ncbi.nlm.nih.gov/gene/?term=58478)(22.373)[CLN3](http://www.ncbi.nlm.nih.gov/gene/?term=1201)(22.373)[POR](http://www.ncbi.nlm.nih.gov/gene/?term=5447)(22.373)[PTK2B](http://www.ncbi.nlm.nih.gov/gene/?term=2185)(22.373) [MAS1](http://www.ncbi.nlm.nih.gov/gene/?term=4142)(22.373)[BMP6](http://www.ncbi.nlm.nih.gov/gene/?term=654)(22.373)[ADRBK1](http://www.ncbi.nlm.nih.gov/gene/?term=156)(22.373)[MAOB](http://www.ncbi.nlm.nih.gov/gene/?term=4129)(22.373)[MCCC1](http://www.ncbi.nlm.nih.gov/gene/?term=56922)(22.373) [P2RX3](http://www.ncbi.nlm.nih.gov/gene/?term=5024)(22.373)[KCNJ11](http://www.ncbi.nlm.nih.gov/gene/?term=3767)(22.373)[SERPINF2](http://www.ncbi.nlm.nih.gov/gene/?term=5345)(22.373)[MCCC2](http://www.ncbi.nlm.nih.gov/gene/?term=64087)(22.373)[UCHL1](http://www.ncbi.nlm.nih.gov/gene/?term=7345)(22.373) [PDE4D](http://www.ncbi.nlm.nih.gov/gene/?term=5144)(22.373)[MZB1](http://www.ncbi.nlm.nih.gov/gene/?term=51237)(22.373)[HRH3](http://www.ncbi.nlm.nih.gov/gene/?term=11255)(22.373)[P2RY1](http://www.ncbi.nlm.nih.gov/gene/?term=5028)(22.373)[AGTRAP](http://www.ncbi.nlm.nih.gov/gene/?term=57085)(22.373) [ORMDL2](http://www.ncbi.nlm.nih.gov/gene/?term=29095)(22.373)[SGK1](http://www.ncbi.nlm.nih.gov/gene/?term=6446)(22.373)[SREBF1](http://www.ncbi.nlm.nih.gov/gene/?term=6720)(22.373)[GAL3ST1](http://www.ncbi.nlm.nih.gov/gene/?term=9514)(22.373)[PLA2G1B](http://www.ncbi.nlm.nih.gov/gene/?term=5319)(22.373) [DPYS](http://www.ncbi.nlm.nih.gov/gene/?term=1807)(22.373)[MRI1](http://www.ncbi.nlm.nih.gov/gene/?term=84245)(22.373)[MSTN](http://www.ncbi.nlm.nih.gov/gene/?term=2660)(22.373)[CACNA1C](http://www.ncbi.nlm.nih.gov/gene/?term=775)(22.373)[AOC1](http://www.ncbi.nlm.nih.gov/gene/?term=26)(22.373) [MAOA](http://www.ncbi.nlm.nih.gov/gene/?term=4128)(22.373)[TYMP](http://www.ncbi.nlm.nih.gov/gene/?term=1890)(22.373)[UTS2R](http://www.ncbi.nlm.nih.gov/gene/?term=2837)(22.373)[ARRB1](http://www.ncbi.nlm.nih.gov/gene/?term=408)(22.373)[TNNC1](http://www.ncbi.nlm.nih.gov/gene/?term=7134)(22.373) [NCOA3](http://www.ncbi.nlm.nih.gov/gene/?term=8202)(22.373)[HRH4](http://www.ncbi.nlm.nih.gov/gene/?term=59340)(22.373)[NMUR2](http://www.ncbi.nlm.nih.gov/gene/?term=56923)(22.373)[TLR9](http://www.ncbi.nlm.nih.gov/gene/?term=54106)(22.373)[ACE](http://www.ncbi.nlm.nih.gov/gene/?term=1636)(22.373) [UGT8](http://www.ncbi.nlm.nih.gov/gene/?term=7368)(22.373)[AGTR2](http://www.ncbi.nlm.nih.gov/gene/?term=186)(22.373)[PYGM](http://www.ncbi.nlm.nih.gov/gene/?term=5837)(22.373)[ABCC9](http://www.ncbi.nlm.nih.gov/gene/?term=10060)(22.373)[CAV1](http://www.ncbi.nlm.nih.gov/gene/?term=857)(22.373) [PYGL](http://www.ncbi.nlm.nih.gov/gene/?term=5836)(22.373)[AVPR2](http://www.ncbi.nlm.nih.gov/gene/?term=554)(22.373)[KCNJ10](http://www.ncbi.nlm.nih.gov/gene/?term=3766)(22.373)[IL10](http://www.ncbi.nlm.nih.gov/gene/?term=3586)(22.373)[GPR101](http://www.ncbi.nlm.nih.gov/gene/?term=83550)(22.373) [PID1](http://www.ncbi.nlm.nih.gov/gene/?term=55022)(22.373)[DECR2](http://www.ncbi.nlm.nih.gov/gene/?term=26063)(22.373)[REN](http://www.ncbi.nlm.nih.gov/gene/?term=5972)(22.373)[PDGFA](http://www.ncbi.nlm.nih.gov/gene/?term=5154)(22.373)[MLYCD](http://www.ncbi.nlm.nih.gov/gene/?term=23417)(22.373) [NMI](http://www.ncbi.nlm.nih.gov/gene/?term=9111)(22.373)[PIK3AP1](http://www.ncbi.nlm.nih.gov/gene/?term=118788)(22.373)[SEC14L2](http://www.ncbi.nlm.nih.gov/gene/?term=23541)(22.373)[DRD5](http://www.ncbi.nlm.nih.gov/gene/?term=1816)(22.373)[ZNF219](http://www.ncbi.nlm.nih.gov/gene/?term=51222)(22.373) [RFK](http://www.ncbi.nlm.nih.gov/gene/?term=55312)(22.373)[ZP3](http://www.ncbi.nlm.nih.gov/gene/?term=7784)(22.373)[PCCB](http://www.ncbi.nlm.nih.gov/gene/?term=5096)(22.373)[ATP2B4](http://www.ncbi.nlm.nih.gov/gene/?term=493)(22.373)[P4HA3](http://www.ncbi.nlm.nih.gov/gene/?term=283208)(22.373) [SLC9A3R1](http://www.ncbi.nlm.nih.gov/gene/?term=9368)(22.373)[NACA](http://www.ncbi.nlm.nih.gov/gene/?term=4666)(22.373)[ADA](http://www.ncbi.nlm.nih.gov/gene/?term=100)(22.373)[POU1F1](http://www.ncbi.nlm.nih.gov/gene/?term=5449)(22.373)[KCNMA1](http://www.ncbi.nlm.nih.gov/gene/?term=3778)(22.373) [GPER1](http://www.ncbi.nlm.nih.gov/gene/?term=2852)(22.373)[KCNE2](http://www.ncbi.nlm.nih.gov/gene/?term=9992)(22.373)[ADORA1](http://www.ncbi.nlm.nih.gov/gene/?term=134)(22.373)[ADAM17](http://www.ncbi.nlm.nih.gov/gene/?term=6868)(22.373)[P4HB](http://www.ncbi.nlm.nih.gov/gene/?term=5034)(22.373) [NOS1](http://www.ncbi.nlm.nih.gov/gene/?term=4842)(22.373)[RGS2](http://www.ncbi.nlm.nih.gov/gene/?term=5997)(22.373)[TRPM8](http://www.ncbi.nlm.nih.gov/gene/?term=79054)(22.373)[UNC93B1](http://www.ncbi.nlm.nih.gov/gene/?term=81622)(22.373)[PDGFB](http://www.ncbi.nlm.nih.gov/gene/?term=5155)(22.373) [P4HA2](http://www.ncbi.nlm.nih.gov/gene/?term=8974)(22.373)[GDPGP1](http://www.ncbi.nlm.nih.gov/gene/?term=390637)(22.373)[EGFR](http://www.ncbi.nlm.nih.gov/gene/?term=1956)(22.373)[MECR](http://www.ncbi.nlm.nih.gov/gene/?term=51102)(22.373)[TPM1](http://www.ncbi.nlm.nih.gov/gene/?term=7168)(22.373) [RPS6KB1](http://www.ncbi.nlm.nih.gov/gene/?term=6198)(22.373)[EDN2](http://www.ncbi.nlm.nih.gov/gene/?term=1907)(22.373)[SYK](http://www.ncbi.nlm.nih.gov/gene/?term=6850)(22.373)[PYGB](http://www.ncbi.nlm.nih.gov/gene/?term=5834)(22.373)[PRKCD](http://www.ncbi.nlm.nih.gov/gene/?term=5580)(22.373) [ABCG1](http://www.ncbi.nlm.nih.gov/gene/?term=9619)(22.373)[RAD51](http://www.ncbi.nlm.nih.gov/gene/?term=5888)(22.373)[PAICS](http://www.ncbi.nlm.nih.gov/gene/?term=10606)(22.373)[ATP8A2](http://www.ncbi.nlm.nih.gov/gene/?term=51761)(22.373)[TRPA1](http://www.ncbi.nlm.nih.gov/gene/?term=8989)(22.373) | |  |
| [Uralenol-3-Methylether](http://www.megabionet.org/tcmid/ingredient/22226/) | This compound doesn't have any potential target with score larger than 20. | |  |
| [Liquiritin](http://www.megabionet.org/tcmid/ingredient/12907/) | [SOAT1](http://www.ncbi.nlm.nih.gov/gene/?term=6646)(48.000)[MTTP](http://www.ncbi.nlm.nih.gov/gene/?term=4547)(48.000)[SOAT2](http://www.ncbi.nlm.nih.gov/gene/?term=8435)(48.000) | |  |
| [Beta-Curcumene](http://www.megabionet.org/tcmid/ingredient/30843/) | [KCND1](http://www.ncbi.nlm.nih.gov/gene/?term=3750)(80.882)[KCNA3](http://www.ncbi.nlm.nih.gov/gene/?term=3738)(80.882)[PRKAB1](http://www.ncbi.nlm.nih.gov/gene/?term=5564)(80.882)[ADH1A](http://www.ncbi.nlm.nih.gov/gene/?term=124)(80.882)[KCNA10](http://www.ncbi.nlm.nih.gov/gene/?term=3744)(80.882) [GAMT](http://www.ncbi.nlm.nih.gov/gene/?term=2593)(80.882)[KCNC3](http://www.ncbi.nlm.nih.gov/gene/?term=3748)(80.882)[KCNA1](http://www.ncbi.nlm.nih.gov/gene/?term=3736)(80.882)[KCNA2](http://www.ncbi.nlm.nih.gov/gene/?term=3737)(80.882)[TPO](http://www.ncbi.nlm.nih.gov/gene/?term=7173)(80.882) [CAT](http://www.ncbi.nlm.nih.gov/gene/?term=847)(80.882)[KCNB1](http://www.ncbi.nlm.nih.gov/gene/?term=3745)(80.882)[ADH1B](http://www.ncbi.nlm.nih.gov/gene/?term=125)(80.882)[DLG4](http://www.ncbi.nlm.nih.gov/gene/?term=1742)(80.882)[KCNC2](http://www.ncbi.nlm.nih.gov/gene/?term=3747)(80.882) [KCNC1](http://www.ncbi.nlm.nih.gov/gene/?term=3746)(80.882)[ADH1C](http://www.ncbi.nlm.nih.gov/gene/?term=126)(80.882)[KCNB2](http://www.ncbi.nlm.nih.gov/gene/?term=9312)(80.882)[KCNA5](http://www.ncbi.nlm.nih.gov/gene/?term=3741)(80.882)[RNASE1](http://www.ncbi.nlm.nih.gov/gene/?term=6035)(80.882) [KCND2](http://www.ncbi.nlm.nih.gov/gene/?term=3751)(80.882)[ALDH2](http://www.ncbi.nlm.nih.gov/gene/?term=217)(80.882)[KCNA4](http://www.ncbi.nlm.nih.gov/gene/?term=3739)(80.882)[KCNA7](http://www.ncbi.nlm.nih.gov/gene/?term=3743)(80.882)[KCNA6](http://www.ncbi.nlm.nih.gov/gene/?term=3742)(80.882) [KCND3](http://www.ncbi.nlm.nih.gov/gene/?term=3752)(80.882)[GUCY1B3](http://www.ncbi.nlm.nih.gov/gene/?term=2983)(55.444)[GATM](http://www.ncbi.nlm.nih.gov/gene/?term=2628)(55.444)[KCNK4](http://www.ncbi.nlm.nih.gov/gene/?term=50801)(55.444)[IYD](http://www.ncbi.nlm.nih.gov/gene/?term=389434)(55.444) [KCNQ1](http://www.ncbi.nlm.nih.gov/gene/?term=3784)(55.444)[FBP1](http://www.ncbi.nlm.nih.gov/gene/?term=2203)(22.373)[DGKI](http://www.ncbi.nlm.nih.gov/gene/?term=9162)(22.373)[ADH7](http://www.ncbi.nlm.nih.gov/gene/?term=131)(22.373)[NFIB](http://www.ncbi.nlm.nih.gov/gene/?term=4781)(22.373) [CYGB](http://www.ncbi.nlm.nih.gov/gene/?term=114757)(22.373)[NRXN3](http://www.ncbi.nlm.nih.gov/gene/?term=9369)(22.373)[LRRC4B](http://www.ncbi.nlm.nih.gov/gene/?term=94030)(22.373)[AQP8](http://www.ncbi.nlm.nih.gov/gene/?term=343)(22.373)[ARX](http://www.ncbi.nlm.nih.gov/gene/?term=170302)(22.373) [IGF1](http://www.ncbi.nlm.nih.gov/gene/?term=3479)(22.373)[SHANK3](http://www.ncbi.nlm.nih.gov/gene/?term=85358)(22.373)[ACY3](http://www.ncbi.nlm.nih.gov/gene/?term=91703)(22.373)[ASPA](http://www.ncbi.nlm.nih.gov/gene/?term=443)(22.373)[NRXN1](http://www.ncbi.nlm.nih.gov/gene/?term=9378)(22.373) [EDN1](http://www.ncbi.nlm.nih.gov/gene/?term=1906)(22.373)[OXT](http://www.ncbi.nlm.nih.gov/gene/?term=5020)(22.373)[MAGI2](http://www.ncbi.nlm.nih.gov/gene/?term=9863)(22.373)[APOE](http://www.ncbi.nlm.nih.gov/gene/?term=348)(22.373)[SOX15](http://www.ncbi.nlm.nih.gov/gene/?term=6665)(22.373) [CNTNAP4](http://www.ncbi.nlm.nih.gov/gene/?term=85445)(22.373)[RNASE4](http://www.ncbi.nlm.nih.gov/gene/?term=6038)(22.373)[RAB3A](http://www.ncbi.nlm.nih.gov/gene/?term=5864)(22.373)[MC4R](http://www.ncbi.nlm.nih.gov/gene/?term=4160)(22.373)[IL1B](http://www.ncbi.nlm.nih.gov/gene/?term=3553)(22.373) [GRIN2A](http://www.ncbi.nlm.nih.gov/gene/?term=2903)(22.373)[SLC17A7](http://www.ncbi.nlm.nih.gov/gene/?term=57030)(22.373)[CYP11A1](http://www.ncbi.nlm.nih.gov/gene/?term=1583)(22.373)[SCN5A](http://www.ncbi.nlm.nih.gov/gene/?term=6331)(22.373)[SCN10A](http://www.ncbi.nlm.nih.gov/gene/?term=6336)(22.373) [SPARC](http://www.ncbi.nlm.nih.gov/gene/?term=6678)(22.373)[SORCS3](http://www.ncbi.nlm.nih.gov/gene/?term=22986)(22.373)[RNASE8](http://www.ncbi.nlm.nih.gov/gene/?term=122665)(22.373)[KCNIP2](http://www.ncbi.nlm.nih.gov/gene/?term=30819)(22.373)[GPX7](http://www.ncbi.nlm.nih.gov/gene/?term=2882)(22.373) [CRLF1](http://www.ncbi.nlm.nih.gov/gene/?term=9244)(22.373)[FADD](http://www.ncbi.nlm.nih.gov/gene/?term=8772)(22.373)[SNTG2](http://www.ncbi.nlm.nih.gov/gene/?term=54221)(22.373)[SCGB1A1](http://www.ncbi.nlm.nih.gov/gene/?term=7356)(22.373)[OXTR](http://www.ncbi.nlm.nih.gov/gene/?term=5021)(22.373) [ALDH1B1](http://www.ncbi.nlm.nih.gov/gene/?term=219)(22.373)[LEP](http://www.ncbi.nlm.nih.gov/gene/?term=3952)(22.373)[NLGN1](http://www.ncbi.nlm.nih.gov/gene/?term=22871)(22.373)[CACNA1D](http://www.ncbi.nlm.nih.gov/gene/?term=776)(22.373)[RAPGEF2](http://www.ncbi.nlm.nih.gov/gene/?term=9693)(22.373) [DARS](http://www.ncbi.nlm.nih.gov/gene/?term=1615)(22.373)[ANK3](http://www.ncbi.nlm.nih.gov/gene/?term=288)(22.373)[KCNE5](http://www.ncbi.nlm.nih.gov/gene/?term=23630)(22.373)[PPARD](http://www.ncbi.nlm.nih.gov/gene/?term=5467)(22.373)[NKX2-1](http://www.ncbi.nlm.nih.gov/gene/?term=7080)(22.373) [MIP](http://www.ncbi.nlm.nih.gov/gene/?term=4284)(22.373)[RYR3](http://www.ncbi.nlm.nih.gov/gene/?term=6263)(22.373)[ZPR1](http://www.ncbi.nlm.nih.gov/gene/?term=8882)(22.373)[ALDH3B1](http://www.ncbi.nlm.nih.gov/gene/?term=221)(22.373)[NPPA](http://www.ncbi.nlm.nih.gov/gene/?term=4878)(22.373) [STX1A](http://www.ncbi.nlm.nih.gov/gene/?term=6804)(22.373)[PAXBP1](http://www.ncbi.nlm.nih.gov/gene/?term=94104)(22.373)[PAX7](http://www.ncbi.nlm.nih.gov/gene/?term=5081)(22.373)[ALDH3B2](http://www.ncbi.nlm.nih.gov/gene/?term=222)(22.373)[RNASE2](http://www.ncbi.nlm.nih.gov/gene/?term=6036)(22.373) [NRXN2](http://www.ncbi.nlm.nih.gov/gene/?term=9379)(22.373)[ADORA1](http://www.ncbi.nlm.nih.gov/gene/?term=134)(22.373)[ADH4](http://www.ncbi.nlm.nih.gov/gene/?term=127)(22.373)[GJA5](http://www.ncbi.nlm.nih.gov/gene/?term=2702)(22.373)[FAS](http://www.ncbi.nlm.nih.gov/gene/?term=355)(22.373) [DAB2IP](http://www.ncbi.nlm.nih.gov/gene/?term=153090)(22.373)[CYP2E1](http://www.ncbi.nlm.nih.gov/gene/?term=1571)(22.373)[ACY1](http://www.ncbi.nlm.nih.gov/gene/?term=95)(22.373) | |  |
| [Hexanoic Acid](http://www.megabionet.org/tcmid/ingredient/23052/) | [CHRM1](http://www.ncbi.nlm.nih.gov/gene/?term=1128)(48.000)[CHRM2](http://www.ncbi.nlm.nih.gov/gene/?term=1129)(48.000) | |  |
| [Liquiritigenin](http://www.megabionet.org/tcmid/ingredient/12902/) | [SOAT1](http://www.ncbi.nlm.nih.gov/gene/?term=6646)(122.778)[MTTP](http://www.ncbi.nlm.nih.gov/gene/?term=4547)(122.778)[SOAT2](http://www.ncbi.nlm.nih.gov/gene/?term=8435)(122.778)[CNR2](http://www.ncbi.nlm.nih.gov/gene/?term=1269)(80.882)[CNR1](http://www.ncbi.nlm.nih.gov/gene/?term=1268)(80.882) [DRD2](http://www.ncbi.nlm.nih.gov/gene/?term=1813)(55.444)[PAWR](http://www.ncbi.nlm.nih.gov/gene/?term=5074)(55.444)[AKT1](http://www.ncbi.nlm.nih.gov/gene/?term=207)(22.373)[CD36](http://www.ncbi.nlm.nih.gov/gene/?term=948)(22.373)[CCR7](http://www.ncbi.nlm.nih.gov/gene/?term=1236)(22.373) [ABHD6](http://www.ncbi.nlm.nih.gov/gene/?term=57406)(22.373)[MGLL](http://www.ncbi.nlm.nih.gov/gene/?term=11343)(22.373)[FCER1G](http://www.ncbi.nlm.nih.gov/gene/?term=2207)(22.373)[SUMO1](http://www.ncbi.nlm.nih.gov/gene/?term=7341)(22.373)[APOH](http://www.ncbi.nlm.nih.gov/gene/?term=350)(22.373) [GPR55](http://www.ncbi.nlm.nih.gov/gene/?term=9290)(22.373)[CHRNB2](http://www.ncbi.nlm.nih.gov/gene/?term=1141)(22.373)[CETP](http://www.ncbi.nlm.nih.gov/gene/?term=1071)(22.373)[FCER1A](http://www.ncbi.nlm.nih.gov/gene/?term=2205)(22.373)[CAV3](http://www.ncbi.nlm.nih.gov/gene/?term=859)(22.373) [RNF207](http://www.ncbi.nlm.nih.gov/gene/?term=388591)(22.373)[C3](http://www.ncbi.nlm.nih.gov/gene/?term=718)(22.373)[DAGLA](http://www.ncbi.nlm.nih.gov/gene/?term=747)(22.373)[PLIN5](http://www.ncbi.nlm.nih.gov/gene/?term=440503)(22.373)[ZP3](http://www.ncbi.nlm.nih.gov/gene/?term=7784)(22.373) | |  |
| [3-Methyl-6,7,8-Trihydropyrrolo[1,2-A]Pyrimidin-2-One](http://www.megabionet.org/tcmid/ingredient/14766/) | [PDE7B](http://www.ncbi.nlm.nih.gov/gene/?term=27115)(80.882)[PDE5A](http://www.ncbi.nlm.nih.gov/gene/?term=8654)(80.882)[PDE9A](http://www.ncbi.nlm.nih.gov/gene/?term=5152)(80.882)[PDE4A](http://www.ncbi.nlm.nih.gov/gene/?term=5141)(80.882)[RYR1](http://www.ncbi.nlm.nih.gov/gene/?term=6261)(80.882) [CYP17A1](http://www.ncbi.nlm.nih.gov/gene/?term=1586)(80.882)[PDE3A](http://www.ncbi.nlm.nih.gov/gene/?term=5139)(80.882)[ADORA2A](http://www.ncbi.nlm.nih.gov/gene/?term=135)(80.882)[ITPR1](http://www.ncbi.nlm.nih.gov/gene/?term=3708)(80.882)[PDE6A](http://www.ncbi.nlm.nih.gov/gene/?term=5145)(80.882) [PRKDC](http://www.ncbi.nlm.nih.gov/gene/?term=5591)(80.882)[PIK3CD](http://www.ncbi.nlm.nih.gov/gene/?term=5293)(80.882)[ESR1](http://www.ncbi.nlm.nih.gov/gene/?term=2099)(80.882)[PDE3B](http://www.ncbi.nlm.nih.gov/gene/?term=5140)(80.882)[PGD](http://www.ncbi.nlm.nih.gov/gene/?term=5226)(80.882) [PIK3CA](http://www.ncbi.nlm.nih.gov/gene/?term=5290)(80.882)[ADORA2B](http://www.ncbi.nlm.nih.gov/gene/?term=136)(80.882)[PDE1A](http://www.ncbi.nlm.nih.gov/gene/?term=5136)(80.882)[PDE4D](http://www.ncbi.nlm.nih.gov/gene/?term=5144)(80.882)[PGR](http://www.ncbi.nlm.nih.gov/gene/?term=5241)(80.882) [PIK3CB](http://www.ncbi.nlm.nih.gov/gene/?term=5291)(80.882)[ITPR2](http://www.ncbi.nlm.nih.gov/gene/?term=3709)(80.882)[PDE1B](http://www.ncbi.nlm.nih.gov/gene/?term=5153)(80.882)[OPRK1](http://www.ncbi.nlm.nih.gov/gene/?term=4986)(80.882)[PDE7A](http://www.ncbi.nlm.nih.gov/gene/?term=5150)(80.882) [ITPR3](http://www.ncbi.nlm.nih.gov/gene/?term=3710)(80.882)[POLA2](http://www.ncbi.nlm.nih.gov/gene/?term=23649)(80.882)[PDE4C](http://www.ncbi.nlm.nih.gov/gene/?term=5143)(80.882)[PDE10A](http://www.ncbi.nlm.nih.gov/gene/?term=10846)(80.882)[PDE1C](http://www.ncbi.nlm.nih.gov/gene/?term=5137)(80.882) [ATM](http://www.ncbi.nlm.nih.gov/gene/?term=472)(80.882)[PDE6B](http://www.ncbi.nlm.nih.gov/gene/?term=5158)(80.882)[PDE4B](http://www.ncbi.nlm.nih.gov/gene/?term=5142)(80.882)[PDE2A](http://www.ncbi.nlm.nih.gov/gene/?term=5138)(80.882)[ADORA1](http://www.ncbi.nlm.nih.gov/gene/?term=134)(80.882) [CYP19A1](http://www.ncbi.nlm.nih.gov/gene/?term=1588)(80.882)[PDE8B](http://www.ncbi.nlm.nih.gov/gene/?term=8622)(80.882)[NT5E](http://www.ncbi.nlm.nih.gov/gene/?term=4907)(80.882)[HDAC2](http://www.ncbi.nlm.nih.gov/gene/?term=3066)(80.882)[PDE6C](http://www.ncbi.nlm.nih.gov/gene/?term=5146)(80.882) [PDE11A](http://www.ncbi.nlm.nih.gov/gene/?term=50940)(80.882)[PDE8A](http://www.ncbi.nlm.nih.gov/gene/?term=5151)(80.882)[NR3C2](http://www.ncbi.nlm.nih.gov/gene/?term=4306)(80.882)[RINT1](http://www.ncbi.nlm.nih.gov/gene/?term=60561)(55.444)[RIPK1](http://www.ncbi.nlm.nih.gov/gene/?term=8737)(55.444) [PIK3R1](http://www.ncbi.nlm.nih.gov/gene/?term=5295)(55.444)[WNT4](http://www.ncbi.nlm.nih.gov/gene/?term=54361)(55.444)[TACR2](http://www.ncbi.nlm.nih.gov/gene/?term=6865)(55.444)[AMPD3](http://www.ncbi.nlm.nih.gov/gene/?term=272)(55.444)[CX3CR1](http://www.ncbi.nlm.nih.gov/gene/?term=1524)(55.444) [ADA](http://www.ncbi.nlm.nih.gov/gene/?term=100)(55.444)[HAP1](http://www.ncbi.nlm.nih.gov/gene/?term=9001)(55.444)[IDNK](http://www.ncbi.nlm.nih.gov/gene/?term=414328)(55.444)[HDAC1](http://www.ncbi.nlm.nih.gov/gene/?term=3065)(23.000)[TFAP2C](http://www.ncbi.nlm.nih.gov/gene/?term=7022)(22.373) [HMGA2](http://www.ncbi.nlm.nih.gov/gene/?term=8091)(22.373)[TNFSF11](http://www.ncbi.nlm.nih.gov/gene/?term=8600)(22.373)[NT5C1A](http://www.ncbi.nlm.nih.gov/gene/?term=84618)(22.373)[ACTN3](http://www.ncbi.nlm.nih.gov/gene/?term=89)(22.373)[NOS1AP](http://www.ncbi.nlm.nih.gov/gene/?term=9722)(22.373) [TAC1](http://www.ncbi.nlm.nih.gov/gene/?term=6863)(22.373)[PHB](http://www.ncbi.nlm.nih.gov/gene/?term=5245)(22.373)[SIRT1](http://www.ncbi.nlm.nih.gov/gene/?term=23411)(22.373)[DGKI](http://www.ncbi.nlm.nih.gov/gene/?term=9162)(22.373)[FKBP1A](http://www.ncbi.nlm.nih.gov/gene/?term=2280)(22.373) [FURIN](http://www.ncbi.nlm.nih.gov/gene/?term=5045)(22.373)[TOX3](http://www.ncbi.nlm.nih.gov/gene/?term=27324)(22.373)[UTS2](http://www.ncbi.nlm.nih.gov/gene/?term=10911)(22.373)[STUB1](http://www.ncbi.nlm.nih.gov/gene/?term=10273)(22.373)[CTR9](http://www.ncbi.nlm.nih.gov/gene/?term=9646)(22.373) [G6PD](http://www.ncbi.nlm.nih.gov/gene/?term=2539)(22.373)[GNAT1](http://www.ncbi.nlm.nih.gov/gene/?term=2779)(22.373)[SIX1](http://www.ncbi.nlm.nih.gov/gene/?term=6495)(22.373)[QDPR](http://www.ncbi.nlm.nih.gov/gene/?term=5860)(22.373)[TGFB1](http://www.ncbi.nlm.nih.gov/gene/?term=7040)(22.373) [ATP11C](http://www.ncbi.nlm.nih.gov/gene/?term=286410)(22.373)[KHDRBS1](http://www.ncbi.nlm.nih.gov/gene/?term=10657)(22.373)[PPP3CB](http://www.ncbi.nlm.nih.gov/gene/?term=5532)(22.373)[SYT2](http://www.ncbi.nlm.nih.gov/gene/?term=127833)(22.373)[SIX4](http://www.ncbi.nlm.nih.gov/gene/?term=51804)(22.373) [SIRT2](http://www.ncbi.nlm.nih.gov/gene/?term=22933)(22.373)[SPI1](http://www.ncbi.nlm.nih.gov/gene/?term=6688)(22.373)[HOMER1](http://www.ncbi.nlm.nih.gov/gene/?term=9456)(22.373)[SHANK3](http://www.ncbi.nlm.nih.gov/gene/?term=85358)(22.373)[TAS1R1](http://www.ncbi.nlm.nih.gov/gene/?term=80835)(22.373) [TRIM28](http://www.ncbi.nlm.nih.gov/gene/?term=10155)(22.373)[XRCC4](http://www.ncbi.nlm.nih.gov/gene/?term=7518)(22.373)[MED1](http://www.ncbi.nlm.nih.gov/gene/?term=5469)(22.373)[NKX3-1](http://www.ncbi.nlm.nih.gov/gene/?term=4824)(22.373)[UBE2B](http://www.ncbi.nlm.nih.gov/gene/?term=7320)(22.373) [ESR2](http://www.ncbi.nlm.nih.gov/gene/?term=2100)(22.373)[HCN4](http://www.ncbi.nlm.nih.gov/gene/?term=10021)(22.373)[EDN1](http://www.ncbi.nlm.nih.gov/gene/?term=1906)(22.373)[CACNA1A](http://www.ncbi.nlm.nih.gov/gene/?term=773)(22.373)[STAT5A](http://www.ncbi.nlm.nih.gov/gene/?term=6776)(22.373) [TP53](http://www.ncbi.nlm.nih.gov/gene/?term=7157)(22.373)[GNAT3](http://www.ncbi.nlm.nih.gov/gene/?term=346562)(22.373)[HELB](http://www.ncbi.nlm.nih.gov/gene/?term=92797)(22.373)[COMT](http://www.ncbi.nlm.nih.gov/gene/?term=1312)(22.373)[SLC26A6](http://www.ncbi.nlm.nih.gov/gene/?term=65010)(22.373) [ADRA2A](http://www.ncbi.nlm.nih.gov/gene/?term=150)(22.373)[YWHAE](http://www.ncbi.nlm.nih.gov/gene/?term=7531)(22.373)[CETN2](http://www.ncbi.nlm.nih.gov/gene/?term=1069)(22.373)[POLA1](http://www.ncbi.nlm.nih.gov/gene/?term=5422)(22.373)[CRP](http://www.ncbi.nlm.nih.gov/gene/?term=1401)(22.373) [CETN1](http://www.ncbi.nlm.nih.gov/gene/?term=1068)(22.373)[NUDT9](http://www.ncbi.nlm.nih.gov/gene/?term=53343)(22.373)[PIK3CG](http://www.ncbi.nlm.nih.gov/gene/?term=5294)(22.373)[BMP2](http://www.ncbi.nlm.nih.gov/gene/?term=650)(22.373)[TNF](http://www.ncbi.nlm.nih.gov/gene/?term=7124)(22.373) [NEDD4](http://www.ncbi.nlm.nih.gov/gene/?term=4734)(22.373)[AGT](http://www.ncbi.nlm.nih.gov/gene/?term=183)(22.373)[FER](http://www.ncbi.nlm.nih.gov/gene/?term=2241)(22.373)[SHH](http://www.ncbi.nlm.nih.gov/gene/?term=6469)(22.373)[SERPINB3](http://www.ncbi.nlm.nih.gov/gene/?term=6317)(22.373) [FOXP3](http://www.ncbi.nlm.nih.gov/gene/?term=50943)(22.373)[CXCL13](http://www.ncbi.nlm.nih.gov/gene/?term=10563)(22.373)[ARID1A](http://www.ncbi.nlm.nih.gov/gene/?term=8289)(22.373)[FGF10](http://www.ncbi.nlm.nih.gov/gene/?term=2255)(22.373)[KCNB1](http://www.ncbi.nlm.nih.gov/gene/?term=3745)(22.373) [LEF1](http://www.ncbi.nlm.nih.gov/gene/?term=51176)(22.373)[PTK2B](http://www.ncbi.nlm.nih.gov/gene/?term=2185)(22.373)[LIG4](http://www.ncbi.nlm.nih.gov/gene/?term=3981)(22.373)[ADRBK1](http://www.ncbi.nlm.nih.gov/gene/?term=156)(22.373)[MAOB](http://www.ncbi.nlm.nih.gov/gene/?term=4129)(22.373) [SORCS3](http://www.ncbi.nlm.nih.gov/gene/?term=22986)(22.373)[ADRB1](http://www.ncbi.nlm.nih.gov/gene/?term=153)(22.373)[ADAM8](http://www.ncbi.nlm.nih.gov/gene/?term=101)(22.373)[VTI1A](http://www.ncbi.nlm.nih.gov/gene/?term=143187)(22.373)[SLC44A4](http://www.ncbi.nlm.nih.gov/gene/?term=80736)(22.373) [ADAP2](http://www.ncbi.nlm.nih.gov/gene/?term=55803)(22.373)[LRRC8A](http://www.ncbi.nlm.nih.gov/gene/?term=56262)(22.373)[FGFR2](http://www.ncbi.nlm.nih.gov/gene/?term=2263)(22.373)[AURKA](http://www.ncbi.nlm.nih.gov/gene/?term=6790)(22.373)[MCM3](http://www.ncbi.nlm.nih.gov/gene/?term=4172)(22.373) [VCP](http://www.ncbi.nlm.nih.gov/gene/?term=7415)(22.373)[GHRL](http://www.ncbi.nlm.nih.gov/gene/?term=51738)(22.373)[BCL11B](http://www.ncbi.nlm.nih.gov/gene/?term=64919)(22.373)[BCL2](http://www.ncbi.nlm.nih.gov/gene/?term=596)(22.373)[8-Mar](http://www.ncbi.nlm.nih.gov/gene/?term=220972)(22.373) [GAS6](http://www.ncbi.nlm.nih.gov/gene/?term=2621)(22.373)[C5](http://www.ncbi.nlm.nih.gov/gene/?term=727)(22.373)[SREBF1](http://www.ncbi.nlm.nih.gov/gene/?term=6720)(22.373)[DRD2](http://www.ncbi.nlm.nih.gov/gene/?term=1813)(22.373)[PLN](http://www.ncbi.nlm.nih.gov/gene/?term=5350)(22.373) [DNM3](http://www.ncbi.nlm.nih.gov/gene/?term=26052)(22.373)[CHRNA3](http://www.ncbi.nlm.nih.gov/gene/?term=1136)(22.373)[TCF3](http://www.ncbi.nlm.nih.gov/gene/?term=6929)(22.373)[SRC](http://www.ncbi.nlm.nih.gov/gene/?term=6714)(22.373)[LONP1](http://www.ncbi.nlm.nih.gov/gene/?term=9361)(22.373) [XCL1](http://www.ncbi.nlm.nih.gov/gene/?term=6375)(22.373)[HSP90AB1](http://www.ncbi.nlm.nih.gov/gene/?term=3326)(22.373)[HIBADH](http://www.ncbi.nlm.nih.gov/gene/?term=11112)(22.373)[STAP1](http://www.ncbi.nlm.nih.gov/gene/?term=26228)(22.373)[HRC](http://www.ncbi.nlm.nih.gov/gene/?term=3270)(22.373) [UTS2R](http://www.ncbi.nlm.nih.gov/gene/?term=2837)(22.373)[SLC6A4](http://www.ncbi.nlm.nih.gov/gene/?term=6532)(22.373)[HMGCR](http://www.ncbi.nlm.nih.gov/gene/?term=3156)(22.373)[HIPK2](http://www.ncbi.nlm.nih.gov/gene/?term=28996)(22.373)[PPP3CA](http://www.ncbi.nlm.nih.gov/gene/?term=5530)(22.373) [RAPGEF2](http://www.ncbi.nlm.nih.gov/gene/?term=9693)(22.373)[TET1](http://www.ncbi.nlm.nih.gov/gene/?term=80312)(22.373)[C2CD5](http://www.ncbi.nlm.nih.gov/gene/?term=9847)(22.373)[CSF2](http://www.ncbi.nlm.nih.gov/gene/?term=1437)(22.373)[TALDO1](http://www.ncbi.nlm.nih.gov/gene/?term=6888)(22.373) [BDKRB2](http://www.ncbi.nlm.nih.gov/gene/?term=624)(22.373)[KCNH2](http://www.ncbi.nlm.nih.gov/gene/?term=3757)(22.373)[MMP28](http://www.ncbi.nlm.nih.gov/gene/?term=79148)(22.373)[HIF1A](http://www.ncbi.nlm.nih.gov/gene/?term=3091)(22.373)[KIT](http://www.ncbi.nlm.nih.gov/gene/?term=3815)(22.373) [INS](http://www.ncbi.nlm.nih.gov/gene/?term=3630)(22.373)[MTOR](http://www.ncbi.nlm.nih.gov/gene/?term=2475)(22.373)[WNT10B](http://www.ncbi.nlm.nih.gov/gene/?term=7480)(22.373)[SLC18A3](http://www.ncbi.nlm.nih.gov/gene/?term=6572)(22.373)[ABCC4](http://www.ncbi.nlm.nih.gov/gene/?term=10257)(22.373) [BNIP3](http://www.ncbi.nlm.nih.gov/gene/?term=664)(22.373)[ZFPM1](http://www.ncbi.nlm.nih.gov/gene/?term=161882)(22.373)[GATA3](http://www.ncbi.nlm.nih.gov/gene/?term=2625)(22.373)[UBR5](http://www.ncbi.nlm.nih.gov/gene/?term=51366)(22.373)[AREG](http://www.ncbi.nlm.nih.gov/gene/?term=374)(22.373) [CRH](http://www.ncbi.nlm.nih.gov/gene/?term=1392)(22.373)[ZFP42](http://www.ncbi.nlm.nih.gov/gene/?term=132625)(22.373)[PIK3R6](http://www.ncbi.nlm.nih.gov/gene/?term=146850)(22.373)[PF4](http://www.ncbi.nlm.nih.gov/gene/?term=5196)(22.373)[ATP1A2](http://www.ncbi.nlm.nih.gov/gene/?term=477)(22.373) [AVPR2](http://www.ncbi.nlm.nih.gov/gene/?term=554)(22.373)[CAMK2D](http://www.ncbi.nlm.nih.gov/gene/?term=817)(22.373)[ADRA1B](http://www.ncbi.nlm.nih.gov/gene/?term=147)(22.373)[IL10](http://www.ncbi.nlm.nih.gov/gene/?term=3586)(22.373)[NEFL](http://www.ncbi.nlm.nih.gov/gene/?term=4747)(22.373) [UCN2](http://www.ncbi.nlm.nih.gov/gene/?term=90226)(22.373)[ATP1A1](http://www.ncbi.nlm.nih.gov/gene/?term=476)(22.373)[RYR3](http://www.ncbi.nlm.nih.gov/gene/?term=6263)(22.373)[PPP3R1](http://www.ncbi.nlm.nih.gov/gene/?term=5534)(22.373)[SPX](http://www.ncbi.nlm.nih.gov/gene/?term=80763)(22.373) [ATP2A1](http://www.ncbi.nlm.nih.gov/gene/?term=487)(22.373)[SMO](http://www.ncbi.nlm.nih.gov/gene/?term=6608)(22.373)[RAG1](http://www.ncbi.nlm.nih.gov/gene/?term=5896)(22.373)[RAB8B](http://www.ncbi.nlm.nih.gov/gene/?term=51762)(22.373)[DKK3](http://www.ncbi.nlm.nih.gov/gene/?term=27122)(22.373) [AHCYL1](http://www.ncbi.nlm.nih.gov/gene/?term=10768)(22.373)[AQP1](http://www.ncbi.nlm.nih.gov/gene/?term=358)(22.373)[TNFAIP3](http://www.ncbi.nlm.nih.gov/gene/?term=7128)(22.373)[TAC4](http://www.ncbi.nlm.nih.gov/gene/?term=255061)(22.373)[DPPA3](http://www.ncbi.nlm.nih.gov/gene/?term=359787)(22.373) [CASQ1](http://www.ncbi.nlm.nih.gov/gene/?term=844)(22.373)[FKBP1B](http://www.ncbi.nlm.nih.gov/gene/?term=2281)(22.373)[CDC42](http://www.ncbi.nlm.nih.gov/gene/?term=998)(22.373)[IL4](http://www.ncbi.nlm.nih.gov/gene/?term=3565)(22.373)[POLB](http://www.ncbi.nlm.nih.gov/gene/?term=5423)(22.373) [IDO1](http://www.ncbi.nlm.nih.gov/gene/?term=3620)(22.373)[ATP2B4](http://www.ncbi.nlm.nih.gov/gene/?term=493)(22.373)[CTNNB1](http://www.ncbi.nlm.nih.gov/gene/?term=1499)(22.373)[PGLS](http://www.ncbi.nlm.nih.gov/gene/?term=25796)(22.373)[SLC9A3R1](http://www.ncbi.nlm.nih.gov/gene/?term=9368)(22.373) [IGF2](http://www.ncbi.nlm.nih.gov/gene/?term=3481)(22.373)[HPS4](http://www.ncbi.nlm.nih.gov/gene/?term=89781)(22.373)[TKT](http://www.ncbi.nlm.nih.gov/gene/?term=7086)(22.373)[DRD1](http://www.ncbi.nlm.nih.gov/gene/?term=1812)(22.373)[ADAP1](http://www.ncbi.nlm.nih.gov/gene/?term=11033)(22.373) [EDA](http://www.ncbi.nlm.nih.gov/gene/?term=1896)(22.373)[RYR2](http://www.ncbi.nlm.nih.gov/gene/?term=6262)(22.373)[CD34](http://www.ncbi.nlm.nih.gov/gene/?term=947)(22.373)[HSP90AA1](http://www.ncbi.nlm.nih.gov/gene/?term=3320)(22.373)[LTA](http://www.ncbi.nlm.nih.gov/gene/?term=4049)(22.373) [SPR](http://www.ncbi.nlm.nih.gov/gene/?term=6697)(22.373)[GPER1](http://www.ncbi.nlm.nih.gov/gene/?term=2852)(22.373)[CBFA2T3](http://www.ncbi.nlm.nih.gov/gene/?term=863)(22.373)[CALHM1](http://www.ncbi.nlm.nih.gov/gene/?term=255022)(22.373)[WNT5A](http://www.ncbi.nlm.nih.gov/gene/?term=7474)(22.373) [SELP](http://www.ncbi.nlm.nih.gov/gene/?term=6403)(22.373)[XRCC6BP1](http://www.ncbi.nlm.nih.gov/gene/?term=91419)(22.373)[PARP10](http://www.ncbi.nlm.nih.gov/gene/?term=84875)(22.373)[HPRT1](http://www.ncbi.nlm.nih.gov/gene/?term=3251)(22.373)[AMICA1](http://www.ncbi.nlm.nih.gov/gene/?term=120425)(22.373) [TAS1R3](http://www.ncbi.nlm.nih.gov/gene/?term=83756)(22.373)[NOS1](http://www.ncbi.nlm.nih.gov/gene/?term=4842)(22.373)[TREM1](http://www.ncbi.nlm.nih.gov/gene/?term=54210)(22.373)[TAL1](http://www.ncbi.nlm.nih.gov/gene/?term=6886)(22.373)[ALAD](http://www.ncbi.nlm.nih.gov/gene/?term=210)(22.373) [PAWR](http://www.ncbi.nlm.nih.gov/gene/?term=5074)(22.373)[SOX9](http://www.ncbi.nlm.nih.gov/gene/?term=6662)(22.373)[BMP5](http://www.ncbi.nlm.nih.gov/gene/?term=653)(22.373)[PDGFB](http://www.ncbi.nlm.nih.gov/gene/?term=5155)(22.373)[RTN2](http://www.ncbi.nlm.nih.gov/gene/?term=6253)(22.373) [CD63](http://www.ncbi.nlm.nih.gov/gene/?term=967)(22.373)[CAMK2G](http://www.ncbi.nlm.nih.gov/gene/?term=818)(22.373)[LAMP2](http://www.ncbi.nlm.nih.gov/gene/?term=3920)(22.373)[NEFH](http://www.ncbi.nlm.nih.gov/gene/?term=4744)(22.373)[GLYR1](http://www.ncbi.nlm.nih.gov/gene/?term=84656)(22.373) [VDR](http://www.ncbi.nlm.nih.gov/gene/?term=7421)(22.373)[GNAS](http://www.ncbi.nlm.nih.gov/gene/?term=2778)(22.373)[NCBP2](http://www.ncbi.nlm.nih.gov/gene/?term=22916)(22.373)[TPM1](http://www.ncbi.nlm.nih.gov/gene/?term=7168)(22.373)[PIK3R5](http://www.ncbi.nlm.nih.gov/gene/?term=23533)(22.373) [PCSK6](http://www.ncbi.nlm.nih.gov/gene/?term=5046)(22.373)[TRIM24](http://www.ncbi.nlm.nih.gov/gene/?term=8805)(22.373)[REST](http://www.ncbi.nlm.nih.gov/gene/?term=5978)(22.373)[CRHR2](http://www.ncbi.nlm.nih.gov/gene/?term=1395)(22.373)[HCN2](http://www.ncbi.nlm.nih.gov/gene/?term=610)(22.373) [RAG2](http://www.ncbi.nlm.nih.gov/gene/?term=5897)(22.373)[NFX1](http://www.ncbi.nlm.nih.gov/gene/?term=4799)(22.373)[TAS1R2](http://www.ncbi.nlm.nih.gov/gene/?term=80834)(22.373) | |  |
| [Acetic Acid](http://www.megabionet.org/tcmid/ingredient/23287/) | This compound doesn't have any potential target with score larger than 20. | |  |
| [Glycitein](http://www.megabionet.org/tcmid/ingredient/8817/) | This compound doesn't have any potential target with score larger than 20. | |  |
| [Licoleafol](http://www.megabionet.org/tcmid/ingredient/12774/) | [SOAT1](http://www.ncbi.nlm.nih.gov/gene/?term=6646)(122.778)[MTTP](http://www.ncbi.nlm.nih.gov/gene/?term=4547)(122.778)[SOAT2](http://www.ncbi.nlm.nih.gov/gene/?term=8435)(122.778) | |  |
| [Octadecanol](http://www.megabionet.org/tcmid/ingredient/15940/) | [TRPV3](http://www.ncbi.nlm.nih.gov/gene/?term=162514)(122.778)[OPRK1](http://www.ncbi.nlm.nih.gov/gene/?term=4986)(122.778)[TRPM8](http://www.ncbi.nlm.nih.gov/gene/?term=79054)(122.778)[TRPA1](http://www.ncbi.nlm.nih.gov/gene/?term=8989)(122.778)[KCNK4](http://www.ncbi.nlm.nih.gov/gene/?term=50801)(55.444) [SCN11A](http://www.ncbi.nlm.nih.gov/gene/?term=11280)(48.000)[SCN2B](http://www.ncbi.nlm.nih.gov/gene/?term=6327)(48.000)[ABAT](http://www.ncbi.nlm.nih.gov/gene/?term=18)(48.000)[SCN1A](http://www.ncbi.nlm.nih.gov/gene/?term=6323)(48.000)[SCN3B](http://www.ncbi.nlm.nih.gov/gene/?term=55800)(48.000) [SCN3A](http://www.ncbi.nlm.nih.gov/gene/?term=6328)(48.000)[SCN7A](http://www.ncbi.nlm.nih.gov/gene/?term=6332)(48.000)[SCN5A](http://www.ncbi.nlm.nih.gov/gene/?term=6331)(48.000)[SCN10A](http://www.ncbi.nlm.nih.gov/gene/?term=6336)(48.000)[AKR1D1](http://www.ncbi.nlm.nih.gov/gene/?term=6718)(48.000) [ALDH5A1](http://www.ncbi.nlm.nih.gov/gene/?term=7915)(48.000)[SCN2A](http://www.ncbi.nlm.nih.gov/gene/?term=6326)(48.000)[HDAC9](http://www.ncbi.nlm.nih.gov/gene/?term=9734)(48.000)[TYR](http://www.ncbi.nlm.nih.gov/gene/?term=7299)(48.000)[SCN9A](http://www.ncbi.nlm.nih.gov/gene/?term=6335)(48.000) [SCN4A](http://www.ncbi.nlm.nih.gov/gene/?term=6329)(48.000)[SCN4B](http://www.ncbi.nlm.nih.gov/gene/?term=6330)(48.000)[SRD5A2](http://www.ncbi.nlm.nih.gov/gene/?term=6716)(48.000)[ACADSB](http://www.ncbi.nlm.nih.gov/gene/?term=36)(48.000)[SCN8A](http://www.ncbi.nlm.nih.gov/gene/?term=6334)(48.000) [SCN1B](http://www.ncbi.nlm.nih.gov/gene/?term=6324)(48.000)[OGDH](http://www.ncbi.nlm.nih.gov/gene/?term=4967)(48.000)[HDAC2](http://www.ncbi.nlm.nih.gov/gene/?term=3066)(48.000)[ACTN3](http://www.ncbi.nlm.nih.gov/gene/?term=89)(22.373)[TAC1](http://www.ncbi.nlm.nih.gov/gene/?term=6863)(22.373) [NGFR](http://www.ncbi.nlm.nih.gov/gene/?term=4804)(22.373)[ADRA2A](http://www.ncbi.nlm.nih.gov/gene/?term=150)(22.373)[TRPV1](http://www.ncbi.nlm.nih.gov/gene/?term=7442)(22.373)[FGF10](http://www.ncbi.nlm.nih.gov/gene/?term=2255)(22.373)[VCP](http://www.ncbi.nlm.nih.gov/gene/?term=7415)(22.373) [HIF1A](http://www.ncbi.nlm.nih.gov/gene/?term=3091)(22.373)[BNIP3](http://www.ncbi.nlm.nih.gov/gene/?term=664)(22.373)[CRH](http://www.ncbi.nlm.nih.gov/gene/?term=1392)(22.373)[NEFL](http://www.ncbi.nlm.nih.gov/gene/?term=4747)(22.373)[UCN2](http://www.ncbi.nlm.nih.gov/gene/?term=90226)(22.373) [SMO](http://www.ncbi.nlm.nih.gov/gene/?term=6608)(22.373)[TACR2](http://www.ncbi.nlm.nih.gov/gene/?term=6865)(22.373)[AQP1](http://www.ncbi.nlm.nih.gov/gene/?term=358)(22.373)[TAC4](http://www.ncbi.nlm.nih.gov/gene/?term=255061)(22.373)[CDH3](http://www.ncbi.nlm.nih.gov/gene/?term=1001)(22.373) [CBFA2T3](http://www.ncbi.nlm.nih.gov/gene/?term=863)(22.373)[NEFH](http://www.ncbi.nlm.nih.gov/gene/?term=4744)(22.373)[CRHR2](http://www.ncbi.nlm.nih.gov/gene/?term=1395)(22.373) | |  |
| [Vanillic Acid](http://www.megabionet.org/tcmid/ingredient/23214/) | This compound doesn't have any potential target with score larger than 20. | |  |
| [Tetrahydropalmatine](http://www.megabionet.org/tcmid/ingredient/21061/) | [ACHE](http://www.ncbi.nlm.nih.gov/gene/?term=43)(122.778)[CHRNA2](http://www.ncbi.nlm.nih.gov/gene/?term=1135)(122.778)[HTR3A](http://www.ncbi.nlm.nih.gov/gene/?term=3359)(122.778)[CHRM2](http://www.ncbi.nlm.nih.gov/gene/?term=1129)(122.778)[CYBA](http://www.ncbi.nlm.nih.gov/gene/?term=1535)(80.882) [CHRNA4](http://www.ncbi.nlm.nih.gov/gene/?term=1137)(80.882)[RAC2](http://www.ncbi.nlm.nih.gov/gene/?term=5880)(80.882)[RAC1](http://www.ncbi.nlm.nih.gov/gene/?term=5879)(80.882)[OPRD1](http://www.ncbi.nlm.nih.gov/gene/?term=4985)(80.882)[NCF4](http://www.ncbi.nlm.nih.gov/gene/?term=4689)(80.882) [CHRNA7](http://www.ncbi.nlm.nih.gov/gene/?term=1139)(80.882)[CYBB](http://www.ncbi.nlm.nih.gov/gene/?term=1536)(80.882)[NCF1](http://www.ncbi.nlm.nih.gov/gene/?term=653361)(80.882)[SIGMAR1](http://www.ncbi.nlm.nih.gov/gene/?term=10280)(80.882)[NCF2](http://www.ncbi.nlm.nih.gov/gene/?term=4688)(80.882) [OPRK1](http://www.ncbi.nlm.nih.gov/gene/?term=4986)(80.882)[CHRNA3](http://www.ncbi.nlm.nih.gov/gene/?term=1136)(80.882)[RAC3](http://www.ncbi.nlm.nih.gov/gene/?term=5881)(80.882)[CHRNB2](http://www.ncbi.nlm.nih.gov/gene/?term=1141)(80.882)[SLC6A4](http://www.ncbi.nlm.nih.gov/gene/?term=6532)(80.882) [OPRM1](http://www.ncbi.nlm.nih.gov/gene/?term=4988)(80.882)[PGRMC1](http://www.ncbi.nlm.nih.gov/gene/?term=10857)(80.882)[GRIN3A](http://www.ncbi.nlm.nih.gov/gene/?term=116443)(80.882)[CHRFAM7A](http://www.ncbi.nlm.nih.gov/gene/?term=89832)(80.882)[CHRNB4](http://www.ncbi.nlm.nih.gov/gene/?term=1143)(80.882) [SLC6A2](http://www.ncbi.nlm.nih.gov/gene/?term=6530)(80.882)[TLR4](http://www.ncbi.nlm.nih.gov/gene/?term=7099)(55.444)[ELANE](http://www.ncbi.nlm.nih.gov/gene/?term=1991)(55.444)[CTSG](http://www.ncbi.nlm.nih.gov/gene/?term=1511)(55.444)[SLC18A1](http://www.ncbi.nlm.nih.gov/gene/?term=6570)(55.444) [CEND1](http://www.ncbi.nlm.nih.gov/gene/?term=51286)(55.444)[SLC22A1](http://www.ncbi.nlm.nih.gov/gene/?term=6580)(55.444)[ADRA1B](http://www.ncbi.nlm.nih.gov/gene/?term=147)(55.444)[WLS](http://www.ncbi.nlm.nih.gov/gene/?term=79971)(55.444)[CHRNA9](http://www.ncbi.nlm.nih.gov/gene/?term=55584)(55.444) [CHRM3](http://www.ncbi.nlm.nih.gov/gene/?term=1131)(48.000)[DRD2](http://www.ncbi.nlm.nih.gov/gene/?term=1813)(48.000)[SLC18A2](http://www.ncbi.nlm.nih.gov/gene/?term=6571)(48.000)[BCHE](http://www.ncbi.nlm.nih.gov/gene/?term=590)(48.000)[ADRA1A](http://www.ncbi.nlm.nih.gov/gene/?term=148)(23.000) [HTR2A](http://www.ncbi.nlm.nih.gov/gene/?term=3356)(23.000)[CHRM1](http://www.ncbi.nlm.nih.gov/gene/?term=1128)(23.000)[HTR2B](http://www.ncbi.nlm.nih.gov/gene/?term=3357)(23.000)[ADRA2A](http://www.ncbi.nlm.nih.gov/gene/?term=150)(23.000)[HTR6](http://www.ncbi.nlm.nih.gov/gene/?term=3362)(23.000) [HTR2C](http://www.ncbi.nlm.nih.gov/gene/?term=3358)(23.000)[ADRA1D](http://www.ncbi.nlm.nih.gov/gene/?term=146)(23.000)[CHRM5](http://www.ncbi.nlm.nih.gov/gene/?term=1133)(23.000)[HRH4](http://www.ncbi.nlm.nih.gov/gene/?term=59340)(23.000)[HRH1](http://www.ncbi.nlm.nih.gov/gene/?term=3269)(23.000) [KCNH2](http://www.ncbi.nlm.nih.gov/gene/?term=3757)(23.000)[HRH2](http://www.ncbi.nlm.nih.gov/gene/?term=3274)(23.000)[HTR1A](http://www.ncbi.nlm.nih.gov/gene/?term=3350)(23.000)[ADRA2C](http://www.ncbi.nlm.nih.gov/gene/?term=152)(23.000)[CHRM4](http://www.ncbi.nlm.nih.gov/gene/?term=1132)(23.000) [ADRA2B](http://www.ncbi.nlm.nih.gov/gene/?term=151)(23.000)[ACTN3](http://www.ncbi.nlm.nih.gov/gene/?term=89)(22.373)[GPM6B](http://www.ncbi.nlm.nih.gov/gene/?term=2824)(22.373)[HMOX1](http://www.ncbi.nlm.nih.gov/gene/?term=3162)(22.373)[TAC1](http://www.ncbi.nlm.nih.gov/gene/?term=6863)(22.373) [MC2R](http://www.ncbi.nlm.nih.gov/gene/?term=4158)(22.373)[SLITRK6](http://www.ncbi.nlm.nih.gov/gene/?term=84189)(22.373)[UTS2](http://www.ncbi.nlm.nih.gov/gene/?term=10911)(22.373)[NAV2](http://www.ncbi.nlm.nih.gov/gene/?term=89797)(22.373)[PDE3A](http://www.ncbi.nlm.nih.gov/gene/?term=5139)(22.373) [SCT](http://www.ncbi.nlm.nih.gov/gene/?term=6343)(22.373)[TUSC2](http://www.ncbi.nlm.nih.gov/gene/?term=11334)(22.373)[RHOA](http://www.ncbi.nlm.nih.gov/gene/?term=387)(22.373)[AVPR1A](http://www.ncbi.nlm.nih.gov/gene/?term=552)(22.373)[ADORA2A](http://www.ncbi.nlm.nih.gov/gene/?term=135)(22.373) [HCN4](http://www.ncbi.nlm.nih.gov/gene/?term=10021)(22.373)[PHKG2](http://www.ncbi.nlm.nih.gov/gene/?term=5261)(22.373)[OXT](http://www.ncbi.nlm.nih.gov/gene/?term=5020)(22.373)[CACNA1A](http://www.ncbi.nlm.nih.gov/gene/?term=773)(22.373)[CCL5](http://www.ncbi.nlm.nih.gov/gene/?term=6352)(22.373) [APLP1](http://www.ncbi.nlm.nih.gov/gene/?term=333)(22.373)[LAMP1](http://www.ncbi.nlm.nih.gov/gene/?term=3916)(22.373)[PPP1R9B](http://www.ncbi.nlm.nih.gov/gene/?term=84687)(22.373)[YWHAE](http://www.ncbi.nlm.nih.gov/gene/?term=7531)(22.373)[SUMO1](http://www.ncbi.nlm.nih.gov/gene/?term=7341)(22.373) [HTR1B](http://www.ncbi.nlm.nih.gov/gene/?term=3351)(22.373)[CNR2](http://www.ncbi.nlm.nih.gov/gene/?term=1269)(22.373)[NOXA1](http://www.ncbi.nlm.nih.gov/gene/?term=10811)(22.373)[MC1R](http://www.ncbi.nlm.nih.gov/gene/?term=4157)(22.373)[MC4R](http://www.ncbi.nlm.nih.gov/gene/?term=4160)(22.373) [IL1B](http://www.ncbi.nlm.nih.gov/gene/?term=3553)(22.373)[AGT](http://www.ncbi.nlm.nih.gov/gene/?term=183)(22.373)[CRHBP](http://www.ncbi.nlm.nih.gov/gene/?term=1393)(22.373)[F2R](http://www.ncbi.nlm.nih.gov/gene/?term=2149)(22.373)[MTNR1B](http://www.ncbi.nlm.nih.gov/gene/?term=4544)(22.373) [FGF10](http://www.ncbi.nlm.nih.gov/gene/?term=2255)(22.373)[EGR1](http://www.ncbi.nlm.nih.gov/gene/?term=1958)(22.373)[NOXO1](http://www.ncbi.nlm.nih.gov/gene/?term=124056)(22.373)[ADAM8](http://www.ncbi.nlm.nih.gov/gene/?term=101)(22.373)[SLC44A4](http://www.ncbi.nlm.nih.gov/gene/?term=80736)(22.373) [FLOT1](http://www.ncbi.nlm.nih.gov/gene/?term=10211)(22.373)[NPY5R](http://www.ncbi.nlm.nih.gov/gene/?term=4889)(22.373)[VCP](http://www.ncbi.nlm.nih.gov/gene/?term=7415)(22.373)[GHRL](http://www.ncbi.nlm.nih.gov/gene/?term=51738)(22.373)[TBR1](http://www.ncbi.nlm.nih.gov/gene/?term=10716)(22.373) [PTPRO](http://www.ncbi.nlm.nih.gov/gene/?term=5800)(22.373)[OXTR](http://www.ncbi.nlm.nih.gov/gene/?term=5021)(22.373)[PAX2](http://www.ncbi.nlm.nih.gov/gene/?term=5076)(22.373)[CACNA1C](http://www.ncbi.nlm.nih.gov/gene/?term=775)(22.373)[F2RL1](http://www.ncbi.nlm.nih.gov/gene/?term=2150)(22.373) [UTS2R](http://www.ncbi.nlm.nih.gov/gene/?term=2837)(22.373)[ACVR2A](http://www.ncbi.nlm.nih.gov/gene/?term=92)(22.373)[RAPGEF2](http://www.ncbi.nlm.nih.gov/gene/?term=9693)(22.373)[TIRAP](http://www.ncbi.nlm.nih.gov/gene/?term=114609)(22.373)[AIF1](http://www.ncbi.nlm.nih.gov/gene/?term=199)(22.373) [GHRH](http://www.ncbi.nlm.nih.gov/gene/?term=2691)(22.373)[HCRT](http://www.ncbi.nlm.nih.gov/gene/?term=3060)(22.373)[RAB3B](http://www.ncbi.nlm.nih.gov/gene/?term=5865)(22.373)[CNR1](http://www.ncbi.nlm.nih.gov/gene/?term=1268)(22.373)[HIF1A](http://www.ncbi.nlm.nih.gov/gene/?term=3091)(22.373) [INS](http://www.ncbi.nlm.nih.gov/gene/?term=3630)(22.373)[ACE](http://www.ncbi.nlm.nih.gov/gene/?term=1636)(22.373)[SLC18A3](http://www.ncbi.nlm.nih.gov/gene/?term=6572)(22.373)[BNIP3](http://www.ncbi.nlm.nih.gov/gene/?term=664)(22.373)[DRD4](http://www.ncbi.nlm.nih.gov/gene/?term=1815)(22.373) [CRH](http://www.ncbi.nlm.nih.gov/gene/?term=1392)(22.373)[GLI3](http://www.ncbi.nlm.nih.gov/gene/?term=2737)(22.373)[PLXNB3](http://www.ncbi.nlm.nih.gov/gene/?term=5365)(22.373)[CAV3](http://www.ncbi.nlm.nih.gov/gene/?term=859)(22.373)[NEFL](http://www.ncbi.nlm.nih.gov/gene/?term=4747)(22.373) [UCN2](http://www.ncbi.nlm.nih.gov/gene/?term=90226)(22.373)[IL6](http://www.ncbi.nlm.nih.gov/gene/?term=3569)(22.373)[RNF207](http://www.ncbi.nlm.nih.gov/gene/?term=388591)(22.373)[TACR2](http://www.ncbi.nlm.nih.gov/gene/?term=6865)(22.373)[PTGS2](http://www.ncbi.nlm.nih.gov/gene/?term=5743)(22.373) [NRP1](http://www.ncbi.nlm.nih.gov/gene/?term=8829)(22.373)[NPPA](http://www.ncbi.nlm.nih.gov/gene/?term=4878)(22.373)[MC5R](http://www.ncbi.nlm.nih.gov/gene/?term=4161)(22.373)[AQP1](http://www.ncbi.nlm.nih.gov/gene/?term=358)(22.373)[MC3R](http://www.ncbi.nlm.nih.gov/gene/?term=4159)(22.373) [TAC4](http://www.ncbi.nlm.nih.gov/gene/?term=255061)(22.373)[TNR](http://www.ncbi.nlm.nih.gov/gene/?term=7143)(22.373)[NOS3](http://www.ncbi.nlm.nih.gov/gene/?term=4846)(22.373)[EPHB1](http://www.ncbi.nlm.nih.gov/gene/?term=2047)(22.373)[SNCA](http://www.ncbi.nlm.nih.gov/gene/?term=6622)(22.373) [DRD1](http://www.ncbi.nlm.nih.gov/gene/?term=1812)(22.373)[PDE2A](http://www.ncbi.nlm.nih.gov/gene/?term=5138)(22.373)[CD34](http://www.ncbi.nlm.nih.gov/gene/?term=947)(22.373)[KCNMA1](http://www.ncbi.nlm.nih.gov/gene/?term=3778)(22.373)[POU4F3](http://www.ncbi.nlm.nih.gov/gene/?term=5459)(22.373) [CBFA2T3](http://www.ncbi.nlm.nih.gov/gene/?term=863)(22.373)[CALCA](http://www.ncbi.nlm.nih.gov/gene/?term=796)(22.373)[PINK1](http://www.ncbi.nlm.nih.gov/gene/?term=65018)(22.373)[NOS1](http://www.ncbi.nlm.nih.gov/gene/?term=4842)(22.373)[TREM1](http://www.ncbi.nlm.nih.gov/gene/?term=54210)(22.373) [GJA5](http://www.ncbi.nlm.nih.gov/gene/?term=2702)(22.373)[PDGFB](http://www.ncbi.nlm.nih.gov/gene/?term=5155)(22.373)[CARTPT](http://www.ncbi.nlm.nih.gov/gene/?term=9607)(22.373)[NEFH](http://www.ncbi.nlm.nih.gov/gene/?term=4744)(22.373)[ATP8B1](http://www.ncbi.nlm.nih.gov/gene/?term=5205)(22.373) [KISS1](http://www.ncbi.nlm.nih.gov/gene/?term=3814)(22.373)[GRM7](http://www.ncbi.nlm.nih.gov/gene/?term=2917)(22.373)[FOS](http://www.ncbi.nlm.nih.gov/gene/?term=2353)(22.373)[CRHR2](http://www.ncbi.nlm.nih.gov/gene/?term=1395)(22.373)[GRIN1](http://www.ncbi.nlm.nih.gov/gene/?term=2902)(22.373) [HCN2](http://www.ncbi.nlm.nih.gov/gene/?term=610)(22.373) | |  |
